# PDB-Explorer: a web-based interactive map of the protein data bank in shape space

**DOI:** 10.1186/s12859-015-0776-9

**Published:** 2015-10-23

**Authors:** Xian Jin, Mahendra Awale, Michaël Zasso, Daniel Kostro, Luc Patiny, Jean-Louis Reymond

**Affiliations:** 1 Department of Chemistry and Biochemistry, University of Berne, Freiestrasse 3, 3012 Berne, Switzerland; 2 Ecole Polytechnique Fédérale de Lausanne (EPFL), Institute of Chemical Sciences and Engineering (ISIC), Lausanne, 1015 Switzerland

**Keywords:** Protein data bank, 3D-fingerprint, Visualization, Chemical space, Molecular shape

## Abstract

**Background:**

The RCSB Protein Data Bank (PDB) provides public access to experimentally determined 3D-structures of biological macromolecules (proteins, peptides and nucleic acids). While various tools are available to explore the PDB, options to access the global structural diversity of the entire PDB and to perceive relationships between PDB structures remain very limited.

**Methods:**

A 136-dimensional atom pair 3D-fingerprint for proteins (3DP) counting categorized atom pairs at increasing through-space distances was designed to represent the molecular shape of PDB-entries. Nearest neighbor searches examples were reported exemplifying the ability of 3DP-similarity to identify closely related biomolecules from small peptides to enzyme and large multiprotein complexes such as virus particles. The principle component analysis was used to obtain the visualization of PDB in 3DP-space.

**Results:**

The 3DP property space groups proteins and protein assemblies according to their 3D-shape similarity, yet shows exquisite ability to distinguish between closely related structures. An interactive website called PDB-Explorer is presented featuring a color-coded interactive map of PDB in 3DP-space. Each pixel of the map contains one or more PDB-entries which are directly visualized as ribbon diagrams when the pixel is selected. The PDB-Explorer website allows performing 3DP-nearest neighbor searches of any PDB-entry or of any structure uploaded as protein-type PDB file. All functionalities on the website are implemented in JavaScript in a platform-independent manner and draw data from a server that is updated daily with the latest PDB additions, ensuring complete and up-to-date coverage. The essentially instantaneous 3DP-similarity search with the PDB-Explorer provides results comparable to those of much slower 3D-alignment algorithms, and automatically clusters proteins from the same superfamilies in tight groups.

**Conclusion:**

A chemical space classification of PDB based on molecular shape was obtained using a new atom-pair 3D-fingerprint for proteins and implemented in a web-based database exploration tool comprising an interactive color-coded map of the PDB chemical space and a nearest neighbor search tool. The PDB-Explorer website is freely available at www.cheminfo.org/pdbexplorer and represents an unprecedented opportunity to interactively visualize and explore the structural diversity of the PDB.

**ᅟ:**

**Graphical abstract:**
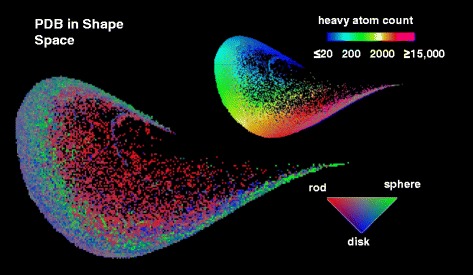
ᅟMaps of PDB in 3DP-space color-coded by heavy atom count and shape.

**Electronic supplementary material:**

The online version of this article (doi:10.1186/s12859-015-0776-9) contains supplementary material, which is available to authorized users.

## Background

One of the striking features of biomolecules is their extremely large diversity spanning from small organic molecules such as metabolites and drugs to large supramolecular complexes such as the ribosome or viral particles. A vast amount of knowledge about these biomolecules has been collected in various public databases, in particular the Protein Data Bank (PDB) which collects over 100,000 different 3-dimensional (3D) structures of biological macromolecules determined by X-ray crystallography, NMR spectroscopy and electron microscopy [1–4]. Despite of this vast amount of information, the overall structural diversity available in the PDB is difficult to perceive. Indeed various tools are available to search the PDB for analogs of specific proteins according to similarities in evolutionary history, sequences, secondary structures and subdomains [5–12]. In the case of 3D-SURFER [13, 14] the PDB is classified according to similarities in protein surface allowing to search for shape analogs among PDB-entries. The CATH [7, 8] contains a subset of the PDB which is directly visible via an overview interface using a hierarchical classification by structural domains. However none of these interfaces provides a direct, global yet comprehensive overview of the PDB irrespective of a specific query, which would be desirable to understand its overall contents.

Herein we report a new exploration tool for the PDB called PDB-Explorer which addresses the need for a global perception of the database by giving direct access to all PDB-entries via an interactive color-coded map representing its entire contents in molecular shape space. This application follows the principle of our recently reported MQN-mapplet and SMIfp-mapplet applications designed to visualize the chemical space of small organic molecules [15–18]. Each individual PDB-entry is placed on the map of the PDB-Explorer according to its 3D-shape as encoded by a new fingerprint called 3DP featuring a generalized version of our recently reported 3D-atom pair fingerprints for small molecules [19, 20]. The PDB-Explorer provides an unprecedented global view of the PDB allowing a detailed exploration of its entire content in a curiosity-driven manner with or without specific queries. This tool is freely available at www.cheminfo.org/pdbexplorer and should greatly facilitate the perception and understanding of the overall diversity of proteins and biological assemblies available in the PDB.

## Methods

### Database selection

The X-ray structures in PDB-Explorer were downloaded from http://www.rcsb.org. The water molecules and hydrogen atoms of each PDB molecule were removed at the beginning. If the number of atoms assigned as “HETATM” occupied more than 20 % of total heavy atom count, this molecule was not included in the database.

### 3D protein atom-pair fingerprint (3DP)

Atoms belonging to the biological assembly defined by the authors are considered, discarding all atoms marked “HETATM” except for those belonging to unusual residues in the sequence (such as phosphorylated residues). 3DP classifies the considered atoms into four different categories: 1) all atoms: all non-hydrogen atoms; 2) positively charged atoms: amino group of lysine in the terminal zeta position or ζ-carbon atom of arginine; 3) negatively charged atom: γ-carbon of aspartic acid or δ-carbon of glutamic acid, phosphorus atoms of DNA, RNA, and phosphate groups on amino acid residues; 4) hydrophobic atoms: all carbon atoms with covalent bonds to other carbon atoms or hydrogen atoms. The molecule is placed into a 3D grid box whose size is determined by the longest atom pair distances on the orthogonal principal axes of the molecule. The box is divided into 12 × 12 × 12 small boxes. In each box category sum atoms are placed at the geometric center of category atoms in that box. All pairs of category sum atoms in the molecule are converted to a Gaussian function centered on the sum atom pair distance d_j_ with a width of 0.18× d_j_, and maximum amplitude equal to the product of the number of atoms composing each of the two category sum atoms composing the pair. All atom pairs Gaussians are added for each category and sampled at 1.45, 1.72, 2.02, 2.39, 2.82, 3.33, 3.93, 4.63, 5.47, 6.45, 7.61, 8.98, 10.60, 12.50, 14.76, 17.41, 20.55, 24.24, 28.61, 33.76, 39.83, 47, 55.46, 65.45, 77.23, 91.23, 107.53, 126.88, 149.72, 176.67, 208.47, 246, 290.28 and 342.53 Å (34 bits, Eq. 1). The 34 bit values in each category are divided by the category atom count^^1.5^, and the resulting ratios are expressed in percent rounded to unity. The 3DP calculation for each bit is described in Eq. 2. The bit values from hydrophobic atom pairs are finally multiplied by 2 and the bit values from positive and negative charged atom pairs are multiplied by 5. The 3DP-fingerprint calculation is written in JavaScript and performed on the user machine for user-defined structures.

1 $$ bi{t}_{i+1} = bi{t}_i\times 1.18,\kern0.5em i=1,2,\cdots, 34 $$

2 $$ \begin{array}{cc}\hfill 3DP\left( bi{t}_i\right)=\left(\left({\displaystyle \sum_{j=1}^m}{e}^{-\frac{bi{t}_i-{d}_j}{2\times {\left({d}_j\times 0.18\right)}^2}}\right)/{n}^{1.5}\right)\times 100,\hfill & \hfill \begin{array}{l}\mathrm{d}:\ \mathrm{atom}\ \mathrm{pair}\ \mathrm{d}\mathrm{istance}\hfill \\ {}\mathrm{m}:\mathrm{number}\ \mathrm{of}\ \mathrm{atom}\ \mathrm{pair}\mathrm{s}\hfill \\ {}\mathrm{n}:\mathrm{category}\ \mathrm{atom}\ \mathrm{count}\hfill \end{array}\hfill \end{array} $$

City-Block Distance (CBD): The CBD between two molecules, A and B, with 136 dimensions of 3DP is calculated as shown in Eq. 3:

3 $$ {\mathrm{CBD}}_{A,\kern0.5em B} = {\displaystyle \sum_{i=1}^{136}}\left|{A}_i-{B}_i\right| $$

### 3DP-similarity fingerprint

The 3DP-fingerprint was transformed into a 200-dimensional 3DP-similarity fingerprint (3DPs) by calculating the similarity value of each of the PDB-entry in the database relative to 200 randomly selected PDB molecules. Similarity values S_3DP_ were calculated from the city-block distance CBD_3DP_ as shown in Eq. 4, with X = median city-block distance observed for distribution of CBDs computed for 1 M random pairs of molecules within the database:

4 $$ {\mathrm{S}}_{3\mathrm{D}\mathrm{P}}=\mathrm{X}/\left({\mathrm{CBD}}_{3\mathrm{D}\mathrm{P}}+\mathrm{X}\right) $$

### Principal component analysis (PCA)

The PCA calculation used the source code from Java program developed based on the tutorial of Lindsay I. Smith (http://www.cs.otago.ac.nz/cosc453/student_tutorials/principal_components.pdf). The Java source code uses mathematical functions from the JSci (A science API for Java: http://jsci.sourceforge.net/) library.

### PDB-maps generation and color-coding

PCA of the 3DP-similarity fingerprint space of PDB database was carried out, and the PC-1 and PC-2 values were computed for each molecule in database. The PC-1 and PC-2 values were binned onto the 2D-grid of size 300 × 300 using the same absolute bin size on the PC-1 and PC-2. The largest (PC_max_) and smallest (PC_min_) PC values appearing in the PC-1 or PC-2 values were used to define the value range ΔPC = PC_max_ − PC_min_ and set the binning scale as ΔPC/300. Afterwards, each molecule was assigned to a point (or bin) on this 2D-grid.

The following molecular properties were computed at each grid point: HAC, percentage of positive, negative, and hydrophobic atoms, and molecular volume occupancy (mvo). The molecular volume occupancy (mvo) describing the compactness of a molecule was computed with the following formula (Eq. 5):

5 $$ \begin{array}{l}\begin{array}{cc}\hfill {O}_{x,y,z} = \left[\frac{1}{n}{\displaystyle \sum_{i=1}^n}{X}_i,\ \frac{1}{n}{\displaystyle \sum_{i=1}^n}{Y}_i,\left.\frac{1}{n}{\displaystyle \sum_{i=1}^n}{Z}_i\right]\right.,\hfill & \hfill \begin{array}{l}\mathrm{O}:\ \mathrm{g}\mathrm{eometry}\ \mathrm{center}\ \mathrm{o}\mathrm{f}\ \mathrm{molecule}\hfill \\ {}\mathrm{n}:\ \mathrm{heavy}\ \mathrm{atom}\ \mathrm{count}\hfill \\ {} Xi,\ \mathrm{Y}i,\ \mathrm{Z}i:\ \mathrm{atom}\ \mathrm{coordinates}\hfill \end{array}\hfill \end{array}\\ {}\begin{array}{cc}\hfill {d}_{avg}=\frac{1}{n}{\displaystyle \sum_{i=1}^n}\sqrt{\left({\left({X}_i-{O}_x\right)}^2+{\left({Y}_i-{O}_y\right)}^2+{\left({Z}_i-{O}_z\right)}^2\right)},\hfill & \hfill \begin{array}{c}\hfill d\mathrm{a}\mathrm{v}\mathrm{g}:\ \mathrm{average}\ \mathrm{distance}\ \mathrm{t}\mathrm{o}\hfill \\ {}\hfill \mathrm{the}\ \mathrm{g}\mathrm{eometry}\ \mathrm{center}\hfill \end{array}\hfill \end{array}\\ {}mvo = \raisebox{1ex}{$\left(\frac{4}{3}\times \pi \times {d_{avg}}^3\right)$}\!\left/ \!\raisebox{-1ex}{$n$}\right.\end{array} $$

Each of the grid point was color coded according to the average and standard deviation of the molecular property at that grid point using the Hue-Saturation-Lightness (HSL) color space, with the Hue value (blue-cyan-green-yellow-red-magenta) representing the average value and the Saturation (fading to grey) representing the standard deviation.

The calculation of normalized principal moment of inertia (nPMI1, nPMI2) was implemented by an in-house Java program written based on the work from Sauer and Schwarz [21]. The position in the (nPMI1, nPMI2) triangle was color coded using the RGB color space assigning the distance to each triangle summit as the relative R, G, and B values.

### PDB-Explorer

PDB- Explorer is a web-based application for the interactive visualization of chemical space of the PDB. The interface of the application was written in JavaScript. A copy of the PDB database has been created and a ribbon image for the structure of each PDB-entry has been generated. The dataset is updated daily based on the project (http://github.com/cheminfo/pdb-database). Free access is provided to the 3DP calculation (http://github.com/cheminfo/pdb-map) and the application package (www.cheminfo.org/pdbexplorer).

## Results and discussion

### Design of the protein shape fingerprint 3DP

In view of a global analysis of the PDB we set out to identify a fingerprint encoding the 3D-structure of biomacromolecules since it is known to play an important role in their biological function, their interaction with other molecules, [22–25] and their evolution [26–29]. While 3D-SURFER uses a 121-dimensional scalar fingerprint to encode the shape of the molecular surface of proteins using 3D Zernike descriptors, [13, 14] we searched for a simpler yet more detailed encoding considering not only the shape of the molecular surface, but also the atom types and the internal structure of the protein. Inspired by the concept of atom-pair fingerprints proposed by Carhart, [30] Sheridan [31] and Schneider [32] to encode pharmacophores in small organic molecules, we recently reported a detailed analysis establishing the suitability of atom-pair fingerprints for 3D-shape and pharmacophore similarity searches in very large databases such as ChEMBL [33] and ZINC [34] using both topological distances read from the 2D-structures [35] and through-space distance read from the 3D-structures [20]. While topological distances cannot be extracted easily from the structures of macromolecules or even do not exist within non-covalent assemblies, an atom-pair analysis of macromolecules should be possible using through-space distances which can be directly computed from the atomic coordinates available in each pdb file. An atom pair 3D-fingerprint, here called 3DP, was therefore envisioned to encode 3D-structures in the PDB.

**Fig. 1:**
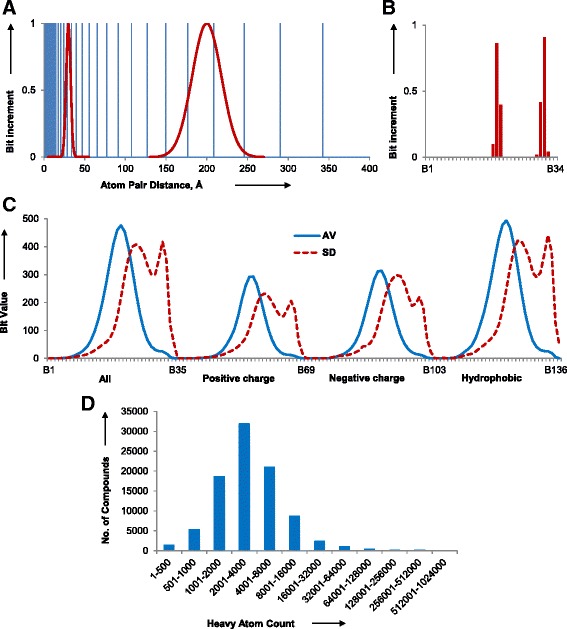
3DP fingerprint design. **a** 34 sampling values between 1.45 and 400 Å (*blue vertical bars*) and example Gaussian corresponding to two atom pair distances (*red line*). **b** Sampling of bit values of B1–B34 for the atom pairs at 30 and 200 Å from the Gaussian functions in **a**. **c** Average (AV, *blue continuous line*) and standard deviation (SD, *red doted line*) of bit values of 3DP for all biological assemblies in the PDB. **d** Distribution of heavy atom count (HAC) values in the PDB. The analysis is based on 91,223 X-ray structures downloaded from the PDB in September 2014, considering in each case the biological assembly as defined by the authors

The 3DP fingerprint was designed to encode the 3D structure of proteins and other biomacromolecules in PDB considering the biological assembly as defined by the authors in each entry. Following our previous approach for small molecules, each atom pair distance was converted to a Gaussian centered on the atom pair distance with a width of 18 % of the distance itself, and the Gaussian was sampled at 34 values between 1.45 Å and 342.53 Å covering all atom pair distances present in PDB (Fig. 1a). For each of the 34 values increments were summed across all atom pairs. The 34 resulting sums were normalized to the heavy atom count to the power of 1.5 (HAC^1.5^) to reduce sensitivity to size (Fig. 1b). The 34 values were computed separately for four different atom categories, using the corresponding sum of category atoms as HAC for normalization. These four categories considered as important for macromolecular properties comprised 1) all atoms; 2) positive charges: lysine and arginine side chains; 3) negative charges: aspartate and glutamate side chains, phosphate groups; 4) hydrophobic atoms, defined here as carbon atoms with covalent bonds only to C or H atoms. Due to the smaller number of charged and hydrophobic atoms compared to all atoms the bit values in the corresponding categories were on average much smaller and were therefore multiplied by 5 for charged atom categories and by 2 for the hydrophobic atom category. The final bit values were expressed in percent and rounded to the integer value. The four combined sets of normalized, rounded values formed the 136-dimensional 3DP fingerprint.

Because most PDB-entries contain thousands of atoms which would require explicit calculation of many millions of atom pair distances (Fig. 1d), the 3DP calculation was simplified by computing the fingerprint using formal “sum atoms”. A maximum of 1728 sum atoms (resulting in a maximum of 1.5 million sum atom pairs) were defined for each PDB-entry by fitting its biological unit into a 12×12×12 grid, using the average coordinates of each atom category within each box as the sum atom coordinates and the number of category atoms within this box as its relative weight. 3DP bit values computed with sum atoms were essentially indistinguishable from those computed with actual atoms.

The 3DP calculation was performed 91,223 X-ray structures downloaded from the PDB in September 2014 (Additional file 1: Supplement 1), considering in each case the biological assembly as defined by the authors [36]. The 3DP fingerprint had a high resolution, with 99.99 % of PDB molecules having unique 3DP fingerprints. The average bit values peaked at 33.76 Å (B20, B122) for all atoms and hydrophobic atom pairs and at 39.83 Å (B55, B89) for positive and negative charge atom pairs, while the corresponding standard deviation covered almost the entire bit value range (Fig. 1c).

### 3DP encodes protein conformations

**Fig. 2:**
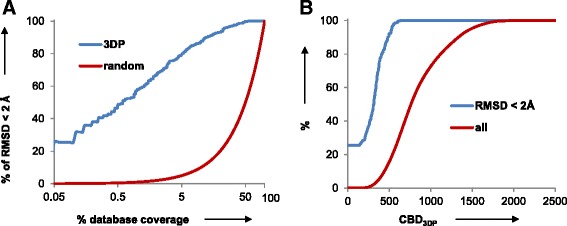
Retrieval of conformer analogs from the MD trajectory of a 24 residue peptide by 3DP similarity. **a** ROC curve for retrieving structures with RMSD < 2 Å, relative the last frame in a 50 ns MD simulation taken as reference, by 3DP similarity (*blue*) and by random selection (*red*), averaged over 50 different MD simulations. **b** Recovery of structures with RMSD < 2 Å to the reference (*blue*) and all structures (*red*) as function of CBD_3DP_ from the reference. The structure alignment and RMSD calculation of all heavy atoms were carried out with the AMBER12 package. The sequence of the 24-mer peptide is MKKRLAYAIIQFLHDQLRHGGLSS

The 3DP fingerprint had a remarkable ability to precisely encode the shape of proteins, as evidenced by investigating the correlation between 3DP similarity and the root mean square deviation (RMSD) [37, 38] in different conformers of the same molecule, as presented with the following three test cases. As a first test case a random coil 24-mer peptide was obtained from PDB-entry 4GOF and simulated using the ff99SB force field from the AMBER12 package, [39] with 1 ns simulated annealing followed by 50 ns free simulation in water solvent, generating a large variety of conformers. The simulation was repeated 50 times, and in each case the last structure was selected as reference. Its conformer analogs were defined as all conformers with RMSD lower than 2 Å to that reference, which comprised up to 136 conformers that were always the last series of frames in the MD run. Retrieval of these conformers from the 2000 conformers of the corresponding trajectory was then performed by sorting using the city-block distance CBD_3DP_ as similarity measure. The recovery was excellent, with an area under the curve (AUC) of 95.85 % for average receiver operator characteristic (ROC) curve, showing that 3DP indeed provided a good encoding of molecular shape in terms of similarity searching (Fig. 2a). All RMSD analogs were found within CBD_3DP_ < 519, while the average distance of these RMSD analogs from the query conformer was 264 and the largest distance was 2164 (Fig. 2b).

**Fig. 3:**
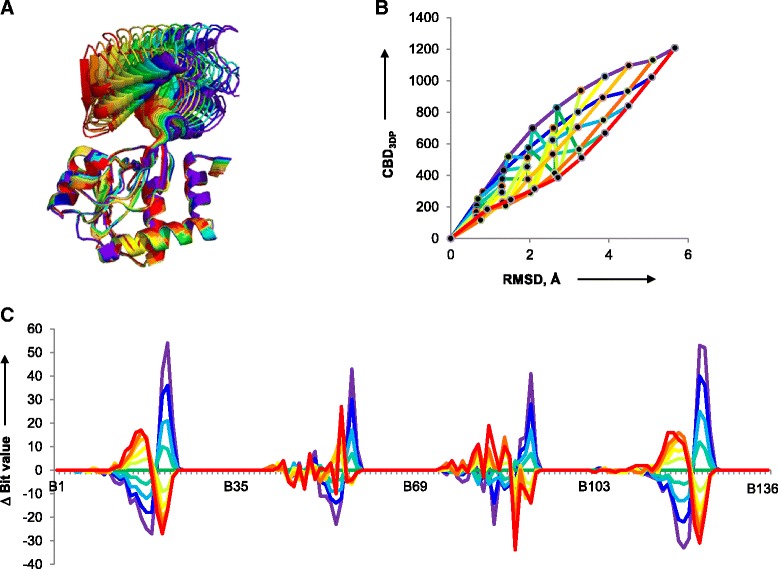
Correlation between RMSD and CBD_3DP_ in conformers of the glutamine binding protein. **a** Overlay of 10 structures from domain movement of glutamine binding protein taken from the Protein Motion Database. The initial structure is PDB-entry 1GGG (*purple*) and last structure is PDB-entry 1WDN (*red*). **b** Correlation between RMSD from all heavy atom alignment and CBD_3DP_. Each *line* indicates different reference structure. *Purple*: 1st (1GGG); *Blue*: 2nd; *Cyan*: 3rd; *Lime Green*: 4th; *Green*: 5th; *Light green*: 6th; *Yellow*: 7th; *Yellow orange*: 8th; *Orange*: 9th; *Red*: 10th (1WDN). **c** Deviation of bit values to the 5th structure

The correlation between 3DP similarity and RMSD was tested in a second case for a larger protein by considering ten domain movement frames for glutamine binding protein (1762 atoms) from the Protein Motion Database [40, 41]. These ten conformers represent different conformations of the binding domain spanning between open (PDB-entry 1GGG, purple structure in Fig. 3a) [42] and closed state (PDB-entry 1WDN, red structure in Fig. 3a) [43]. A good correlation was observed between 3DP similarity and RMSD (calculated with all-atom alignment by Maestro 8.5 [44]), in particular when considering conformer pairs from one extreme of the movement range (purple and red lines in Fig. 3b). Representation of the bit value changes showed that 3DP perceived the conformational change at the level of each of the four different atom type categories (Fig. 3c).

**Fig. 4:**
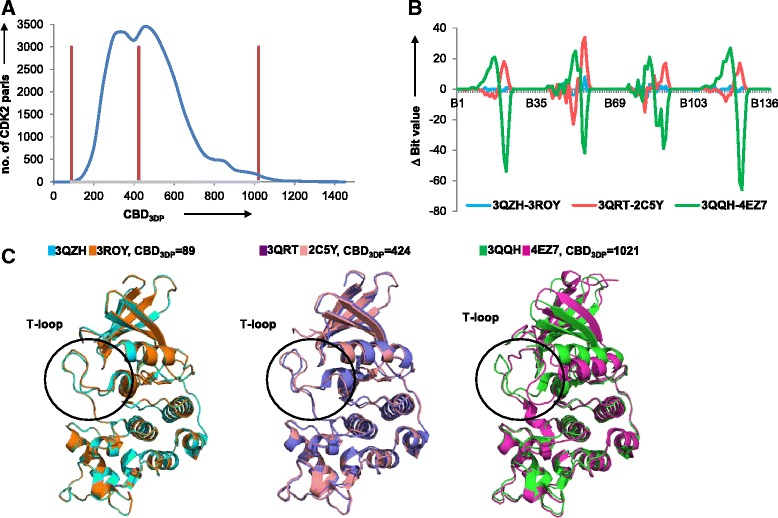
3DP distinguishes between closely related CDK2 T-loop conformers. **a** Frequency histogram of pairwise CBD_3DP_ values from 245 CDK2 conformations (*blue*). **b** Three pairs of CKD2s, 3QZH-3ROY, 3QRT-2C5Y, and 3QQH-4EZ7, were analyzed. They had CBD_3DP_ values 89 (*left red bar* in a), 424 (*middle red bar* in a) and 1021 (*right red bar* in a) respectively. Differences of bit values are shown for 3QZH-3ROY (*blue*), 3QRT-2C5Y (*red*), and 3QQH-4EZ7 (*green*). **c** Alignment of 3QZH (*blue*) and 3ROY (*orange*); 3QRT (*slate blue*) and 2C5Y (*coral*); 3QQH (*green*) and 4EZ7 (*magenta*)

The sensitivity of 3DP similarity to slight changes in protein conformation was evaluated in a third test case considering CDK2 (cyclin-dependent kinase 2), an important player in cell cycle regulation [45]. The activity of CDK2 is induced by conformational changes occurring upon ligand binding, in particular at the level of the large T-loop [46]. A total of 245 X-ray structures of CDK2 monomers bound with various small molecule inhibitors are available in the PDB (Additional file 1: Supplement 2). These CDK2 structures are nearly identical in sequence (>97 % identity), but show different conformation at the level of the T-loop due to an induced-fit adaptation of the protein around the various ligands. Although these 245 CDK2 structures were almost identical in terms of their global molecular shape, the 3DP analysis readily distinguished between the different T-loop conformers, with pairwise CBD_3DP_ distances between 75 and 1415 CBD_3DP_ units and maximum population around 300 ≤ CBD_3DP_ ≤ 500 (Fig. 4a). Three different pairs were analyzed closer in terms of differences in their 3DP values (Fig. 4b) and their overall structures superimposed by all-atom alignment (Fig. 4c) [47]. In terms of the 3DP values differences were visible in all four atom pair categories. In terms of the protein structures, the closest pair 3QZH/3ROY at CBD_3DP_ = 89 and the intermediate pair 3QRT/2C5Y at CBD_3DP_ = 424 only significantly differed at the level of the T-loop. For the 3QQH/4EZ7 pair at CBD_3DP_ = 1021, deviations were not only visible at the level of the T-loop but also in the α-helix and β-sheet at the top of the structures. The CDK2 analysis clearly showed that 3DP was able to perceive small conformational differences residing in a loop orientation between otherwise highly shape similar proteins.

### 3DP analysis of the RSCB protein databank

To facilitate access to PDB-entries a graphical user interface was created based on an interactive color-coded map representing the 3DP chemical space using the Mapplet principle previously reported for small molecule databases, by which database entries are displayed in a visualization window as the mouse cursor moves on the map, and connects to a fingerprint similarity search window to allow nearest neighbor searches [15–18]. Although 78 % of the data variability was represented in (PC1, PC2)-plane obtained by principal component analysis (PCA) of the 3DP dataset, this direct PCA map contained many scattered pixels with uneven occupancies and was not suitable as an interface (data not shown). We therefore generated an alternative representation of 3DP by similarity mapping, which produces more compact and evenly populated maps with a fairly good rendering for various chemical spaces [18, 48, 49].

**Fig. 5:**
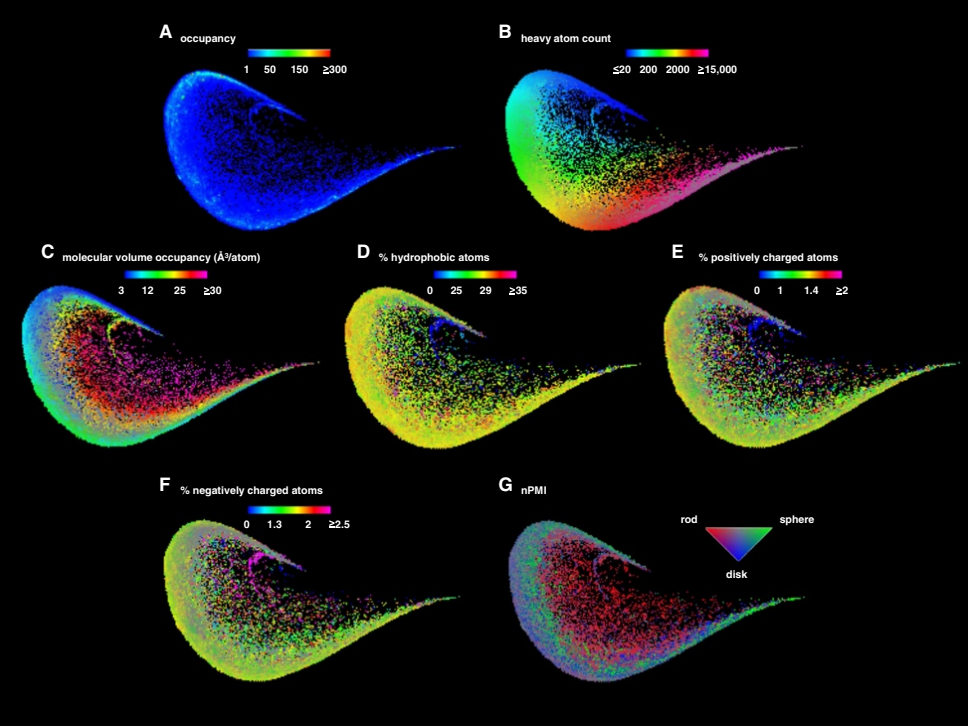
PDB-maps of 3DP-similarity space color-coded by (**a**) occupancy, (**b**) heavy atom count, (**c**) molecular volume occupancy (mvo), (**d**) percentage of hydrophobic atoms, (**e**) percentage of positively charged atoms, (**f**) percentage of negatively charged atoms. The color-coding is from *blue* (lowest values) to *magenta* (highest values). (**g**) PDB-map color-coded by nPMI values. The rod-like structures are *red* color; the spherical structures are *green* color; the disc-like structures are *blue* color. The maps were computed from 91,223 X-ray structures from the PDB downloaded in September 2014, considering in each case the biological assembly as defined by the author

To create the similarity map, 3DP similarity values relative to 200 randomly selected reference molecules from the PDB were calculated, and the (PC1, PC2)-plane obtained by PCA of the resulting 200-dimensional 3DP-similarity fingerprint was represented. This (PC1,PC2)-plane, called similarity map, covered 98 % of the similarity fingerprint data variability and had a compact, comet-like shape without peripheral pixels. Furthermore the map had a relatively even occupancy of pixels which was highly suitable as a representation of the PDB in 3DP space (Fig. 5a). PDB-entries were distributed on the map according to their size along the edge of the comet (Fig. 5b). The position on the map was influenced by the molecular volume occupancy (mvo, Å^3^/atom), a property defined here as the volume of the sphere with a radius corresponding to the average distance of all atoms to the center of gravity, divided by the total number of atoms, with the more compact, globular structure present at the comet edge and less compact structures at the center (Fig. 5c). The map also separated PDB-entries with different fractions of positively charged atoms, negatively charged atoms, and hydrophobic atoms, whereby the distribution pattern for the fraction of hydrophobic and positively charged atoms was somewhat similar (Fig. 5d–f). Color-coding by the normalized principal moments of inertia vector (nPMI1, nPMI2) distinguishing between rod-like, disc-like and sphere-like shapes, [21] showed that rod-like PDB-entries were located at the comet center, while the more spherical entries were distributed around the rest of the map (Fig. 5g).

**Fig. 6:**
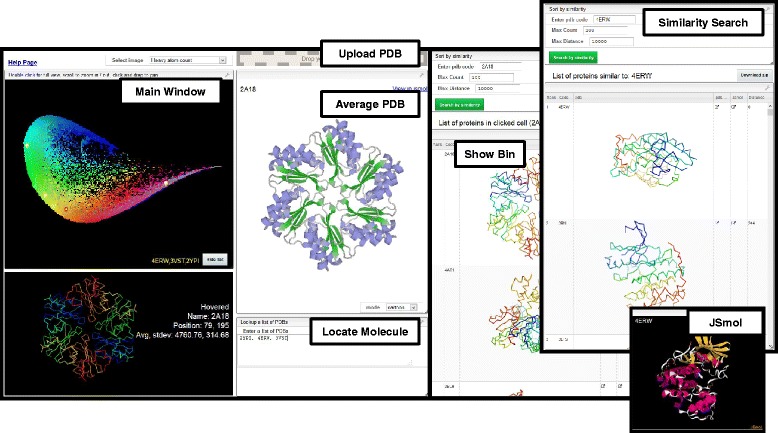
Interface of PDB-Explorer website. *Main window*: color-coded similarity map of PDB in 3DP-similarity space, with image of the protein in the pixel marked by the mouse cursor on the map. *Upload PDB*: place to load a user-defined PDB-file to be shown on the 3DP-similarity map (structure must contain properly annotated atoms). *Average PDB*: interactive 3D-view of the molecule corresponding to the most average entry in selected pixel. *Locate Molecule*: interface to type PDB entry codes to be shown on the map. *Show Bin*: full list of all proteins contain in the selected pixel. *Similarity Search*: window to enter PDB-code and search for nearest neighbours in 3DP-space, and display of the nearest neighbour list. *JSmol*: 3D-display of selected entry

The color-coded similarity maps were combined with the 3DP-similarity search tool into a web-based application called “PDB-Explorer” for interactive visualization and 3DP-similarity search through the entire PDB (Fig. 6). The website uses the same principles as our previously reported MQN-mapplet, [15–17] and is based on the open-source project visualizer (http://github.com/NPellet/visualizer) that was already successfully used for another cheminformatic project [50]. The PDB-Explorer consists of a main window to browse through various color-coded rendering of the 3DP-similarity map. The average PDB molecule in each pixel is shown in the 3D-viewer of PV, [51] and the “Show Bin” table details the contents of any selected pixel on the map from which each PDB-entry, shown as ribbon image, can be inspected closer as 3D-model by opening a secondary JSmol window. The “Locate Molecule” function allows locating any given PDB-entries on the map and the “Similarity Search” option offers nearest neighbors of any PDB-entry in the 3DP space within seconds. The application also contains uploading function to locate and search nearest neighbors of any structure represented as pdb file. All computations are performed locally by the browser of the user machine. The color-coded similarity maps and the database for similarity search are updated every day by adding new entries in the PDB. The detailed PDB-Explorer functions are described in the HELP page.

### Exploring PDB using the PDB-Explorer

The PDB-Explorer allows the rapid analysis and overview of the entire PDB as well as detailed searches around selected PDB-entries using 3DP similarity as a guiding principle. Its use is exemplified with three case studies detailed below which further demonstrate the remarkable ability of 3DP to classify proteins according to their 3D structure.

**Fig. 7:**
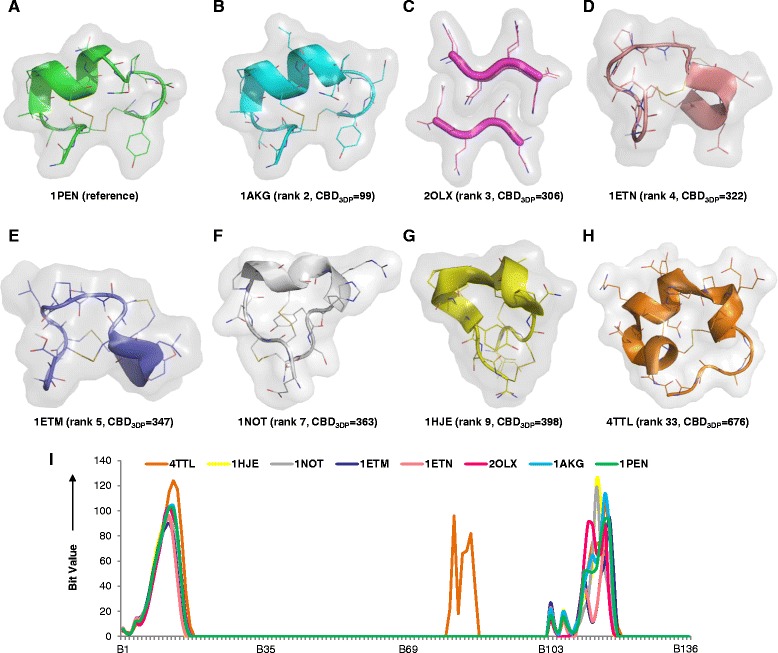
Analogs of α-conotoxin 1PEN retrieved by 3DP similarity. **a** Reference molecule, 1PEN. **b**-**h** Structures of selected nearest neighbors and CBD_3DP_ to the reference. **i** Bit value profiles of conotoxins and analogs

The case of the conotoxins, a group of 10 to 30 residues neurotoxic peptides containing multiple intramolecular disulfide bridges and a variety of secondary structures, [52–54] provides an example of the smallest molecules listed in the PDB. Alpha-conotoxin PnIA (PDB-entry 1PEN) with 17 residues serves as reference molecule (Fig. 7a). The closest 3DP analog of 1PEN retrieved by 3DP similarity is 1AKG, which belongs to the similar α-conotoxin family and is also retrieved as nearest neighbor by BLAST search with 83 % sequence similarity (Fig. 7b) [55]. By contrast the second analog found by 3DP similarity is a short fibril peptide complex (Fig. 7c), which has a completely different secondary structure and sequence, but a similar overall size and shape. The 3rd (Fig. 7d) and 4th analog (Fig. 7e) are both from the enterotoxin family featuring a conotoxin-like shape containing double disulfide bridges, again without significant sequence similarity to the reference. Three further conotoxins missed by sequence similarity appear in the 3DP nearest neighbor search at rank 7 (1NOT, Fig. 7f), rank 9 (1HJE, Fig. 7g) and rank 33 (4TTL, Fig. 7h). In the latter case the bit value profile shows that 4TTL is the only sequence containing negatively charged residues, while all other cases do not contain any charged residues (Fig. 7i).

**Fig. 8:**
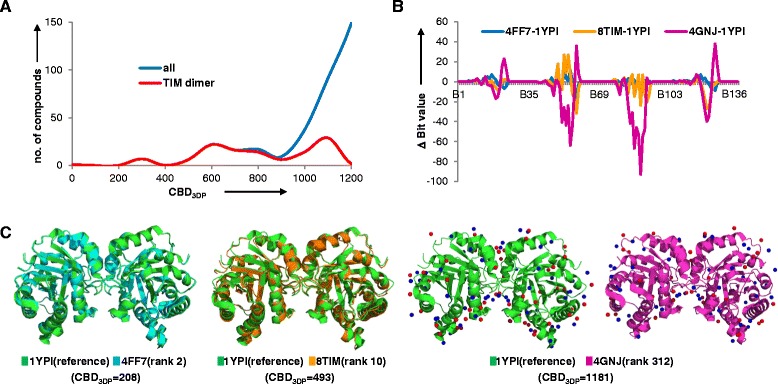
Similarity search result for triose phosphate isomerase (TIM) dimer, 1YPI. **a** Frequency histogram of CBD_3DP_ between all of the structures in PDB and 1YPI (*blue*), and all of the TIM dimers and 1YPI (*red*). **b** Deviation of bit values of 4FF7, 8TIM and 4GNJ to the reference 1YPI. **c** Comparison of the structures of reference 1YPI and rank2, rank10 and rank 321 analogs retrieved from similarity search. The positively charged atoms are shown as *blue spheres* and negatively charged atoms are shown as *red spheres* for 1YPI and 4GNJ

3DP similarity also retrieves shape analogs of molecular assemblies, as exemplified here with triose phosphate isomerase (TIM), a homodimeric enzyme which contains around 250 amino acids in the monomer [56]. The wild type TIM dimer from yeast (PDB-entry 1YPI) is selected as the reference. The top 100 neighbors of 1YPI are also protein dimers, with essentially all neighbors of 1YPI up to CBD_3DP_ = 800 being TIM enzymes. All TIM structures in PDB (Additional file 1: Supplement 3) in fact occur within a CBD_3DP_ distance of 1200 except 13 TIM enzymes ranked beyond rank 300 (Fig. 8a). The differences in 3DP values are illustrated for the nearest neighbor 4FF7, the rank 10 analog 8TIM at distance CBD_3DP_ = 493 representative of the bulk of TIM structures, and 4GNJ found at rank 312 and CBD_3DP_ = 1181 representative of the more distant group around CBD_3DP_ = 1100 (Fig. 8b). The nearest neighbor of 1YPI, 4FF7, is also a yeast TIM with 99 % sequence similarity and nearly identical structure. PDB-entry 8TIM at rank 10 is a TIM dimer from a different organism (gallus gallus) with only 52 % of sequence similarity. In both cases the shape and fold differences to the reference are quite small, and the larger distance of 8TIM stems from differences in positively charged atoms that are not directly visible in the protein shape (Fig. 8b/c). The difference in the number and position of charged atoms also explains the further distance from the reference of 4GNJ, a TIM dimer from leishmania siamensis.

**Fig. 9:**
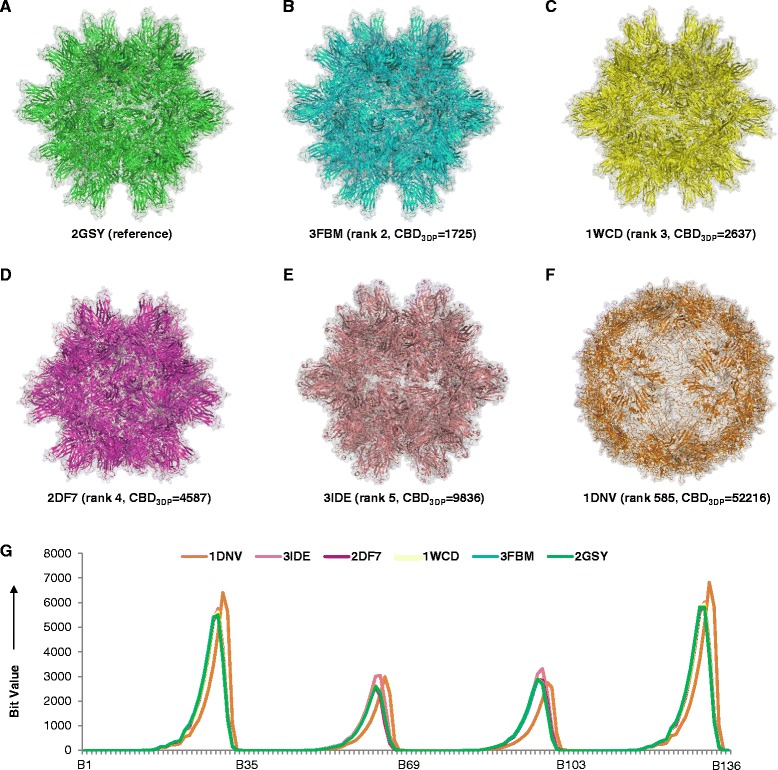
Similarity search result for virus capsid, 2GSY. **a**-**e** The structures of reference and top 4 analogs retrieved from similarity search. **f** Parvovirus capsid protein, 1DNV. **g** Bit values of compounds a-f

The PDB-Explorer furthermore provides interesting insight at the level of very large protein assemblies, as exemplified here for the case of a virus capsid. Starting with the subviral particle of the bursal disease virus capsid (PDB-entry 2GSY) [57] containing 27,120 residues (Fig. 9a), four analogs are readily identified within the distance range CBD_3DP_ < 10,000. The nearest neighbor is 3FBM (Fig. 9b) which is a mutant protein of the query 2GSY, [58] and the second and third closest structures 1WCD and 2DF7 are capsids from the same bursal disease virus (Fig. 9c, d). The fourth analog (PDB-entry 3IDE) is the protein coat of Infectious Pancreatic Necrosis Virus (IPNV) [59] which has similar spikes and forms a similar icosahedral capsid organization as the capsid of infectious bursal disease virus (Fig. 9e) [59]. Interestingly the parvovirus capsid protein 1DNV (Fig. 9f), which is very close in size to the reference 2GSY, only appears much further away in CBD_3DP_ space because its spherical shape does not feature spikes, which significantly impact its 3DP fingerprint profile (Fig. 9g).

**Fig. 10:**
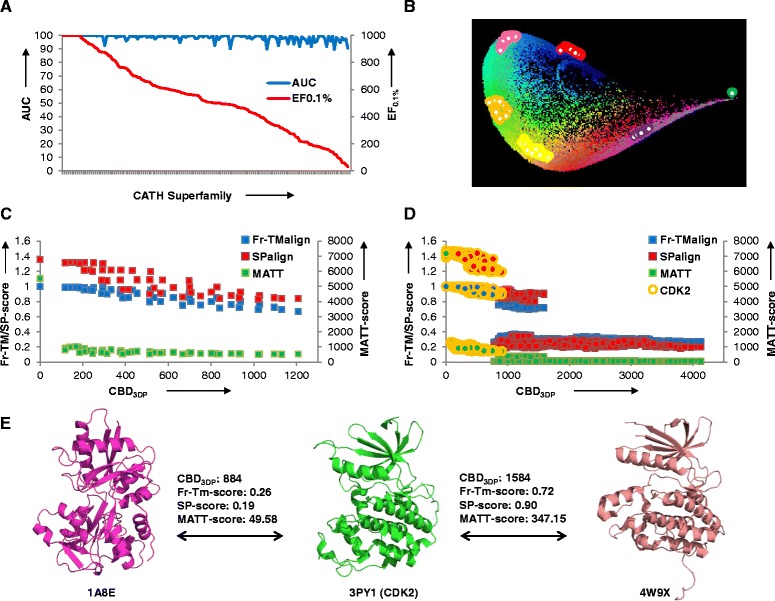
Classification of CATH superfamilies in 3DP-space and comparison of 3DP with structural alignment tools Fr-TMalign, SPalign and MATT. **a** AUC and EF_0.1%_ values for ROC curves recovering 150 CATH superfamilies from the entire PDB by 3DP-similarity. **b** Locations of 6 CATH superfamilies (1.10.246.10, 2.30.30.40, 2.60.120.20, 3.10.320.10, 3.40.50.200 and 3.40.309.10) on the PDB-map. **c** Correlation between alignment scores (Fr-TMalign, SPalign, MATT) and CBD_3DP_ obtained from 10 domain movement frames of the glutamine binding protein (Fig. 3). *Red square*: Fr-TMalign; *Blue square*: SPalign; *Green square*: MATT. **d** Correlation between alignment scores (Fr-TMalign, SPalign, MATT) and CBD_3DP_ for 50 CDK2 and 50 decoys (1225 CDK2 pairs and 2500 CDK2-decoy cross-pairs). *Red square*: Fr-TMalign; *Blue square*: SPalign; *Green square*: MATT; *Orange circle*: CDK2 proteins. **e** Example of shape analogs of the CDK2 protein 3PY1 identified by 3DP-similarity or 3D-alignment tools. 1A8E is detected as shape analog of 3PY1 by 3DP similarity, but not by any of the three alignment tools. 4W9X is similar to 3PY1 in each of the three alignment tools, but is not a close analog by 3DP similarity

3DP space automatically clusters proteins from the same superfamily in tight groups provided that they are in a similar size range. This property is illustrated here for the recovery of each family from a benchmark dataset of 150 CATH superfamilies, each containing between 12 and 1533 PDB entries [7, 8], considering proteins in the majority size range (Additional file 1: Supplement 4). The ROC (receiver operator characteristic) curves for recovering superfamily members from the PDB-entry closest to all same superfamily members in 3DP-space give very high AUC (area under the curve, all above 90 %) and generally high EF_0.1%_ values (enrichment factor at 0.1 % database coverage in the range 29–1000, Fig. 10a). Most superfamilies also appear as tight groups on the PDB-map (Fig. 10b). The clustering of protein superfamilies in 3DP-space reflects the fact that the definition of these families considers similarities in folds in addition to function.

### Comparing 3DP with protein structure alignment tools

The performance of 3DP was compared with three protein structure alignment tools, Fr-TMalign [60, 61], SPalign [62] and MATT [63]. Fr-TMalign is applied to pairwise structure alignment based on fragment similarity, while SPalign is designed for detecting proteins with similar fold and similar function of DNA or RNA binding. MATT (Multiple Alignment with Translations and Twists) is a program to align multiple protein structures allowing certain flexibility between fragments. These alignment methods are computationally intensive and therefore only applicable to a limited number of comparisons. They are size independent and focus on backbone alignment resulting in a focus on secondary structures. On the other hand 3DP is size dependent and considers all protein atoms indiscriminately, resulting in a sensitivity to the overall shape rather than to secondary structures. Remarkably, 3DP allows an essentially instantaneous comparison with the entire PDB when using the PDB-Explorer website.

In a first comparative study, all pairwise alignment scores were computed for the ten domain movement frames for glutamine binding protein and compared with CBD_3DP_. The data showed that CBD_3DP_, which performs comparably to overall structure RMSD as dicussed above (Fig. 3), had similar trend but higher sensitivity to conformer differences than Fr-TMalign or SPalign. MATT was highly sensitive to small conformational changes and classified all non-identical conformer pairs as low scoring (Fig. 10c).

A further comparison of 3DP with structure alignment tools was carried out for 50 homologous CDK2 proteins (Fig. 4) and 50 non-CDK2 decoy proteins. The 100 structures were in similar size range from 2100–2600 heavy atoms, and decoys consisted of non-homologous proteins with pairwise sequence identity lower than 30 %. Alignment scores and 3DP distances were computed for the 1225 CDK2 pairs and the 2500 CDK2-decoy pairs (Fig. 10d). 3DP made a relatively clear cut between CDK2 pairs and CDK2-decoy cross-pairs at CBD_3DP_ = 750, with all CDK2 pairs found within the range CBD_3DP_ < 1000. However the 3DP comparison recognized some decoys such as 1A8E (serum transferrin) as CDK2-like due to an overall similar shape, although this decoy had a clearly different fold as correctly analyzed by each of the three alignment tools (Fig. 10e, left). The three alignment methods correctly assigned a high score to all CDK2 pairs, but also returned a high core with part of the CDK2-decoys which were not recognized as CDK2 like by 3DP. For example decoy 4W9X, which is a non-CDK2 kinase, clearly showed a partly homologous fold to CDK2 leading to a high alignment score, but also showed substantial differences with the presence of a central helix and an extended terminal absent from CDK2 which resulted in a relatively high 3DP-distance to CDK2s such as 3PY1 (Fig. 10e, right).

## Conclusion

The 3D-structure of biomolecules in the PDB were encoded in the 136-dimensional 3D atom-pair fingerprint 3DP counting the number of atom pairs at increasing distance intervals for all atoms, positively charged, negatively charged, and hydrophobic atoms. The 3DP fingerprint perceives the spatial distribution of shape, hydrophobicity and charges in molecular objects across a very broad size range. 3DP nearest neighbors are shown in various examples to be closely related shape and fold analogs.

The 3DP property space is represented in form of an interactive color-coded similarity map distributing PDB-entries by molecular size and shape, and connected to a similarity search function which identifies nearest neighbors of any PDB-entry in the 136-dimensional 3DP-space. These tools are combined in the PDB-Explorer website running on JavaScript in a platform-independent manner and drawing data from a server that is updated daily with the latest PDB additions, ensuring a complete and most up-to-date coverage. The PDB`-Explorer website is publicly accessible at www.cheminfo.org/pdbexplorer and represents an unprecedented opportunity to interactively visualize and explore the structural diversity of the PDB.

## Availability of supporting data

The data sets supporting the results of this article are included within the article and its additional file.

## Additional file

Additional file 1:
**Supplement 1.** PDB IDs of 91,223 X-ray structures downloaded from the PDB in September 2014. **Supplement 2.** PDB IDs of X-ray structures of cyclin-dependent kinase 2 (CDK2). **Supplement 3.** PDB IDs of X-ray structures of triose phosphate isomerase (TIM) dimer. **Supplement 4.** IDs of CATH superfamilies and PDB IDs in each CATH superfamily. (DOCX 297 kb)
