# Supplementary material for: PDB-Explorer: a web-based interactive map of the protein data bank in shape space
Source: BMC Bioinformatics. 2015 Oct 23;16:339. doi: 10.1186/s12859-015-0776-9 (PMC4619230; doi:10.1186/s12859-015-0776-9)
Supplement: Additional file 1: — Supplement 1. PDB IDs of 91,223 X-ray structures downloaded from the PDB in September 2014. Supplement 2. PDB IDs of X-ray structures of cyclin-dependent kinase 2 (CDK2). Supplement 3. PDB IDs of X-ray structures of triose phosphate isomerase (TIM) dimer. Supplement 4. IDs of CATH superfamilies and PDB IDs in each CATH superfamily. (DOCX 297 kb) [file 12859_2015_776_MOESM1_ESM.docx]

**Supplement 1: PDB IDs of 91,223 X-ray structures downloaded from the PDB in September 2014**

1C51, 1C53, 1FE1, 1ILX, 1QZV, 2QQP, 101D, 101M, 102D, 102L, 102M, 103L, 103M, 104L, 104M, 105M, 106M, 107L, 107M, 108L, 108M, 109D, 109L, 109M, 10GS, 10MH, 110L, 110M, 111D, 111L, 111M, 112D, 112L, 112M, 113D, 113L, 114D, 114L, 115L, 116D, 117D, 117E, 118D, 118L, 119D, 119L, 11AS, 11BA, 11BG, 11GS, 120L, 121D, 121P, 122D, 122L, 123D, 123L, 125L, 126D, 126L, 127D, 127L, 128D, 128L, 129D, 129L, 12AS, 12CA, 12E8, 12GS, 130D, 130L, 131D, 131L, 132L, 133D, 133L, 134L, 135L, 137D, 137L, 138D, 138L, 139L, 13GS, 13PK, 140L, 141L, 142L, 143L, 144D, 144L, 145L, 146L, 147L, 148L, 149L, 14GS, 150D, 150L, 151L, 152L, 153D, 153L, 154L, 155C, 155L, 156L, 157L, 158D, 158L, 159D, 159L, 15C8, 160D, 160L, 161D, 161L, 162L, 163L, 164L, 165L, 166D, 166L, 167D, 167L, 168D, 168L, 169L, 16GS, 16PK, 16VP, 170L, 171L, 172D, 172L, 173D, 173L, 174L, 175L, 176L, 177L, 178D, 178L, 17GS, 180D, 180L, 181D, 181L, 182L, 183D, 183L, 184D, 184L, 185L, 186L, 187D, 187L, 188D, 188L, 189D, 189L, 18GS, 190D, 190L, 1914, 191D, 191L, 192D, 192L, 193L, 194D, 194L, 195D, 195L, 196D, 196L, 197D, 197L, 198L, 199L, 19GS, 19HC, 1A00, 1A01, 1A02, 1A04, 1A05, 1A06, 1A07, 1A08, 1A09, 1A0A, 1A0B, 1A0C, 1A0D, 1A0E, 1A0F, 1A0G, 1A0H, 1A0I, 1A0J, 1A0K, 1A0L, 1A0M, 1A0O, 1A0P, 1A0Q, 1A0R, 1A0S, 1A0T, 1A0U, 1A0Z, 1A12, 1A14, 1A15, 1A16, 1A17, 1A18, 1A19, 1A1A, 1A1B, 1A1C, 1A1E, 1A1F, 1A1G, 1A1H, 1A1I, 1A1J, 1A1K, 1A1L, 1A1M, 1A1N, 1A1O, 1A1Q, 1A1R, 1A1S, 1A1V, 1A1X, 1A21, 1A22, 1A25, 1A26, 1A27, 1A28, 1A29, 1A2A, 1A2B, 1A2C, 1A2D, 1A2E, 1A2F, 1A2G, 1A2J, 1A2K, 1A2L, 1A2M, 1A2N, 1A2O, 1A2P, 1A2Q, 1A2T, 1A2U, 1A2V, 1A2W, 1A2X, 1A2Y, 1A2Z, 1A30, 1A31, 1A32, 1A33, 1A35, 1A36, 1A37, 1A38, 1A39, 1A3A, 1A3B, 1A3C, 1A3D, 1A3E, 1A3F, 1A3G, 1A3H, 1A3I, 1A3J, 1A3K, 1A3L, 1A3N, 1A3O, 1A3Q, 1A3R, 1A3S, 1A3T, 1A3U, 1A3V, 1A3W, 1A3X, 1A3Y, 1A3Z, 1A40, 1A41, 1A42, 1A43, 1A44, 1A45, 1A46, 1A47, 1A48, 1A49, 1A4A, 1A4B, 1A4C, 1A4E, 1A4F, 1A4G, 1A4H, 1A4I, 1A4J, 1A4K, 1A4L, 1A4M, 1A4O, 1A4P, 1A4Q, 1A4R, 1A4S, 1A4U, 1A4V, 1A4W, 1A4X, 1A4Y, 1A4Z, 1A50, 1A52, 1A53, 1A54, 1A55, 1A58, 1A59, 1A5A, 1A5B, 1A5C, 1A5D, 1A5F, 1A5G, 1A5H, 1A5I, 1A5K, 1A5L, 1A5M, 1A5N, 1A5O, 1A5P, 1A5Q, 1A5S, 1A5T, 1A5U, 1A5V, 1A5W, 1A5X, 1A5Y, 1A5Z, 1A61, 1A62, 1A64, 1A65, 1A68, 1A69, 1A6A, 1A6D, 1A6E, 1A6F, 1A6G, 1A6I, 1A6J, 1A6K, 1A6L, 1A6M, 1A6N, 1A6P, 1A6Q, 1A6R, 1A6T, 1A6U, 1A6V, 1A6W, 1A6Y, 1A6Z, 1A70, 1A71, 1A72, 1A73, 1A74, 1A75, 1A76, 1A77, 1A78, 1A79, 1A7A, 1A7B, 1A7C, 1A7D, 1A7E, 1A7G, 1A7H, 1A7J, 1A7K, 1A7L, 1A7N, 1A7O, 1A7P, 1A7Q, 1A7R, 1A7S, 1A7T, 1A7U, 1A7V, 1A7W, 1A7X, 1A80, 1A81, 1A82, 1A85, 1A86, 1A87, 1A88, 1A8A, 1A8B, 1A8D, 1A8E, 1A8F, 1A8G, 1A8H, 1A8I, 1A8J, 1A8K, 1A8L, 1A8M, 1A8O, 1A8P, 1A8Q, 1A8R, 1A8S, 1A8T, 1A8U, 1A8V, 1A8Y, 1A8Z, 1A92, 1A94, 1A95, 1A96, 1A97, 1A98, 1A99, 1A9B, 1A9C, 1A9E, 1A9M, 1A9O, 1A9P, 1A9Q, 1A9R, 1A9S, 1A9T, 1A9U, 1A9W, 1A9X, 1A9Y, 1A9Z, 1AA0, 1AA1, 1AA2, 1AA4, 1AA6, 1AA7, 1AAC, 1AAJ, 1AAL, 1AAM, 1AAN, 1AAP, 1AAQ, 1AAR, 1AAT, 1AAW, 1AAX, 1AAY, 1AAZ, 1AB0, 1AB1, 1AB4, 1AB5, 1AB6, 1AB8, 1AB9, 1ABA, 1ABB, 1ABE, 1ABF, 1ABI, 1ABJ, 1ABN, 1ABO, 1ABQ, 1ABR, 1ABS, 1ABW, 1ABY, 1AC1, 1AC4, 1AC5, 1AC6, 1AC8, 1ACB, 1ACC, 1ACD, 1ACF, 1ACJ, 1ACL, 1ACM, 1ACO, 1ACV, 1ACX, 1ACY, 1AD0, 1AD1, 1AD2, 1AD3, 1AD4, 1AD5, 1AD6, 1AD8, 1AD9, 1ADB, 1ADC, 1ADD, 1ADE, 1ADF, 1ADG, 1ADI, 1ADJ, 1ADL, 1ADO, 1ADQ, 1ADS, 1ADU, 1ADV, 1ADW, 1ADY, 1AE1, 1AE2, 1AE3, 1AE4, 1AE5, 1AE6, 1AE7, 1AE8, 1AE9, 1AEB, 1AEC, 1AED, 1AEE, 1AEF, 1AEG, 1AEH, 1AEI, 1AEJ, 1AEK, 1AEM, 1AEN, 1AEO, 1AEP, 1AEQ, 1AER, 1AES, 1AET, 1AEU, 1AEV, 1AEW, 1AEX, 1AF0, 1AF2, 1AF3, 1AF4, 1AF5, 1AF6, 1AF7, 1AF9, 1AFA, 1AFB, 1AFC, 1AFD, 1AFE, 1AFK, 1AFL, 1AFQ, 1AFR, 1AFS, 1AFU, 1AFV, 1AFW, 1AG0, 1AG1, 1AG6, 1AG8, 1AG9, 1AGB, 1AGC, 1AGD, 1AGE, 1AGF, 1AGI, 1AGJ, 1AGM, 1AGN, 1AGP, 1AGQ, 1AGR, 1AGS, 1AGW, 1AGX, 1AGY, 1AH0, 1AH3, 1AH4, 1AH5, 1AH6, 1AH7, 1AH8, 1AHA, 1AHB, 1AHC, 1AHE, 1AHF, 1AHG, 1AHH, 1AHI, 1AHJ, 1AHN, 1AHO, 1AHP, 1AHQ, 1AHR, 1AHS, 1AHT, 1AHU, 1AHV, 1AHW, 1AHX, 1AHY, 1AHZ, 1AI1, 1AI2, 1AI3, 1AI4, 1AI5, 1AI6, 1AI7, 1AI8, 1AI9, 1AIA, 1AIB, 1AIC, 1AID, 1AIE, 1AIF, 1AIG, 1AIH, 1AII, 1AIJ, 1AIK, 1AIL, 1AIM, 1AIN, 1AIO, 1AIP, 1AIQ, 1AIR, 1AIS, 1AIU, 1AIV, 1AIX, 1AIZ, 1AJ0, 1AJ2, 1AJ5, 1AJ6, 1AJ7, 1AJ8, 1AJ9, 1AJA, 1AJB, 1AJC, 1AJD, 1AJG, 1AJH, 1AJJ, 1AJK, 1AJM, 1AJN, 1AJO, 1AJP, 1AJQ, 1AJR, 1AJS, 1AJV, 1AJX, 1AJZ, 1AK0, 1AK1, 1AK2, 1AK4, 1AK5, 1AK9, 1AKA, 1AKB, 1AKC, 1AKD, 1AKE, 1AKG, 1AKH, 1AKI, 1AKJ, 1AKL, 1AKM, 1AKN, 1AKO, 1AKQ, 1AKR, 1AKS, 1AKT, 1AKU, 1AKV, 1AKW, 1AKY, 1AKZ, 1AL1, 1AL3, 1AL6, 1AL7, 1AL8, 1ALA, 1ALB, 1ALC, 1ALD, 1ALH, 1ALI, 1ALJ, 1ALK, 1ALL, 1ALN, 1ALQ, 1ALU, 1ALV, 1ALW, 1ALY, 1AM1, 1AM2, 1AM4, 1AM5, 1AM6, 1AM7, 1AM9, 1AMA, 1AME, 1AMF, 1AMH, 1AMI, 1AMJ, 1AMK, 1AMM, 1AMN, 1AMO, 1AMP, 1AMQ, 1AMR, 1AMS, 1AMU, 1AMW, 1AMX, 1AMY, 1AMZ, 1AN0, 1AN1, 1AN2, 1AN4, 1AN5, 1AN7, 1AN8, 1AN9, 1ANB, 1ANC, 1AND, 1ANE, 1ANF, 1ANG, 1ANI, 1ANJ, 1ANK, 1ANN, 1ANT, 1ANU, 1ANV, 1ANW, 1ANX, 1AO0, 1AO3, 1AO5, 1AO6, 1AO7, 1AOA, 1AOB, 1AOC, 1AOD, 1AOE, 1AOF, 1AOG, 1AOH, 1AOI, 1AOJ, 1AOK, 1AOL, 1AOM, 1AON, 1AOP, 1AOQ, 1AOR, 1AOS, 1AOV, 1AOW, 1AOX, 1AOZ, 1AP2, 1AP5, 1AP6, 1AP9, 1APA, 1APB, 1APH, 1APL, 1APM, 1APN, 1APT, 1APU, 1APV, 1APW, 1APX, 1APY, 1APZ, 1AQ0, 1AQ1, 1AQ2, 1AQ6, 1AQ7, 1AQ8, 1AQB, 1AQC, 1AQD, 1AQE, 1AQF, 1AQH, 1AQI, 1AQJ, 1AQK, 1AQL, 1AQM, 1AQN, 1AQP, 1AQT, 1AQU, 1AQV, 1AQW, 1AQX, 1AQY, 1AQZ, 1AR0, 1AR1, 1AR2, 1AR4, 1AR5, 1ARB, 1ARC, 1ARG, 1ARH, 1ARI, 1ARL, 1ARM, 1ARO, 1ARP, 1ARS, 1ART, 1ARU, 1ARV, 1ARW, 1ARX, 1ARY, 1ARZ, 1AS0, 1AS2, 1AS3, 1AS4, 1AS6, 1AS7, 1AS8, 1ASA, 1ASB, 1ASC, 1ASD, 1ASE, 1ASF, 1ASG, 1ASH, 1ASK, 1ASL, 1ASM, 1ASN, 1ASO, 1ASP, 1ASQ, 1ASS, 1AST, 1ASU, 1ASV, 1ASW, 1ASX, 1AT0, 1AT1, 1AT3, 1AT5, 1AT6, 1ATG, 1ATH, 1ATI, 1ATJ, 1ATK, 1ATL, 1ATN, 1ATP, 1ATR, 1ATS, 1ATT, 1ATU, 1ATZ, 1AU0, 1AU1, 1AU2, 1AU3, 1AU4, 1AU7, 1AU8, 1AU9, 1AUA, 1AUC, 1AUE, 1AUG, 1AUI, 1AUJ, 1AUK, 1AUM, 1AUN, 1AUO, 1AUP, 1AUQ, 1AUR, 1AUS, 1AUT, 1AUV, 1AUW, 1AUX, 1AV1, 1AV4, 1AV5, 1AV7, 1AV8, 1AVA, 1AVB, 1AVC, 1AVD, 1AVE, 1AVF, 1AVG, 1AVH, 1AVK, 1AVL, 1AVM, 1AVN, 1AVO, 1AVP, 1AVQ, 1AVR, 1AVS, 1AVT, 1AVU, 1AVV, 1AVW, 1AVX, 1AVY, 1AVZ, 1AW1, 1AW2, 1AW5, 1AW7, 1AW8, 1AW9, 1AWB, 1AWC, 1AWD, 1AWF, 1AWH, 1AWI, 1AWP, 1AWQ, 1AWR, 1AWS, 1AWT, 1AWU, 1AWV, 1AX0, 1AX1, 1AX2, 1AX4, 1AX8, 1AX9, 1AXA, 1AXB, 1AXC, 1AXD, 1AXE, 1AXG, 1AXI, 1AXK, 1AXM, 1AXN, 1AXQ, 1AXR, 1AXS, 1AXT, 1AXW, 1AXY, 1AXZ, 1AY0, 1AY1, 1AY2, 1AY4, 1AY5, 1AY6, 1AY7, 1AY8, 1AY9, 1AYA, 1AYB, 1AYC, 1AYD, 1AYE, 1AYF, 1AYI, 1AYL, 1AYO, 1AYP, 1AYR, 1AYU, 1AYV, 1AYW, 1AYX, 1AYY, 1AYZ, 1AZ0, 1AZ1, 1AZ2, 1AZ3, 1AZ4, 1AZ5, 1AZ8, 1AZB, 1AZC, 1AZD, 1AZF, 1AZI, 1AZL, 1AZM, 1AZN, 1AZO, 1AZP, 1AZQ, 1AZR, 1AZS, 1AZT, 1AZU, 1AZV, 1AZW, 1AZX, 1AZY, 1AZZ, 1B00, 1B01, 1B02, 1B04, 1B05, 1B06, 1B07, 1B08, 1B09, 1B0A, 1B0B, 1B0C, 1B0D, 1B0E, 1B0F, 1B0G, 1B0H, 1B0I, 1B0J, 1B0K, 1B0L, 1B0M, 1B0N, 1B0O, 1B0P, 1B0R, 1B0T, 1B0U, 1B0V, 1B0W, 1B0X, 1B0Y, 1B0Z, 1B11, 1B12, 1B13, 1B14, 1B15, 1B16, 1B17, 1B18, 1B19, 1B1B, 1B1C, 1B1E, 1B1H, 1B1I, 1B1J, 1B1U, 1B1X, 1B1Y, 1B1Z, 1B20, 1B21, 1B24, 1B25, 1B26, 1B27, 1B2A, 1B2B, 1B2C, 1B2D, 1B2E, 1B2F, 1B2G, 1B2H, 1B2J, 1B2K, 1B2L, 1B2M, 1B2O, 1B2P, 1B2R, 1B2S, 1B2U, 1B2V, 1B2W, 1B2X, 1B2Y, 1B2Z, 1B30, 1B31, 1B32, 1B33, 1B34, 1B37, 1B38, 1B39, 1B3A, 1B3B, 1B3D, 1B3E, 1B3F, 1B3G, 1B3H, 1B3J, 1B3K, 1B3L, 1B3N, 1B3O, 1B3Q, 1B3R, 1B3S, 1B3T, 1B3U, 1B3V, 1B3W, 1B3X, 1B3Y, 1B3Z, 1B40, 1B41, 1B42, 1B43, 1B44, 1B46, 1B47, 1B48, 1B49, 1B4A, 1B4B, 1B4D, 1B4E, 1B4F, 1B4H, 1B4J, 1B4K, 1B4L, 1B4N, 1B4P, 1B4S, 1B4T, 1B4U, 1B4V, 1B4W, 1B4X, 1B4Z, 1B51, 1B52, 1B54, 1B55, 1B56, 1B57, 1B58, 1B59, 1B5D, 1B5E, 1B5F, 1B5G, 1B5H, 1B5I, 1B5J, 1B5L, 1B5M, 1B5O, 1B5P, 1B5Q, 1B5S, 1B5T, 1B5U, 1B5V, 1B5W, 1B5X, 1B5Y, 1B5Z, 1B62, 1B63, 1B65, 1B66, 1B67, 1B68, 1B6A, 1B6B, 1B6C, 1B6D, 1B6E, 1B6G, 1B6H, 1B6I, 1B6J, 1B6K, 1B6L, 1B6M, 1B6P, 1B6Q, 1B6R, 1B6S, 1B6T, 1B6U, 1B6V, 1B6W, 1B6Z, 1B70, 1B71, 1B72, 1B73, 1B74, 1B76, 1B77, 1B78, 1B79, 1B7A, 1B7B, 1B7D, 1B7E, 1B7G, 1B7H, 1B7I, 1B7J, 1B7K, 1B7L, 1B7M, 1B7N, 1B7O, 1B7P, 1B7Q, 1B7R, 1B7S, 1B7T, 1B7U, 1B7V, 1B7X, 1B7Y, 1B7Z, 1B80, 1B82, 1B85, 1B86, 1B87, 1B88, 1B89, 1B8A, 1B8C, 1B8D, 1B8E, 1B8F, 1B8G, 1B8H, 1B8I, 1B8J, 1B8K, 1B8L, 1B8M, 1B8N, 1B8O, 1B8P, 1B8R, 1B8S, 1B8U, 1B8V, 1B8X, 1B8Y, 1B8Z, 1B90, 1B92, 1B93, 1B94, 1B95, 1B96, 1B97, 1B98, 1B99, 1B9A, 1B9B, 1B9C, 1B9D, 1B9E, 1B9F, 1B9H, 1B9I, 1B9J, 1B9K, 1B9L, 1B9M, 1B9N, 1B9O, 1B9S, 1B9T, 1B9V, 1B9W, 1B9X, 1B9Y, 1B9Z, 1BA0, 1BA1, 1BA2, 1BA3, 1BA7, 1BA8, 1BAB, 1BAF, 1BAG, 1BAI, 1BAJ, 1BAM, 1BAN, 1BAO, 1BAP, 1BAR, 1BAS, 1BAV, 1BAW, 1BAY, 1BAZ, 1BB0, 1BB1, 1BB3, 1BB4, 1BB5, 1BB6, 1BB7, 1BB9, 1BBB, 1BBC, 1BBD, 1BBH, 1BBJ, 1BBP, 1BBR, 1BBS, 1BBU, 1BBW, 1BBZ, 1BC0, 1BC1, 1BC2, 1BC3, 1BC5, 1BC7, 1BC8, 1BCC, 1BCD, 1BCF, 1BCG, 1BCH, 1BCJ, 1BCK, 1BCM, 1BCO, 1BCP, 1BCS, 1BCU, 1BCW, 1BCX, 1BCY, 1BCZ, 1BD0, 1BD1, 1BD2, 1BD3, 1BD4, 1BD7, 1BD8, 1BD9, 1BDA, 1BDB, 1BDF, 1BDG, 1BDH, 1BDI, 1BDJ, 1BDL, 1BDM, 1BDN, 1BDO, 1BDQ, 1BDR, 1BDT, 1BDU, 1BDV, 1BDX, 1BDY, 1BE0, 1BE3, 1BE4, 1BE6, 1BE7, 1BE8, 1BE9, 1BEA, 1BEB, 1BEC, 1BED, 1BEE, 1BEH, 1BEJ, 1BEK, 1BEL, 1BEM, 1BEN, 1BEO, 1BEP, 1BEQ, 1BES, 1BET, 1BEU, 1BEX, 1BEY, 1BEZ, 1BF2, 1BF3, 1BF4, 1BF5, 1BF6, 1BFA, 1BFB, 1BFC, 1BFD, 1BFE, 1BFF, 1BFG, 1BFK, 1BFN, 1BFO, 1BFP, 1BFR, 1BFS, 1BFT, 1BFU, 1BFV, 1BG0, 1BG1, 1BG2, 1BG3, 1BG4, 1BG5, 1BG6, 1BG7, 1BG8, 1BG9, 1BGA, 1BGB, 1BGC, 1BGD, 1BGE, 1BGF, 1BGG, 1BGI, 1BGJ, 1BGL, 1BGM, 1BGN, 1BGO, 1BGP, 1BGQ, 1BGS, 1BGT, 1BGU, 1BGV, 1BGW, 1BGX, 1BGY, 1BH0, 1BH2, 1BH3, 1BH5, 1BH6, 1BH8, 1BH9, 1BHC, 1BHD, 1BHE, 1BHF, 1BHG, 1BHH, 1BHJ, 1BHL, 1BHM, 1BHN, 1BHO, 1BHP, 1BHQ, 1BHS, 1BHT, 1BHW, 1BHX, 1BHY, 1BHZ, 1BI0, 1BI1, 1BI2, 1BI3, 1BI4, 1BI5, 1BI7, 1BI8, 1BI9, 1BIA, 1BIB, 1BIC, 1BID, 1BIF, 1BIH, 1BII, 1BIJ, 1BIK, 1BIL, 1BIM, 1BIN, 1BIO, 1BIQ, 1BIR, 1BIS, 1BIT, 1BIU, 1BIW, 1BIX, 1BIY, 1BIZ, 1BJ0, 1BJ1, 1BJ3, 1BJ4, 1BJ5, 1BJ7, 1BJ9, 1BJA, 1BJE, 1BJF, 1BJG, 1BJI, 1BJJ, 1BJK, 1BJM, 1BJN, 1BJO, 1BJP, 1BJQ, 1BJR, 1BJT, 1BJU, 1BJV, 1BJW, 1BJY, 1BJZ, 1BK0, 1BK1, 1BK2, 1BK4, 1BK5, 1BK6, 1BK7, 1BK9, 1BKA, 1BKB, 1BKC, 1BKD, 1BKE, 1BKF, 1BKG, 1BKH, 1BKJ, 1BKL, 1BKM, 1BKN, 1BKO, 1BKP, 1BKR, 1BKS, 1BKW, 1BKX, 1BKY, 1BKZ, 1BL0, 1BL3, 1BL4, 1BL5, 1BL6, 1BL7, 1BL8, 1BL9, 1BLB, 1BLC, 1BLE, 1BLF, 1BLH, 1BLI, 1BLL, 1BLN, 1BLP, 1BLS, 1BLU, 1BLX, 1BLZ, 1BM0, 1BM1, 1BM2, 1BM3, 1BM7, 1BM8, 1BM9, 1BMA, 1BMB, 1BMC, 1BMD, 1BMF, 1BMG, 1BMK, 1BML, 1BMM, 1BMN, 1BMO, 1BMP, 1BMQ, 1BMS, 1BMT, 1BMZ, 1BN1, 1BN3, 1BN4, 1BN5, 1BN6, 1BN7, 1BN8, 1BNA, 1BNC, 1BND, 1BNE, 1BNF, 1BNG, 1BNI, 1BNJ, 1BNK, 1BNL, 1BNM, 1BNN, 1BNQ, 1BNS, 1BNT, 1BNU, 1BNV, 1BNW, 1BNZ, 1BO1, 1BO4, 1BO5, 1BO6, 1BO7, 1BO8, 1BOA, 1BOB, 1BOF, 1BOG, 1BOH, 1BOI, 1BOL, 1BOO, 1BOQ, 1BOS, 1BOT, 1BOU, 1BOW, 1BOX, 1BOY, 1BOZ, 1BP0, 1BP1, 1BP2, 1BP3, 1BP4, 1BP5, 1BP6, 1BP7, 1BPB, 1BPD, 1BPE, 1BPH, 1BPI, 1BPJ, 1BPL, 1BPM, 1BPN, 1BPO, 1BPQ, 1BPT, 1BPW, 1BPX, 1BPY, 1BPZ, 1BQ1, 1BQ2, 1BQ3, 1BQ4, 1BQ5, 1BQ6, 1BQ7, 1BQ8, 1BQ9, 1BQA, 1BQB, 1BQC, 1BQD, 1BQE, 1BQG, 1BQH, 1BQI, 1BQJ, 1BQK, 1BQL, 1BQM, 1BQN, 1BQO, 1BQP, 1BQQ, 1BQR, 1BQS, 1BQU, 1BQY, 1BR1, 1BR2, 1BR4, 1BR5, 1BR6, 1BR8, 1BR9, 1BRA, 1BRB, 1BRC, 1BRE, 1BRF, 1BRG, 1BRH, 1BRI, 1BRJ, 1BRK, 1BRL, 1BRM, 1BRO, 1BRP, 1BRQ, 1BRR, 1BRS, 1BRT, 1BRU, 1BRW, 1BRX, 1BRY, 1BS0, 1BS1, 1BS2, 1BS3, 1BS4, 1BS5, 1BS6, 1BS7, 1BS8, 1BS9, 1BSA, 1BSB, 1BSC, 1BSD, 1BSE, 1BSF, 1BSG, 1BSI, 1BSJ, 1BSK, 1BSL, 1BSM, 1BSO, 1BSP, 1BSQ, 1BSR, 1BSS, 1BSU, 1BSV, 1BSW, 1BSX, 1BSY, 1BSZ, 1BT0, 1BT1, 1BT2, 1BT3, 1BT4, 1BT5, 1BT6, 1BT8, 1BT9, 1BTC, 1BTE, 1BTG, 1BTH, 1BTI, 1BTJ, 1BTK, 1BTL, 1BTM, 1BTN, 1BTO, 1BTP, 1BTU, 1BTW, 1BTX, 1BTY, 1BTZ, 1BU1, 1BU2, 1BU3, 1BU4, 1BU5, 1BU6, 1BU7, 1BU8, 1BUA, 1BUC, 1BUD, 1BUE, 1BUG, 1BUH, 1BUI, 1BUL, 1BUN, 1BUO, 1BUP, 1BUU, 1BUV, 1BUW, 1BUX, 1BV1, 1BV3, 1BV4, 1BV7, 1BV9, 1BVA, 1BVB, 1BVC, 1BVD, 1BVI, 1BVK, 1BVL, 1BVN, 1BVO, 1BVP, 1BVQ, 1BVR, 1BVS, 1BVT, 1BVU, 1BVV, 1BVW, 1BVX, 1BVY, 1BVZ, 1BW0, 1BW8, 1BW9, 1BWA, 1BWB, 1BWC, 1BWD, 1BWF, 1BWH, 1BWI, 1BWJ, 1BWK, 1BWL, 1BWN, 1BWO, 1BWP, 1BWQ, 1BWR, 1BWS, 1BWU, 1BWV, 1BWW, 1BWZ, 1BX0, 1BX1, 1BX2, 1BX3, 1BX4, 1BX6, 1BX7, 1BX8, 1BXA, 1BXB, 1BXC, 1BXE, 1BXG, 1BXH, 1BXI, 1BXK, 1BXM, 1BXO, 1BXQ, 1BXR, 1BXS, 1BXT, 1BXU, 1BXV, 1BXW, 1BXX, 1BXY, 1BXZ, 1BY2, 1BY3, 1BY4, 1BY5, 1BY7, 1BY8, 1BY9, 1BYA, 1BYB, 1BYC, 1BYD, 1BYE, 1BYF, 1BYG, 1BYH, 1BYI, 1BYK, 1BYL, 1BYO, 1BYP, 1BYQ, 1BYR, 1BYS, 1BYU, 1BYW, 1BYZ, 1BZ0, 1BZ1, 1BZ4, 1BZ5, 1BZ6, 1BZ7, 1BZ8, 1BZ9, 1BZA, 1BZC, 1BZD, 1BZE, 1BZH, 1BZJ, 1BZL, 1BZM, 1BZO, 1BZP, 1BZQ, 1BZR, 1BZS, 1BZW, 1BZX, 1BZY, 1BZZ, 1C02, 1C03, 1C08, 1C09, 1C0B, 1C0C, 1C0E, 1C0F, 1C0G, 1C0I, 1C0K, 1C0L, 1C0M, 1C0N, 1C0P, 1C0T, 1C0U, 1C0W, 1C10, 1C12, 1C14, 1C16, 1C1A, 1C1B, 1C1C, 1C1D, 1C1E, 1C1F, 1C1G, 1C1H, 1C1J, 1C1K, 1C1L, 1C1M, 1C1N, 1C1O, 1C1P, 1C1Q, 1C1R, 1C1S, 1C1T, 1C1U, 1C1V, 1C1W, 1C1X, 1C1Y, 1C1Z, 1C21, 1C22, 1C23, 1C24, 1C25, 1C26, 1C27, 1C28, 1C29, 1C2A, 1C2D, 1C2E, 1C2F, 1C2G, 1C2H, 1C2I, 1C2J, 1C2K, 1C2L, 1C2M, 1C2P, 1C2R, 1C2T, 1C2Y, 1C30, 1C39, 1C3A, 1C3B, 1C3C, 1C3D, 1C3E, 1C3F, 1C3G, 1C3H, 1C3I, 1C3J, 1C3K, 1C3L, 1C3M, 1C3N, 1C3P, 1C3Q, 1C3R, 1C3S, 1C3U, 1C3V, 1C3W, 1C3X, 1C40, 1C41, 1C43, 1C44, 1C45, 1C46, 1C47, 1C48, 1C4A, 1C4C, 1C4F, 1C4G, 1C4K, 1C4O, 1C4P, 1C4Q, 1C4R, 1C4T, 1C4U, 1C4V, 1C4W, 1C4X, 1C4Y, 1C4Z, 1C50, 1C52, 1C5B, 1C5C, 1C5D, 1C5E, 1C5F, 1C5G, 1C5H, 1C5I, 1C5K, 1C5L, 1C5M, 1C5N, 1C5O, 1C5P, 1C5Q, 1C5R, 1C5S, 1C5T, 1C5U, 1C5V, 1C5W, 1C5X, 1C5Y, 1C5Z, 1C60, 1C61, 1C62, 1C63, 1C64, 1C65, 1C66, 1C67, 1C68, 1C69, 1C6A, 1C6B, 1C6C, 1C6D, 1C6E, 1C6F, 1C6G, 1C6H, 1C6I, 1C6J, 1C6K, 1C6L, 1C6M, 1C6N, 1C6O, 1C6P, 1C6Q, 1C6R, 1C6T, 1C6V, 1C6X, 1C6Y, 1C6Z, 1C70, 1C72, 1C74, 1C75, 1C76, 1C77, 1C78, 1C79, 1C7B, 1C7C, 1C7D, 1C7E, 1C7F, 1C7G, 1C7H, 1C7I, 1C7J, 1C7K, 1C7N, 1C7O, 1C7P, 1C7Q, 1C7R, 1C7S, 1C7T, 1C7Y, 1C7Z, 1C80, 1C81, 1C82, 1C83, 1C84, 1C85, 1C86, 1C87, 1C88, 1C8B, 1C8C, 1C8I, 1C8J, 1C8K, 1C8L, 1C8O, 1C8Q, 1C8R, 1C8S, 1C8T, 1C8U, 1C8V, 1C8W, 1C8X, 1C8Y, 1C8Z, 1C90, 1C91, 1C92, 1C93, 1C94, 1C96, 1C97, 1C9B, 1C9C, 1C9D, 1C9E, 1C9H, 1C9I, 1C9J, 1C9K, 1C9L, 1C9M, 1C9N, 1C9O, 1C9P, 1C9S, 1C9T, 1C9U, 1C9V, 1C9W, 1C9X, 1C9Y, 1C9Z, 1CA0, 1CA1, 1CA2, 1CA3, 1CA4, 1CA5, 1CA6, 1CA7, 1CA8, 1CA9, 1CAA, 1CAD, 1CAH, 1CAI, 1CAJ, 1CAK, 1CAL, 1CAM, 1CAN, 1CAO, 1CAQ, 1CAU, 1CAV, 1CAW, 1CAX, 1CAY, 1CAZ, 1CB0, 1CB2, 1CB4, 1CB5, 1CB6, 1CB7, 1CB8, 1CBF, 1CBG, 1CBI, 1CBJ, 1CBK, 1CBL, 1CBM, 1CBN, 1CBO, 1CBQ, 1CBR, 1CBS, 1CBU, 1CBV, 1CBW, 1CBX, 1CBY, 1CC0, 1CC1, 1CC2, 1CC3, 1CC4, 1CC5, 1CC6, 1CC7, 1CC8, 1CCA, 1CCB, 1CCC, 1CCD, 1CCE, 1CCG, 1CCI, 1CCJ, 1CCK, 1CCL, 1CCP, 1CCR, 1CCS, 1CCT, 1CCU, 1CCW, 1CCZ, 1CD0, 1CD1, 1CD2, 1CD5, 1CD8, 1CD9, 1CDC, 1CDD, 1CDE, 1CDG, 1CDH, 1CDI, 1CDJ, 1CDK, 1CDL, 1CDM, 1CDO, 1CDP, 1CDT, 1CDU, 1CDW, 1CDY, 1CDZ, 1CE0, 1CE1, 1CE2, 1CE5, 1CE6, 1CE7, 1CE9, 1CEA, 1CEB, 1CEC, 1CEF, 1CEG, 1CEH, 1CEI, 1CEL, 1CEM, 1CEN, 1CEO, 1CEQ, 1CER, 1CES, 1CET, 1CEV, 1CEW, 1CEX, 1CEZ, 1CF0, 1CF1, 1CF2, 1CF3, 1CF5, 1CF7, 1CF8, 1CF9, 1CFB, 1CFJ, 1CFM, 1CFN, 1CFQ, 1CFR, 1CFS, 1CFT, 1CFV, 1CFW, 1CFY, 1CFZ, 1CG0, 1CG1, 1CG2, 1CG3, 1CG4, 1CG5, 1CG6, 1CG8, 1CG9, 1CGC, 1CGE, 1CGF, 1CGH, 1CGI, 1CGJ, 1CGK, 1CGL, 1CGN, 1CGO, 1CGP, 1CGQ, 1CGS, 1CGT, 1CGU, 1CGV, 1CGW, 1CGX, 1CGY, 1CGZ, 1CH0, 1CH1, 1CH2, 1CH3, 1CH4, 1CH5, 1CH7, 1CH8, 1CH9, 1CHD, 1CHG, 1CHH, 1CHI, 1CHJ, 1CHK, 1CHM, 1CHN, 1CHO, 1CHP, 1CHQ, 1CHU, 1CHW, 1CHZ, 1CI0, 1CI1, 1CI3, 1CI4, 1CI6, 1CI7, 1CI8, 1CI9, 1CIA, 1CIB, 1CIC, 1CID, 1CIE, 1CIF, 1CIG, 1CIH, 1CII, 1CIJ, 1CIK, 1CIL, 1CIM, 1CIN, 1CIO, 1CIP, 1CIQ, 1CIT, 1CIU, 1CIV, 1CIW, 1CIY, 1CIZ, 1CJ0, 1CJ1, 1CJ2, 1CJ3, 1CJ4, 1CJ6, 1CJ7, 1CJ8, 1CJ9, 1CJA, 1CJB, 1CJC, 1CJD, 1CJE, 1CJF, 1CJK, 1CJL, 1CJM, 1CJP, 1CJQ, 1CJR, 1CJS, 1CJT, 1CJU, 1CJV, 1CJW, 1CJX, 1CJY, 1CK0, 1CK1, 1CK3, 1CK4, 1CK6, 1CK7, 1CKA, 1CKB, 1CKC, 1CKD, 1CKE, 1CKF, 1CKG, 1CKH, 1CKI, 1CKJ, 1CKL, 1CKM, 1CKN, 1CKO, 1CKP, 1CKQ, 1CKS, 1CKT, 1CKU, 1CL0, 1CL1, 1CL2, 1CL5, 1CL6, 1CL7, 1CL8, 1CLA, 1CLC, 1CLE, 1CLI, 1CLK, 1CLL, 1CLM, 1CLO, 1CLP, 1CLQ, 1CLS, 1CLU, 1CLV, 1CLW, 1CLX, 1CLY, 1CLZ, 1CM0, 1CM1, 1CM2, 1CM3, 1CM4, 1CM5, 1CM7, 1CM8, 1CM9, 1CMA, 1CMB, 1CMC, 1CMI, 1CMJ, 1CMK, 1CML, 1CMN, 1CMP, 1CMQ, 1CMS, 1CMT, 1CMU, 1CMV, 1CMX, 1CMY, 1CN0, 1CN1, 1CN3, 1CN4, 1CNB, 1CNC, 1CNE, 1CNF, 1CNG, 1CNH, 1CNI, 1CNJ, 1CNK, 1CNM, 1CNO, 1CNQ, 1CNR, 1CNS, 1CNT, 1CNU, 1CNV, 1CNW, 1CNX, 1CNY, 1CNZ, 1CO6, 1CO7, 1CO8, 1CO9, 1COA, 1COB, 1COF, 1COH, 1COI, 1COJ, 1COL, 1COM, 1CON, 1COS, 1COT, 1COW, 1COY, 1COZ, 1CP0, 1CP2, 1CP3, 1CP4, 1CP5, 1CP6, 1CP7, 1CP9, 1CPB, 1CPC, 1CPD, 1CPE, 1CPF, 1CPG, 1CPH, 1CPI, 1CPJ, 1CPM, 1CPN, 1CPO, 1CPQ, 1CPR, 1CPS, 1CPT, 1CPU, 1CPW, 1CPX, 1CPY, 1CQ1, 1CQ3, 1CQ4, 1CQ6, 1CQ7, 1CQ8, 1CQ9, 1CQA, 1CQD, 1CQE, 1CQF, 1CQI, 1CQJ, 1CQK, 1CQM, 1CQN, 1CQP, 1CQQ, 1CQR, 1CQS, 1CQT, 1CQV, 1CQW, 1CQX, 1CQY, 1CQZ, 1CR0, 1CR1, 1CR2, 1CR4, 1CR5, 1CR6, 1CR7, 1CR9, 1CRA, 1CRB, 1CRC, 1CRG, 1CRH, 1CRI, 1CRJ, 1CRK, 1CRL, 1CRM, 1CRN, 1CRU, 1CRW, 1CRX, 1CRY, 1CRZ, 1CS1, 1CS3, 1CS4, 1CS6, 1CS7, 1CS8, 1CSB, 1CSC, 1CSE, 1CSG, 1CSH, 1CSI, 1CSJ, 1CSK, 1CSM, 1CSN, 1CSO, 1CSP, 1CSQ, 1CSR, 1CSS, 1CSU, 1CSV, 1CSW, 1CSX, 1CT0, 1CT1, 1CT2, 1CT4, 1CT5, 1CT8, 1CT9, 1CTE, 1CTF, 1CTJ, 1CTM, 1CTN, 1CTP, 1CTQ, 1CTR, 1CTS, 1CTT, 1CTU, 1CTW, 1CTX, 1CTY, 1CTZ, 1CU0, 1CU1, 1CU2, 1CU3, 1CU4, 1CU5, 1CU6, 1CUA, 1CUB, 1CUC, 1CUD, 1CUE, 1CUF, 1CUG, 1CUH, 1CUI, 1CUJ, 1CUK, 1CUL, 1CUN, 1CUO, 1CUP, 1CUQ, 1CUS, 1CUU, 1CUV, 1CUW, 1CUX, 1CUY, 1CUZ, 1CV0, 1CV1, 1CV2, 1CV3, 1CV4, 1CV5, 1CV6, 1CV7, 1CV8, 1CVA, 1CVB, 1CVC, 1CVD, 1CVE, 1CVF, 1CVH, 1CVI, 1CVK, 1CVL, 1CVM, 1CVN, 1CVR, 1CVS, 1CVU, 1CVW, 1CVY, 1CVZ, 1CW0, 1CW1, 1CW2, 1CW3, 1CW4, 1CW7, 1CW9, 1CWA, 1CWB, 1CWC, 1CWD, 1CWE, 1CWF, 1CWH, 1CWI, 1CWJ, 1CWK, 1CWL, 1CWM, 1CWN, 1CWO, 1CWQ, 1CWR, 1CWS, 1CWT, 1CWU, 1CWV, 1CWY, 1CX2, 1CX4, 1CX6, 1CX7, 1CX8, 1CX9, 1CXA, 1CXC, 1CXE, 1CXF, 1CXH, 1CXI, 1CXK, 1CXL, 1CXP, 1CXQ, 1CXU, 1CXV, 1CXY, 1CXZ, 1CY0, 1CY1, 1CY2, 1CY4, 1CY5, 1CY6, 1CY7, 1CY8, 1CY9, 1CYC, 1CYD, 1CYF, 1CYG, 1CYI, 1CYJ, 1CYN, 1CYO, 1CYQ, 1CYW, 1CYX, 1CYY, 1CZ0, 1CZ1, 1CZ3, 1CZ7, 1CZ8, 1CZ9, 1CZA, 1CZB, 1CZC, 1CZD, 1CZE, 1CZF, 1CZG, 1CZH, 1CZI, 1CZJ, 1CZK, 1CZL, 1CZM, 1CZN, 1CZO, 1CZP, 1CZR, 1CZS, 1CZT, 1CZU, 1CZV, 1CZW, 1CZY, 1CZZ, 1D00, 1D01, 1D02, 1D03, 1D04, 1D06, 1D07, 1D09, 1D0A, 1D0B, 1D0C, 1D0D, 1D0E, 1D0G, 1D0H, 1D0I, 1D0J, 1D0K, 1D0L, 1D0M, 1D0N, 1D0O, 1D0Q, 1D0S, 1D0V, 1D0X, 1D0Y, 1D0Z, 1D13, 1D16, 1D1A, 1D1B, 1D1C, 1D1G, 1D1I, 1D1J, 1D1K, 1D1L, 1D1M, 1D1P, 1D1Q, 1D1S, 1D1T, 1D1U, 1D1V, 1D1W, 1D1X, 1D1Y, 1D1Z, 1D23, 1D24, 1D26, 1D27, 1D28, 1D29, 1D2A, 1D2C, 1D2E, 1D2F, 1D2G, 1D2H, 1D2I, 1D2K, 1D2M, 1D2N, 1D2O, 1D2P, 1D2Q, 1D2R, 1D2S, 1D2T, 1D2U, 1D2V, 1D2W, 1D2Y, 1D2Z, 1D30, 1D31, 1D39, 1D3A, 1D3B, 1D3C, 1D3D, 1D3F, 1D3G, 1D3H, 1D3J, 1D3K, 1D3L, 1D3M, 1D3N, 1D3P, 1D3Q, 1D3R, 1D3S, 1D3T, 1D3U, 1D3V, 1D3W, 1D3Y, 1D43, 1D44, 1D45, 1D46, 1D48, 1D49, 1D4A, 1D4C, 1D4D, 1D4E, 1D4F, 1D4H, 1D4I, 1D4J, 1D4K, 1D4L, 1D4N, 1D4O, 1D4P, 1D4S, 1D4T, 1D4V, 1D4W, 1D4X, 1D4Y, 1D4Z, 1D53, 1D56, 1D57, 1D59, 1D5A, 1D5B, 1D5C, 1D5D, 1D5E, 1D5F, 1D5H, 1D5I, 1D5J, 1D5L, 1D5M, 1D5N, 1D5R, 1D5S, 1D5T, 1D5W, 1D5X, 1D5Y, 1D5Z, 1D60, 1D61, 1D62, 1D63, 1D64, 1D65, 1D66, 1D6A, 1D6E, 1D6F, 1D6H, 1D6I, 1D6J, 1D6M, 1D6N, 1D6O, 1D6P, 1D6Q, 1D6R, 1D6S, 1D6U, 1D6V, 1D6W, 1D6Y, 1D6Z, 1D75, 1D76, 1D77, 1D78, 1D79, 1D7A, 1D7B, 1D7C, 1D7D, 1D7E, 1D7F, 1D7H, 1D7I, 1D7J, 1D7K, 1D7L, 1D7M, 1D7O, 1D7P, 1D7R, 1D7S, 1D7U, 1D7V, 1D7W, 1D7X, 1D7Y, 1D80, 1D81, 1D82, 1D85, 1D86, 1D87, 1D88, 1D89, 1D8A, 1D8C, 1D8D, 1D8E, 1D8F, 1D8G, 1D8H, 1D8I, 1D8L, 1D8M, 1D8S, 1D8T, 1D8U, 1D8W, 1D8X, 1D8Y, 1D90, 1D91, 1D92, 1D93, 1D98, 1D99, 1D9C, 1D9D, 1D9E, 1D9F, 1D9G, 1D9H, 1D9I, 1D9K, 1D9Q, 1D9R, 1D9U, 1D9V, 1D9W, 1D9X, 1D9Y, 1D9Z, 1DA1, 1DA2, 1DA3, 1DAA, 1DAB, 1DAD, 1DAE, 1DAF, 1DAG, 1DAH, 1DAI, 1DAJ, 1DAK, 1DAM, 1DAN, 1DAO, 1DAP, 1DAR, 1DAW, 1DAY, 1DAZ, 1DB1, 1DB2, 1DB3, 1DB4, 1DB5, 1DBA, 1DBB, 1DBF, 1DBG, 1DBH, 1DBI, 1DBJ, 1DBK, 1DBM, 1DBN, 1DBO, 1DBP, 1DBQ, 1DBR, 1DBS, 1DBT, 1DBU, 1DBV, 1DBW, 1DBX, 1DBZ, 1DC0, 1DC1, 1DC3, 1DC4, 1DC5, 1DC6, 1DC9, 1DCA, 1DCB, 1DCC, 1DCD, 1DCE, 1DCF, 1DCG, 1DCH, 1DCI, 1DCK, 1DCL, 1DCM, 1DCN, 1DCO, 1DCP, 1DCQ, 1DCR, 1DCS, 1DCT, 1DCU, 1DCV, 1DCW, 1DCY, 1DD1, 1DD3, 1DD4, 1DD5, 1DD6, 1DD7, 1DD8, 1DD9, 1DDE, 1DDG, 1DDH, 1DDI, 1DDJ, 1DDK, 1DDN, 1DDO, 1DDR, 1DDS, 1DDT, 1DDU, 1DDV, 1DDW, 1DDX, 1DDZ, 1DE0, 1DE4, 1DE5, 1DE6, 1DE7, 1DE8, 1DE9, 1DEA, 1DEB, 1DED, 1DEE, 1DEG, 1DEH, 1DEI, 1DEJ, 1DEK, 1DEL, 1DEO, 1DEQ, 1DET, 1DEU, 1DEV, 1DEW, 1DEX, 1DF0, 1DF1, 1DF4, 1DF5, 1DF7, 1DF8, 1DFA, 1DFB, 1DFC, 1DFF, 1DFG, 1DFH, 1DFI, 1DFJ, 1DFK, 1DFL, 1DFM, 1DFN, 1DFO, 1DFP, 1DFQ, 1DFV, 1DFX, 1DG1, 1DG3, 1DG5, 1DG6, 1DG7, 1DG8, 1DG9, 1DGB, 1DGC, 1DGE, 1DGF, 1DGG, 1DGH, 1DGJ, 1DGK, 1DGL, 1DGM, 1DGP, 1DGR, 1DGS, 1DGW, 1DH3, 1DHF, 1DHG, 1DHI, 1DHJ, 1DHK, 1DHN, 1DHP, 1DHR, 1DHS, 1DHT, 1DHY, 1DI0, 1DI1, 1DI3, 1DI4, 1DI5, 1DI6, 1DI7, 1DI8, 1DI9, 1DIA, 1DIB, 1DIC, 1DID, 1DIE, 1DIF, 1DIG, 1DIH, 1DII, 1DIK, 1DIL, 1DIM, 1DIN, 1DIO, 1DIQ, 1DIR, 1DIT, 1DIV, 1DIW, 1DIX, 1DIY, 1DIZ, 1DJ0, 1DJ1, 1DJ2, 1DJ3, 1DJ5, 1DJ6, 1DJ7, 1DJ8, 1DJ9, 1DJA, 1DJB, 1DJC, 1DJE, 1DJG, 1DJH, 1DJI, 1DJL, 1DJN, 1DJO, 1DJP, 1DJQ, 1DJR, 1DJS, 1DJT, 1DJU, 1DJW, 1DJX, 1DJY, 1DJZ, 1DK0, 1DK4, 1DK5, 1DK7, 1DK8, 1DKA, 1DKD, 1DKE, 1DKF, 1DKG, 1DKH, 1DKI, 1DKJ, 1DKK, 1DKL, 1DKM, 1DKN, 1DKO, 1DKP, 1DKQ, 1DKR, 1DKS, 1DKT, 1DKU, 1DKW, 1DKX, 1DKY, 1DKZ, 1DL2, 1DL3, 1DL5, 1DL7, 1DLA, 1DLB, 1DLC, 1DLE, 1DLF, 1DLG, 1DLH, 1DLI, 1DLJ, 1DLK, 1DLL, 1DLM, 1DLO, 1DLP, 1DLQ, 1DLR, 1DLS, 1DLT, 1DLU, 1DLV, 1DLW, 1DLY, 1DM0, 1DM1, 1DM2, 1DM3, 1DM4, 1DM5, 1DM6, 1DM7, 1DM8, 1DM9, 1DMA, 1DMB, 1DMG, 1DMH, 1DMI, 1DMJ, 1DMK, 1DML, 1DMM, 1DMN, 1DMP, 1DMQ, 1DMR, 1DMS, 1DMT, 1DMU, 1DMW, 1DMX, 1DMY, 1DN0, 1DN2, 1DN6, 1DN8, 1DN9, 1DNA, 1DNC, 1DNE, 1DNF, 1DNH, 1DNK, 1DNL, 1DNM, 1DNP, 1DNS, 1DNU, 1DNW, 1DNX, 1DNZ, 1DO0, 1DO1, 1DO2, 1DO3, 1DO4, 1DO5, 1DO6, 1DO7, 1DO8, 1DOA, 1DOB, 1DOC, 1DOD, 1DOE, 1DOF, 1DOG, 1DOH, 1DOI, 1DOJ, 1DOK, 1DOL, 1DOR, 1DOS, 1DOT, 1DOU, 1DOV, 1DOW, 1DOZ, 1DP0, 1DP2, 1DP4, 1DP5, 1DP6, 1DP7, 1DP8, 1DP9, 1DPC, 1DPE, 1DPF, 1DPG, 1DPH, 1DPI, 1DPJ, 1DPL, 1DPM, 1DPN, 1DPO, 1DPP, 1DPR, 1DPS, 1DPT, 1DPW, 1DPX, 1DPY, 1DPZ, 1DQ0, 1DQ1, 1DQ2, 1DQ3, 1DQ4, 1DQ5, 1DQ6, 1DQ7, 1DQ8, 1DQ9, 1DQA, 1DQD, 1DQE, 1DQG, 1DQI, 1DQJ, 1DQK, 1DQL, 1DQM, 1DQN, 1DQO, 1DQP, 1DQQ, 1DQR, 1DQS, 1DQT, 1DQU, 1DQV, 1DQW, 1DQX, 1DQY, 1DQZ, 1DR0, 1DR1, 1DR2, 1DR3, 1DR4, 1DR5, 1DR6, 1DR7, 1DR8, 1DR9, 1DRA, 1DRB, 1DRE, 1DRF, 1DRG, 1DRH, 1DRJ, 1DRK, 1DRM, 1DRT, 1DRU, 1DRV, 1DRW, 1DRY, 1DS0, 1DS1, 1DS2, 1DS3, 1DS4, 1DS5, 1DS6, 1DS7, 1DS8, 1DSB, 1DSE, 1DSF, 1DSG, 1DSL, 1DSN, 1DSO, 1DSP, 1DSS, 1DST, 1DSU, 1DSX, 1DSY, 1DSZ, 1DT0, 1DT1, 1DT2, 1DT3, 1DT4, 1DT5, 1DT6, 1DT9, 1DTD, 1DTE, 1DTG, 1DTH, 1DTI, 1DTJ, 1DTL, 1DTM, 1DTN, 1DTO, 1DTP, 1DTQ, 1DTS, 1DTT, 1DTU, 1DTW, 1DTX, 1DTY, 1DTZ, 1DU0, 1DU3, 1DU4, 1DU5, 1DUA, 1DUB, 1DUC, 1DUD, 1DUE, 1DUG, 1DUI, 1DUK, 1DUN, 1DUO, 1DUP, 1DUR, 1DUS, 1DUT, 1DUV, 1DUW, 1DUX, 1DUY, 1DUZ, 1DV1, 1DV2, 1DV3, 1DV4, 1DV6, 1DV7, 1DV8, 1DVA, 1DVB, 1DVE, 1DVF, 1DVG, 1DVI, 1DVJ, 1DVK, 1DVL, 1DVM, 1DVN, 1DVO, 1DVP, 1DVQ, 1DVR, 1DVS, 1DVT, 1DVU, 1DVX, 1DVY, 1DVZ, 1DW0, 1DW1, 1DW2, 1DW3, 1DW6, 1DW9, 1DWA, 1DWB, 1DWC, 1DWD, 1DWE, 1DWF, 1DWG, 1DWH, 1DWI, 1DWJ, 1DWK, 1DWO, 1DWP, 1DWQ, 1DWR, 1DWS, 1DWT, 1DWU, 1DWV, 1DWW, 1DWX, 1DX4, 1DX5, 1DX6, 1DX9, 1DXC, 1DXD, 1DXE, 1DXF, 1DXG, 1DXI, 1DXJ, 1DXK, 1DXL, 1DXM, 1DXO, 1DXP, 1DXQ, 1DXR, 1DXS, 1DXT, 1DXU, 1DXV, 1DXX, 1DXY, 1DY0, 1DY1, 1DY2, 1DY3, 1DY4, 1DY5, 1DY6, 1DY7, 1DY8, 1DY9, 1DYA, 1DYB, 1DYC, 1DYD, 1DYE, 1DYF, 1DYG, 1DYH, 1DYI, 1DYJ, 1DYK, 1DYM, 1DYN, 1DYO, 1DYP, 1DYQ, 1DYR, 1DYS, 1DYT, 1DYU, 1DYW, 1DYZ, 1DZ0, 1DZ3, 1DZ4, 1DZ6, 1DZ8, 1DZ9, 1DZA, 1DZB, 1DZE, 1DZF, 1DZG, 1DZH, 1DZI, 1DZJ, 1DZK, 1DZM, 1DZN, 1DZO, 1DZP, 1DZQ, 1DZR, 1DZT, 1DZU, 1DZV, 1DZW, 1DZX, 1DZY, 1DZZ, 1E00, 1E02, 1E03, 1E04, 1E05, 1E06, 1E0B, 1E0C, 1E0D, 1E0F, 1E0J, 1E0K, 1E0O, 1E0P, 1E0R, 1E0S, 1E0T, 1E0U, 1E0V, 1E0W, 1E0X, 1E0Y, 1E12, 1E14, 1E15, 1E18, 1E19, 1E1A, 1E1C, 1E1D, 1E1E, 1E1F, 1E1H, 1E1K, 1E1L, 1E1M, 1E1N, 1E1O, 1E1Q, 1E1R, 1E1T, 1E1V, 1E1X, 1E1Y, 1E1Z, 1E20, 1E21, 1E22, 1E24, 1E25, 1E26, 1E27, 1E28, 1E29, 1E2A, 1E2D, 1E2E, 1E2F, 1E2G, 1E2H, 1E2I, 1E2J, 1E2K, 1E2L, 1E2M, 1E2N, 1E2P, 1E2Q, 1E2R, 1E2S, 1E2T, 1E2U, 1E2V, 1E2W, 1E2X, 1E2Y, 1E2Z, 1E30, 1E31, 1E32, 1E33, 1E34, 1E35, 1E36, 1E37, 1E38, 1E39, 1E3A, 1E3B, 1E3C, 1E3D, 1E3E, 1E3F, 1E3G, 1E3H, 1E3I, 1E3J, 1E3K, 1E3L, 1E3M, 1E3O, 1E3P, 1E3Q, 1E3R, 1E3S, 1E3U, 1E3V, 1E3W, 1E3X, 1E3Z, 1E40, 1E42, 1E43, 1E44, 1E46, 1E47, 1E48, 1E49, 1E4A, 1E4B, 1E4C, 1E4D, 1E4E, 1E4F, 1E4G, 1E4H, 1E4I, 1E4J, 1E4K, 1E4L, 1E4M, 1E4N, 1E4O, 1E4V, 1E4W, 1E4X, 1E4Y, 1E50, 1E51, 1E54, 1E55, 1E56, 1E58, 1E59, 1E5A, 1E5D, 1E5E, 1E5F, 1E5H, 1E5I, 1E5J, 1E5K, 1E5L, 1E5M, 1E5N, 1E5O, 1E5P, 1E5Q, 1E5R, 1E5S, 1E5T, 1E5V, 1E5W, 1E5X, 1E5Y, 1E5Z, 1E60, 1E61, 1E62, 1E63, 1E64, 1E65, 1E66, 1E67, 1E69, 1E6A, 1E6B, 1E6C, 1E6D, 1E6E, 1E6F, 1E6G, 1E6H, 1E6I, 1E6J, 1E6K, 1E6L, 1E6M, 1E6N, 1E6O, 1E6P, 1E6Q, 1E6R, 1E6S, 1E6U, 1E6V, 1E6W, 1E6X, 1E6Y, 1E6Z, 1E70, 1E71, 1E72, 1E73, 1E77, 1E78, 1E79, 1E7A, 1E7B, 1E7C, 1E7D, 1E7E, 1E7F, 1E7G, 1E7H, 1E7I, 1E7L, 1E7M, 1E7N, 1E7O, 1E7P, 1E7Q, 1E7R, 1E7S, 1E7U, 1E7V, 1E7W, 1E7Y, 1E7Z, 1E80, 1E81, 1E82, 1E83, 1E84, 1E85, 1E86, 1E87, 1E89, 1E8A, 1E8C, 1E8D, 1E8F, 1E8G, 1E8H, 1E8I, 1E8K, 1E8M, 1E8N, 1E8S, 1E8T, 1E8U, 1E8V, 1E8W, 1E8X, 1E8Y, 1E8Z, 1E90, 1E92, 1E93, 1E94, 1E96, 1E97, 1E98, 1E99, 1E9A, 1E9B, 1E9C, 1E9D, 1E9E, 1E9F, 1E9G, 1E9H, 1E9I, 1E9L, 1E9M, 1E9N, 1E9O, 1E9P, 1E9Q, 1E9R, 1E9S, 1E9V, 1E9X, 1E9Y, 1E9Z, 1EA0, 1EA1, 1EA2, 1EA3, 1EA4, 1EA5, 1EA6, 1EA7, 1EA8, 1EA9, 1EAA, 1EAB, 1EAC, 1EAD, 1EAE, 1EAF, 1EAG, 1EAI, 1EAJ, 1EAK, 1EAM, 1EAN, 1EAO, 1EAP, 1EAQ, 1EAR, 1EAS, 1EAT, 1EAU, 1EAV, 1EAW, 1EAX, 1EAY, 1EAZ, 1EB0, 1EB1, 1EB2, 1EB3, 1EB4, 1EB6, 1EB7, 1EB8, 1EB9, 1EBA, 1EBB, 1EBC, 1EBD, 1EBE, 1EBF, 1EBG, 1EBH, 1EBK, 1EBL, 1EBM, 1EBO, 1EBP, 1EBT, 1EBU, 1EBV, 1EBW, 1EBY, 1EBZ, 1EC0, 1EC1, 1EC2, 1EC3, 1EC5, 1EC7, 1EC8, 1EC9, 1ECA, 1ECB, 1ECC, 1ECD, 1ECE, 1ECF, 1ECG, 1ECJ, 1ECL, 1ECM, 1ECN, 1ECO, 1ECP, 1ECQ, 1ECR, 1ECS, 1ECV, 1ECW, 1ECX, 1ECZ, 1ED1, 1ED3, 1ED4, 1ED5, 1ED6, 1ED8, 1ED9, 1EDB, 1EDD, 1EDE, 1EDG, 1EDH, 1EDM, 1EDN, 1EDO, 1EDQ, 1EDR, 1EDT, 1EDU, 1EDY, 1EDZ, 1EE0, 1EE1, 1EE2, 1EE3, 1EE4, 1EE5, 1EE6, 1EE8, 1EE9, 1EEA, 1EED, 1EEF, 1EEH, 1EEI, 1EEJ, 1EEL, 1EEM, 1EEN, 1EEO, 1EEP, 1EEQ, 1EER, 1EET, 1EEU, 1EEX, 1EEY, 1EEZ, 1EF0, 1EF1, 1EF2, 1EF3, 1EF7, 1EF8, 1EF9, 1EFA, 1EFC, 1EFD, 1EFG, 1EFH, 1EFI, 1EFK, 1EFL, 1EFM, 1EFN, 1EFP, 1EFQ, 1EFR, 1EFT, 1EFU, 1EFV, 1EFX, 1EFY, 1EFZ, 1EG1, 1EG2, 1EG3, 1EG4, 1EG5, 1EG6, 1EG7, 1EG9, 1EGA, 1EGC, 1EGD, 1EGE, 1EGG, 1EGH, 1EGI, 1EGJ, 1EGM, 1EGN, 1EGP, 1EGQ, 1EGU, 1EGV, 1EGW, 1EGY, 1EGZ, 1EH1, 1EH3, 1EH4, 1EH5, 1EH6, 1EH7, 1EH8, 1EH9, 1EHA, 1EHB, 1EHC, 1EHD, 1EHE, 1EHF, 1EHG, 1EHH, 1EHI, 1EHK, 1EHL, 1EHN, 1EHV, 1EHW, 1EHY, 1EI1, 1EI3, 1EI5, 1EI6, 1EI7, 1EI9, 1EIA, 1EIB, 1EIC, 1EID, 1EIE, 1EIF, 1EIL, 1EIN, 1EIQ, 1EIR, 1EIS, 1EIX, 1EIZ, 1EJ0, 1EJ1, 1EJ2, 1EJ3, 1EJ4, 1EJ7, 1EJ8, 1EJ9, 1EJA, 1EJB, 1EJC, 1EJD, 1EJE, 1EJF, 1EJG, 1EJH, 1EJI, 1EJJ, 1EJL, 1EJM, 1EJN, 1EJO, 1EJR, 1EJS, 1EJT, 1EJU, 1EJV, 1EJW, 1EJX, 1EJY, 1EK0, 1EK1, 1EK2, 1EK3, 1EK4, 1EK5, 1EK6, 1EK8, 1EK9, 1EKB, 1EKE, 1EKF, 1EKG, 1EKJ, 1EKK, 1EKL, 1EKM, 1EKO, 1EKP, 1EKQ, 1EKR, 1EKS, 1EKU, 1EKV, 1EKX, 1EL1, 1EL3, 1EL4, 1EL5, 1EL6, 1EL7, 1EL8, 1EL9, 1ELA, 1ELB, 1ELC, 1ELD, 1ELE, 1ELF, 1ELG, 1ELI, 1ELJ, 1ELK, 1ELL, 1ELM, 1ELO, 1ELP, 1ELQ, 1ELR, 1ELS, 1ELT, 1ELU, 1ELV, 1ELW, 1ELX, 1ELY, 1ELZ, 1EM1, 1EM2, 1EM6, 1EM7, 1EM8, 1EM9, 1EMA, 1EMB, 1EMC, 1EMD, 1EME, 1EMF, 1EMG, 1EMH, 1EMI, 1EMJ, 1EMK, 1EML, 1EMM, 1EMR, 1EMS, 1EMT, 1EMU, 1EMV, 1EMY, 1EN2, 1EN3, 1EN4, 1EN5, 1EN6, 1EN7, 1EN8, 1EN9, 1ENA, 1ENC, 1ENE, 1ENF, 1ENH, 1ENI, 1ENJ, 1ENK, 1ENM, 1ENN, 1ENO, 1ENP, 1ENQ, 1ENR, 1ENS, 1ENT, 1ENU, 1ENV, 1ENX, 1ENY, 1ENZ, 1EO2, 1EO3, 1EO4, 1EO5, 1EO6, 1EO7, 1EO8, 1EO9, 1EOA, 1EOB, 1EOC, 1EOD, 1EOE, 1EOF, 1EOG, 1EOH, 1EOI, 1EOJ, 1EOK, 1EOL, 1EOM, 1EON, 1EOO, 1EOP, 1EOS, 1EOU, 1EOV, 1EOW, 1EP0, 1EP1, 1EP2, 1EP3, 1EP4, 1EP5, 1EP6, 1EP7, 1EP8, 1EP9, 1EPA, 1EPB, 1EPF, 1EPL, 1EPM, 1EPN, 1EPO, 1EPP, 1EPQ, 1EPR, 1EPS, 1EPT, 1EPU, 1EPV, 1EPW, 1EPX, 1EPY, 1EPZ, 1EQ2, 1EQ4, 1EQ5, 1EQ6, 1EQ7, 1EQ9, 1EQA, 1EQB, 1EQC, 1EQD, 1EQE, 1EQF, 1EQG, 1EQH, 1EQJ, 1EQM, 1EQN, 1EQP, 1EQQ, 1EQR, 1EQT, 1EQU, 1EQV, 1EQW, 1EQY, 1EQZ, 1ER8, 1ERB, 1ERE, 1ERI, 1ERJ, 1ERK, 1ERM, 1ERN, 1ERO, 1ERQ, 1ERR, 1ERT, 1ERU, 1ERV, 1ERW, 1ERX, 1ERZ, 1ES0, 1ES1, 1ES2, 1ES3, 1ES4, 1ES5, 1ES6, 1ES7, 1ES8, 1ES9, 1ESA, 1ESB, 1ESC, 1ESD, 1ESE, 1ESF, 1ESG, 1ESI, 1ESJ, 1ESL, 1ESM, 1ESN, 1ESO, 1ESP, 1ESQ, 1ESR, 1EST, 1ESU, 1ESV, 1ESW, 1ESZ, 1ET0, 1ET1, 1ET5, 1ET6, 1ET7, 1ET8, 1ET9, 1ETA, 1ETB, 1ETE, 1ETH, 1ETJ, 1ETK, 1ETL, 1ETM, 1ETN, 1ETO, 1ETP, 1ETQ, 1ETR, 1ETS, 1ETT, 1ETU, 1ETV, 1ETW, 1ETX, 1ETY, 1ETZ, 1EU1, 1EU3, 1EU4, 1EU5, 1EU8, 1EUA, 1EUC, 1EUD, 1EUE, 1EUF, 1EUG, 1EUH, 1EUI, 1EUJ, 1EUM, 1EUN, 1EUO, 1EUP, 1EUR, 1EUS, 1EUT, 1EUU, 1EUV, 1EUW, 1EUZ, 1EV2, 1EV3, 1EV4, 1EV5, 1EV6, 1EV7, 1EV8, 1EV9, 1EVE, 1EVF, 1EVG, 1EVH, 1EVI, 1EVJ, 1EVK, 1EVL, 1EVP, 1EVQ, 1EVR, 1EVS, 1EVT, 1EVU, 1EVW, 1EVX, 1EVY, 1EVZ, 1EW0, 1EW2, 1EW3, 1EW4, 1EW6, 1EW8, 1EW9, 1EWA, 1EWC, 1EWD, 1EWE, 1EWF, 1EWH, 1EWJ, 1EWK, 1EWL, 1EWM, 1EWN, 1EWO, 1EWP, 1EWQ, 1EWR, 1EWT, 1EWV, 1EWX, 1EWY, 1EWZ, 1EX0, 1EX1, 1EX2, 1EX3, 1EX4, 1EX5, 1EX6, 1EX7, 1EX8, 1EX9, 1EXA, 1EXB, 1EXC, 1EXF, 1EXI, 1EXJ, 1EXM, 1EXN, 1EXP, 1EXQ, 1EXR, 1EXS, 1EXT, 1EXU, 1EXV, 1EXW, 1EXX, 1EXZ, 1EY0, 1EY2, 1EY3, 1EY4, 1EY5, 1EY6, 1EY7, 1EY8, 1EY9, 1EYA, 1EYB, 1EYC, 1EYD, 1EYE, 1EYG, 1EYH, 1EYI, 1EYJ, 1EYK, 1EYL, 1EYM, 1EYN, 1EYP, 1EYQ, 1EYR, 1EYS, 1EYT, 1EYU, 1EYV, 1EYW, 1EYX, 1EYY, 1EYZ, 1EZ0, 1EZ1, 1EZ2, 1EZ3, 1EZ4, 1EZ6, 1EZ8, 1EZ9, 1EZF, 1EZG, 1EZI, 1EZJ, 1EZK, 1EZL, 1EZM, 1EZQ, 1EZR, 1EZS, 1EZU, 1EZV, 1EZW, 1EZX, 1EZZ, 1F00, 1F02, 1F05, 1F06, 1F07, 1F08, 1F09, 1F0B, 1F0C, 1F0I, 1F0J, 1F0K, 1F0L, 1F0M, 1F0N, 1F0O, 1F0P, 1F0Q, 1F0R, 1F0S, 1F0T, 1F0U, 1F0V, 1F0W, 1F0X, 1F0Y, 1F10, 1F11, 1F12, 1F13, 1F14, 1F17, 1F18, 1F1A, 1F1B, 1F1C, 1F1D, 1F1E, 1F1F, 1F1G, 1F1H, 1F1J, 1F1M, 1F1O, 1F1R, 1F1S, 1F1U, 1F1V, 1F1W, 1F1X, 1F1Z, 1F20, 1F21, 1F23, 1F24, 1F25, 1F26, 1F28, 1F29, 1F2A, 1F2B, 1F2C, 1F2D, 1F2E, 1F2F, 1F2I, 1F2J, 1F2K, 1F2L, 1F2M, 1F2O, 1F2P, 1F2Q, 1F2S, 1F2T, 1F2U, 1F2V, 1F2W, 1F2X, 1F2Y, 1F2Z, 1F30, 1F31, 1F32, 1F33, 1F34, 1F35, 1F36, 1F37, 1F38, 1F39, 1F3A, 1F3B, 1F3D, 1F3E, 1F3F, 1F3G, 1F3H, 1F3J, 1F3L, 1F3M, 1F3O, 1F3P, 1F3T, 1F3U, 1F3V, 1F3W, 1F3X, 1F3Z, 1F41, 1F42, 1F44, 1F45, 1F46, 1F47, 1F48, 1F49, 1F4A, 1F4B, 1F4C, 1F4D, 1F4E, 1F4F, 1F4G, 1F4H, 1F4J, 1F4K, 1F4L, 1F4M, 1F4N, 1F4O, 1F4P, 1F4Q, 1F4R, 1F4T, 1F4U, 1F4V, 1F4W, 1F4X, 1F4Y, 1F4Z, 1F50, 1F51, 1F52, 1F56, 1F57, 1F58, 1F59, 1F5A, 1F5B, 1F5C, 1F5F, 1F5J, 1F5K, 1F5L, 1F5M, 1F5N, 1F5O, 1F5P, 1F5Q, 1F5R, 1F5S, 1F5T, 1F5V, 1F5W, 1F5Z, 1F60, 1F61, 1F63, 1F65, 1F66, 1F69, 1F6A, 1F6B, 1F6C, 1F6D, 1F6E, 1F6F, 1F6I, 1F6J, 1F6K, 1F6L, 1F6M, 1F6N, 1F6O, 1F6P, 1F6R, 1F6S, 1F6T, 1F6W, 1F6Y, 1F73, 1F74, 1F75, 1F76, 1F77, 1F7A, 1F7B, 1F7C, 1F7D, 1F7K, 1F7L, 1F7N, 1F7O, 1F7P, 1F7Q, 1F7R, 1F7S, 1F7T, 1F7Z, 1F80, 1F82, 1F86, 1F88, 1F89, 1F8A, 1F8B, 1F8C, 1F8D, 1F8E, 1F8F, 1F8G, 1F8I, 1F8M, 1F8N, 1F8Q, 1F8R, 1F8S, 1F8T, 1F8U, 1F8W, 1F8X, 1F8Y, 1F90, 1F91, 1F92, 1F93, 1F94, 1F97, 1F98, 1F99, 1F9A, 1F9B, 1F9C, 1F9D, 1F9E, 1F9F, 1F9G, 1F9H, 1F9I, 1F9J, 1F9K, 1F9M, 1F9N, 1F9O, 1F9P, 1F9Q, 1F9R, 1F9S, 1F9T, 1F9U, 1F9V, 1F9W, 1F9Z, 1FA0, 1FA2, 1FA5, 1FA6, 1FA7, 1FA8, 1FA9, 1FAA, 1FAE, 1FAG, 1FAH, 1FAI, 1FAJ, 1FAK, 1FAN, 1FAO, 1FAP, 1FAS, 1FAT, 1FAV, 1FAW, 1FAX, 1FAY, 1FAZ, 1FB0, 1FB1, 1FB2, 1FB5, 1FB6, 1FB7, 1FB8, 1FBA, 1FBC, 1FBD, 1FBE, 1FBF, 1FBG, 1FBH, 1FBI, 1FBL, 1FBM, 1FBN, 1FBO, 1FBP, 1FBQ, 1FBS, 1FBT, 1FBU, 1FBV, 1FBW, 1FBX, 1FBY, 1FBZ, 1FC0, 1FC1, 1FC2, 1FC3, 1FC4, 1FC5, 1FC6, 1FC7, 1FC9, 1FCA, 1FCB, 1FCC, 1FCD, 1FCE, 1FCF, 1FCG, 1FCH, 1FCJ, 1FCK, 1FCM, 1FCN, 1FCO, 1FCP, 1FCQ, 1FCS, 1FCU, 1FCV, 1FCX, 1FCY, 1FCZ, 1FD0, 1FD2, 1FD3, 1FD4, 1FD5, 1FD7, 1FD9, 1FDA, 1FDB, 1FDD, 1FDG, 1FDH, 1FDI, 1FDJ, 1FDK, 1FDL, 1FDN, 1FDO, 1FDP, 1FDQ, 1FDR, 1FDS, 1FDT, 1FDU, 1FDV, 1FDW, 1FDY, 1FDZ, 1FE0, 1FE2, 1FE3, 1FE4, 1FE5, 1FE6, 1FE8, 1FEA, 1FEB, 1FEC, 1FEE, 1FEH, 1FEJ, 1FEL, 1FEM, 1FEN, 1FEP, 1FER, 1FEV, 1FEW, 1FEZ, 1FF0, 1FF2, 1FF3, 1FF4, 1FF5, 1FF9, 1FFA, 1FFB, 1FFC, 1FFD, 1FFE, 1FFF, 1FFG, 1FFH, 1FFI, 1FFL, 1FFN, 1FFO, 1FFP, 1FFQ, 1FFR, 1FFS, 1FFT, 1FFU, 1FFV, 1FFW, 1FFX, 1FG2, 1FG3, 1FG4, 1FG5, 1FG6, 1FG7, 1FG8, 1FG9, 1FGA, 1FGB, 1FGC, 1FGG, 1FGH, 1FGI, 1FGJ, 1FGK, 1FGL, 1FGM, 1FGN, 1FGO, 1FGQ, 1FGR, 1FGS, 1FGT, 1FGU, 1FGV, 1FGX, 1FGY, 1FGZ, 1FH0, 1FH2, 1FH5, 1FH7, 1FH8, 1FH9, 1FHA, 1FHD, 1FHE, 1FHF, 1FHG, 1FHH, 1FHI, 1FHJ, 1FHL, 1FHM, 1FHN, 1FHU, 1FHV, 1FHW, 1FHX, 1FHY, 1FHZ, 1FI1, 1FI2, 1FI4, 1FI8, 1FIA, 1FIB, 1FIC, 1FID, 1FIE, 1FIF, 1FIG, 1FIH, 1FIK, 1FIL, 1FIM, 1FIN, 1FIO, 1FIP, 1FIQ, 1FIT, 1FIU, 1FIV, 1FIW, 1FIY, 1FIZ, 1FJ0, 1FJ1, 1FJ2, 1FJ3, 1FJ4, 1FJ6, 1FJ8, 1FJ9, 1FJH, 1FJJ, 1FJL, 1FJM, 1FJO, 1FJQ, 1FJR, 1FJS, 1FJT, 1FJU, 1FJV, 1FJW, 1FJX, 1FK0, 1FK1, 1FK2, 1FK3, 1FK4, 1FK5, 1FK6, 1FK7, 1FK8, 1FK9, 1FKB, 1FKD, 1FKF, 1FKG, 1FKH, 1FKI, 1FKJ, 1FKK, 1FKL, 1FKM, 1FKN, 1FKO, 1FKP, 1FKQ, 1FKV, 1FKW, 1FKX, 1FL0, 1FL1, 1FL2, 1FL3, 1FL5, 1FL6, 1FL7, 1FL9, 1FLA, 1FLC, 1FLD, 1FLE, 1FLG, 1FLH, 1FLJ, 1FLK, 1FLL, 1FLM, 1FLN, 1FLO, 1FLP, 1FLQ, 1FLR, 1FLT, 1FLU, 1FLV, 1FLW, 1FLY, 1FLZ, 1FM0, 1FM2, 1FM4, 1FM5, 1FM6, 1FM7, 1FM8, 1FM9, 1FMA, 1FMB, 1FMC, 1FMG, 1FMI, 1FMJ, 1FMK, 1FML, 1FMO, 1FMQ, 1FMS, 1FMT, 1FMU, 1FMV, 1FMW, 1FMX, 1FMZ, 1FN0, 1FN2, 1FN3, 1FN4, 1FN5, 1FN6, 1FN7, 1FN8, 1FN9, 1FNA, 1FNB, 1FNC, 1FND, 1FNE, 1FNF, 1FNG, 1FNH, 1FNI, 1FNJ, 1FNK, 1FNL, 1FNM, 1FNN, 1FNO, 1FNP, 1FNQ, 1FNS, 1FNT, 1FNU, 1FNV, 1FNW, 1FNY, 1FNZ, 1FO0, 1FO1, 1FO2, 1FO3, 1FO4, 1FO6, 1FO8, 1FO9, 1FOA, 1FOB, 1FOC, 1FOE, 1FOF, 1FOH, 1FOI, 1FOJ, 1FOK, 1FOL, 1FON, 1FOO, 1FOP, 1FOR, 1FOS, 1FOT, 1FOU, 1FP1, 1FP2, 1FP3, 1FP4, 1FP5, 1FP6, 1FP7, 1FP8, 1FP9, 1FPB, 1FPC, 1FPD, 1FPE, 1FPF, 1FPG, 1FPH, 1FPI, 1FPJ, 1FPK, 1FPL, 1FPM, 1FPO, 1FPP, 1FPQ, 1FPR, 1FPS, 1FPT, 1FPU, 1FPX, 1FPY, 1FPZ, 1FQ0, 1FQ1, 1FQ2, 1FQ3, 1FQ4, 1FQ5, 1FQ6, 1FQ7, 1FQ8, 1FQ9, 1FQA, 1FQB, 1FQC, 1FQD, 1FQE, 1FQF, 1FQG, 1FQI, 1FQJ, 1FQK, 1FQL, 1FQM, 1FQN, 1FQO, 1FQR, 1FQT, 1FQV, 1FQW, 1FQX, 1FR1, 1FR2, 1FR3, 1FR4, 1FR5, 1FR6, 1FR7, 1FR8, 1FR9, 1FRB, 1FRD, 1FRF, 1FRG, 1FRH, 1FRI, 1FRJ, 1FRK, 1FRL, 1FRM, 1FRN, 1FRO, 1FRP, 1FRQ, 1FRR, 1FRS, 1FRT, 1FRV, 1FRW, 1FRX, 1FRZ, 1FS0, 1FS1, 1FS2, 1FS3, 1FS4, 1FS5, 1FS6, 1FS7, 1FS8, 1FS9, 1FSA, 1FSC, 1FSE, 1FSF, 1FSG, 1FSI, 1FSJ, 1FSK, 1FSL, 1FSN, 1FSO, 1FSQ, 1FSR, 1FSS, 1FST, 1FSU, 1FSW, 1FSX, 1FSY, 1FSZ, 1FT0, 1FT1, 1FT2, 1FT3, 1FT4, 1FT5, 1FT6, 1FT7, 1FT8, 1FT9, 1FTA, 1FTC, 1FTD, 1FTE, 1FTF, 1FTG, 1FTH, 1FTJ, 1FTK, 1FTL, 1FTM, 1FTN, 1FTO, 1FTP, 1FTQ, 1FTR, 1FTS, 1FTW, 1FTX, 1FTY, 1FU0, 1FU1, 1FU4, 1FU7, 1FU8, 1FUA, 1FUE, 1FUG, 1FUI, 1FUJ, 1FUK, 1FUN, 1FUO, 1FUP, 1FUQ, 1FUR, 1FUS, 1FUT, 1FUU, 1FUX, 1FUY, 1FV0, 1FV1, 1FV2, 1FV3, 1FV9, 1FVA, 1FVC, 1FVD, 1FVE, 1FVF, 1FVG, 1FVH, 1FVI, 1FVJ, 1FVK, 1FVO, 1FVP, 1FVR, 1FVT, 1FVU, 1FVV, 1FVX, 1FW0, 1FW1, 1FW2, 1FW3, 1FW4, 1FW6, 1FW8, 1FW9, 1FWA, 1FWB, 1FWC, 1FWD, 1FWE, 1FWF, 1FWG, 1FWH, 1FWI, 1FWJ, 1FWK, 1FWL, 1FWM, 1FWN, 1FWR, 1FWS, 1FWT, 1FWU, 1FWV, 1FWW, 1FWX, 1FWY, 1FWZ, 1FX0, 1FX1, 1FX2, 1FX3, 1FX4, 1FX5, 1FX6, 1FX7, 1FX8, 1FX9, 1FXA, 1FXD, 1FXF, 1FXH, 1FXI, 1FXJ, 1FXK, 1FXO, 1FXP, 1FXQ, 1FXR, 1FXS, 1FXU, 1FXV, 1FXW, 1FXX, 1FXY, 1FXZ, 1FY1, 1FY2, 1FY3, 1FY4, 1FY5, 1FY6, 1FY7, 1FY8, 1FY9, 1FYA, 1FYD, 1FYE, 1FYF, 1FYH, 1FYK, 1FYL, 1FYM, 1FYN, 1FYR, 1FYS, 1FYT, 1FYU, 1FYV, 1FYW, 1FYX, 1FYZ, 1FZ0, 1FZ1, 1FZ2, 1FZ3, 1FZ4, 1FZ5, 1FZ6, 1FZ7, 1FZ8, 1FZ9, 1FZA, 1FZB, 1FZC, 1FZD, 1FZE, 1FZF, 1FZG, 1FZH, 1FZI, 1FZJ, 1FZK, 1FZM, 1FZO, 1FZP, 1FZQ, 1FZR, 1FZU, 1FZV, 1FZW, 1FZY, 1FZZ, 1G00, 1G01, 1G02, 1G05, 1G06, 1G07, 1G08, 1G09, 1G0A, 1G0B, 1G0C, 1G0D, 1G0E, 1G0F, 1G0G, 1G0H, 1G0I, 1G0J, 1G0K, 1G0L, 1G0M, 1G0N, 1G0O, 1G0P, 1G0Q, 1G0R, 1G0S, 1G0T, 1G0U, 1G0V, 1G0W, 1G0X, 1G0Y, 1G0Z, 1G12, 1G13, 1G15, 1G16, 1G17, 1G18, 1G19, 1G1A, 1G1B, 1G1C, 1G1D, 1G1F, 1G1G, 1G1H, 1G1I, 1G1J, 1G1K, 1G1L, 1G1M, 1G1O, 1G1Q, 1G1R, 1G1S, 1G1T, 1G1U, 1G1V, 1G1W, 1G1Y, 1G20, 1G21, 1G23, 1G24, 1G27, 1G28, 1G29, 1G2A, 1G2B, 1G2C, 1G2D, 1G2F, 1G2I, 1G2K, 1G2L, 1G2M, 1G2N, 1G2O, 1G2P, 1G2Q, 1G2R, 1G2U, 1G2V, 1G2W, 1G2X, 1G2Y, 1G2Z, 1G30, 1G31, 1G32, 1G33, 1G35, 1G36, 1G37, 1G38, 1G39, 1G3B, 1G3C, 1G3D, 1G3E, 1G3I, 1G3J, 1G3K, 1G3L, 1G3M, 1G3N, 1G3O, 1G3P, 1G3Q, 1G3R, 1G3S, 1G3T, 1G3U, 1G3V, 1G3W, 1G3X, 1G3Y, 1G3Z, 1G40, 1G41, 1G42, 1G43, 1G44, 1G45, 1G46, 1G48, 1G49, 1G4A, 1G4B, 1G4C, 1G4E, 1G4H, 1G4I, 1G4J, 1G4K, 1G4M, 1G4O, 1G4P, 1G4R, 1G4S, 1G4T, 1G4U, 1G4V, 1G4W, 1G4X, 1G4Y, 1G50, 1G51, 1G52, 1G53, 1G54, 1G55, 1G57, 1G58, 1G5A, 1G5B, 1G5C, 1G5F, 1G5G, 1G5H, 1G5I, 1G5N, 1G5P, 1G5Q, 1G5R, 1G5S, 1G5T, 1G5U, 1G5X, 1G5Y, 1G5Z, 1G60, 1G61, 1G62, 1G63, 1G64, 1G65, 1G66, 1G67, 1G68, 1G69, 1G6A, 1G6B, 1G6C, 1G6D, 1G6G, 1G6H, 1G6I, 1G6K, 1G6L, 1G6N, 1G6O, 1G6Q, 1G6R, 1G6S, 1G6T, 1G6U, 1G6V, 1G6W, 1G6X, 1G6Y, 1G71, 1G72, 1G73, 1G74, 1G75, 1G76, 1G77, 1G78, 1G79, 1G7A, 1G7B, 1G7C, 1G7F, 1G7G, 1G7H, 1G7I, 1G7J, 1G7K, 1G7L, 1G7M, 1G7N, 1G7P, 1G7Q, 1G7R, 1G7S, 1G7T, 1G7U, 1G7V, 1G7W, 1G7X, 1G7Y, 1G81, 1G82, 1G83, 1G85, 1G86, 1G87, 1G88, 1G8A, 1G8E, 1G8F, 1G8G, 1G8H, 1G8I, 1G8J, 1G8K, 1G8L, 1G8M, 1G8N, 1G8O, 1G8P, 1G8Q, 1G8R, 1G8S, 1G8T, 1G8U, 1G8V, 1G8W, 1G8X, 1G8Y, 1G8Z, 1G93, 1G94, 1G95, 1G96, 1G97, 1G98, 1G99, 1G9A, 1G9B, 1G9C, 1G9D, 1G9F, 1G9G, 1G9H, 1G9I, 1G9J, 1G9K, 1G9M, 1G9N, 1G9O, 1G9Q, 1G9R, 1G9S, 1G9T, 1G9U, 1G9V, 1G9W, 1G9X, 1G9Y, 1G9Z, 1GA0, 1GA1, 1GA2, 1GA4, 1GA5, 1GA6, 1GA7, 1GA8, 1GA9, 1GAD, 1GAE, 1GAF, 1GAG, 1GAH, 1GAI, 1GAJ, 1GAK, 1GAL, 1GAM, 1GAN, 1GAO, 1GAQ, 1GAR, 1GAV, 1GAW, 1GAY, 1GAZ, 1GB0, 1GB2, 1GB3, 1GB5, 1GB6, 1GB7, 1GB8, 1GB9, 1GBA, 1GBB, 1GBC, 1GBD, 1GBE, 1GBF, 1GBG, 1GBH, 1GBI, 1GBJ, 1GBK, 1GBL, 1GBM, 1GBN, 1GBO, 1GBS, 1GBT, 1GBU, 1GBV, 1GBW, 1GBX, 1GBY, 1GBZ, 1GC0, 1GC1, 1GC2, 1GC3, 1GC4, 1GC5, 1GC6, 1GC7, 1GC8, 1GC9, 1GCA, 1GCB, 1GCD, 1GCE, 1GCG, 1GCI, 1GCJ, 1GCK, 1GCL, 1GCM, 1GCN, 1GCO, 1GCP, 1GCQ, 1GCS, 1GCT, 1GCU, 1GCV, 1GCW, 1GCY, 1GCZ, 1GD0, 1GD1, 1GD2, 1GD6, 1GD7, 1GD8, 1GD9, 1GDD, 1GDE, 1GDH, 1GDI, 1GDJ, 1GDK, 1GDL, 1GDN, 1GDQ, 1GDR, 1GDT, 1GDU, 1GDV, 1GDW, 1GDX, 1GE0, 1GE1, 1GE2, 1GE3, 1GE4, 1GE5, 1GE6, 1GE7, 1GE8, 1GEB, 1GEC, 1GED, 1GEE, 1GEF, 1GEG, 1GEH, 1GEI, 1GEJ, 1GEK, 1GEM, 1GEN, 1GEQ, 1GER, 1GES, 1GET, 1GEU, 1GEV, 1GEW, 1GEX, 1GEY, 1GEZ, 1GF0, 1GF3, 1GF4, 1GF5, 1GF6, 1GF7, 1GF8, 1GF9, 1GFA, 1GFE, 1GFG, 1GFH, 1GFI, 1GFJ, 1GFK, 1GFL, 1GFM, 1GFN, 1GFO, 1GFP, 1GFQ, 1GFR, 1GFS, 1GFT, 1GFU, 1GFV, 1GFW, 1GFY, 1GFZ, 1GG0, 1GG1, 1GG2, 1GG3, 1GG4, 1GG5, 1GG6, 1GG8, 1GG9, 1GGB, 1GGC, 1GGD, 1GGE, 1GGF, 1GGG, 1GGH, 1GGI, 1GGJ, 1GGK, 1GGL, 1GGM, 1GGN, 1GGO, 1GGP, 1GGQ, 1GGT, 1GGU, 1GGV, 1GGX, 1GGY, 1GGZ, 1GH0, 1GH2, 1GH4, 1GH6, 1GH7, 1GHA, 1GHB, 1GHD, 1GHE, 1GHF, 1GHI, 1GHL, 1GHM, 1GHO, 1GHP, 1GHQ, 1GHR, 1GHS, 1GHV, 1GHW, 1GHX, 1GHY, 1GHZ, 1GI0, 1GI1, 1GI2, 1GI3, 1GI4, 1GI5, 1GI6, 1GI7, 1GI8, 1GI9, 1GIA, 1GIC, 1GIF, 1GIG, 1GIH, 1GII, 1GIJ, 1GIK, 1GIL, 1GIM, 1GIN, 1GIQ, 1GIR, 1GIS, 1GIT, 1GIU, 1GIY, 1GJ4, 1GJ5, 1GJ6, 1GJ7, 1GJ8, 1GJ9, 1GJA, 1GJB, 1GJC, 1GJD, 1GJI, 1GJM, 1GJN, 1GJO, 1GJP, 1GJQ, 1GJR, 1GJU, 1GJV, 1GJW, 1GJY, 1GK0, 1GK1, 1GK2, 1GK3, 1GK4, 1GK6, 1GK7, 1GK8, 1GK9, 1GKA, 1GKB, 1GKC, 1GKD, 1GKE, 1GKF, 1GKH, 1GKI, 1GKJ, 1GKK, 1GKL, 1GKM, 1GKO, 1GKP, 1GKQ, 1GKR, 1GKU, 1GKX, 1GKY, 1GKZ, 1GL0, 1GL1, 1GL2, 1GL3, 1GL4, 1GL6, 1GL7, 1GL9, 1GLA, 1GLB, 1GLC, 1GLD, 1GLE, 1GLF, 1GLG, 1GLH, 1GLI, 1GLJ, 1GLL, 1GLM, 1GLN, 1GLO, 1GLP, 1GLQ, 1GLU, 1GLV, 1GM4, 1GM5, 1GM6, 1GM7, 1GM8, 1GM9, 1GMB, 1GMC, 1GMD, 1GME, 1GMG, 1GMH, 1GMI, 1GMJ, 1GML, 1GMM, 1GMN, 1GMO, 1GMP, 1GMQ, 1GMR, 1GMU, 1GMV, 1GMW, 1GMX, 1GMY, 1GMZ, 1GN0, 1GN1, 1GN2, 1GN3, 1GN4, 1GN6, 1GN8, 1GN9, 1GND, 1GNE, 1GNG, 1GNH, 1GNI, 1GNJ, 1GNK, 1GNL, 1GNM, 1GNN, 1GNO, 1GNP, 1GNQ, 1GNR, 1GNS, 1GNT, 1GNU, 1GNV, 1GNW, 1GNX, 1GNY, 1GNZ, 1GO2, 1GO3, 1GO4, 1GO7, 1GO8, 1GOA, 1GOB, 1GOC, 1GOD, 1GOF, 1GOG, 1GOH, 1GOI, 1GOJ, 1GOK, 1GOL, 1GOM, 1GON, 1GOO, 1GOQ, 1GOR, 1GOS, 1GOT, 1GOU, 1GOV, 1GOW, 1GOX, 1GOY, 1GOZ, 1GP0, 1GP1, 1GP2, 1GP3, 1GP4, 1GP5, 1GP6, 1GP7, 1GP9, 1GPA, 1GPB, 1GPC, 1GPD, 1GPE, 1GPF, 1GPH, 1GPI, 1GPJ, 1GPK, 1GPL, 1GPM, 1GPN, 1GPO, 1GPP, 1GPQ, 1GPR, 1GPU, 1GPW, 1GPY, 1GPZ, 1GQ1, 1GQ2, 1GQ3, 1GQ4, 1GQ5, 1GQ6, 1GQ7, 1GQ8, 1GQ9, 1GQA, 1GQB, 1GQC, 1GQE, 1GQF, 1GQG, 1GQH, 1GQI, 1GQJ, 1GQK, 1GQL, 1GQM, 1GQN, 1GQO, 1GQP, 1GQQ, 1GQR, 1GQS, 1GQT, 1GQU, 1GQV, 1GQW, 1GQY, 1GQZ, 1GR0, 1GR1, 1GR2, 1GR3, 1GR7, 1GRA, 1GRB, 1GRC, 1GRE, 1GRF, 1GRG, 1GRH, 1GRI, 1GRJ, 1GRL, 1GRN, 1GRO, 1GRP, 1GRQ, 1GRR, 1GRT, 1GRV, 1GRW, 1GS0, 1GS3, 1GS4, 1GS5, 1GS6, 1GS7, 1GS8, 1GS9, 1GSA, 1GSB, 1GSC, 1GSD, 1GSE, 1GSF, 1GSG, 1GSH, 1GSI, 1GSJ, 1GSK, 1GSL, 1GSM, 1GSN, 1GSO, 1GSP, 1GSQ, 1GSS, 1GSU, 1GSV, 1GSW, 1GSX, 1GSY, 1GSZ, 1GT0, 1GT1, 1GT3, 1GT4, 1GT5, 1GT6, 1GT7, 1GT8, 1GT9, 1GTA, 1GTB, 1GTD, 1GTE, 1GTF, 1GTG, 1GTH, 1GTI, 1GTJ, 1GTK, 1GTL, 1GTM, 1GTN, 1GTO, 1GTP, 1GTQ, 1GTT, 1GTU, 1GTV, 1GTW, 1GTZ, 1GU0, 1GU1, 1GU2, 1GU3, 1GU4, 1GU5, 1GU6, 1GU7, 1GU8, 1GU9, 1GUA, 1GUB, 1GUD, 1GUE, 1GUF, 1GUG, 1GUH, 1GUI, 1GUJ, 1GUK, 1GUL, 1GUM, 1GUN, 1GUO, 1GUP, 1GUQ, 1GUS, 1GUT, 1GUU, 1GUV, 1GUX, 1GUY, 1GUZ, 1GV0, 1GV1, 1GV2, 1GV3, 1GV4, 1GV5, 1GV7, 1GV8, 1GV9, 1GVC, 1GVD, 1GVE, 1GVF, 1GVG, 1GVH, 1GVI, 1GVJ, 1GVK, 1GVL, 1GVM, 1GVN, 1GVO, 1GVP, 1GVQ, 1GVR, 1GVS, 1GVT, 1GVU, 1GVV, 1GVW, 1GVX, 1GVY, 1GVZ, 1GW0, 1GW1, 1GW2, 1GW6, 1GW9, 1GWA, 1GWB, 1GWC, 1GWD, 1GWE, 1GWF, 1GWG, 1GWH, 1GWI, 1GWJ, 1GWK, 1GWL, 1GWM, 1GWN, 1GWO, 1GWQ, 1GWR, 1GWS, 1GWT, 1GWU, 1GWV, 1GWW, 1GWX, 1GWY, 1GWZ, 1GX0, 1GX1, 1GX2, 1GX3, 1GX4, 1GX5, 1GX6, 1GX8, 1GX9, 1GXA, 1GXB, 1GXC, 1GXD, 1GXF, 1GXJ, 1GXK, 1GXL, 1GXM, 1GXN, 1GXO, 1GXP, 1GXQ, 1GXR, 1GXS, 1GXT, 1GXU, 1GXW, 1GXY, 1GXZ, 1GY0, 1GY1, 1GY2, 1GY3, 1GY5, 1GY6, 1GY7, 1GY8, 1GY9, 1GYB, 1GYC, 1GYD, 1GYE, 1GYG, 1GYH, 1GYJ, 1GYK, 1GYL, 1GYM, 1GYN, 1GYO, 1GYP, 1GYQ, 1GYR, 1GYT, 1GYU, 1GYV, 1GYW, 1GYX, 1GYY, 1GZ0, 1GZ1, 1GZ2, 1GZ3, 1GZ4, 1GZ5, 1GZ6, 1GZ7, 1GZ8, 1GZ9, 1GZA, 1GZB, 1GZC, 1GZD, 1GZE, 1GZF, 1GZG, 1GZH, 1GZI, 1GZJ, 1GZK, 1GZL, 1GZM, 1GZN, 1GZO, 1GZP, 1GZQ, 1GZR, 1GZS, 1GZT, 1GZU, 1GZV, 1GZW, 1GZX, 1GZY, 1GZZ, 1H00, 1H01, 1H02, 1H03, 1H04, 1H05, 1H07, 1H08, 1H09, 1H0A, 1H0B, 1H0C, 1H0D, 1H0G, 1H0H, 1H0I, 1H0J, 1H0K, 1H0M, 1H0N, 1H0O, 1H0P, 1H0R, 1H0S, 1H0V, 1H0W, 1H0X, 1H0Y, 1H10, 1H11, 1H12, 1H13, 1H14, 1H15, 1H16, 1H17, 1H18, 1H19, 1H1A, 1H1B, 1H1C, 1H1D, 1H1H, 1H1I, 1H1L, 1H1M, 1H1N, 1H1O, 1H1P, 1H1Q, 1H1R, 1H1S, 1H1T, 1H1V, 1H1W, 1H1X, 1H1Y, 1H1Z, 1H21, 1H22, 1H23, 1H24, 1H25, 1H26, 1H27, 1H28, 1H29, 1H2A, 1H2B, 1H2E, 1H2F, 1H2G, 1H2H, 1H2I, 1H2J, 1H2K, 1H2L, 1H2M, 1H2N, 1H2P, 1H2Q, 1H2R, 1H2S, 1H2T, 1H2U, 1H2V, 1H2W, 1H2X, 1H2Y, 1H2Z, 1H30, 1H31, 1H32, 1H33, 1H34, 1H35, 1H36, 1H37, 1H39, 1H3A, 1H3B, 1H3C, 1H3D, 1H3F, 1H3G, 1H3I, 1H3J, 1H3L, 1H3M, 1H3N, 1H3O, 1H3P, 1H3Q, 1H3T, 1H3U, 1H3V, 1H3W, 1H3X, 1H3Y, 1H41, 1H42, 1H43, 1H44, 1H45, 1H46, 1H47, 1H48, 1H49, 1H4A, 1H4C, 1H4D, 1H4E, 1H4F, 1H4G, 1H4H, 1H4I, 1H4J, 1H4K, 1H4L, 1H4M, 1H4N, 1H4O, 1H4P, 1H4R, 1H4T, 1H4U, 1H4V, 1H4W, 1H4X, 1H4Y, 1H4Z, 1H50, 1H51, 1H52, 1H53, 1H54, 1H55, 1H56, 1H57, 1H58, 1H59, 1H5A, 1H5B, 1H5C, 1H5D, 1H5E, 1H5F, 1H5G, 1H5H, 1H5I, 1H5J, 1H5K, 1H5L, 1H5M, 1H5N, 1H5Q, 1H5R, 1H5S, 1H5T, 1H5U, 1H5V, 1H5W, 1H5X, 1H5Y, 1H5Z, 1H60, 1H61, 1H62, 1H63, 1H64, 1H65, 1H66, 1H68, 1H69, 1H6A, 1H6B, 1H6C, 1H6D, 1H6E, 1H6F, 1H6G, 1H6H, 1H6J, 1H6K, 1H6L, 1H6M, 1H6N, 1H6O, 1H6P, 1H6R, 1H6S, 1H6T, 1H6U, 1H6V, 1H6W, 1H6X, 1H6Y, 1H70, 1H71, 1H72, 1H73, 1H74, 1H75, 1H76, 1H78, 1H79, 1H7A, 1H7B, 1H7C, 1H7E, 1H7F, 1H7G, 1H7H, 1H7I, 1H7K, 1H7L, 1H7M, 1H7N, 1H7O, 1H7P, 1H7Q, 1H7R, 1H7S, 1H7T, 1H7U, 1H7W, 1H7X, 1H7Z, 1H80, 1H81, 1H82, 1H83, 1H84, 1H85, 1H86, 1H87, 1H88, 1H89, 1H8A, 1H8D, 1H8E, 1H8F, 1H8G, 1H8H, 1H8I, 1H8K, 1H8L, 1H8N, 1H8O, 1H8P, 1H8S, 1H8U, 1H8V, 1H8X, 1H8Y, 1H8Z, 1H91, 1H93, 1H94, 1H96, 1H97, 1H98, 1H99, 1H9A, 1H9B, 1H9D, 1H9G, 1H9H, 1H9I, 1H9J, 1H9K, 1H9L, 1H9M, 1H9N, 1H9O, 1H9P, 1H9Q, 1H9R, 1H9S, 1H9T, 1H9U, 1H9V, 1H9W, 1H9X, 1H9Y, 1H9Z, 1HA0, 1HA1, 1HA2, 1HA3, 1HA4, 1HA5, 1HA7, 1HAB, 1HAC, 1HAG, 1HAH, 1HAI, 1HAK, 1HAN, 1HAO, 1HAP, 1HAR, 1HAU, 1HAV, 1HAW, 1HAX, 1HAY, 1HAZ, 1HB0, 1HB1, 1HB2, 1HB3, 1HB4, 1HB6, 1HB8, 1HBA, 1HBB, 1HBG, 1HBH, 1HBI, 1HBJ, 1HBK, 1HBM, 1HBN, 1HBO, 1HBP, 1HBQ, 1HBR, 1HBS, 1HBT, 1HBU, 1HBV, 1HBX, 1HBY, 1HBZ, 1HC0, 1HC1, 1HC7, 1HC9, 1HCA, 1HCB, 1HCF, 1HCG, 1HCH, 1HCI, 1HCJ, 1HCK, 1HCL, 1HCM, 1HCN, 1HCO, 1HCQ, 1HCR, 1HCU, 1HCV, 1HCX, 1HCY, 1HCZ, 1HD2, 1HD3, 1HD5, 1HD7, 1HD8, 1HDA, 1HDB, 1HDC, 1HDD, 1HDE, 1HDF, 1HDG, 1HDH, 1HDI, 1HDK, 1HDM, 1HDO, 1HDQ, 1HDR, 1HDS, 1HDT, 1HDU, 1HDX, 1HDY, 1HDZ, 1HE1, 1HE2, 1HE3, 1HE4, 1HE5, 1HE7, 1HE8, 1HE9, 1HEA, 1HEB, 1HEC, 1HED, 1HEE, 1HEF, 1HEG, 1HEI, 1HEK, 1HEL, 1HEM, 1HEN, 1HEO, 1HEP, 1HEQ, 1HER, 1HES, 1HET, 1HEU, 1HEW, 1HEX, 1HEY, 1HEZ, 1HF0, 1HF2, 1HF3, 1HF4, 1HF6, 1HF8, 1HFA, 1HFB, 1HFC, 1HFD, 1HFE, 1HFJ, 1HFK, 1HFO, 1HFP, 1HFQ, 1HFR, 1HFS, 1HFU, 1HFW, 1HFX, 1HFY, 1HFZ, 1HG0, 1HG1, 1HG2, 1HG3, 1HG4, 1HG5, 1HG7, 1HG8, 1HGA, 1HGB, 1HGC, 1HGD, 1HGE, 1HGF, 1HGG, 1HGH, 1HGI, 1HGJ, 1HGT, 1HGU, 1HGW, 1HGX, 1HGY, 1HH1, 1HH2, 1HH4, 1HH5, 1HH6, 1HH7, 1HH8, 1HH9, 1HHG, 1HHH, 1HHI, 1HHJ, 1HHK, 1HHL, 1HHO, 1HHP, 1HHQ, 1HHS, 1HHT, 1HI0, 1HI1, 1HI2, 1HI3, 1HI4, 1HI5, 1HI6, 1HI8, 1HI9, 1HIA, 1HIB, 1HIG, 1HIH, 1HII, 1HIJ, 1HIK, 1HIL, 1HIM, 1HIN, 1HIO, 1HIP, 1HIV, 1HIW, 1HIX, 1HIY, 1HIZ, 1HJ1, 1HJ3, 1HJ4, 1HJ5, 1HJ6, 1HJ8, 1HJ9, 1HJA, 1HJB, 1HJC, 1HJE, 1HJF, 1HJG, 1HJJ, 1HJK, 1HJL, 1HJO, 1HJP, 1HJQ, 1HJR, 1HJS, 1HJT, 1HJU, 1HJV, 1HJW, 1HJX, 1HJZ, 1HK0, 1HK1, 1HK2, 1HK3, 1HK4, 1HK5, 1HK7, 1HK8, 1HK9, 1HKA, 1HKB, 1HKC, 1HKD, 1HKF, 1HKG, 1HKH, 1HKI, 1HKJ, 1HKK, 1HKL, 1HKM, 1HKN, 1HKQ, 1HKU, 1HKV, 1HKW, 1HKX, 1HL2, 1HL3, 1HL4, 1HL5, 1HL6, 1HL7, 1HL8, 1HL9, 1HLA, 1HLB, 1HLC, 1HLD, 1HLE, 1HLF, 1HLG, 1HLK, 1HLM, 1HLO, 1HLP, 1HLQ, 1HLT, 1HLU, 1HLV, 1HLW, 1HLZ, 1HM0, 1HM2, 1HM3, 1HM4, 1HM5, 1HM6, 1HM7, 1HM8, 1HM9, 1HMC, 1HMD, 1HMK, 1HML, 1HMO, 1HMP, 1HMR, 1HMS, 1HMT, 1HMU, 1HMV, 1HMW, 1HMY, 1HN0, 1HN1, 1HN2, 1HN4, 1HN9, 1HNA, 1HNB, 1HNC, 1HND, 1HNE, 1HNF, 1HNG, 1HNH, 1HNI, 1HNJ, 1HNK, 1HNL, 1HNN, 1HNO, 1HNU, 1HNV, 1HNY, 1HO1, 1HO3, 1HO4, 1HO5, 1HO8, 1HOC, 1HOE, 1HON, 1HOO, 1HOP, 1HOR, 1HOS, 1HOT, 1HOW, 1HOX, 1HOZ, 1HP0, 1HP1, 1HP4, 1HP5, 1HP7, 1HPB, 1HPC, 1HPG, 1HPI, 1HPL, 1HPM, 1HPO, 1HPS, 1HPT, 1HPU, 1HPV, 1HPX, 1HPZ, 1HQ0, 1HQ2, 1HQ3, 1HQ4, 1HQ5, 1HQ6, 1HQ7, 1HQ8, 1HQA, 1HQC, 1HQD, 1HQE, 1HQF, 1HQG, 1HQH, 1HQJ, 1HQK, 1HQL, 1HQM, 1HQN, 1HQO, 1HQP, 1HQQ, 1HQR, 1HQS, 1HQT, 1HQU, 1HQV, 1HQW, 1HQX, 1HQY, 1HQZ, 1HR3, 1HR6, 1HR7, 1HR8, 1HR9, 1HRB, 1HRC, 1HRD, 1HRH, 1HRK, 1HRM, 1HRN, 1HRO, 1HRP, 1HRS, 1HRT, 1HRU, 1HS6, 1HSA, 1HSB, 1HSE, 1HSG, 1HSH, 1HSI, 1HSJ, 1HSK, 1HSL, 1HSO, 1HSR, 1HSS, 1HST, 1HSW, 1HSX, 1HSY, 1HSZ, 1HT0, 1HT1, 1HT2, 1HT3, 1HT5, 1HT6, 1HT8, 1HT9, 1HTA, 1HTB, 1HTD, 1HTE, 1HTF, 1HTG, 1HTI, 1HTJ, 1HTL, 1HTM, 1HTN, 1HTO, 1HTP, 1HTQ, 1HTR, 1HTT, 1HTV, 1HTW, 1HTY, 1HTZ, 1HU0, 1HU3, 1HU8, 1HU9, 1HUC, 1HUF, 1HUG, 1HUH, 1HUJ, 1HUK, 1HUL, 1HUO, 1HUP, 1HUQ, 1HUR, 1HUS, 1HUT, 1HUU, 1HUV, 1HUW, 1HUX, 1HUY, 1HUZ, 1HV0, 1HV1, 1HV4, 1HV5, 1HV6, 1HV7, 1HV8, 1HV9, 1HVA, 1HVB, 1HVC, 1HVD, 1HVE, 1HVF, 1HVG, 1HVH, 1HVI, 1HVJ, 1HVK, 1HVL, 1HVQ, 1HVR, 1HVS, 1HVV, 1HVX, 1HVY, 1HW1, 1HW2, 1HW3, 1HW4, 1HW5, 1HW6, 1HW7, 1HW8, 1HW9, 1HWG, 1HWH, 1HWI, 1HWJ, 1HWK, 1HWL, 1HWM, 1HWN, 1HWO, 1HWP, 1HWR, 1HWT, 1HWU, 1HWW, 1HWY, 1HWZ, 1HX0, 1HX1, 1HX3, 1HX5, 1HX6, 1HX8, 1HX9, 1HXA, 1HXB, 1HXC, 1HXD, 1HXE, 1HXF, 1HXG, 1HXH, 1HXI, 1HXJ, 1HXK, 1HXL, 1HXM, 1HXN, 1HXP, 1HXQ, 1HXR, 1HXT, 1HXU, 1HXW, 1HXX, 1HXY, 1HXZ, 1HY0, 1HY1, 1HY2, 1HY3, 1HY5, 1HY7, 1HYB, 1HYE, 1HYF, 1HYG, 1HYH, 1HYL, 1HYN, 1HYO, 1HYP, 1HYQ, 1HYR, 1HYT, 1HYU, 1HYV, 1HYZ, 1HZ1, 1HZ4, 1HZ5, 1HZ6, 1HZ9, 1HZA, 1HZB, 1HZC, 1HZD, 1HZF, 1HZG, 1HZH, 1HZI, 1HZJ, 1HZO, 1HZP, 1HZT, 1HZU, 1HZV, 1HZW, 1HZX, 1HZY, 1HZZ, 1I00, 1I01, 1I04, 1I05, 1I06, 1I07, 1I08, 1I09, 1I0A, 1I0B, 1I0C, 1I0D, 1I0E, 1I0F, 1I0G, 1I0H, 1I0I, 1I0J, 1I0K, 1I0L, 1I0M, 1I0N, 1I0O, 1I0P, 1I0Q, 1I0R, 1I0S, 1I0T, 1I0V, 1I0X, 1I0Z, 1I10, 1I12, 1I13, 1I14, 1I19, 1I1A, 1I1B, 1I1C, 1I1D, 1I1E, 1I1F, 1I1G, 1I1H, 1I1I, 1I1J, 1I1K, 1I1L, 1I1M, 1I1N, 1I1O, 1I1P, 1I1Q, 1I1R, 1I1W, 1I1X, 1I1Y, 1I1Z, 1I20, 1I21, 1I22, 1I24, 1I27, 1I29, 1I2A, 1I2B, 1I2C, 1I2D, 1I2E, 1I2F, 1I2G, 1I2H, 1I2K, 1I2L, 1I2M, 1I2N, 1I2O, 1I2P, 1I2Q, 1I2R, 1I2S, 1I2T, 1I2W, 1I2Z, 1I30, 1I31, 1I32, 1I33, 1I36, 1I37, 1I38, 1I39, 1I3A, 1I3C, 1I3D, 1I3E, 1I3F, 1I3G, 1I3H, 1I3I, 1I3J, 1I3K, 1I3L, 1I3M, 1I3N, 1I3O, 1I3P, 1I3Q, 1I3R, 1I3S, 1I3T, 1I3U, 1I3V, 1I3Z, 1I40, 1I41, 1I43, 1I44, 1I45, 1I47, 1I48, 1I49, 1I4A, 1I4D, 1I4E, 1I4F, 1I4G, 1I4H, 1I4J, 1I4K, 1I4L, 1I4M, 1I4N, 1I4O, 1I4P, 1I4Q, 1I4R, 1I4S, 1I4T, 1I4U, 1I4W, 1I4X, 1I4Y, 1I4Z, 1I50, 1I51, 1I52, 1I53, 1I54, 1I55, 1I57, 1I58, 1I59, 1I5A, 1I5B, 1I5C, 1I5D, 1I5E, 1I5F, 1I5G, 1I5I, 1I5K, 1I5N, 1I5O, 1I5P, 1I5Q, 1I5R, 1I5S, 1I5W, 1I5X, 1I5Y, 1I5Z, 1I60, 1I69, 1I6A, 1I6B, 1I6I, 1I6J, 1I6K, 1I6L, 1I6M, 1I6N, 1I6O, 1I6P, 1I6Q, 1I6S, 1I6T, 1I6V, 1I6W, 1I6X, 1I70, 1I71, 1I72, 1I73, 1I74, 1I75, 1I76, 1I77, 1I78, 1I79, 1I7A, 1I7B, 1I7C, 1I7D, 1I7E, 1I7F, 1I7G, 1I7H, 1I7I, 1I7K, 1I7L, 1I7M, 1I7N, 1I7O, 1I7P, 1I7Q, 1I7R, 1I7S, 1I7T, 1I7U, 1I7W, 1I7X, 1I7Y, 1I7Z, 1I80, 1I81, 1I82, 1I83, 1I85, 1I86, 1I88, 1I89, 1I8A, 1I8B, 1I8D, 1I8F, 1I8I, 1I8J, 1I8K, 1I8L, 1I8M, 1I8N, 1I8O, 1I8P, 1I8Q, 1I8T, 1I8U, 1I8V, 1I8Z, 1I90, 1I91, 1I92, 1I9A, 1I9B, 1I9C, 1I9D, 1I9E, 1I9G, 1I9H, 1I9I, 1I9J, 1I9L, 1I9M, 1I9N, 1I9O, 1I9P, 1I9Q, 1I9R, 1I9S, 1I9T, 1I9W, 1I9Y, 1I9Z, 1IA1, 1IA2, 1IA3, 1IA4, 1IA5, 1IA6, 1IA7, 1IA8, 1IA9, 1IAA, 1IAB, 1IAC, 1IAD, 1IAE, 1IAG, 1IAH, 1IAI, 1IAJ, 1IAK, 1IAL, 1IAM, 1IAN, 1IAO, 1IAP, 1IAQ, 1IAR, 1IAS, 1IAT, 1IAU, 1IAV, 1IAW, 1IAX, 1IAY, 1IAZ, 1IB0, 1IB1, 1IB2, 1IB4, 1IB5, 1IB6, 1IBB, 1IBC, 1IBD, 1IBE, 1IBF, 1IBG, 1IBH, 1IBJ, 1IBQ, 1IBR, 1IBS, 1IBT, 1IBU, 1IBV, 1IBW, 1IBY, 1IBZ, 1IC0, 1IC1, 1IC2, 1IC4, 1IC5, 1IC6, 1IC7, 1IC8, 1ICC, 1ICE, 1ICF, 1ICI, 1ICJ, 1ICK, 1ICM, 1ICN, 1ICP, 1ICQ, 1ICR, 1ICS, 1ICT, 1ICU, 1ICV, 1ICW, 1ICX, 1ID0, 1ID1, 1ID2, 1ID3, 1ID4, 1ID5, 1IDA, 1IDB, 1IDC, 1IDD, 1IDE, 1IDF, 1IDJ, 1IDK, 1IDM, 1IDN, 1IDO, 1IDP, 1IDQ, 1IDR, 1IDS, 1IDT, 1IDU, 1IE0, 1IE3, 1IE4, 1IE7, 1IE8, 1IE9, 1IEA, 1IEB, 1IEC, 1IED, 1IEE, 1IEF, 1IEG, 1IEI, 1IEJ, 1IEL, 1IEM, 1IEP, 1IEQ, 1IER, 1IES, 1IEV, 1IEW, 1IEX, 1IF1, 1IF2, 1IF4, 1IF5, 1IF6, 1IF7, 1IF8, 1IF9, 1IFA, 1IFB, 1IFC, 1IFG, 1IFH, 1IFQ, 1IFR, 1IFS, 1IFT, 1IFU, 1IFV, 1IFX, 1IG0, 1IG1, 1IG3, 1IG5, 1IG7, 1IG8, 1IG9, 1IGB, 1IGC, 1IGD, 1IGF, 1IGI, 1IGJ, 1IGM, 1IGN, 1IGO, 1IGP, 1IGQ, 1IGR, 1IGS, 1IGT, 1IGU, 1IGV, 1IGW, 1IGX, 1IGY, 1IGZ, 1IH1, 1IH4, 1IH6, 1IH7, 1IH8, 1IHB, 1IHC, 1IHD, 1IHF, 1IHG, 1IHH, 1IHI, 1IHJ, 1IHK, 1IHN, 1IHO, 1IHP, 1IHR, 1IHS, 1IHT, 1IHU, 1IHX, 1IHY, 1IHZ, 1II0, 1II2, 1II3, 1II4, 1II5, 1II6, 1II7, 1II8, 1II9, 1IIB, 1IIC, 1IID, 1IIG, 1IIH, 1III, 1IIK, 1IIL, 1IIM, 1IIN, 1IIP, 1IIQ, 1IIR, 1IIT, 1IIU, 1IIW, 1IIZ, 1IJ0, 1IJ1, 1IJ2, 1IJ3, 1IJ5, 1IJ6, 1IJ8, 1IJ9, 1IJB, 1IJE, 1IJF, 1IJG, 1IJH, 1IJI, 1IJJ, 1IJK, 1IJL, 1IJN, 1IJQ, 1IJR, 1IJT, 1IJU, 1IJV, 1IJW, 1IJX, 1IJY, 1IK3, 1IK4, 1IK6, 1IK7, 1IK9, 1IKA, 1IKE, 1IKF, 1IKG, 1IKI, 1IKJ, 1IKK, 1IKN, 1IKO, 1IKP, 1IKQ, 1IKT, 1IKV, 1IKW, 1IKX, 1IKY, 1IL0, 1IL1, 1IL3, 1IL4, 1IL5, 1IL9, 1ILC, 1ILD, 1ILE, 1ILG, 1ILH, 1ILK, 1ILR, 1ILS, 1ILT, 1ILU, 1ILV, 1ILW, 1ILZ, 1IM0, 1IM2, 1IM3, 1IM4, 1IM5, 1IM6, 1IM8, 1IM9, 1IMA, 1IMB, 1IMC, 1IMD, 1IME, 1IMF, 1IMH, 1IMJ, 1IMV, 1IMX, 1IN0, 1IN4, 1IN5, 1IN6, 1IN7, 1IN8, 1INC, 1IND, 1INE, 1INF, 1ING, 1INH, 1INI, 1INJ, 1INL, 1INN, 1INO, 1INP, 1INQ, 1INR, 1INU, 1INV, 1INW, 1INX, 1INY, 1IO0, 1IO1, 1IO2, 1IO3, 1IO4, 1IO7, 1IO8, 1IO9, 1IOA, 1IOB, 1IOC, 1IOD, 1IOE, 1IOF, 1IOI, 1IOK, 1IOL, 1IOM, 1ION, 1IOO, 1IOP, 1IOQ, 1IOR, 1IOS, 1IOT, 1IOV, 1IOW, 1IOZ, 1IP1, 1IP2, 1IP3, 1IP4, 1IP5, 1IP6, 1IP7, 1IPA, 1IPB, 1IPC, 1IPD, 1IPE, 1IPF, 1IPH, 1IPI, 1IPJ, 1IPK, 1IPP, 1IPS, 1IPW, 1IQ0, 1IQ1, 1IQ4, 1IQ5, 1IQ6, 1IQ7, 1IQ8, 1IQ9, 1IQA, 1IQB, 1IQC, 1IQD, 1IQE, 1IQF, 1IQG, 1IQH, 1IQI, 1IQJ, 1IQK, 1IQL, 1IQM, 1IQN, 1IQP, 1IQQ, 1IQR, 1IQU, 1IQV, 1IQW, 1IQX, 1IQY, 1IQZ, 1IR0, 1IR1, 1IR2, 1IR3, 1IR6, 1IR7, 1IR8, 1IR9, 1IRA, 1IRB, 1IRC, 1IRD, 1IRE, 1IRI, 1IRJ, 1IRK, 1IRM, 1IRN, 1IRO, 1IRQ, 1IRU, 1IRV, 1IRW, 1IRX, 1IS0, 1IS1, 1IS2, 1IS3, 1IS4, 1IS5, 1IS6, 1IS7, 1IS8, 1IS9, 1ISA, 1ISB, 1ISC, 1ISE, 1ISF, 1ISG, 1ISH, 1ISI, 1ISJ, 1ISM, 1ISN, 1ISO, 1ISP, 1ISQ, 1ISR, 1ISS, 1IST, 1ISU, 1ISV, 1ISW, 1ISX, 1ISY, 1ISZ, 1IT0, 1IT2, 1IT3, 1IT6, 1IT7, 1IT8, 1IT9, 1ITB, 1ITC, 1ITG, 1ITH, 1ITK, 1ITO, 1ITQ, 1ITT, 1ITU, 1ITV, 1ITW, 1ITX, 1ITZ, 1IU1, 1IU3, 1IU4, 1IU5, 1IU7, 1IU8, 1IU9, 1IUA, 1IUB, 1IUC, 1IUD, 1IUE, 1IUG, 1IUH, 1IUJ, 1IUK, 1IUL, 1IUN, 1IUO, 1IUP, 1IUQ, 1IUS, 1IUT, 1IUU, 1IUV, 1IUW, 1IUX, 1IUZ, 1IV1, 1IV2, 1IV3, 1IV4, 1IV5, 1IV7, 1IV8, 1IV9, 1IVB, 1IVC, 1IVD, 1IVE, 1IVF, 1IVG, 1IVH, 1IVI, 1IVJ, 1IVL, 1IVN, 1IVO, 1IVP, 1IVQ, 1IVR, 1IVU, 1IVV, 1IVW, 1IVX, 1IVY, 1IW0, 1IW1, 1IW2, 1IW6, 1IW7, 1IW8, 1IW9, 1IWA, 1IWB, 1IWD, 1IWE, 1IWG, 1IWH, 1IWI, 1IWJ, 1IWK, 1IWL, 1IWM, 1IWN, 1IWO, 1IWP, 1IWQ, 1IWT, 1IWU, 1IWV, 1IWW, 1IWX, 1IWY, 1IWZ, 1IX0, 1IX1, 1IX2, 1IX3, 1IX4, 1IX6, 1IX7, 1IX8, 1IX9, 1IXB, 1IXC, 1IXE, 1IXF, 1IXG, 1IXH, 1IXI, 1IXJ, 1IXK, 1IXL, 1IXM, 1IXN, 1IXO, 1IXP, 1IXQ, 1IXR, 1IXS, 1IXV, 1IXX, 1IXY, 1IXZ, 1IY0, 1IY1, 1IY2, 1IY7, 1IY8, 1IY9, 1IYB, 1IYD, 1IYE, 1IYH, 1IYI, 1IYJ, 1IYK, 1IYL, 1IYN, 1IYO, 1IYP, 1IYQ, 1IYS, 1IYW, 1IYX, 1IYZ, 1IZ0, 1IZ1, 1IZ2, 1IZ3, 1IZ4, 1IZ5, 1IZ6, 1IZ7, 1IZ8, 1IZ9, 1IZA, 1IZB, 1IZC, 1IZD, 1IZE, 1IZH, 1IZI, 1IZJ, 1IZK, 1IZL, 1IZM, 1IZN, 1IZO, 1IZP, 1IZQ, 1IZR, 1IZY, 1IZZ, 1J00, 1J01, 1J02, 1J04, 1J05, 1J06, 1J07, 1J08, 1J09, 1J0A, 1J0B, 1J0C, 1J0D, 1J0E, 1J0H, 1J0I, 1J0J, 1J0K, 1J0M, 1J0N, 1J0O, 1J0R, 1J0W, 1J0X, 1J0Y, 1J0Z, 1J10, 1J11, 1J12, 1J14, 1J15, 1J16, 1J17, 1J18, 1J19, 1J1A, 1J1B, 1J1C, 1J1D, 1J1E, 1J1F, 1J1G, 1J1I, 1J1J, 1J1L, 1J1M, 1J1N, 1J1O, 1J1P, 1J1Q, 1J1R, 1J1S, 1J1T, 1J1V, 1J1W, 1J1X, 1J1Y, 1J1Z, 1J20, 1J21, 1J22, 1J23, 1J24, 1J25, 1J27, 1J2A, 1J2C, 1J2E, 1J2F, 1J2G, 1J2J, 1J2L, 1J2P, 1J2Q, 1J2R, 1J2T, 1J2U, 1J2V, 1J2W, 1J2X, 1J2Y, 1J2Z, 1J30, 1J31, 1J32, 1J33, 1J34, 1J35, 1J36, 1J37, 1J38, 1J39, 1J3A, 1J3B, 1J3E, 1J3F, 1J3H, 1J3I, 1J3J, 1J3K, 1J3L, 1J3M, 1J3N, 1J3P, 1J3Q, 1J3R, 1J3U, 1J3W, 1J3Y, 1J3Z, 1J40, 1J41, 1J42, 1J48, 1J49, 1J4A, 1J4B, 1J4E, 1J4G, 1J4H, 1J4I, 1J4J, 1J4N, 1J4R, 1J4S, 1J4T, 1J4U, 1J4X, 1J4Z, 1J51, 1J52, 1J53, 1J54, 1J55, 1J58, 1J59, 1J5O, 1J5P, 1J5Q, 1J5S, 1J5T, 1J5U, 1J5W, 1J5X, 1J5Y, 1J6O, 1J6P, 1J6R, 1J6U, 1J6V, 1J6W, 1J6X, 1J6Z, 1J70, 1J71, 1J72, 1J73, 1J74, 1J75, 1J77, 1J78, 1J79, 1J7A, 1J7B, 1J7C, 1J7D, 1J7E, 1J7G, 1J7I, 1J7J, 1J7K, 1J7L, 1J7N, 1J7S, 1J7U, 1J7V, 1J7W, 1J7X, 1J7Y, 1J7Z, 1J80, 1J81, 1J82, 1J83, 1J84, 1J85, 1J86, 1J87, 1J88, 1J89, 1J8A, 1J8B, 1J8D, 1J8E, 1J8F, 1J8H, 1J8L, 1J8M, 1J8Q, 1J8R, 1J8S, 1J8T, 1J8U, 1J8V, 1J8Y, 1J90, 1J91, 1J93, 1J95, 1J96, 1J97, 1J98, 1J99, 1J9A, 1J9B, 1J9C, 1J9E, 1J9G, 1J9J, 1J9K, 1J9L, 1J9M, 1J9Q, 1J9R, 1J9S, 1J9T, 1J9W, 1J9Y, 1J9Z, 1JA0, 1JA1, 1JA3, 1JA8, 1JA9, 1JAB, 1JAC, 1JAD, 1JAE, 1JAF, 1JAH, 1JAI, 1JAK, 1JAL, 1JAM, 1JAN, 1JAO, 1JAP, 1JAQ, 1JAT, 1JAW, 1JAX, 1JAY, 1JAZ, 1JB1, 1JB2, 1JB3, 1JB4, 1JB5, 1JB6, 1JB7, 1JB9, 1JBB, 1JBC, 1JBE, 1JBG, 1JBK, 1JBM, 1JBO, 1JBP, 1JBQ, 1JBU, 1JBV, 1JBW, 1JBY, 1JBZ, 1JC0, 1JC1, 1JC4, 1JC5, 1JC7, 1JC9, 1JCA, 1JCC, 1JCD, 1JCE, 1JCF, 1JCG, 1JCH, 1JCI, 1JCJ, 1JCK, 1JCL, 1JCM, 1JCN, 1JCQ, 1JCR, 1JCS, 1JCT, 1JCV, 1JCX, 1JCY, 1JCZ, 1JD0, 1JD1, 1JD2, 1JD3, 1JD4, 1JD5, 1JD6, 1JD7, 1JD9, 1JDA, 1JDB, 1JDC, 1JDD, 1JDE, 1JDF, 1JDH, 1JDI, 1JDJ, 1JDL, 1JDN, 1JDO, 1JDP, 1JDR, 1JDS, 1JDT, 1JDU, 1JDV, 1JDW, 1JDX, 1JDY, 1JDZ, 1JE0, 1JE1, 1JE5, 1JE6, 1JE8, 1JEA, 1JEB, 1JEC, 1JED, 1JEE, 1JEF, 1JEH, 1JEJ, 1JEK, 1JEN, 1JEO, 1JEP, 1JEQ, 1JER, 1JES, 1JET, 1JEU, 1JEV, 1JEY, 1JEZ, 1JF0, 1JF1, 1JF2, 1JF3, 1JF4, 1JF5, 1JF6, 1JF7, 1JF8, 1JF9, 1JFA, 1JFB, 1JFC, 1JFD, 1JFG, 1JFH, 1JFI, 1JFL, 1JFM, 1JFQ, 1JFR, 1JFS, 1JFT, 1JFU, 1JFV, 1JFX, 1JFZ, 1JG0, 1JG1, 1JG2, 1JG3, 1JG4, 1JG5, 1JG6, 1JG7, 1JG8, 1JG9, 1JGC, 1JGD, 1JGE, 1JGG, 1JGI, 1JGJ, 1JGL, 1JGM, 1JGR, 1JGS, 1JGT, 1JGU, 1JGV, 1JGW, 1JGX, 1JGY, 1JGZ, 1JH0, 1JH1, 1JH5, 1JH6, 1JH7, 1JH8, 1JH9, 1JHA, 1JHC, 1JHD, 1JHE, 1JHF, 1JHG, 1JHH, 1JHJ, 1JHK, 1JHL, 1JHM, 1JHN, 1JHO, 1JHP, 1JHQ, 1JHR, 1JHS, 1JHT, 1JHU, 1JHV, 1JHW, 1JHX, 1JHY, 1JHZ, 1JI0, 1JI1, 1JI2, 1JI3, 1JI4, 1JI5, 1JI6, 1JI7, 1JIA, 1JIB, 1JIE, 1JIF, 1JIG, 1JIH, 1JII, 1JIJ, 1JIK, 1JIL, 1JIM, 1JIN, 1JIO, 1JIP, 1JIQ, 1JIR, 1JIS, 1JIT, 1JIU, 1JIV, 1JIW, 1JIX, 1JIY, 1JIZ, 1JJ0, 1JJ1, 1JJ3, 1JJ4, 1JJ6, 1JJ7, 1JJ8, 1JJ9, 1JJA, 1JJB, 1JJC, 1JJE, 1JJF, 1JJH, 1JJI, 1JJK, 1JJO, 1JJT, 1JJU, 1JJV, 1JJW, 1JK0, 1JK1, 1JK2, 1JK3, 1JK4, 1JK6, 1JK7, 1JK8, 1JK9, 1JKA, 1JKB, 1JKC, 1JKD, 1JKE, 1JKF, 1JKG, 1JKH, 1JKI, 1JKJ, 1JKK, 1JKL, 1JKM, 1JKO, 1JKP, 1JKQ, 1JKR, 1JKS, 1JKT, 1JKU, 1JKV, 1JKW, 1JKX, 1JKY, 1JL0, 1JL1, 1JL2, 1JL3, 1JL4, 1JL5, 1JL6, 1JL7, 1JL8, 1JL9, 1JLA, 1JLB, 1JLC, 1JLD, 1JLE, 1JLF, 1JLG, 1JLH, 1JLJ, 1JLK, 1JLL, 1JLM, 1JLN, 1JLQ, 1JLR, 1JLS, 1JLT, 1JLU, 1JLV, 1JLW, 1JLX, 1JLY, 1JM0, 1JM1, 1JM6, 1JMA, 1JMB, 1JMC, 1JME, 1JMF, 1JMG, 1JMH, 1JMI, 1JMJ, 1JMK, 1JML, 1JMM, 1JMO, 1JMS, 1JMT, 1JMU, 1JMV, 1JMW, 1JMX, 1JMY, 1JMZ, 1JN0, 1JN1, 1JN2, 1JN3, 1JN4, 1JN5, 1JN6, 1JN9, 1JNB, 1JND, 1JNE, 1JNF, 1JNH, 1JNI, 1JNK, 1JNL, 1JNM, 1JNN, 1JNP, 1JNQ, 1JNR, 1JNU, 1JNV, 1JNW, 1JNX, 1JNY, 1JNZ, 1JO0, 1JO8, 1JOA, 1JOB, 1JOC, 1JOD, 1JOE, 1JOF, 1JOG, 1JOI, 1JOJ, 1JOL, 1JOM, 1JON, 1JOP, 1JOS, 1JOT, 1JOU, 1JOV, 1JOW, 1JP3, 1JP4, 1JP5, 1JP6, 1JP7, 1JP8, 1JP9, 1JPA, 1JPB, 1JPC, 1JPD, 1JPE, 1JPF, 1JPG, 1JPH, 1JPI, 1JPJ, 1JPK, 1JPL, 1JPM, 1JPN, 1JPO, 1JPP, 1JPQ, 1JPR, 1JPS, 1JPT, 1JPU, 1JPV, 1JPW, 1JPX, 1JPY, 1JPZ, 1JQ0, 1JQ3, 1JQ5, 1JQ6, 1JQ7, 1JQ8, 1JQ9, 1JQA, 1JQB, 1JQC, 1JQD, 1JQE, 1JQF, 1JQG, 1JQH, 1JQI, 1JQJ, 1JQK, 1JQL, 1JQN, 1JQO, 1JQP, 1JQQ, 1JQU, 1JQV, 1JQW, 1JQX, 1JQY, 1JQZ, 1JR0, 1JR1, 1JR2, 1JR3, 1JR4, 1JR7, 1JR8, 1JR9, 1JRA, 1JRB, 1JRC, 1JRE, 1JRG, 1JRH, 1JRI, 1JRK, 1JRL, 1JRN, 1JRO, 1JRP, 1JRQ, 1JRR, 1JRS, 1JRT, 1JRX, 1JRY, 1JRZ, 1JS0, 1JS1, 1JS2, 1JS3, 1JS4, 1JS6, 1JS8, 1JS9, 1JSC, 1JSD, 1JSE, 1JSF, 1JSG, 1JSH, 1JSI, 1JSL, 1JSM, 1JSN, 1JSO, 1JSR, 1JSS, 1JST, 1JSU, 1JSV, 1JSW, 1JSX, 1JSY, 1JSZ, 1JT0, 1JT1, 1JT2, 1JT3, 1JT4, 1JT5, 1JT6, 1JT7, 1JT9, 1JTA, 1JTC, 1JTD, 1JTE, 1JTF, 1JTG, 1JTH, 1JTI, 1JTK, 1JTL, 1JTM, 1JTN, 1JTO, 1JTP, 1JTQ, 1JTS, 1JTT, 1JTU, 1JTV, 1JTX, 1JTY, 1JTZ, 1JU2, 1JU3, 1JU4, 1JU6, 1JU9, 1JUB, 1JUC, 1JUD, 1JUE, 1JUF, 1JUG, 1JUH, 1JUI, 1JUJ, 1JUK, 1JUL, 1JUM, 1JUO, 1JUP, 1JUQ, 1JUS, 1JUT, 1JUV, 1JUX, 1JUY, 1JV0, 1JV1, 1JV2, 1JV3, 1JV4, 1JV5, 1JV6, 1JV7, 1JVA, 1JVB, 1JVD, 1JVG, 1JVI, 1JVJ, 1JVK, 1JVL, 1JVM, 1JVN, 1JVO, 1JVP, 1JVQ, 1JVS, 1JVT, 1JVU, 1JVV, 1JVW, 1JVX, 1JVY, 1JVZ, 1JW0, 1JW1, 1JW4, 1JW5, 1JW6, 1JW8, 1JW9, 1JWA, 1JWB, 1JWF, 1JWG, 1JWH, 1JWI, 1JWJ, 1JWK, 1JWL, 1JWM, 1JWN, 1JWO, 1JWP, 1JWQ, 1JWR, 1JWS, 1JWT, 1JWU, 1JWV, 1JWX, 1JWY, 1JWZ, 1JX0, 1JX1, 1JX2, 1JX4, 1JX6, 1JX7, 1JX9, 1JXA, 1JXB, 1JXE, 1JXG, 1JXH, 1JXI, 1JXJ, 1JXK, 1JXL, 1JXM, 1JXN, 1JXO, 1JXP, 1JXQ, 1JXT, 1JXU, 1JXV, 1JXW, 1JXX, 1JXY, 1JXZ, 1JY0, 1JY1, 1JY2, 1JY3, 1JY5, 1JY7, 1JY8, 1JYA, 1JYB, 1JYC, 1JYD, 1JYE, 1JYF, 1JYH, 1JYI, 1JYJ, 1JYK, 1JYL, 1JYM, 1JYN, 1JYO, 1JYQ, 1JYR, 1JYS, 1JYU, 1JYV, 1JYW, 1JYX, 1JYY, 1JYZ, 1JZ0, 1JZ1, 1JZ2, 1JZ3, 1JZ4, 1JZ5, 1JZ6, 1JZ7, 1JZ8, 1JZA, 1JZB, 1JZD, 1JZE, 1JZF, 1JZG, 1JZH, 1JZI, 1JZJ, 1JZK, 1JZL, 1JZM, 1JZN, 1JZO, 1JZQ, 1JZR, 1JZS, 1JZT, 1JZW, 1K02, 1K03, 1K04, 1K05, 1K06, 1K07, 1K08, 1K0A, 1K0B, 1K0C, 1K0D, 1K0E, 1K0F, 1K0G, 1K0I, 1K0J, 1K0K, 1K0L, 1K0M, 1K0N, 1K0O, 1K0R, 1K0U, 1K0W, 1K0Y, 1K0Z, 1K12, 1K1A, 1K1B, 1K1D, 1K1E, 1K1F, 1K1I, 1K1J, 1K1K, 1K1L, 1K1M, 1K1N, 1K1O, 1K1P, 1K1Q, 1K1S, 1K1T, 1K1U, 1K1W, 1K1X, 1K1Y, 1K20, 1K21, 1K22, 1K23, 1K24, 1K25, 1K26, 1K27, 1K28, 1K2A, 1K2B, 1K2C, 1K2D, 1K2E, 1K2F, 1K2I, 1K2L, 1K2O, 1K2P, 1K2R, 1K2S, 1K2T, 1K2U, 1K2V, 1K2W, 1K2X, 1K2Y, 1K2Z, 1K30, 1K32, 1K33, 1K34, 1K35, 1K38, 1K39, 1K3A, 1K3B, 1K3C, 1K3D, 1K3E, 1K3F, 1K3I, 1K3L, 1K3O, 1K3R, 1K3S, 1K3T, 1K3U, 1K3W, 1K3X, 1K3Y, 1K3Z, 1K40, 1K41, 1K44, 1K46, 1K47, 1K49, 1K4C, 1K4D, 1K4E, 1K4F, 1K4G, 1K4H, 1K4I, 1K4J, 1K4K, 1K4L, 1K4M, 1K4N, 1K4O, 1K4P, 1K4Q, 1K4S, 1K4T, 1K4V, 1K4W, 1K4Y, 1K4Z, 1K50, 1K51, 1K52, 1K53, 1K54, 1K55, 1K56, 1K57, 1K58, 1K59, 1K5A, 1K5B, 1K5C, 1K5D, 1K5G, 1K5H, 1K5J, 1K5N, 1K5P, 1K5Q, 1K5S, 1K5U, 1K5V, 1K61, 1K62, 1K63, 1K66, 1K68, 1K6A, 1K6C, 1K6D, 1K6E, 1K6F, 1K6I, 1K6J, 1K6K, 1K6L, 1K6M, 1K6N, 1K6O, 1K6P, 1K6Q, 1K6R, 1K6S, 1K6T, 1K6U, 1K6V, 1K6W, 1K6X, 1K6Y, 1K6Z, 1K70, 1K72, 1K74, 1K75, 1K77, 1K78, 1K79, 1K7A, 1K7C, 1K7D, 1K7E, 1K7F, 1K7G, 1K7H, 1K7I, 1K7J, 1K7K, 1K7L, 1K7Q, 1K7S, 1K7T, 1K7U, 1K7V, 1K7W, 1K7X, 1K7Y, 1K82, 1K83, 1K86, 1K88, 1K89, 1K8C, 1K8D, 1K8F, 1K8G, 1K8I, 1K8K, 1K8P, 1K8Q, 1K8R, 1K8T, 1K8U, 1K8X, 1K8Y, 1K8Z, 1K90, 1K92, 1K93, 1K94, 1K95, 1K96, 1K97, 1K98, 1K9A, 1K9B, 1K9D, 1K9E, 1K9F, 1K9I, 1K9J, 1K9K, 1K9O, 1K9P, 1K9S, 1K9T, 1K9U, 1K9V, 1K9X, 1K9Y, 1K9Z, 1KA0, 1KA1, 1KA2, 1KA4, 1KA8, 1KA9, 1KAA, 1KAB, 1KAC, 1KAE, 1KAF, 1KAG, 1KAH, 1KAK, 1KAM, 1KAN, 1KAO, 1KAP, 1KAQ, 1KAR, 1KAS, 1KAV, 1KAW, 1KAX, 1KAY, 1KAZ, 1KB0, 1KB2, 1KB3, 1KB4, 1KB5, 1KB6, 1KB9, 1KBA, 1KBB, 1KBC, 1KBG, 1KBI, 1KBJ, 1KBK, 1KBL, 1KBN, 1KBO, 1KBP, 1KBQ, 1KBR, 1KBU, 1KBV, 1KBW, 1KBY, 1KBZ, 1KC1, 1KC2, 1KC3, 1KC5, 1KC6, 1KC7, 1KCA, 1KCB, 1KCC, 1KCD, 1KCE, 1KCF, 1KCG, 1KCK, 1KCL, 1KCM, 1KCQ, 1KCR, 1KCS, 1KCT, 1KCU, 1KCV, 1KCW, 1KCX, 1KCZ, 1KD0, 1KD2, 1KD7, 1KD8, 1KD9, 1KDA, 1KDB, 1KDC, 1KDD, 1KDG, 1KDH, 1KDI, 1KDJ, 1KDK, 1KDM, 1KDN, 1KDO, 1KDP, 1KDQ, 1KDR, 1KDS, 1KDT, 1KDV, 1KDW, 1KDY, 1KDZ, 1KE0, 1KE1, 1KE2, 1KE3, 1KE4, 1KE5, 1KE6, 1KE7, 1KE8, 1KE9, 1KEA, 1KEB, 1KEC, 1KEE, 1KEG, 1KEH, 1KEI, 1KEJ, 1KEK, 1KEL, 1KEM, 1KEN, 1KEO, 1KEP, 1KEQ, 1KER, 1KET, 1KEU, 1KEV, 1KEW, 1KEX, 1KEY, 1KEZ, 1KF0, 1KF1, 1KF2, 1KF3, 1KF4, 1KF5, 1KF6, 1KF7, 1KF8, 1KF9, 1KFA, 1KFB, 1KFC, 1KFD, 1KFE, 1KFF, 1KFG, 1KFI, 1KFJ, 1KFK, 1KFL, 1KFM, 1KFN, 1KFQ, 1KFR, 1KFS, 1KFU, 1KFV, 1KFW, 1KFX, 1KFY, 1KG0, 1KG2, 1KG3, 1KG4, 1KG5, 1KG6, 1KG7, 1KG8, 1KG9, 1KGA, 1KGB, 1KGC, 1KGD, 1KGE, 1KGF, 1KGG, 1KGI, 1KGJ, 1KGN, 1KGO, 1KGP, 1KGQ, 1KGS, 1KGT, 1KGU, 1KGW, 1KGX, 1KGY, 1KGZ, 1KH0, 1KH1, 1KH2, 1KH3, 1KH4, 1KH5, 1KH7, 1KH8, 1KH9, 1KHB, 1KHC, 1KHD, 1KHE, 1KHF, 1KHG, 1KHH, 1KHI, 1KHJ, 1KHK, 1KHL, 1KHN, 1KHO, 1KHP, 1KHQ, 1KHR, 1KHT, 1KHU, 1KHV, 1KHW, 1KHX, 1KHY, 1KHZ, 1KI0, 1KI1, 1KI2, 1KI3, 1KI4, 1KI6, 1KI7, 1KI8, 1KI9, 1KIA, 1KIB, 1KIC, 1KID, 1KIE, 1KIF, 1KIG, 1KIJ, 1KIL, 1KIM, 1KIP, 1KIQ, 1KIR, 1KIT, 1KIU, 1KIV, 1KIX, 1KIY, 1KIZ, 1KJ1, 1KJ2, 1KJ3, 1KJ4, 1KJ7, 1KJ8, 1KJ9, 1KJF, 1KJG, 1KJH, 1KJI, 1KJJ, 1KJL, 1KJM, 1KJN, 1KJO, 1KJP, 1KJQ, 1KJR, 1KJT, 1KJV, 1KJW, 1KJX, 1KJY, 1KJZ, 1KK0, 1KK1, 1KK2, 1KK3, 1KK4, 1KK5, 1KK6, 1KK7, 1KK8, 1KK9, 1KKB, 1KKC, 1KKE, 1KKF, 1KKH, 1KKJ, 1KKK, 1KKL, 1KKM, 1KKO, 1KKP, 1KKQ, 1KKR, 1KKT, 1KKU, 1KL1, 1KL2, 1KL3, 1KL4, 1KL5, 1KL6, 1KL7, 1KL9, 1KLF, 1KLG, 1KLI, 1KLJ, 1KLK, 1KLL, 1KLM, 1KLN, 1KLO, 1KLT, 1KLU, 1KLX, 1KLY, 1KLZ, 1KM0, 1KM1, 1KM2, 1KM3, 1KM4, 1KM5, 1KM6, 1KM8, 1KM9, 1KMB, 1KMC, 1KME, 1KMH, 1KMI, 1KMJ, 1KMK, 1KMM, 1KMN, 1KMO, 1KMP, 1KMQ, 1KMS, 1KMT, 1KMV, 1KMY, 1KMZ, 1KN0, 1KN1, 1KN2, 1KN3, 1KN4, 1KN9, 1KNA, 1KNB, 1KNC, 1KND, 1KNE, 1KNF, 1KNG, 1KNI, 1KNJ, 1KNK, 1KNL, 1KNM, 1KNO, 1KNP, 1KNQ, 1KNR, 1KNT, 1KNU, 1KNV, 1KNW, 1KNX, 1KNY, 1KO0, 1KO1, 1KO2, 1KO3, 1KO4, 1KO5, 1KO6, 1KO7, 1KO8, 1KO9, 1KOA, 1KOB, 1KOE, 1KOF, 1KOH, 1KOI, 1KOJ, 1KOK, 1KOL, 1KON, 1KOO, 1KOP, 1KOQ, 1KOR, 1KOU, 1KP2, 1KP3, 1KP4, 1KP5, 1KP6, 1KP8, 1KP9, 1KPA, 1KPB, 1KPC, 1KPE, 1KPF, 1KPG, 1KPH, 1KPI, 1KPK, 1KPL, 1KPM, 1KPO, 1KPR, 1KPS, 1KPT, 1KPU, 1KPV, 1KQ0, 1KQ1, 1KQ3, 1KQ4, 1KQ5, 1KQ6, 1KQ7, 1KQ9, 1KQA, 1KQB, 1KQC, 1KQD, 1KQF, 1KQG, 1KQJ, 1KQL, 1KQM, 1KQN, 1KQO, 1KQP, 1KQR, 1KQU, 1KQW, 1KQX, 1KQY, 1KQZ, 1KR0, 1KR1, 1KR2, 1KR3, 1KR4, 1KR5, 1KR6, 1KR7, 1KRA, 1KRB, 1KRC, 1KRE, 1KRF, 1KRH, 1KRJ, 1KRL, 1KRM, 1KRN, 1KRO, 1KRP, 1KRQ, 1KRR, 1KRU, 1KRV, 1KS2, 1KS3, 1KS4, 1KS5, 1KS7, 1KS8, 1KS9, 1KSC, 1KSD, 1KSF, 1KSG, 1KSH, 1KSI, 1KSJ, 1KSK, 1KSL, 1KSN, 1KSO, 1KSP, 1KSS, 1KSU, 1KSV, 1KSW, 1KSX, 1KSY, 1KSZ, 1KT0, 1KT1, 1KT2, 1KT3, 1KT4, 1KT5, 1KT6, 1KT7, 1KT8, 1KT9, 1KTA, 1KTB, 1KTC, 1KTD, 1KTE, 1KTG, 1KTH, 1KTI, 1KTJ, 1KTK, 1KTL, 1KTN, 1KTO, 1KTP, 1KTQ, 1KTR, 1KTS, 1KTT, 1KTV, 1KTW, 1KTZ, 1KU0, 1KU1, 1KU2, 1KU3, 1KU5, 1KU6, 1KU7, 1KU8, 1KU9, 1KUF, 1KUG, 1KUH, 1KUI, 1KUJ, 1KUK, 1KUT, 1KUU, 1KUV, 1KUX, 1KUY, 1KV0, 1KV1, 1KV2, 1KV3, 1KV5, 1KV6, 1KV7, 1KV8, 1KV9, 1KVA, 1KVB, 1KVC, 1KVD, 1KVE, 1KVK, 1KVL, 1KVM, 1KVO, 1KVQ, 1KVR, 1KVS, 1KVT, 1KVU, 1KVW, 1KVX, 1KVY, 1KW0, 1KW1, 1KW2, 1KW3, 1KW4, 1KW5, 1KW6, 1KW7, 1KW8, 1KW9, 1KWA, 1KWB, 1KWC, 1KWF, 1KWG, 1KWH, 1KWI, 1KWK, 1KWM, 1KWN, 1KWO, 1KWP, 1KWQ, 1KWR, 1KWS, 1KWT, 1KWU, 1KWV, 1KWW, 1KWX, 1KWY, 1KWZ, 1KX0, 1KX1, 1KX3, 1KX4, 1KX5, 1KX8, 1KX9, 1KXA, 1KXB, 1KXC, 1KXD, 1KXE, 1KXF, 1KXG, 1KXH, 1KXI, 1KXJ, 1KXM, 1KXN, 1KXO, 1KXP, 1KXQ, 1KXR, 1KXT, 1KXU, 1KXV, 1KXW, 1KXX, 1KXY, 1KXZ, 1KY0, 1KY1, 1KY2, 1KY3, 1KY4, 1KY5, 1KY6, 1KY7, 1KY8, 1KY9, 1KYA, 1KYC, 1KYD, 1KYF, 1KYH, 1KYI, 1KYN, 1KYO, 1KYP, 1KYQ, 1KYR, 1KYS, 1KYT, 1KYU, 1KYV, 1KYW, 1KYX, 1KYY, 1KYZ, 1KZ1, 1KZ4, 1KZ6, 1KZ7, 1KZ8, 1KZ9, 1KZA, 1KZB, 1KZC, 1KZD, 1KZE, 1KZF, 1KZG, 1KZH, 1KZI, 1KZJ, 1KZK, 1KZL, 1KZM, 1KZN, 1KZO, 1KZP, 1KZQ, 1KZY, 1KZZ, 1L00, 1L01, 1L02, 1L03, 1L04, 1L05, 1L06, 1L07, 1L08, 1L09, 1L0A, 1L0B, 1L0C, 1L0D, 1L0E, 1L0F, 1L0G, 1L0H, 1L0I, 1L0J, 1L0K, 1L0L, 1L0N, 1L0O, 1L0P, 1L0Q, 1L0S, 1L0V, 1L0W, 1L0X, 1L0Y, 1L0Z, 1L10, 1L11, 1L12, 1L13, 1L14, 1L15, 1L16, 1L17, 1L18, 1L19, 1L1D, 1L1E, 1L1F, 1L1G, 1L1H, 1L1J, 1L1L, 1L1N, 1L1O, 1L1Q, 1L1R, 1L1S, 1L1T, 1L1Y, 1L1Z, 1L20, 1L21, 1L22, 1L23, 1L24, 1L25, 1L26, 1L27, 1L28, 1L29, 1L2A, 1L2B, 1L2C, 1L2D, 1L2E, 1L2F, 1L2G, 1L2H, 1L2I, 1L2J, 1L2L, 1L2O, 1L2P, 1L2Q, 1L2S, 1L2T, 1L2U, 1L2W, 1L30, 1L31, 1L32, 1L33, 1L34, 1L35, 1L36, 1L37, 1L38, 1L39, 1L3A, 1L3B, 1L3C, 1L3F, 1L3I, 1L3J, 1L3K, 1L3L, 1L3P, 1L3R, 1L3S, 1L3T, 1L3U, 1L3V, 1L3W, 1L40, 1L41, 1L42, 1L43, 1L44, 1L45, 1L46, 1L47, 1L48, 1L49, 1L4A, 1L4B, 1L4D, 1L4E, 1L4F, 1L4G, 1L4H, 1L4I, 1L4J, 1L4K, 1L4L, 1L4M, 1L4N, 1L4U, 1L4X, 1L4Y, 1L4Z, 1L50, 1L51, 1L52, 1L53, 1L54, 1L55, 1L56, 1L57, 1L58, 1L59, 1L5A, 1L5B, 1L5F, 1L5G, 1L5H, 1L5J, 1L5K, 1L5L, 1L5M, 1L5N, 1L5O, 1L5P, 1L5Q, 1L5R, 1L5S, 1L5T, 1L5U, 1L5V, 1L5W, 1L5X, 1L5Y, 1L5Z, 1L60, 1L61, 1L62, 1L63, 1L64, 1L65, 1L66, 1L67, 1L68, 1L69, 1L6B, 1L6F, 1L6G, 1L6I, 1L6J, 1L6L, 1L6M, 1L6O, 1L6P, 1L6R, 1L6S, 1L6W, 1L6X, 1L6Y, 1L6Z, 1L70, 1L71, 1L72, 1L73, 1L74, 1L75, 1L76, 1L77, 1L79, 1L7A, 1L7C, 1L7D, 1L7E, 1L7F, 1L7G, 1L7H, 1L7I, 1L7J, 1L7K, 1L7L, 1L7M, 1L7N, 1L7O, 1L7P, 1L7Q, 1L7R, 1L7T, 1L7V, 1L7X, 1L7Z, 1L80, 1L81, 1L82, 1L83, 1L84, 1L85, 1L86, 1L87, 1L88, 1L89, 1L8A, 1L8B, 1L8D, 1L8F, 1L8G, 1L8H, 1L8I, 1L8J, 1L8K, 1L8L, 1L8N, 1L8O, 1L8P, 1L8Q, 1L8R, 1L8S, 1L8T, 1L8W, 1L8X, 1L90, 1L91, 1L92, 1L93, 1L94, 1L95, 1L96, 1L97, 1L98, 1L99, 1L9B, 1L9C, 1L9D, 1L9E, 1L9G, 1L9H, 1L9J, 1L9K, 1L9L, 1L9M, 1L9N, 1L9O, 1L9P, 1L9Q, 1L9R, 1L9S, 1L9T, 1L9U, 1L9V, 1L9W, 1L9X, 1L9Y, 1L9Z, 1LA1, 1LA2, 1LA6, 1LAA, 1LAF, 1LAG, 1LAH, 1LAM, 1LAN, 1LAP, 1LAR, 1LAT, 1LAU, 1LAV, 1LAW, 1LAX, 1LAY, 1LB1, 1LB2, 1LB3, 1LB4, 1LB5, 1LB6, 1LB8, 1LB9, 1LBA, 1LBB, 1LBC, 1LBD, 1LBE, 1LBF, 1LBG, 1LBH, 1LBI, 1LBK, 1LBL, 1LBM, 1LBQ, 1LBS, 1LBT, 1LBU, 1LBV, 1LBW, 1LBX, 1LBY, 1LBZ, 1LC0, 1LC3, 1LC5, 1LC7, 1LC8, 1LCA, 1LCB, 1LCE, 1LCF, 1LCI, 1LCJ, 1LCK, 1LCL, 1LCN, 1LCO, 1LCP, 1LCS, 1LCT, 1LCU, 1LCV, 1LCW, 1LCY, 1LCZ, 1LD3, 1LD7, 1LD8, 1LD9, 1LDA, 1LDB, 1LDC, 1LDD, 1LDE, 1LDF, 1LDG, 1LDI, 1LDJ, 1LDK, 1LDM, 1LDN, 1LDO, 1LDP, 1LDQ, 1LDS, 1LDT, 1LDY, 1LE2, 1LE4, 1LE5, 1LE6, 1LE7, 1LE8, 1LE9, 1LEC, 1LED, 1LEE, 1LEG, 1LEH, 1LEI, 1LEK, 1LEL, 1LEM, 1LEN, 1LEO, 1LEP, 1LES, 1LEV, 1LEW, 1LEX, 1LEY, 1LEZ, 1LF0, 1LF1, 1LF2, 1LF3, 1LF4, 1LF5, 1LF6, 1LF7, 1LF8, 1LF9, 1LFA, 1LFB, 1LFD, 1LFG, 1LFH, 1LFI, 1LFK, 1LFL, 1LFM, 1LFO, 1LFP, 1LFQ, 1LFT, 1LFV, 1LFW, 1LFY, 1LFZ, 1LG1, 1LG2, 1LG5, 1LG6, 1LG7, 1LG9, 1LGA, 1LGB, 1LGC, 1LGD, 1LGF, 1LGN, 1LGP, 1LGQ, 1LGR, 1LGT, 1LGU, 1LGV, 1LGW, 1LGX, 1LGY, 1LH0, 1LH1, 1LH2, 1LH3, 1LH5, 1LH6, 1LH7, 1LHC, 1LHD, 1LHE, 1LHF, 1LHG, 1LHH, 1LHI, 1LHJ, 1LHK, 1LHL, 1LHM, 1LHN, 1LHO, 1LHP, 1LHR, 1LHS, 1LHT, 1LHU, 1LHV, 1LHW, 1LHY, 1LHZ, 1LI0, 1LI1, 1LI2, 1LI3, 1LI4, 1LI5, 1LI6, 1LI7, 1LI9, 1LIA, 1LIB, 1LIC, 1LID, 1LIE, 1LIF, 1LIH, 1LII, 1LIJ, 1LIK, 1LIL, 1LIN, 1LIO, 1LIS, 1LIT, 1LJ0, 1LJ1, 1LJ2, 1LJ3, 1LJ4, 1LJ5, 1LJ7, 1LJ8, 1LJ9, 1LJE, 1LJF, 1LJG, 1LJH, 1LJI, 1LJJ, 1LJK, 1LJL, 1LJM, 1LJN, 1LJO, 1LJP, 1LJR, 1LJT, 1LJU, 1LJW, 1LJX, 1LJY, 1LK0, 1LK2, 1LK3, 1LK5, 1LK6, 1LK7, 1LK9, 1LKA, 1LKB, 1LKC, 1LKD, 1LKE, 1LKF, 1LKI, 1LKK, 1LKL, 1LKM, 1LKO, 1LKP, 1LKR, 1LKS, 1LKT, 1LKV, 1LKX, 1LKY, 1LKZ, 1LL0, 1LL1, 1LL2, 1LL3, 1LL4, 1LL5, 1LL6, 1LL7, 1LL9, 1LLA, 1LLB, 1LLC, 1LLD, 1LLF, 1LLH, 1LLI, 1LLM, 1LLN, 1LLO, 1LLP, 1LLQ, 1LLR, 1LLS, 1LLT, 1LLU, 1LLW, 1LLZ, 1LM1, 1LM3, 1LM4, 1LM5, 1LM6, 1LM7, 1LM8, 1LMA, 1LMB, 1LMC, 1LME, 1LMH, 1LMI, 1LMK, 1LML, 1LMN, 1LMO, 1LMP, 1LMQ, 1LMT, 1LMW, 1LN0, 1LN1, 1LN2, 1LN3, 1LN4, 1LN8, 1LNA, 1LNB, 1LNC, 1LND, 1LNE, 1LNF, 1LNH, 1LNI, 1LNL, 1LNM, 1LNQ, 1LNS, 1LNU, 1LNW, 1LNX, 1LNY, 1LNZ, 1LO0, 1LO2, 1LO3, 1LO4, 1LO5, 1LO6, 1LO7, 1LO8, 1LO9, 1LOA, 1LOB, 1LOC, 1LOD, 1LOE, 1LOF, 1LOG, 1LOH, 1LOJ, 1LOK, 1LOL, 1LOM, 1LON, 1LOO, 1LOP, 1LOQ, 1LOR, 1LOS, 1LOT, 1LOU, 1LOV, 1LOW, 1LOX, 1LOY, 1LOZ, 1LP1, 1LP4, 1LP6, 1LP7, 1LP8, 1LP9, 1LPA, 1LPB, 1LPC, 1LPD, 1LPE, 1LPF, 1LPG, 1LPH, 1LPI, 1LPJ, 1LPK, 1LPL, 1LPM, 1LPN, 1LPO, 1LPP, 1LPQ, 1LPS, 1LPU, 1LPY, 1LPZ, 1LQ0, 1LQ1, 1LQ2, 1LQ8, 1LQ9, 1LQA, 1LQB, 1LQD, 1LQE, 1LQF, 1LQG, 1LQJ, 1LQK, 1LQL, 1LQM, 1LQO, 1LQP, 1LQS, 1LQT, 1LQU, 1LQV, 1LQW, 1LQX, 1LQY, 1LR0, 1LR2, 1LR3, 1LR4, 1LR5, 1LR6, 1LR7, 1LR8, 1LR9, 1LRA, 1LRH, 1LRI, 1LRJ, 1LRK, 1LRL, 1LRM, 1LRN, 1LRO, 1LRP, 1LRQ, 1LRR, 1LRT, 1LRU, 1LRV, 1LRW, 1LRY, 1LRZ, 1LS1, 1LS3, 1LS5, 1LS6, 1LS9, 1LSA, 1LSB, 1LSC, 1LSD, 1LSE, 1LSF, 1LSG, 1LSH, 1LSJ, 1LSL, 1LSM, 1LSN, 1LSO, 1LSP, 1LSQ, 1LSS, 1LST, 1LSU, 1LSV, 1LSW, 1LSX, 1LSY, 1LSZ, 1LT0, 1LT1, 1LT3, 1LT4, 1LT5, 1LT6, 1LT7, 1LT8, 1LT9, 1LTA, 1LTB, 1LTD, 1LTE, 1LTG, 1LTH, 1LTI, 1LTJ, 1LTK, 1LTL, 1LTM, 1LTO, 1LTQ, 1LTR, 1LTS, 1LTT, 1LTU, 1LTV, 1LTW, 1LTX, 1LTZ, 1LU0, 1LU1, 1LU2, 1LU4, 1LU5, 1LU9, 1LUA, 1LUC, 1LUE, 1LUF, 1LUG, 1LUJ, 1LUL, 1LUQ, 1LUR, 1LUV, 1LUW, 1LUZ, 1LV0, 1LV1, 1LV2, 1LV5, 1LV7, 1LV8, 1LVA, 1LVB, 1LVC, 1LVE, 1LVF, 1LVG, 1LVH, 1LVK, 1LVL, 1LVM, 1LVN, 1LVO, 1LVU, 1LVW, 1LVY, 1LW0, 1LW1, 1LW2, 1LW3, 1LW4, 1LW5, 1LW6, 1LW7, 1LW9, 1LWB, 1LWC, 1LWD, 1LWE, 1LWF, 1LWG, 1LWH, 1LWI, 1LWJ, 1LWK, 1LWL, 1LWN, 1LWO, 1LWS, 1LWT, 1LWU, 1LWV, 1LWW, 1LWX, 1LWY, 1LX5, 1LX6, 1LX7, 1LXA, 1LXC, 1LXD, 1LXE, 1LXI, 1LXJ, 1LXK, 1LXM, 1LXN, 1LXT, 1LXY, 1LXZ, 1LY0, 1LY1, 1LY2, 1LY3, 1LY4, 1LY8, 1LY9, 1LYA, 1LYB, 1LYC, 1LYD, 1LYE, 1LYF, 1LYG, 1LYH, 1LYI, 1LYJ, 1LYK, 1LYL, 1LYN, 1LYO, 1LYQ, 1LYS, 1LYV, 1LYW, 1LYX, 1LYY, 1LYZ, 1LZ0, 1LZ1, 1LZ2, 1LZ4, 1LZ5, 1LZ6, 1LZ7, 1LZ8, 1LZ9, 1LZA, 1LZB, 1LZC, 1LZD, 1LZE, 1LZG, 1LZH, 1LZI, 1LZJ, 1LZK, 1LZL, 1LZO, 1LZQ, 1LZR, 1LZS, 1LZT, 1LZV, 1LZW, 1LZX, 1LZY, 1LZZ, 1M00, 1M01, 1M03, 1M04, 1M05, 1M07, 1M08, 1M0B, 1M0D, 1M0E, 1M0I, 1M0K, 1M0L, 1M0M, 1M0N, 1M0O, 1M0P, 1M0Q, 1M0S, 1M0T, 1M0U, 1M0W, 1M0Z, 1M10, 1M13, 1M14, 1M15, 1M16, 1M17, 1M18, 1M19, 1M1A, 1M1B, 1M1D, 1M1E, 1M1F, 1M1G, 1M1H, 1M1J, 1M1L, 1M1M, 1M1N, 1M1O, 1M1S, 1M1T, 1M1U, 1M1X, 1M1Y, 1M1Z, 1M20, 1M21, 1M22, 1M26, 1M27, 1M2A, 1M2B, 1M2D, 1M2G, 1M2H, 1M2I, 1M2J, 1M2K, 1M2M, 1M2N, 1M2O, 1M2P, 1M2Q, 1M2R, 1M2T, 1M2V, 1M2W, 1M2X, 1M2Z, 1M32, 1M33, 1M34, 1M35, 1M38, 1M3D, 1M3E, 1M3H, 1M3I, 1M3J, 1M3K, 1M3Q, 1M3S, 1M3U, 1M3W, 1M3X, 1M3Y, 1M3Z, 1M40, 1M41, 1M43, 1M44, 1M45, 1M46, 1M47, 1M48, 1M49, 1M4A, 1M4B, 1M4C, 1M4D, 1M4G, 1M4H, 1M4I, 1M4J, 1M4K, 1M4L, 1M4M, 1M4N, 1M4R, 1M4S, 1M4T, 1M4U, 1M4V, 1M4W, 1M4Y, 1M4Z, 1M51, 1M52, 1M53, 1M54, 1M55, 1M56, 1M57, 1M59, 1M5A, 1M5B, 1M5C, 1M5D, 1M5E, 1M5F, 1M5H, 1M5I, 1M5N, 1M5Q, 1M5R, 1M5S, 1M5T, 1M5U, 1M5W, 1M5X, 1M5Y, 1M61, 1M63, 1M64, 1M65, 1M66, 1M67, 1M68, 1M6B, 1M6C, 1M6D, 1M6E, 1M6F, 1M6G, 1M6H, 1M6I, 1M6J, 1M6K, 1M6M, 1M6N, 1M6O, 1M6P, 1M6R, 1M6S, 1M6T, 1M6U, 1M6V, 1M6W, 1M6X, 1M6Y, 1M6Z, 1M70, 1M71, 1M72, 1M73, 1M74, 1M75, 1M76, 1M77, 1M78, 1M79, 1M7A, 1M7B, 1M7D, 1M7E, 1M7G, 1M7H, 1M7I, 1M7J, 1M7N, 1M7O, 1M7P, 1M7Q, 1M7R, 1M7S, 1M7U, 1M7V, 1M7W, 1M7X, 1M7Y, 1M7Z, 1M83, 1M85, 1M8A, 1M8D, 1M8E, 1M8F, 1M8G, 1M8H, 1M8I, 1M8J, 1M8K, 1M8N, 1M8P, 1M8R, 1M8S, 1M8T, 1M8U, 1M8Z, 1M93, 1M98, 1M99, 1M9A, 1M9B, 1M9C, 1M9D, 1M9E, 1M9F, 1M9H, 1M9I, 1M9J, 1M9K, 1M9M, 1M9N, 1M9P, 1M9Q, 1M9R, 1M9S, 1M9T, 1M9U, 1M9X, 1M9Y, 1M9Z, 1MA0, 1MA1, 1MA3, 1MA7, 1MA8, 1MA9, 1MAA, 1MAB, 1MAC, 1MAE, 1MAF, 1MAH, 1MAI, 1MAL, 1MAM, 1MAP, 1MAQ, 1MAR, 1MAS, 1MAT, 1MAU, 1MAV, 1MAW, 1MAX, 1MAY, 1MAZ, 1MB0, 1MB1, 1MB2, 1MB3, 1MB4, 1MB8, 1MB9, 1MBA, 1MBB, 1MBC, 1MBD, 1MBI, 1MBL, 1MBM, 1MBN, 1MBO, 1MBQ, 1MBS, 1MBT, 1MBU, 1MBV, 1MBX, 1MBY, 1MBZ, 1MC0, 1MC1, 1MC2, 1MC3, 1MC4, 1MC5, 1MC8, 1MC9, 1MCB, 1MCC, 1MCD, 1MCE, 1MCF, 1MCH, 1MCI, 1MCJ, 1MCK, 1MCN, 1MCO, 1MCP, 1MCQ, 1MCR, 1MCS, 1MCT, 1MCV, 1MCW, 1MCX, 1MCY, 1MCZ, 1MD0, 1MD2, 1MD3, 1MD4, 1MD6, 1MD7, 1MD8, 1MD9, 1MDA, 1MDB, 1MDC, 1MDF, 1MDL, 1MDM, 1MDN, 1MDO, 1MDP, 1MDQ, 1MDR, 1MDT, 1MDU, 1MDV, 1MDW, 1MDX, 1MDY, 1MDZ, 1ME3, 1ME4, 1ME5, 1ME6, 1ME7, 1ME8, 1ME9, 1MEE, 1MEG, 1MEH, 1MEI, 1MEJ, 1MEL, 1MEM, 1MEN, 1MEO, 1MEP, 1MER, 1MES, 1MET, 1MEU, 1MEW, 1MEX, 1MEY, 1MEZ, 1MF0, 1MF1, 1MF2, 1MF4, 1MF5, 1MF7, 1MF8, 1MFA, 1MFB, 1MFC, 1MFD, 1MFE, 1MFF, 1MFG, 1MFI, 1MFL, 1MFM, 1MFP, 1MFR, 1MFT, 1MFU, 1MFV, 1MFW, 1MFZ, 1MG0, 1MG1, 1MG2, 1MG3, 1MG4, 1MG5, 1MG6, 1MG7, 1MG9, 1MGN, 1MGO, 1MGP, 1MGQ, 1MGR, 1MGT, 1MGV, 1MGW, 1MGY, 1MH0, 1MH1, 1MH2, 1MH3, 1MH4, 1MH5, 1MH7, 1MH8, 1MH9, 1MHC, 1MHD, 1MHE, 1MHH, 1MHL, 1MHM, 1MHN, 1MHO, 1MHP, 1MHQ, 1MHT, 1MHW, 1MHX, 1MHY, 1MHZ, 1MI0, 1MI1, 1MI3, 1MI4, 1MI5, 1MI7, 1MI8, 1MID, 1MIE, 1MIF, 1MIH, 1MIJ, 1MIK, 1MIL, 1MIM, 1MIO, 1MIQ, 1MIR, 1MIU, 1MIV, 1MIW, 1MIX, 1MIY, 1MIZ, 1MJ0, 1MJ2, 1MJ3, 1MJ4, 1MJ5, 1MJ7, 1MJ8, 1MJ9, 1MJA, 1MJB, 1MJC, 1MJE, 1MJF, 1MJG, 1MJH, 1MJJ, 1MJK, 1MJL, 1MJM, 1MJN, 1MJO, 1MJP, 1MJQ, 1MJS, 1MJT, 1MJU, 1MJV, 1MJW, 1MJX, 1MJY, 1MJZ, 1MK0, 1MK1, 1MK2, 1MK4, 1MK5, 1MK7, 1MK8, 1MK9, 1MKA, 1MKB, 1MKD, 1MKF, 1MKG, 1MKH, 1MKI, 1MKJ, 1MKK, 1MKM, 1MKO, 1MKP, 1MKQ, 1MKR, 1MKS, 1MKT, 1MKU, 1MKV, 1MKW, 1MKX, 1MKY, 1MKZ, 1ML0, 1ML1, 1ML2, 1ML3, 1ML4, 1ML6, 1ML7, 1ML8, 1ML9, 1MLA, 1MLB, 1MLC, 1MLD, 1MLF, 1MLG, 1MLH, 1MLI, 1MLJ, 1MLK, 1MLL, 1MLM, 1MLN, 1MLO, 1MLQ, 1MLR, 1MLS, 1MLU, 1MLV, 1MLW, 1MLX, 1MLY, 1MLZ, 1MM6, 1MM7, 1MM8, 1MM9, 1MMA, 1MMB, 1MMD, 1MMF, 1MMG, 1MMI, 1MMJ, 1MMK, 1MML, 1MMM, 1MMN, 1MMO, 1MMP, 1MMQ, 1MMR, 1MMT, 1MMU, 1MMV, 1MMW, 1MMX, 1MMY, 1MMZ, 1MN0, 1MN1, 1MN2, 1MN3, 1MN4, 1MN6, 1MN7, 1MN8, 1MN9, 1MNA, 1MNC, 1MND, 1MNE, 1MNF, 1MNG, 1MNH, 1MNI, 1MNJ, 1MNK, 1MNM, 1MNN, 1MNO, 1MNP, 1MNQ, 1MNS, 1MNU, 1MNZ, 1MO0, 1MO1, 1MO2, 1MO3, 1MO4, 1MO5, 1MO6, 1MO9, 1MOA, 1MOB, 1MOC, 1MOD, 1MOE, 1MOF, 1MOG, 1MOH, 1MOJ, 1MOK, 1MOL, 1MOM, 1MOO, 1MOP, 1MOQ, 1MOR, 1MOS, 1MOU, 1MOV, 1MOW, 1MOX, 1MOY, 1MOZ, 1MP0, 1MP2, 1MP3, 1MP4, 1MP5, 1MP8, 1MP9, 1MPA, 1MPB, 1MPC, 1MPD, 1MPF, 1MPG, 1MPJ, 1MPL, 1MPM, 1MPN, 1MPO, 1MPP, 1MPQ, 1MPR, 1MPS, 1MPT, 1MPU, 1MPW, 1MPX, 1MPY, 1MQ0, 1MQ2, 1MQ3, 1MQ4, 1MQ5, 1MQ6, 1MQ7, 1MQ8, 1MQ9, 1MQA, 1MQB, 1MQD, 1MQE, 1MQF, 1MQG, 1MQH, 1MQI, 1MQJ, 1MQK, 1MQL, 1MQM, 1MQN, 1MQO, 1MQP, 1MQQ, 1MQR, 1MQS, 1MQV, 1MQW, 1MR1, 1MR2, 1MR3, 1MR5, 1MR7, 1MR8, 1MR9, 1MRA, 1MRC, 1MRD, 1MRE, 1MRF, 1MRG, 1MRH, 1MRI, 1MRJ, 1MRK, 1MRL, 1MRN, 1MRO, 1MRP, 1MRQ, 1MRR, 1MRS, 1MRU, 1MRV, 1MRW, 1MRX, 1MRY, 1MRZ, 1MS0, 1MS1, 1MS3, 1MS4, 1MS5, 1MS6, 1MS7, 1MS8, 1MS9, 1MSA, 1MSB, 1MSC, 1MSD, 1MSI, 1MSJ, 1MSK, 1MSM, 1MSN, 1MSO, 1MSP, 1MSS, 1MST, 1MSV, 1MT0, 1MT1, 1MT3, 1MT5, 1MT6, 1MT7, 1MT8, 1MT9, 1MTB, 1MTC, 1MTI, 1MTJ, 1MTK, 1MTL, 1MTN, 1MTO, 1MTP, 1MTR, 1MTS, 1MTU, 1MTV, 1MTW, 1MTY, 1MTZ, 1MU0, 1MU2, 1MU4, 1MU5, 1MU6, 1MU7, 1MU8, 1MU9, 1MUA, 1MUC, 1MUD, 1MUE, 1MUF, 1MUG, 1MUH, 1MUI, 1MUJ, 1MUK, 1MUL, 1MUM, 1MUN, 1MUO, 1MUP, 1MUQ, 1MUS, 1MUU, 1MUW, 1MUY, 1MV5, 1MV8, 1MV9, 1MVA, 1MVB, 1MVC, 1MVE, 1MVF, 1MVH, 1MVK, 1MVL, 1MVN, 1MVO, 1MVP, 1MVQ, 1MVS, 1MVT, 1MVU, 1MVX, 1MVY, 1MW0, 1MW1, 1MW2, 1MW3, 1MW5, 1MW7, 1MW8, 1MW9, 1MWA, 1MWC, 1MWD, 1MWE, 1MWH, 1MWI, 1MWJ, 1MWK, 1MWM, 1MWO, 1MWP, 1MWQ, 1MWR, 1MWS, 1MWT, 1MWU, 1MWV, 1MWW, 1MX0, 1MX1, 1MX2, 1MX3, 1MX4, 1MX5, 1MX6, 1MX9, 1MXA, 1MXB, 1MXC, 1MXD, 1MXE, 1MXF, 1MXG, 1MXH, 1MXI, 1MXO, 1MXR, 1MXS, 1MXT, 1MXU, 1MXV, 1MXW, 1MXX, 1MXY, 1MXZ, 1MY0, 1MY1, 1MY2, 1MY3, 1MY4, 1MY5, 1MY6, 1MY7, 1MY8, 1MYG, 1MYH, 1MYI, 1MYJ, 1MYK, 1MYL, 1MYM, 1MYP, 1MYR, 1MYT, 1MYW, 1MYZ, 1MZ0, 1MZ4, 1MZ5, 1MZ6, 1MZ8, 1MZ9, 1MZA, 1MZB, 1MZC, 1MZD, 1MZE, 1MZF, 1MZG, 1MZH, 1MZJ, 1MZL, 1MZM, 1MZN, 1MZO, 1MZR, 1MZS, 1MZU, 1MZV, 1MZW, 1MZY, 1MZZ, 1N00, 1N04, 1N05, 1N06, 1N07, 1N08, 1N0E, 1N0F, 1N0G, 1N0H, 1N0I, 1N0J, 1N0L, 1N0N, 1N0Q, 1N0R, 1N0S, 1N0T, 1N0U, 1N0V, 1N0W, 1N0X, 1N0Y, 1N10, 1N11, 1N12, 1N13, 1N15, 1N18, 1N19, 1N1A, 1N1B, 1N1C, 1N1D, 1N1E, 1N1F, 1N1G, 1N1I, 1N1J, 1N1L, 1N1M, 1N1O, 1N1P, 1N1Q, 1N1S, 1N1T, 1N1V, 1N1X, 1N1Y, 1N1Z, 1N20, 1N21, 1N22, 1N23, 1N24, 1N25, 1N26, 1N28, 1N29, 1N2A, 1N2B, 1N2C, 1N2D, 1N2E, 1N2F, 1N2G, 1N2H, 1N2I, 1N2J, 1N2K, 1N2L, 1N2M, 1N2N, 1N2O, 1N2R, 1N2S, 1N2T, 1N2V, 1N2X, 1N2Z, 1N31, 1N39, 1N3A, 1N3B, 1N3C, 1N3E, 1N3F, 1N3I, 1N3L, 1N3N, 1N3O, 1N3P, 1N3Q, 1N3R, 1N3S, 1N3T, 1N3U, 1N3W, 1N3X, 1N3Y, 1N3Z, 1N40, 1N41, 1N42, 1N43, 1N44, 1N45, 1N46, 1N47, 1N48, 1N49, 1N4A, 1N4D, 1N4E, 1N4F, 1N4G, 1N4H, 1N4J, 1N4K, 1N4L, 1N4M, 1N4O, 1N4P, 1N4Q, 1N4R, 1N4S, 1N4U, 1N4V, 1N4W, 1N4X, 1N50, 1N51, 1N52, 1N54, 1N55, 1N56, 1N57, 1N59, 1N5A, 1N5B, 1N5C, 1N5D, 1N5I, 1N5J, 1N5K, 1N5L, 1N5M, 1N5N, 1N5O, 1N5Q, 1N5R, 1N5S, 1N5T, 1N5U, 1N5V, 1N5W, 1N5X, 1N5Y, 1N5Z, 1N60, 1N61, 1N62, 1N63, 1N64, 1N67, 1N68, 1N69, 1N6A, 1N6B, 1N6C, 1N6D, 1N6E, 1N6F, 1N6H, 1N6I, 1N6J, 1N6K, 1N6L, 1N6M, 1N6N, 1N6O, 1N6P, 1N6Q, 1N6R, 1N6X, 1N6Y, 1N70, 1N71, 1N73, 1N75, 1N76, 1N7D, 1N7E, 1N7F, 1N7G, 1N7H, 1N7I, 1N7J, 1N7K, 1N7M, 1N7N, 1N7O, 1N7P, 1N7Q, 1N7R, 1N7S, 1N7U, 1N7V, 1N7W, 1N7X, 1N7Y, 1N7Z, 1N80, 1N81, 1N82, 1N83, 1N84, 1N86, 1N8B, 1N8E, 1N8F, 1N8I, 1N8J, 1N8K, 1N8N, 1N8O, 1N8P, 1N8Q, 1N8S, 1N8T, 1N8U, 1N8V, 1N8W, 1N8Y, 1N8Z, 1N90, 1N92, 1N93, 1N94, 1N95, 1N97, 1N99, 1N9A, 1N9B, 1N9E, 1N9F, 1N9G, 1N9H, 1N9I, 1N9K, 1N9L, 1N9M, 1N9N, 1N9O, 1N9P, 1N9R, 1N9S, 1N9W, 1N9X, 1N9Y, 1N9Z, 1NA0, 1NA3, 1NA5, 1NA6, 1NA7, 1NA8, 1NAA, 1NAE, 1NAF, 1NAG, 1NAH, 1NAI, 1NAK, 1NAL, 1NAM, 1NAN, 1NAP, 1NAQ, 1NAR, 1NAS, 1NAT, 1NAV, 1NAW, 1NAX, 1NAY, 1NAZ, 1NB0, 1NB2, 1NB3, 1NB4, 1NB5, 1NB6, 1NB8, 1NB9, 1NBA, 1NBB, 1NBC, 1NBE, 1NBF, 1NBH, 1NBI, 1NBM, 1NBO, 1NBP, 1NBQ, 1NBU, 1NBV, 1NBW, 1NBX, 1NBY, 1NBZ, 1NC1, 1NC2, 1NC3, 1NC4, 1NC5, 1NC6, 1NC7, 1NC9, 1NCA, 1NCB, 1NCC, 1NCD, 1NCE, 1NCF, 1NCG, 1NCH, 1NCI, 1NCJ, 1NCL, 1NCN, 1NCO, 1NCW, 1NCX, 1NCY, 1NCZ, 1ND0, 1ND1, 1ND4, 1ND5, 1ND6, 1ND7, 1NDA, 1NDB, 1NDC, 1NDD, 1NDE, 1NDF, 1NDG, 1NDH, 1NDI, 1NDJ, 1NDK, 1NDL, 1NDM, 1NDN, 1NDO, 1NDP, 1NDQ, 1NDR, 1NDS, 1NDT, 1NDU, 1NDV, 1NDW, 1NDY, 1NDZ, 1NE2, 1NE4, 1NE6, 1NE7, 1NE8, 1NE9, 1NEC, 1NED, 1NEG, 1NEJ, 1NEK, 1NEL, 1NEN, 1NEP, 1NES, 1NEU, 1NEX, 1NEY, 1NEZ, 1NF0, 1NF1, 1NF2, 1NF3, 1NF4, 1NF5, 1NF6, 1NF7, 1NF8, 1NF9, 1NFB, 1NFD, 1NFF, 1NFG, 1NFH, 1NFI, 1NFJ, 1NFK, 1NFN, 1NFO, 1NFP, 1NFQ, 1NFR, 1NFS, 1NFT, 1NFU, 1NFV, 1NFW, 1NFX, 1NFY, 1NFZ, 1NG1, 1NG2, 1NG3, 1NG4, 1NG5, 1NG6, 1NG9, 1NGA, 1NGB, 1NGC, 1NGD, 1NGE, 1NGF, 1NGG, 1NGH, 1NGI, 1NGJ, 1NGK, 1NGM, 1NGN, 1NGP, 1NGQ, 1NGS, 1NGT, 1NGW, 1NGX, 1NGY, 1NGZ, 1NH0, 1NH1, 1NH2, 1NH3, 1NH6, 1NH7, 1NH8, 1NH9, 1NHB, 1NHC, 1NHE, 1NHG, 1NHH, 1NHI, 1NHJ, 1NHK, 1NHL, 1NHP, 1NHQ, 1NHR, 1NHS, 1NHT, 1NHU, 1NHV, 1NHW, 1NHX, 1NHY, 1NHZ, 1NI0, 1NI1, 1NI2, 1NI3, 1NI4, 1NI5, 1NI6, 1NI9, 1NIA, 1NIB, 1NIC, 1NID, 1NIE, 1NIF, 1NIG, 1NIH, 1NIJ, 1NIK, 1NIO, 1NIP, 1NIR, 1NIS, 1NIT, 1NIU, 1NIV, 1NIW, 1NJ1, 1NJ2, 1NJ4, 1NJ5, 1NJ6, 1NJ8, 1NJ9, 1NJA, 1NJB, 1NJC, 1NJD, 1NJE, 1NJF, 1NJG, 1NJH, 1NJJ, 1NJK, 1NJR, 1NJS, 1NJT, 1NJU, 1NJW, 1NJX, 1NJY, 1NJZ, 1NK0, 1NK1, 1NK4, 1NK5, 1NK6, 1NK7, 1NK8, 1NK9, 1NKB, 1NKC, 1NKD, 1NKE, 1NKG, 1NKH, 1NKI, 1NKK, 1NKM, 1NKN, 1NKO, 1NKP, 1NKQ, 1NKR, 1NKS, 1NKT, 1NKV, 1NKX, 1NL0, 1NL1, 1NL2, 1NL3, 1NL4, 1NL5, 1NL6, 1NL7, 1NL9, 1NLB, 1NLD, 1NLF, 1NLI, 1NLJ, 1NLK, 1NLM, 1NLN, 1NLQ, 1NLR, 1NLS, 1NLT, 1NLU, 1NLV, 1NLW, 1NLX, 1NLY, 1NLZ, 1NM0, 1NM1, 1NM2, 1NM3, 1NM5, 1NM6, 1NM8, 1NM9, 1NMA, 1NMB, 1NMC, 1NMD, 1NME, 1NMK, 1NML, 1NMM, 1NMN, 1NMO, 1NMP, 1NMQ, 1NMS, 1NMT, 1NMU, 1NMX, 1NMY, 1NMZ, 1NN0, 1NN1, 1NN2, 1NN3, 1NN4, 1NN5, 1NN6, 1NN7, 1NNA, 1NNB, 1NNC, 1NND, 1NNE, 1NNF, 1NNH, 1NNI, 1NNJ, 1NNK, 1NNL, 1NNO, 1NNP, 1NNQ, 1NNR, 1NNS, 1NNT, 1NNU, 1NNW, 1NNX, 1NNY, 1NO1, 1NO3, 1NO4, 1NO5, 1NO6, 1NO7, 1NO9, 1NOA, 1NOB, 1NOC, 1NOD, 1NOF, 1NOG, 1NOH, 1NOI, 1NOJ, 1NOK, 1NOL, 1NOM, 1NON, 1NOO, 1NOP, 1NOS, 1NOT, 1NOU, 1NOW, 1NOX, 1NOY, 1NOZ, 1NP0, 1NP1, 1NP2, 1NP3, 1NP4, 1NP6, 1NP7, 1NP8, 1NPA, 1NPB, 1NPC, 1NPD, 1NPE, 1NPF, 1NPG, 1NPH, 1NPI, 1NPJ, 1NPK, 1NPL, 1NPM, 1NPN, 1NPO, 1NPP, 1NPR, 1NPS, 1NPT, 1NPU, 1NPV, 1NPW, 1NPX, 1NPY, 1NPZ, 1NQ0, 1NQ1, 1NQ2, 1NQ3, 1NQ5, 1NQ6, 1NQ7, 1NQ9, 1NQA, 1NQB, 1NQC, 1NQD, 1NQE, 1NQF, 1NQG, 1NQH, 1NQI, 1NQJ, 1NQK, 1NQL, 1NQM, 1NQN, 1NQO, 1NQP, 1NQS, 1NQT, 1NQU, 1NQV, 1NQW, 1NQX, 1NQY, 1NQZ, 1NR0, 1NR1, 1NR2, 1NR4, 1NR5, 1NR6, 1NR7, 1NR9, 1NRF, 1NRG, 1NRI, 1NRJ, 1NRK, 1NRL, 1NRN, 1NRO, 1NRP, 1NRQ, 1NRR, 1NRS, 1NRV, 1NRW, 1NRX, 1NRZ, 1NS0, 1NS2, 1NS3, 1NS4, 1NS5, 1NS6, 1NS7, 1NS8, 1NS9, 1NSA, 1NSB, 1NSC, 1NSD, 1NSE, 1NSF, 1NSG, 1NSI, 1NSJ, 1NSK, 1NSL, 1NSM, 1NSN, 1NSP, 1NSQ, 1NSR, 1NSS, 1NST, 1NSU, 1NSV, 1NSW, 1NSX, 1NSY, 1NSZ, 1NT0, 1NT1, 1NT2, 1NT3, 1NT4, 1NT8, 1NT9, 1NTD, 1NTE, 1NTF, 1NTG, 1NTH, 1NTK, 1NTM, 1NTN, 1NTO, 1NTV, 1NTY, 1NTZ, 1NU0, 1NU1, 1NU2, 1NU3, 1NU4, 1NU5, 1NU6, 1NU7, 1NU8, 1NU9, 1NUA, 1NUB, 1NUC, 1NUD, 1NUE, 1NUF, 1NUG, 1NUH, 1NUI, 1NUK, 1NUL, 1NUN, 1NUO, 1NUP, 1NUQ, 1NUR, 1NUS, 1NUT, 1NUU, 1NUW, 1NUX, 1NUY, 1NUZ, 1NV0, 1NV1, 1NV2, 1NV3, 1NV4, 1NV5, 1NV6, 1NV7, 1NV8, 1NV9, 1NVA, 1NVB, 1NVD, 1NVE, 1NVF, 1NVG, 1NVI, 1NVJ, 1NVK, 1NVM, 1NVN, 1NVP, 1NVQ, 1NVR, 1NVS, 1NVT, 1NVU, 1NVV, 1NVW, 1NVX, 1NVY, 1NW1, 1NW2, 1NW3, 1NW4, 1NW5, 1NW6, 1NW7, 1NW8, 1NW9, 1NWA, 1NWC, 1NWE, 1NWG, 1NWH, 1NWI, 1NWK, 1NWL, 1NWM, 1NWN, 1NWO, 1NWP, 1NWQ, 1NWR, 1NWS, 1NWT, 1NWU, 1NWW, 1NWZ, 1NX0, 1NX1, 1NX2, 1NX3, 1NX4, 1NX6, 1NX8, 1NX9, 1NXB, 1NXC, 1NXD, 1NXE, 1NXF, 1NXG, 1NXH, 1NXJ, 1NXK, 1NXM, 1NXO, 1NXP, 1NXQ, 1NXS, 1NXT, 1NXU, 1NXV, 1NXW, 1NXX, 1NXY, 1NXZ, 1NY0, 1NY1, 1NY2, 1NY3, 1NY5, 1NY6, 1NYC, 1NYE, 1NYH, 1NYK, 1NYL, 1NYM, 1NYQ, 1NYR, 1NYS, 1NYT, 1NYU, 1NYW, 1NYX, 1NYY, 1NZ0, 1NZ2, 1NZ3, 1NZ4, 1NZ5, 1NZ6, 1NZ7, 1NZA, 1NZB, 1NZC, 1NZD, 1NZE, 1NZF, 1NZG, 1NZI, 1NZJ, 1NZK, 1NZL, 1NZN, 1NZO, 1NZQ, 1NZR, 1NZU, 1NZV, 1NZW, 1NZX, 1NZY, 1NZZ, 1O00, 1O01, 1O02, 1O03, 1O04, 1O05, 1O06, 1O07, 1O08, 1O0A, 1O0D, 1O0E, 1O0F, 1O0H, 1O0M, 1O0N, 1O0O, 1O0Q, 1O0R, 1O0S, 1O0T, 1O0V, 1O0W, 1O0X, 1O12, 1O13, 1O16, 1O17, 1O1H, 1O1I, 1O1J, 1O1K, 1O1L, 1O1M, 1O1N, 1O1O, 1O1P, 1O1R, 1O1S, 1O1T, 1O1X, 1O1Y, 1O1Z, 1O20, 1O22, 1O23, 1O24, 1O25, 1O26, 1O27, 1O28, 1O29, 1O2A, 1O2B, 1O2D, 1O2E, 1O2G, 1O2H, 1O2I, 1O2J, 1O2K, 1O2L, 1O2M, 1O2N, 1O2O, 1O2P, 1O2Q, 1O2R, 1O2S, 1O2T, 1O2U, 1O2V, 1O2W, 1O2X, 1O2Y, 1O2Z, 1O30, 1O31, 1O32, 1O33, 1O34, 1O35, 1O36, 1O37, 1O38, 1O39, 1O3A, 1O3B, 1O3C, 1O3D, 1O3E, 1O3F, 1O3G, 1O3H, 1O3I, 1O3J, 1O3K, 1O3L, 1O3M, 1O3N, 1O3O, 1O3P, 1O3Q, 1O3R, 1O3S, 1O3T, 1O3U, 1O3W, 1O3X, 1O3Y, 1O41, 1O42, 1O43, 1O44, 1O45, 1O46, 1O47, 1O48, 1O49, 1O4A, 1O4B, 1O4C, 1O4D, 1O4E, 1O4F, 1O4G, 1O4H, 1O4I, 1O4J, 1O4K, 1O4L, 1O4M, 1O4N, 1O4O, 1O4P, 1O4Q, 1O4R, 1O4S, 1O4T, 1O4U, 1O4V, 1O4W, 1O4Y, 1O4Z, 1O50, 1O51, 1O54, 1O55, 1O56, 1O57, 1O58, 1O59, 1O5A, 1O5B, 1O5C, 1O5D, 1O5E, 1O5F, 1O5G, 1O5H, 1O5I, 1O5J, 1O5K, 1O5L, 1O5M, 1O5O, 1O5Q, 1O5R, 1O5T, 1O5U, 1O5W, 1O5X, 1O5Z, 1O60, 1O61, 1O62, 1O63, 1O64, 1O65, 1O66, 1O67, 1O68, 1O69, 1O6A, 1O6B, 1O6C, 1O6D, 1O6E, 1O6F, 1O6G, 1O6H, 1O6I, 1O6J, 1O6K, 1O6L, 1O6O, 1O6P, 1O6Q, 1O6R, 1O6S, 1O6T, 1O6U, 1O6V, 1O6Y, 1O6Z, 1O70, 1O71, 1O72, 1O73, 1O75, 1O76, 1O77, 1O79, 1O7A, 1O7D, 1O7E, 1O7F, 1O7G, 1O7H, 1O7I, 1O7J, 1O7K, 1O7L, 1O7M, 1O7N, 1O7O, 1O7P, 1O7Q, 1O7S, 1O7T, 1O7U, 1O7V, 1O7W, 1O7X, 1O7Y, 1O7Z, 1O80, 1O81, 1O82, 1O83, 1O84, 1O85, 1O86, 1O87, 1O88, 1O89, 1O8A, 1O8B, 1O8C, 1O8D, 1O8E, 1O8F, 1O8G, 1O8H, 1O8I, 1O8J, 1O8K, 1O8L, 1O8M, 1O8N, 1O8O, 1O8P, 1O8Q, 1O8S, 1O8U, 1O8V, 1O8W, 1O8X, 1O90, 1O91, 1O92, 1O93, 1O94, 1O95, 1O96, 1O97, 1O98, 1O99, 1O9B, 1O9C, 1O9D, 1O9E, 1O9F, 1O9G, 1O9H, 1O9I, 1O9J, 1O9K, 1O9L, 1O9N, 1O9O, 1O9P, 1O9Q, 1O9R, 1O9S, 1O9T, 1O9U, 1O9V, 1O9W, 1O9X, 1O9Y, 1O9Z, 1OA0, 1OA1, 1OA2, 1OA3, 1OA4, 1OA7, 1OA8, 1OA9, 1OAA, 1OAB, 1OAC, 1OAD, 1OAE, 1OAF, 1OAG, 1OAH, 1OAI, 1OAJ, 1OAK, 1OAL, 1OAN, 1OAO, 1OAP, 1OAQ, 1OAR, 1OAS, 1OAT, 1OAU, 1OAX, 1OAY, 1OAZ, 1OB0, 1OB1, 1OB3, 1OB8, 1OB9, 1OBA, 1OBB, 1OBC, 1OBD, 1OBF, 1OBG, 1OBH, 1OBI, 1OBJ, 1OBK, 1OBL, 1OBM, 1OBN, 1OBO, 1OBP, 1OBQ, 1OBR, 1OBS, 1OBT, 1OBU, 1OBV, 1OBW, 1OBX, 1OBY, 1OBZ, 1OC0, 1OC1, 1OC2, 1OC3, 1OC4, 1OC5, 1OC6, 1OC7, 1OC8, 1OC9, 1OCB, 1OCC, 1OCE, 1OCH, 1OCJ, 1OCK, 1OCL, 1OCM, 1OCN, 1OCO, 1OCQ, 1OCR, 1OCS, 1OCT, 1OCU, 1OCV, 1OCW, 1OCX, 1OCY, 1OCZ, 1OD0, 1OD1, 1OD2, 1OD3, 1OD4, 1OD5, 1OD6, 1OD7, 1OD8, 1OD9, 1ODA, 1ODB, 1ODC, 1ODD, 1ODE, 1ODF, 1ODG, 1ODH, 1ODI, 1ODJ, 1ODK, 1ODL, 1ODM, 1ODN, 1ODO, 1ODS, 1ODT, 1ODU, 1ODV, 1ODW, 1ODX, 1ODY, 1ODZ, 1OE0, 1OE1, 1OE2, 1OE3, 1OE4, 1OE5, 1OE6, 1OE7, 1OE8, 1OE9, 1OEB, 1OEC, 1OEE, 1OEJ, 1OEK, 1OEL, 1OEM, 1OEN, 1OEO, 1OEP, 1OES, 1OET, 1OEU, 1OEV, 1OEW, 1OEX, 1OEY, 1OEZ, 1OF0, 1OF1, 1OF2, 1OF3, 1OF4, 1OF5, 1OF6, 1OF8, 1OFA, 1OFB, 1OFC, 1OFD, 1OFE, 1OFF, 1OFG, 1OFH, 1OFI, 1OFJ, 1OFK, 1OFL, 1OFM, 1OFN, 1OFO, 1OFP, 1OFQ, 1OFR, 1OFS, 1OFT, 1OFU, 1OFV, 1OFW, 1OFY, 1OFZ, 1OG0, 1OG1, 1OG2, 1OG3, 1OG4, 1OG5, 1OG6, 1OGA, 1OGB, 1OGC, 1OGD, 1OGE, 1OGF, 1OGG, 1OGH, 1OGI, 1OGJ, 1OGK, 1OGL, 1OGM, 1OGO, 1OGP, 1OGQ, 1OGS, 1OGT, 1OGU, 1OGV, 1OGW, 1OGX, 1OGY, 1OGZ, 1OH0, 1OH2, 1OH3, 1OH4, 1OH5, 1OH6, 1OH7, 1OH8, 1OH9, 1OHA, 1OHB, 1OHC, 1OHD, 1OHE, 1OHH, 1OHJ, 1OHK, 1OHL, 1OHO, 1OHP, 1OHQ, 1OHR, 1OHS, 1OHT, 1OHU, 1OHV, 1OHW, 1OHY, 1OHZ, 1OI0, 1OI1, 1OI2, 1OI3, 1OI4, 1OI6, 1OI7, 1OI8, 1OI9, 1OIA, 1OIB, 1OID, 1OIE, 1OIF, 1OIH, 1OII, 1OIJ, 1OIK, 1OIL, 1OIM, 1OIN, 1OIO, 1OIP, 1OIQ, 1OIR, 1OIS, 1OIT, 1OIU, 1OIV, 1OIW, 1OIX, 1OIY, 1OIZ, 1OJ1, 1OJ4, 1OJ5, 1OJ6, 1OJ7, 1OJ8, 1OJ9, 1OJA, 1OJC, 1OJD, 1OJH, 1OJI, 1OJJ, 1OJK, 1OJL, 1OJM, 1OJN, 1OJO, 1OJP, 1OJQ, 1OJR, 1OJT, 1OJV, 1OJW, 1OJX, 1OJY, 1OJZ, 1OK0, 1OK1, 1OK2, 1OK3, 1OK4, 1OK6, 1OK7, 1OK8, 1OK9, 1OKB, 1OKC, 1OKE, 1OKG, 1OKH, 1OKI, 1OKJ, 1OKK, 1OKL, 1OKM, 1OKN, 1OKO, 1OKQ, 1OKR, 1OKS, 1OKT, 1OKV, 1OKW, 1OKX, 1OKY, 1OKZ, 1OL0, 1OL1, 1OL2, 1OL5, 1OL6, 1OL7, 1OLA, 1OLC, 1OLL, 1OLM, 1OLO, 1OLP, 1OLQ, 1OLR, 1OLS, 1OLT, 1OLU, 1OLX, 1OLZ, 1OM0, 1OM1, 1OM3, 1OM4, 1OM5, 1OM6, 1OM7, 1OM8, 1OM9, 1OMD, 1OME, 1OMH, 1OMI, 1OMJ, 1OMO, 1OMP, 1OMR, 1OMS, 1OMV, 1OMW, 1OMX, 1OMY, 1OMZ, 1ON0, 1ON1, 1ON2, 1ON3, 1ON6, 1ON7, 1ON8, 1ON9, 1ONA, 1ONC, 1ONE, 1ONF, 1ONG, 1ONH, 1ONI, 1ONJ, 1ONK, 1ONL, 1ONN, 1ONO, 1ONP, 1ONQ, 1ONR, 1ONS, 1ONW, 1ONX, 1ONY, 1ONZ, 1OO0, 1OO2, 1OO5, 1OO6, 1OO8, 1OOC, 1OOE, 1OOF, 1OOG, 1OOH, 1OOI, 1OOJ, 1OOK, 1OON, 1OOQ, 1OOT, 1OOW, 1OOY, 1OOZ, 1OP0, 1OP2, 1OP3, 1OP5, 1OP8, 1OP9, 1OPA, 1OPB, 1OPC, 1OPD, 1OPE, 1OPF, 1OPG, 1OPH, 1OPJ, 1OPK, 1OPL, 1OPM, 1OPR, 1OPS, 1OPX, 1OPY, 1OQ1, 1OQ4, 1OQ5, 1OQ7, 1OQ9, 1OQB, 1OQC, 1OQD, 1OQE, 1OQF, 1OQG, 1OQH, 1OQJ, 1OQL, 1OQM, 1OQN, 1OQO, 1OQQ, 1OQR, 1OQS, 1OQU, 1OQV, 1OQW, 1OQX, 1OR0, 1OR2, 1OR3, 1OR4, 1OR6, 1OR7, 1OR8, 1ORB, 1ORC, 1ORD, 1ORE, 1ORF, 1ORG, 1ORH, 1ORI, 1ORJ, 1ORK, 1ORN, 1ORO, 1ORP, 1ORQ, 1ORR, 1ORS, 1ORT, 1ORU, 1ORV, 1ORW, 1ORY, 1OS0, 1OS1, 1OS2, 1OS3, 1OS4, 1OS5, 1OS7, 1OS8, 1OS9, 1OSA, 1OSB, 1OSC, 1OSD, 1OSE, 1OSF, 1OSG, 1OSH, 1OSI, 1OSJ, 1OSM, 1OSN, 1OSP, 1OSS, 1OSV, 1OSY, 1OSZ, 1OT1, 1OT2, 1OT3, 1OT5, 1OT6, 1OT7, 1OT8, 1OT9, 1OTA, 1OTB, 1OTC, 1OTD, 1OTE, 1OTF, 1OTG, 1OTH, 1OTI, 1OTJ, 1OTK, 1OTM, 1OTN, 1OTO, 1OTP, 1OTS, 1OTT, 1OTU, 1OTV, 1OTW, 1OTX, 1OTY, 1OTZ, 1OU0, 1OU4, 1OU5, 1OU6, 1OU8, 1OU9, 1OUA, 1OUB, 1OUC, 1OUD, 1OUE, 1OUF, 1OUG, 1OUH, 1OUI, 1OUJ, 1OUK, 1OUL, 1OUM, 1OUN, 1OUO, 1OUP, 1OUQ, 1OUR, 1OUS, 1OUT, 1OUU, 1OUV, 1OUW, 1OUX, 1OUY, 1OUZ, 1OV3, 1OV4, 1OV5, 1OV6, 1OV7, 1OV8, 1OV9, 1OVA, 1OVB, 1OVD, 1OVE, 1OVG, 1OVH, 1OVJ, 1OVK, 1OVL, 1OVM, 1OVN, 1OVO, 1OVP, 1OVR, 1OVS, 1OVT, 1OVU, 1OVV, 1OVW, 1OVZ, 1OW0, 1OW1, 1OW2, 1OW3, 1OW4, 1OW6, 1OW7, 1OW8, 1OWB, 1OWC, 1OWD, 1OWE, 1OWF, 1OWG, 1OWH, 1OWI, 1OWJ, 1OWK, 1OWL, 1OWM, 1OWN, 1OWO, 1OWP, 1OWQ, 1OWR, 1OWS, 1OWY, 1OWZ, 1OX0, 1OX1, 1OX3, 1OX4, 1OX5, 1OX6, 1OX7, 1OX8, 1OX9, 1OXA, 1OXB, 1OXC, 1OXD, 1OXE, 1OXF, 1OXG, 1OXH, 1OXJ, 1OXK, 1OXL, 1OXM, 1OXN, 1OXO, 1OXP, 1OXQ, 1OXR, 1OXS, 1OXT, 1OXU, 1OXV, 1OXW, 1OXX, 1OXY, 1OXZ, 1OY0, 1OY1, 1OY3, 1OY5, 1OY6, 1OY7, 1OY8, 1OY9, 1OYA, 1OYB, 1OYC, 1OYD, 1OYE, 1OYF, 1OYG, 1OYH, 1OYJ, 1OYK, 1OYL, 1OYN, 1OYO, 1OYP, 1OYQ, 1OYR, 1OYS, 1OYT, 1OYU, 1OYV, 1OYW, 1OYX, 1OYY, 1OYZ, 1OZ0, 1OZ1, 1OZ2, 1OZ3, 1OZ6, 1OZ7, 1OZ9, 1OZA, 1OZB, 1OZE, 1OZF, 1OZG, 1OZH, 1OZJ, 1OZL, 1OZM, 1OZN, 1OZP, 1OZQ, 1OZR, 1OZT, 1OZU, 1OZV, 1OZW, 1OZY, 1P01, 1P02, 1P03, 1P04, 1P05, 1P06, 1P09, 1P0B, 1P0C, 1P0D, 1P0E, 1P0F, 1P0H, 1P0I, 1P0K, 1P0M, 1P0N, 1P0P, 1P0Q, 1P0S, 1P0T, 1P0V, 1P0W, 1P0X, 1P0Y, 1P0Z, 1P10, 1P11, 1P12, 1P13, 1P14, 1P15, 1P16, 1P17, 1P18, 1P19, 1P1B, 1P1C, 1P1F, 1P1G, 1P1H, 1P1I, 1P1J, 1P1K, 1P1L, 1P1M, 1P1N, 1P1O, 1P1Q, 1P1R, 1P1U, 1P1V, 1P1W, 1P1X, 1P1Y, 1P1Z, 1P22, 1P24, 1P25, 1P26, 1P27, 1P28, 1P29, 1P2A, 1P2B, 1P2C, 1P2D, 1P2E, 1P2F, 1P2G, 1P2H, 1P2I, 1P2J, 1P2K, 1P2L, 1P2M, 1P2N, 1P2O, 1P2P, 1P2Q, 1P2R, 1P2S, 1P2T, 1P2U, 1P2V, 1P2X, 1P2Y, 1P2Z, 1P30, 1P31, 1P32, 1P33, 1P34, 1P35, 1P36, 1P37, 1P38, 1P39, 1P3A, 1P3B, 1P3C, 1P3D, 1P3E, 1P3F, 1P3G, 1P3H, 1P3I, 1P3J, 1P3K, 1P3L, 1P3M, 1P3N, 1P3O, 1P3P, 1P3Q, 1P3R, 1P3T, 1P3U, 1P3V, 1P3W, 1P3Y, 1P42, 1P43, 1P44, 1P45, 1P46, 1P47, 1P48, 1P49, 1P4A, 1P4B, 1P4C, 1P4D, 1P4E, 1P4F, 1P4G, 1P4H, 1P4I, 1P4J, 1P4K, 1P4L, 1P4M, 1P4N, 1P4O, 1P4P, 1P4R, 1P4T, 1P4U, 1P4V, 1P4X, 1P4Y, 1P4Z, 1P50, 1P51, 1P52, 1P53, 1P54, 1P56, 1P57, 1P59, 1P5B, 1P5C, 1P5D, 1P5E, 1P5F, 1P5G, 1P5H, 1P5J, 1P5Q, 1P5R, 1P5S, 1P5T, 1P5U, 1P5V, 1P5X, 1P5Z, 1P60, 1P61, 1P62, 1P63, 1P64, 1P65, 1P69, 1P6A, 1P6B, 1P6C, 1P6D, 1P6E, 1P6F, 1P6H, 1P6I, 1P6J, 1P6K, 1P6L, 1P6M, 1P6N, 1P6O, 1P6P, 1P6W, 1P6X, 1P6Y, 1P71, 1P72, 1P73, 1P74, 1P75, 1P77, 1P78, 1P7B, 1P7C, 1P7D, 1P7G, 1P7H, 1P7I, 1P7J, 1P7K, 1P7L, 1P7N, 1P7O, 1P7P, 1P7Q, 1P7R, 1P7S, 1P7T, 1P7V, 1P7W, 1P7Y, 1P7Z, 1P80, 1P81, 1P84, 1P8C, 1P8D, 1P8F, 1P8H, 1P8I, 1P8J, 1P8K, 1P8L, 1P8M, 1P8N, 1P8O, 1P8P, 1P8Q, 1P8R, 1P8S, 1P8T, 1P8U, 1P8V, 1P8X, 1P8Z, 1P90, 1P91, 1P92, 1P93, 1P99, 1P9A, 1P9B, 1P9E, 1P9G, 1P9H, 1P9I, 1P9L, 1P9M, 1P9N, 1P9O, 1P9P, 1P9Q, 1P9R, 1P9S, 1P9U, 1P9W, 1P9Y, 1PA0, 1PA1, 1PA2, 1PA3, 1PA6, 1PA7, 1PA9, 1PAD, 1PAE, 1PAF, 1PAG, 1PAH, 1PAL, 1PAM, 1PAQ, 1PAR, 1PAU, 1PAX, 1PAZ, 1PB0, 1PB1, 1PB3, 1PB7, 1PB8, 1PB9, 1PBB, 1PBC, 1PBD, 1PBE, 1PBF, 1PBG, 1PBH, 1PBI, 1PBJ, 1PBK, 1PBN, 1PBO, 1PBP, 1PBQ, 1PBT, 1PBV, 1PBW, 1PBX, 1PBY, 1PC3, 1PC4, 1PC5, 1PC6, 1PC8, 1PC9, 1PCA, 1PCF, 1PCG, 1PCH, 1PCI, 1PCJ, 1PCK, 1PCL, 1PCM, 1PCQ, 1PCR, 1PCS, 1PCV, 1PCW, 1PCX, 1PCZ, 1PD0, 1PD1, 1PD2, 1PD3, 1PD5, 1PD8, 1PD9, 1PDA, 1PDB, 1PDG, 1PDH, 1PDK, 1PDN, 1PDO, 1PDQ, 1PDR, 1PDU, 1PDV, 1PDW, 1PDY, 1PDZ, 1PE0, 1PE1, 1PE5, 1PE6, 1PE7, 1PE8, 1PE9, 1PEA, 1PEB, 1PED, 1PEE, 1PEF, 1PEG, 1PEK, 1PEM, 1PEN, 1PEO, 1PEQ, 1PER, 1PEU, 1PEV, 1PEW, 1PEX, 1PEY, 1PEZ, 1PF3, 1PF5, 1PF7, 1PF8, 1PF9, 1PFB, 1PFC, 1PFF, 1PFG, 1PFK, 1PFO, 1PFP, 1PFQ, 1PFR, 1PFU, 1PFV, 1PFW, 1PFX, 1PFY, 1PFZ, 1PG0, 1PG2, 1PG3, 1PG4, 1PG5, 1PG6, 1PG7, 1PG8, 1PGA, 1PGB, 1PGE, 1PGF, 1PGG, 1PGI, 1PGJ, 1PGN, 1PGO, 1PGP, 1PGQ, 1PGR, 1PGS, 1PGT, 1PGU, 1PGV, 1PGX, 1PGZ, 1PH0, 1PH1, 1PH2, 1PH3, 1PH4, 1PH5, 1PH6, 1PH7, 1PH8, 1PH9, 1PHA, 1PHB, 1PHC, 1PHD, 1PHE, 1PHF, 1PHG, 1PHH, 1PHJ, 1PHK, 1PHM, 1PHN, 1PHO, 1PHP, 1PHQ, 1PHR, 1PHS, 1PHT, 1PHW, 1PHZ, 1PI1, 1PI2, 1PI3, 1PI4, 1PI5, 1PI6, 1PID, 1PIE, 1PIF, 1PIG, 1PII, 1PIL, 1PIM, 1PIN, 1PIO, 1PIP, 1PIQ, 1PIU, 1PIW, 1PIX, 1PIY, 1PIZ, 1PJ0, 1PJ1, 1PJ2, 1PJ3, 1PJ4, 1PJ5, 1PJ6, 1PJ7, 1PJ8, 1PJ9, 1PJA, 1PJB, 1PJC, 1PJH, 1PJI, 1PJJ, 1PJK, 1PJL, 1PJM, 1PJN, 1PJP, 1PJQ, 1PJR, 1PJS, 1PJT, 1PJU, 1PJX, 1PK0, 1PK1, 1PK3, 1PK4, 1PK5, 1PK6, 1PK7, 1PK8, 1PK9, 1PKD, 1PKE, 1PKF, 1PKG, 1PKH, 1PKJ, 1PKK, 1PKL, 1PKM, 1PKN, 1PKO, 1PKP, 1PKQ, 1PKR, 1PKU, 1PKV, 1PKW, 1PKX, 1PKY, 1PKZ, 1PL0, 1PL1, 1PL2, 1PL3, 1PL4, 1PL5, 1PL6, 1PL7, 1PL8, 1PL9, 1PLC, 1PLF, 1PLG, 1PLJ, 1PLK, 1PLL, 1PLQ, 1PLR, 1PLU, 1PM1, 1PM2, 1PM3, 1PM4, 1PM5, 1PM7, 1PM9, 1PMA, 1PMB, 1PMD, 1PME, 1PMH, 1PMI, 1PMJ, 1PMK, 1PML, 1PMM, 1PMN, 1PMO, 1PMP, 1PMQ, 1PMT, 1PMU, 1PMV, 1PMY, 1PN0, 1PN2, 1PN3, 1PN4, 1PN9, 1PNC, 1PND, 1PNE, 1PNF, 1PNG, 1PNK, 1PNL, 1PNM, 1PNO, 1PNQ, 1PNR, 1PNT, 1PNV, 1PNZ, 1PO0, 1PO3, 1PO5, 1PO6, 1PO7, 1PO8, 1PO9, 1POA, 1POB, 1POC, 1POD, 1POE, 1POH, 1POI, 1POJ, 1POK, 1POO, 1POP, 1POS, 1POT, 1POW, 1POX, 1POY, 1PP0, 1PP1, 1PP2, 1PP3, 1PP4, 1PP6, 1PP7, 1PP8, 1PP9, 1PPA, 1PPB, 1PPC, 1PPD, 1PPE, 1PPF, 1PPG, 1PPH, 1PPI, 1PPJ, 1PPK, 1PPL, 1PPM, 1PPN, 1PPO, 1PPP, 1PPT, 1PPV, 1PPW, 1PPY, 1PPZ, 1PQ0, 1PQ1, 1PQ2, 1PQ3, 1PQ4, 1PQ5, 1PQ6, 1PQ7, 1PQ8, 1PQ9, 1PQA, 1PQC, 1PQD, 1PQE, 1PQF, 1PQH, 1PQI, 1PQJ, 1PQK, 1PQM, 1PQO, 1PQP, 1PQU, 1PQV, 1PQW, 1PQY, 1PQZ, 1PR0, 1PR1, 1PR2, 1PR3, 1PR4, 1PR5, 1PR6, 1PR9, 1PRC, 1PRE, 1PRG, 1PRH, 1PRN, 1PRO, 1PRP, 1PRQ, 1PRT, 1PRW, 1PRX, 1PRY, 1PRZ, 1PS0, 1PS1, 1PS3, 1PS5, 1PS6, 1PS7, 1PS8, 1PS9, 1PSA, 1PSC, 1PSD, 1PSH, 1PSI, 1PSJ, 1PSK, 1PSN, 1PSO, 1PSP, 1PSQ, 1PSR, 1PSS, 1PST, 1PSU, 1PSW, 1PSZ, 1PT0, 1PT1, 1PT2, 1PT3, 1PT5, 1PT6, 1PT7, 1PT8, 1PT9, 1PTA, 1PTD, 1PTF, 1PTG, 1PTH, 1PTJ, 1PTK, 1PTM, 1PTO, 1PTQ, 1PTR, 1PTS, 1PTT, 1PTU, 1PTV, 1PTW, 1PTX, 1PTY, 1PTZ, 1PU0, 1PU2, 1PU4, 1PU5, 1PU6, 1PU7, 1PU8, 1PU9, 1PUA, 1PUB, 1PUC, 1PUD, 1PUE, 1PUF, 1PUG, 1PUI, 1PUJ, 1PUM, 1PUO, 1PUU, 1PUY, 1PV1, 1PV2, 1PV4, 1PV5, 1PV6, 1PV7, 1PV8, 1PV9, 1PVA, 1PVB, 1PVD, 1PVF, 1PVG, 1PVH, 1PVI, 1PVJ, 1PVL, 1PVM, 1PVN, 1PVP, 1PVQ, 1PVR, 1PVS, 1PVT, 1PVU, 1PVV, 1PVW, 1PVX, 1PVY, 1PW1, 1PW2, 1PW3, 1PW4, 1PW5, 1PW6, 1PW7, 1PW8, 1PW9, 1PWA, 1PWB, 1PWC, 1PWD, 1PWE, 1PWG, 1PWH, 1PWL, 1PWM, 1PWO, 1PWP, 1PWQ, 1PWT, 1PWU, 1PWV, 1PWW, 1PWX, 1PWY, 1PWZ, 1PX0, 1PX2, 1PX3, 1PX4, 1PX5, 1PX6, 1PX7, 1PX8, 1PXA, 1PXB, 1PXC, 1PXD, 1PXG, 1PXH, 1PXI, 1PXJ, 1PXK, 1PXL, 1PXM, 1PXN, 1PXO, 1PXP, 1PXR, 1PXS, 1PXT, 1PXU, 1PXV, 1PXW, 1PXX, 1PXY, 1PXZ, 1PY0, 1PY1, 1PY2, 1PY3, 1PY4, 1PY5, 1PY6, 1PY9, 1PYA, 1PYB, 1PYD, 1PYE, 1PYF, 1PYG, 1PYH, 1PYI, 1PYK, 1PYL, 1PYM, 1PYN, 1PYO, 1PYP, 1PYQ, 1PYS, 1PYT, 1PYU, 1PYW, 1PYX, 1PYY, 1PZ0, 1PZ1, 1PZ2, 1PZ3, 1PZ4, 1PZ5, 1PZ7, 1PZ8, 1PZ9, 1PZA, 1PZB, 1PZC, 1PZD, 1PZE, 1PZF, 1PZG, 1PZH, 1PZI, 1PZJ, 1PZK, 1PZL, 1PZM, 1PZN, 1PZO, 1PZP, 1PZS, 1PZT, 1PZU, 1PZV, 1PZW, 1PZX, 1PZY, 1PZZ, 1Q03, 1Q04, 1Q05, 1Q06, 1Q07, 1Q08, 1Q09, 1Q0A, 1Q0B, 1Q0C, 1Q0D, 1Q0E, 1Q0F, 1Q0G, 1Q0H, 1Q0K, 1Q0L, 1Q0M, 1Q0N, 1Q0O, 1Q0P, 1Q0Q, 1Q0R, 1Q0S, 1Q0T, 1Q0U, 1Q0X, 1Q0Y, 1Q0Z, 1Q11, 1Q12, 1Q13, 1Q14, 1Q15, 1Q16, 1Q17, 1Q18, 1Q19, 1Q1A, 1Q1B, 1Q1C, 1Q1E, 1Q1F, 1Q1G, 1Q1H, 1Q1J, 1Q1K, 1Q1L, 1Q1M, 1Q1N, 1Q1P, 1Q1Q, 1Q1R, 1Q1S, 1Q1T, 1Q1U, 1Q1W, 1Q1Y, 1Q1Z, 1Q20, 1Q21, 1Q22, 1Q23, 1Q24, 1Q25, 1Q2B, 1Q2C, 1Q2D, 1Q2E, 1Q2H, 1Q2L, 1Q2O, 1Q2P, 1Q2Q, 1Q2U, 1Q2V, 1Q2W, 1Q2X, 1Q2Y, 1Q31, 1Q32, 1Q33, 1Q34, 1Q35, 1Q36, 1Q39, 1Q3A, 1Q3B, 1Q3C, 1Q3D, 1Q3E, 1Q3F, 1Q3G, 1Q3H, 1Q3I, 1Q3K, 1Q3L, 1Q3N, 1Q3O, 1Q3P, 1Q3Q, 1Q3R, 1Q3S, 1Q3U, 1Q3V, 1Q3W, 1Q3X, 1Q40, 1Q41, 1Q42, 1Q43, 1Q44, 1Q45, 1Q46, 1Q47, 1Q4A, 1Q4B, 1Q4C, 1Q4D, 1Q4E, 1Q4G, 1Q4J, 1Q4K, 1Q4L, 1Q4N, 1Q4O, 1Q4Q, 1Q4R, 1Q4S, 1Q4T, 1Q4U, 1Q4V, 1Q4W, 1Q4X, 1Q50, 1Q51, 1Q52, 1Q54, 1Q57, 1Q5D, 1Q5E, 1Q5H, 1Q5I, 1Q5J, 1Q5K, 1Q5M, 1Q5N, 1Q5O, 1Q5P, 1Q5Q, 1Q5R, 1Q5T, 1Q5U, 1Q5V, 1Q5X, 1Q5Y, 1Q5Z, 1Q61, 1Q62, 1Q63, 1Q65, 1Q66, 1Q67, 1Q6C, 1Q6D, 1Q6E, 1Q6F, 1Q6G, 1Q6H, 1Q6I, 1Q6J, 1Q6K, 1Q6L, 1Q6M, 1Q6N, 1Q6O, 1Q6P, 1Q6Q, 1Q6R, 1Q6S, 1Q6T, 1Q6U, 1Q6V, 1Q6W, 1Q6X, 1Q6Y, 1Q6Z, 1Q72, 1Q73, 1Q74, 1Q77, 1Q78, 1Q79, 1Q7A, 1Q7B, 1Q7C, 1Q7E, 1Q7F, 1Q7G, 1Q7H, 1Q7L, 1Q7M, 1Q7Q, 1Q7R, 1Q7S, 1Q7T, 1Q7Z, 1Q83, 1Q84, 1Q85, 1Q87, 1Q88, 1Q89, 1Q8A, 1Q8B, 1Q8C, 1Q8D, 1Q8F, 1Q8H, 1Q8I, 1Q8J, 1Q8M, 1Q8O, 1Q8P, 1Q8Q, 1Q8R, 1Q8S, 1Q8T, 1Q8U, 1Q8V, 1Q8W, 1Q8Y, 1Q8Z, 1Q90, 1Q91, 1Q92, 1Q94, 1Q95, 1Q97, 1Q98, 1Q99, 1Q9B, 1Q9C, 1Q9D, 1Q9E, 1Q9H, 1Q9I, 1Q9J, 1Q9K, 1Q9L, 1Q9M, 1Q9O, 1Q9S, 1Q9U, 1Q9W, 1Q9X, 1Q9Y, 1QA0, 1QA1, 1QA2, 1QA3, 1QA7, 1QA9, 1QAB, 1QAC, 1QAD, 1QAE, 1QAF, 1QAG, 1QAH, 1QAI, 1QAJ, 1QAK, 1QAL, 1QAM, 1QAN, 1QAO, 1QAP, 1QAQ, 1QAS, 1QAT, 1QAU, 1QAV, 1QAW, 1QAX, 1QAY, 1QAZ, 1QB0, 1QB1, 1QB2, 1QB3, 1QB4, 1QB5, 1QB6, 1QB7, 1QB8, 1QB9, 1QBA, 1QBB, 1QBE, 1QBG, 1QBI, 1QBJ, 1QBK, 1QBL, 1QBM, 1QBN, 1QBO, 1QBQ, 1QBR, 1QBS, 1QBT, 1QBU, 1QBV, 1QBZ, 1QC1, 1QC5, 1QC6, 1QC7, 1QC9, 1QCA, 1QCB, 1QCC, 1QCD, 1QCF, 1QCG, 1QCI, 1QCJ, 1QCN, 1QCO, 1QCP, 1QCQ, 1QCR, 1QCS, 1QCW, 1QCX, 1QCY, 1QCZ, 1QD0, 1QD1, 1QD2, 1QD5, 1QD6, 1QD7, 1QD9, 1QDB, 1QDC, 1QDD, 1QDE, 1QDL, 1QDM, 1QDN, 1QDO, 1QDQ, 1QDR, 1QDS, 1QDT, 1QDU, 1QDV, 1QDW, 1QE0, 1QE1, 1QE3, 1QE5, 1QE6, 1QEW, 1QEX, 1QEZ, 1QF0, 1QF1, 1QF2, 1QF3, 1QF4, 1QF5, 1QF7, 1QF8, 1QF9, 1QFC, 1QFE, 1QFF, 1QFG, 1QFH, 1QFJ, 1QFK, 1QFL, 1QFM, 1QFO, 1QFP, 1QFS, 1QFT, 1QFU, 1QFV, 1QFW, 1QFX, 1QFY, 1QFZ, 1QG0, 1QG2, 1QG3, 1QG4, 1QG5, 1QG6, 1QG7, 1QG8, 1QGA, 1QGD, 1QGE, 1QGF, 1QGH, 1QGI, 1QGJ, 1QGK, 1QGL, 1QGN, 1QGO, 1QGQ, 1QGR, 1QGS, 1QGU, 1QGV, 1QGW, 1QGX, 1QGY, 1QGZ, 1QH0, 1QH1, 1QH3, 1QH4, 1QH5, 1QH6, 1QH7, 1QH8, 1QH9, 1QHA, 1QHB, 1QHC, 1QHD, 1QHE, 1QHF, 1QHG, 1QHH, 1QHI, 1QHJ, 1QHL, 1QHM, 1QHN, 1QHO, 1QHP, 1QHQ, 1QHR, 1QHS, 1QHT, 1QHU, 1QHV, 1QHW, 1QHX, 1QHY, 1QHZ, 1QI0, 1QI1, 1QI2, 1QI3, 1QI4, 1QI5, 1QI6, 1QI7, 1QI8, 1QI9, 1QIA, 1QIB, 1QIC, 1QID, 1QIE, 1QIF, 1QIG, 1QIH, 1QII, 1QIJ, 1QIK, 1QIL, 1QIM, 1QIN, 1QIO, 1QIP, 1QIQ, 1QIR, 1QIS, 1QIT, 1QIU, 1QIV, 1QIW, 1QIX, 1QIY, 1QIZ, 1QJ0, 1QJ1, 1QJ3, 1QJ4, 1QJ5, 1QJ6, 1QJ7, 1QJ8, 1QJ9, 1QJA, 1QJB, 1QJC, 1QJD, 1QJE, 1QJF, 1QJG, 1QJH, 1QJI, 1QJJ, 1QJM, 1QJP, 1QJQ, 1QJS, 1QJV, 1QJW, 1QK0, 1QK1, 1QK2, 1QK3, 1QK4, 1QK5, 1QK8, 1QKA, 1QKB, 1QKC, 1QKD, 1QKE, 1QKI, 1QKJ, 1QKK, 1QKM, 1QKN, 1QKO, 1QKP, 1QKQ, 1QKR, 1QKS, 1QKT, 1QKU, 1QKW, 1QKX, 1QKZ, 1QL0, 1QL3, 1QL4, 1QL6, 1QL7, 1QL8, 1QL9, 1QLB, 1QLE, 1QLF, 1QLG, 1QLH, 1QLJ, 1QLL, 1QLM, 1QLP, 1QLQ, 1QLR, 1QLS, 1QLT, 1QLU, 1QLV, 1QLW, 1QM4, 1QM5, 1QM6, 1QM7, 1QM8, 1QMA, 1QMB, 1QMD, 1QME, 1QMF, 1QMG, 1QMH, 1QMI, 1QMJ, 1QML, 1QMN, 1QMO, 1QMP, 1QMQ, 1QMR, 1QMT, 1QMU, 1QMV, 1QMY, 1QMZ, 1QN2, 1QN3, 1QN4, 1QN5, 1QN6, 1QN7, 1QN8, 1QN9, 1QNA, 1QNB, 1QNC, 1QNE, 1QNF, 1QNG, 1QNH, 1QNI, 1QNJ, 1QNL, 1QNM, 1QNN, 1QNO, 1QNP, 1QNQ, 1QNR, 1QNS, 1QNT, 1QNU, 1QNV, 1QNW, 1QNX, 1QNY, 1QO0, 1QO1, 1QO2, 1QO3, 1QO4, 1QO5, 1QO7, 1QO8, 1QO9, 1QOA, 1QOB, 1QOF, 1QOG, 1QOH, 1QOI, 1QOJ, 1QOK, 1QOL, 1QOM, 1QON, 1QOO, 1QOP, 1QOQ, 1QOR, 1QOS, 1QOT, 1QOU, 1QOV, 1QOX, 1QOY, 1QOZ, 1QP0, 1QP1, 1QP4, 1QP5, 1QP7, 1QP8, 1QP9, 1QPA, 1QPB, 1QPC, 1QPD, 1QPE, 1QPF, 1QPG, 1QPH, 1QPI, 1QPJ, 1QPK, 1QPL, 1QPN, 1QPO, 1QPP, 1QPQ, 1QPR, 1QPS, 1QPV, 1QPW, 1QPX, 1QPZ, 1QQ0, 1QQ1, 1QQ2, 1QQ4, 1QQ5, 1QQ6, 1QQ7, 1QQ9, 1QQA, 1QQB, 1QQC, 1QQD, 1QQE, 1QQF, 1QQG, 1QQH, 1QQJ, 1QQK, 1QQL, 1QQM, 1QQN, 1QQO, 1QQQ, 1QQR, 1QQS, 1QQT, 1QQU, 1QQW, 1QQY, 1QR0, 1QR1, 1QR2, 1QR3, 1QR4, 1QR6, 1QR7, 1QR8, 1QR9, 1QRA, 1QRB, 1QRC, 1QRD, 1QRE, 1QRF, 1QRG, 1QRH, 1QRI, 1QRK, 1QRL, 1QRM, 1QRN, 1QRP, 1QRQ, 1QRR, 1QRV, 1QRW, 1QRX, 1QRZ, 1QS0, 1QS1, 1QS2, 1QS4, 1QS5, 1QS7, 1QS8, 1QS9, 1QSA, 1QSB, 1QSC, 1QSD, 1QSE, 1QSF, 1QSG, 1QSH, 1QSI, 1QSJ, 1QSL, 1QSM, 1QSN, 1QSO, 1QSP, 1QSQ, 1QSR, 1QSS, 1QST, 1QSW, 1QSY, 1QT1, 1QT3, 1QT4, 1QT5, 1QT6, 1QT7, 1QT8, 1QT9, 1QTB, 1QTC, 1QTD, 1QTE, 1QTF, 1QTH, 1QTI, 1QTJ, 1QTK, 1QTM, 1QTN, 1QTO, 1QTP, 1QTR, 1QTS, 1QTV, 1QTW, 1QTX, 1QTY, 1QTZ, 1QU0, 1QU1, 1QU4, 1QU7, 1QU9, 1QUA, 1QUB, 1QUD, 1QUE, 1QUF, 1QUG, 1QUH, 1QUI, 1QUJ, 1QUK, 1QUL, 1QUM, 1QUN, 1QUO, 1QUP, 1QUQ, 1QUR, 1QUS, 1QUT, 1QUU, 1QUV, 1QV0, 1QV1, 1QV4, 1QV6, 1QV7, 1QV8, 1QV9, 1QVA, 1QVB, 1QVC, 1QVE, 1QVI, 1QVJ, 1QVN, 1QVO, 1QVR, 1QVS, 1QVT, 1QVU, 1QVV, 1QVW, 1QVY, 1QVZ, 1QW0, 1QW2, 1QW4, 1QW5, 1QW6, 1QW7, 1QW8, 1QW9, 1QWC, 1QWD, 1QWG, 1QWH, 1QWI, 1QWJ, 1QWK, 1QWL, 1QWM, 1QWN, 1QWO, 1QWR, 1QWS, 1QWT, 1QWU, 1QWX, 1QWY, 1QWZ, 1QX0, 1QX1, 1QX2, 1QX3, 1QX4, 1QX5, 1QX6, 1QX7, 1QX8, 1QXA, 1QXD, 1QXE, 1QXH, 1QXJ, 1QXK, 1QXL, 1QXM, 1QXO, 1QXP, 1QXR, 1QXS, 1QXT, 1QXW, 1QXX, 1QXY, 1QXZ, 1QY0, 1QY1, 1QY2, 1QY3, 1QY4, 1QY5, 1QY6, 1QY7, 1QY8, 1QY9, 1QYA, 1QYB, 1QYC, 1QYD, 1QYE, 1QYF, 1QYG, 1QYI, 1QYK, 1QYL, 1QYM, 1QYN, 1QYO, 1QYQ, 1QYR, 1QYS, 1QYU, 1QYV, 1QYW, 1QYX, 1QYY, 1QYZ, 1QZ0, 1QZ1, 1QZ2, 1QZ3, 1QZ4, 1QZ5, 1QZ6, 1QZ7, 1QZ8, 1QZ9, 1QZF, 1QZG, 1QZH, 1QZL, 1QZM, 1QZN, 1QZQ, 1QZR, 1QZT, 1QZU, 1QZX, 1QZY, 1QZZ, 1R00, 1R03, 1R0A, 1R0B, 1R0C, 1R0D, 1R0E, 1R0F, 1R0G, 1R0H, 1R0I, 1R0J, 1R0K, 1R0L, 1R0M, 1R0N, 1R0O, 1R0P, 1R0Q, 1R0R, 1R0S, 1R0U, 1R0V, 1R0W, 1R0X, 1R0Y, 1R0Z, 1R10, 1R11, 1R12, 1R13, 1R14, 1R15, 1R16, 1R17, 1R18, 1R19, 1R1C, 1R1D, 1R1H, 1R1I, 1R1J, 1R1K, 1R1L, 1R1M, 1R1N, 1R1O, 1R1P, 1R1Q, 1R1R, 1R1S, 1R1T, 1R1U, 1R1V, 1R1W, 1R1X, 1R1Y, 1R1Z, 1R20, 1R22, 1R23, 1R24, 1R26, 1R27, 1R28, 1R29, 1R2B, 1R2C, 1R2D, 1R2E, 1R2F, 1R2G, 1R2H, 1R2I, 1R2J, 1R2K, 1R2M, 1R2O, 1R2Q, 1R2R, 1R2S, 1R2T, 1R2Y, 1R2Z, 1R30, 1R31, 1R33, 1R34, 1R35, 1R37, 1R38, 1R39, 1R3C, 1R3D, 1R3F, 1R3G, 1R3H, 1R3I, 1R3J, 1R3K, 1R3L, 1R3M, 1R3N, 1R3Q, 1R3R, 1R3S, 1R3T, 1R3U, 1R3V, 1R3W, 1R3Y, 1R42, 1R43, 1R44, 1R45, 1R46, 1R47, 1R49, 1R4A, 1R4B, 1R4C, 1R4F, 1R4I, 1R4L, 1R4M, 1R4N, 1R4O, 1R4P, 1R4Q, 1R4R, 1R4S, 1R4U, 1R4V, 1R4W, 1R4X, 1R4Z, 1R50, 1R51, 1R52, 1R53, 1R54, 1R55, 1R56, 1R58, 1R59, 1R5A, 1R5B, 1R5C, 1R5D, 1R5G, 1R5H, 1R5I, 1R5J, 1R5K, 1R5L, 1R5M, 1R5N, 1R5O, 1R5P, 1R5Q, 1R5T, 1R5U, 1R5V, 1R5W, 1R5X, 1R5Y, 1R5Z, 1R61, 1R62, 1R64, 1R65, 1R66, 1R67, 1R69, 1R6A, 1R6B, 1R6C, 1R6D, 1R6F, 1R6G, 1R6J, 1R6K, 1R6L, 1R6M, 1R6N, 1R6O, 1R6Q, 1R6T, 1R6U, 1R6V, 1R6W, 1R6X, 1R6Y, 1R6Z, 1R71, 1R74, 1R75, 1R76, 1R77, 1R78, 1R7A, 1R7H, 1R7I, 1R7J, 1R7L, 1R7M, 1R7O, 1R7R, 1R7S, 1R7T, 1R7U, 1R7V, 1R7X, 1R7Y, 1R80, 1R81, 1R82, 1R85, 1R86, 1R87, 1R88, 1R89, 1R8A, 1R8B, 1R8C, 1R8D, 1R8E, 1R8G, 1R8H, 1R8I, 1R8J, 1R8K, 1R8L, 1R8M, 1R8N, 1R8O, 1R8Q, 1R8S, 1R8W, 1R8X, 1R8Y, 1R94, 1R95, 1R9C, 1R9D, 1R9G, 1R9H, 1R9J, 1R9L, 1R9M, 1R9N, 1R9O, 1R9Q, 1R9W, 1R9X, 1R9Y, 1R9Z, 1RA0, 1RA1, 1RA2, 1RA3, 1RA4, 1RA5, 1RA6, 1RA7, 1RA8, 1RA9, 1RAA, 1RAB, 1RAC, 1RAD, 1RAE, 1RAF, 1RAG, 1RAH, 1RAI, 1RAJ, 1RAK, 1RAL, 1RAM, 1RAO, 1RAP, 1RAQ, 1RAR, 1RAS, 1RAT, 1RAV, 1RAY, 1RAZ, 1RB0, 1RB2, 1RB3, 1RB4, 1RB5, 1RB6, 1RB7, 1RB9, 1RBA, 1RBB, 1RBC, 1RBD, 1RBE, 1RBF, 1RBG, 1RBH, 1RBI, 1RBJ, 1RBL, 1RBM, 1RBN, 1RBO, 1RBP, 1RBQ, 1RBR, 1RBS, 1RBT, 1RBU, 1RBV, 1RBW, 1RBX, 1RBY, 1RBZ, 1RC0, 1RC1, 1RC2, 1RC4, 1RC5, 1RC6, 1RC8, 1RC9, 1RCA, 1RCB, 1RCC, 1RCD, 1RCE, 1RCF, 1RCG, 1RCI, 1RCJ, 1RCM, 1RCN, 1RCO, 1RCP, 1RCQ, 1RCT, 1RCU, 1RCV, 1RCW, 1RCX, 1RCY, 1RD3, 1RD4, 1RD5, 1RD6, 1RD7, 1RD8, 1RD9, 1RDA, 1RDB, 1RDC, 1RDD, 1RDF, 1RDG, 1RDH, 1RDI, 1RDJ, 1RDK, 1RDL, 1RDM, 1RDN, 1RDO, 1RDP, 1RDQ, 1RDR, 1RDS, 1RDT, 1RDV, 1RDW, 1RDX, 1RDY, 1RDZ, 1RE0, 1RE1, 1RE2, 1RE3, 1RE4, 1RE5, 1RE7, 1RE8, 1RE9, 1REA, 1REC, 1RED, 1REE, 1REF, 1REG, 1REI, 1REJ, 1REK, 1REM, 1REO, 1REP, 1REQ, 1RER, 1REU, 1REV, 1REW, 1REX, 1REY, 1REZ, 1RF0, 1RF1, 1RF2, 1RF3, 1RF4, 1RF5, 1RF6, 1RF7, 1RF9, 1RFB, 1RFD, 1RFE, 1RFF, 1RFG, 1RFI, 1RFJ, 1RFK, 1RFN, 1RFP, 1RFQ, 1RFS, 1RFT, 1RFU, 1RFV, 1RFX, 1RFY, 1RFZ, 1RG0, 1RG1, 1RG2, 1RG5, 1RG7, 1RG8, 1RG9, 1RGA, 1RGB, 1RGC, 1RGE, 1RGF, 1RGG, 1RGH, 1RGI, 1RGK, 1RGL, 1RGN, 1RGP, 1RGQ, 1RGS, 1RGT, 1RGU, 1RGV, 1RGX, 1RGY, 1RGZ, 1RH0, 1RH1, 1RH2, 1RH3, 1RH4, 1RH5, 1RH6, 1RH7, 1RH9, 1RHA, 1RHB, 1RHC, 1RHD, 1RHF, 1RHG, 1RHH, 1RHJ, 1RHK, 1RHL, 1RHM, 1RHO, 1RHP, 1RHQ, 1RHR, 1RHS, 1RHU, 1RHY, 1RHZ, 1RI1, 1RI2, 1RI3, 1RI4, 1RI5, 1RI6, 1RI7, 1RI8, 1RIB, 1RID, 1RIE, 1RIF, 1RIH, 1RII, 1RIL, 1RIN, 1RIO, 1RIQ, 1RIR, 1RIS, 1RIT, 1RIU, 1RIV, 1RIW, 1RIY, 1RJ1, 1RJ2, 1RJ4, 1RJ5, 1RJ6, 1RJ7, 1RJ8, 1RJ9, 1RJB, 1RJC, 1RJD, 1RJE, 1RJF, 1RJG, 1RJK, 1RJL, 1RJM, 1RJN, 1RJO, 1RJP, 1RJQ, 1RJR, 1RJU, 1RJW, 1RJX, 1RJY, 1RJZ, 1RK0, 1RK1, 1RK2, 1RK3, 1RK4, 1RK5, 1RK6, 1RK8, 1RKA, 1RKB, 1RKC, 1RKD, 1RKE, 1RKG, 1RKH, 1RKI, 1RKM, 1RKP, 1RKQ, 1RKR, 1RKS, 1RKT, 1RKU, 1RKV, 1RKW, 1RKX, 1RKY, 1RL0, 1RL2, 1RL3, 1RL4, 1RL6, 1RL8, 1RL9, 1RLA, 1RLB, 1RLC, 1RLD, 1RLH, 1RLI, 1RLJ, 1RLK, 1RLM, 1RLO, 1RLR, 1RLS, 1RLT, 1RLU, 1RLV, 1RLW, 1RLZ, 1RM0, 1RM1, 1RM3, 1RM4, 1RM5, 1RM6, 1RM8, 1RM9, 1RMD, 1RMF, 1RMG, 1RMH, 1RMM, 1RMO, 1RMP, 1RMQ, 1RMR, 1RMS, 1RMT, 1RMY, 1RMZ, 1RN1, 1RN4, 1RN7, 1RN8, 1RNB, 1RNC, 1RND, 1RNE, 1RNF, 1RNH, 1RNI, 1RNJ, 1RNL, 1RNM, 1RNN, 1RNO, 1RNQ, 1RNR, 1RNT, 1RNU, 1RNV, 1RNW, 1RNX, 1RNY, 1RNZ, 1RO0, 1RO2, 1RO5, 1RO6, 1RO7, 1RO8, 1RO9, 1ROA, 1ROB, 1ROC, 1ROM, 1ROP, 1ROR, 1ROS, 1ROV, 1ROW, 1ROZ, 1RP0, 1RP1, 1RP3, 1RP4, 1RP5, 1RP7, 1RP8, 1RP9, 1RPA, 1RPE, 1RPF, 1RPG, 1RPH, 1RPI, 1RPJ, 1RPK, 1RPL, 1RPM, 1RPN, 1RPO, 1RPQ, 1RPS, 1RPT, 1RPW, 1RPX, 1RPY, 1RPZ, 1RQ0, 1RQ1, 1RQ2, 1RQ3, 1RQ4, 1RQ5, 1RQ7, 1RQ9, 1RQA, 1RQB, 1RQC, 1RQD, 1RQE, 1RQF, 1RQG, 1RQH, 1RQI, 1RQJ, 1RQK, 1RQL, 1RQN, 1RQP, 1RQQ, 1RQR, 1RQW, 1RQX, 1RQY, 1RR2, 1RR6, 1RR7, 1RR8, 1RR9, 1RRA, 1RRC, 1RRE, 1RRF, 1RRG, 1RRH, 1RRI, 1RRJ, 1RRK, 1RRL, 1RRM, 1RRO, 1RRP, 1RRQ, 1RRS, 1RRV, 1RRW, 1RRX, 1RRY, 1RS0, 1RS2, 1RS4, 1RS6, 1RS7, 1RS8, 1RS9, 1RSB, 1RSC, 1RSD, 1RSE, 1RSG, 1RSI, 1RSM, 1RSN, 1RSR, 1RSS, 1RST, 1RSU, 1RSV, 1RSY, 1RSZ, 1RT1, 1RT2, 1RT3, 1RT4, 1RT5, 1RT6, 1RT7, 1RT8, 1RT9, 1RTA, 1RTB, 1RTC, 1RTD, 1RTE, 1RTF, 1RTG, 1RTH, 1RTI, 1RTJ, 1RTK, 1RTL, 1RTM, 1RTP, 1RTQ, 1RTR, 1RTS, 1RTT, 1RTU, 1RTV, 1RTW, 1RTX, 1RTY, 1RTZ, 1RU0, 1RU1, 1RU2, 1RU3, 1RU4, 1RU7, 1RU9, 1RUA, 1RUK, 1RUL, 1RUM, 1RUN, 1RUO, 1RUP, 1RUQ, 1RUR, 1RUS, 1RUT, 1RUV, 1RUW, 1RUY, 1RUZ, 1RV0, 1RV1, 1RV3, 1RV4, 1RV5, 1RV6, 1RV7, 1RV8, 1RV9, 1RVA, 1RVB, 1RVC, 1RVD, 1RVE, 1RVG, 1RVJ, 1RVK, 1RVT, 1RVU, 1RVV, 1RVW, 1RVX, 1RVY, 1RVZ, 1RW0, 1RW1, 1RW4, 1RW8, 1RW9, 1RWA, 1RWB, 1RWC, 1RWE, 1RWF, 1RWG, 1RWH, 1RWI, 1RWJ, 1RWK, 1RWL, 1RWM, 1RWN, 1RWO, 1RWP, 1RWQ, 1RWR, 1RWV, 1RWW, 1RWX, 1RWY, 1RWZ, 1RX0, 1RX1, 1RX2, 1RX3, 1RX4, 1RX5, 1RX6, 1RX7, 1RX8, 1RX9, 1RXC, 1RXD, 1RXE, 1RXF, 1RXG, 1RXH, 1RXI, 1RXJ, 1RXK, 1RXM, 1RXO, 1RXP, 1RXQ, 1RXS, 1RXT, 1RXU, 1RXV, 1RXW, 1RXX, 1RXY, 1RXZ, 1RY0, 1RY2, 1RY5, 1RY6, 1RY7, 1RY8, 1RY9, 1RYA, 1RYB, 1RYC, 1RYD, 1RYE, 1RYF, 1RYH, 1RYI, 1RYL, 1RYM, 1RYN, 1RYO, 1RYP, 1RYQ, 1RYR, 1RYS, 1RYT, 1RYW, 1RYX, 1RYY, 1RYZ, 1RZ0, 1RZ1, 1RZ2, 1RZ3, 1RZ4, 1RZ5, 1RZ6, 1RZ7, 1RZ8, 1RZ9, 1RZA, 1RZB, 1RZC, 1RZD, 1RZE, 1RZF, 1RZG, 1RZH, 1RZI, 1RZJ, 1RZK, 1RZL, 1RZM, 1RZN, 1RZO, 1RZP, 1RZQ, 1RZR, 1RZT, 1RZU, 1RZV, 1RZX, 1RZY, 1RZZ, 1S00, 1S01, 1S02, 1S06, 1S07, 1S08, 1S09, 1S0A, 1S0B, 1S0C, 1S0D, 1S0E, 1S0F, 1S0G, 1S0H, 1S0I, 1S0J, 1S0L, 1S0M, 1S0N, 1S0O, 1S0P, 1S0Q, 1S0R, 1S0U, 1S0W, 1S0X, 1S0Y, 1S0Z, 1S10, 1S12, 1S13, 1S14, 1S16, 1S17, 1S18, 1S19, 1S1A, 1S1C, 1S1D, 1S1E, 1S1F, 1S1G, 1S1J, 1S1K, 1S1L, 1S1M, 1S1P, 1S1Q, 1S1R, 1S1S, 1S1T, 1S1U, 1S1V, 1S1W, 1S1X, 1S1Y, 1S1Z, 1S20, 1S21, 1S22, 1S23, 1S26, 1S28, 1S29, 1S2A, 1S2B, 1S2C, 1S2D, 1S2E, 1S2G, 1S2I, 1S2J, 1S2K, 1S2L, 1S2M, 1S2N, 1S2O, 1S2P, 1S2Q, 1S2R, 1S2T, 1S2U, 1S2V, 1S2W, 1S2X, 1S2Y, 1S2Z, 1S30, 1S31, 1S32, 1S35, 1S36, 1S38, 1S39, 1S3B, 1S3C, 1S3D, 1S3E, 1S3F, 1S3G, 1S3H, 1S3I, 1S3J, 1S3K, 1S3L, 1S3M, 1S3N, 1S3O, 1S3P, 1S3Q, 1S3R, 1S3S, 1S3T, 1S3U, 1S3V, 1S3W, 1S3X, 1S3Y, 1S3Z, 1S44, 1S45, 1S46, 1S47, 1S48, 1S49, 1S4B, 1S4C, 1S4D, 1S4E, 1S4F, 1S4I, 1S4K, 1S4M, 1S4N, 1S4O, 1S4P, 1S4Q, 1S4R, 1S4S, 1S4U, 1S4V, 1S4Y, 1S50, 1S51, 1S52, 1S53, 1S54, 1S55, 1S56, 1S57, 1S59, 1S5A, 1S5B, 1S5C, 1S5D, 1S5E, 1S5F, 1S5G, 1S5H, 1S5I, 1S5J, 1S5K, 1S5L, 1S5M, 1S5N, 1S5O, 1S5P, 1S5S, 1S5T, 1S5U, 1S5V, 1S5W, 1S5X, 1S5Y, 1S5Z, 1S60, 1S61, 1S63, 1S64, 1S66, 1S67, 1S68, 1S69, 1S6A, 1S6B, 1S6C, 1S6F, 1S6H, 1S6M, 1S6P, 1S6Q, 1S6R, 1S6V, 1S6Y, 1S6Z, 1S70, 1S73, 1S78, 1S7C, 1S7D, 1S7F, 1S7G, 1S7H, 1S7I, 1S7J, 1S7K, 1S7L, 1S7M, 1S7N, 1S7O, 1S7Q, 1S7R, 1S7S, 1S7T, 1S7U, 1S7V, 1S7W, 1S7X, 1S7Y, 1S7Z, 1S80, 1S81, 1S82, 1S83, 1S84, 1S85, 1S89, 1S8A, 1S8C, 1S8D, 1S8E, 1S8F, 1S8G, 1S8H, 1S8I, 1S8J, 1S8L, 1S8N, 1S8O, 1S94, 1S95, 1S96, 1S97, 1S98, 1S99, 1S9A, 1S9B, 1S9C, 1S9D, 1S9E, 1S9F, 1S9G, 1S9H, 1S9I, 1S9J, 1S9K, 1S9P, 1S9Q, 1S9R, 1S9T, 1S9U, 1S9V, 1S9W, 1S9X, 1S9Y, 1S9Z, 1SA0, 1SA1, 1SA3, 1SA4, 1SA5, 1SAC, 1SAR, 1SAT, 1SAU, 1SAV, 1SAW, 1SAX, 1SAY, 1SAZ, 1SB1, 1SB2, 1SB3, 1SB7, 1SB8, 1SB9, 1SBB, 1SBC, 1SBD, 1SBE, 1SBF, 1SBG, 1SBH, 1SBI, 1SBK, 1SBM, 1SBN, 1SBP, 1SBQ, 1SBR, 1SBS, 1SBT, 1SBW, 1SBX, 1SBY, 1SBZ, 1SC0, 1SC1, 1SC3, 1SC4, 1SC5, 1SC6, 1SC7, 1SC8, 1SC9, 1SCA, 1SCB, 1SCD, 1SCE, 1SCF, 1SCH, 1SCI, 1SCJ, 1SCK, 1SCM, 1SCN, 1SCQ, 1SCR, 1SCS, 1SCU, 1SCW, 1SCZ, 1SD0, 1SD1, 1SD2, 1SD3, 1SD4, 1SD5, 1SD6, 1SD7, 1SD8, 1SD9, 1SDA, 1SDB, 1SDD, 1SDE, 1SDI, 1SDJ, 1SDK, 1SDL, 1SDM, 1SDN, 1SDO, 1SDQ, 1SDT, 1SDU, 1SDV, 1SDW, 1SDX, 1SDY, 1SDZ, 1SE0, 1SE2, 1SE3, 1SE4, 1SE6, 1SE8, 1SEB, 1SED, 1SEF, 1SEG, 1SEH, 1SEI, 1SEJ, 1SEK, 1SEL, 1SEM, 1SEN, 1SEP, 1SEQ, 1SES, 1SET, 1SEU, 1SEV, 1SEZ, 1SF2, 1SF3, 1SF5, 1SF8, 1SF9, 1SFC, 1SFD, 1SFE, 1SFF, 1SFH, 1SFI, 1SFJ, 1SFK, 1SFL, 1SFN, 1SFP, 1SFQ, 1SFR, 1SFS, 1SFT, 1SFU, 1SFX, 1SFY, 1SG0, 1SG1, 1SG2, 1SG3, 1SG4, 1SG6, 1SG8, 1SG9, 1SGC, 1SGD, 1SGE, 1SGF, 1SGH, 1SGI, 1SGJ, 1SGK, 1SGL, 1SGM, 1SGN, 1SGP, 1SGQ, 1SGR, 1SGS, 1SGT, 1SGU, 1SGV, 1SGW, 1SGY, 1SGZ, 1SH0, 1SH2, 1SH3, 1SH5, 1SH6, 1SH7, 1SH8, 1SH9, 1SHA, 1SHB, 1SHD, 1SHF, 1SHG, 1SHH, 1SHJ, 1SHK, 1SHL, 1SHM, 1SHN, 1SHQ, 1SHR, 1SHS, 1SHT, 1SHU, 1SHV, 1SHW, 1SHX, 1SHY, 1SHZ, 1SI0, 1SI1, 1SI4, 1SI5, 1SI6, 1SI7, 1SI8, 1SI9, 1SIB, 1SIF, 1SIG, 1SIH, 1SII, 1SIJ, 1SIO, 1SIP, 1SIQ, 1SIR, 1SIU, 1SIV, 1SIW, 1SIX, 1SIZ, 1SJ0, 1SJ1, 1SJ2, 1SJ5, 1SJ7, 1SJ8, 1SJ9, 1SJA, 1SJB, 1SJC, 1SJD, 1SJE, 1SJH, 1SJI, 1SJM, 1SJN, 1SJP, 1SJS, 1SJV, 1SJW, 1SJX, 1SJY, 1SJZ, 1SK0, 1SK1, 1SK2, 1SK3, 1SK4, 1SK5, 1SK6, 1SK7, 1SK8, 1SK9, 1SKA, 1SKB, 1SKF, 1SKG, 1SKJ, 1SKM, 1SKN, 1SKO, 1SKQ, 1SKR, 1SKS, 1SKU, 1SKV, 1SKW, 1SKX, 1SKY, 1SKZ, 1SL0, 1SL1, 1SL2, 1SL3, 1SL4, 1SL5, 1SL6, 1SL7, 1SL8, 1SL9, 1SLA, 1SLB, 1SLC, 1SLD, 1SLE, 1SLF, 1SLG, 1SLH, 1SLI, 1SLL, 1SLM, 1SLN, 1SLQ, 1SLT, 1SLU, 1SLV, 1SLW, 1SLX, 1SLY, 1SM2, 1SM3, 1SM4, 1SM5, 1SM8, 1SM9, 1SMA, 1SMB, 1SMC, 1SMD, 1SME, 1SMF, 1SMH, 1SMI, 1SMJ, 1SMK, 1SML, 1SMM, 1SMN, 1SMO, 1SMP, 1SMQ, 1SMR, 1SMS, 1SMT, 1SMU, 1SMW, 1SMX, 1SMY, 1SN0, 1SN1, 1SN2, 1SN4, 1SN5, 1SN7, 1SN8, 1SN9, 1SNB, 1SNC, 1SND, 1SNF, 1SNG, 1SNK, 1SNM, 1SNN, 1SNO, 1SNP, 1SNQ, 1SNR, 1SNT, 1SNU, 1SNX, 1SNY, 1SNZ, 1SO0, 1SO2, 1SO3, 1SO4, 1SO5, 1SO6, 1SO7, 1SO8, 1SOA, 1SOF, 1SOG, 1SOI, 1SOJ, 1SOK, 1SOM, 1SON, 1SOO, 1SOQ, 1SOS, 1SOT, 1SOV, 1SOW, 1SOX, 1SOZ, 1SP3, 1SP4, 1SP5, 1SP8, 1SP9, 1SPA, 1SPB, 1SPD, 1SPE, 1SPG, 1SPH, 1SPI, 1SPJ, 1SPP, 1SPQ, 1SPR, 1SPS, 1SPU, 1SPV, 1SPX, 1SQ0, 1SQ1, 1SQ2, 1SQ3, 1SQ4, 1SQ5, 1SQ6, 1SQ7, 1SQ9, 1SQA, 1SQB, 1SQC, 1SQD, 1SQE, 1SQF, 1SQG, 1SQH, 1SQI, 1SQJ, 1SQK, 1SQL, 1SQM, 1SQN, 1SQO, 1SQP, 1SQQ, 1SQS, 1SQT, 1SQU, 1SQV, 1SQW, 1SQX, 1SQY, 1SQZ, 1SR0, 1SR4, 1SR5, 1SR6, 1SR7, 1SR8, 1SR9, 1SRA, 1SRD, 1SRE, 1SRF, 1SRG, 1SRH, 1SRI, 1SRJ, 1SRN, 1SRP, 1SRQ, 1SRR, 1SRS, 1SRU, 1SRV, 1SRX, 1SRY, 1SS4, 1SS8, 1SS9, 1SSA, 1SSB, 1SSC, 1SSD, 1SSG, 1SSH, 1SSM, 1SSP, 1SSQ, 1SST, 1SSW, 1SSX, 1SSY, 1ST0, 1ST2, 1ST3, 1ST4, 1ST6, 1ST8, 1ST9, 1STA, 1STB, 1STC, 1STD, 1STE, 1STF, 1STG, 1STH, 1STM, 1STN, 1STO, 1STP, 1STQ, 1STR, 1STS, 1STX, 1STY, 1STZ, 1SU0, 1SU1, 1SU2, 1SU3, 1SU4, 1SU5, 1SU6, 1SU7, 1SU8, 1SU9, 1SUA, 1SUB, 1SUC, 1SUD, 1SUE, 1SUF, 1SUG, 1SUI, 1SUJ, 1SUL, 1SUM, 1SUO, 1SUP, 1SUQ, 1SUR, 1SUS, 1SUU, 1SUW, 1SUX, 1SUZ, 1SV0, 1SV2, 1SV3, 1SV4, 1SV5, 1SV6, 1SV9, 1SVB, 1SVC, 1SVD, 1SVE, 1SVF, 1SVG, 1SVH, 1SVI, 1SVK, 1SVL, 1SVM, 1SVN, 1SVO, 1SVP, 1SVS, 1SVT, 1SVU, 1SVV, 1SVW, 1SVX, 1SVY, 1SVZ, 1SW0, 1SW1, 1SW2, 1SW3, 1SW4, 1SW5, 1SW6, 1SW7, 1SWA, 1SWB, 1SWC, 1SWD, 1SWE, 1SWF, 1SWG, 1SWH, 1SWI, 1SWJ, 1SWK, 1SWL, 1SWM, 1SWN, 1SWO, 1SWP, 1SWQ, 1SWR, 1SWS, 1SWT, 1SWU, 1SWV, 1SWW, 1SWX, 1SWY, 1SWZ, 1SX2, 1SX3, 1SX4, 1SX5, 1SX6, 1SX7, 1SX8, 1SXA, 1SXB, 1SXC, 1SXG, 1SXH, 1SXI, 1SXJ, 1SXK, 1SXN, 1SXP, 1SXQ, 1SXR, 1SXS, 1SXT, 1SXU, 1SXV, 1SXW, 1SXX, 1SXY, 1SXZ, 1SY0, 1SY1, 1SY2, 1SY3, 1SY6, 1SY7, 1SYB, 1SYC, 1SYD, 1SYE, 1SYF, 1SYG, 1SYH, 1SYI, 1SYK, 1SYL, 1SYN, 1SYO, 1SYQ, 1SYR, 1SYS, 1SYT, 1SYV, 1SYX, 1SYY, 1SZ0, 1SZ2, 1SZ3, 1SZ6, 1SZ7, 1SZ8, 1SZ9, 1SZA, 1SZB, 1SZC, 1SZD, 1SZE, 1SZF, 1SZG, 1SZH, 1SZI, 1SZJ, 1SZK, 1SZM, 1SZN, 1SZO, 1SZP, 1SZQ, 1SZR, 1SZS, 1SZT, 1SZU, 1SZW, 1SZX, 1SZZ, 1T00, 1T01, 1T02, 1T03, 1T04, 1T05, 1T06, 1T07, 1T08, 1T09, 1T0A, 1T0B, 1T0F, 1T0H, 1T0I, 1T0J, 1T0L, 1T0M, 1T0N, 1T0O, 1T0P, 1T0Q, 1T0R, 1T0S, 1T0T, 1T0U, 1T0Z, 1T10, 1T11, 1T13, 1T14, 1T15, 1T16, 1T18, 1T19, 1T1A, 1T1B, 1T1C, 1T1D, 1T1E, 1T1F, 1T1G, 1T1I, 1T1J, 1T1L, 1T1N, 1T1R, 1T1S, 1T1U, 1T1V, 1T1W, 1T1X, 1T1Y, 1T1Z, 1T20, 1T21, 1T22, 1T24, 1T25, 1T26, 1T27, 1T29, 1T2A, 1T2B, 1T2C, 1T2D, 1T2E, 1T2F, 1T2H, 1T2I, 1T2J, 1T2K, 1T2L, 1T2N, 1T2O, 1T2P, 1T2Q, 1T2T, 1T2U, 1T2V, 1T2W, 1T2X, 1T31, 1T32, 1T33, 1T34, 1T35, 1T36, 1T37, 1T38, 1T39, 1T3A, 1T3B, 1T3C, 1T3D, 1T3E, 1T3F, 1T3G, 1T3H, 1T3I, 1T3J, 1T3L, 1T3M, 1T3N, 1T3P, 1T3Q, 1T3R, 1T3S, 1T3T, 1T3U, 1T3W, 1T3X, 1T3Y, 1T3Z, 1T40, 1T41, 1T43, 1T44, 1T45, 1T46, 1T47, 1T48, 1T49, 1T4A, 1T4B, 1T4C, 1T4D, 1T4E, 1T4F, 1T4G, 1T4I, 1T4J, 1T4K, 1T4M, 1T4O, 1T4P, 1T4Q, 1T4R, 1T4S, 1T4T, 1T4U, 1T4V, 1T4W, 1T56, 1T57, 1T5A, 1T5B, 1T5C, 1T5D, 1T5E, 1T5F, 1T5G, 1T5H, 1T5I, 1T5J, 1T5K, 1T5L, 1T5O, 1T5P, 1T5R, 1T5S, 1T5T, 1T5W, 1T5X, 1T5Y, 1T5Z, 1T60, 1T61, 1T62, 1T63, 1T64, 1T65, 1T66, 1T67, 1T68, 1T69, 1T6A, 1T6B, 1T6C, 1T6D, 1T6E, 1T6F, 1T6G, 1T6H, 1T6I, 1T6J, 1T6K, 1T6L, 1T6M, 1T6N, 1T6O, 1T6P, 1T6Q, 1T6S, 1T6T, 1T6U, 1T6V, 1T6X, 1T6Y, 1T6Z, 1T70, 1T71, 1T72, 1T73, 1T74, 1T75, 1T76, 1T77, 1T79, 1T7A, 1T7B, 1T7C, 1T7D, 1T7E, 1T7F, 1T7H, 1T7I, 1T7J, 1T7K, 1T7L, 1T7M, 1T7N, 1T7O, 1T7P, 1T7Q, 1T7R, 1T7S, 1T7T, 1T7V, 1T7W, 1T7X, 1T7Y, 1T7Z, 1T80, 1T82, 1T83, 1T85, 1T86, 1T87, 1T88, 1T89, 1T8A, 1T8B, 1T8E, 1T8F, 1T8G, 1T8H, 1T8I, 1T8K, 1T8L, 1T8M, 1T8N, 1T8O, 1T8P, 1T8Q, 1T8R, 1T8S, 1T8T, 1T8U, 1T8W, 1T8X, 1T8Y, 1T8Z, 1T90, 1T91, 1T92, 1T93, 1T94, 1T95, 1T96, 1T97, 1T98, 1T99, 1T9A, 1T9B, 1T9C, 1T9D, 1T9F, 1T9G, 1T9H, 1T9I, 1T9J, 1T9K, 1T9M, 1T9N, 1T9O, 1T9P, 1T9Q, 1T9R, 1T9S, 1T9T, 1T9U, 1T9V, 1T9W, 1T9X, 1T9Y, 1T9Z, 1TA0, 1TA1, 1TA2, 1TA3, 1TA4, 1TA6, 1TA8, 1TA9, 1TAB, 1TAD, 1TAE, 1TAF, 1TAG, 1TAH, 1TAL, 1TAQ, 1TAR, 1TAS, 1TAT, 1TAU, 1TAW, 1TAY, 1TAZ, 1TB0, 1TB3, 1TB4, 1TB5, 1TB6, 1TB7, 1TBB, 1TBE, 1TBF, 1TBG, 1TBH, 1TBJ, 1TBL, 1TBP, 1TBQ, 1TBR, 1TBT, 1TBU, 1TBW, 1TBX, 1TBY, 1TBZ, 1TC0, 1TC1, 1TC2, 1TC3, 1TC5, 1TC6, 1TC8, 1TCA, 1TCB, 1TCC, 1TCD, 1TCF, 1TCM, 1TCO, 1TCR, 1TCS, 1TCU, 1TCV, 1TCW, 1TCX, 1TCY, 1TCZ, 1TD0, 1TD1, 1TD2, 1TD3, 1TD4, 1TD5, 1TD6, 1TD7, 1TD9, 1TDA, 1TDB, 1TDC, 1TDE, 1TDF, 1TDG, 1TDH, 1TDI, 1TDJ, 1TDK, 1TDL, 1TDN, 1TDO, 1TDQ, 1TDR, 1TDT, 1TDU, 1TDV, 1TDW, 1TDY, 1TDZ, 1TE0, 1TE1, 1TE2, 1TE3, 1TE5, 1TE6, 1TEC, 1TED, 1TEE, 1TEF, 1TEG, 1TEH, 1TEI, 1TEJ, 1TEL, 1TEM, 1TEN, 1TEQ, 1TES, 1TET, 1TEU, 1TEV, 1TEW, 1TEX, 1TEZ, 1TF0, 1TF1, 1TF2, 1TF4, 1TF5, 1TF6, 1TF7, 1TF8, 1TF9, 1TFA, 1TFC, 1TFD, 1TFE, 1TFF, 1TFG, 1TFH, 1TFJ, 1TFK, 1TFM, 1TFO, 1TFP, 1TFR, 1TFU, 1TFV, 1TFX, 1TFZ, 1TG0, 1TG1, 1TG2, 1TG3, 1TG4, 1TG5, 1TG6, 1TG7, 1TG8, 1TG9, 1TGB, 1TGC, 1TGH, 1TGJ, 1TGK, 1TGL, 1TGM, 1TGN, 1TGO, 1TGR, 1TGS, 1TGT, 1TGU, 1TGV, 1TGX, 1TGY, 1TGZ, 1TH0, 1TH1, 1TH2, 1TH3, 1TH4, 1TH6, 1TH7, 1TH8, 1TH9, 1THA, 1THB, 1THC, 1THE, 1THF, 1THG, 1THI, 1THJ, 1THK, 1THL, 1THM, 1THN, 1THO, 1THP, 1THQ, 1THR, 1THS, 1THT, 1THU, 1THV, 1THW, 1THX, 1THY, 1THZ, 1TI1, 1TI2, 1TI4, 1TI6, 1TI7, 1TI8, 1TIA, 1TIB, 1TIC, 1TID, 1TIE, 1TIF, 1TIG, 1TII, 1TIJ, 1TIK, 1TIL, 1TIM, 1TIO, 1TIP, 1TIQ, 1TIS, 1TIW, 1TIY, 1TJ0, 1TJ1, 1TJ2, 1TJ3, 1TJ4, 1TJ5, 1TJ6, 1TJ7, 1TJ9, 1TJB, 1TJC, 1TJD, 1TJE, 1TJF, 1TJG, 1TJH, 1TJI, 1TJJ, 1TJK, 1TJL, 1TJM, 1TJN, 1TJO, 1TJP, 1TJR, 1TJS, 1TJT, 1TJU, 1TJV, 1TJW, 1TJX, 1TJY, 1TK0, 1TK1, 1TK2, 1TK3, 1TK4, 1TK5, 1TK6, 1TK8, 1TK9, 1TKA, 1TKB, 1TKC, 1TKD, 1TKE, 1TKF, 1TKG, 1TKH, 1TKI, 1TKJ, 1TKK, 1TKL, 1TKO, 1TKP, 1TKR, 1TKS, 1TKT, 1TKU, 1TKX, 1TKY, 1TKZ, 1TL1, 1TL2, 1TL3, 1TL7, 1TL8, 1TL9, 1TLA, 1TLB, 1TLC, 1TLD, 1TLF, 1TLG, 1TLI, 1TLJ, 1TLK, 1TLL, 1TLM, 1TLO, 1TLP, 1TLQ, 1TLS, 1TLT, 1TLU, 1TLV, 1TLW, 1TLX, 1TLY, 1TLZ, 1TM0, 1TM1, 1TM2, 1TM3, 1TM4, 1TM5, 1TM7, 1TMB, 1TMC, 1TMG, 1TMH, 1TMI, 1TMJ, 1TMK, 1TML, 1TMM, 1TMN, 1TMO, 1TMQ, 1TMT, 1TMU, 1TMX, 1TMY, 1TN0, 1TN3, 1TN4, 1TN5, 1TN6, 1TN7, 1TN8, 1TNB, 1TND, 1TNF, 1TNG, 1TNH, 1TNI, 1TNJ, 1TNK, 1TNL, 1TNO, 1TNR, 1TNU, 1TNY, 1TNZ, 1TO0, 1TO1, 1TO2, 1TO3, 1TO4, 1TO5, 1TO6, 1TO9, 1TOA, 1TOC, 1TOE, 1TOG, 1TOH, 1TOI, 1TOJ, 1TOK, 1TOL, 1TOM, 1TON, 1TOO, 1TOP, 1TOQ, 1TOU, 1TOV, 1TOW, 1TOX, 1TP0, 1TP2, 1TP3, 1TP5, 1TP6, 1TP7, 1TP8, 1TP9, 1TPA, 1TPB, 1TPC, 1TPD, 1TPE, 1TPF, 1TPH, 1TPK, 1TPL, 1TPO, 1TPP, 1TPS, 1TPT, 1TPU, 1TPV, 1TPW, 1TPX, 1TPY, 1TPZ, 1TQ0, 1TQ2, 1TQ3, 1TQ4, 1TQ5, 1TQ6, 1TQ7, 1TQ8, 1TQ9, 1TQB, 1TQC, 1TQD, 1TQE, 1TQF, 1TQG, 1TQH, 1TQI, 1TQJ, 1TQL, 1TQM, 1TQN, 1TQO, 1TQP, 1TQQ, 1TQS, 1TQT, 1TQU, 1TQV, 1TQW, 1TQX, 1TQY, 1TR0, 1TR1, 1TR2, 1TR5, 1TR7, 1TR8, 1TR9, 1TRB, 1TRD, 1TRE, 1TRG, 1TRH, 1TRI, 1TRK, 1TRM, 1TRN, 1TRO, 1TRP, 1TRQ, 1TRR, 1TRY, 1TRZ, 1TS0, 1TS2, 1TS3, 1TS4, 1TS5, 1TS6, 1TS7, 1TS8, 1TS9, 1TSD, 1TSF, 1TSH, 1TSI, 1TSJ, 1TSL, 1TSM, 1TSN, 1TSP, 1TSQ, 1TSR, 1TSU, 1TSV, 1TSW, 1TSX, 1TSY, 1TSZ, 1TT0, 1TT1, 1TT2, 1TT4, 1TT5, 1TT6, 1TT7, 1TT8, 1TT9, 1TTA, 1TTB, 1TTC, 1TTH, 1TTI, 1TTJ, 1TTM, 1TTO, 1TTP, 1TTQ, 1TTR, 1TTU, 1TTW, 1TTZ, 1TU0, 1TU1, 1TU3, 1TU4, 1TU5, 1TU6, 1TU7, 1TU8, 1TU9, 1TUA, 1TUC, 1TUD, 1TUE, 1TUF, 1TUG, 1TUH, 1TUI, 1TUK, 1TUL, 1TUO, 1TUP, 1TUU, 1TUV, 1TUW, 1TUX, 1TUY, 1TV2, 1TV3, 1TV4, 1TV5, 1TV6, 1TV7, 1TV8, 1TV9, 1TVA, 1TVB, 1TVD, 1TVE, 1TVF, 1TVG, 1TVH, 1TVL, 1TVN, 1TVO, 1TVP, 1TVQ, 1TVR, 1TVU, 1TVV, 1TVW, 1TVX, 1TVY, 1TVZ, 1TW0, 1TW1, 1TW2, 1TW3, 1TW4, 1TW5, 1TW6, 1TW7, 1TW8, 1TW9, 1TWA, 1TWB, 1TWC, 1TWD, 1TWE, 1TWF, 1TWG, 1TWH, 1TWI, 1TWJ, 1TWL, 1TWM, 1TWN, 1TWQ, 1TWR, 1TWS, 1TWU, 1TWW, 1TWX, 1TWY, 1TWZ, 1TX0, 1TX2, 1TX3, 1TX4, 1TX6, 1TX7, 1TX8, 1TX9, 1TXC, 1TXD, 1TXF, 1TXG, 1TXI, 1TXJ, 1TXK, 1TXL, 1TXN, 1TXO, 1TXQ, 1TXR, 1TXT, 1TXU, 1TXX, 1TXY, 1TXZ, 1TY0, 1TY2, 1TY4, 1TY8, 1TY9, 1TYA, 1TYB, 1TYC, 1TYD, 1TYE, 1TYF, 1TYG, 1TYH, 1TYJ, 1TYL, 1TYM, 1TYN, 1TYO, 1TYP, 1TYQ, 1TYR, 1TYS, 1TYT, 1TYU, 1TYV, 1TYW, 1TYX, 1TYY, 1TYZ, 1TZ0, 1TZ2, 1TZ3, 1TZ6, 1TZ7, 1TZ8, 1TZ9, 1TZA, 1TZB, 1TZC, 1TZD, 1TZE, 1TZF, 1TZG, 1TZH, 1TZI, 1TZJ, 1TZK, 1TZL, 1TZM, 1TZN, 1TZO, 1TZP, 1TZQ, 1TZS, 1TZT, 1TZU, 1TZV, 1TZW, 1TZX, 1TZY, 1TZZ, 1U00, 1U02, 1U04, 1U05, 1U06, 1U07, 1U08, 1U09, 1U0A, 1U0C, 1U0D, 1U0E, 1U0F, 1U0G, 1U0H, 1U0J, 1U0K, 1U0L, 1U0M, 1U0N, 1U0O, 1U0Q, 1U0R, 1U0S, 1U0T, 1U0U, 1U0V, 1U0W, 1U0X, 1U0Y, 1U0Z, 1U10, 1U11, 1U12, 1U13, 1U14, 1U15, 1U16, 1U17, 1U18, 1U19, 1U1B, 1U1C, 1U1D, 1U1E, 1U1F, 1U1G, 1U1H, 1U1I, 1U1J, 1U1K, 1U1L, 1U1M, 1U1N, 1U1O, 1U1P, 1U1Q, 1U1R, 1U1S, 1U1T, 1U1U, 1U1V, 1U1W, 1U1X, 1U1Z, 1U20, 1U21, 1U22, 1U24, 1U25, 1U26, 1U27, 1U28, 1U29, 1U2B, 1U2C, 1U2D, 1U2E, 1U2G, 1U2H, 1U2J, 1U2K, 1U2L, 1U2M, 1U2O, 1U2P, 1U2Q, 1U2R, 1U2S, 1U2T, 1U2V, 1U2W, 1U2X, 1U2Y, 1U2Z, 1U30, 1U31, 1U32, 1U33, 1U35, 1U36, 1U3A, 1U3C, 1U3D, 1U3E, 1U3F, 1U3G, 1U3H, 1U3I, 1U3J, 1U3L, 1U3P, 1U3Q, 1U3R, 1U3S, 1U3T, 1U3U, 1U3V, 1U3W, 1U3Y, 1U3Z, 1U40, 1U41, 1U42, 1U43, 1U45, 1U46, 1U47, 1U48, 1U49, 1U4B, 1U4C, 1U4D, 1U4E, 1U4F, 1U4G, 1U4H, 1U4J, 1U4L, 1U4M, 1U4N, 1U4O, 1U4P, 1U4Q, 1U4R, 1U4S, 1U53, 1U54, 1U55, 1U56, 1U58, 1U59, 1U5A, 1U5B, 1U5C, 1U5D, 1U5E, 1U5F, 1U5G, 1U5H, 1U5I, 1U5J, 1U5K, 1U5O, 1U5P, 1U5Q, 1U5R, 1U5T, 1U5U, 1U5V, 1U5W, 1U5X, 1U5Y, 1U5Z, 1U60, 1U61, 1U65, 1U67, 1U68, 1U69, 1U6A, 1U6D, 1U6E, 1U6G, 1U6H, 1U6I, 1U6J, 1U6K, 1U6L, 1U6M, 1U6Q, 1U6R, 1U6S, 1U6T, 1U6Z, 1U70, 1U71, 1U72, 1U73, 1U74, 1U75, 1U76, 1U77, 1U78, 1U79, 1U7B, 1U7C, 1U7D, 1U7F, 1U7G, 1U7H, 1U7I, 1U7K, 1U7L, 1U7N, 1U7O, 1U7P, 1U7R, 1U7S, 1U7T, 1U7U, 1U7V, 1U7W, 1U7X, 1U7Z, 1U80, 1U83, 1U84, 1U87, 1U88, 1U8A, 1U8B, 1U8C, 1U8E, 1U8F, 1U8G, 1U8H, 1U8I, 1U8J, 1U8K, 1U8L, 1U8M, 1U8N, 1U8O, 1U8P, 1U8Q, 1U8R, 1U8S, 1U8T, 1U8U, 1U8V, 1U8W, 1U8X, 1U8Y, 1U8Z, 1U90, 1U91, 1U92, 1U93, 1U94, 1U95, 1U98, 1U99, 1U9A, 1U9B, 1U9C, 1U9D, 1U9E, 1U9F, 1U9G, 1U9H, 1U9I, 1U9J, 1U9K, 1U9L, 1U9M, 1U9N, 1U9O, 1U9P, 1U9Q, 1U9R, 1U9T, 1U9U, 1U9V, 1U9W, 1U9X, 1U9Y, 1U9Z, 1UA0, 1UA1, 1UA2, 1UA3, 1UA4, 1UA5, 1UA6, 1UA7, 1UA8, 1UAA, 1UAC, 1UAD, 1UAE, 1UAG, 1UAI, 1UAJ, 1UAK, 1UAL, 1UAM, 1UAN, 1UAQ, 1UAR, 1UAS, 1UAT, 1UAX, 1UAY, 1UAZ, 1UB0, 1UB2, 1UB3, 1UB4, 1UB5, 1UB6, 1UB7, 1UB8, 1UB9, 1UBB, 1UBC, 1UBD, 1UBE, 1UBF, 1UBG, 1UBH, 1UBI, 1UBJ, 1UBK, 1UBL, 1UBM, 1UBN, 1UBO, 1UBP, 1UBQ, 1UBR, 1UBS, 1UBT, 1UBU, 1UBV, 1UBW, 1UBX, 1UBY, 1UBZ, 1UC0, 1UC2, 1UC3, 1UC4, 1UC5, 1UC7, 1UC8, 1UC9, 1UCA, 1UCB, 1UCC, 1UCD, 1UCF, 1UCG, 1UCH, 1UCI, 1UCJ, 1UCK, 1UCL, 1UCN, 1UCO, 1UCQ, 1UCR, 1UCS, 1UCT, 1UCW, 1UCX, 1UCY, 1UD0, 1UD1, 1UD2, 1UD3, 1UD4, 1UD5, 1UD6, 1UD8, 1UD9, 1UDA, 1UDB, 1UDC, 1UDD, 1UDE, 1UDG, 1UDH, 1UDI, 1UDN, 1UDO, 1UDQ, 1UDR, 1UDS, 1UDT, 1UDU, 1UDV, 1UDW, 1UDX, 1UDY, 1UDZ, 1UE0, 1UE1, 1UE2, 1UE3, 1UE4, 1UE5, 1UE6, 1UE7, 1UE8, 1UEA, 1UEB, 1UEC, 1UED, 1UEF, 1UEG, 1UEH, 1UEI, 1UEJ, 1UEK, 1UER, 1UES, 1UET, 1UEU, 1UEV, 1UEX, 1UF3, 1UF4, 1UF5, 1UF7, 1UF8, 1UF9, 1UFA, 1UFB, 1UFH, 1UFI, 1UFJ, 1UFK, 1UFL, 1UFO, 1UFP, 1UFQ, 1UFR, 1UFU, 1UFV, 1UFY, 1UG3, 1UG4, 1UG6, 1UG9, 1UGA, 1UGB, 1UGC, 1UGD, 1UGE, 1UGF, 1UGG, 1UGH, 1UGI, 1UGM, 1UGN, 1UGP, 1UGQ, 1UGR, 1UGS, 1UGU, 1UGW, 1UGX, 1UGY, 1UH0, 1UH1, 1UH2, 1UH3, 1UH4, 1UH5, 1UH7, 1UH8, 1UH9, 1UHA, 1UHB, 1UHD, 1UHE, 1UHG, 1UHH, 1UHI, 1UHJ, 1UHK, 1UHL, 1UHN, 1UHO, 1UHV, 1UHX, 1UHY, 1UI0, 1UI1, 1UI5, 1UI6, 1UI7, 1UI8, 1UI9, 1UIA, 1UIB, 1UIC, 1UID, 1UIE, 1UIF, 1UIG, 1UIH, 1UII, 1UIJ, 1UIK, 1UIM, 1UIN, 1UIO, 1UIP, 1UIR, 1UIS, 1UIU, 1UIV, 1UIW, 1UIX, 1UIY, 1UIZ, 1UJ0, 1UJ1, 1UJ2, 1UJ3, 1UJ4, 1UJ5, 1UJ6, 1UJ8, 1UJB, 1UJC, 1UJJ, 1UJK, 1UJM, 1UJN, 1UJP, 1UJQ, 1UJW, 1UJZ, 1UK0, 1UK1, 1UK2, 1UK3, 1UK4, 1UK6, 1UK7, 1UK8, 1UK9, 1UKA, 1UKB, 1UKC, 1UKE, 1UKF, 1UKG, 1UKH, 1UKI, 1UKJ, 1UKK, 1UKL, 1UKM, 1UKO, 1UKP, 1UKQ, 1UKR, 1UKS, 1UKT, 1UKU, 1UKV, 1UKW, 1UKY, 1UKZ, 1UL1, 1UL3, 1UL9, 1ULA, 1ULB, 1ULC, 1ULD, 1ULE, 1ULF, 1ULG, 1ULH, 1ULI, 1ULJ, 1ULK, 1ULM, 1ULN, 1ULQ, 1ULR, 1ULS, 1ULT, 1ULU, 1ULV, 1ULW, 1ULX, 1ULY, 1ULZ, 1UM0, 1UM2, 1UM4, 1UM5, 1UM6, 1UM8, 1UM9, 1UMA, 1UMB, 1UMC, 1UMD, 1UMF, 1UMG, 1UMH, 1UMI, 1UMJ, 1UMK, 1UML, 1UMN, 1UMO, 1UMP, 1UMR, 1UMU, 1UMV, 1UMW, 1UMX, 1UMY, 1UMZ, 1UN0, 1UN1, 1UN2, 1UN3, 1UN4, 1UN5, 1UN8, 1UN9, 1UNA, 1UNB, 1UNE, 1UNF, 1UNG, 1UNH, 1UNK, 1UNL, 1UNN, 1UNP, 1UNQ, 1UNR, 1UNS, 1UNT, 1UNU, 1UNV, 1UNW, 1UNX, 1UNY, 1UNZ, 1UO0, 1UO1, 1UO2, 1UO3, 1UO4, 1UO5, 1UO6, 1UO9, 1UOB, 1UOC, 1UOD, 1UOE, 1UOF, 1UOG, 1UOH, 1UOJ, 1UOK, 1UOL, 1UOM, 1UOO, 1UOP, 1UOQ, 1UOR, 1UOS, 1UOT, 1UOU, 1UOV, 1UOW, 1UOY, 1UOZ, 1UP0, 1UP1, 1UP2, 1UP3, 1UP4, 1UP5, 1UP6, 1UP7, 1UP8, 1UP9, 1UPA, 1UPB, 1UPC, 1UPD, 1UPF, 1UPG, 1UPI, 1UPJ, 1UPK, 1UPL, 1UPM, 1UPP, 1UPQ, 1UPR, 1UPS, 1UPT, 1UPU, 1UPV, 1UPW, 1UPX, 1UQ4, 1UQ5, 1UQR, 1UQS, 1UQT, 1UQU, 1UQW, 1UQX, 1UQY, 1UQZ, 1UR0, 1UR1, 1UR2, 1UR3, 1UR4, 1UR5, 1UR8, 1UR9, 1URA, 1URB, 1URC, 1URD, 1URG, 1URH, 1URI, 1URJ, 1URL, 1URM, 1URO, 1URP, 1URQ, 1URR, 1URS, 1URT, 1URU, 1URV, 1URW, 1URX, 1URY, 1URZ, 1US0, 1US1, 1US2, 1US3, 1US4, 1US5, 1US6, 1US7, 1US8, 1USB, 1USC, 1USD, 1USE, 1USF, 1USG, 1USH, 1USI, 1USK, 1USL, 1USM, 1USN, 1USO, 1USP, 1USQ, 1USR, 1USU, 1USV, 1USW, 1USX, 1USY, 1USZ, 1UT0, 1UT1, 1UT2, 1UT4, 1UT5, 1UT6, 1UT7, 1UT8, 1UT9, 1UTB, 1UTC, 1UTD, 1UTE, 1UTG, 1UTH, 1UTI, 1UTJ, 1UTK, 1UTL, 1UTM, 1UTN, 1UTO, 1UTP, 1UTQ, 1UTT, 1UTU, 1UTX, 1UTY, 1UTZ, 1UU0, 1UU1, 1UU2, 1UU3, 1UU4, 1UU5, 1UU6, 1UU7, 1UU8, 1UU9, 1UUE, 1UUF, 1UUG, 1UUH, 1UUJ, 1UUM, 1UUN, 1UUO, 1UUP, 1UUQ, 1UUR, 1UUS, 1UUT, 1UUV, 1UUW, 1UUX, 1UUY, 1UUZ, 1UV0, 1UV4, 1UV5, 1UV6, 1UV7, 1UVA, 1UVB, 1UVC, 1UVH, 1UVO, 1UVP, 1UVQ, 1UVR, 1UVS, 1UVT, 1UVU, 1UVX, 1UVY, 1UVZ, 1UW1, 1UW3, 1UW4, 1UW5, 1UW6, 1UW7, 1UW8, 1UW9, 1UWA, 1UWB, 1UWC, 1UWE, 1UWF, 1UWG, 1UWH, 1UWI, 1UWJ, 1UWK, 1UWL, 1UWM, 1UWN, 1UWP, 1UWQ, 1UWR, 1UWS, 1UWT, 1UWU, 1UWV, 1UWW, 1UWX, 1UWY, 1UWZ, 1UX0, 1UX1, 1UX2, 1UX4, 1UX5, 1UX6, 1UX7, 1UX8, 1UX9, 1UXA, 1UXB, 1UXE, 1UXG, 1UXH, 1UXI, 1UXJ, 1UXK, 1UXL, 1UXM, 1UXN, 1UXO, 1UXP, 1UXQ, 1UXR, 1UXS, 1UXT, 1UXU, 1UXV, 1UXW, 1UXX, 1UXY, 1UXZ, 1UY0, 1UY1, 1UY2, 1UY3, 1UY4, 1UY6, 1UY7, 1UY8, 1UY9, 1UYC, 1UYD, 1UYE, 1UYF, 1UYG, 1UYH, 1UYI, 1UYJ, 1UYK, 1UYL, 1UYM, 1UYN, 1UYO, 1UYP, 1UYQ, 1UYR, 1UYS, 1UYT, 1UYU, 1UYV, 1UYW, 1UYX, 1UYY, 1UYZ, 1UZ0, 1UZ1, 1UZ2, 1UZ3, 1UZ4, 1UZ5, 1UZ6, 1UZ8, 1UZ9, 1UZA, 1UZB, 1UZD, 1UZE, 1UZF, 1UZG, 1UZH, 1UZI, 1UZJ, 1UZK, 1UZL, 1UZM, 1UZN, 1UZP, 1UZQ, 1UZR, 1UZU, 1UZV, 1UZW, 1UZX, 1UZY, 1UZZ, 1V00, 1V02, 1V03, 1V04, 1V05, 1V07, 1V08, 1V0A, 1V0B, 1V0C, 1V0D, 1V0E, 1V0F, 1V0H, 1V0J, 1V0K, 1V0L, 1V0M, 1V0N, 1V0O, 1V0P, 1V0R, 1V0S, 1V0T, 1V0U, 1V0V, 1V0W, 1V0Y, 1V0Z, 1V10, 1V11, 1V13, 1V14, 1V15, 1V16, 1V18, 1V19, 1V1A, 1V1B, 1V1F, 1V1G, 1V1H, 1V1I, 1V1J, 1V1K, 1V1M, 1V1O, 1V1P, 1V1Q, 1V1R, 1V1S, 1V1T, 1V25, 1V26, 1V29, 1V2A, 1V2B, 1V2D, 1V2E, 1V2F, 1V2G, 1V2H, 1V2I, 1V2J, 1V2K, 1V2L, 1V2M, 1V2N, 1V2O, 1V2P, 1V2Q, 1V2R, 1V2S, 1V2T, 1V2U, 1V2V, 1V2W, 1V2X, 1V2Z, 1V30, 1V33, 1V34, 1V35, 1V37, 1V39, 1V3B, 1V3C, 1V3D, 1V3E, 1V3H, 1V3I, 1V3J, 1V3K, 1V3L, 1V3M, 1V3N, 1V3O, 1V3P, 1V3Q, 1V3R, 1V3S, 1V3T, 1V3U, 1V3V, 1V3W, 1V3X, 1V3Y, 1V3Z, 1V40, 1V41, 1V43, 1V45, 1V47, 1V48, 1V4A, 1V4B, 1V4E, 1V4G, 1V4H, 1V4I, 1V4J, 1V4K, 1V4L, 1V4N, 1V4P, 1V4S, 1V4T, 1V4U, 1V4V, 1V4W, 1V4X, 1V4Y, 1V51, 1V53, 1V54, 1V55, 1V57, 1V58, 1V59, 1V5B, 1V5C, 1V5D, 1V5E, 1V5F, 1V5G, 1V5H, 1V5I, 1V5V, 1V5W, 1V5X, 1V5Y, 1V5Z, 1V67, 1V6A, 1V6C, 1V6D, 1V6H, 1V6I, 1V6J, 1V6K, 1V6L, 1V6M, 1V6N, 1V6O, 1V6P, 1V6S, 1V6T, 1V6U, 1V6V, 1V6W, 1V6X, 1V6Y, 1V6Z, 1V70, 1V71, 1V72, 1V73, 1V74, 1V75, 1V76, 1V77, 1V79, 1V7A, 1V7C, 1V7L, 1V7M, 1V7N, 1V7O, 1V7P, 1V7Q, 1V7R, 1V7S, 1V7T, 1V7U, 1V7V, 1V7W, 1V7X, 1V7Y, 1V7Z, 1V82, 1V83, 1V84, 1V8B, 1V8C, 1V8D, 1V8E, 1V8F, 1V8G, 1V8H, 1V8I, 1V8J, 1V8K, 1V8L, 1V8M, 1V8N, 1V8O, 1V8P, 1V8Q, 1V8R, 1V8S, 1V8T, 1V8U, 1V8V, 1V8W, 1V8X, 1V8Y, 1V8Z, 1V93, 1V94, 1V96, 1V97, 1V98, 1V9A, 1V9C, 1V9D, 1V9E, 1V9F, 1V9H, 1V9I, 1V9K, 1V9L, 1V9M, 1V9N, 1V9O, 1V9P, 1V9Q, 1V9S, 1V9T, 1V9Y, 1V9Z, 1VA0, 1VA4, 1VA5, 1VA6, 1VA7, 1VAC, 1VAD, 1VAF, 1VAG, 1VAH, 1VAI, 1VAJ, 1VAK, 1VAL, 1VAM, 1VAO, 1VAP, 1VAR, 1VAS, 1VAT, 1VAU, 1VAV, 1VAX, 1VAY, 1VB0, 1VB2, 1VB3, 1VB4, 1VB5, 1VB6, 1VB9, 1VBF, 1VBG, 1VBH, 1VBI, 1VBJ, 1VBK, 1VBL, 1VBM, 1VBN, 1VBO, 1VBP, 1VBR, 1VBS, 1VBT, 1VBU, 1VBV, 1VBW, 1VC1, 1VC2, 1VC3, 1VC4, 1VC8, 1VC9, 1VCA, 1VCB, 1VCC, 1VCD, 1VCE, 1VCF, 1VCG, 1VCH, 1VCI, 1VCJ, 1VCK, 1VCL, 1VCM, 1VCN, 1VCO, 1VCP, 1VCQ, 1VCT, 1VCU, 1VCV, 1VCW, 1VCY, 1VCZ, 1VD1, 1VD3, 1VD5, 1VD6, 1VDC, 1VDD, 1VDE, 1VDF, 1VDG, 1VDH, 1VDK, 1VDM, 1VDN, 1VDP, 1VDQ, 1VDR, 1VDS, 1VDT, 1VDV, 1VDW, 1VDX, 1VDZ, 1VE0, 1VE1, 1VE2, 1VE3, 1VE4, 1VE5, 1VE6, 1VE7, 1VE8, 1VE9, 1VEA, 1VEB, 1VEC, 1VED, 1VEF, 1VEI, 1VEL, 1VEM, 1VEN, 1VEO, 1VEP, 1VEQ, 1VER, 1VES, 1VET, 1VEU, 1VEV, 1VEW, 1VEY, 1VEZ, 1VF1, 1VF2, 1VF3, 1VF4, 1VF5, 1VF6, 1VF7, 1VF8, 1VFA, 1VFB, 1VFD, 1VFE, 1VFF, 1VFH, 1VFJ, 1VFL, 1VFM, 1VFN, 1VFO, 1VFP, 1VFQ, 1VFR, 1VFS, 1VFT, 1VFU, 1VFV, 1VFW, 1VFX, 1VFY, 1VFZ, 1VG0, 1VG1, 1VG2, 1VG3, 1VG4, 1VG6, 1VG7, 1VG8, 1VG9, 1VGA, 1VGC, 1VGE, 1VGF, 1VGG, 1VGI, 1VGJ, 1VGK, 1VGL, 1VGM, 1VGN, 1VGO, 1VGP, 1VGQ, 1VGR, 1VGT, 1VGU, 1VGV, 1VGW, 1VGX, 1VGY, 1VGZ, 1VH0, 1VH1, 1VH2, 1VH3, 1VH4, 1VH5, 1VH6, 1VH7, 1VH8, 1VH9, 1VHA, 1VHB, 1VHC, 1VHD, 1VHE, 1VHF, 1VHG, 1VHH, 1VHI, 1VHJ, 1VHK, 1VHL, 1VHM, 1VHN, 1VHO, 1VHQ, 1VHR, 1VHS, 1VHT, 1VHU, 1VHV, 1VHW, 1VHX, 1VHY, 1VHZ, 1VI0, 1VI1, 1VI2, 1VI3, 1VI4, 1VI5, 1VI6, 1VI7, 1VI8, 1VI9, 1VIA, 1VIC, 1VID, 1VIE, 1VIF, 1VIJ, 1VIK, 1VIM, 1VIN, 1VIO, 1VIP, 1VIQ, 1VIS, 1VIT, 1VIU, 1VIV, 1VIW, 1VIX, 1VIY, 1VIZ, 1VJ0, 1VJ1, 1VJ2, 1VJ3, 1VJ4, 1VJ5, 1VJ7, 1VJ9, 1VJA, 1VJB, 1VJC, 1VJD, 1VJE, 1VJF, 1VJG, 1VJH, 1VJI, 1VJK, 1VJL, 1VJM, 1VJN, 1VJO, 1VJQ, 1VJR, 1VJS, 1VJT, 1VJU, 1VJV, 1VJW, 1VJX, 1VJY, 1VJZ, 1VK0, 1VK1, 1VK2, 1VK3, 1VK4, 1VK5, 1VK6, 1VK8, 1VK9, 1VKA, 1VKB, 1VKC, 1VKD, 1VKE, 1VKF, 1VKG, 1VKH, 1VKI, 1VKJ, 1VKK, 1VKL, 1VKM, 1VKN, 1VKO, 1VKP, 1VKQ, 1VKU, 1VKW, 1VKX, 1VKY, 1VKZ, 1VL0, 1VL1, 1VL2, 1VL4, 1VL5, 1VL6, 1VL7, 1VL8, 1VL9, 1VLA, 1VLB, 1VLC, 1VLD, 1VLE, 1VLF, 1VLG, 1VLH, 1VLI, 1VLJ, 1VLK, 1VLL, 1VLM, 1VLN, 1VLO, 1VLP, 1VLQ, 1VLR, 1VLS, 1VLT, 1VLU, 1VLV, 1VLW, 1VLX, 1VLY, 1VLZ, 1VM0, 1VM1, 1VM6, 1VM7, 1VM8, 1VM9, 1VMA, 1VMB, 1VMD, 1VME, 1VMF, 1VMG, 1VMH, 1VMI, 1VMJ, 1VMK, 1VMO, 1VNC, 1VNE, 1VNF, 1VNG, 1VNH, 1VNI, 1VNS, 1VOK, 1VOL, 1VOM, 1VOT, 1VP2, 1VP3, 1VP4, 1VP5, 1VP6, 1VP7, 1VP8, 1VP9, 1VPA, 1VPB, 1VPD, 1VPE, 1VPF, 1VPH, 1VPI, 1VPK, 1VPL, 1VPM, 1VPN, 1VPO, 1VPP, 1VPQ, 1VPR, 1VPS, 1VPT, 1VPV, 1VPW, 1VPX, 1VPY, 1VPZ, 1VQ0, 1VQ1, 1VQ2, 1VQ3, 1VQA, 1VQB, 1VQC, 1VQD, 1VQE, 1VQF, 1VQG, 1VQH, 1VQI, 1VQJ, 1VQQ, 1VQR, 1VQS, 1VQT, 1VQU, 1VQV, 1VQW, 1VQY, 1VQZ, 1VR0, 1VR1, 1VR2, 1VR3, 1VR4, 1VR5, 1VR6, 1VR7, 1VR8, 1VR9, 1VRA, 1VRB, 1VRD, 1VRG, 1VRK, 1VRL, 1VRM, 1VRN, 1VRP, 1VRQ, 1VRR, 1VRS, 1VRT, 1VRU, 1VRW, 1VRX, 1VS0, 1VS1, 1VS3, 1VSB, 1VSC, 1VSD, 1VSE, 1VSF, 1VSG, 1VSH, 1VSI, 1VSJ, 1VSK, 1VSL, 1VSM, 1VSN, 1VSO, 1VSR, 1VST, 1VSU, 1VSV, 1VSW, 1VSX, 1VSY, 1VT0, 1VT5, 1VT6, 1VT7, 1VT8, 1VT9, 1VTA, 1VTB, 1VTC, 1VTD, 1VTE, 1VTJ, 1VTK, 1VTL, 1VTN, 1VTO, 1VTR, 1VTT, 1VTU, 1VTY, 1VTZ, 1VU0, 1VU1, 1VU2, 1VU3, 1VUB, 1VW5, 1VW6, 1VWA, 1VWB, 1VWC, 1VWD, 1VWE, 1VWF, 1VWG, 1VWH, 1VWI, 1VWJ, 1VWK, 1VWL, 1VWM, 1VWN, 1VWO, 1VWP, 1VWQ, 1VWR, 1VWT, 1VXA, 1VXB, 1VXC, 1VXD, 1VXE, 1VXF, 1VXG, 1VXH, 1VXO, 1VXR, 1VYB, 1VYD, 1VYF, 1VYG, 1VYH, 1VYI, 1VYJ, 1VYK, 1VYM, 1VYO, 1VYP, 1VYQ, 1VYR, 1VYS, 1VYT, 1VYU, 1VYV, 1VYW, 1VYZ, 1VZ0, 1VZ2, 1VZ3, 1VZ4, 1VZ5, 1VZ6, 1VZ7, 1VZ8, 1VZA, 1VZB, 1VZC, 1VZD, 1VZE, 1VZG, 1VZH, 1VZI, 1VZJ, 1VZK, 1VZM, 1VZO, 1VZQ, 1VZT, 1VZU, 1VZV, 1VZW, 1VZX, 1VZY, 1VZZ, 1W00, 1W01, 1W02, 1W03, 1W04, 1W05, 1W06, 1W07, 1W08, 1W0C, 1W0D, 1W0E, 1W0F, 1W0G, 1W0H, 1W0I, 1W0J, 1W0K, 1W0M, 1W0N, 1W0O, 1W0P, 1W0T, 1W0U, 1W0V, 1W0W, 1W0X, 1W0Y, 1W0Z, 1W10, 1W11, 1W12, 1W13, 1W14, 1W15, 1W16, 1W17, 1W18, 1W19, 1W1A, 1W1B, 1W1D, 1W1G, 1W1H, 1W1I, 1W1J, 1W1K, 1W1L, 1W1M, 1W1O, 1W1P, 1W1Q, 1W1R, 1W1S, 1W1T, 1W1U, 1W1V, 1W1W, 1W1X, 1W1Y, 1W1Z, 1W20, 1W21, 1W22, 1W23, 1W24, 1W25, 1W26, 1W27, 1W28, 1W29, 1W2A, 1W2C, 1W2D, 1W2E, 1W2F, 1W2G, 1W2H, 1W2I, 1W2K, 1W2L, 1W2M, 1W2N, 1W2O, 1W2P, 1W2T, 1W2U, 1W2V, 1W2W, 1W2X, 1W2Y, 1W2Z, 1W30, 1W31, 1W32, 1W33, 1W34, 1W35, 1W36, 1W37, 1W3A, 1W3B, 1W3C, 1W3E, 1W3F, 1W3G, 1W3H, 1W3I, 1W3J, 1W3K, 1W3L, 1W3N, 1W3O, 1W3P, 1W3Q, 1W3R, 1W3S, 1W3T, 1W3U, 1W3V, 1W3W, 1W3X, 1W3Y, 1W3Z, 1W40, 1W41, 1W42, 1W44, 1W45, 1W46, 1W47, 1W48, 1W49, 1W4A, 1W4B, 1W4C, 1W4L, 1W4N, 1W4O, 1W4P, 1W4Q, 1W4R, 1W4S, 1W4T, 1W4V, 1W4W, 1W4X, 1W4Y, 1W4Z, 1W50, 1W51, 1W52, 1W53, 1W54, 1W55, 1W56, 1W57, 1W58, 1W59, 1W5A, 1W5B, 1W5C, 1W5D, 1W5E, 1W5F, 1W5G, 1W5H, 1W5I, 1W5J, 1W5K, 1W5L, 1W5M, 1W5N, 1W5O, 1W5P, 1W5Q, 1W5R, 1W5S, 1W5T, 1W5V, 1W5W, 1W5X, 1W5Y, 1W5Z, 1W60, 1W61, 1W62, 1W63, 1W66, 1W68, 1W69, 1W6C, 1W6F, 1W6G, 1W6H, 1W6I, 1W6J, 1W6K, 1W6L, 1W6M, 1W6N, 1W6O, 1W6P, 1W6Q, 1W6R, 1W6S, 1W6T, 1W6U, 1W6W, 1W6X, 1W6Y, 1W6Z, 1W70, 1W72, 1W73, 1W74, 1W75, 1W76, 1W77, 1W78, 1W79, 1W7A, 1W7B, 1W7C, 1W7F, 1W7G, 1W7H, 1W7I, 1W7J, 1W7K, 1W7L, 1W7M, 1W7N, 1W7O, 1W7P, 1W7S, 1W7T, 1W7U, 1W7V, 1W7W, 1W7X, 1W7Z, 1W80, 1W81, 1W82, 1W83, 1W84, 1W85, 1W87, 1W88, 1W89, 1W8A, 1W8B, 1W8C, 1W8D, 1W8E, 1W8F, 1W8G, 1W8H, 1W8I, 1W8J, 1W8K, 1W8L, 1W8M, 1W8N, 1W8O, 1W8P, 1W8Q, 1W8S, 1W8T, 1W8U, 1W8V, 1W8W, 1W8Y, 1W8Z, 1W90, 1W91, 1W92, 1W93, 1W94, 1W96, 1W97, 1W98, 1W99, 1W9A, 1W9B, 1W9C, 1W9D, 1W9E, 1W9F, 1W9G, 1W9H, 1W9I, 1W9J, 1W9K, 1W9L, 1W9M, 1W9O, 1W9P, 1W9Q, 1W9S, 1W9T, 1W9U, 1W9V, 1W9W, 1W9X, 1W9Y, 1W9Z, 1WA0, 1WA1, 1WA2, 1WA3, 1WA4, 1WA5, 1WA6, 1WA9, 1WAA, 1WAB, 1WAC, 1WAD, 1WAE, 1WAF, 1WAJ, 1WAK, 1WAL, 1WAM, 1WAO, 1WAP, 1WAQ, 1WAR, 1WAS, 1WAT, 1WAU, 1WAV, 1WAW, 1WAX, 1WAY, 1WB0, 1WB4, 1WB5, 1WB6, 1WB7, 1WB8, 1WB9, 1WBA, 1WBB, 1WBC, 1WBD, 1WBE, 1WBF, 1WBG, 1WBH, 1WBI, 1WBJ, 1WBK, 1WBL, 1WBM, 1WBN, 1WBO, 1WBP, 1WBQ, 1WBS, 1WBT, 1WBU, 1WBV, 1WBW, 1WBX, 1WBY, 1WBZ, 1WC0, 1WC1, 1WC2, 1WC3, 1WC4, 1WC5, 1WC6, 1WC7, 1WC8, 1WC9, 1WCB, 1WCC, 1WCE, 1WCF, 1WCG, 1WCH, 1WCI, 1WCK, 1WCM, 1WCQ, 1WCS, 1WCU, 1WCV, 1WCW, 1WCX, 1WCY, 1WCZ, 1WD0, 1WD1, 1WD3, 1WD4, 1WD5, 1WD6, 1WD7, 1WD8, 1WD9, 1WDA, 1WDC, 1WDD, 1WDE, 1WDF, 1WDG, 1WDI, 1WDJ, 1WDK, 1WDL, 1WDM, 1WDN, 1WDP, 1WDQ, 1WDR, 1WDS, 1WDT, 1WDU, 1WDV, 1WDW, 1WDX, 1WDY, 1WDZ, 1WE0, 1WE1, 1WE2, 1WE3, 1WE4, 1WE5, 1WEF, 1WEG, 1WEH, 1WEI, 1WEJ, 1WEK, 1WER, 1WET, 1WF3, 1WF4, 1WFA, 1WFB, 1WFC, 1WFX, 1WG0, 1WG3, 1WG8, 1WGB, 1WGC, 1WGI, 1WGJ, 1WGT, 1WGZ, 1WHI, 1WHO, 1WHP, 1WHS, 1WHT, 1WHZ, 1WIO, 1WIP, 1WIQ, 1WIW, 1WIY, 1WJ9, 1WJG, 1WJX, 1WK2, 1WK4, 1WK8, 1WK9, 1WKA, 1WKB, 1WKC, 1WKD, 1WKE, 1WKF, 1WKG, 1WKH, 1WKJ, 1WKK, 1WKL, 1WKM, 1WKO, 1WKP, 1WKQ, 1WKR, 1WKU, 1WKV, 1WKW, 1WKX, 1WKY, 1WKZ, 1WL0, 1WL1, 1WL2, 1WL3, 1WL4, 1WL5, 1WL6, 1WL7, 1WL8, 1WL9, 1WLA, 1WLC, 1WLD, 1WLE, 1WLF, 1WLG, 1WLH, 1WLI, 1WLJ, 1WLK, 1WLR, 1WLS, 1WLT, 1WLU, 1WLV, 1WLW, 1WLY, 1WLZ, 1WM0, 1WM1, 1WM2, 1WM3, 1WM5, 1WM6, 1WM9, 1WMA, 1WMB, 1WMD, 1WME, 1WMF, 1WMG, 1WMH, 1WMI, 1WMK, 1WMM, 1WMN, 1WMO, 1WMP, 1WMR, 1WMS, 1WMU, 1WMW, 1WMX, 1WMY, 1WMZ, 1WN0, 1WN1, 1WN2, 1WN3, 1WN5, 1WN6, 1WN7, 1WN9, 1WNA, 1WNB, 1WNC, 1WND, 1WNF, 1WNG, 1WNH, 1WNI, 1WNL, 1WNO, 1WNR, 1WNS, 1WNT, 1WNU, 1WNV, 1WNW, 1WNX, 1WNY, 1WNZ, 1WO2, 1WO8, 1WOA, 1WOB, 1WOC, 1WOD, 1WOF, 1WOG, 1WOH, 1WOI, 1WOJ, 1WOK, 1WOL, 1WOM, 1WOO, 1WOP, 1WOQ, 1WOR, 1WOS, 1WOU, 1WOV, 1WOW, 1WOX, 1WOY, 1WOZ, 1WP0, 1WP1, 1WP4, 1WP5, 1WP6, 1WP7, 1WP8, 1WP9, 1WPA, 1WPB, 1WPC, 1WPG, 1WPL, 1WPM, 1WPN, 1WPO, 1WPP, 1WPQ, 1WPR, 1WPS, 1WPT, 1WPV, 1WPW, 1WPX, 1WPY, 1WQ1, 1WQ3, 1WQ4, 1WQ5, 1WQ6, 1WQ7, 1WQ8, 1WQ9, 1WQA, 1WQF, 1WQG, 1WQH, 1WQJ, 1WQL, 1WQM, 1WQN, 1WQO, 1WQP, 1WQQ, 1WQR, 1WQS, 1WQV, 1WQW, 1WQY, 1WR2, 1WR6, 1WR8, 1WRA, 1WRB, 1WRD, 1WRI, 1WRJ, 1WRK, 1WRL, 1WRM, 1WRN, 1WRO, 1WRP, 1WRR, 1WRU, 1WRV, 1WRZ, 1WS0, 1WS1, 1WS2, 1WS3, 1WS4, 1WS5, 1WS6, 1WS7, 1WS8, 1WS9, 1WSA, 1WSB, 1WSC, 1WSD, 1WSE, 1WSF, 1WSG, 1WSH, 1WSI, 1WSJ, 1WSP, 1WSR, 1WSS, 1WST, 1WSV, 1WSW, 1WSZ, 1WT0, 1WT1, 1WT2, 1WT3, 1WT5, 1WT6, 1WT9, 1WTA, 1WTC, 1WTD, 1WTE, 1WTF, 1WTG, 1WTH, 1WTJ, 1WTL, 1WTM, 1WTN, 1WTO, 1WTP, 1WTQ, 1WTR, 1WTV, 1WTW, 1WTX, 1WTY, 1WU1, 1WU2, 1WU3, 1WU4, 1WU5, 1WU6, 1WU7, 1WU8, 1WU9, 1WUA, 1WUB, 1WUD, 1WUE, 1WUF, 1WUH, 1WUI, 1WUJ, 1WUK, 1WUL, 1WUN, 1WUO, 1WUP, 1WUQ, 1WUR, 1WUT, 1WUU, 1WUV, 1WUW, 1WUY, 1WV0, 1WV1, 1WV2, 1WV3, 1WV4, 1WV5, 1WV6, 1WV7, 1WV8, 1WV9, 1WVA, 1WVB, 1WVC, 1WVE, 1WVF, 1WVG, 1WVH, 1WVI, 1WVJ, 1WVL, 1WVM, 1WVN, 1WVP, 1WVQ, 1WVR, 1WVT, 1WVU, 1WVV, 1WVW, 1WVX, 1WVY, 1WW1, 1WW2, 1WW3, 1WW4, 1WW5, 1WW6, 1WW7, 1WW8, 1WW9, 1WWA, 1WWB, 1WWC, 1WWH, 1WWI, 1WWJ, 1WWK, 1WWL, 1WWM, 1WWP, 1WWR, 1WWS, 1WWW, 1WWZ, 1WX0, 1WX1, 1WX2, 1WX4, 1WX5, 1WXC, 1WXD, 1WXE, 1WXF, 1WXG, 1WXH, 1WXI, 1WXJ, 1WXO, 1WXQ, 1WXR, 1WXW, 1WXX, 1WXY, 1WXZ, 1WY0, 1WY1, 1WY2, 1WY3, 1WY4, 1WY5, 1WY6, 1WY7, 1WY9, 1WYB, 1WYC, 1WYD, 1WYE, 1WYG, 1WYI, 1WYK, 1WYT, 1WYU, 1WYV, 1WYW, 1WYX, 1WYY, 1WYZ, 1WZ1, 1WZ3, 1WZ7, 1WZ8, 1WZ9, 1WZA, 1WZC, 1WZD, 1WZE, 1WZF, 1WZG, 1WZI, 1WZK, 1WZL, 1WZM, 1WZN, 1WZO, 1WZU, 1WZV, 1WZW, 1WZX, 1WZY, 1WZZ, 1X01, 1X03, 1X04, 1X06, 1X07, 1X08, 1X09, 1X0A, 1X0C, 1X0G, 1X0I, 1X0J, 1X0K, 1X0L, 1X0M, 1X0P, 1X0R, 1X0S, 1X0T, 1X0U, 1X0V, 1X0X, 1X10, 1X11, 1X12, 1X13, 1X14, 1X15, 1X19, 1X1A, 1X1B, 1X1C, 1X1D, 1X1E, 1X1H, 1X1I, 1X1J, 1X1K, 1X1N, 1X1O, 1X1P, 1X1Q, 1X1R, 1X1S, 1X1T, 1X1U, 1X1V, 1X1W, 1X1X, 1X1Y, 1X1Z, 1X23, 1X24, 1X25, 1X27, 1X28, 1X29, 1X2A, 1X2B, 1X2E, 1X2G, 1X2H, 1X2I, 1X2J, 1X2R, 1X2T, 1X2W, 1X31, 1X36, 1X38, 1X39, 1X3E, 1X3F, 1X3G, 1X3K, 1X3L, 1X3M, 1X3N, 1X3O, 1X3S, 1X3W, 1X3X, 1X3Z, 1X42, 1X46, 1X54, 1X55, 1X56, 1X6I, 1X6J, 1X6L, 1X6M, 1X6N, 1X6O, 1X6P, 1X6Q, 1X6R, 1X6U, 1X6V, 1X6X, 1X6Y, 1X6Z, 1X70, 1X71, 1X74, 1X75, 1X76, 1X77, 1X78, 1X79, 1X7A, 1X7B, 1X7D, 1X7E, 1X7F, 1X7G, 1X7H, 1X7I, 1X7J, 1X7N, 1X7O, 1X7P, 1X7Q, 1X7R, 1X7S, 1X7T, 1X7U, 1X7V, 1X7W, 1X7X, 1X7Y, 1X7Z, 1X80, 1X81, 1X82, 1X83, 1X84, 1X86, 1X87, 1X88, 1X89, 1X8B, 1X8C, 1X8D, 1X8E, 1X8F, 1X8G, 1X8H, 1X8I, 1X8J, 1X8K, 1X8L, 1X8M, 1X8N, 1X8O, 1X8P, 1X8Q, 1X8R, 1X8S, 1X8T, 1X8U, 1X8V, 1X8X, 1X8Y, 1X8Z, 1X90, 1X91, 1X92, 1X94, 1X96, 1X97, 1X98, 1X99, 1X9D, 1X9E, 1X9F, 1X9G, 1X9H, 1X9I, 1X9J, 1X9M, 1X9N, 1X9Q, 1X9R, 1X9S, 1X9U, 1X9W, 1X9Y, 1X9Z, 1XA0, 1XA1, 1XA2, 1XA3, 1XA4, 1XA5, 1XA6, 1XA7, 1XA8, 1XA9, 1XAA, 1XAB, 1XAC, 1XAD, 1XAE, 1XAF, 1XAG, 1XAH, 1XAI, 1XAJ, 1XAK, 1XAL, 1XAM, 1XAN, 1XAO, 1XAP, 1XAR, 1XAS, 1XAT, 1XAU, 1XAW, 1XB0, 1XB1, 1XB2, 1XB3, 1XB4, 1XB6, 1XB7, 1XB8, 1XB9, 1XBA, 1XBB, 1XBC, 1XBF, 1XBI, 1XBN, 1XBO, 1XBR, 1XBS, 1XBT, 1XBU, 1XBV, 1XBW, 1XBX, 1XBY, 1XBZ, 1XC1, 1XC3, 1XC4, 1XC6, 1XC7, 1XC8, 1XC9, 1XCA, 1XCB, 1XCC, 1XCD, 1XCF, 1XCG, 1XCH, 1XCJ, 1XCK, 1XCL, 1XCM, 1XCO, 1XCP, 1XCQ, 1XCR, 1XCS, 1XCT, 1XCU, 1XCV, 1XCW, 1XCX, 1XD0, 1XD1, 1XD2, 1XD3, 1XD4, 1XD5, 1XD6, 1XD7, 1XD8, 1XD9, 1XDA, 1XDB, 1XDC, 1XDD, 1XDF, 1XDG, 1XDH, 1XDI, 1XDJ, 1XDK, 1XDL, 1XDM, 1XDN, 1XDO, 1XDP, 1XDQ, 1XDS, 1XDT, 1XDU, 1XDV, 1XDW, 1XDY, 1XDZ, 1XE0, 1XE1, 1XE3, 1XE4, 1XE5, 1XE6, 1XE7, 1XE8, 1XEA, 1XEB, 1XEC, 1XED, 1XEF, 1XEG, 1XEI, 1XEJ, 1XEK, 1XEL, 1XEM, 1XEN, 1XEO, 1XEP, 1XEQ, 1XER, 1XES, 1XET, 1XEU, 1XEV, 1XEW, 1XEX, 1XEY, 1XEZ, 1XF0, 1XF1, 1XF2, 1XF3, 1XF4, 1XF5, 1XF6, 1XF8, 1XF9, 1XFA, 1XFB, 1XFC, 1XFD, 1XFF, 1XFG, 1XFH, 1XFI, 1XFJ, 1XFK, 1XFO, 1XFP, 1XFS, 1XFU, 1XFV, 1XFW, 1XFX, 1XFY, 1XFZ, 1XG0, 1XG2, 1XG3, 1XG4, 1XG5, 1XG6, 1XG7, 1XG8, 1XGD, 1XGE, 1XGF, 1XGI, 1XGJ, 1XGK, 1XGM, 1XGN, 1XGO, 1XGP, 1XGQ, 1XGR, 1XGS, 1XGT, 1XGU, 1XGV, 1XGW, 1XGY, 1XGZ, 1XH0, 1XH1, 1XH2, 1XH3, 1XH4, 1XH5, 1XH6, 1XH7, 1XH8, 1XH9, 1XHA, 1XHB, 1XHC, 1XHD, 1XHE, 1XHF, 1XHG, 1XHK, 1XHL, 1XHM, 1XHN, 1XHO, 1XHU, 1XHV, 1XHX, 1XHY, 1XHZ, 1XI0, 1XI1, 1XI2, 1XI3, 1XI6, 1XI8, 1XI9, 1XIA, 1XIB, 1XIC, 1XID, 1XIE, 1XIF, 1XIG, 1XIH, 1XII, 1XIJ, 1XIK, 1XIL, 1XIM, 1XIN, 1XIO, 1XIP, 1XIQ, 1XIS, 1XIU, 1XIV, 1XIW, 1XIX, 1XIY, 1XIZ, 1XJ0, 1XJ2, 1XJ3, 1XJ4, 1XJ5, 1XJ6, 1XJ7, 1XJA, 1XJB, 1XJC, 1XJD, 1XJE, 1XJF, 1XJG, 1XJI, 1XJJ, 1XJK, 1XJL, 1XJM, 1XJN, 1XJO, 1XJQ, 1XJT, 1XJU, 1XJV, 1XJW, 1XJX, 1XJY, 1XJZ, 1XK0, 1XK1, 1XK2, 1XK3, 1XK4, 1XK5, 1XK6, 1XK7, 1XK8, 1XK9, 1XKA, 1XKB, 1XKD, 1XKF, 1XKG, 1XKH, 1XKI, 1XKJ, 1XKK, 1XKL, 1XKN, 1XKO, 1XKP, 1XKQ, 1XKR, 1XKS, 1XKT, 1XKU, 1XKV, 1XKW, 1XKX, 1XKY, 1XKZ, 1XL0, 1XL1, 1XL2, 1XL3, 1XL4, 1XL5, 1XL6, 1XL7, 1XL8, 1XL9, 1XLA, 1XLB, 1XLC, 1XLD, 1XLE, 1XLF, 1XLG, 1XLH, 1XLI, 1XLJ, 1XLK, 1XLL, 1XLM, 1XLN, 1XLO, 1XLP, 1XLQ, 1XLR, 1XLS, 1XLT, 1XLU, 1XLV, 1XLW, 1XLX, 1XLY, 1XLZ, 1XM1, 1XM2, 1XM3, 1XM4, 1XM5, 1XM6, 1XM7, 1XM8, 1XM9, 1XMA, 1XMB, 1XMC, 1XMD, 1XME, 1XMF, 1XMG, 1XMH, 1XMI, 1XMJ, 1XMK, 1XML, 1XMM, 1XMN, 1XMP, 1XMS, 1XMT, 1XMU, 1XMV, 1XMX, 1XMY, 1XMZ, 1XN0, 1XN1, 1XN2, 1XN3, 1XN4, 1XNB, 1XNC, 1XND, 1XNF, 1XNG, 1XNH, 1XNI, 1XNJ, 1XNK, 1XNN, 1XNS, 1XNV, 1XNW, 1XNX, 1XNY, 1XNZ, 1XO0, 1XO1, 1XO2, 1XO5, 1XO6, 1XO7, 1XOC, 1XOD, 1XOE, 1XOF, 1XOG, 1XOI, 1XOM, 1XON, 1XOQ, 1XOR, 1XOS, 1XOT, 1XOU, 1XOV, 1XOW, 1XOZ, 1XP0, 1XP1, 1XP3, 1XP4, 1XP5, 1XP6, 1XP8, 1XP9, 1XPB, 1XPC, 1XPG, 1XPH, 1XPI, 1XPJ, 1XPK, 1XPL, 1XPM, 1XPP, 1XPQ, 1XPS, 1XPT, 1XPX, 1XPY, 1XPZ, 1XQ0, 1XQ1, 1XQ3, 1XQ4, 1XQ5, 1XQ6, 1XQ7, 1XQ9, 1XQA, 1XQB, 1XQC, 1XQD, 1XQE, 1XQF, 1XQG, 1XQH, 1XQI, 1XQJ, 1XQK, 1XQL, 1XQM, 1XQO, 1XQP, 1XQR, 1XQS, 1XQU, 1XQV, 1XQW, 1XQX, 1XQY, 1XQZ, 1XR1, 1XR2, 1XR3, 1XR4, 1XR5, 1XR6, 1XR7, 1XR8, 1XR9, 1XRA, 1XRB, 1XRC, 1XRE, 1XRF, 1XRG, 1XRH, 1XRI, 1XRJ, 1XRK, 1XRL, 1XRM, 1XRN, 1XRO, 1XRP, 1XRQ, 1XRR, 1XRS, 1XRT, 1XRU, 1XRV, 1XRX, 1XRY, 1XS0, 1XS1, 1XS2, 1XS4, 1XS5, 1XS6, 1XS7, 1XSD, 1XSE, 1XSI, 1XSJ, 1XSK, 1XSL, 1XSM, 1XSN, 1XSO, 1XSP, 1XSQ, 1XSR, 1XSS, 1XSV, 1XSZ, 1XT0, 1XT3, 1XT4, 1XT5, 1XT6, 1XT8, 1XT9, 1XTA, 1XTB, 1XTC, 1XTD, 1XTE, 1XTF, 1XTG, 1XTI, 1XTJ, 1XTK, 1XTL, 1XTM, 1XTN, 1XTO, 1XTP, 1XTQ, 1XTR, 1XTS, 1XTT, 1XTU, 1XTV, 1XTY, 1XTZ, 1XU1, 1XU2, 1XU3, 1XU4, 1XU5, 1XU7, 1XU8, 1XU9, 1XUA, 1XUB, 1XUC, 1XUD, 1XUF, 1XUG, 1XUH, 1XUI, 1XUJ, 1XUK, 1XUO, 1XUP, 1XUQ, 1XUR, 1XUU, 1XUV, 1XUW, 1XUX, 1XUZ, 1XV2, 1XV5, 1XV8, 1XV9, 1XVA, 1XVB, 1XVC, 1XVD, 1XVE, 1XVF, 1XVG, 1XVI, 1XVJ, 1XVL, 1XVM, 1XVO, 1XVP, 1XVQ, 1XVS, 1XVT, 1XVU, 1XVV, 1XVW, 1XVX, 1XVY, 1XW2, 1XW3, 1XW4, 1XW5, 1XW6, 1XW7, 1XW8, 1XW9, 1XWA, 1XWB, 1XWC, 1XWD, 1XWF, 1XWG, 1XWI, 1XWJ, 1XWK, 1XWL, 1XWM, 1XWO, 1XWQ, 1XWR, 1XWS, 1XWT, 1XWV, 1XWW, 1XWY, 1XX1, 1XX2, 1XX4, 1XX5, 1XX6, 1XX7, 1XX9, 1XXA, 1XXB, 1XXC, 1XXD, 1XXF, 1XXG, 1XXH, 1XXI, 1XXJ, 1XXL, 1XXM, 1XXN, 1XXO, 1XXP, 1XXQ, 1XXR, 1XXS, 1XXT, 1XXU, 1XXV, 1XXW, 1XXX, 1XY0, 1XY1, 1XY2, 1XY3, 1XY7, 1XYA, 1XYB, 1XYC, 1XYE, 1XYF, 1XYG, 1XYH, 1XYI, 1XYL, 1XYM, 1XYN, 1XYO, 1XYP, 1XYS, 1XYV, 1XYY, 1XYZ, 1XZ0, 1XZ1, 1XZ2, 1XZ3, 1XZ4, 1XZ5, 1XZ6, 1XZ7, 1XZ8, 1XZA, 1XZB, 1XZC, 1XZD, 1XZE, 1XZF, 1XZG, 1XZH, 1XZI, 1XZJ, 1XZK, 1XZL, 1XZM, 1XZN, 1XZO, 1XZP, 1XZQ, 1XZU, 1XZV, 1XZW, 1XZX, 1XZZ, 1Y01, 1Y02, 1Y07, 1Y08, 1Y09, 1Y0A, 1Y0B, 1Y0C, 1Y0D, 1Y0E, 1Y0G, 1Y0H, 1Y0K, 1Y0L, 1Y0M, 1Y0N, 1Y0O, 1Y0P, 1Y0R, 1Y0S, 1Y0T, 1Y0U, 1Y0V, 1Y0W, 1Y0X, 1Y0Y, 1Y0Z, 1Y10, 1Y11, 1Y12, 1Y13, 1Y14, 1Y17, 1Y18, 1Y19, 1Y1A, 1Y1D, 1Y1E, 1Y1F, 1Y1G, 1Y1H, 1Y1I, 1Y1J, 1Y1K, 1Y1L, 1Y1M, 1Y1N, 1Y1O, 1Y1P, 1Y1Q, 1Y1R, 1Y1S, 1Y1T, 1Y1U, 1Y1V, 1Y1X, 1Y1Y, 1Y1Z, 1Y20, 1Y21, 1Y22, 1Y23, 1Y25, 1Y28, 1Y2A, 1Y2B, 1Y2C, 1Y2D, 1Y2E, 1Y2F, 1Y2G, 1Y2H, 1Y2I, 1Y2J, 1Y2K, 1Y2M, 1Y2O, 1Y2Q, 1Y2T, 1Y2U, 1Y2V, 1Y2W, 1Y2X, 1Y2Z, 1Y30, 1Y31, 1Y33, 1Y34, 1Y35, 1Y37, 1Y38, 1Y3A, 1Y3B, 1Y3C, 1Y3D, 1Y3F, 1Y3G, 1Y3H, 1Y3I, 1Y3N, 1Y3P, 1Y3Q, 1Y3T, 1Y3U, 1Y3V, 1Y3W, 1Y3X, 1Y3Y, 1Y42, 1Y43, 1Y44, 1Y45, 1Y46, 1Y47, 1Y48, 1Y4A, 1Y4B, 1Y4C, 1Y4D, 1Y4F, 1Y4G, 1Y4H, 1Y4I, 1Y4J, 1Y4K, 1Y4L, 1Y4M, 1Y4P, 1Y4Q, 1Y4R, 1Y4S, 1Y4T, 1Y4U, 1Y4V, 1Y4W, 1Y4Y, 1Y4Z, 1Y50, 1Y51, 1Y52, 1Y53, 1Y54, 1Y55, 1Y56, 1Y57, 1Y59, 1Y5A, 1Y5B, 1Y5E, 1Y5F, 1Y5H, 1Y5I, 1Y5J, 1Y5K, 1Y5L, 1Y5M, 1Y5N, 1Y5R, 1Y5U, 1Y5V, 1Y5W, 1Y5X, 1Y5Y, 1Y60, 1Y62, 1Y63, 1Y64, 1Y65, 1Y66, 1Y67, 1Y6A, 1Y6B, 1Y6E, 1Y6F, 1Y6G, 1Y6H, 1Y6I, 1Y6J, 1Y6K, 1Y6L, 1Y6M, 1Y6N, 1Y6O, 1Y6P, 1Y6Q, 1Y6R, 1Y6V, 1Y6W, 1Y6X, 1Y6Z, 1Y71, 1Y75, 1Y79, 1Y7A, 1Y7B, 1Y7C, 1Y7D, 1Y7E, 1Y7F, 1Y7G, 1Y7H, 1Y7I, 1Y7L, 1Y7M, 1Y7O, 1Y7P, 1Y7R, 1Y7T, 1Y7U, 1Y7V, 1Y7W, 1Y7Y, 1Y7Z, 1Y80, 1Y81, 1Y82, 1Y83, 1Y84, 1Y85, 1Y86, 1Y88, 1Y89, 1Y8A, 1Y8C, 1Y8E, 1Y8G, 1Y8H, 1Y8I, 1Y8J, 1Y8K, 1Y8L, 1Y8N, 1Y8O, 1Y8P, 1Y8Q, 1Y8R, 1Y8T, 1Y8V, 1Y8W, 1Y8X, 1Y8Y, 1Y8Z, 1Y91, 1Y92, 1Y93, 1Y94, 1Y96, 1Y97, 1Y98, 1Y9A, 1Y9B, 1Y9D, 1Y9E, 1Y9F, 1Y9G, 1Y9I, 1Y9K, 1Y9L, 1Y9M, 1Y9Q, 1Y9R, 1Y9S, 1Y9T, 1Y9U, 1Y9W, 1Y9Z, 1YA0, 1YA3, 1YA4, 1YA5, 1YA6, 1YA7, 1YA8, 1YA9, 1YAA, 1YAB, 1YAC, 1YAD, 1YAE, 1YAF, 1YAG, 1YAH, 1YAI, 1YAJ, 1YAK, 1YAL, 1YAM, 1YAN, 1YAO, 1YAP, 1YAQ, 1YAR, 1YAS, 1YAT, 1YAU, 1YAV, 1YAX, 1YAZ, 1YB0, 1YB1, 1YB2, 1YB3, 1YB4, 1YB5, 1YB6, 1YB7, 1YB9, 1YBA, 1YBC, 1YBD, 1YBE, 1YBF, 1YBG, 1YBH, 1YBI, 1YBK, 1YBM, 1YBO, 1YBQ, 1YBT, 1YBU, 1YBV, 1YBW, 1YBX, 1YBY, 1YBZ, 1YC0, 1YC1, 1YC2, 1YC3, 1YC4, 1YC5, 1YC6, 1YC7, 1YC8, 1YC9, 1YCA, 1YCB, 1YCC, 1YCD, 1YCE, 1YCF, 1YCG, 1YCH, 1YCI, 1YCJ, 1YCK, 1YCL, 1YCN, 1YCO, 1YCP, 1YCQ, 1YCR, 1YCS, 1YCY, 1YCZ, 1YD0, 1YD1, 1YD2, 1YD3, 1YD4, 1YD5, 1YD6, 1YD7, 1YD8, 1YD9, 1YDA, 1YDB, 1YDC, 1YDD, 1YDE, 1YDF, 1YDG, 1YDH, 1YDI, 1YDK, 1YDL, 1YDM, 1YDN, 1YDO, 1YDP, 1YDR, 1YDS, 1YDT, 1YDV, 1YDW, 1YDX, 1YDY, 1YDZ, 1YE0, 1YE1, 1YE2, 1YE3, 1YE4, 1YE5, 1YE6, 1YE8, 1YE9, 1YEA, 1YEB, 1YEC, 1YED, 1YEE, 1YEF, 1YEG, 1YEH, 1YEI, 1YEJ, 1YEK, 1YEM, 1YEN, 1YEO, 1YEP, 1YEQ, 1YER, 1YES, 1YET, 1YEU, 1YEV, 1YEW, 1YEX, 1YEY, 1YF0, 1YF1, 1YF2, 1YF3, 1YF4, 1YF5, 1YF6, 1YF8, 1YF9, 1YFD, 1YFE, 1YFF, 1YFH, 1YFI, 1YFJ, 1YFK, 1YFL, 1YFM, 1YFN, 1YFO, 1YFP, 1YFQ, 1YFR, 1YFS, 1YFT, 1YFU, 1YFW, 1YFX, 1YFY, 1YFZ, 1YG2, 1YG5, 1YG6, 1YG8, 1YG9, 1YGA, 1YGB, 1YGC, 1YGD, 1YGE, 1YGF, 1YGG, 1YGH, 1YGJ, 1YGK, 1YGP, 1YGR, 1YGS, 1YGT, 1YGU, 1YGY, 1YGZ, 1YH2, 1YH3, 1YH8, 1YH9, 1YHA, 1YHB, 1YHC, 1YHE, 1YHF, 1YHG, 1YHH, 1YHI, 1YHJ, 1YHK, 1YHL, 1YHM, 1YHN, 1YHR, 1YHS, 1YHT, 1YHU, 1YHV, 1YHW, 1YHY, 1YHZ, 1YI0, 1YI1, 1YI3, 1YI4, 1YI5, 1YI6, 1YI7, 1YI8, 1YI9, 1YIA, 1YIB, 1YID, 1YIE, 1YIF, 1YIG, 1YIH, 1YII, 1YIK, 1YIL, 1YIM, 1YIN, 1YIO, 1YIP, 1YIQ, 1YIR, 1YIS, 1YIV, 1YIW, 1YIX, 1YIY, 1YIZ, 1YJ0, 1YJ1, 1YJ2, 1YJ3, 1YJ4, 1YJ5, 1YJ6, 1YJ7, 1YJ8, 1YJA, 1YJB, 1YJC, 1YJD, 1YJE, 1YJF, 1YJG, 1YJH, 1YJK, 1YJL, 1YJM, 1YJO, 1YJP, 1YJQ, 1YJS, 1YJX, 1YJY, 1YJZ, 1YK0, 1YK1, 1YK3, 1YK4, 1YK5, 1YK7, 1YK8, 1YK9, 1YKB, 1YKC, 1YKD, 1YKE, 1YKF, 1YKH, 1YKI, 1YKJ, 1YKK, 1YKL, 1YKM, 1YKN, 1YKO, 1YKP, 1YKR, 1YKS, 1YKT, 1YKW, 1YKX, 1YKY, 1YKZ, 1YL0, 1YL1, 1YL5, 1YL6, 1YL7, 1YLA, 1YLC, 1YLD, 1YLE, 1YLF, 1YLH, 1YLI, 1YLJ, 1YLK, 1YLL, 1YLM, 1YLN, 1YLO, 1YLP, 1YLQ, 1YLR, 1YLT, 1YLU, 1YLV, 1YLW, 1YLX, 1YLY, 1YLZ, 1YM0, 1YM1, 1YM2, 1YM3, 1YM4, 1YM5, 1YM7, 1YM9, 1YMA, 1YMB, 1YMC, 1YMD, 1YME, 1YMF, 1YMG, 1YMH, 1YMK, 1YML, 1YMM, 1YMN, 1YMP, 1YMQ, 1YMR, 1YMS, 1YMT, 1YMU, 1YMV, 1YMW, 1YMX, 1YMY, 1YN3, 1YN4, 1YN5, 1YN6, 1YN7, 1YN8, 1YN9, 1YNA, 1YNB, 1YND, 1YNF, 1YNH, 1YNI, 1YNJ, 1YNK, 1YNL, 1YNM, 1YNN, 1YNO, 1YNP, 1YNQ, 1YNR, 1YNS, 1YNT, 1YNU, 1YNV, 1YNW, 1YNY, 1YNZ, 1YO0, 1YO1, 1YO2, 1YO3, 1YO5, 1YO6, 1YO7, 1YO8, 1YOA, 1YOB, 1YOC, 1YOD, 1YOE, 1YOG, 1YOH, 1YOI, 1YOJ, 1YOK, 1YOL, 1YOM, 1YON, 1YOO, 1YOU, 1YOV, 1YOW, 1YOX, 1YOY, 1YOZ, 1YP0, 1YP1, 1YP2, 1YP3, 1YP4, 1YP5, 1YP6, 1YP7, 1YP9, 1YPA, 1YPB, 1YPC, 1YPE, 1YPF, 1YPG, 1YPH, 1YPI, 1YPJ, 1YPK, 1YPL, 1YPM, 1YPN, 1YPO, 1YPP, 1YPQ, 1YPR, 1YPT, 1YPU, 1YPV, 1YPX, 1YPY, 1YPZ, 1YQ1, 1YQ2, 1YQ3, 1YQ4, 1YQ5, 1YQ6, 1YQ7, 1YQ8, 1YQ9, 1YQB, 1YQC, 1YQD, 1YQE, 1YQF, 1YQG, 1YQH, 1YQJ, 1YQK, 1YQL, 1YQM, 1YQN, 1YQO, 1YQP, 1YQQ, 1YQR, 1YQS, 1YQT, 1YQU, 1YQV, 1YQW, 1YQX, 1YQY, 1YQZ, 1YR0, 1YR2, 1YR3, 1YR5, 1YR6, 1YR7, 1YR8, 1YR9, 1YRA, 1YRB, 1YRC, 1YRD, 1YRE, 1YRF, 1YRG, 1YRH, 1YRI, 1YRK, 1YRL, 1YRN, 1YRO, 1YRP, 1YRQ, 1YRR, 1YRS, 1YRT, 1YRU, 1YRV, 1YRW, 1YRX, 1YRY, 1YRZ, 1YS0, 1YS1, 1YS2, 1YS3, 1YS4, 1YS6, 1YS7, 1YS9, 1YSA, 1YSB, 1YSC, 1YSD, 1YSJ, 1YSL, 1YSO, 1YSP, 1YSQ, 1YSR, 1YST, 1YSZ, 1YT0, 1YT1, 1YT2, 1YT3, 1YT4, 1YT5, 1YT7, 1YT8, 1YT9, 1YTA, 1YTB, 1YTC, 1YTD, 1YTE, 1YTF, 1YTG, 1YTH, 1YTI, 1YTJ, 1YTK, 1YTL, 1YTM, 1YTN, 1YTO, 1YTQ, 1YTS, 1YTT, 1YTV, 1YTW, 1YTZ, 1YU0, 1YU1, 1YU2, 1YU3, 1YU4, 1YU5, 1YU6, 1YU7, 1YU8, 1YU9, 1YUC, 1YUD, 1YUE, 1YUH, 1YUK, 1YUL, 1YUM, 1YUN, 1YUO, 1YUP, 1YUW, 1YUX, 1YUY, 1YUZ, 1YV0, 1YV1, 1YV2, 1YV3, 1YV4, 1YV5, 1YV6, 1YV7, 1YV9, 1YVB, 1YVD, 1YVE, 1YVF, 1YVG, 1YVH, 1YVI, 1YVJ, 1YVK, 1YVL, 1YVM, 1YVN, 1YVO, 1YVQ, 1YVR, 1YVS, 1YVT, 1YVU, 1YVW, 1YVX, 1YVY, 1YVZ, 1YW0, 1YW1, 1YW2, 1YW4, 1YW5, 1YW6, 1YW7, 1YW8, 1YW9, 1YWA, 1YWB, 1YWC, 1YWD, 1YWF, 1YWG, 1YWH, 1YWK, 1YWM, 1YWN, 1YWO, 1YWP, 1YWQ, 1YWR, 1YWT, 1YWV, 1YX1, 1YX2, 1YX9, 1YXA, 1YXB, 1YXC, 1YXD, 1YXH, 1YXI, 1YXJ, 1YXK, 1YXL, 1YXM, 1YXO, 1YXQ, 1YXS, 1YXT, 1YXU, 1YXV, 1YXW, 1YXX, 1YXY, 1YY3, 1YY4, 1YY5, 1YY6, 1YY7, 1YY8, 1YY9, 1YYA, 1YYD, 1YYE, 1YYF, 1YYG, 1YYH, 1YYL, 1YYM, 1YYN, 1YYP, 1YYQ, 1YYR, 1YYS, 1YYT, 1YYU, 1YYV, 1YYY, 1YYZ, 1YZ0, 1YZ1, 1YZ3, 1YZ4, 1YZ5, 1YZ6, 1YZ7, 1YZE, 1YZF, 1YZG, 1YZH, 1YZI, 1YZK, 1YZL, 1YZM, 1YZN, 1YZP, 1YZQ, 1YZR, 1YZT, 1YZU, 1YZV, 1YZW, 1YZX, 1YZY, 1YZZ, 1Z01, 1Z02, 1Z03, 1Z05, 1Z06, 1Z07, 1Z08, 1Z0A, 1Z0B, 1Z0C, 1Z0D, 1Z0E, 1Z0F, 1Z0G, 1Z0H, 1Z0I, 1Z0J, 1Z0K, 1Z0M, 1Z0N, 1Z0P, 1Z0S, 1Z0T, 1Z0U, 1Z0V, 1Z0W, 1Z0X, 1Z0Z, 1Z10, 1Z11, 1Z12, 1Z13, 1Z15, 1Z16, 1Z17, 1Z18, 1Z19, 1Z1A, 1Z1B, 1Z1E, 1Z1F, 1Z1G, 1Z1H, 1Z1I, 1Z1J, 1Z1L, 1Z1N, 1Z1P, 1Z1Q, 1Z1R, 1Z1S, 1Z1W, 1Z1X, 1Z1Y, 1Z21, 1Z22, 1Z24, 1Z25, 1Z26, 1Z27, 1Z28, 1Z29, 1Z2A, 1Z2B, 1Z2C, 1Z2I, 1Z2L, 1Z2M, 1Z2N, 1Z2O, 1Z2P, 1Z2U, 1Z2V, 1Z2W, 1Z2X, 1Z2Z, 1Z32, 1Z33, 1Z34, 1Z35, 1Z36, 1Z37, 1Z38, 1Z39, 1Z3A, 1Z3C, 1Z3D, 1Z3E, 1Z3F, 1Z3G, 1Z3H, 1Z3I, 1Z3L, 1Z3M, 1Z3N, 1Z3P, 1Z3Q, 1Z3S, 1Z3T, 1Z3U, 1Z3V, 1Z3W, 1Z3X, 1Z3Y, 1Z3Z, 1Z40, 1Z41, 1Z42, 1Z44, 1Z45, 1Z47, 1Z48, 1Z4A, 1Z4E, 1Z4I, 1Z4J, 1Z4K, 1Z4L, 1Z4M, 1Z4N, 1Z4O, 1Z4P, 1Z4Q, 1Z4R, 1Z4S, 1Z4U, 1Z4V, 1Z4W, 1Z4X, 1Z4Y, 1Z4Z, 1Z50, 1Z52, 1Z53, 1Z54, 1Z55, 1Z56, 1Z57, 1Z59, 1Z5A, 1Z5B, 1Z5C, 1Z5G, 1Z5H, 1Z5L, 1Z5M, 1Z5N, 1Z5O, 1Z5P, 1Z5R, 1Z5S, 1Z5U, 1Z5V, 1Z5W, 1Z5X, 1Z5Y, 1Z5Z, 1Z62, 1Z63, 1Z67, 1Z68, 1Z69, 1Z6A, 1Z6B, 1Z6D, 1Z6E, 1Z6F, 1Z6G, 1Z6I, 1Z6J, 1Z6K, 1Z6L, 1Z6M, 1Z6N, 1Z6O, 1Z6P, 1Z6Q, 1Z6R, 1Z6S, 1Z6T, 1Z6U, 1Z6X, 1Z6Y, 1Z6Z, 1Z70, 1Z71, 1Z72, 1Z73, 1Z74, 1Z75, 1Z76, 1Z77, 1Z78, 1Z7A, 1Z7B, 1Z7C, 1Z7D, 1Z7E, 1Z7G, 1Z7H, 1Z7I, 1Z7J, 1Z7K, 1Z7L, 1Z7M, 1Z7N, 1Z7Q, 1Z7U, 1Z7W, 1Z7X, 1Z7Y, 1Z81, 1Z82, 1Z83, 1Z84, 1Z85, 1Z88, 1Z89, 1Z8A, 1Z8C, 1Z8D, 1Z8F, 1Z8G, 1Z8H, 1Z8I, 1Z8J, 1Z8K, 1Z8L, 1Z8N, 1Z8O, 1Z8P, 1Z8Q, 1Z8T, 1Z8U, 1Z8V, 1Z8W, 1Z8X, 1Z90, 1Z91, 1Z92, 1Z93, 1Z94, 1Z95, 1Z96, 1Z97, 1Z98, 1Z9A, 1Z9C, 1Z9D, 1Z9F, 1Z9G, 1Z9H, 1Z9J, 1Z9K, 1Z9L, 1Z9M, 1Z9N, 1Z9O, 1Z9P, 1Z9S, 1Z9T, 1Z9U, 1Z9W, 1Z9X, 1Z9Y, 1Z9Z, 1ZA0, 1ZA1, 1ZA2, 1ZA3, 1ZA4, 1ZA5, 1ZA6, 1ZA7, 1ZAA, 1ZAB, 1ZAF, 1ZAG, 1ZAH, 1ZAI, 1ZAJ, 1ZAK, 1ZAL, 1ZAN, 1ZAO, 1ZAP, 1ZAR, 1ZAT, 1ZAU, 1ZAV, 1ZAW, 1ZAX, 1ZAY, 1ZB1, 1ZB5, 1ZB6, 1ZB7, 1ZB8, 1ZB9, 1ZBB, 1ZBC, 1ZBD, 1ZBF, 1ZBG, 1ZBK, 1ZBM, 1ZBO, 1ZBP, 1ZBQ, 1ZBR, 1ZBS, 1ZBT, 1ZBU, 1ZBV, 1ZBW, 1ZBX, 1ZBY, 1ZBZ, 1ZC0, 1ZC2, 1ZC3, 1ZC4, 1ZC6, 1ZC9, 1ZCA, 1ZCB, 1ZCC, 1ZCD, 1ZCE, 1ZCF, 1ZCH, 1ZCJ, 1ZCK, 1ZCL, 1ZCM, 1ZCN, 1ZCO, 1ZCP, 1ZCR, 1ZCT, 1ZCU, 1ZCV, 1ZCW, 1ZCY, 1ZCZ, 1ZD0, 1ZD1, 1ZD2, 1ZD3, 1ZD4, 1ZD5, 1ZD6, 1ZD7, 1ZD8, 1ZD9, 1ZDE, 1ZDF, 1ZDG, 1ZDL, 1ZDM, 1ZDN, 1ZDP, 1ZDQ, 1ZDR, 1ZDS, 1ZDT, 1ZDU, 1ZDW, 1ZDY, 1ZE1, 1ZE3, 1ZE8, 1ZEA, 1ZEB, 1ZED, 1ZEE, 1ZEF, 1ZEG, 1ZEH, 1ZEI, 1ZEJ, 1ZEL, 1ZEM, 1ZEN, 1ZEO, 1ZEQ, 1ZES, 1ZET, 1ZEW, 1ZEX, 1ZEY, 1ZEZ, 1ZF0, 1ZF1, 1ZF2, 1ZF3, 1ZF4, 1ZF5, 1ZF6, 1ZF7, 1ZF8, 1ZF9, 1ZFA, 1ZFB, 1ZFC, 1ZFE, 1ZFF, 1ZFG, 1ZFH, 1ZFJ, 1ZFK, 1ZFM, 1ZFN, 1ZFP, 1ZFQ, 1ZG1, 1ZG3, 1ZG4, 1ZG5, 1ZG6, 1ZG7, 1ZG8, 1ZG9, 1ZGA, 1ZGB, 1ZGC, 1ZGD, 1ZGE, 1ZGF, 1ZGH, 1ZGI, 1ZGJ, 1ZGK, 1ZGL, 1ZGN, 1ZGO, 1ZGP, 1ZGQ, 1ZGR, 1ZGS, 1ZGT, 1ZGV, 1ZGX, 1ZGY, 1ZGZ, 1ZH0, 1ZH1, 1ZH2, 1ZH4, 1ZH6, 1ZH7, 1ZH8, 1ZH9, 1ZHA, 1ZHB, 1ZHF, 1ZHG, 1ZHH, 1ZHI, 1ZHJ, 1ZHK, 1ZHL, 1ZHM, 1ZHN, 1ZHP, 1ZHQ, 1ZHR, 1ZHS, 1ZHT, 1ZHV, 1ZHW, 1ZHX, 1ZHY, 1ZHZ, 1ZI0, 1ZI1, 1ZI3, 1ZI4, 1ZI5, 1ZI6, 1ZI7, 1ZI8, 1ZI9, 1ZIA, 1ZIB, 1ZIC, 1ZID, 1ZIE, 1ZII, 1ZIJ, 1ZIK, 1ZIL, 1ZIM, 1ZIN, 1ZIO, 1ZIP, 1ZIQ, 1ZIR, 1ZIS, 1ZIU, 1ZIV, 1ZIW, 1ZIX, 1ZIY, 1ZIZ, 1ZJ0, 1ZJ1, 1ZJ2, 1ZJ3, 1ZJ4, 1ZJ5, 1ZJ6, 1ZJ7, 1ZJ8, 1ZJ9, 1ZJA, 1ZJB, 1ZJC, 1ZJD, 1ZJE, 1ZJF, 1ZJG, 1ZJH, 1ZJI, 1ZJJ, 1ZJK, 1ZJL, 1ZJM, 1ZJN, 1ZJO, 1ZJP, 1ZJR, 1ZJY, 1ZJZ, 1ZK0, 1ZK1, 1ZK2, 1ZK3, 1ZK4, 1ZK5, 1ZK7, 1ZK8, 1ZK9, 1ZKA, 1ZKB, 1ZKC, 1ZKD, 1ZKE, 1ZKF, 1ZKG, 1ZKI, 1ZKJ, 1ZKK, 1ZKL, 1ZKM, 1ZKN, 1ZKO, 1ZKP, 1ZKQ, 1ZKR, 1ZKW, 1ZKX, 1ZKY, 1ZKZ, 1ZL0, 1ZL1, 1ZL2, 1ZL5, 1ZL6, 1ZL7, 1ZL9, 1ZLA, 1ZLB, 1ZLD, 1ZLE, 1ZLF, 1ZLH, 1ZLI, 1ZLJ, 1ZLK, 1ZLM, 1ZLP, 1ZLQ, 1ZLR, 1ZLS, 1ZLT, 1ZLU, 1ZLV, 1ZLW, 1ZLX, 1ZLY, 1ZLZ, 1ZM0, 1ZM1, 1ZM2, 1ZM3, 1ZM4, 1ZM5, 1ZM6, 1ZM7, 1ZM8, 1ZM9, 1ZMA, 1ZMB, 1ZMC, 1ZMD, 1ZME, 1ZMF, 1ZMG, 1ZMH, 1ZMI, 1ZMJ, 1ZMK, 1ZML, 1ZMM, 1ZMN, 1ZMO, 1ZMP, 1ZMQ, 1ZMR, 1ZMS, 1ZMT, 1ZMU, 1ZMV, 1ZMW, 1ZMX, 1ZMY, 1ZN2, 1ZN3, 1ZN6, 1ZN7, 1ZN8, 1ZN9, 1ZNA, 1ZNB, 1ZNC, 1ZND, 1ZNE, 1ZNG, 1ZNH, 1ZNI, 1ZNJ, 1ZNK, 1ZNL, 1ZNN, 1ZNO, 1ZNP, 1ZNQ, 1ZNS, 1ZNV, 1ZNW, 1ZNX, 1ZNY, 1ZNZ, 1ZO2, 1ZO4, 1ZO8, 1ZO9, 1ZOA, 1ZOB, 1ZOD, 1ZOE, 1ZOF, 1ZOG, 1ZOH, 1ZOI, 1ZOL, 1ZOM, 1ZON, 1ZOO, 1ZOP, 1ZOQ, 1ZOR, 1ZOS, 1ZOT, 1ZOV, 1ZOW, 1ZOX, 1ZOY, 1ZP0, 1ZP2, 1ZP3, 1ZP4, 1ZP5, 1ZP6, 1ZP7, 1ZP8, 1ZP9, 1ZPA, 1ZPB, 1ZPC, 1ZPD, 1ZPE, 1ZPG, 1ZPH, 1ZPI, 1ZPK, 1ZPL, 1ZPQ, 1ZPR, 1ZPS, 1ZPT, 1ZPU, 1ZPV, 1ZPW, 1ZPZ, 1ZQ1, 1ZQ5, 1ZQ7, 1ZQ9, 1ZQA, 1ZQB, 1ZQC, 1ZQD, 1ZQE, 1ZQF, 1ZQG, 1ZQH, 1ZQI, 1ZQJ, 1ZQK, 1ZQL, 1ZQM, 1ZQN, 1ZQO, 1ZQP, 1ZQQ, 1ZQR, 1ZQS, 1ZQT, 1ZQU, 1ZQV, 1ZQW, 1ZQX, 1ZQY, 1ZQZ, 1ZR0, 1ZR2, 1ZR3, 1ZR4, 1ZR5, 1ZR6, 1ZR8, 1ZRB, 1ZRC, 1ZRD, 1ZRE, 1ZRF, 1ZRH, 1ZRK, 1ZRL, 1ZRM, 1ZRN, 1ZRO, 1ZRQ, 1ZRS, 1ZRT, 1ZRU, 1ZRZ, 1ZS0, 1ZS2, 1ZS3, 1ZS4, 1ZS6, 1ZS7, 1ZS8, 1ZS9, 1ZSA, 1ZSB, 1ZSC, 1ZSD, 1ZSF, 1ZSH, 1ZSJ, 1ZSK, 1ZSL, 1ZSN, 1ZSO, 1ZSP, 1ZSQ, 1ZSR, 1ZSV, 1ZSW, 1ZSX, 1ZSY, 1ZSZ, 1ZT1, 1ZT2, 1ZT3, 1ZT4, 1ZT5, 1ZT7, 1ZT9, 1ZTB, 1ZTC, 1ZTD, 1ZTE, 1ZTF, 1ZTG, 1ZTH, 1ZTJ, 1ZTK, 1ZTL, 1ZTM, 1ZTP, 1ZTQ, 1ZTT, 1ZTU, 1ZTV, 1ZTW, 1ZTX, 1ZTY, 1ZTZ, 1ZU0, 1ZU3, 1ZU4, 1ZU5, 1ZU8, 1ZUA, 1ZUC, 1ZUD, 1ZUH, 1ZUI, 1ZUJ, 1ZUK, 1ZUM, 1ZUN, 1ZUO, 1ZUP, 1ZUQ, 1ZUR, 1ZUT, 1ZUU, 1ZUW, 1ZUX, 1ZUY, 1ZUZ, 1ZV1, 1ZV2, 1ZV4, 1ZV5, 1ZV7, 1ZV8, 1ZV9, 1ZVA, 1ZVB, 1ZVC, 1ZVD, 1ZVE, 1ZVF, 1ZVG, 1ZVH, 1ZVI, 1ZVJ, 1ZVK, 1ZVL, 1ZVM, 1ZVN, 1ZVP, 1ZVQ, 1ZVR, 1ZVS, 1ZVT, 1ZVU, 1ZVV, 1ZVW, 1ZVX, 1ZVY, 1ZVZ, 1ZW0, 1ZW1, 1ZW2, 1ZW3, 1ZW5, 1ZW6, 1ZW9, 1ZWH, 1ZWI, 1ZWJ, 1ZWK, 1ZWL, 1ZWN, 1ZWP, 1ZWS, 1ZWW, 1ZWX, 1ZWY, 1ZWZ, 1ZX1, 1ZX2, 1ZX3, 1ZX4, 1ZX5, 1ZX6, 1ZX8, 1ZX9, 1ZXB, 1ZXC, 1ZXE, 1ZXI, 1ZXJ, 1ZXK, 1ZXL, 1ZXM, 1ZXN, 1ZXO, 1ZXQ, 1ZXT, 1ZXU, 1ZXV, 1ZXX, 1ZXY, 1ZXZ, 1ZY0, 1ZY1, 1ZY2, 1ZY4, 1ZY5, 1ZY7, 1ZY8, 1ZY9, 1ZYB, 1ZYC, 1ZYD, 1ZYE, 1ZYJ, 1ZYK, 1ZYL, 1ZYM, 1ZYN, 1ZYO, 1ZYP, 1ZYQ, 1ZYR, 1ZYS, 1ZYT, 1ZYU, 1ZYV, 1ZYW, 1ZYX, 1ZYZ, 1ZZ0, 1ZZ1, 1ZZ2, 1ZZ3, 1ZZ6, 1ZZ7, 1ZZ8, 1ZZ9, 1ZZB, 1ZZC, 1ZZD, 1ZZE, 1ZZG, 1ZZH, 1ZZI, 1ZZJ, 1ZZK, 1ZZL, 1ZZM, 1ZZO, 1ZZQ, 1ZZR, 1ZZS, 1ZZT, 1ZZU, 1ZZW, 1ZZY, 1ZZZ, 200D, 200L, 201L, 205L, 206D, 206L, 207L, 208D, 208L, 209D, 209L, 20GS, 210L, 211L, 212D, 212L, 213D, 213L, 214L, 215L, 216D, 216L, 217D, 217L, 218D, 218L, 219L, 21BI, 220D, 220L, 221D, 221L, 221P, 222L, 223D, 223L, 224L, 225L, 226L, 227D, 227L, 228L, 229L, 22GS, 230L, 231L, 232D, 232L, 233D, 233L, 234L, 235L, 236L, 237D, 237L, 238D, 238L, 239D, 239L, 240D, 240L, 241D, 241L, 242D, 242L, 243D, 243L, 244D, 244L, 245L, 246L, 247L, 248L, 249D, 249L, 250D, 250L, 251D, 251L, 252D, 252L, 253D, 253L, 254L, 255L, 256B, 256D, 256L, 257D, 257L, 258L, 259L, 25C8, 260D, 260L, 261D, 261L, 262L, 263D, 264D, 265D, 266D, 267D, 268D, 269D, 270D, 271D, 272D, 274D, 275D, 279D, 281D, 282D, 284D, 285D, 286D, 287D, 289D, 28DN, 290D, 291D, 292D, 293D, 294D, 295D, 296D, 297D, 298D, 2A01, 2A03, 2A06, 2A07, 2A08, 2A0B, 2A0C, 2A0F, 2A0I, 2A0J, 2A0K, 2A0L, 2A0M, 2A0N, 2A0Q, 2A0S, 2A0U, 2A0W, 2A0X, 2A0Y, 2A0Z, 2A10, 2A11, 2A13, 2A14, 2A15, 2A18, 2A19, 2A1A, 2A1B, 2A1D, 2A1E, 2A1F, 2A1H, 2A1I, 2A1J, 2A1K, 2A1L, 2A1M, 2A1N, 2A1O, 2A1S, 2A1T, 2A1U, 2A1V, 2A1W, 2A1X, 2A1Y, 2A21, 2A22, 2A25, 2A26, 2A27, 2A28, 2A2A, 2A2C, 2A2D, 2A2F, 2A2G, 2A2I, 2A2J, 2A2K, 2A2L, 2A2M, 2A2N, 2A2O, 2A2Q, 2A2R, 2A2S, 2A2T, 2A2U, 2A2X, 2A2Z, 2A30, 2A31, 2A32, 2A33, 2A35, 2A38, 2A39, 2A3A, 2A3B, 2A3C, 2A3E, 2A3F, 2A3G, 2A3H, 2A3I, 2A3K, 2A3L, 2A3M, 2A3N, 2A3P, 2A3Q, 2A3R, 2A3T, 2A3U, 2A3V, 2A3W, 2A3X, 2A3Y, 2A3Z, 2A40, 2A41, 2A42, 2A45, 2A46, 2A47, 2A48, 2A49, 2A4A, 2A4C, 2A4D, 2A4E, 2A4F, 2A4G, 2A4K, 2A4L, 2A4M, 2A4N, 2A4O, 2A4Q, 2A4R, 2A4T, 2A4V, 2A4W, 2A4X, 2A4Z, 2A50, 2A52, 2A53, 2A54, 2A56, 2A57, 2A58, 2A59, 2A5A, 2A5B, 2A5C, 2A5D, 2A5F, 2A5G, 2A5H, 2A5I, 2A5J, 2A5K, 2A5L, 2A5S, 2A5T, 2A5U, 2A5V, 2A5W, 2A5X, 2A5Y, 2A5Z, 2A61, 2A62, 2A65, 2A66, 2A67, 2A68, 2A69, 2A6A, 2A6B, 2A6C, 2A6D, 2A6E, 2A6H, 2A6I, 2A6J, 2A6K, 2A6L, 2A6M, 2A6N, 2A6O, 2A6P, 2A6Q, 2A6R, 2A6S, 2A6T, 2A6V, 2A6W, 2A6X, 2A6Y, 2A6Z, 2A70, 2A71, 2A72, 2A73, 2A74, 2A75, 2A77, 2A78, 2A79, 2A7A, 2A7B, 2A7C, 2A7D, 2A7E, 2A7F, 2A7G, 2A7H, 2A7I, 2A7J, 2A7K, 2A7L, 2A7M, 2A7N, 2A7P, 2A7Q, 2A7R, 2A7S, 2A7T, 2A7W, 2A7X, 2A81, 2A83, 2A84, 2A85, 2A86, 2A87, 2A88, 2A89, 2A8A, 2A8B, 2A8C, 2A8D, 2A8E, 2A8F, 2A8G, 2A8H, 2A8I, 2A8J, 2A8K, 2A8L, 2A8M, 2A8N, 2A8P, 2A8Q, 2A8R, 2A8S, 2A8T, 2A8U, 2A8W, 2A8X, 2A8Y, 2A8Z, 2A90, 2A91, 2A92, 2A94, 2A96, 2A97, 2A98, 2A99, 2A9A, 2A9B, 2A9C, 2A9D, 2A9E, 2A9F, 2A9G, 2A9I, 2A9J, 2A9K, 2A9M, 2A9N, 2A9O, 2A9P, 2A9Q, 2A9R, 2A9S, 2A9U, 2A9V, 2A9W, 2A9Y, 2A9Z, 2AA0, 2AA1, 2AA2, 2AA3, 2AA4, 2AA5, 2AA6, 2AA7, 2AA9, 2AAA, 2AAB, 2AAC, 2AAD, 2AAE, 2AAF, 2AAG, 2AAI, 2AAJ, 2AAK, 2AAL, 2AAM, 2AAN, 2AAO, 2AAQ, 2AAT, 2AAW, 2AAX, 2AAY, 2AAZ, 2AB0, 2AB1, 2AB2, 2AB5, 2AB6, 2AB8, 2ABA, 2ABB, 2ABE, 2ABH, 2ABI, 2ABJ, 2ABK, 2ABL, 2ABM, 2ABQ, 2ABR, 2ABS, 2ABW, 2ABX, 2ABZ, 2AC0, 2AC1, 2AC2, 2AC3, 2AC4, 2AC5, 2AC7, 2ACA, 2ACE, 2ACF, 2ACG, 2ACH, 2ACI, 2ACJ, 2ACK, 2ACL, 2ACO, 2ACP, 2ACQ, 2ACR, 2ACS, 2ACT, 2ACU, 2ACV, 2ACW, 2ACX, 2ACY, 2ACZ, 2AD1, 2AD5, 2AD6, 2AD7, 2AD8, 2ADA, 2ADD, 2ADE, 2ADF, 2ADG, 2ADI, 2ADJ, 2ADM, 2ADO, 2ADP, 2ADQ, 2ADU, 2ADV, 2ADY, 2AE0, 2AE1, 2AE2, 2AE3, 2AE4, 2AE5, 2AE6, 2AE7, 2AE8, 2AEB, 2AEC, 2AEE, 2AEF, 2AEG, 2AEH, 2AEI, 2AEJ, 2AEK, 2AEL, 2AEM, 2AEN, 2AEO, 2AEP, 2AEQ, 2AER, 2AES, 2AET, 2AEU, 2AEV, 2AEW, 2AEX, 2AEY, 2AEZ, 2AF0, 2AF1, 2AF3, 2AF4, 2AF5, 2AF6, 2AF7, 2AF9, 2AFA, 2AFB, 2AFC, 2AFG, 2AFH, 2AFI, 2AFK, 2AFM, 2AFN, 2AFO, 2AFQ, 2AFR, 2AFS, 2AFT, 2AFU, 2AFV, 2AFW, 2AFX, 2AFY, 2AFZ, 2AG0, 2AG1, 2AG2, 2AG3, 2AG4, 2AG5, 2AG6, 2AG8, 2AG9, 2AGC, 2AGD, 2AGE, 2AGG, 2AGI, 2AGJ, 2AGK, 2AGL, 2AGO, 2AGP, 2AGQ, 2AGS, 2AGT, 2AGV, 2AGW, 2AGX, 2AGY, 2AGZ, 2AH0, 2AH1, 2AH2, 2AH4, 2AH5, 2AH6, 2AH7, 2AH8, 2AH9, 2AHA, 2AHB, 2AHC, 2AHD, 2AHE, 2AHF, 2AHG, 2AHI, 2AHJ, 2AHK, 2AHL, 2AHM, 2AHN, 2AHO, 2AHP, 2AHR, 2AHS, 2AHU, 2AHV, 2AHW, 2AHX, 2AHY, 2AHZ, 2AI0, 2AI1, 2AI2, 2AI3, 2AI7, 2AI8, 2AI9, 2AIA, 2AIB, 2AID, 2AIE, 2AIF, 2AIG, 2AII, 2AIJ, 2AIK, 2AIM, 2AIO, 2AIP, 2AIQ, 2AIR, 2AIU, 2AIX, 2AJ2, 2AJ3, 2AJ4, 2AJ6, 2AJ7, 2AJ8, 2AJ9, 2AJA, 2AJB, 2AJC, 2AJD, 2AJF, 2AJG, 2AJH, 2AJI, 2AJL, 2AJP, 2AJQ, 2AJR, 2AJS, 2AJT, 2AJU, 2AJV, 2AJX, 2AJY, 2AJZ, 2AK1, 2AK2, 2AK3, 2AK4, 2AK5, 2AK7, 2AKA, 2AKC, 2AKF, 2AKJ, 2AKM, 2AKO, 2AKP, 2AKQ, 2AKR, 2AKW, 2AKY, 2AKZ, 2AL0, 2AL1, 2AL2, 2AL4, 2AL5, 2AL6, 2AL7, 2ALA, 2ALD, 2ALE, 2ALF, 2ALG, 2ALL, 2ALM, 2ALP, 2ALR, 2ALU, 2ALV, 2ALW, 2ALX, 2ALY, 2ALZ, 2AM1, 2AM2, 2AM3, 2AM4, 2AM5, 2AM9, 2AMA, 2AMB, 2AMC, 2AMD, 2AME, 2AMF, 2AMG, 2AMH, 2AMJ, 2AML, 2AMM, 2AMO, 2AMP, 2AMQ, 2AMS, 2AMT, 2AMU, 2AMV, 2AMX, 2AMY, 2AN0, 2AN1, 2AN2, 2AN3, 2AN4, 2AN5, 2AN6, 2AN9, 2ANA, 2ANB, 2ANC, 2ANE, 2ANG, 2ANH, 2ANI, 2ANJ, 2ANK, 2ANL, 2ANM, 2ANO, 2ANP, 2ANQ, 2ANS, 2ANT, 2ANU, 2ANV, 2ANW, 2ANX, 2ANY, 2ANZ, 2AO2, 2AO6, 2AO7, 2AO9, 2AOA, 2AOC, 2AOD, 2AOE, 2AOF, 2AOG, 2AOH, 2AOI, 2AOJ, 2AOP, 2AOQ, 2AOR, 2AOS, 2AOT, 2AOU, 2AOV, 2AOW, 2AOX, 2AOZ, 2AP1, 2AP2, 2AP3, 2AP6, 2AP9, 2APB, 2APC, 2APF, 2APG, 2APH, 2APJ, 2APL, 2APO, 2APQ, 2APR, 2APS, 2APT, 2APV, 2APW, 2APX, 2AQ1, 2AQ2, 2AQ3, 2AQ4, 2AQ5, 2AQ6, 2AQ7, 2AQ8, 2AQ9, 2AQB, 2AQD, 2AQH, 2AQI, 2AQJ, 2AQK, 2AQL, 2AQN, 2AQO, 2AQP, 2AQQ, 2AQR, 2AQS, 2AQT, 2AQU, 2AQV, 2AQW, 2AQX, 2AQZ, 2AR0, 2AR1, 2AR3, 2AR5, 2AR6, 2AR7, 2AR8, 2AR9, 2ARA, 2ARB, 2ARC, 2ARD, 2ARE, 2ARH, 2ARJ, 2ARK, 2ARL, 2ARM, 2ARO, 2ARP, 2ARQ, 2ARR, 2ARS, 2ART, 2ARU, 2ARV, 2ARX, 2ARY, 2ARZ, 2AS0, 2AS1, 2AS2, 2AS3, 2AS4, 2AS5, 2AS6, 2AS8, 2AS9, 2ASC, 2ASD, 2ASF, 2ASH, 2ASI, 2ASJ, 2ASK, 2ASL, 2ASM, 2ASN, 2ASO, 2ASP, 2ASR, 2ASS, 2AST, 2ASU, 2ASV, 2AT0, 2AT1, 2AT2, 2AT3, 2AT5, 2AT6, 2AT8, 2ATA, 2ATB, 2ATE, 2ATF, 2ATH, 2ATI, 2ATJ, 2ATK, 2ATL, 2ATM, 2ATO, 2ATP, 2ATQ, 2ATR, 2ATS, 2ATV, 2ATX, 2ATZ, 2AU0, 2AU1, 2AU3, 2AU5, 2AU6, 2AU7, 2AU8, 2AU9, 2AUA, 2AUB, 2AUC, 2AUD, 2AUG, 2AUH, 2AUJ, 2AUK, 2AUM, 2AUN, 2AUO, 2AUP, 2AUQ, 2AUR, 2AUS, 2AUT, 2AUU, 2AUW, 2AUX, 2AUY, 2AUZ, 2AV0, 2AV1, 2AV3, 2AV4, 2AV5, 2AV6, 2AV7, 2AV8, 2AV9, 2AVD, 2AVF, 2AVH, 2AVI, 2AVJ, 2AVK, 2AVM, 2AVN, 2AVO, 2AVP, 2AVQ, 2AVS, 2AVT, 2AVU, 2AVV, 2AVW, 2AW1, 2AW2, 2AW3, 2AW5, 2AW6, 2AW9, 2AWA, 2AWC, 2AWD, 2AWF, 2AWG, 2AWH, 2AWI, 2AWJ, 2AWK, 2AWL, 2AWM, 2AWN, 2AWO, 2AWP, 2AWU, 2AWW, 2AWX, 2AWY, 2AWZ, 2AX0, 2AX1, 2AX2, 2AX3, 2AX4, 2AX6, 2AX7, 2AX8, 2AX9, 2AXA, 2AXB, 2AXC, 2AXE, 2AXF, 2AXG, 2AXH, 2AXI, 2AXJ, 2AXM, 2AXN, 2AXO, 2AXP, 2AXQ, 2AXR, 2AXT, 2AXU, 2AXV, 2AXW, 2AXY, 2AXZ, 2AY0, 2AY1, 2AY2, 2AY3, 2AY4, 2AY5, 2AY6, 2AY7, 2AY8, 2AY9, 2AYB, 2AYD, 2AYE, 2AYG, 2AYH, 2AYI, 2AYL, 2AYN, 2AYO, 2AYP, 2AYQ, 2AYR, 2AYS, 2AYT, 2AYU, 2AYV, 2AYW, 2AZ1, 2AZ3, 2AZ4, 2AZ5, 2AZ8, 2AZ9, 2AZA, 2AZB, 2AZC, 2AZD, 2AZE, 2AZJ, 2AZK, 2AZL, 2AZM, 2AZN, 2AZO, 2AZP, 2AZQ, 2AZR, 2AZT, 2AZU, 2AZW, 2AZY, 2AZZ, 2B00, 2B01, 2B02, 2B03, 2B04, 2B05, 2B06, 2B07, 2B08, 2B0A, 2B0C, 2B0D, 2B0E, 2B0J, 2B0K, 2B0L, 2B0M, 2B0O, 2B0P, 2B0Q, 2B0R, 2B0S, 2B0T, 2B0U, 2B0V, 2B0Z, 2B10, 2B11, 2B12, 2B13, 2B14, 2B15, 2B16, 2B17, 2B18, 2B1A, 2B1B, 2B1C, 2B1D, 2B1E, 2B1F, 2B1G, 2B1H, 2B1I, 2B1J, 2B1K, 2B1L, 2B1M, 2B1N, 2B1P, 2B1Q, 2B1R, 2B1V, 2B1X, 2B1Y, 2B1Z, 2B20, 2B21, 2B22, 2B23, 2B24, 2B25, 2B26, 2B29, 2B2A, 2B2B, 2B2C, 2B2F, 2B2H, 2B2I, 2B2J, 2B2K, 2B2N, 2B2O, 2B2Q, 2B2R, 2B2S, 2B2T, 2B2U, 2B2V, 2B2W, 2B2X, 2B2Y, 2B30, 2B31, 2B33, 2B34, 2B35, 2B36, 2B37, 2B39, 2B3B, 2B3D, 2B3E, 2B3F, 2B3G, 2B3H, 2B3K, 2B3L, 2B3M, 2B3O, 2B3P, 2B3Q, 2B3R, 2B3S, 2B3T, 2B3U, 2B3V, 2B3X, 2B3Y, 2B3Z, 2B42, 2B43, 2B44, 2B45, 2B46, 2B48, 2B49, 2B4A, 2B4B, 2B4C, 2B4D, 2B4E, 2B4F, 2B4G, 2B4H, 2B4I, 2B4J, 2B4K, 2B4L, 2B4M, 2B4O, 2B4P, 2B4Q, 2B4R, 2B4S, 2B4T, 2B4U, 2B4V, 2B4W, 2B4X, 2B4Y, 2B4Z, 2B50, 2B51, 2B52, 2B53, 2B54, 2B55, 2B56, 2B58, 2B59, 2B5A, 2B5D, 2B5E, 2B5F, 2B5G, 2B5H, 2B5I, 2B5J, 2B5L, 2B5M, 2B5N, 2B5O, 2B5R, 2B5S, 2B5T, 2B5U, 2B5V, 2B5W, 2B5Z, 2B60, 2B61, 2B65, 2B67, 2B69, 2B6A, 2B6C, 2B6E, 2B6H, 2B6M, 2B6N, 2B6P, 2B6T, 2B6W, 2B6X, 2B6Y, 2B6Z, 2B70, 2B71, 2B72, 2B73, 2B74, 2B75, 2B76, 2B77, 2B78, 2B7A, 2B7B, 2B7C, 2B7D, 2B7F, 2B7H, 2B7J, 2B7K, 2B7L, 2B7M, 2B7N, 2B7O, 2B7P, 2B7Q, 2B7R, 2B7S, 2B7U, 2B7X, 2B7Y, 2B7Z, 2B81, 2B82, 2B83, 2B8E, 2B8H, 2B8I, 2B8J, 2B8K, 2B8L, 2B8M, 2B8N, 2B8O, 2B8P, 2B8Q, 2B8T, 2B8U, 2B8V, 2B8W, 2B8X, 2B8Y, 2B8Z, 2B90, 2B91, 2B92, 2B94, 2B96, 2B97, 2B98, 2B99, 2B9A, 2B9B, 2B9C, 2B9D, 2B9E, 2B9F, 2B9H, 2B9I, 2B9J, 2B9L, 2B9R, 2B9S, 2B9U, 2B9V, 2B9W, 2B9X, 2B9Y, 2BA0, 2BA1, 2BA2, 2BA9, 2BAA, 2BAB, 2BAC, 2BAG, 2BAJ, 2BAK, 2BAL, 2BAM, 2BAN, 2BAP, 2BAQ, 2BAS, 2BAT, 2BAW, 2BAX, 2BAY, 2BAZ, 2BB0, 2BB2, 2BB3, 2BB4, 2BB5, 2BB6, 2BB7, 2BB9, 2BBA, 2BBB, 2BBC, 2BBD, 2BBE, 2BBF, 2BBH, 2BBJ, 2BBK, 2BBO, 2BBQ, 2BBR, 2BBS, 2BBT, 2BBW, 2BBZ, 2BC0, 2BC1, 2BC2, 2BC3, 2BC4, 2BC5, 2BC9, 2BCC, 2BCD, 2BCE, 2BCG, 2BCH, 2BCJ, 2BCK, 2BCM, 2BCN, 2BCO, 2BCP, 2BCQ, 2BCR, 2BCS, 2BCT, 2BCU, 2BCV, 2BCX, 2BD0, 2BD1, 2BD2, 2BD3, 2BD4, 2BD5, 2BD7, 2BD8, 2BD9, 2BDA, 2BDB, 2BDC, 2BDD, 2BDE, 2BDF, 2BDG, 2BDH, 2BDI, 2BDJ, 2BDL, 2BDM, 2BDN, 2BDP, 2BDQ, 2BDR, 2BDT, 2BDU, 2BDV, 2BDW, 2BDX, 2BDY, 2BDZ, 2BE1, 2BE2, 2BE3, 2BE4, 2BE5, 2BE6, 2BE7, 2BE9, 2BEA, 2BEB, 2BEC, 2BED, 2BEF, 2BEH, 2BEI, 2BEJ, 2BEK, 2BEL, 2BEM, 2BEN, 2BEO, 2BEP, 2BEQ, 2BER, 2BES, 2BET, 2BEU, 2BEV, 2BEW, 2BEX, 2BEZ, 2BF0, 2BF1, 2BF2, 2BF3, 2BF4, 2BF5, 2BF6, 2BF7, 2BF8, 2BF9, 2BFA, 2BFB, 2BFC, 2BFD, 2BFE, 2BFF, 2BFG, 2BFH, 2BFI, 2BFK, 2BFL, 2BFM, 2BFN, 2BFO, 2BFP, 2BFQ, 2BFR, 2BFV, 2BFW, 2BFX, 2BFY, 2BFZ, 2BG1, 2BG2, 2BG5, 2BG6, 2BG7, 2BG8, 2BGA, 2BGC, 2BGD, 2BGE, 2BGH, 2BGI, 2BGJ, 2BGK, 2BGL, 2BGM, 2BGN, 2BGQ, 2BGR, 2BGS, 2BGT, 2BGU, 2BGV, 2BGW, 2BH0, 2BH1, 2BH3, 2BH4, 2BH5, 2BH7, 2BH8, 2BH9, 2BHA, 2BHB, 2BHC, 2BHD, 2BHE, 2BHF, 2BHG, 2BHH, 2BHJ, 2BHK, 2BHL, 2BHM, 2BHN, 2BHO, 2BHP, 2BHQ, 2BHR, 2BHS, 2BHT, 2BHU, 2BHV, 2BHX, 2BHY, 2BHZ, 2BI0, 2BI1, 2BI2, 2BI3, 2BI4, 2BI5, 2BI7, 2BI8, 2BI9, 2BIA, 2BIB, 2BIE, 2BIF, 2BIG, 2BIH, 2BII, 2BIJ, 2BIK, 2BIL, 2BIM, 2BIN, 2BIO, 2BIP, 2BIQ, 2BIR, 2BIS, 2BIT, 2BIU, 2BIV, 2BIW, 2BIX, 2BIY, 2BJ0, 2BJ1, 2BJ3, 2BJ4, 2BJ7, 2BJ8, 2BJ9, 2BJA, 2BJB, 2BJD, 2BJE, 2BJF, 2BJG, 2BJH, 2BJI, 2BJJ, 2BJK, 2BJM, 2BJN, 2BJO, 2BJQ, 2BJR, 2BJS, 2BJU, 2BJV, 2BJW, 2BJY, 2BK0, 2BK3, 2BK4, 2BK5, 2BK6, 2BK8, 2BK9, 2BKA, 2BKB, 2BKC, 2BKE, 2BKF, 2BKG, 2BKH, 2BKI, 2BKJ, 2BKK, 2BKL, 2BKM, 2BKN, 2BKO, 2BKP, 2BKQ, 2BKR, 2BKS, 2BKT, 2BKU, 2BKV, 2BKW, 2BKX, 2BKY, 2BKZ, 2BL0, 2BL1, 2BL2, 2BL4, 2BL7, 2BL8, 2BL9, 2BLA, 2BLB, 2BLC, 2BLE, 2BLF, 2BLG, 2BLH, 2BLI, 2BLJ, 2BLL, 2BLM, 2BLN, 2BLO, 2BLP, 2BLQ, 2BLR, 2BLS, 2BLU, 2BLV, 2BLW, 2BLX, 2BLY, 2BLZ, 2BM0, 2BM1, 2BM2, 2BM3, 2BM4, 2BM5, 2BM6, 2BM7, 2BM8, 2BM9, 2BMA, 2BMB, 2BMC, 2BMD, 2BME, 2BMF, 2BMG, 2BMH, 2BMI, 2BMJ, 2BMK, 2BML, 2BMM, 2BMO, 2BMQ, 2BMR, 2BMU, 2BMV, 2BMW, 2BMX, 2BMY, 2BMZ, 2BN0, 2BN1, 2BN2, 2BN3, 2BN4, 2BN7, 2BNA, 2BND, 2BNE, 2BNF, 2BNG, 2BNH, 2BNI, 2BNJ, 2BNK, 2BNL, 2BNM, 2BNN, 2BNO, 2BNP, 2BNQ, 2BNR, 2BNS, 2BNU, 2BNW, 2BNX, 2BNZ, 2BO0, 2BO1, 2BO2, 2BO3, 2BO4, 2BO6, 2BO7, 2BO8, 2BO9, 2BOA, 2BOB, 2BOC, 2BOD, 2BOE, 2BOF, 2BOG, 2BOH, 2BOI, 2BOJ, 2BOK, 2BOL, 2BON, 2BOO, 2BOP, 2BOQ, 2BOS, 2BOU, 2BOV, 2BOW, 2BOX, 2BOY, 2BOZ, 2BP0, 2BP1, 2BP2, 2BP3, 2BP5, 2BP6, 2BP7, 2BP8, 2BPB, 2BPC, 2BPD, 2BPE, 2BPF, 2BPG, 2BPH, 2BPI, 2BPM, 2BPO, 2BPP, 2BPQ, 2BPS, 2BPT, 2BPU, 2BPV, 2BPW, 2BPX, 2BPY, 2BPZ, 2BQ0, 2BQ1, 2BQ3, 2BQ4, 2BQ6, 2BQ7, 2BQ8, 2BQA, 2BQB, 2BQC, 2BQD, 2BQE, 2BQF, 2BQG, 2BQH, 2BQI, 2BQJ, 2BQK, 2BQL, 2BQM, 2BQN, 2BQO, 2BQP, 2BQQ, 2BQR, 2BQU, 2BQV, 2BQW, 2BQX, 2BQY, 2BQZ, 2BR0, 2BR1, 2BR2, 2BR3, 2BR4, 2BR5, 2BR6, 2BR7, 2BR8, 2BR9, 2BRA, 2BRB, 2BRC, 2BRE, 2BRF, 2BRG, 2BRH, 2BRI, 2BRJ, 2BRK, 2BRL, 2BRM, 2BRN, 2BRO, 2BRP, 2BRQ, 2BRR, 2BRS, 2BRT, 2BRV, 2BRW, 2BRX, 2BRY, 2BS2, 2BS3, 2BS4, 2BS5, 2BS6, 2BS7, 2BS8, 2BS9, 2BSA, 2BSB, 2BSC, 2BSD, 2BSE, 2BSF, 2BSH, 2BSI, 2BSJ, 2BSK, 2BSL, 2BSM, 2BSP, 2BSQ, 2BSR, 2BSS, 2BST, 2BSW, 2BSX, 2BSY, 2BSZ, 2BT0, 2BT1, 2BT2, 2BT3, 2BT4, 2BT6, 2BT7, 2BT8, 2BT9, 2BTC, 2BTD, 2BTF, 2BTI, 2BTJ, 2BTL, 2BTM, 2BTN, 2BTO, 2BTP, 2BTQ, 2BTR, 2BTS, 2BTU, 2BTW, 2BTY, 2BTZ, 2BU2, 2BU3, 2BU4, 2BU5, 2BU6, 2BU7, 2BU8, 2BU9, 2BUA, 2BUB, 2BUC, 2BUE, 2BUF, 2BUH, 2BUI, 2BUJ, 2BUK, 2BUM, 2BUO, 2BUP, 2BUQ, 2BUR, 2BUT, 2BUU, 2BUV, 2BUW, 2BUX, 2BUY, 2BUZ, 2BV0, 2BV1, 2BV2, 2BV3, 2BV4, 2BV5, 2BV6, 2BV7, 2BV8, 2BV9, 2BVA, 2BVC, 2BVD, 2BVE, 2BVF, 2BVG, 2BVH, 2BVJ, 2BVL, 2BVM, 2BVN, 2BVO, 2BVP, 2BVQ, 2BVR, 2BVS, 2BVT, 2BVU, 2BVV, 2BVW, 2BVX, 2BVY, 2BVZ, 2BW0, 2BW1, 2BW3, 2BW4, 2BW5, 2BW7, 2BW8, 2BW9, 2BWA, 2BWB, 2BWC, 2BWD, 2BWE, 2BWF, 2BWG, 2BWH, 2BWI, 2BWJ, 2BWK, 2BWL, 2BWM, 2BWN, 2BWO, 2BWP, 2BWQ, 2BWR, 2BWS, 2BWT, 2BWU, 2BWV, 2BWW, 2BWX, 2BWY, 2BX3, 2BX4, 2BX5, 2BX6, 2BX7, 2BX8, 2BX9, 2BXA, 2BXB, 2BXC, 2BXD, 2BXE, 2BXF, 2BXG, 2BXH, 2BXI, 2BXJ, 2BXK, 2BXL, 2BXM, 2BXN, 2BXO, 2BXP, 2BXQ, 2BXR, 2BXS, 2BXT, 2BXU, 2BXV, 2BXW, 2BXX, 2BXY, 2BXZ, 2BY0, 2BY1, 2BY2, 2BY3, 2BY4, 2BY5, 2BY6, 2BY7, 2BY8, 2BY9, 2BYA, 2BYB, 2BYC, 2BYD, 2BYG, 2BYH, 2BYI, 2BYJ, 2BYK, 2BYL, 2BYM, 2BYN, 2BYO, 2BYP, 2BYQ, 2BYR, 2BYS, 2BYV, 2BYW, 2BYX, 2BYY, 2BYZ, 2BZ0, 2BZ1, 2BZ3, 2BZ4, 2BZ5, 2BZ6, 2BZ7, 2BZ8, 2BZ9, 2BZA, 2BZC, 2BZD, 2BZF, 2BZG, 2BZH, 2BZI, 2BZJ, 2BZK, 2BZL, 2BZN, 2BZR, 2BZS, 2BZU, 2BZV, 2BZW, 2BZX, 2BZY, 2BZZ, 2C00, 2C01, 2C02, 2C03, 2C04, 2C05, 2C07, 2C08, 2C0A, 2C0C, 2C0D, 2C0E, 2C0F, 2C0G, 2C0H, 2C0I, 2C0J, 2C0K, 2C0L, 2C0M, 2C0N, 2C0O, 2C0P, 2C0Q, 2C0R, 2C0T, 2C0U, 2C0Y, 2C0Z, 2C10, 2C11, 2C12, 2C13, 2C14, 2C15, 2C16, 2C18, 2C19, 2C1A, 2C1B, 2C1C, 2C1D, 2C1E, 2C1F, 2C1G, 2C1H, 2C1I, 2C1J, 2C1L, 2C1M, 2C1N, 2C1O, 2C1P, 2C1Q, 2C1S, 2C1T, 2C1U, 2C1V, 2C1W, 2C1X, 2C1Y, 2C1Z, 2C20, 2C21, 2C22, 2C23, 2C24, 2C25, 2C26, 2C27, 2C28, 2C29, 2C2A, 2C2B, 2C2C, 2C2D, 2C2E, 2C2F, 2C2G, 2C2H, 2C2I, 2C2J, 2C2K, 2C2L, 2C2M, 2C2N, 2C2O, 2C2P, 2C2Q, 2C2R, 2C2S, 2C2T, 2C2U, 2C2V, 2C2W, 2C2X, 2C2Y, 2C2Z, 2C30, 2C31, 2C32, 2C35, 2C36, 2C37, 2C38, 2C39, 2C3A, 2C3B, 2C3C, 2C3D, 2C3E, 2C3F, 2C3G, 2C3H, 2C3I, 2C3J, 2C3K, 2C3L, 2C3M, 2C3N, 2C3O, 2C3P, 2C3Q, 2C3S, 2C3T, 2C3U, 2C3V, 2C3W, 2C3X, 2C3Y, 2C3Z, 2C40, 2C41, 2C42, 2C43, 2C44, 2C45, 2C46, 2C47, 2C49, 2C4A, 2C4B, 2C4C, 2C4D, 2C4E, 2C4F, 2C4G, 2C4H, 2C4I, 2C4J, 2C4K, 2C4L, 2C4M, 2C4N, 2C4P, 2C4T, 2C4U, 2C4V, 2C4W, 2C4X, 2C53, 2C54, 2C56, 2C57, 2C58, 2C59, 2C5A, 2C5B, 2C5C, 2C5D, 2C5E, 2C5F, 2C5G, 2C5H, 2C5I, 2C5J, 2C5K, 2C5L, 2C5N, 2C5O, 2C5Q, 2C5R, 2C5S, 2C5U, 2C5V, 2C5W, 2C5X, 2C5Y, 2C60, 2C61, 2C62, 2C63, 2C64, 2C65, 2C66, 2C67, 2C68, 2C69, 2C6C, 2C6D, 2C6E, 2C6F, 2C6G, 2C6H, 2C6I, 2C6J, 2C6K, 2C6L, 2C6M, 2C6N, 2C6O, 2C6P, 2C6Q, 2C6R, 2C6T, 2C6U, 2C6W, 2C6X, 2C6Y, 2C6Z, 2C70, 2C71, 2C72, 2C73, 2C74, 2C75, 2C76, 2C77, 2C78, 2C79, 2C7A, 2C7B, 2C7F, 2C7G, 2C7I, 2C7J, 2C7K, 2C7L, 2C7M, 2C7N, 2C7O, 2C7P, 2C7Q, 2C7R, 2C7S, 2C7T, 2C7U, 2C7V, 2C7W, 2C7X, 2C7Y, 2C7Z, 2C80, 2C81, 2C82, 2C83, 2C84, 2C86, 2C88, 2C89, 2C8A, 2C8B, 2C8C, 2C8D, 2C8E, 2C8F, 2C8G, 2C8H, 2C8J, 2C8K, 2C8L, 2C8M, 2C8N, 2C8O, 2C8P, 2C8Q, 2C8R, 2C8S, 2C8T, 2C8U, 2C8V, 2C8W, 2C8X, 2C8Y, 2C8Z, 2C90, 2C91, 2C92, 2C93, 2C94, 2C95, 2C96, 2C97, 2C98, 2C99, 2C9A, 2C9B, 2C9C, 2C9D, 2C9E, 2C9H, 2C9I, 2C9J, 2C9K, 2C9L, 2C9M, 2C9N, 2C9O, 2C9P, 2C9Q, 2C9R, 2C9S, 2C9T, 2C9U, 2C9V, 2C9W, 2C9X, 2C9Y, 2C9Z, 2CA0, 2CA1, 2CA2, 2CA3, 2CA4, 2CA5, 2CA6, 2CA8, 2CA9, 2CAB, 2CAD, 2CAG, 2CAH, 2CAI, 2CAJ, 2CAK, 2CAL, 2CAM, 2CAN, 2CAQ, 2CAR, 2CAU, 2CAV, 2CAX, 2CAY, 2CAZ, 2CB0, 2CB1, 2CB2, 2CB3, 2CB4, 2CB5, 2CB6, 2CB8, 2CB9, 2CBA, 2CBB, 2CBC, 2CBD, 2CBE, 2CBF, 2CBG, 2CBI, 2CBJ, 2CBL, 2CBM, 2CBN, 2CBO, 2CBP, 2CBQ, 2CBR, 2CBS, 2CBT, 2CBU, 2CBV, 2CBX, 2CBY, 2CBZ, 2CC0, 2CC1, 2CC2, 2CC3, 2CC6, 2CC7, 2CC8, 2CC9, 2CCA, 2CCB, 2CCC, 2CCD, 2CCE, 2CCF, 2CCG, 2CCH, 2CCI, 2CCJ, 2CCK, 2CCL, 2CCM, 2CCN, 2CCP, 2CCQ, 2CCR, 2CCS, 2CCT, 2CCU, 2CCV, 2CCW, 2CCY, 2CCZ, 2CD0, 2CD2, 2CD7, 2CD8, 2CD9, 2CDA, 2CDB, 2CDC, 2CDE, 2CDF, 2CDG, 2CDH, 2CDM, 2CDN, 2CDO, 2CDP, 2CDQ, 2CDR, 2CDS, 2CDT, 2CDU, 2CDV, 2CDY, 2CDZ, 2CE0, 2CE1, 2CE2, 2CE3, 2CE4, 2CE6, 2CE7, 2CE8, 2CE9, 2CEA, 2CEI, 2CEJ, 2CEK, 2CEL, 2CEM, 2CEN, 2CEO, 2CEP, 2CEQ, 2CER, 2CES, 2CET, 2CEU, 2CEV, 2CEX, 2CEY, 2CF2, 2CF4, 2CF5, 2CF6, 2CF7, 2CF8, 2CF9, 2CFA, 2CFB, 2CFC, 2CFD, 2CFE, 2CFF, 2CFG, 2CFH, 2CFI, 2CFK, 2CFL, 2CFM, 2CFO, 2CFP, 2CFQ, 2CFR, 2CFS, 2CFT, 2CFU, 2CFV, 2CFW, 2CFX, 2CFY, 2CFZ, 2CG0, 2CG1, 2CG2, 2CG3, 2CG4, 2CG5, 2CG6, 2CG7, 2CG8, 2CG9, 2CGA, 2CGE, 2CGF, 2CGH, 2CGI, 2CGJ, 2CGK, 2CGL, 2CGN, 2CGO, 2CGP, 2CGQ, 2CGR, 2CGU, 2CGV, 2CGW, 2CGX, 2CGY, 2CGZ, 2CH1, 2CH2, 2CH4, 2CH5, 2CH6, 2CH7, 2CH8, 2CH9, 2CHA, 2CHB, 2CHC, 2CHD, 2CHE, 2CHF, 2CHG, 2CHH, 2CHI, 2CHL, 2CHM, 2CHN, 2CHO, 2CHP, 2CHQ, 2CHR, 2CHS, 2CHT, 2CHU, 2CHV, 2CHW, 2CHX, 2CHY, 2CHZ, 2CI0, 2CI1, 2CI2, 2CI3, 2CI4, 2CI5, 2CI6, 2CI7, 2CI8, 2CI9, 2CIA, 2CIB, 2CIC, 2CIE, 2CIF, 2CIG, 2CIH, 2CII, 2CIJ, 2CIK, 2CIM, 2CIN, 2CIO, 2CIP, 2CIQ, 2CIR, 2CIS, 2CIT, 2CIU, 2CIV, 2CIW, 2CIX, 2CIY, 2CIZ, 2CJ0, 2CJ1, 2CJ2, 2CJ3, 2CJ4, 2CJ5, 2CJ6, 2CJ7, 2CJ8, 2CJ9, 2CJA, 2CJB, 2CJC, 2CJD, 2CJE, 2CJF, 2CJG, 2CJH, 2CJI, 2CJJ, 2CJL, 2CJM, 2CJP, 2CJQ, 2CJR, 2CJS, 2CJT, 2CJU, 2CJW, 2CJX, 2CJY, 2CJZ, 2CK0, 2CK1, 2CK2, 2CK3, 2CKB, 2CKD, 2CKE, 2CKF, 2CKG, 2CKH, 2CKI, 2CKJ, 2CKK, 2CKL, 2CKM, 2CKO, 2CKP, 2CKQ, 2CKR, 2CKS, 2CKW, 2CKX, 2CKZ, 2CL0, 2CL2, 2CL3, 2CL4, 2CL5, 2CL6, 2CL7, 2CL8, 2CLA, 2CLB, 2CLC, 2CLD, 2CLE, 2CLF, 2CLH, 2CLI, 2CLK, 2CLL, 2CLM, 2CLO, 2CLP, 2CLQ, 2CLR, 2CLS, 2CLT, 2CLU, 2CLV, 2CLW, 2CLX, 2CLY, 2CLZ, 2CM0, 2CM1, 2CM2, 2CM3, 2CM4, 2CM5, 2CM6, 2CM7, 2CM8, 2CM9, 2CMA, 2CMB, 2CMC, 2CMD, 2CME, 2CMF, 2CMG, 2CMH, 2CMJ, 2CMK, 2CML, 2CMM, 2CMN, 2CMO, 2CMP, 2CMR, 2CMT, 2CMU, 2CMV, 2CMW, 2CMY, 2CMZ, 2CN0, 2CN1, 2CN2, 2CN3, 2CN4, 2CN5, 2CN6, 2CN7, 2CN8, 2CNA, 2CNB, 2CNC, 2CND, 2CNE, 2CNF, 2CNG, 2CNH, 2CNI, 2CNK, 2CNL, 2CNM, 2CNN, 2CNO, 2CNQ, 2CNS, 2CNT, 2CNU, 2CNV, 2CNW, 2CNX, 2CNY, 2CNZ, 2CO0, 2CO1, 2CO2, 2CO3, 2CO4, 2CO5, 2CO6, 2CO7, 2COG, 2COI, 2COJ, 2COL, 2COQ, 2COV, 2CP4, 2CPG, 2CPK, 2CPL, 2CPO, 2CPP, 2CPU, 2CQS, 2CQT, 2CQZ, 2CRK, 2CRO, 2CRX, 2CS7, 2CSB, 2CSC, 2CSD, 2CSG, 2CSL, 2CSM, 2CSN, 2CST, 2CSU, 2CT9, 2CTB, 2CTC, 2CTH, 2CTS, 2CTV, 2CTX, 2CTZ, 2CU0, 2CU2, 2CU3, 2CU5, 2CU6, 2CU9, 2CUA, 2CUK, 2CUL, 2CUN, 2CUO, 2CUT, 2CUU, 2CUW, 2CUY, 2CUZ, 2CV3, 2CV4, 2CV5, 2CV6, 2CV8, 2CV9, 2CVB, 2CVC, 2CVD, 2CVE, 2CVF, 2CVH, 2CVI, 2CVJ, 2CVK, 2CVL, 2CVO, 2CVP, 2CVQ, 2CVS, 2CVT, 2CVU, 2CVV, 2CVW, 2CVX, 2CVY, 2CVZ, 2CW0, 2CW2, 2CW3, 2CW4, 2CW5, 2CW6, 2CW7, 2CW8, 2CW9, 2CWA, 2CWC, 2CWD, 2CWE, 2CWF, 2CWG, 2CWH, 2CWI, 2CWJ, 2CWK, 2CWL, 2CWM, 2CWN, 2CWO, 2CWP, 2CWQ, 2CWR, 2CWS, 2CWT, 2CWU, 2CWV, 2CWW, 2CWX, 2CWY, 2CWZ, 2CX0, 2CX1, 2CX3, 2CX4, 2CX5, 2CX6, 2CX7, 2CX8, 2CX9, 2CXA, 2CXB, 2CXC, 2CXD, 2CXE, 2CXF, 2CXG, 2CXH, 2CXI, 2CXK, 2CXL, 2CXN, 2CXO, 2CXP, 2CXQ, 2CXR, 2CXS, 2CXT, 2CXU, 2CXV, 2CXX, 2CXY, 2CY0, 2CY1, 2CY2, 2CY3, 2CY4, 2CY5, 2CY6, 2CY7, 2CY8, 2CY9, 2CYA, 2CYB, 2CYC, 2CYD, 2CYE, 2CYF, 2CYG, 2CYH, 2CYJ, 2CYM, 2CYP, 2CYX, 2CYY, 2CYZ, 2CZ0, 2CZ1, 2CZ2, 2CZ3, 2CZ4, 2CZ5, 2CZ6, 2CZ7, 2CZ8, 2CZ9, 2CZC, 2CZD, 2CZE, 2CZF, 2CZG, 2CZH, 2CZI, 2CZK, 2CZL, 2CZQ, 2CZR, 2CZS, 2CZT, 2CZU, 2CZV, 2CZW, 2D00, 2D01, 2D02, 2D03, 2D04, 2D05, 2D06, 2D07, 2D09, 2D0A, 2D0B, 2D0C, 2D0D, 2D0E, 2D0F, 2D0G, 2D0H, 2D0I, 2D0J, 2D0K, 2D0N, 2D0O, 2D0P, 2D0Q, 2D0S, 2D0T, 2D0U, 2D0V, 2D0W, 2D10, 2D11, 2D13, 2D16, 2D1C, 2D1E, 2D1F, 2D1G, 2D1H, 2D1I, 2D1J, 2D1K, 2D1L, 2D1N, 2D1O, 2D1P, 2D1Q, 2D1R, 2D1S, 2D1T, 2D1V, 2D1W, 2D1X, 2D1Y, 2D1Z, 2D20, 2D22, 2D23, 2D24, 2D25, 2D26, 2D27, 2D28, 2D29, 2D2A, 2D2C, 2D2D, 2D2E, 2D2F, 2D2G, 2D2H, 2D2I, 2D2J, 2D2M, 2D2N, 2D2O, 2D2Q, 2D2R, 2D2S, 2D2V, 2D2X, 2D2Z, 2D30, 2D31, 2D32, 2D33, 2D36, 2D37, 2D38, 2D39, 2D3A, 2D3B, 2D3C, 2D3D, 2D3E, 2D3F, 2D3G, 2D3H, 2D3I, 2D3K, 2D3L, 2D3M, 2D3N, 2D3P, 2D3Q, 2D3R, 2D3S, 2D3T, 2D3U, 2D3V, 2D3W, 2D3Y, 2D3Z, 2D40, 2D41, 2D42, 2D43, 2D44, 2D45, 2D47, 2D48, 2D4A, 2D4C, 2D4D, 2D4E, 2D4F, 2D4G, 2D4H, 2D4I, 2D4J, 2D4K, 2D4L, 2D4M, 2D4N, 2D4O, 2D4P, 2D4Q, 2D4R, 2D4U, 2D4V, 2D4W, 2D4X, 2D4Y, 2D4Z, 2D51, 2D52, 2D54, 2D55, 2D58, 2D59, 2D5A, 2D5B, 2D5C, 2D5D, 2D5F, 2D5G, 2D5H, 2D5I, 2D5J, 2D5K, 2D5L, 2D5M, 2D5N, 2D5R, 2D5V, 2D5W, 2D5X, 2D5Y, 2D5Z, 2D60, 2D61, 2D62, 2D63, 2D64, 2D65, 2D66, 2D68, 2D69, 2D6B, 2D6C, 2D6K, 2D6L, 2D6M, 2D6N, 2D6O, 2D6P, 2D6Y, 2D73, 2D74, 2D7C, 2D7D, 2D7E, 2D7F, 2D7G, 2D7H, 2D7I, 2D7J, 2D7R, 2D7S, 2D7T, 2D7U, 2D7V, 2D7Y, 2D7Z, 2D80, 2D81, 2D8A, 2D8D, 2D8E, 2D8L, 2D8N, 2D8O, 2D8P, 2D8W, 2D91, 2D94, 2D95, 2D97, 2D98, 2D9Q, 2D9R, 2DAA, 2DAB, 2DAP, 2DB0, 2DB4, 2DB7, 2DBB, 2DBE, 2DBI, 2DBL, 2DBN, 2DBO, 2DBQ, 2DBR, 2DBS, 2DBT, 2DBU, 2DBV, 2DBW, 2DBX, 2DBY, 2DBZ, 2DC0, 2DC1, 2DC3, 2DC4, 2DC5, 2DC6, 2DC7, 2DC8, 2DC9, 2DCA, 2DCB, 2DCC, 2DCD, 2DCF, 2DCG, 2DCH, 2DCJ, 2DCK, 2DCL, 2DCM, 2DCN, 2DCT, 2DCU, 2DCY, 2DCZ, 2DD4, 2DD5, 2DD7, 2DD8, 2DD9, 2DDA, 2DDB, 2DDC, 2DDD, 2DDF, 2DDG, 2DDH, 2DDK, 2DDM, 2DDO, 2DDQ, 2DDR, 2DDS, 2DDT, 2DDU, 2DDW, 2DDX, 2DDZ, 2DE0, 2DE2, 2DE3, 2DE4, 2DE5, 2DE6, 2DE7, 2DE8, 2DE9, 2DEA, 2DEB, 2DEC, 2DEG, 2DEH, 2DEI, 2DEJ, 2DEK, 2DEM, 2DEO, 2DEP, 2DEQ, 2DEV, 2DEW, 2DEX, 2DEY, 2DF3, 2DF4, 2DF5, 2DF6, 2DF8, 2DFA, 2DFB, 2DFC, 2DFD, 2DFE, 2DFF, 2DFH, 2DFI, 2DFJ, 2DFK, 2DFL, 2DFN, 2DFP, 2DFT, 2DFU, 2DFV, 2DFX, 2DFY, 2DG0, 2DG1, 2DG2, 2DG3, 2DG4, 2DG5, 2DG6, 2DG7, 2DG8, 2DG9, 2DGA, 2DGB, 2DGC, 2DGD, 2DGE, 2DGJ, 2DGK, 2DGL, 2DGM, 2DGN, 2DH1, 2DH2, 2DH3, 2DH4, 2DH5, 2DH6, 2DHB, 2DHC, 2DHD, 2DHE, 2DHF, 2DHH, 2DHN, 2DHO, 2DHQ, 2DHR, 2DHT, 2DI3, 2DI4, 2DIE, 2DIJ, 2DIK, 2DIO, 2DJ5, 2DJ6, 2DJF, 2DJG, 2DJH, 2DJI, 2DJL, 2DJW, 2DJX, 2DJZ, 2DKA, 2DKB, 2DKC, 2DKD, 2DKE, 2DKF, 2DKG, 2DKH, 2DKI, 2DKJ, 2DKK, 2DKN, 2DKO, 2DKV, 2DL2, 2DLA, 2DLB, 2DLD, 2DLF, 2DLI, 2DLN, 2DM5, 2DM6, 2DM9, 2DMA, 2DMR, 2DN1, 2DN2, 2DN3, 2DND, 2DNJ, 2DNS, 2DO2, 2DOB, 2DOH, 2DOI, 2DOJ, 2DOK, 2DOO, 2DOQ, 2DOR, 2DOU, 2DP3, 2DP4, 2DP5, 2DP6, 2DP7, 2DP8, 2DP9, 2DPB, 2DPC, 2DPD, 2DPE, 2DPF, 2DPG, 2DPH, 2DPI, 2DPJ, 2DPK, 2DPL, 2DPM, 2DPN, 2DPP, 2DPS, 2DPT, 2DPU, 2DPW, 2DPX, 2DPY, 2DPZ, 2DQ0, 2DQ3, 2DQ4, 2DQ6, 2DQ7, 2DQA, 2DQB, 2DQC, 2DQD, 2DQE, 2DQF, 2DQG, 2DQH, 2DQI, 2DQJ, 2DQK, 2DQL, 2DQM, 2DQN, 2DQR, 2DQS, 2DQT, 2DQU, 2DQV, 2DQW, 2DQX, 2DQY, 2DQZ, 2DR0, 2DR1, 2DR3, 2DR6, 2DRC, 2DRD, 2DRE, 2DRH, 2DRI, 2DRJ, 2DRK, 2DRM, 2DRO, 2DRP, 2DRQ, 2DRR, 2DRS, 2DRU, 2DRV, 2DRW, 2DRY, 2DRZ, 2DS0, 2DS1, 2DS2, 2DS5, 2DS6, 2DS7, 2DS8, 2DS9, 2DSA, 2DSB, 2DSC, 2DSD, 2DSF, 2DSG, 2DSH, 2DSI, 2DSJ, 2DSK, 2DSL, 2DSN, 2DSO, 2DSP, 2DSQ, 2DSR, 2DST, 2DSU, 2DSV, 2DSW, 2DSX, 2DSY, 2DSZ, 2DT0, 2DT1, 2DT2, 2DT3, 2DT4, 2DT5, 2DT8, 2DT9, 2DTC, 2DTD, 2DTE, 2DTG, 2DTH, 2DTI, 2DTJ, 2DTM, 2DTN, 2DTO, 2DTR, 2DTS, 2DTT, 2DTU, 2DTW, 2DTX, 2DTY, 2DTZ, 2DU0, 2DU1, 2DU2, 2DU7, 2DU8, 2DU9, 2DUA, 2DUB, 2DUC, 2DUD, 2DUE, 2DUF, 2DUG, 2DUH, 2DUI, 2DUJ, 2DUK, 2DUL, 2DUM, 2DUO, 2DUP, 2DUQ, 2DUR, 2DUT, 2DUU, 2DUV, 2DUX, 2DUY, 2DUZ, 2DV0, 2DV1, 2DV2, 2DV3, 2DV4, 2DV5, 2DV6, 2DV7, 2DV9, 2DVA, 2DVB, 2DVC, 2DVD, 2DVE, 2DVF, 2DVG, 2DVK, 2DVL, 2DVM, 2DVN, 2DVO, 2DVP, 2DVQ, 2DVR, 2DVS, 2DVT, 2DVU, 2DVV, 2DVW, 2DVX, 2DVY, 2DVZ, 2DW0, 2DW1, 2DW2, 2DW4, 2DW5, 2DW6, 2DW7, 2DWA, 2DWB, 2DWC, 2DWD, 2DWE, 2DWG, 2DWH, 2DWI, 2DWJ, 2DWK, 2DWL, 2DWM, 2DWN, 2DWO, 2DWP, 2DWQ, 2DWR, 2DWS, 2DWT, 2DWU, 2DWW, 2DWX, 2DWY, 2DWZ, 2DX0, 2DX1, 2DX5, 2DX6, 2DX7, 2DX8, 2DXA, 2DXB, 2DXC, 2DXD, 2DXE, 2DXF, 2DXL, 2DXN, 2DXP, 2DXQ, 2DXR, 2DXS, 2DXT, 2DXU, 2DXV, 2DXW, 2DXX, 2DXY, 2DY0, 2DY1, 2DY2, 2DY3, 2DY4, 2DY5, 2DY9, 2DYA, 2DYB, 2DYC, 2DYH, 2DYI, 2DYJ, 2DYK, 2DYL, 2DYM, 2DYN, 2DYO, 2DYP, 2DYQ, 2DYR, 2DYS, 2DYT, 2DYU, 2DYV, 2DYW, 2DYX, 2DYY, 2DZ7, 2DZ9, 2DZA, 2DZB, 2DZC, 2DZD, 2DZE, 2DZN, 2DZO, 2DZP, 2DZS, 2DZT, 2DZU, 2DZV, 2DZW, 2DZX, 2DZY, 2DZZ, 2E00, 2E01, 2E02, 2E03, 2E07, 2E08, 2E09, 2E0A, 2E0C, 2E0I, 2E0J, 2E0K, 2E0L, 2E0M, 2E0N, 2E0O, 2E0P, 2E0Q, 2E0S, 2E0T, 2E0W, 2E0X, 2E0Y, 2E10, 2E11, 2E12, 2E15, 2E16, 2E17, 2E18, 2E1A, 2E1B, 2E1C, 2E1D, 2E1E, 2E1F, 2E1H, 2E1M, 2E1N, 2E1P, 2E1Q, 2E1R, 2E1S, 2E1T, 2E1U, 2E1V, 2E1W, 2E1Y, 2E1Z, 2E20, 2E21, 2E22, 2E24, 2E25, 2E26, 2E27, 2E28, 2E2A, 2E2B, 2E2C, 2E2D, 2E2E, 2E2G, 2E2K, 2E2L, 2E2M, 2E2N, 2E2O, 2E2P, 2E2Q, 2E2R, 2E2T, 2E2U, 2E2V, 2E2X, 2E2Y, 2E31, 2E32, 2E33, 2E37, 2E39, 2E3A, 2E3B, 2E3C, 2E3D, 2E3H, 2E3I, 2E3J, 2E3K, 2E3M, 2E3N, 2E3O, 2E3P, 2E3Q, 2E3R, 2E3S, 2E3T, 2E3U, 2E3V, 2E3W, 2E3X, 2E3Z, 2E40, 2E41, 2E42, 2E43, 2E46, 2E47, 2E48, 2E49, 2E4A, 2E4F, 2E4G, 2E4L, 2E4M, 2E4N, 2E4O, 2E4P, 2E4Q, 2E4R, 2E4T, 2E4U, 2E4V, 2E4W, 2E4X, 2E4Y, 2E4Z, 2E50, 2E51, 2E52, 2E53, 2E54, 2E55, 2E56, 2E59, 2E5A, 2E5B, 2E5C, 2E5D, 2E5F, 2E5M, 2E5V, 2E5W, 2E5X, 2E5Y, 2E64, 2E65, 2E66, 2E67, 2E68, 2E69, 2E6A, 2E6B, 2E6C, 2E6D, 2E6E, 2E6F, 2E6G, 2E6H, 2E6K, 2E6L, 2E6M, 2E6U, 2E6V, 2E6X, 2E6Y, 2E74, 2E75, 2E76, 2E77, 2E7A, 2E7D, 2E7E, 2E7F, 2E7I, 2E7J, 2E7L, 2E7P, 2E7Q, 2E7R, 2E7S, 2E7T, 2E7U, 2E7V, 2E7W, 2E7X, 2E7Y, 2E7Z, 2E80, 2E81, 2E82, 2E83, 2E84, 2E85, 2E86, 2E87, 2E88, 2E89, 2E8A, 2E8B, 2E8C, 2E8E, 2E8F, 2E8G, 2E8H, 2E8I, 2E8Q, 2E8R, 2E8S, 2E8T, 2E8U, 2E8V, 2E8W, 2E8X, 2E8Y, 2E8Z, 2E90, 2E91, 2E92, 2E93, 2E94, 2E95, 2E98, 2E99, 2E9A, 2E9B, 2E9C, 2E9D, 2E9E, 2E9F, 2E9L, 2E9M, 2E9N, 2E9O, 2E9P, 2E9Q, 2E9S, 2E9U, 2E9V, 2E9W, 2E9X, 2E9Y, 2EA0, 2EA1, 2EA2, 2EA3, 2EA4, 2EA7, 2EA9, 2EAA, 2EAB, 2EAC, 2EAD, 2EAE, 2EAK, 2EAL, 2EAQ, 2EAR, 2EAS, 2EAT, 2EAU, 2EAV, 2EAW, 2EAX, 2EAY, 2EB0, 2EB1, 2EB2, 2EB3, 2EB4, 2EB5, 2EB6, 2EB7, 2EB8, 2EB9, 2EBA, 2EBB, 2EBD, 2EBE, 2EBF, 2EBG, 2EBH, 2EBJ, 2EBN, 2EBO, 2EBS, 2EBY, 2EC2, 2EC5, 2EC6, 2EC8, 2EC9, 2ECE, 2ECF, 2ECK, 2ECO, 2ECP, 2ECQ, 2ECR, 2ECS, 2ECU, 2ED3, 2ED4, 2ED5, 2ED6, 2EDA, 2EDC, 2EDM, 2EEK, 2EEN, 2EEO, 2EEP, 2EEQ, 2EER, 2EEX, 2EEY, 2EEZ, 2EF0, 2EF1, 2EF2, 2EF4, 2EF5, 2EF6, 2EF7, 2EF8, 2EF9, 2EFB, 2EFC, 2EFD, 2EFE, 2EFF, 2EFG, 2EFH, 2EFJ, 2EFK, 2EFL, 2EFN, 2EFO, 2EFP, 2EFQ, 2EFR, 2EFS, 2EFT, 2EFU, 2EFV, 2EFW, 2EFX, 2EFY, 2EG1, 2EG2, 2EG3, 2EG4, 2EG5, 2EG6, 2EG7, 2EG8, 2EG9, 2EGB, 2EGD, 2EGG, 2EGH, 2EGI, 2EGJ, 2EGK, 2EGL, 2EGN, 2EGO, 2EGR, 2EGS, 2EGT, 2EGU, 2EGV, 2EGW, 2EGY, 2EGZ, 2EH1, 2EH2, 2EH3, 2EH4, 2EH5, 2EH6, 2EH7, 2EH8, 2EH9, 2EHA, 2EHB, 2EHC, 2EHD, 2EHG, 2EHH, 2EHJ, 2EHL, 2EHO, 2EHP, 2EHQ, 2EHS, 2EHT, 2EHU, 2EHW, 2EHZ, 2EI0, 2EI1, 2EI2, 2EI3, 2EI4, 2EI5, 2EI6, 2EI7, 2EI8, 2EI9, 2EIA, 2EIB, 2EIC, 2EID, 2EIE, 2EIF, 2EIG, 2EIH, 2EII, 2EIJ, 2EIK, 2EIL, 2EIM, 2EIN, 2EIO, 2EIP, 2EIQ, 2EIR, 2EIS, 2EIT, 2EIU, 2EIV, 2EIW, 2EIX, 2EIY, 2EIZ, 2EJ0, 2EJ1, 2EJ2, 2EJ3, 2EJ5, 2EJ6, 2EJ8, 2EJ9, 2EJA, 2EJB, 2EJC, 2EJD, 2EJF, 2EJG, 2EJJ, 2EJK, 2EJL, 2EJN, 2EJQ, 2EJR, 2EJT, 2EJU, 2EJV, 2EJW, 2EJX, 2EJZ, 2EK0, 2EK1, 2EK2, 2EK3, 2EK4, 2EK5, 2EK6, 2EK7, 2EK8, 2EK9, 2EKA, 2EKB, 2EKC, 2EKD, 2EKE, 2EKG, 2EKL, 2EKM, 2EKN, 2EKP, 2EKQ, 2EKS, 2EKT, 2EKU, 2EKY, 2EKZ, 2EL0, 2EL1, 2EL2, 2EL3, 2EL7, 2EL9, 2ELA, 2ELB, 2ELC, 2ELD, 2ELE, 2ELF, 2ELG, 2EMD, 2EMN, 2EMO, 2EMQ, 2EMR, 2EMS, 2EMT, 2EMU, 2EN5, 2ENB, 2END, 2ENG, 2ENI, 2ENR, 2ENU, 2ENW, 2ENX, 2EO0, 2EO4, 2EO5, 2EO7, 2EO8, 2EOA, 2EP5, 2EP7, 2EPE, 2EPF, 2EPG, 2EPH, 2EPI, 2EPJ, 2EPK, 2EPL, 2EPM, 2EPN, 2EPO, 2EQ5, 2EQ6, 2EQ7, 2EQ8, 2EQ9, 2EQA, 2EQB, 2EQD, 2EQL, 2ER0, 2ER6, 2ER7, 2ER8, 2ER9, 2ERA, 2ERB, 2ERC, 2ERE, 2ERF, 2ERG, 2ERH, 2ERJ, 2ERK, 2ERL, 2ERO, 2ERP, 2ERQ, 2ERV, 2ERW, 2ERX, 2ERY, 2ERZ, 2ES0, 2ES2, 2ES3, 2ES4, 2ES7, 2ES9, 2ESA, 2ESB, 2ESC, 2ESD, 2ESF, 2ESH, 2ESK, 2ESL, 2ESM, 2ESN, 2ESO, 2ESP, 2ESQ, 2ESR, 2ESS, 2EST, 2ESU, 2ESV, 2ESW, 2ET1, 2ET2, 2ET6, 2ET7, 2ETA, 2ETB, 2ETC, 2ETD, 2ETE, 2ETF, 2ETH, 2ETJ, 2ETK, 2ETL, 2ETM, 2ETN, 2ETR, 2ETS, 2ETV, 2ETW, 2ETX, 2EU1, 2EU2, 2EU3, 2EU7, 2EU8, 2EU9, 2EUA, 2EUC, 2EUD, 2EUF, 2EUG, 2EUH, 2EUI, 2EUK, 2EUL, 2EUM, 2EUN, 2EUO, 2EUP, 2EUQ, 2EUR, 2EUS, 2EUT, 2EUU, 2EUV, 2EUW, 2EUX, 2EUZ, 2EV0, 2EV1, 2EV2, 2EV3, 2EV4, 2EV5, 2EV6, 2EV9, 2EVA, 2EVB, 2EVC, 2EVD, 2EVE, 2EVF, 2EVG, 2EVH, 2EVI, 2EVJ, 2EVK, 2EVL, 2EVM, 2EVO, 2EVP, 2EVR, 2EVS, 2EVT, 2EVU, 2EVV, 2EVW, 2EVX, 2EW0, 2EW1, 2EW2, 2EW5, 2EW6, 2EW7, 2EW8, 2EWA, 2EWB, 2EWC, 2EWD, 2EWE, 2EWF, 2EWG, 2EWH, 2EWI, 2EWJ, 2EWK, 2EWM, 2EWN, 2EWO, 2EWP, 2EWR, 2EWS, 2EWT, 2EWU, 2EWV, 2EWW, 2EWY, 2EX0, 2EX1, 2EX2, 2EX3, 2EX4, 2EX5, 2EX6, 2EX8, 2EX9, 2EXA, 2EXB, 2EXC, 2EXE, 2EXH, 2EXI, 2EXJ, 2EXK, 2EXL, 2EXM, 2EXO, 2EXR, 2EXS, 2EXT, 2EXU, 2EXV, 2EXW, 2EXX, 2EXY, 2EXZ, 2EY1, 2EY2, 2EY4, 2EY5, 2EY6, 2EYF, 2EYH, 2EYI, 2EYJ, 2EYL, 2EYM, 2EYN, 2EYO, 2EYP, 2EYQ, 2EYR, 2EYS, 2EYT, 2EYU, 2EZ0, 2EZ1, 2EZ2, 2EZ4, 2EZ7, 2EZ8, 2EZ9, 2EZT, 2EZU, 2EZV, 2F00, 2F01, 2F02, 2F03, 2F06, 2F07, 2F08, 2F0A, 2F0C, 2F0D, 2F0E, 2F0F, 2F0G, 2F0H, 2F0I, 2F0J, 2F0K, 2F0L, 2F0M, 2F0N, 2F0O, 2F0P, 2F0Q, 2F0R, 2F0S, 2F0T, 2F0U, 2F0V, 2F0W, 2F0X, 2F0Y, 2F0Z, 2F10, 2F11, 2F12, 2F13, 2F14, 2F15, 2F16, 2F17, 2F18, 2F19, 2F1A, 2F1B, 2F1C, 2F1D, 2F1F, 2F1G, 2F1H, 2F1I, 2F1J, 2F1K, 2F1L, 2F1M, 2F1N, 2F1O, 2F1R, 2F1S, 2F1T, 2F1V, 2F1W, 2F1X, 2F1Y, 2F1Z, 2F20, 2F21, 2F22, 2F23, 2F24, 2F25, 2F26, 2F27, 2F28, 2F29, 2F2A, 2F2B, 2F2C, 2F2E, 2F2F, 2F2G, 2F2H, 2F2K, 2F2L, 2F2N, 2F2O, 2F2P, 2F2Q, 2F2S, 2F2T, 2F2U, 2F2V, 2F2W, 2F2X, 2F30, 2F31, 2F32, 2F34, 2F35, 2F36, 2F37, 2F38, 2F3B, 2F3C, 2F3D, 2F3E, 2F3F, 2F3G, 2F3K, 2F3L, 2F3M, 2F3N, 2F3O, 2F3P, 2F3Q, 2F3R, 2F3S, 2F3T, 2F3U, 2F3X, 2F3Y, 2F3Z, 2F41, 2F42, 2F43, 2F44, 2F46, 2F47, 2F48, 2F49, 2F4A, 2F4B, 2F4E, 2F4F, 2F4G, 2F4I, 2F4J, 2F4K, 2F4L, 2F4M, 2F4N, 2F4O, 2F4P, 2F4Q, 2F4W, 2F4Y, 2F4Z, 2F51, 2F53, 2F54, 2F55, 2F56, 2F57, 2F58, 2F59, 2F5A, 2F5B, 2F5C, 2F5D, 2F5E, 2F5F, 2F5G, 2F5I, 2F5J, 2F5K, 2F5M, 2F5N, 2F5O, 2F5P, 2F5Q, 2F5S, 2F5T, 2F5U, 2F5V, 2F5W, 2F5X, 2F5Y, 2F5Z, 2F60, 2F61, 2F62, 2F64, 2F66, 2F67, 2F68, 2F69, 2F6A, 2F6B, 2F6C, 2F6D, 2F6E, 2F6F, 2F6G, 2F6H, 2F6I, 2F6J, 2F6K, 2F6L, 2F6M, 2F6N, 2F6P, 2F6Q, 2F6R, 2F6S, 2F6T, 2F6U, 2F6V, 2F6W, 2F6X, 2F6Y, 2F6Z, 2F70, 2F71, 2F73, 2F74, 2F78, 2F7A, 2F7B, 2F7C, 2F7D, 2F7E, 2F7F, 2F7I, 2F7K, 2F7L, 2F7M, 2F7N, 2F7O, 2F7P, 2F7Q, 2F7R, 2F7S, 2F7T, 2F7V, 2F7W, 2F7X, 2F7Y, 2F7Z, 2F80, 2F81, 2F82, 2F83, 2F84, 2F86, 2F89, 2F8A, 2F8C, 2F8D, 2F8E, 2F8F, 2F8G, 2F8H, 2F8I, 2F8J, 2F8L, 2F8M, 2F8N, 2F8O, 2F8P, 2F8Q, 2F8V, 2F8W, 2F8X, 2F8Y, 2F8Z, 2F90, 2F91, 2F92, 2F93, 2F94, 2F95, 2F96, 2F97, 2F98, 2F99, 2F9A, 2F9B, 2F9C, 2F9D, 2F9F, 2F9G, 2F9H, 2F9I, 2F9J, 2F9K, 2F9L, 2F9M, 2F9N, 2F9O, 2F9P, 2F9Q, 2F9R, 2F9S, 2F9T, 2F9U, 2F9V, 2F9W, 2F9Y, 2F9Z, 2FA0, 2FA1, 2FA2, 2FA3, 2FA4, 2FA5, 2FA7, 2FA8, 2FA9, 2FAC, 2FAD, 2FAE, 2FAF, 2FAH, 2FAI, 2FAK, 2FAL, 2FAM, 2FAO, 2FAP, 2FAQ, 2FAR, 2FAT, 2FAU, 2FAV, 2FAW, 2FAX, 2FAZ, 2FB0, 2FB1, 2FB2, 2FB3, 2FB4, 2FB5, 2FB6, 2FB8, 2FB9, 2FBA, 2FBB, 2FBD, 2FBE, 2FBH, 2FBI, 2FBJ, 2FBK, 2FBL, 2FBM, 2FBN, 2FBO, 2FBP, 2FBQ, 2FBR, 2FBT, 2FBV, 2FBW, 2FBX, 2FBY, 2FBZ, 2FC0, 2FC1, 2FC2, 2FC3, 2FCA, 2FCB, 2FCC, 2FCF, 2FCH, 2FCJ, 2FCK, 2FCL, 2FCM, 2FCN, 2FCO, 2FCP, 2FCQ, 2FCR, 2FCS, 2FCT, 2FCU, 2FCV, 2FCW, 2FD2, 2FD3, 2FD4, 2FD5, 2FD6, 2FD7, 2FD8, 2FD9, 2FDA, 2FDB, 2FDC, 2FDD, 2FDE, 2FDF, 2FDG, 2FDH, 2FDI, 2FDJ, 2FDK, 2FDM, 2FDN, 2FDO, 2FDP, 2FDQ, 2FDR, 2FDS, 2FDU, 2FDV, 2FDW, 2FDX, 2FDY, 2FE1, 2FE3, 2FE4, 2FE5, 2FE6, 2FE7, 2FE8, 2FEA, 2FEC, 2FED, 2FEE, 2FEF, 2FEL, 2FEM, 2FEN, 2FEO, 2FEP, 2FEQ, 2FER, 2FES, 2FET, 2FEU, 2FEX, 2FEZ, 2FF1, 2FF2, 2FF3, 2FF4, 2FF5, 2FF6, 2FF7, 2FFA, 2FFB, 2FFC, 2FFD, 2FFF, 2FFG, 2FFH, 2FFI, 2FFJ, 2FFL, 2FFM, 2FFN, 2FFQ, 2FFR, 2FFS, 2FFU, 2FFV, 2FFX, 2FFY, 2FFZ, 2FG0, 2FG1, 2FG4, 2FG5, 2FG6, 2FG7, 2FG8, 2FG9, 2FGB, 2FGC, 2FGE, 2FGF, 2FGG, 2FGH, 2FGI, 2FGJ, 2FGK, 2FGL, 2FGN, 2FGO, 2FGQ, 2FGR, 2FGS, 2FGT, 2FGU, 2FGV, 2FGW, 2FGY, 2FGZ, 2FH1, 2FH2, 2FH3, 2FH4, 2FH5, 2FH6, 2FH7, 2FH8, 2FH9, 2FHA, 2FHB, 2FHC, 2FHD, 2FHE, 2FHF, 2FHG, 2FHH, 2FHI, 2FHJ, 2FHK, 2FHL, 2FHN, 2FHP, 2FHQ, 2FHR, 2FHS, 2FHT, 2FHX, 2FHY, 2FHZ, 2FI0, 2FI1, 2FI3, 2FI4, 2FI5, 2FI7, 2FI9, 2FIA, 2FIB, 2FIC, 2FID, 2FIE, 2FIF, 2FIH, 2FII, 2FIK, 2FIM, 2FIO, 2FIP, 2FIQ, 2FIR, 2FIT, 2FIU, 2FIV, 2FIW, 2FIX, 2FIY, 2FJ0, 2FJ1, 2FJ2, 2FJ7, 2FJ8, 2FJ9, 2FJA, 2FJB, 2FJC, 2FJD, 2FJE, 2FJF, 2FJG, 2FJH, 2FJI, 2FJK, 2FJM, 2FJN, 2FJP, 2FJR, 2FJS, 2FJT, 2FJU, 2FJV, 2FJW, 2FJX, 2FJY, 2FJZ, 2FK0, 2FK1, 2FK2, 2FK3, 2FK5, 2FK7, 2FK8, 2FK9, 2FKA, 2FKB, 2FKC, 2FKD, 2FKE, 2FKF, 2FKG, 2FKH, 2FKJ, 2FKK, 2FKL, 2FKM, 2FKN, 2FKO, 2FKP, 2FKY, 2FKZ, 2FL0, 2FL1, 2FL2, 2FL3, 2FL4, 2FL5, 2FL6, 2FL7, 2FLA, 2FLB, 2FLC, 2FLD, 2FLE, 2FLF, 2FLH, 2FLI, 2FLK, 2FLL, 2FLM, 2FLN, 2FLO, 2FLP, 2FLQ, 2FLR, 2FLS, 2FLT, 2FLU, 2FLV, 2FLW, 2FLZ, 2FM0, 2FM1, 2FM2, 2FM3, 2FM5, 2FM6, 2FM7, 2FM8, 2FM9, 2FMA, 2FMB, 2FME, 2FMF, 2FMG, 2FMH, 2FMI, 2FMJ, 2FMK, 2FML, 2FMM, 2FMN, 2FMO, 2FMP, 2FMQ, 2FMS, 2FMU, 2FMX, 2FMY, 2FMZ, 2FN0, 2FN1, 2FN3, 2FN4, 2FN6, 2FN7, 2FN8, 2FN9, 2FNA, 2FNC, 2FNE, 2FNI, 2FNJ, 2FNK, 2FNM, 2FNN, 2FNO, 2FNP, 2FNQ, 2FNS, 2FNT, 2FNU, 2FNW, 2FNX, 2FNZ, 2FO0, 2FO1, 2FO3, 2FO4, 2FO5, 2FO7, 2FO9, 2FOA, 2FOB, 2FOC, 2FOD, 2FOE, 2FOF, 2FOG, 2FOH, 2FOI, 2FOJ, 2FOK, 2FOL, 2FOM, 2FON, 2FOO, 2FOP, 2FOQ, 2FOR, 2FOS, 2FOT, 2FOU, 2FOV, 2FOX, 2FOY, 2FOZ, 2FP0, 2FP1, 2FP2, 2FP3, 2FP4, 2FP7, 2FP8, 2FP9, 2FPB, 2FPC, 2FPD, 2FPE, 2FPF, 2FPG, 2FPH, 2FPI, 2FPK, 2FPL, 2FPM, 2FPN, 2FPO, 2FPP, 2FPQ, 2FPR, 2FPS, 2FPT, 2FPU, 2FPV, 2FPW, 2FPX, 2FPY, 2FPZ, 2FQ1, 2FQ3, 2FQ4, 2FQ6, 2FQ9, 2FQD, 2FQE, 2FQF, 2FQG, 2FQI, 2FQL, 2FQM, 2FQO, 2FQP, 2FQQ, 2FQT, 2FQW, 2FQX, 2FQY, 2FQZ, 2FR0, 2FR1, 2FR2, 2FR3, 2FR4, 2FR5, 2FR6, 2FR7, 2FR8, 2FRA, 2FRD, 2FRE, 2FRF, 2FRG, 2FRH, 2FRI, 2FRJ, 2FRK, 2FRM, 2FRQ, 2FRS, 2FRV, 2FRX, 2FRZ, 2FS2, 2FS4, 2FS5, 2FS6, 2FS7, 2FS8, 2FS9, 2FSA, 2FSD, 2FSE, 2FSF, 2FSG, 2FSH, 2FSI, 2FSJ, 2FSK, 2FSL, 2FSM, 2FSN, 2FSO, 2FSQ, 2FSR, 2FSS, 2FST, 2FSU, 2FSV, 2FSW, 2FSX, 2FSZ, 2FT0, 2FT2, 2FT3, 2FT6, 2FT7, 2FT8, 2FT9, 2FTA, 2FTB, 2FTD, 2FTK, 2FTL, 2FTM, 2FTN, 2FTO, 2FTP, 2FTQ, 2FTR, 2FTS, 2FTW, 2FTX, 2FTY, 2FTZ, 2FU0, 2FU2, 2FU3, 2FU4, 2FU5, 2FU6, 2FU7, 2FU8, 2FU9, 2FUA, 2FUB, 2FUC, 2FUD, 2FUE, 2FUF, 2FUG, 2FUJ, 2FUK, 2FUL, 2FUM, 2FUN, 2FUP, 2FUQ, 2FUR, 2FUS, 2FUT, 2FUV, 2FUZ, 2FV0, 2FV1, 2FV2, 2FV5, 2FV7, 2FV8, 2FV9, 2FVC, 2FVD, 2FVG, 2FVH, 2FVJ, 2FVK, 2FVL, 2FVM, 2FVP, 2FVQ, 2FVR, 2FVS, 2FVU, 2FVV, 2FVX, 2FVY, 2FVZ, 2FW0, 2FW1, 2FW2, 2FW3, 2FW4, 2FW5, 2FW6, 2FW7, 2FW8, 2FW9, 2FWA, 2FWB, 2FWE, 2FWF, 2FWG, 2FWH, 2FWI, 2FWJ, 2FWM, 2FWN, 2FWO, 2FWP, 2FWQ, 2FWR, 2FWT, 2FWV, 2FWW, 2FWY, 2FWZ, 2FX0, 2FX2, 2FX3, 2FX4, 2FX5, 2FX6, 2FX7, 2FX8, 2FX9, 2FXA, 2FXD, 2FXE, 2FXF, 2FXG, 2FXH, 2FXI, 2FXJ, 2FXK, 2FXL, 2FXM, 2FXO, 2FXQ, 2FXR, 2FXS, 2FXT, 2FXU, 2FXV, 2FY2, 2FY3, 2FY4, 2FY5, 2FY6, 2FY7, 2FY8, 2FYA, 2FYB, 2FYC, 2FYD, 2FYE, 2FYF, 2FYG, 2FYI, 2FYK, 2FYM, 2FYN, 2FYO, 2FYP, 2FYQ, 2FYR, 2FYS, 2FYT, 2FYU, 2FYV, 2FYW, 2FYX, 2FYY, 2FYZ, 2FZ3, 2FZ4, 2FZ6, 2FZ8, 2FZ9, 2FZA, 2FZB, 2FZC, 2FZD, 2FZE, 2FZF, 2FZG, 2FZH, 2FZI, 2FZJ, 2FZK, 2FZL, 2FZM, 2FZN, 2FZP, 2FZS, 2FZT, 2FZU, 2FZV, 2FZW, 2FZZ, 2G00, 2G01, 2G02, 2G03, 2G04, 2G06, 2G07, 2G08, 2G09, 2G0A, 2G0B, 2G0C, 2G0D, 2G0E, 2G0F, 2G0G, 2G0H, 2G0I, 2G0J, 2G0N, 2G0R, 2G0S, 2G0T, 2G0V, 2G0W, 2G0X, 2G0Y, 2G0Z, 2G10, 2G11, 2G12, 2G13, 2G14, 2G15, 2G16, 2G17, 2G18, 2G19, 2G1A, 2G1H, 2G1J, 2G1K, 2G1L, 2G1M, 2G1N, 2G1O, 2G1P, 2G1Q, 2G1R, 2G1S, 2G1T, 2G1U, 2G1Y, 2G1Z, 2G20, 2G21, 2G22, 2G24, 2G25, 2G26, 2G27, 2G28, 2G29, 2G2C, 2G2D, 2G2F, 2G2H, 2G2I, 2G2L, 2G2N, 2G2O, 2G2P, 2G2Q, 2G2R, 2G2S, 2G2U, 2G2W, 2G2X, 2G2Y, 2G2Z, 2G30, 2G36, 2G37, 2G38, 2G39, 2G3A, 2G3B, 2G3D, 2G3F, 2G3H, 2G3I, 2G3J, 2G3K, 2G3M, 2G3N, 2G3O, 2G3P, 2G3R, 2G3T, 2G3V, 2G3W, 2G3X, 2G3Y, 2G3Z, 2G40, 2G41, 2G42, 2G43, 2G44, 2G45, 2G47, 2G48, 2G49, 2G4C, 2G4D, 2G4E, 2G4F, 2G4G, 2G4H, 2G4I, 2G4J, 2G4K, 2G4L, 2G4M, 2G4N, 2G4O, 2G4P, 2G4Q, 2G4R, 2G4S, 2G4T, 2G4U, 2G4V, 2G4W, 2G4X, 2G4Y, 2G4Z, 2G50, 2G51, 2G52, 2G54, 2G55, 2G56, 2G58, 2G59, 2G5B, 2G5C, 2G5D, 2G5F, 2G5G, 2G5H, 2G5I, 2G5L, 2G5N, 2G5O, 2G5P, 2G5R, 2G5T, 2G5U, 2G5V, 2G5W, 2G5X, 2G5Z, 2G60, 2G62, 2G63, 2G64, 2G67, 2G69, 2G6B, 2G6D, 2G6E, 2G6F, 2G6G, 2G6H, 2G6I, 2G6J, 2G6K, 2G6L, 2G6M, 2G6N, 2G6O, 2G6P, 2G6Q, 2G6T, 2G6V, 2G6W, 2G6X, 2G6Y, 2G6Z, 2G70, 2G71, 2G72, 2G73, 2G74, 2G75, 2G76, 2G77, 2G78, 2G79, 2G7B, 2G7C, 2G7E, 2G7F, 2G7G, 2G7I, 2G7K, 2G7L, 2G7M, 2G7N, 2G7O, 2G7P, 2G7Q, 2G7R, 2G7S, 2G7U, 2G7Y, 2G7Z, 2G80, 2G81, 2G82, 2G83, 2G84, 2G85, 2G86, 2G87, 2G88, 2G89, 2G8A, 2G8C, 2G8D, 2G8E, 2G8J, 2G8L, 2G8N, 2G8Q, 2G8R, 2G8S, 2G8T, 2G8X, 2G8Y, 2G8Z, 2G93, 2G94, 2G95, 2G96, 2G97, 2G98, 2G99, 2G9A, 2G9D, 2G9E, 2G9F, 2G9G, 2G9H, 2G9I, 2G9K, 2G9N, 2G9Q, 2G9R, 2G9T, 2G9U, 2G9V, 2G9W, 2G9X, 2G9Y, 2G9Z, 2GA0, 2GA1, 2GA2, 2GA3, 2GA4, 2GA6, 2GA8, 2GA9, 2GAA, 2GAB, 2GAC, 2GAE, 2GAF, 2GAG, 2GAH, 2GAI, 2GAJ, 2GAK, 2GAL, 2GAM, 2GAN, 2GAO, 2GAR, 2GAS, 2GAU, 2GAW, 2GAX, 2GAZ, 2GB0, 2GB2, 2GB3, 2GB4, 2GB5, 2GB7, 2GB9, 2GBA, 2GBB, 2GBC, 2GBF, 2GBG, 2GBI, 2GBJ, 2GBK, 2GBL, 2GBM, 2GBN, 2GBO, 2GBP, 2GBR, 2GBT, 2GBU, 2GBV, 2GBW, 2GBX, 2GBY, 2GBZ, 2GC0, 2GC1, 2GC2, 2GC3, 2GC4, 2GC5, 2GC6, 2GC7, 2GC8, 2GC9, 2GCA, 2GCB, 2GCD, 2GCE, 2GCG, 2GCH, 2GCI, 2GCJ, 2GCL, 2GCN, 2GCO, 2GCP, 2GCQ, 2GCT, 2GCU, 2GCY, 2GD0, 2GD1, 2GD2, 2GD4, 2GD5, 2GD6, 2GD8, 2GD9, 2GDC, 2GDD, 2GDE, 2GDF, 2GDG, 2GDJ, 2GDM, 2GDN, 2GDO, 2GDQ, 2GDR, 2GDS, 2GDU, 2GDV, 2GDZ, 2GE3, 2GE5, 2GE7, 2GE8, 2GEB, 2GEC, 2GED, 2GEE, 2GEF, 2GEH, 2GEJ, 2GEK, 2GEL, 2GEM, 2GEN, 2GEP, 2GEQ, 2GER, 2GES, 2GET, 2GEU, 2GEV, 2GEW, 2GEX, 2GEY, 2GEZ, 2GF0, 2GF2, 2GF3, 2GF4, 2GF6, 2GF7, 2GF9, 2GFA, 2GFB, 2GFC, 2GFD, 2GFE, 2GFF, 2GFG, 2GFH, 2GFI, 2GFJ, 2GFK, 2GFN, 2GFO, 2GFP, 2GFQ, 2GFS, 2GFT, 2GFV, 2GFW, 2GFX, 2GFY, 2GG0, 2GG2, 2GG3, 2GG4, 2GG5, 2GG6, 2GG7, 2GG8, 2GG9, 2GGA, 2GGB, 2GGC, 2GGD, 2GGE, 2GGG, 2GGH, 2GGI, 2GGJ, 2GGK, 2GGL, 2GGM, 2GGN, 2GGO, 2GGQ, 2GGS, 2GGT, 2GGU, 2GGV, 2GGX, 2GGZ, 2GH0, 2GH1, 2GH2, 2GH4, 2GH5, 2GH6, 2GH7, 2GH9, 2GHA, 2GHB, 2GHC, 2GHD, 2GHE, 2GHG, 2GHH, 2GHI, 2GHJ, 2GHK, 2GHL, 2GHM, 2GHO, 2GHP, 2GHQ, 2GHR, 2GHS, 2GHT, 2GHU, 2GHV, 2GHW, 2GHY, 2GHZ, 2GI0, 2GI3, 2GI7, 2GI9, 2GIA, 2GIB, 2GID, 2GIE, 2GIF, 2GIG, 2GIH, 2GII, 2GIJ, 2GIL, 2GIM, 2GIN, 2GIQ, 2GIR, 2GIT, 2GIU, 2GIV, 2GIX, 2GIY, 2GIZ, 2GJ1, 2GJ2, 2GJ3, 2GJ4, 2GJ5, 2GJ6, 2GJ7, 2GJ8, 2GJ9, 2GJA, 2GJB, 2GJD, 2GJG, 2GJJ, 2GJK, 2GJL, 2GJM, 2GJN, 2GJP, 2GJR, 2GJS, 2GJT, 2GJU, 2GJV, 2GJX, 2GJZ, 2GK0, 2GK1, 2GK2, 2GK3, 2GK4, 2GK6, 2GK7, 2GK9, 2GKE, 2GKG, 2GKI, 2GKJ, 2GKL, 2GKM, 2GKN, 2GKO, 2GKP, 2GKR, 2GKS, 2GKT, 2GKV, 2GKW, 2GL0, 2GL2, 2GL3, 2GL5, 2GL6, 2GL7, 2GL8, 2GL9, 2GLF, 2GLI, 2GLJ, 2GLK, 2GLL, 2GLM, 2GLN, 2GLP, 2GLQ, 2GLR, 2GLS, 2GLT, 2GLU, 2GLV, 2GLX, 2GLZ, 2GM1, 2GM3, 2GM4, 2GM5, 2GM6, 2GM7, 2GM8, 2GM9, 2GME, 2GMF, 2GMH, 2GMI, 2GMJ, 2GMK, 2GML, 2GMM, 2GMN, 2GMP, 2GMQ, 2GMR, 2GMS, 2GMT, 2GMU, 2GMV, 2GMW, 2GMX, 2GMY, 2GN0, 2GN1, 2GN2, 2GN3, 2GN4, 2GN5, 2GN6, 2GN7, 2GN8, 2GN9, 2GNA, 2GNB, 2GNC, 2GND, 2GNF, 2GNG, 2GNH, 2GNI, 2GNJ, 2GNK, 2GNL, 2GNM, 2GNN, 2GNO, 2GNP, 2GNQ, 2GNS, 2GNT, 2GNU, 2GNV, 2GNW, 2GNX, 2GO1, 2GO2, 2GO3, 2GO4, 2GO7, 2GO8, 2GOI, 2GOJ, 2GOK, 2GOL, 2GOM, 2GON, 2GOO, 2GOP, 2GOT, 2GOU, 2GOX, 2GOY, 2GP0, 2GP3, 2GP4, 2GP5, 2GP6, 2GP7, 2GP9, 2GPA, 2GPB, 2GPC, 2GPE, 2GPH, 2GPI, 2GPJ, 2GPL, 2GPN, 2GPO, 2GPP, 2GPR, 2GPS, 2GPT, 2GPU, 2GPV, 2GPW, 2GPY, 2GPZ, 2GQ0, 2GQ1, 2GQ2, 2GQ3, 2GQ8, 2GQ9, 2GQA, 2GQD, 2GQF, 2GQG, 2GQN, 2GQP, 2GQQ, 2GQR, 2GQS, 2GQT, 2GQU, 2GQV, 2GQW, 2GQX, 2GR0, 2GR1, 2GR2, 2GR3, 2GR6, 2GR7, 2GR8, 2GR9, 2GRA, 2GRC, 2GRE, 2GRF, 2GRH, 2GRJ, 2GRK, 2GRL, 2GRM, 2GRN, 2GRO, 2GRP, 2GRQ, 2GRR, 2GRT, 2GRU, 2GRV, 2GRX, 2GRY, 2GRZ, 2GS2, 2GS3, 2GS4, 2GS5, 2GS6, 2GS7, 2GS8, 2GS9, 2GSA, 2GSC, 2GSD, 2GSE, 2GSF, 2GSG, 2GSH, 2GSI, 2GSJ, 2GSK, 2GSL, 2GSM, 2GSN, 2GSO, 2GSP, 2GSQ, 2GSR, 2GSS, 2GST, 2GSU, 2GSV, 2GSW, 2GSZ, 2GT1, 2GT2, 2GT4, 2GT7, 2GT8, 2GT9, 2GTA, 2GTB, 2GTC, 2GTD, 2GTE, 2GTF, 2GTG, 2GTH, 2GTI, 2GTK, 2GTM, 2GTN, 2GTP, 2GTQ, 2GTR, 2GTS, 2GTU, 2GTW, 2GTX, 2GTY, 2GTZ, 2GU0, 2GU1, 2GU2, 2GU3, 2GU4, 2GU5, 2GU6, 2GU7, 2GU8, 2GU9, 2GUB, 2GUC, 2GUD, 2GUE, 2GUF, 2GUG, 2GUH, 2GUI, 2GUJ, 2GUK, 2GUM, 2GUO, 2GUP, 2GUS, 2GUU, 2GUV, 2GUW, 2GUX, 2GUY, 2GUZ, 2GV0, 2GV2, 2GV5, 2GV6, 2GV7, 2GV8, 2GV9, 2GVC, 2GVD, 2GVF, 2GVG, 2GVH, 2GVI, 2GVJ, 2GVK, 2GVL, 2GVM, 2GVN, 2GVQ, 2GVR, 2GVU, 2GVV, 2GVW, 2GVX, 2GVY, 2GVZ, 2GW0, 2GW1, 2GW2, 2GW3, 2GW4, 2GW5, 2GW8, 2GWA, 2GWC, 2GWD, 2GWE, 2GWF, 2GWG, 2GWH, 2GWJ, 2GWK, 2GWL, 2GWM, 2GWN, 2GWO, 2GWQ, 2GWR, 2GWS, 2GWW, 2GWX, 2GX0, 2GX2, 2GX4, 2GX5, 2GX6, 2GX8, 2GX9, 2GXA, 2GXF, 2GXG, 2GXQ, 2GXS, 2GXU, 2GY5, 2GY7, 2GYD, 2GYI, 2GYK, 2GYO, 2GYP, 2GYQ, 2GYR, 2GYS, 2GYU, 2GYV, 2GYW, 2GYX, 2GYY, 2GYZ, 2GZ1, 2GZ2, 2GZ3, 2GZ4, 2GZ5, 2GZ6, 2GZ7, 2GZ8, 2GZ9, 2GZA, 2GZB, 2GZD, 2GZE, 2GZF, 2GZG, 2GZH, 2GZI, 2GZJ, 2GZL, 2GZM, 2GZQ, 2GZR, 2GZS, 2GZV, 2GZW, 2GZX, 2H00, 2H01, 2H02, 2H03, 2H04, 2H05, 2H06, 2H07, 2H08, 2H09, 2H0A, 2H0B, 2H0D, 2H0E, 2H0F, 2H0G, 2H0H, 2H0I, 2H0J, 2H0K, 2H0L, 2H0M, 2H0Q, 2H0R, 2H0T, 2H0U, 2H0V, 2H0Y, 2H10, 2H11, 2H12, 2H13, 2H14, 2H15, 2H16, 2H17, 2H18, 2H19, 2H1A, 2H1B, 2H1C, 2H1D, 2H1E, 2H1F, 2H1G, 2H1H, 2H1I, 2H1J, 2H1K, 2H1L, 2H1N, 2H1O, 2H1P, 2H1R, 2H1S, 2H1T, 2H1U, 2H1V, 2H1W, 2H1X, 2H1Y, 2H21, 2H23, 2H24, 2H26, 2H27, 2H28, 2H29, 2H2A, 2H2B, 2H2C, 2H2D, 2H2E, 2H2F, 2H2G, 2H2H, 2H2I, 2H2J, 2H2K, 2H2N, 2H2P, 2H2Q, 2H2R, 2H2S, 2H2T, 2H2U, 2H2W, 2H2Y, 2H2Z, 2H30, 2H31, 2H32, 2H34, 2H36, 2H39, 2H3B, 2H3D, 2H3E, 2H3G, 2H3H, 2H3L, 2H3M, 2H3N, 2H3P, 2H3R, 2H3U, 2H3W, 2H3X, 2H40, 2H42, 2H43, 2H44, 2H46, 2H47, 2H48, 2H4C, 2H4E, 2H4F, 2H4G, 2H4H, 2H4I, 2H4J, 2H4K, 2H4L, 2H4M, 2H4N, 2H4O, 2H4P, 2H4Q, 2H4R, 2H4T, 2H4U, 2H4V, 2H4W, 2H4X, 2H4Y, 2H4Z, 2H51, 2H52, 2H54, 2H55, 2H56, 2H57, 2H58, 2H59, 2H5A, 2H5C, 2H5D, 2H5E, 2H5F, 2H5G, 2H5I, 2H5J, 2H5K, 2H5L, 2H5N, 2H5O, 2H5P, 2H5Q, 2H5R, 2H5S, 2H5U, 2H5X, 2H5Y, 2H5Z, 2H61, 2H62, 2H63, 2H64, 2H65, 2H66, 2H68, 2H6A, 2H6B, 2H6C, 2H6D, 2H6E, 2H6F, 2H6G, 2H6H, 2H6I, 2H6J, 2H6K, 2H6L, 2H6M, 2H6N, 2H6P, 2H6Q, 2H6R, 2H6S, 2H6T, 2H6U, 2H6V, 2H6X, 2H6Y, 2H6Z, 2H70, 2H71, 2H72, 2H73, 2H74, 2H75, 2H76, 2H77, 2H79, 2H7C, 2H7F, 2H7G, 2H7H, 2H7J, 2H7O, 2H7Q, 2H7R, 2H7S, 2H7V, 2H7W, 2H7X, 2H7Y, 2H7Z, 2H84, 2H85, 2H88, 2H89, 2H8C, 2H8D, 2H8E, 2H8F, 2H8G, 2H8H, 2H8I, 2H8K, 2H8L, 2H8M, 2H8N, 2H8O, 2H8P, 2H8Q, 2H8R, 2H8U, 2H8V, 2H8X, 2H8Z, 2H90, 2H92, 2H94, 2H96, 2H98, 2H99, 2H9A, 2H9B, 2H9C, 2H9D, 2H9E, 2H9F, 2H9G, 2H9H, 2H9I, 2H9J, 2H9K, 2H9L, 2H9M, 2H9N, 2H9P, 2H9T, 2H9U, 2H9V, 2H9W, 2H9Y, 2HA0, 2HA2, 2HA3, 2HA4, 2HA5, 2HA6, 2HA7, 2HA8, 2HA9, 2HAD, 2HAE, 2HAF, 2HAG, 2HAH, 2HAI, 2HAK, 2HAL, 2HAM, 2HAN, 2HAP, 2HAQ, 2HAR, 2HAS, 2HAU, 2HAV, 2HAW, 2HAX, 2HAY, 2HAZ, 2HB0, 2HB1, 2HB2, 2HB3, 2HB4, 2HB5, 2HB6, 2HB7, 2HB8, 2HB9, 2HBA, 2HBB, 2HBC, 2HBD, 2HBE, 2HBF, 2HBG, 2HBH, 2HBJ, 2HBK, 2HBL, 2HBM, 2HBN, 2HBO, 2HBQ, 2HBR, 2HBS, 2HBT, 2HBU, 2HBV, 2HBW, 2HBX, 2HBY, 2HBZ, 2HC0, 2HC1, 2HC2, 2HC4, 2HC7, 2HC8, 2HC9, 2HCA, 2HCB, 2HCD, 2HCF, 2HCG, 2HCH, 2HCI, 2HCJ, 2HCK, 2HCM, 2HCN, 2HCO, 2HCR, 2HCS, 2HCT, 2HCU, 2HCV, 2HCZ, 2HD0, 2HD1, 2HD3, 2HD4, 2HD5, 2HD6, 2HD9, 2HDA, 2HDB, 2HDD, 2HDF, 2HDH, 2HDI, 2HDJ, 2HDK, 2HDN, 2HDO, 2HDQ, 2HDR, 2HDS, 2HDU, 2HDV, 2HDW, 2HDX, 2HDZ, 2HE0, 2HE2, 2HE3, 2HE4, 2HE5, 2HE7, 2HE8, 2HE9, 2HEA, 2HEB, 2HEC, 2HED, 2HEE, 2HEF, 2HEG, 2HEH, 2HEI, 2HEJ, 2HEK, 2HEL, 2HEN, 2HEO, 2HES, 2HET, 2HEU, 2HEV, 2HEW, 2HEX, 2HEY, 2HEZ, 2HF0, 2HF1, 2HF2, 2HF3, 2HF4, 2HF7, 2HF8, 2HF9, 2HFB, 2HFC, 2HFE, 2HFF, 2HFG, 2HFJ, 2HFK, 2HFN, 2HFO, 2HFP, 2HFS, 2HFT, 2HFU, 2HFW, 2HFZ, 2HG0, 2HG1, 2HG2, 2HG3, 2HG4, 2HG5, 2HG8, 2HG9, 2HGD, 2HGS, 2HGT, 2HGV, 2HGW, 2HGX, 2HGY, 2HGZ, 2HH0, 2HH1, 2HH5, 2HH6, 2HH7, 2HH9, 2HHA, 2HHB, 2HHC, 2HHD, 2HHE, 2HHF, 2HHG, 2HHJ, 2HHK, 2HHL, 2HHM, 2HHN, 2HHP, 2HHQ, 2HHS, 2HHT, 2HHU, 2HHV, 2HHW, 2HHX, 2HHZ, 2HI0, 2HI1, 2HI2, 2HI4, 2HI7, 2HI8, 2HI9, 2HIA, 2HIB, 2HIG, 2HIH, 2HII, 2HIJ, 2HIK, 2HIM, 2HIN, 2HIO, 2HIP, 2HIQ, 2HIS, 2HIT, 2HIV, 2HIW, 2HIX, 2HIY, 2HIZ, 2HJ0, 2HJ1, 2HJ3, 2HJ4, 2HJ6, 2HJ9, 2HJB, 2HJD, 2HJE, 2HJF, 2HJG, 2HJH, 2HJK, 2HJL, 2HJM, 2HJN, 2HJO, 2HJP, 2HJR, 2HJS, 2HJV, 2HJW, 2HK0, 2HK1, 2HK2, 2HK3, 2HK5, 2HK6, 2HK7, 2HK8, 2HK9, 2HKA, 2HKD, 2HKE, 2HKF, 2HKH, 2HKI, 2HKJ, 2HKK, 2HKL, 2HKM, 2HKN, 2HKO, 2HKP, 2HKQ, 2HKR, 2HKU, 2HKV, 2HKX, 2HKZ, 2HL0, 2HL1, 2HL2, 2HL3, 2HL4, 2HL5, 2HL6, 2HL7, 2HL8, 2HL9, 2HLA, 2HLC, 2HLD, 2HLE, 2HLF, 2HLH, 2HLJ, 2HLN, 2HLO, 2HLP, 2HLQ, 2HLR, 2HLS, 2HLV, 2HLY, 2HLZ, 2HM1, 2HM7, 2HMA, 2HMB, 2HMC, 2HMF, 2HMG, 2HMH, 2HMI, 2HMJ, 2HMK, 2HML, 2HMM, 2HMN, 2HMO, 2HMP, 2HMQ, 2HMS, 2HMT, 2HMU, 2HMV, 2HMW, 2HMY, 2HMZ, 2HN1, 2HN2, 2HN7, 2HN9, 2HNC, 2HND, 2HNE, 2HNF, 2HNG, 2HNH, 2HNI, 2HNK, 2HNL, 2HNP, 2HNQ, 2HNT, 2HNU, 2HNV, 2HNW, 2HNX, 2HNY, 2HNZ, 2HO0, 2HO1, 2HO2, 2HO4, 2HO5, 2HOB, 2HOC, 2HOD, 2HOE, 2HOF, 2HOG, 2HOH, 2HOI, 2HOQ, 2HOR, 2HOS, 2HOT, 2HOW, 2HOX, 2HOY, 2HOZ, 2HP0, 2HP1, 2HP2, 2HP3, 2HP4, 2HP5, 2HP6, 2HP7, 2HP9, 2HPA, 2HPB, 2HPC, 2HPD, 2HPE, 2HPF, 2HPG, 2HPH, 2HPI, 2HPJ, 2HPL, 2HPM, 2HPO, 2HPP, 2HPQ, 2HPR, 2HPS, 2HPT, 2HPV, 2HPW, 2HPY, 2HPZ, 2HQ0, 2HQ1, 2HQ4, 2HQ5, 2HQ6, 2HQ7, 2HQ8, 2HQ9, 2HQA, 2HQB, 2HQC, 2HQD, 2HQE, 2HQF, 2HQG, 2HQH, 2HQJ, 2HQK, 2HQL, 2HQM, 2HQQ, 2HQS, 2HQT, 2HQU, 2HQV, 2HQW, 2HQX, 2HQY, 2HQZ, 2HR0, 2HR1, 2HR2, 2HR3, 2HR5, 2HR6, 2HR7, 2HR8, 2HRA, 2HRB, 2HRC, 2HRD, 2HRE, 2HRG, 2HRH, 2HRI, 2HRK, 2HRL, 2HRM, 2HRO, 2HRP, 2HRQ, 2HRR, 2HRS, 2HRT, 2HRU, 2HRV, 2HRW, 2HRX, 2HRY, 2HRZ, 2HS0, 2HS1, 2HS2, 2HS3, 2HS4, 2HS5, 2HS6, 2HS8, 2HSA, 2HSB, 2HSD, 2HSE, 2HSG, 2HSH, 2HSI, 2HSJ, 2HSM, 2HSN, 2HSQ, 2HSW, 2HSZ, 2HT0, 2HT2, 2HT3, 2HT4, 2HT5, 2HT6, 2HT7, 2HT8, 2HT9, 2HTA, 2HTB, 2HTD, 2HTE, 2HTH, 2HTI, 2HTK, 2HTL, 2HTM, 2HTN, 2HTO, 2HTQ, 2HTR, 2HTS, 2HTT, 2HTU, 2HTV, 2HTW, 2HTX, 2HTY, 2HU0, 2HU1, 2HU2, 2HU3, 2HU4, 2HU5, 2HU6, 2HU7, 2HU8, 2HU9, 2HUB, 2HUC, 2HUE, 2HUF, 2HUH, 2HUI, 2HUJ, 2HUK, 2HUL, 2HUM, 2HUN, 2HUO, 2HUP, 2HUQ, 2HUR, 2HUT, 2HUU, 2HUV, 2HUW, 2HUX, 2HUZ, 2HV2, 2HV5, 2HV6, 2HV7, 2HV8, 2HV9, 2HVB, 2HVC, 2HVD, 2HVE, 2HVF, 2HVG, 2HVH, 2HVI, 2HVJ, 2HVK, 2HVL, 2HVM, 2HVN, 2HVO, 2HVP, 2HVQ, 2HVS, 2HVV, 2HVW, 2HVX, 2HW1, 2HW2, 2HW3, 2HW4, 2HW5, 2HW6, 2HW7, 2HW9, 2HWA, 2HWG, 2HWH, 2HWI, 2HWJ, 2HWK, 2HWL, 2HWM, 2HWN, 2HWO, 2HWP, 2HWQ, 2HWR, 2HWU, 2HWV, 2HWW, 2HWX, 2HWY, 2HWZ, 2HX0, 2HX1, 2HX2, 2HX3, 2HX4, 2HX5, 2HX7, 2HX8, 2HX9, 2HXA, 2HXB, 2HXC, 2HXD, 2HXG, 2HXI, 2HXK, 2HXL, 2HXM, 2HXO, 2HXP, 2HXQ, 2HXR, 2HXS, 2HXT, 2HXU, 2HXV, 2HXW, 2HXX, 2HXY, 2HXZ, 2HY0, 2HY1, 2HY3, 2HY5, 2HY6, 2HY7, 2HY8, 2HYB, 2HYD, 2HYE, 2HYF, 2HYG, 2HYJ, 2HYK, 2HYO, 2HYP, 2HYQ, 2HYR, 2HYS, 2HYT, 2HYU, 2HYV, 2HYW, 2HYX, 2HYY, 2HYZ, 2HZ0, 2HZ1, 2HZ2, 2HZ3, 2HZ4, 2HZ5, 2HZ6, 2HZ7, 2HZ9, 2HZA, 2HZB, 2HZC, 2HZE, 2HZF, 2HZG, 2HZH, 2HZI, 2HZK, 2HZL, 2HZM, 2HZN, 2HZP, 2HZQ, 2HZR, 2HZS, 2HZT, 2HZV, 2HZY, 2I00, 2I02, 2I03, 2I04, 2I05, 2I06, 2I07, 2I08, 2I0A, 2I0B, 2I0C, 2I0D, 2I0E, 2I0F, 2I0G, 2I0H, 2I0I, 2I0J, 2I0K, 2I0L, 2I0M, 2I0O, 2I0Q, 2I0R, 2I0S, 2I0T, 2I0U, 2I0V, 2I0W, 2I0X, 2I0Y, 2I0Z, 2I10, 2I13, 2I14, 2I15, 2I16, 2I17, 2I19, 2I1A, 2I1B, 2I1J, 2I1K, 2I1L, 2I1M, 2I1N, 2I1O, 2I1Q, 2I1R, 2I1S, 2I1U, 2I1V, 2I1W, 2I1X, 2I1Y, 2I20, 2I21, 2I22, 2I24, 2I25, 2I26, 2I27, 2I29, 2I2A, 2I2B, 2I2C, 2I2D, 2I2F, 2I2I, 2I2L, 2I2O, 2I2Q, 2I2R, 2I2S, 2I2W, 2I2X, 2I2Z, 2I30, 2I32, 2I33, 2I34, 2I35, 2I36, 2I37, 2I39, 2I3A, 2I3C, 2I3D, 2I3F, 2I3G, 2I3H, 2I3I, 2I3O, 2I3P, 2I3Q, 2I3R, 2I3S, 2I3T, 2I3U, 2I3V, 2I3W, 2I3Y, 2I3Z, 2I40, 2I42, 2I44, 2I45, 2I46, 2I47, 2I48, 2I49, 2I4A, 2I4B, 2I4C, 2I4D, 2I4E, 2I4G, 2I4H, 2I4I, 2I4J, 2I4L, 2I4M, 2I4N, 2I4O, 2I4P, 2I4Q, 2I4R, 2I4S, 2I4T, 2I4U, 2I4V, 2I4W, 2I4X, 2I4Z, 2I51, 2I52, 2I53, 2I54, 2I55, 2I56, 2I57, 2I58, 2I5A, 2I5B, 2I5C, 2I5D, 2I5E, 2I5F, 2I5G, 2I5H, 2I5I, 2I5J, 2I5K, 2I5L, 2I5M, 2I5N, 2I5P, 2I5Q, 2I5R, 2I5S, 2I5T, 2I5U, 2I5V, 2I5W, 2I5X, 2I5Y, 2I5Z, 2I60, 2I61, 2I62, 2I65, 2I66, 2I67, 2I69, 2I6A, 2I6B, 2I6D, 2I6E, 2I6F, 2I6G, 2I6H, 2I6I, 2I6J, 2I6K, 2I6L, 2I6M, 2I6O, 2I6P, 2I6Q, 2I6R, 2I6S, 2I6T, 2I6U, 2I6V, 2I6W, 2I6X, 2I6Y, 2I6Z, 2I71, 2I72, 2I74, 2I75, 2I76, 2I78, 2I79, 2I7A, 2I7B, 2I7C, 2I7D, 2I7F, 2I7G, 2I7H, 2I7N, 2I7O, 2I7P, 2I7Q, 2I7R, 2I7S, 2I7T, 2I7V, 2I7X, 2I80, 2I81, 2I87, 2I88, 2I89, 2I8A, 2I8B, 2I8C, 2I8D, 2I8E, 2I8T, 2I8U, 2I99, 2I9A, 2I9B, 2I9C, 2I9D, 2I9E, 2I9F, 2I9G, 2I9I, 2I9K, 2I9L, 2I9P, 2I9T, 2I9U, 2I9V, 2I9W, 2I9X, 2I9Z, 2IA0, 2IA1, 2IA2, 2IA4, 2IA5, 2IA6, 2IA7, 2IA8, 2IA9, 2IAA, 2IAB, 2IAD, 2IAE, 2IAF, 2IAG, 2IAH, 2IAI, 2IAJ, 2IAK, 2IAL, 2IAM, 2IAN, 2IAO, 2IAP, 2IAQ, 2IAR, 2IAS, 2IAT, 2IAU, 2IAV, 2IAW, 2IAX, 2IAY, 2IAZ, 2IB0, 2IB5, 2IB6, 2IB7, 2IB8, 2IB9, 2IBA, 2IBB, 2IBD, 2IBF, 2IBG, 2IBI, 2IBJ, 2IBK, 2IBL, 2IBM, 2IBN, 2IBO, 2IBP, 2IBS, 2IBT, 2IBU, 2IBW, 2IBX, 2IBY, 2IBZ, 2IC0, 2IC1, 2IC2, 2IC3, 2IC5, 2IC6, 2IC7, 2IC8, 2IC9, 2ICA, 2ICC, 2ICE, 2ICF, 2ICG, 2ICH, 2ICI, 2ICJ, 2ICK, 2ICP, 2ICQ, 2ICR, 2ICS, 2ICT, 2ICU, 2ICV, 2ICW, 2ICX, 2ICY, 2ID0, 2ID1, 2ID2, 2ID3, 2ID4, 2ID5, 2ID6, 2ID7, 2ID8, 2ID9, 2IDB, 2IDC, 2IDE, 2IDF, 2IDG, 2IDH, 2IDJ, 2IDK, 2IDL, 2IDM, 2IDO, 2IDQ, 2IDR, 2IDS, 2IDT, 2IDU, 2IDV, 2IDW, 2IDX, 2IDZ, 2IE0, 2IE1, 2IE2, 2IE3, 2IE4, 2IE6, 2IE7, 2IE8, 2IEA, 2IEB, 2IEC, 2IED, 2IEE, 2IEF, 2IEG, 2IEH, 2IEI, 2IEJ, 2IEK, 2IEL, 2IEN, 2IEO, 2IEP, 2IEQ, 2IER, 2IES, 2IEW, 2IEX, 2IEY, 2IEZ, 2IF0, 2IF2, 2IF4, 2IF5, 2IF6, 2IF7, 2IF8, 2IF9, 2IFA, 2IFB, 2IFC, 2IFD, 2IFF, 2IFG, 2IFQ, 2IFR, 2IFT, 2IFU, 2IFV, 2IFW, 2IFX, 2IFY, 2IG0, 2IG2, 2IG3, 2IG6, 2IG7, 2IG8, 2IG9, 2IGA, 2IGB, 2IGC, 2IGD, 2IGF, 2IGI, 2IGK, 2IGL, 2IGM, 2IGN, 2IGO, 2IGP, 2IGQ, 2IGS, 2IGT, 2IGV, 2IGW, 2IGX, 2IGY, 2IH1, 2IH2, 2IH3, 2IH4, 2IH5, 2IH8, 2IH9, 2IHB, 2IHC, 2IHD, 2IHE, 2IHF, 2IHJ, 2IHK, 2IHL, 2IHM, 2IHN, 2IHO, 2IHP, 2IHQ, 2IHR, 2IHS, 2IHT, 2IHU, 2IHV, 2IHW, 2IHY, 2IHZ, 2II0, 2II1, 2II2, 2II3, 2II4, 2II5, 2II6, 2II7, 2II8, 2II9, 2IIA, 2IIB, 2IIC, 2IID, 2IIE, 2IIF, 2IIH, 2III, 2IIK, 2IIM, 2IIP, 2IIQ, 2IIR, 2IIT, 2IIU, 2IIV, 2IIY, 2IIZ, 2IJ0, 2IJ2, 2IJ3, 2IJ4, 2IJ5, 2IJ7, 2IJ9, 2IJA, 2IJC, 2IJD, 2IJE, 2IJF, 2IJG, 2IJH, 2IJI, 2IJJ, 2IJK, 2IJL, 2IJM, 2IJN, 2IJO, 2IJQ, 2IJR, 2IJX, 2IJZ, 2IK0, 2IK1, 2IK2, 2IK4, 2IK6, 2IK7, 2IK8, 2IK9, 2IKB, 2IKC, 2IKF, 2IKG, 2IKH, 2IKI, 2IKJ, 2IKK, 2IKO, 2IKQ, 2IKS, 2IKU, 2IL1, 2IL2, 2IL3, 2IL4, 2IL5, 2ILA, 2ILI, 2ILK, 2ILL, 2ILM, 2ILN, 2ILP, 2ILR, 2ILT, 2ILU, 2ILV, 2ILY, 2ILZ, 2IM0, 2IM1, 2IM2, 2IM3, 2IM5, 2IM8, 2IM9, 2IMA, 2IMB, 2IMC, 2IMD, 2IME, 2IMF, 2IMG, 2IMH, 2IMI, 2IMJ, 2IMK, 2IML, 2IMM, 2IMN, 2IMO, 2IMP, 2IMQ, 2IMR, 2IMS, 2IMT, 2IMW, 2IMZ, 2IN0, 2IN3, 2IN4, 2IN5, 2IN6, 2IN8, 2IN9, 2INB, 2INC, 2IND, 2INE, 2INF, 2ING, 2INN, 2INP, 2INR, 2INS, 2INT, 2INU, 2INV, 2INW, 2INX, 2INY, 2INZ, 2IO0, 2IO1, 2IO2, 2IO3, 2IO4, 2IO5, 2IO6, 2IO7, 2IO8, 2IO9, 2IOA, 2IOB, 2IOC, 2IOD, 2IOF, 2IOG, 2IOH, 2IOI, 2IOJ, 2IOK, 2IOL, 2IOM, 2ION, 2IOO, 2IOP, 2IOQ, 2IOR, 2IOS, 2IOT, 2IOU, 2IOV, 2IOY, 2IP1, 2IP2, 2IP4, 2IP6, 2IPB, 2IPC, 2IPF, 2IPG, 2IPH, 2IPI, 2IPJ, 2IPK, 2IPL, 2IPM, 2IPN, 2IPO, 2IPP, 2IPQ, 2IPR, 2IPS, 2IPT, 2IPU, 2IPW, 2IPX, 2IPZ, 2IQ0, 2IQ1, 2IQ5, 2IQ6, 2IQ7, 2IQ9, 2IQA, 2IQC, 2IQD, 2IQF, 2IQG, 2IQH, 2IQI, 2IQJ, 2IQQ, 2IQT, 2IQX, 2IQY, 2IRF, 2IRM, 2IRP, 2IRT, 2IRU, 2IRV, 2IRW, 2IRX, 2IRY, 2IRZ, 2IS0, 2IS1, 2IS2, 2IS3, 2IS4, 2IS5, 2IS6, 2IS7, 2IS8, 2IS9, 2ISA, 2ISB, 2ISC, 2ISD, 2ISE, 2ISF, 2ISG, 2ISH, 2ISI, 2ISJ, 2ISK, 2ISL, 2ISM, 2ISN, 2ISO, 2ISP, 2ISQ, 2ISS, 2IST, 2ISV, 2ISW, 2ISY, 2ISZ, 2IT0, 2IT1, 2IT2, 2IT3, 2IT4, 2IT5, 2IT6, 2IT9, 2ITB, 2ITC, 2ITD, 2ITE, 2ITF, 2ITG, 2ITJ, 2ITK, 2ITL, 2ITM, 2ITN, 2ITO, 2ITP, 2ITQ, 2ITT, 2ITU, 2ITV, 2ITW, 2ITX, 2ITY, 2ITZ, 2IU0, 2IU1, 2IU2, 2IU3, 2IU4, 2IU5, 2IU6, 2IU7, 2IU8, 2IU9, 2IUA, 2IUB, 2IUC, 2IUF, 2IUG, 2IUH, 2IUI, 2IUJ, 2IUK, 2IUL, 2IUM, 2IUN, 2IUO, 2IUP, 2IUQ, 2IUR, 2IUS, 2IUT, 2IUU, 2IUV, 2IUW, 2IUX, 2IUY, 2IUZ, 2IV0, 2IV1, 2IV2, 2IV3, 2IV7, 2IV8, 2IV9, 2IVB, 2IVD, 2IVE, 2IVF, 2IVG, 2IVH, 2IVI, 2IVJ, 2IVK, 2IVM, 2IVN, 2IVO, 2IVP, 2IVQ, 2IVS, 2IVT, 2IVU, 2IVV, 2IVX, 2IVY, 2IVZ, 2IW0, 2IW1, 2IW2, 2IW3, 2IW4, 2IW5, 2IW6, 2IW8, 2IW9, 2IWA, 2IWB, 2IWC, 2IWD, 2IWE, 2IWF, 2IWG, 2IWH, 2IWI, 2IWK, 2IWL, 2IWM, 2IWN, 2IWO, 2IWP, 2IWQ, 2IWR, 2IWS, 2IWT, 2IWU, 2IWW, 2IWX, 2IWY, 2IWZ, 2IX0, 2IX2, 2IX3, 2IX4, 2IX5, 2IX6, 2IX7, 2IX9, 2IXA, 2IXB, 2IXC, 2IXD, 2IXE, 2IXF, 2IXG, 2IXH, 2IXI, 2IXJ, 2IXK, 2IXL, 2IXM, 2IXN, 2IXO, 2IXP, 2IXR, 2IXS, 2IXT, 2IXU, 2IXV, 2IY0, 2IY1, 2IY2, 2IY4, 2IY6, 2IY7, 2IY8, 2IY9, 2IYA, 2IYB, 2IYC, 2IYD, 2IYE, 2IYF, 2IYG, 2IYI, 2IYJ, 2IYK, 2IYL, 2IYN, 2IYO, 2IYP, 2IYQ, 2IYR, 2IYS, 2IYT, 2IYU, 2IYV, 2IYW, 2IYX, 2IYY, 2IYZ, 2IZ0, 2IZ1, 2IZ5, 2IZ6, 2IZ7, 2IZA, 2IZB, 2IZC, 2IZD, 2IZE, 2IZF, 2IZG, 2IZH, 2IZI, 2IZJ, 2IZK, 2IZL, 2IZO, 2IZP, 2IZR, 2IZS, 2IZT, 2IZU, 2IZV, 2IZX, 2IZY, 2IZZ, 2J04, 2J05, 2J06, 2J07, 2J08, 2J09, 2J0A, 2J0B, 2J0D, 2J0E, 2J0F, 2J0G, 2J0H, 2J0I, 2J0J, 2J0K, 2J0L, 2J0M, 2J0N, 2J0O, 2J0P, 2J0R, 2J0T, 2J0U, 2J0V, 2J0W, 2J0X, 2J0Y, 2J12, 2J13, 2J14, 2J16, 2J17, 2J18, 2J19, 2J1A, 2J1D, 2J1E, 2J1G, 2J1K, 2J1L, 2J1M, 2J1N, 2J1O, 2J1P, 2J1Q, 2J1R, 2J1S, 2J1T, 2J1U, 2J1V, 2J1W, 2J1X, 2J1Y, 2J1Z, 2J20, 2J21, 2J22, 2J23, 2J24, 2J25, 2J27, 2J2C, 2J2F, 2J2I, 2J2J, 2J2M, 2J2P, 2J2U, 2J2Z, 2J30, 2J31, 2J32, 2J33, 2J34, 2J38, 2J3D, 2J3E, 2J3F, 2J3G, 2J3H, 2J3I, 2J3J, 2J3K, 2J3L, 2J3M, 2J3N, 2J3O, 2J3P, 2J3Q, 2J3R, 2J3S, 2J3T, 2J3U, 2J3V, 2J3W, 2J3X, 2J3Z, 2J40, 2J41, 2J42, 2J43, 2J44, 2J45, 2J46, 2J47, 2J49, 2J4A, 2J4B, 2J4C, 2J4D, 2J4E, 2J4F, 2J4G, 2J4H, 2J4I, 2J4J, 2J4K, 2J4L, 2J4O, 2J4Q, 2J4R, 2J4S, 2J4T, 2J4U, 2J4W, 2J4X, 2J4Y, 2J4Z, 2J50, 2J51, 2J55, 2J56, 2J57, 2J58, 2J59, 2J5A, 2J5B, 2J5C, 2J5E, 2J5F, 2J5G, 2J5I, 2J5K, 2J5L, 2J5M, 2J5N, 2J5Q, 2J5R, 2J5S, 2J5T, 2J5U, 2J5V, 2J5W, 2J5X, 2J5Y, 2J5Z, 2J60, 2J61, 2J62, 2J63, 2J64, 2J65, 2J66, 2J67, 2J68, 2J69, 2J6A, 2J6B, 2J6C, 2J6E, 2J6F, 2J6G, 2J6H, 2J6I, 2J6J, 2J6K, 2J6L, 2J6M, 2J6O, 2J6P, 2J6R, 2J6S, 2J6T, 2J6U, 2J6V, 2J6W, 2J6X, 2J6Y, 2J6Z, 2J70, 2J71, 2J72, 2J73, 2J74, 2J75, 2J77, 2J78, 2J79, 2J7A, 2J7B, 2J7C, 2J7D, 2J7E, 2J7F, 2J7G, 2J7H, 2J7I, 2J7J, 2J7K, 2J7L, 2J7M, 2J7N, 2J7O, 2J7P, 2J7Q, 2J7T, 2J7U, 2J7V, 2J7W, 2J7X, 2J7Y, 2J7Z, 2J80, 2J82, 2J83, 2J85, 2J86, 2J87, 2J88, 2J89, 2J8A, 2J8B, 2J8C, 2J8D, 2J8F, 2J8G, 2J8H, 2J8I, 2J8K, 2J8M, 2J8N, 2J8O, 2J8Q, 2J8R, 2J8S, 2J8T, 2J8U, 2J8W, 2J8X, 2J8Y, 2J8Z, 2J90, 2J91, 2J92, 2J94, 2J95, 2J96, 2J97, 2J98, 2J9A, 2J9B, 2J9C, 2J9D, 2J9E, 2J9F, 2J9G, 2J9H, 2J9J, 2J9K, 2J9L, 2J9M, 2J9N, 2J9O, 2J9P, 2J9Q, 2J9R, 2J9T, 2J9U, 2J9V, 2J9W, 2J9X, 2J9Y, 2J9Z, 2JA1, 2JA2, 2JA3, 2JA4, 2JA9, 2JAA, 2JAB, 2JAC, 2JAD, 2JAE, 2JAF, 2JAG, 2JAH, 2JAI, 2JAJ, 2JAK, 2JAL, 2JAM, 2JAN, 2JAO, 2JAP, 2JAQ, 2JAR, 2JAS, 2JAT, 2JAU, 2JAV, 2JAW, 2JAX, 2JAY, 2JAZ, 2JB0, 2JB1, 2JB2, 2JB3, 2JB4, 2JB5, 2JB6, 2JB7, 2JB8, 2JB9, 2JBA, 2JBF, 2JBG, 2JBH, 2JBJ, 2JBK, 2JBL, 2JBM, 2JBO, 2JBP, 2JBR, 2JBS, 2JBT, 2JBU, 2JBV, 2JBW, 2JBX, 2JBY, 2JBZ, 2JC0, 2JC1, 2JC2, 2JC3, 2JC4, 2JC5, 2JC6, 2JC7, 2JC9, 2JCA, 2JCB, 2JCC, 2JCD, 2JCG, 2JCH, 2JCJ, 2JCK, 2JCL, 2JCM, 2JCN, 2JCO, 2JCP, 2JCQ, 2JCR, 2JCS, 2JCV, 2JCW, 2JCX, 2JCY, 2JD0, 2JD1, 2JD2, 2JD3, 2JD4, 2JD5, 2JD6, 2JD7, 2JD8, 2JD9, 2JDA, 2JDC, 2JDD, 2JDF, 2JDG, 2JDH, 2JDI, 2JDJ, 2JDK, 2JDL, 2JDM, 2JDN, 2JDO, 2JDP, 2JDQ, 2JDR, 2JDS, 2JDT, 2JDU, 2JDV, 2JDW, 2JDX, 2JDY, 2JDZ, 2JE0, 2JE1, 2JE2, 2JE3, 2JE4, 2JE5, 2JE6, 2JE7, 2JE8, 2JE9, 2JEB, 2JEC, 2JED, 2JEE, 2JEF, 2JEG, 2JEI, 2JEJ, 2JEK, 2JEL, 2JEM, 2JEN, 2JEO, 2JEP, 2JEQ, 2JER, 2JES, 2JET, 2JEU, 2JEV, 2JEW, 2JEX, 2JEY, 2JEZ, 2JF0, 2JF1, 2JF2, 2JF3, 2JF4, 2JF5, 2JF6, 2JF7, 2JF9, 2JFA, 2JFB, 2JFC, 2JFD, 2JFE, 2JFF, 2JFG, 2JFH, 2JFK, 2JFL, 2JFM, 2JFN, 2JFO, 2JFP, 2JFQ, 2JFR, 2JFS, 2JFT, 2JFU, 2JFV, 2JFW, 2JFX, 2JFY, 2JFZ, 2JG0, 2JG1, 2JG2, 2JG3, 2JG4, 2JG5, 2JG6, 2JG7, 2JG8, 2JG9, 2JGA, 2JGB, 2JGC, 2JGD, 2JGE, 2JGF, 2JGI, 2JGJ, 2JGK, 2JGL, 2JGM, 2JGN, 2JGO, 2JGP, 2JGQ, 2JGR, 2JGS, 2JGT, 2JGU, 2JGV, 2JGY, 2JGZ, 2JH0, 2JH1, 2JH2, 2JH3, 2JH5, 2JH6, 2JH7, 2JH8, 2JH9, 2JHA, 2JHC, 2JHD, 2JHE, 2JHF, 2JHG, 2JHH, 2JHI, 2JHJ, 2JHK, 2JHL, 2JHM, 2JHN, 2JHO, 2JHP, 2JHQ, 2JHR, 2JHS, 2JHT, 2JHU, 2JHV, 2JHW, 2JHX, 2JHY, 2JHZ, 2JI0, 2JI1, 2JI2, 2JI3, 2JI4, 2JI5, 2JI6, 2JI7, 2JI8, 2JI9, 2JIA, 2JIB, 2JIC, 2JID, 2JIE, 2JIF, 2JIG, 2JIH, 2JII, 2JIJ, 2JIK, 2JIL, 2JIM, 2JIN, 2JIO, 2JIP, 2JIQ, 2JIR, 2JIS, 2JIT, 2JIU, 2JIV, 2JIW, 2JIX, 2JIY, 2JIZ, 2JJ0, 2JJ1, 2JJ2, 2JJ3, 2JJ4, 2JJ6, 2JJ7, 2JJ8, 2JJ9, 2JJB, 2JJC, 2JJD, 2JJE, 2JJF, 2JJG, 2JJH, 2JJI, 2JJJ, 2JJK, 2JJL, 2JJM, 2JJN, 2JJO, 2JJP, 2JJQ, 2JJR, 2JJS, 2JJT, 2JJU, 2JJV, 2JJW, 2JJX, 2JJY, 2JJZ, 2JK0, 2JK1, 2JK2, 2JK3, 2JK4, 2JK5, 2JK6, 2JK7, 2JK8, 2JK9, 2JKA, 2JKB, 2JKC, 2JKD, 2JKE, 2JKF, 2JKG, 2JKH, 2JKI, 2JKJ, 2JKK, 2JKL, 2JKM, 2JKN, 2JKO, 2JKP, 2JKQ, 2JKR, 2JKS, 2JKT, 2JKU, 2JKV, 2JKW, 2JKX, 2JKY, 2JKZ, 2JL1, 2JL4, 2JL9, 2JLA, 2JLB, 2JLC, 2JLD, 2JLE, 2JLF, 2JLG, 2JLH, 2JLI, 2JLJ, 2JLL, 2JLM, 2JLN, 2JLP, 2JLQ, 2JLR, 2JLS, 2JXR, 2KAI, 2KAU, 2KCE, 2KFN, 2KFZ, 2KI5, 2KIN, 2KMB, 2KNT, 2KTQ, 2KZZ, 2L78, 2LAL, 2LAO, 2LBD, 2LBP, 2LDB, 2LDX, 2LGS, 2LH1, 2LH2, 2LH3, 2LH5, 2LH6, 2LH7, 2LHB, 2LHM, 2LIG, 2LIP, 2LIS, 2LIV, 2LJR, 2LKF, 2LPR, 2LTN, 2LVE, 2LYM, 2LYN, 2LYO, 2LYZ, 2LZ2, 2LZH, 2LZM, 2LZT, 2MAD, 2MAN, 2MAS, 2MAT, 2MBR, 2MBW, 2MCG, 2MCM, 2MCP, 2MEA, 2MEB, 2MEC, 2MED, 2MEE, 2MEF, 2MEG, 2MEH, 2MEI, 2MGA, 2MGB, 2MGC, 2MGD, 2MGE, 2MGF, 2MGG, 2MGH, 2MGI, 2MGJ, 2MGK, 2MGL, 2MGM, 2MHA, 2MHB, 2MHR, 2MIB, 2MIN, 2MIP, 2MJP, 2MLL, 2MLT, 2MNR, 2MPA, 2MPR, 2MS2, 2MSB, 2MSI, 2MSJ, 2MSP, 2MTA, 2MUC, 2MYA, 2MYB, 2MYC, 2MYD, 2MYE, 2MYS, 2NAC, 2NAD, 2NAP, 2NCD, 2NCK, 2NG1, 2NGR, 2NIP, 2NL8, 2NL9, 2NLA, 2NLB, 2NLC, 2NLD, 2NLE, 2NLF, 2NLG, 2NLH, 2NLI, 2NLJ, 2NLK, 2NLL, 2NLM, 2NLO, 2NLP, 2NLQ, 2NLR, 2NLS, 2NLV, 2NLX, 2NLY, 2NLZ, 2NM0, 2NM1, 2NM2, 2NM3, 2NML, 2NMM, 2NMN, 2NMO, 2NMP, 2NMR, 2NMS, 2NMT, 2NMU, 2NMV, 2NMX, 2NMY, 2NMZ, 2NN1, 2NN2, 2NN3, 2NN4, 2NN5, 2NN6, 2NN7, 2NN8, 2NNA, 2NNB, 2NNC, 2NND, 2NNE, 2NNF, 2NNG, 2NNH, 2NNI, 2NNJ, 2NNK, 2NNL, 2NNN, 2NNO, 2NNP, 2NNQ, 2NNR, 2NNS, 2NNU, 2NNV, 2NNW, 2NNX, 2NNY, 2NO0, 2NO1, 2NO2, 2NO3, 2NO4, 2NO5, 2NO6, 2NO7, 2NO9, 2NOA, 2NOB, 2NOD, 2NOE, 2NOF, 2NOG, 2NOH, 2NOI, 2NOJ, 2NOL, 2NOM, 2NOO, 2NOP, 2NOS, 2NOT, 2NOV, 2NOW, 2NOX, 2NOY, 2NOZ, 2NP0, 2NP1, 2NP2, 2NP3, 2NP5, 2NP6, 2NP7, 2NP8, 2NP9, 2NPA, 2NPC, 2NPD, 2NPE, 2NPF, 2NPG, 2NPH, 2NPI, 2NPJ, 2NPK, 2NPM, 2NPN, 2NPO, 2NPP, 2NPQ, 2NPS, 2NPT, 2NPX, 2NQ2, 2NQ3, 2NQ5, 2NQ6, 2NQ7, 2NQ8, 2NQ9, 2NQA, 2NQB, 2NQC, 2NQD, 2NQG, 2NQH, 2NQI, 2NQJ, 2NQK, 2NQL, 2NQM, 2NQN, 2NQO, 2NQQ, 2NQR, 2NQS, 2NQT, 2NQU, 2NQV, 2NQW, 2NQX, 2NQY, 2NQZ, 2NR4, 2NR5, 2NR6, 2NR7, 2NR9, 2NRA, 2NRB, 2NRC, 2NRD, 2NRF, 2NRH, 2NRJ, 2NRK, 2NRL, 2NRM, 2NRN, 2NRO, 2NRP, 2NRQ, 2NRR, 2NRS, 2NRT, 2NRU, 2NRV, 2NRW, 2NRX, 2NRY, 2NRZ, 2NS0, 2NS1, 2NS2, 2NS6, 2NS7, 2NS8, 2NS9, 2NSA, 2NSB, 2NSC, 2NSD, 2NSE, 2NSF, 2NSG, 2NSH, 2NSI, 2NSJ, 2NSL, 2NSM, 2NSN, 2NSO, 2NSP, 2NSQ, 2NSR, 2NSS, 2NST, 2NSX, 2NSY, 2NSZ, 2NT0, 2NT1, 2NT2, 2NT3, 2NT4, 2NT6, 2NT7, 2NT8, 2NT9, 2NTA, 2NTB, 2NTC, 2NTD, 2NTE, 2NTF, 2NTG, 2NTH, 2NTI, 2NTJ, 2NTK, 2NTL, 2NTM, 2NTN, 2NTO, 2NTP, 2NTQ, 2NTR, 2NTS, 2NTT, 2NTU, 2NTV, 2NTW, 2NTX, 2NTY, 2NTZ, 2NU0, 2NU1, 2NU2, 2NU3, 2NU4, 2NU5, 2NU6, 2NU7, 2NU8, 2NU9, 2NUA, 2NUB, 2NUC, 2NUD, 2NUH, 2NUI, 2NUJ, 2NUK, 2NUL, 2NUM, 2NUN, 2NUO, 2NUP, 2NUT, 2NUU, 2NUV, 2NUW, 2NUX, 2NUY, 2NUZ, 2NV0, 2NV1, 2NV2, 2NV4, 2NV5, 2NV6, 2NV7, 2NV9, 2NVA, 2NVB, 2NVC, 2NVD, 2NVE, 2NVF, 2NVG, 2NVH, 2NVK, 2NVL, 2NVM, 2NVN, 2NVO, 2NVP, 2NVU, 2NVV, 2NVW, 2NVY, 2NW0, 2NW2, 2NW3, 2NW4, 2NW6, 2NW7, 2NW8, 2NW9, 2NWA, 2NWB, 2NWC, 2NWD, 2NWF, 2NWG, 2NWH, 2NWI, 2NWJ, 2NWL, 2NWN, 2NWO, 2NWP, 2NWQ, 2NWR, 2NWS, 2NWU, 2NWV, 2NWW, 2NWX, 2NWY, 2NWZ, 2NX0, 2NX1, 2NX2, 2NX3, 2NX4, 2NX5, 2NX8, 2NX9, 2NXA, 2NXB, 2NXC, 2NXD, 2NXE, 2NXF, 2NXG, 2NXH, 2NXI, 2NXJ, 2NXL, 2NXM, 2NXN, 2NXO, 2NXP, 2NXQ, 2NXR, 2NXS, 2NXT, 2NXV, 2NXW, 2NXX, 2NXY, 2NXZ, 2NY0, 2NY1, 2NY2, 2NY3, 2NY4, 2NY5, 2NY6, 2NY7, 2NYA, 2NYB, 2NYC, 2NYD, 2NYE, 2NYF, 2NYG, 2NYH, 2NYI, 2NYJ, 2NYK, 2NYL, 2NYM, 2NYN, 2NYP, 2NYQ, 2NYR, 2NYS, 2NYT, 2NYU, 2NYV, 2NYX, 2NYY, 2NYZ, 2NZ0, 2NZ1, 2NZ2, 2NZ5, 2NZ6, 2NZ7, 2NZ8, 2NZ9, 2NZA, 2NZC, 2NZD, 2NZE, 2NZF, 2NZH, 2NZI, 2NZJ, 2NZL, 2NZM, 2NZO, 2NZT, 2NZU, 2NZV, 2NZW, 2NZX, 2NZY, 2O02, 2O03, 2O04, 2O05, 2O06, 2O07, 2O08, 2O09, 2O0A, 2O0B, 2O0C, 2O0D, 2O0E, 2O0G, 2O0H, 2O0I, 2O0J, 2O0K, 2O0L, 2O0M, 2O0O, 2O0P, 2O0Q, 2O0R, 2O0T, 2O0U, 2O0V, 2O0W, 2O0X, 2O0Y, 2O0Z, 2O11, 2O12, 2O14, 2O15, 2O16, 2O17, 2O18, 2O19, 2O1A, 2O1B, 2O1C, 2O1D, 2O1E, 2O1F, 2O1G, 2O1H, 2O1J, 2O1K, 2O1L, 2O1M, 2O1N, 2O1O, 2O1P, 2O1Q, 2O1S, 2O1T, 2O1U, 2O1V, 2O1W, 2O1X, 2O1Z, 2O20, 2O23, 2O24, 2O25, 2O26, 2O27, 2O28, 2O29, 2O2A, 2O2B, 2O2C, 2O2D, 2O2E, 2O2G, 2O2H, 2O2I, 2O2J, 2O2K, 2O2L, 2O2P, 2O2Q, 2O2R, 2O2S, 2O2T, 2O2U, 2O2V, 2O2X, 2O2Y, 2O2Z, 2O30, 2O31, 2O34, 2O35, 2O36, 2O37, 2O38, 2O39, 2O3A, 2O3B, 2O3C, 2O3E, 2O3F, 2O3G, 2O3H, 2O3I, 2O3J, 2O3K, 2O3L, 2O3O, 2O3P, 2O3Q, 2O3R, 2O3S, 2O3T, 2O3U, 2O3Z, 2O40, 2O42, 2O48, 2O49, 2O4A, 2O4C, 2O4D, 2O4F, 2O4G, 2O4H, 2O4I, 2O4J, 2O4K, 2O4L, 2O4M, 2O4N, 2O4P, 2O4Q, 2O4R, 2O4S, 2O4T, 2O4U, 2O4V, 2O4W, 2O4X, 2O4Z, 2O50, 2O51, 2O52, 2O53, 2O54, 2O55, 2O56, 2O57, 2O58, 2O59, 2O5A, 2O5B, 2O5C, 2O5D, 2O5E, 2O5F, 2O5G, 2O5H, 2O5K, 2O5L, 2O5M, 2O5N, 2O5O, 2O5P, 2O5Q, 2O5R, 2O5S, 2O5T, 2O5U, 2O5V, 2O5W, 2O5X, 2O5Y, 2O5Z, 2O60, 2O61, 2O62, 2O63, 2O64, 2O65, 2O66, 2O67, 2O68, 2O69, 2O6A, 2O6B, 2O6C, 2O6D, 2O6E, 2O6F, 2O6G, 2O6H, 2O6I, 2O6K, 2O6L, 2O6M, 2O6N, 2O6P, 2O6Q, 2O6R, 2O6S, 2O6T, 2O6U, 2O6V, 2O6W, 2O6X, 2O6Y, 2O70, 2O71, 2O72, 2O73, 2O74, 2O78, 2O79, 2O7A, 2O7B, 2O7C, 2O7D, 2O7E, 2O7F, 2O7G, 2O7H, 2O7I, 2O7K, 2O7L, 2O7M, 2O7N, 2O7O, 2O7P, 2O7Q, 2O7R, 2O7S, 2O7T, 2O7U, 2O7V, 2O84, 2O85, 2O86, 2O87, 2O88, 2O89, 2O8A, 2O8B, 2O8C, 2O8D, 2O8E, 2O8F, 2O8G, 2O8H, 2O8I, 2O8J, 2O8L, 2O8M, 2O8N, 2O8O, 2O8P, 2O8Q, 2O8R, 2O8S, 2O8T, 2O8U, 2O8V, 2O8W, 2O8X, 2O8Y, 2O90, 2O92, 2O93, 2O94, 2O95, 2O96, 2O97, 2O98, 2O99, 2O9A, 2O9B, 2O9C, 2O9D, 2O9E, 2O9F, 2O9G, 2O9I, 2O9J, 2O9K, 2O9O, 2O9P, 2O9Q, 2O9R, 2O9S, 2O9T, 2O9U, 2O9V, 2O9X, 2O9Z, 2OA0, 2OA1, 2OA2, 2OA5, 2OA6, 2OA7, 2OA8, 2OA9, 2OAA, 2OAC, 2OAD, 2OAE, 2OAF, 2OAG, 2OAH, 2OAI, 2OAJ, 2OAL, 2OAM, 2OAN, 2OAP, 2OAQ, 2OAR, 2OAS, 2OAT, 2OAU, 2OAW, 2OAX, 2OAY, 2OAZ, 2OB0, 2OB1, 2OB2, 2OB3, 2OB4, 2OB5, 2OB9, 2OBA, 2OBB, 2OBC, 2OBD, 2OBE, 2OBF, 2OBG, 2OBH, 2OBI, 2OBJ, 2OBK, 2OBL, 2OBM, 2OBN, 2OBO, 2OBP, 2OBQ, 2OBR, 2OBS, 2OBT, 2OBV, 2OBX, 2OBY, 2OBZ, 2OC0, 2OC1, 2OC2, 2OC3, 2OC4, 2OC5, 2OC6, 2OC7, 2OC8, 2OC9, 2OCA, 2OCB, 2OCC, 2OCD, 2OCE, 2OCF, 2OCG, 2OCH, 2OCI, 2OCJ, 2OCK, 2OCL, 2OCP, 2OCS, 2OCT, 2OCU, 2OCV, 2OCX, 2OCY, 2OCZ, 2OD0, 2OD2, 2OD3, 2OD4, 2OD5, 2OD6, 2OD7, 2OD8, 2OD9, 2ODA, 2ODB, 2ODE, 2ODF, 2ODH, 2ODI, 2ODJ, 2ODK, 2ODL, 2ODM, 2ODN, 2ODO, 2ODP, 2ODQ, 2ODR, 2ODT, 2ODU, 2ODV, 2ODW, 2ODY, 2OE0, 2OE1, 2OE2, 2OE3, 2OE4, 2OE7, 2OE9, 2OEA, 2OEB, 2OEC, 2OEE, 2OEF, 2OEG, 2OEI, 2OEJ, 2OEK, 2OEL, 2OEM, 2OEN, 2OEO, 2OEP, 2OEQ, 2OER, 2OES, 2OEV, 2OEW, 2OEX, 2OEZ, 2OF0, 2OF1, 2OF2, 2OF3, 2OF4, 2OF5, 2OF7, 2OF8, 2OF9, 2OFA, 2OFB, 2OFC, 2OFD, 2OFE, 2OFF, 2OFI, 2OFJ, 2OFK, 2OFM, 2OFO, 2OFP, 2OFR, 2OFS, 2OFU, 2OFV, 2OFW, 2OFX, 2OFY, 2OFZ, 2OG0, 2OG1, 2OG2, 2OG3, 2OG4, 2OG5, 2OG6, 2OG7, 2OG8, 2OG9, 2OGA, 2OGB, 2OGD, 2OGE, 2OGF, 2OGG, 2OGI, 2OGJ, 2OGK, 2OGQ, 2OGR, 2OGS, 2OGT, 2OGU, 2OGV, 2OGW, 2OGX, 2OGY, 2OGZ, 2OH0, 2OH1, 2OH2, 2OH3, 2OH4, 2OH5, 2OH6, 2OH7, 2OH8, 2OH9, 2OHA, 2OHB, 2OHC, 2OHD, 2OHE, 2OHF, 2OHG, 2OHH, 2OHI, 2OHJ, 2OHK, 2OHL, 2OHM, 2OHN, 2OHO, 2OHP, 2OHQ, 2OHR, 2OHS, 2OHT, 2OHU, 2OHV, 2OHW, 2OHX, 2OHY, 2OI0, 2OI2, 2OI4, 2OI5, 2OI6, 2OI7, 2OI8, 2OI9, 2OIB, 2OIC, 2OID, 2OIE, 2OIF, 2OIG, 2OIK, 2OIL, 2OIN, 2OIP, 2OIQ, 2OIT, 2OIV, 2OIW, 2OIX, 2OIZ, 2OJ1, 2OJ4, 2OJ5, 2OJ6, 2OJ9, 2OJE, 2OJF, 2OJG, 2OJH, 2OJI, 2OJJ, 2OJK, 2OJL, 2OJP, 2OJQ, 2OJR, 2OJT, 2OJU, 2OJV, 2OJW, 2OJX, 2OJY, 2OJZ, 2OK0, 2OK1, 2OK2, 2OK3, 2OK4, 2OK5, 2OK6, 2OK7, 2OK8, 2OK9, 2OKA, 2OKB, 2OKC, 2OKD, 2OKE, 2OKF, 2OKG, 2OKH, 2OKI, 2OKJ, 2OKK, 2OKL, 2OKM, 2OKN, 2OKO, 2OKQ, 2OKR, 2OKS, 2OKT, 2OKU, 2OKV, 2OKW, 2OKX, 2OKY, 2OKZ, 2OL0, 2OL1, 2OL2, 2OL3, 2OL4, 2OL5, 2OL6, 2OL7, 2OL8, 2OL9, 2OLA, 2OLB, 2OLC, 2OLD, 2OLE, 2OLG, 2OLH, 2OLI, 2OLJ, 2OLK, 2OLM, 2OLN, 2OLO, 2OLP, 2OLQ, 2OLR, 2OLS, 2OLT, 2OLU, 2OLV, 2OLW, 2OLX, 2OLY, 2OLZ, 2OM0, 2OM1, 2OM2, 2OM5, 2OM6, 2OM9, 2OMA, 2OMB, 2OMD, 2OME, 2OMF, 2OMG, 2OMH, 2OMI, 2OMK, 2OML, 2OMM, 2OMN, 2OMO, 2OMP, 2OMQ, 2OMT, 2OMU, 2OMV, 2OMW, 2OMX, 2OMY, 2OMZ, 2ON3, 2ON5, 2ON6, 2ON7, 2ON8, 2ON9, 2ONA, 2ONB, 2ONC, 2OND, 2ONE, 2ONF, 2ONG, 2ONH, 2ONI, 2ONJ, 2ONK, 2ONL, 2ONM, 2ONN, 2ONO, 2ONP, 2ONQ, 2ONR, 2ONS, 2ONT, 2ONU, 2ONV, 2ONW, 2ONX, 2ONY, 2ONZ, 2OO0, 2OO1, 2OO2, 2OO3, 2OO4, 2OO5, 2OO6, 2OO7, 2OO8, 2OO9, 2OOA, 2OOB, 2OOC, 2OOD, 2OOE, 2OOF, 2OOG, 2OOH, 2OOI, 2OOJ, 2OOK, 2OOL, 2OOQ, 2OOR, 2OOS, 2OOT, 2OOV, 2OOW, 2OOX, 2OOY, 2OOZ, 2OP0, 2OP1, 2OP2, 2OP3, 2OP4, 2OP5, 2OP6, 2OP8, 2OP9, 2OPA, 2OPB, 2OPC, 2OPD, 2OPE, 2OPF, 2OPG, 2OPH, 2OPI, 2OPJ, 2OPK, 2OPL, 2OPM, 2OPN, 2OPO, 2OPP, 2OPQ, 2OPR, 2OPS, 2OPT, 2OPW, 2OPX, 2OPY, 2OPZ, 2OQ0, 2OQ1, 2OQ2, 2OQ4, 2OQ5, 2OQ6, 2OQ7, 2OQA, 2OQB, 2OQC, 2OQD, 2OQE, 2OQF, 2OQG, 2OQH, 2OQI, 2OQJ, 2OQK, 2OQL, 2OQM, 2OQN, 2OQO, 2OQQ, 2OQR, 2OQT, 2OQU, 2OQV, 2OQW, 2OQX, 2OQY, 2OQZ, 2OR0, 2OR1, 2OR2, 2OR3, 2OR4, 2OR7, 2OR8, 2OR9, 2ORA, 2ORB, 2ORD, 2ORE, 2ORF, 2ORG, 2ORH, 2ORI, 2ORJ, 2ORK, 2ORM, 2ORO, 2ORP, 2ORQ, 2ORR, 2ORS, 2ORT, 2ORV, 2ORW, 2ORX, 2ORY, 2ORZ, 2OS0, 2OS1, 2OS2, 2OS3, 2OS5, 2OS7, 2OS8, 2OS9, 2OSA, 2OSB, 2OSC, 2OSD, 2OSE, 2OSF, 2OSH, 2OSL, 2OSM, 2OSN, 2OSO, 2OSS, 2OST, 2OSU, 2OSV, 2OSW, 2OSX, 2OSY, 2OSZ, 2OT0, 2OT1, 2OT3, 2OT4, 2OT5, 2OT7, 2OT8, 2OT9, 2OTA, 2OTB, 2OTC, 2OTD, 2OTE, 2OTF, 2OTG, 2OTH, 2OTM, 2OTN, 2OTO, 2OTP, 2OTT, 2OTU, 2OTV, 2OTW, 2OTX, 2OTY, 2OTZ, 2OU0, 2OU1, 2OU2, 2OU3, 2OU4, 2OU5, 2OU6, 2OU7, 2OU8, 2OU9, 2OUA, 2OUB, 2OUC, 2OUD, 2OUG, 2OUH, 2OUI, 2OUJ, 2OUK, 2OUL, 2OUM, 2OUN, 2OUO, 2OUP, 2OUQ, 2OUR, 2OUS, 2OUU, 2OUV, 2OUW, 2OUX, 2OUY, 2OUZ, 2OV0, 2OV1, 2OV2, 2OV3, 2OV4, 2OV5, 2OV7, 2OV8, 2OV9, 2OVA, 2OVB, 2OVC, 2OVD, 2OVE, 2OVF, 2OVG, 2OVH, 2OVI, 2OVJ, 2OVL, 2OVM, 2OVO, 2OVP, 2OVQ, 2OVR, 2OVS, 2OVU, 2OVV, 2OVW, 2OVX, 2OVY, 2OVZ, 2OW0, 2OW1, 2OW2, 2OW3, 2OW4, 2OW6, 2OW7, 2OW9, 2OWA, 2OWB, 2OWC, 2OWD, 2OWE, 2OWF, 2OWG, 2OWH, 2OWJ, 2OWK, 2OWL, 2OWM, 2OWN, 2OWO, 2OWP, 2OWQ, 2OWR, 2OWS, 2OWT, 2OWU, 2OWV, 2OWW, 2OWX, 2OWY, 2OWZ, 2OX0, 2OX1, 2OX3, 2OX4, 2OX5, 2OX6, 2OX7, 2OX8, 2OX9, 2OXB, 2OXC, 2OXD, 2OXE, 2OXF, 2OXG, 2OXH, 2OXI, 2OXL, 2OXM, 2OXN, 2OXO, 2OXP, 2OXQ, 2OXR, 2OXS, 2OXT, 2OXU, 2OXV, 2OXW, 2OXX, 2OXY, 2OXZ, 2OY0, 2OY1, 2OY2, 2OY3, 2OY4, 2OY5, 2OY7, 2OY8, 2OY9, 2OYA, 2OYB, 2OYC, 2OYE, 2OYF, 2OYH, 2OYI, 2OYK, 2OYL, 2OYM, 2OYN, 2OYO, 2OYP, 2OYQ, 2OYR, 2OYS, 2OYT, 2OYU, 2OYY, 2OYZ, 2OZ0, 2OZ1, 2OZ2, 2OZ3, 2OZ4, 2OZ5, 2OZ6, 2OZ7, 2OZ8, 2OZ9, 2OZA, 2OZE, 2OZF, 2OZG, 2OZH, 2OZJ, 2OZK, 2OZL, 2OZM, 2OZN, 2OZO, 2OZP, 2OZQ, 2OZR, 2OZS, 2OZT, 2OZU, 2OZV, 2OZY, 2OZZ, 2P02, 2P04, 2P05, 2P06, 2P08, 2P09, 2P0A, 2P0B, 2P0C, 2P0D, 2P0E, 2P0F, 2P0G, 2P0H, 2P0I, 2P0J, 2P0K, 2P0L, 2P0M, 2P0N, 2P0O, 2P0R, 2P0S, 2P0T, 2P0U, 2P0V, 2P0W, 2P0Y, 2P10, 2P11, 2P12, 2P13, 2P14, 2P15, 2P16, 2P17, 2P18, 2P19, 2P1A, 2P1B, 2P1C, 2P1D, 2P1E, 2P1F, 2P1G, 2P1H, 2P1I, 2P1J, 2P1L, 2P1M, 2P1N, 2P1O, 2P1P, 2P1Q, 2P1R, 2P1S, 2P1T, 2P1U, 2P1V, 2P1W, 2P1X, 2P1Y, 2P1Z, 2P20, 2P22, 2P23, 2P24, 2P25, 2P26, 2P27, 2P28, 2P2A, 2P2B, 2P2C, 2P2D, 2P2E, 2P2F, 2P2G, 2P2H, 2P2I, 2P2J, 2P2K, 2P2L, 2P2M, 2P2N, 2P2O, 2P2Q, 2P2R, 2P2S, 2P2T, 2P2U, 2P2V, 2P2W, 2P2X, 2P2Y, 2P2Z, 2P30, 2P31, 2P32, 2P33, 2P34, 2P35, 2P37, 2P38, 2P39, 2P3A, 2P3B, 2P3C, 2P3D, 2P3E, 2P3F, 2P3G, 2P3H, 2P3I, 2P3J, 2P3K, 2P3L, 2P3N, 2P3O, 2P3P, 2P3Q, 2P3S, 2P3T, 2P3U, 2P3V, 2P3W, 2P3X, 2P3Y, 2P3Z, 2P40, 2P41, 2P42, 2P43, 2P44, 2P45, 2P46, 2P47, 2P48, 2P49, 2P4A, 2P4B, 2P4D, 2P4E, 2P4F, 2P4G, 2P4H, 2P4I, 2P4J, 2P4K, 2P4M, 2P4O, 2P4P, 2P4Q, 2P4R, 2P4S, 2P4T, 2P4U, 2P4V, 2P4W, 2P4X, 2P4Y, 2P4Z, 2P50, 2P51, 2P52, 2P53, 2P54, 2P55, 2P56, 2P57, 2P58, 2P59, 2P5B, 2P5C, 2P5D, 2P5E, 2P5F, 2P5G, 2P5I, 2P5K, 2P5L, 2P5M, 2P5N, 2P5O, 2P5P, 2P5Q, 2P5R, 2P5S, 2P5T, 2P5U, 2P5V, 2P5W, 2P5X, 2P5Y, 2P5Z, 2P61, 2P62, 2P63, 2P64, 2P65, 2P66, 2P67, 2P68, 2P69, 2P6A, 2P6B, 2P6C, 2P6D, 2P6E, 2P6F, 2P6G, 2P6H, 2P6I, 2P6K, 2P6L, 2P6M, 2P6N, 2P6O, 2P6P, 2P6R, 2P6S, 2P6T, 2P6U, 2P6V, 2P6W, 2P6X, 2P6Y, 2P6Z, 2P70, 2P71, 2P72, 2P73, 2P74, 2P75, 2P76, 2P77, 2P78, 2P79, 2P7A, 2P7G, 2P7H, 2P7I, 2P7J, 2P7K, 2P7L, 2P7M, 2P7N, 2P7O, 2P7P, 2P7Q, 2P7S, 2P7T, 2P7U, 2P7V, 2P7Z, 2P82, 2P83, 2P84, 2P85, 2P86, 2P87, 2P88, 2P8B, 2P8C, 2P8D, 2P8E, 2P8G, 2P8H, 2P8I, 2P8J, 2P8L, 2P8M, 2P8N, 2P8O, 2P8P, 2P8Q, 2P8R, 2P8S, 2P8T, 2P8U, 2P8V, 2P90, 2P91, 2P92, 2P93, 2P94, 2P95, 2P97, 2P98, 2P99, 2P9A, 2P9B, 2P9C, 2P9D, 2P9E, 2P9F, 2P9G, 2P9H, 2P9I, 2P9J, 2P9K, 2P9L, 2P9M, 2P9N, 2P9O, 2P9P, 2P9Q, 2P9R, 2P9S, 2P9T, 2P9U, 2P9V, 2P9W, 2P9X, 2P9Y, 2P9Z, 2PA0, 2PA1, 2PA2, 2PA3, 2PA4, 2PA5, 2PA6, 2PA7, 2PA8, 2PAA, 2PAB, 2PAD, 2PAE, 2PAF, 2PAG, 2PAH, 2PAJ, 2PAK, 2PAL, 2PAM, 2PAN, 2PAQ, 2PAR, 2PAU, 2PAV, 2PAW, 2PAX, 2PB0, 2PB1, 2PB2, 2PB4, 2PB5, 2PB6, 2PB7, 2PB8, 2PB9, 2PBC, 2PBD, 2PBE, 2PBF, 2PBG, 2PBH, 2PBI, 2PBJ, 2PBK, 2PBL, 2PBN, 2PBO, 2PBP, 2PBQ, 2PBR, 2PBW, 2PBX, 2PBY, 2PBZ, 2PC0, 2PC1, 2PC2, 2PC4, 2PC5, 2PC6, 2PC8, 2PC9, 2PCA, 2PCB, 2PCC, 2PCD, 2PCE, 2PCG, 2PCH, 2PCI, 2PCJ, 2PCK, 2PCL, 2PCM, 2PCN, 2PCP, 2PCQ, 2PCR, 2PCS, 2PCU, 2PCX, 2PCY, 2PD0, 2PD1, 2PD2, 2PD3, 2PD4, 2PD5, 2PD6, 2PD7, 2PD8, 2PD9, 2PDA, 2PDB, 2PDC, 2PDF, 2PDG, 2PDH, 2PDI, 2PDJ, 2PDK, 2PDL, 2PDM, 2PDN, 2PDO, 2PDP, 2PDQ, 2PDR, 2PDT, 2PDU, 2PDW, 2PDX, 2PDY, 2PE0, 2PE1, 2PE2, 2PE3, 2PE4, 2PE5, 2PE6, 2PE7, 2PE8, 2PEB, 2PEC, 2PED, 2PEE, 2PEF, 2PEG, 2PEH, 2PEI, 2PEJ, 2PEK, 2PEL, 2PEM, 2PEN, 2PEO, 2PEQ, 2PER, 2PES, 2PET, 2PEV, 2PEX, 2PEY, 2PEZ, 2PF0, 2PF1, 2PF2, 2PF4, 2PF5, 2PF6, 2PF8, 2PFB, 2PFC, 2PFD, 2PFE, 2PFG, 2PFH, 2PFI, 2PFJ, 2PFK, 2PFL, 2PFM, 2PFN, 2PFO, 2PFP, 2PFQ, 2PFR, 2PFS, 2PFT, 2PFV, 2PFW, 2PFX, 2PFY, 2PFZ, 2PG0, 2PG1, 2PG2, 2PG3, 2PG4, 2PG5, 2PG6, 2PG7, 2PG8, 2PGA, 2PGB, 2PGC, 2PGD, 2PGE, 2PGF, 2PGG, 2PGH, 2PGI, 2PGJ, 2PGK, 2PGL, 2PGN, 2PGO, 2PGQ, 2PGR, 2PGS, 2PGT, 2PGV, 2PGW, 2PGX, 2PGY, 2PGZ, 2PH0, 2PH1, 2PH3, 2PH4, 2PH5, 2PH6, 2PH7, 2PH8, 2PH9, 2PHA, 2PHB, 2PHC, 2PHD, 2PHF, 2PHH, 2PHI, 2PHK, 2PHL, 2PHM, 2PHN, 2PHO, 2PHP, 2PHR, 2PHT, 2PHU, 2PHW, 2PHX, 2PHY, 2PHZ, 2PI0, 2PI2, 2PI3, 2PI4, 2PI5, 2PI6, 2PI7, 2PI8, 2PIA, 2PIC, 2PID, 2PIE, 2PIF, 2PIG, 2PIH, 2PII, 2PIJ, 2PIL, 2PIM, 2PIN, 2PIO, 2PIP, 2PIQ, 2PIR, 2PIS, 2PIT, 2PIU, 2PIV, 2PIW, 2PIX, 2PIY, 2PIZ, 2PJ0, 2PJ1, 2PJ2, 2PJ3, 2PJ4, 2PJ5, 2PJ6, 2PJ7, 2PJ8, 2PJ9, 2PJA, 2PJB, 2PJC, 2PJD, 2PJJ, 2PJL, 2PJO, 2PJQ, 2PJS, 2PJT, 2PJU, 2PJW, 2PJY, 2PJZ, 2PK0, 2PK2, 2PK3, 2PK4, 2PK5, 2PK6, 2PK7, 2PK8, 2PK9, 2PKA, 2PKC, 2PKD, 2PKE, 2PKF, 2PKG, 2PKH, 2PKK, 2PKL, 2PKM, 2PKN, 2PKO, 2PKP, 2PKQ, 2PKR, 2PKS, 2PKT, 2PKV, 2PKW, 2PKX, 2PKY, 2PL0, 2PL1, 2PL2, 2PL3, 2PL4, 2PL5, 2PL6, 2PL7, 2PL8, 2PL9, 2PLA, 2PLB, 2PLC, 2PLF, 2PLG, 2PLH, 2PLI, 2PLJ, 2PLK, 2PLL, 2PLM, 2PLN, 2PLO, 2PLQ, 2PLR, 2PLS, 2PLT, 2PLU, 2PLW, 2PLX, 2PLZ, 2PM1, 2PM4, 2PM5, 2PM6, 2PM7, 2PM8, 2PM9, 2PMA, 2PMB, 2PMC, 2PMD, 2PME, 2PMF, 2PMH, 2PMI, 2PMJ, 2PMK, 2PML, 2PMN, 2PMO, 2PMP, 2PMQ, 2PMR, 2PMS, 2PMT, 2PMU, 2PMV, 2PMW, 2PMY, 2PMZ, 2PN0, 2PN1, 2PN2, 2PN5, 2PN6, 2PN7, 2PN8, 2PNC, 2PND, 2PNE, 2PNF, 2PNH, 2PNJ, 2PNK, 2PNL, 2PNM, 2PNN, 2PNO, 2PNQ, 2PNR, 2PNS, 2PNT, 2PNU, 2PNV, 2PNW, 2PNX, 2PNY, 2PNZ, 2PO0, 2PO2, 2PO3, 2PO4, 2PO5, 2PO6, 2PO7, 2POB, 2POC, 2POD, 2POE, 2POF, 2POG, 2POH, 2POI, 2POK, 2POL, 2POM, 2POO, 2POP, 2POQ, 2POR, 2POS, 2POT, 2POU, 2POV, 2POW, 2POX, 2POY, 2POZ, 2PP0, 2PP1, 2PP3, 2PP6, 2PP7, 2PP8, 2PP9, 2PPA, 2PPC, 2PPD, 2PPE, 2PPF, 2PPG, 2PPI, 2PPL, 2PPN, 2PPO, 2PPP, 2PPQ, 2PPT, 2PPV, 2PPW, 2PPX, 2PPY, 2PQ0, 2PQ2, 2PQ3, 2PQ5, 2PQ6, 2PQ7, 2PQ8, 2PQ9, 2PQA, 2PQB, 2PQC, 2PQD, 2PQF, 2PQG, 2PQI, 2PQJ, 2PQK, 2PQL, 2PQM, 2PQN, 2PQQ, 2PQR, 2PQS, 2PQT, 2PQU, 2PQV, 2PQW, 2PQX, 2PQY, 2PQZ, 2PR0, 2PR1, 2PR2, 2PR3, 2PR4, 2PR5, 2PR6, 2PR7, 2PR8, 2PR9, 2PRB, 2PRC, 2PRD, 2PRE, 2PRG, 2PRH, 2PRI, 2PRJ, 2PRK, 2PRL, 2PRM, 2PRN, 2PRO, 2PRQ, 2PRR, 2PRS, 2PRT, 2PRV, 2PRX, 2PRY, 2PRZ, 2PS0, 2PS1, 2PS2, 2PS3, 2PS4, 2PS5, 2PS6, 2PS7, 2PS8, 2PS9, 2PSB, 2PSD, 2PSE, 2PSF, 2PSG, 2PSH, 2PSJ, 2PSM, 2PSN, 2PSO, 2PSP, 2PSQ, 2PSR, 2PSS, 2PST, 2PSU, 2PSV, 2PSW, 2PSX, 2PSY, 2PSZ, 2PT0, 2PT1, 2PT2, 2PT3, 2PT5, 2PT6, 2PT7, 2PT9, 2PTC, 2PTD, 2PTF, 2PTG, 2PTH, 2PTK, 2PTM, 2PTN, 2PTQ, 2PTR, 2PTS, 2PTT, 2PTU, 2PTV, 2PTW, 2PTX, 2PTY, 2PTZ, 2PU0, 2PU1, 2PU2, 2PU3, 2PU4, 2PU5, 2PU7, 2PU8, 2PU9, 2PUA, 2PUB, 2PUC, 2PUD, 2PUE, 2PUF, 2PUG, 2PUH, 2PUI, 2PUJ, 2PUK, 2PUL, 2PUM, 2PUN, 2PUO, 2PUP, 2PUQ, 2PUR, 2PUS, 2PUT, 2PUU, 2PUV, 2PUW, 2PUX, 2PUY, 2PUZ, 2PV0, 2PV1, 2PV2, 2PV3, 2PV4, 2PV7, 2PV9, 2PVA, 2PVB, 2PVC, 2PVD, 2PVE, 2PVF, 2PVG, 2PVH, 2PVI, 2PVJ, 2PVK, 2PVL, 2PVM, 2PVN, 2PVO, 2PVP, 2PVQ, 2PVR, 2PVS, 2PVT, 2PVU, 2PVV, 2PVW, 2PVX, 2PVY, 2PVZ, 2PW0, 2PW1, 2PW2, 2PW3, 2PW5, 2PW6, 2PW7, 2PW8, 2PW9, 2PWA, 2PWB, 2PWC, 2PWD, 2PWE, 2PWF, 2PWG, 2PWH, 2PWJ, 2PWL, 2PWM, 2PWN, 2PWO, 2PWP, 2PWQ, 2PWR, 2PWS, 2PWU, 2PWV, 2PWW, 2PWX, 2PWY, 2PWZ, 2PX0, 2PX1, 2PX2, 2PX3, 2PX4, 2PX5, 2PX6, 2PX7, 2PX8, 2PXA, 2PXC, 2PXH, 2PXI, 2PXJ, 2PXR, 2PXS, 2PXW, 2PXX, 2PXY, 2PXZ, 2PY0, 2PY2, 2PY3, 2PY4, 2PY5, 2PY6, 2PY7, 2PY8, 2PYA, 2PYB, 2PYC, 2PYD, 2PYE, 2PYF, 2PYG, 2PYH, 2PYI, 2PYJ, 2PYK, 2PYL, 2PYM, 2PYN, 2PYO, 2PYP, 2PYQ, 2PYR, 2PYS, 2PYT, 2PYU, 2PYW, 2PYX, 2PYY, 2PYZ, 2PZ0, 2PZ1, 2PZ5, 2PZ8, 2PZ9, 2PZA, 2PZB, 2PZD, 2PZE, 2PZF, 2PZG, 2PZH, 2PZI, 2PZJ, 2PZK, 2PZL, 2PZM, 2PZN, 2PZP, 2PZR, 2PZS, 2PZT, 2PZU, 2PZV, 2PZW, 2PZX, 2PZY, 2PZZ, 2Q00, 2Q01, 2Q02, 2Q03, 2Q04, 2Q05, 2Q06, 2Q07, 2Q08, 2Q09, 2Q0A, 2Q0B, 2Q0C, 2Q0D, 2Q0E, 2Q0F, 2Q0G, 2Q0H, 2Q0I, 2Q0J, 2Q0K, 2Q0L, 2Q0M, 2Q0N, 2Q0O, 2Q0Q, 2Q0R, 2Q0S, 2Q0T, 2Q0U, 2Q0V, 2Q0X, 2Q0Y, 2Q0Z, 2Q10, 2Q11, 2Q12, 2Q13, 2Q14, 2Q15, 2Q16, 2Q17, 2Q18, 2Q19, 2Q1A, 2Q1B, 2Q1C, 2Q1D, 2Q1E, 2Q1F, 2Q1H, 2Q1J, 2Q1K, 2Q1L, 2Q1M, 2Q1N, 2Q1P, 2Q1Q, 2Q1S, 2Q1T, 2Q1U, 2Q1V, 2Q1W, 2Q1X, 2Q1Y, 2Q1Z, 2Q20, 2Q21, 2Q22, 2Q24, 2Q27, 2Q28, 2Q29, 2Q2A, 2Q2B, 2Q2C, 2Q2E, 2Q2F, 2Q2G, 2Q2H, 2Q2I, 2Q2J, 2Q2K, 2Q2L, 2Q2M, 2Q2N, 2Q2O, 2Q2P, 2Q2Q, 2Q2R, 2Q2T, 2Q2U, 2Q2V, 2Q2W, 2Q2X, 2Q2Y, 2Q2Z, 2Q30, 2Q31, 2Q32, 2Q34, 2Q35, 2Q36, 2Q37, 2Q38, 2Q39, 2Q3A, 2Q3B, 2Q3C, 2Q3D, 2Q3E, 2Q3F, 2Q3G, 2Q3H, 2Q3J, 2Q3K, 2Q3L, 2Q3M, 2Q3N, 2Q3O, 2Q3P, 2Q3Q, 2Q3R, 2Q3S, 2Q3T, 2Q3U, 2Q3V, 2Q3W, 2Q3X, 2Q3Y, 2Q3Z, 2Q40, 2Q41, 2Q42, 2Q43, 2Q44, 2Q45, 2Q46, 2Q47, 2Q48, 2Q49, 2Q4A, 2Q4B, 2Q4C, 2Q4D, 2Q4E, 2Q4F, 2Q4G, 2Q4H, 2Q4I, 2Q4J, 2Q4K, 2Q4L, 2Q4M, 2Q4N, 2Q4O, 2Q4P, 2Q4Q, 2Q4R, 2Q4S, 2Q4T, 2Q4U, 2Q4V, 2Q4W, 2Q4X, 2Q4Y, 2Q4Z, 2Q50, 2Q51, 2Q52, 2Q53, 2Q54, 2Q55, 2Q57, 2Q58, 2Q59, 2Q5A, 2Q5B, 2Q5C, 2Q5D, 2Q5E, 2Q5F, 2Q5G, 2Q5H, 2Q5I, 2Q5J, 2Q5K, 2Q5L, 2Q5O, 2Q5P, 2Q5Q, 2Q5R, 2Q5S, 2Q5T, 2Q5U, 2Q5W, 2Q5X, 2Q5Y, 2Q5Z, 2Q60, 2Q61, 2Q62, 2Q63, 2Q64, 2Q67, 2Q68, 2Q69, 2Q6A, 2Q6B, 2Q6C, 2Q6D, 2Q6E, 2Q6F, 2Q6G, 2Q6H, 2Q6I, 2Q6J, 2Q6K, 2Q6L, 2Q6M, 2Q6N, 2Q6O, 2Q6P, 2Q6Q, 2Q6R, 2Q6S, 2Q6T, 2Q6U, 2Q6V, 2Q6W, 2Q6Z, 2Q70, 2Q71, 2Q72, 2Q73, 2Q74, 2Q76, 2Q78, 2Q79, 2Q7A, 2Q7B, 2Q7C, 2Q7D, 2Q7E, 2Q7F, 2Q7G, 2Q7H, 2Q7I, 2Q7J, 2Q7K, 2Q7L, 2Q7M, 2Q7N, 2Q7O, 2Q7Q, 2Q7R, 2Q7S, 2Q7T, 2Q7U, 2Q7V, 2Q7W, 2Q7X, 2Q7Y, 2Q80, 2Q81, 2Q82, 2Q83, 2Q85, 2Q86, 2Q87, 2Q88, 2Q89, 2Q8A, 2Q8B, 2Q8C, 2Q8D, 2Q8E, 2Q8F, 2Q8G, 2Q8H, 2Q8I, 2Q8J, 2Q8K, 2Q8L, 2Q8M, 2Q8N, 2Q8O, 2Q8P, 2Q8Q, 2Q8R, 2Q8S, 2Q8T, 2Q8U, 2Q8V, 2Q8W, 2Q8X, 2Q8Y, 2Q8Z, 2Q91, 2Q92, 2Q93, 2Q94, 2Q95, 2Q96, 2Q97, 2Q98, 2Q99, 2Q9A, 2Q9B, 2Q9C, 2Q9D, 2Q9E, 2Q9F, 2Q9G, 2Q9H, 2Q9I, 2Q9J, 2Q9K, 2Q9L, 2Q9M, 2Q9N, 2Q9O, 2Q9P, 2Q9Q, 2Q9R, 2Q9S, 2Q9T, 2Q9U, 2Q9V, 2Q9X, 2Q9Y, 2Q9Z, 2QA1, 2QA2, 2QA3, 2QA5, 2QA6, 2QA7, 2QA8, 2QA9, 2QAA, 2QAB, 2QAC, 2QAD, 2QAE, 2QAF, 2QAG, 2QAI, 2QAJ, 2QAK, 2QAP, 2QAR, 2QAS, 2QAZ, 2QB0, 2QB1, 2QB2, 2QB3, 2QB4, 2QB5, 2QB6, 2QB7, 2QB8, 2QBL, 2QBM, 2QBN, 2QBO, 2QBP, 2QBQ, 2QBR, 2QBS, 2QBT, 2QBU, 2QBV, 2QBW, 2QBX, 2QBY, 2QC1, 2QC2, 2QC3, 2QC5, 2QC6, 2QC7, 2QC8, 2QC9, 2QCA, 2QCB, 2QCC, 2QCD, 2QCE, 2QCF, 2QCG, 2QCH, 2QCI, 2QCJ, 2QCK, 2QCL, 2QCM, 2QCN, 2QCO, 2QCP, 2QCQ, 2QCS, 2QCT, 2QCU, 2QCV, 2QCW, 2QCX, 2QCY, 2QCZ, 2QD0, 2QD1, 2QD2, 2QD3, 2QD4, 2QD5, 2QD6, 2QD7, 2QD8, 2QD9, 2QDB, 2QDC, 2QDD, 2QDE, 2QDF, 2QDG, 2QDH, 2QDI, 2QDJ, 2QDK, 2QDL, 2QDM, 2QDN, 2QDO, 2QDP, 2QDQ, 2QDR, 2QDS, 2QDT, 2QDV, 2QDW, 2QDX, 2QDY, 2QDZ, 2QE0, 2QE2, 2QE3, 2QE4, 2QE5, 2QE6, 2QE7, 2QE8, 2QE9, 2QEA, 2QEB, 2QEC, 2QED, 2QEE, 2QEF, 2QEG, 2QEH, 2QEI, 2QEJ, 2QEL, 2QEN, 2QEO, 2QEP, 2QEQ, 2QER, 2QES, 2QET, 2QEU, 2QEV, 2QEW, 2QEY, 2QEZ, 2QF0, 2QF1, 2QF2, 2QF3, 2QF4, 2QF5, 2QF6, 2QF7, 2QF8, 2QF9, 2QFA, 2QFB, 2QFC, 2QFD, 2QFE, 2QFF, 2QFI, 2QFK, 2QFL, 2QFN, 2QFO, 2QFP, 2QFQ, 2QFR, 2QFS, 2QFT, 2QFU, 2QFV, 2QFW, 2QFX, 2QFY, 2QFZ, 2QG0, 2QG1, 2QG2, 2QG3, 2QG4, 2QG5, 2QG6, 2QG7, 2QG8, 2QG9, 2QGA, 2QGB, 2QGC, 2QGD, 2QGE, 2QGF, 2QGG, 2QGH, 2QGI, 2QGM, 2QGN, 2QGO, 2QGQ, 2QGR, 2QGS, 2QGT, 2QGU, 2QGV, 2QGW, 2QGX, 2QGY, 2QGZ, 2QH0, 2QH1, 2QH5, 2QH6, 2QH7, 2QH9, 2QHA, 2QHB, 2QHC, 2QHD, 2QHE, 2QHF, 2QHK, 2QHL, 2QHM, 2QHN, 2QHO, 2QHP, 2QHQ, 2QHR, 2QHS, 2QHT, 2QHU, 2QHV, 2QHW, 2QHX, 2QHY, 2QHZ, 2QI0, 2QI1, 2QI2, 2QI3, 2QI4, 2QI5, 2QI6, 2QI7, 2QI8, 2QI9, 2QIA, 2QIB, 2QIC, 2QIE, 2QIF, 2QIH, 2QII, 2QIK, 2QIL, 2QIM, 2QIN, 2QIO, 2QIP, 2QIQ, 2QIR, 2QIS, 2QIU, 2QIV, 2QIW, 2QIY, 2QIZ, 2QJ0, 2QJ1, 2QJ2, 2QJ3, 2QJ4, 2QJ5, 2QJ6, 2QJ7, 2QJ8, 2QJ9, 2QJA, 2QJB, 2QJC, 2QJD, 2QJE, 2QJF, 2QJG, 2QJH, 2QJI, 2QJJ, 2QJK, 2QJL, 2QJM, 2QJN, 2QJO, 2QJP, 2QJR, 2QJS, 2QJT, 2QJU, 2QJV, 2QJW, 2QJX, 2QJY, 2QJZ, 2QK0, 2QK1, 2QK2, 2QK4, 2QK5, 2QK7, 2QK8, 2QKA, 2QKC, 2QKD, 2QKE, 2QKF, 2QKH, 2QKI, 2QKL, 2QKM, 2QKN, 2QKO, 2QKP, 2QKQ, 2QKR, 2QKS, 2QKT, 2QKU, 2QKV, 2QKW, 2QKX, 2QKY, 2QL1, 2QL2, 2QL3, 2QL5, 2QL6, 2QL7, 2QL8, 2QL9, 2QLA, 2QLB, 2QLC, 2QLD, 2QLE, 2QLF, 2QLG, 2QLH, 2QLI, 2QLJ, 2QLK, 2QLL, 2QLM, 2QLN, 2QLP, 2QLQ, 2QLR, 2QLS, 2QLT, 2QLU, 2QLV, 2QLW, 2QLX, 2QLY, 2QLZ, 2QM0, 2QM1, 2QM2, 2QM3, 2QM4, 2QM6, 2QM7, 2QM8, 2QM9, 2QMA, 2QMB, 2QMC, 2QMD, 2QME, 2QMF, 2QMG, 2QMH, 2QMI, 2QMJ, 2QMK, 2QML, 2QMM, 2QMO, 2QMP, 2QMQ, 2QMR, 2QMS, 2QMT, 2QMU, 2QMW, 2QMX, 2QMY, 2QMZ, 2QN0, 2QN1, 2QN2, 2QN3, 2QN4, 2QN5, 2QN6, 2QN7, 2QN8, 2QN9, 2QNA, 2QNB, 2QNC, 2QND, 2QNE, 2QNF, 2QNG, 2QNI, 2QNJ, 2QNK, 2QNL, 2QNN, 2QNO, 2QNP, 2QNQ, 2QNR, 2QNT, 2QNU, 2QNV, 2QNW, 2QNX, 2QNY, 2QNZ, 2QO0, 2QO1, 2QO2, 2QO3, 2QO4, 2QO5, 2QO6, 2QO7, 2QO8, 2QO9, 2QOA, 2QOB, 2QOC, 2QOD, 2QOE, 2QOF, 2QOG, 2QOH, 2QOI, 2QOJ, 2QOK, 2QOL, 2QOM, 2QON, 2QOO, 2QOP, 2QOQ, 2QOR, 2QOS, 2QP2, 2QP3, 2QP4, 2QP6, 2QP8, 2QP9, 2QPA, 2QPD, 2QPE, 2QPF, 2QPJ, 2QPK, 2QPL, 2QPM, 2QPN, 2QPO, 2QPP, 2QPQ, 2QPS, 2QPT, 2QPU, 2QPV, 2QPW, 2QPX, 2QPY, 2QPZ, 2QQ0, 2QQ1, 2QQ2, 2QQ3, 2QQ4, 2QQ5, 2QQ6, 2QQ7, 2QQ8, 2QQ9, 2QQA, 2QQB, 2QQC, 2QQD, 2QQE, 2QQF, 2QQG, 2QQH, 2QQI, 2QQJ, 2QQK, 2QQL, 2QQM, 2QQN, 2QQO, 2QQQ, 2QQR, 2QQS, 2QQT, 2QQU, 2QQV, 2QQW, 2QQY, 2QQZ, 2QR0, 2QR1, 2QR2, 2QR3, 2QR4, 2QR5, 2QR6, 2QR7, 2QR8, 2QR9, 2QRA, 2QRB, 2QRC, 2QRD, 2QRE, 2QRF, 2QRG, 2QRH, 2QRI, 2QRJ, 2QRK, 2QRL, 2QRM, 2QRN, 2QRO, 2QRP, 2QRQ, 2QRR, 2QRS, 2QRT, 2QRU, 2QRV, 2QRW, 2QRX, 2QRY, 2QRZ, 2QS1, 2QS2, 2QS3, 2QS4, 2QS6, 2QS7, 2QS8, 2QS9, 2QSA, 2QSB, 2QSC, 2QSD, 2QSE, 2QSF, 2QSG, 2QSH, 2QSI, 2QSJ, 2QSK, 2QSP, 2QSQ, 2QSR, 2QSS, 2QST, 2QSU, 2QSV, 2QSW, 2QSX, 2QSY, 2QSZ, 2QT0, 2QT1, 2QT2, 2QT3, 2QT4, 2QT5, 2QT6, 2QT7, 2QT8, 2QT9, 2QTA, 2QTB, 2QTC, 2QTD, 2QTE, 2QTF, 2QTG, 2QTH, 2QTI, 2QTK, 2QTL, 2QTM, 2QTN, 2QTO, 2QTP, 2QTQ, 2QTR, 2QTS, 2QTT, 2QTU, 2QTV, 2QTW, 2QTX, 2QTY, 2QTZ, 2QU0, 2QU1, 2QU2, 2QU3, 2QU5, 2QU6, 2QU7, 2QU8, 2QU9, 2QUA, 2QUB, 2QUD, 2QUE, 2QUF, 2QUG, 2QUH, 2QUI, 2QUJ, 2QUK, 2QUL, 2QUM, 2QUN, 2QUO, 2QUP, 2QUQ, 2QUR, 2QUT, 2QUU, 2QUV, 2QUY, 2QUZ, 2QV0, 2QV1, 2QV2, 2QV3, 2QV4, 2QV5, 2QV6, 2QV7, 2QV8, 2QVA, 2QVB, 2QVC, 2QVD, 2QVE, 2QVF, 2QVG, 2QVH, 2QVI, 2QVJ, 2QVK, 2QVL, 2QVM, 2QVN, 2QVO, 2QVP, 2QVR, 2QVS, 2QVT, 2QVU, 2QVV, 2QVW, 2QVX, 2QVY, 2QVZ, 2QW0, 2QW1, 2QW4, 2QW5, 2QW6, 2QW7, 2QW8, 2QW9, 2QWA, 2QWB, 2QWC, 2QWD, 2QWE, 2QWF, 2QWG, 2QWH, 2QWI, 2QWJ, 2QWK, 2QWL, 2QWM, 2QWN, 2QWO, 2QWP, 2QWQ, 2QWR, 2QWS, 2QWT, 2QWU, 2QWV, 2QWW, 2QWX, 2QWZ, 2QX0, 2QX1, 2QX2, 2QX3, 2QX4, 2QX5, 2QX6, 2QX7, 2QX8, 2QX9, 2QXF, 2QXG, 2QXH, 2QXI, 2QXJ, 2QXL, 2QXM, 2QXS, 2QXT, 2QXU, 2QXV, 2QXW, 2QXX, 2QXY, 2QXZ, 2QY0, 2QY1, 2QY2, 2QY6, 2QY7, 2QY9, 2QYA, 2QYB, 2QYC, 2QYF, 2QYG, 2QYH, 2QYI, 2QYJ, 2QYK, 2QYL, 2QYM, 2QYN, 2QYO, 2QYP, 2QYQ, 2QYS, 2QYT, 2QYU, 2QYV, 2QYW, 2QYZ, 2QZ0, 2QZ2, 2QZ3, 2QZ4, 2QZ5, 2QZ6, 2QZ7, 2QZ8, 2QZ9, 2QZA, 2QZB, 2QZC, 2QZE, 2QZG, 2QZI, 2QZJ, 2QZK, 2QZL, 2QZO, 2QZP, 2QZQ, 2QZR, 2QZS, 2QZT, 2QZU, 2QZV, 2QZW, 2QZX, 2QZY, 2QZZ, 2R00, 2R01, 2R02, 2R03, 2R05, 2R09, 2R0B, 2R0C, 2R0D, 2R0F, 2R0G, 2R0H, 2R0I, 2R0J, 2R0K, 2R0L, 2R0M, 2R0N, 2R0O, 2R0P, 2R0Q, 2R0R, 2R0S, 2R0T, 2R0U, 2R0V, 2R0W, 2R0X, 2R0Y, 2R0Z, 2R10, 2R11, 2R13, 2R14, 2R15, 2R16, 2R17, 2R18, 2R19, 2R1A, 2R1B, 2R1D, 2R1F, 2R1H, 2R1I, 2R1J, 2R1K, 2R1L, 2R1M, 2R1N, 2R1P, 2R1Q, 2R1R, 2R1T, 2R1U, 2R1V, 2R1W, 2R1X, 2R1Y, 2R1Z, 2R23, 2R24, 2R25, 2R26, 2R27, 2R28, 2R29, 2R2A, 2R2B, 2R2C, 2R2D, 2R2E, 2R2F, 2R2G, 2R2H, 2R2I, 2R2J, 2R2K, 2R2L, 2R2M, 2R2N, 2R2O, 2R2P, 2R2Q, 2R2R, 2R2S, 2R2T, 2R2U, 2R2V, 2R2W, 2R2X, 2R2Y, 2R2Z, 2R30, 2R31, 2R32, 2R33, 2R34, 2R35, 2R36, 2R37, 2R38, 2R39, 2R3A, 2R3B, 2R3D, 2R3E, 2R3F, 2R3G, 2R3H, 2R3I, 2R3J, 2R3K, 2R3L, 2R3M, 2R3N, 2R3O, 2R3P, 2R3Q, 2R3R, 2R3S, 2R3T, 2R3U, 2R3V, 2R3W, 2R3X, 2R3Y, 2R3Z, 2R40, 2R41, 2R42, 2R43, 2R44, 2R45, 2R46, 2R47, 2R48, 2R49, 2R4B, 2R4E, 2R4F, 2R4G, 2R4H, 2R4I, 2R4J, 2R4L, 2R4N, 2R4O, 2R4P, 2R4Q, 2R4R, 2R4S, 2R4T, 2R4U, 2R4V, 2R4W, 2R4X, 2R4Y, 2R4Z, 2R50, 2R51, 2R52, 2R53, 2R55, 2R56, 2R57, 2R58, 2R59, 2R5A, 2R5C, 2R5E, 2R5F, 2R5G, 2R5H, 2R5I, 2R5J, 2R5K, 2R5L, 2R5M, 2R5N, 2R5O, 2R5P, 2R5Q, 2R5R, 2R5S, 2R5T, 2R5U, 2R5V, 2R5W, 2R5X, 2R5Y, 2R5Z, 2R60, 2R61, 2R62, 2R64, 2R65, 2R66, 2R68, 2R69, 2R6A, 2R6C, 2R6D, 2R6E, 2R6F, 2R6G, 2R6H, 2R6I, 2R6J, 2R6K, 2R6M, 2R6N, 2R6O, 2R6Q, 2R6R, 2R6S, 2R6T, 2R6U, 2R6V, 2R6W, 2R6X, 2R6Y, 2R6Z, 2R70, 2R71, 2R72, 2R73, 2R74, 2R75, 2R76, 2R77, 2R78, 2R79, 2R7A, 2R7B, 2R7C, 2R7D, 2R7E, 2R7F, 2R7G, 2R7H, 2R7I, 2R7J, 2R7K, 2R7L, 2R7M, 2R7N, 2R7O, 2R7P, 2R7Q, 2R80, 2R82, 2R83, 2R84, 2R85, 2R86, 2R87, 2R88, 2R89, 2R8A, 2R8B, 2R8D, 2R8E, 2R8F, 2R8G, 2R8H, 2R8I, 2R8J, 2R8K, 2R8N, 2R8O, 2R8P, 2R8Q, 2R8R, 2R8T, 2R8U, 2R8V, 2R8W, 2R8X, 2R8Y, 2R8Z, 2R90, 2R91, 2R94, 2R96, 2R97, 2R98, 2R99, 2R9A, 2R9B, 2R9C, 2R9E, 2R9F, 2R9G, 2R9H, 2R9I, 2R9J, 2R9K, 2R9L, 2R9M, 2R9N, 2R9O, 2R9P, 2R9Q, 2R9R, 2R9S, 2R9U, 2R9V, 2R9W, 2R9X, 2R9Y, 2R9Z, 2RA0, 2RA1, 2RA2, 2RA3, 2RA4, 2RA5, 2RA6, 2RA8, 2RA9, 2RAA, 2RAB, 2RAC, 2RAD, 2RAE, 2RAF, 2RAG, 2RAH, 2RAI, 2RAJ, 2RAK, 2RAL, 2RAM, 2RAN, 2RAO, 2RAP, 2RAQ, 2RAR, 2RAS, 2RAT, 2RAU, 2RAV, 2RAW, 2RAX, 2RAY, 2RAZ, 2RB0, 2RB1, 2RB2, 2RB3, 2RB4, 2RB5, 2RB6, 2RB7, 2RB8, 2RB9, 2RBA, 2RBB, 2RBC, 2RBD, 2RBE, 2RBF, 2RBG, 2RBH, 2RBI, 2RBK, 2RBL, 2RBM, 2RBN, 2RBO, 2RBP, 2RBQ, 2RBR, 2RBS, 2RBT, 2RBU, 2RBV, 2RBW, 2RBX, 2RBY, 2RBZ, 2RC0, 2RC1, 2RC2, 2RC3, 2RC4, 2RC5, 2RC6, 2RC7, 2RC8, 2RC9, 2RCA, 2RCB, 2RCC, 2RCD, 2RCE, 2RCF, 2RCH, 2RCI, 2RCK, 2RCL, 2RCM, 2RCN, 2RCQ, 2RCR, 2RCS, 2RCT, 2RCU, 2RCV, 2RCW, 2RCX, 2RCY, 2RCZ, 2RD0, 2RD1, 2RD3, 2RD4, 2RD5, 2RD6, 2RD7, 2RD8, 2RD9, 2RDA, 2RDB, 2RDC, 2RDD, 2RDE, 2RDF, 2RDG, 2RDH, 2RDI, 2RDJ, 2RDK, 2RDL, 2RDM, 2RDN, 2RDP, 2RDQ, 2RDR, 2RDS, 2RDT, 2RDU, 2RDV, 2RDW, 2RDX, 2RDY, 2RDZ, 2RE1, 2RE2, 2RE3, 2RE7, 2RE9, 2REA, 2REB, 2RED, 2REE, 2REF, 2REG, 2REH, 2REI, 2REJ, 2REK, 2REM, 2REN, 2REO, 2REP, 2REQ, 2RER, 2RES, 2RET, 2REU, 2REW, 2REX, 2REY, 2REZ, 2RF0, 2RF1, 2RF2, 2RF3, 2RF4, 2RF5, 2RF6, 2RF7, 2RF8, 2RF9, 2RFA, 2RFB, 2RFC, 2RFD, 2RFE, 2RFF, 2RFG, 2RFH, 2RFI, 2RFJ, 2RFL, 2RFM, 2RFN, 2RFO, 2RFQ, 2RFR, 2RFS, 2RFT, 2RFU, 2RFV, 2RFW, 2RFX, 2RFY, 2RFZ, 2RG0, 2RG1, 2RG2, 2RG3, 2RG4, 2RG5, 2RG6, 2RG7, 2RG8, 2RG9, 2RGA, 2RGB, 2RGC, 2RGD, 2RGE, 2RGG, 2RGH, 2RGI, 2RGJ, 2RGK, 2RGL, 2RGM, 2RGN, 2RGO, 2RGP, 2RGQ, 2RGR, 2RGS, 2RGT, 2RGU, 2RGV, 2RGW, 2RGX, 2RGY, 2RGZ, 2RH0, 2RH1, 2RH2, 2RH3, 2RH4, 2RH5, 2RH6, 2RH7, 2RH8, 2RH9, 2RHA, 2RHB, 2RHC, 2RHD, 2RHE, 2RHF, 2RHG, 2RHH, 2RHI, 2RHJ, 2RHK, 2RHL, 2RHM, 2RHO, 2RHP, 2RHQ, 2RHR, 2RHS, 2RHT, 2RHU, 2RHW, 2RHX, 2RHY, 2RHZ, 2RI0, 2RI1, 2RI2, 2RI3, 2RI4, 2RI5, 2RI6, 2RI7, 2RI8, 2RI9, 2RIA, 2RIB, 2RIC, 2RID, 2RIE, 2RIF, 2RIG, 2RIH, 2RII, 2RIJ, 2RIK, 2RIL, 2RIM, 2RIN, 2RIO, 2RIP, 2RIQ, 2RIR, 2RIS, 2RIT, 2RIU, 2RIV, 2RIW, 2RIX, 2RIY, 2RIZ, 2RJ0, 2RJ1, 2RJ2, 2RJ3, 2RJ4, 2RJ5, 2RJ6, 2RJ7, 2RJ8, 2RJ9, 2RJB, 2RJC, 2RJD, 2RJE, 2RJF, 2RJG, 2RJH, 2RJI, 2RJK, 2RJL, 2RJM, 2RJN, 2RJO, 2RJP, 2RJQ, 2RJR, 2RJS, 2RJT, 2RJV, 2RJW, 2RJX, 2RJY, 2RJZ, 2RK0, 2RK1, 2RK2, 2RK3, 2RK4, 2RK5, 2RK6, 2RK7, 2RK8, 2RK9, 2RKA, 2RKB, 2RKC, 2RKD, 2RKE, 2RKF, 2RKG, 2RKH, 2RKI, 2RKK, 2RKL, 2RKM, 2RKN, 2RKO, 2RKQ, 2RKS, 2RKT, 2RKU, 2RKV, 2RKW, 2RKX, 2RKY, 2RKZ, 2RL0, 2RL1, 2RL2, 2RL3, 2RL5, 2RL7, 2RL8, 2RL9, 2RLA, 2RLB, 2RLC, 2RLD, 2RLN, 2RMA, 2RMB, 2RMC, 2RMP, 2RN2, 2RNF, 2RNS, 2RNT, 2ROM, 2ROX, 2ROY, 2RSL, 2RSP, 2RTA, 2RTB, 2RTC, 2RTD, 2RTE, 2RTF, 2RTG, 2RTH, 2RTI, 2RTJ, 2RTK, 2RTL, 2RTM, 2RTN, 2RTO, 2RTP, 2RTQ, 2RTR, 2RUS, 2RVE, 2SAK, 2SAM, 2SAR, 2SAS, 2SBA, 2SBL, 2SBT, 2SCP, 2SCU, 2SEB, 2SEC, 2SEM, 2SFA, 2SFP, 2SGA, 2SGD, 2SGE, 2SGF, 2SGP, 2SGQ, 2SHK, 2SHP, 2SIC, 2SIL, 2SIM, 2SIV, 2SKC, 2SKD, 2SKE, 2SLI, 2SN3, 2SNI, 2SNM, 2SNS, 2SNV, 2SNW, 2SOD, 2SPC, 2SPG, 2SPL, 2SPM, 2SPN, 2SPO, 2SPT, 2SQC, 2SRC, 2SSP, 2ST1, 2STA, 2STB, 2STD, 2TAA, 2TBS, 2TCI, 2TCL, 2TCT, 2TDD, 2TDM, 2TDT, 2TDX, 2TEC, 2TEP, 2TGA, 2TGD, 2TGI, 2TGP, 2TGT, 2THF, 2THI, 2TIO, 2TIR, 2TLD, 2TLI, 2TLX, 2TMA, 2TMD, 2TMG, 2TMK, 2TMN, 2TMY, 2TN4, 2TNF, 2TOD, 2TOH, 2TPI, 2TPL, 2TPR, 2TPS, 2TPT, 2TRC, 2TRH, 2TRM, 2TRS, 2TRT, 2TRX, 2TRY, 2TS1, 2TSA, 2TSB, 2TSC, 2TSR, 2TSS, 2TSY, 2TUN, 2TYS, 2UAG, 2UBP, 2UCZ, 2UDP, 2UGI, 2UKD, 2ULL, 2UP1, 2UPJ, 2USH, 2USN, 2UTG, 2UU7, 2UU8, 2UUD, 2UUE, 2UUF, 2UUG, 2UUH, 2UUI, 2UUJ, 2UUK, 2UUL, 2UUM, 2UUN, 2UUO, 2UUP, 2UUQ, 2UUR, 2UUS, 2UUT, 2UUU, 2UUV, 2UUW, 2UUX, 2UUY, 2UUZ, 2UV0, 2UV1, 2UV2, 2UV3, 2UV4, 2UV5, 2UV6, 2UV7, 2UV9, 2UVB, 2UVD, 2UVE, 2UVF, 2UVG, 2UVH, 2UVI, 2UVJ, 2UVK, 2UVL, 2UVM, 2UVN, 2UVO, 2UVP, 2UVQ, 2UVR, 2UVU, 2UVV, 2UVW, 2UVX, 2UVY, 2UVZ, 2UW0, 2UW1, 2UW2, 2UW3, 2UW4, 2UW5, 2UW6, 2UW7, 2UW8, 2UW9, 2UWA, 2UWB, 2UWC, 2UWD, 2UWE, 2UWF, 2UWH, 2UWI, 2UWJ, 2UWL, 2UWN, 2UWO, 2UWP, 2UWR, 2UWS, 2UWT, 2UWU, 2UWV, 2UWW, 2UWX, 2UX0, 2UX1, 2UX2, 2UX3, 2UX4, 2UX5, 2UX6, 2UX7, 2UX8, 2UX9, 2UXA, 2UXE, 2UXF, 2UXG, 2UXH, 2UXI, 2UXJ, 2UXK, 2UXL, 2UXM, 2UXN, 2UXO, 2UXP, 2UXQ, 2UXR, 2UXS, 2UXT, 2UXU, 2UXV, 2UXW, 2UXX, 2UXY, 2UXZ, 2UY0, 2UY1, 2UY2, 2UY3, 2UY4, 2UY5, 2UY6, 2UY7, 2UY8, 2UY9, 2UYA, 2UYB, 2UYC, 2UYD, 2UYE, 2UYF, 2UYG, 2UYH, 2UYI, 2UYJ, 2UYK, 2UYL, 2UYM, 2UYN, 2UYO, 2UYP, 2UYQ, 2UYR, 2UYT, 2UYU, 2UYV, 2UYW, 2UYX, 2UYY, 2UYZ, 2UZ0, 2UZ1, 2UZ2, 2UZ3, 2UZ4, 2UZ6, 2UZ8, 2UZ9, 2UZA, 2UZB, 2UZC, 2UZD, 2UZE, 2UZF, 2UZH, 2UZI, 2UZJ, 2UZK, 2UZL, 2UZN, 2UZO, 2UZP, 2UZQ, 2UZR, 2UZS, 2UZT, 2UZU, 2UZV, 2UZW, 2UZX, 2UZY, 2UZZ, 2V00, 2V01, 2V02, 2V03, 2V04, 2V05, 2V06, 2V07, 2V08, 2V09, 2V0A, 2V0B, 2V0C, 2V0D, 2V0H, 2V0I, 2V0J, 2V0K, 2V0L, 2V0M, 2V0N, 2V0O, 2V0P, 2V0R, 2V0S, 2V0T, 2V0U, 2V0V, 2V0W, 2V0X, 2V0Y, 2V0Z, 2V10, 2V11, 2V12, 2V13, 2V14, 2V15, 2V16, 2V17, 2V18, 2V19, 2V1A, 2V1B, 2V1C, 2V1D, 2V1E, 2V1F, 2V1G, 2V1H, 2V1I, 2V1J, 2V1K, 2V1L, 2V1M, 2V1O, 2V1P, 2V1Q, 2V1R, 2V1S, 2V1T, 2V1U, 2V1W, 2V1X, 2V1Y, 2V1Z, 2V20, 2V21, 2V22, 2V23, 2V24, 2V25, 2V26, 2V27, 2V28, 2V29, 2V2A, 2V2B, 2V2C, 2V2D, 2V2E, 2V2F, 2V2G, 2V2H, 2V2I, 2V2J, 2V2K, 2V2L, 2V2M, 2V2N, 2V2O, 2V2P, 2V2Q, 2V2R, 2V2S, 2V2T, 2V2U, 2V2V, 2V2W, 2V2X, 2V2Z, 2V30, 2V32, 2V33, 2V34, 2V35, 2V36, 2V38, 2V3A, 2V3B, 2V3D, 2V3E, 2V3F, 2V3G, 2V3H, 2V3I, 2V3J, 2V3K, 2V3M, 2V3N, 2V3O, 2V3P, 2V3Q, 2V3R, 2V3S, 2V3T, 2V3U, 2V3V, 2V3W, 2V3X, 2V3Y, 2V3Z, 2V40, 2V41, 2V42, 2V43, 2V45, 2V4A, 2V4B, 2V4C, 2V4D, 2V4E, 2V4H, 2V4I, 2V4J, 2V4L, 2V4M, 2V4N, 2V4O, 2V4Q, 2V4R, 2V4U, 2V4V, 2V4X, 2V4Y, 2V4Z, 2V50, 2V51, 2V52, 2V53, 2V54, 2V55, 2V57, 2V58, 2V59, 2V5A, 2V5B, 2V5C, 2V5D, 2V5E, 2V5F, 2V5G, 2V5H, 2V5I, 2V5J, 2V5K, 2V5L, 2V5M, 2V5N, 2V5O, 2V5P, 2V5Q, 2V5R, 2V5S, 2V5T, 2V5U, 2V5V, 2V5W, 2V5X, 2V5Y, 2V5Z, 2V60, 2V61, 2V62, 2V63, 2V64, 2V65, 2V66, 2V67, 2V68, 2V69, 2V6A, 2V6B, 2V6C, 2V6E, 2V6F, 2V6G, 2V6H, 2V6I, 2V6J, 2V6K, 2V6M, 2V6N, 2V6O, 2V6Q, 2V6S, 2V6T, 2V6U, 2V6V, 2V6X, 2V6Y, 2V70, 2V71, 2V72, 2V73, 2V74, 2V75, 2V76, 2V77, 2V78, 2V79, 2V7A, 2V7B, 2V7C, 2V7D, 2V7E, 2V7F, 2V7G, 2V7H, 2V7I, 2V7J, 2V7K, 2V7L, 2V7M, 2V7N, 2V7O, 2V7P, 2V7Q, 2V7S, 2V7T, 2V7U, 2V7V, 2V7W, 2V7X, 2V7Y, 2V7Z, 2V81, 2V82, 2V83, 2V84, 2V85, 2V86, 2V87, 2V88, 2V89, 2V8A, 2V8B, 2V8C, 2V8D, 2V8E, 2V8F, 2V8G, 2V8H, 2V8I, 2V8J, 2V8K, 2V8L, 2V8M, 2V8N, 2V8O, 2V8P, 2V8Q, 2V8S, 2V8T, 2V8U, 2V8V, 2V8W, 2V8X, 2V8Y, 2V8Z, 2V90, 2V91, 2V92, 2V94, 2V95, 2V96, 2V97, 2V98, 2V9A, 2V9B, 2V9C, 2V9D, 2V9E, 2V9F, 2V9G, 2V9I, 2V9J, 2V9K, 2V9L, 2V9M, 2V9N, 2V9O, 2V9P, 2V9Q, 2V9R, 2V9S, 2V9T, 2V9U, 2V9V, 2V9W, 2V9X, 2V9Y, 2V9Z, 2VA0, 2VA1, 2VA2, 2VA3, 2VA5, 2VA6, 2VA7, 2VA8, 2VA9, 2VAA, 2VAB, 2VAC, 2VAD, 2VAE, 2VAF, 2VAG, 2VAJ, 2VAK, 2VAM, 2VAN, 2VAO, 2VAP, 2VAQ, 2VAR, 2VAS, 2VAT, 2VAU, 2VAV, 2VAW, 2VAX, 2VAY, 2VB0, 2VB1, 2VB2, 2VB3, 2VB6, 2VB7, 2VB8, 2VB9, 2VBA, 2VBB, 2VBC, 2VBD, 2VBE, 2VBF, 2VBG, 2VBI, 2VBJ, 2VBK, 2VBL, 2VBM, 2VBN, 2VBO, 2VBP, 2VBQ, 2VBS, 2VBT, 2VBU, 2VBV, 2VBW, 2VBX, 2VBY, 2VBZ, 2VC0, 2VC1, 2VC2, 2VC3, 2VC4, 2VC5, 2VC6, 2VC7, 2VC8, 2VC9, 2VCA, 2VCB, 2VCC, 2VCE, 2VCF, 2VCG, 2VCH, 2VCI, 2VCJ, 2VCK, 2VCL, 2VCM, 2VCN, 2VCO, 2VCP, 2VCQ, 2VCS, 2VCT, 2VCV, 2VCW, 2VCX, 2VCY, 2VCZ, 2VD0, 2VD1, 2VD2, 2VD3, 2VD4, 2VD5, 2VD6, 2VD7, 2VD8, 2VD9, 2VDB, 2VDD, 2VDE, 2VDF, 2VDG, 2VDH, 2VDI, 2VDJ, 2VDK, 2VDL, 2VDM, 2VDN, 2VDO, 2VDP, 2VDQ, 2VDR, 2VDT, 2VDU, 2VDV, 2VDW, 2VDX, 2VDY, 2VE1, 2VE3, 2VE4, 2VE6, 2VE7, 2VE8, 2VE9, 2VEA, 2VEB, 2VEC, 2VED, 2VEE, 2VEF, 2VEG, 2VEI, 2VEK, 2VEL, 2VEM, 2VEN, 2VEO, 2VEP, 2VEQ, 2VES, 2VET, 2VEU, 2VEV, 2VEW, 2VEX, 2VEY, 2VEZ, 2VF0, 2VF2, 2VF3, 2VF4, 2VF5, 2VF6, 2VF7, 2VF8, 2VF9, 2VFA, 2VFB, 2VFC, 2VFD, 2VFE, 2VFF, 2VFG, 2VFH, 2VFI, 2VFJ, 2VFK, 2VFL, 2VFM, 2VFN, 2VFO, 2VFP, 2VFQ, 2VFR, 2VFS, 2VFT, 2VFU, 2VFV, 2VFW, 2VFX, 2VFY, 2VFZ, 2VG0, 2VG1, 2VG2, 2VG3, 2VG4, 2VG5, 2VG6, 2VG7, 2VG8, 2VG9, 2VGA, 2VGB, 2VGC, 2VGD, 2VGE, 2VGF, 2VGG, 2VGI, 2VGJ, 2VGK, 2VGL, 2VGM, 2VGN, 2VGO, 2VGP, 2VGQ, 2VGR, 2VGS, 2VGT, 2VGU, 2VGV, 2VGW, 2VGX, 2VGY, 2VGZ, 2VH0, 2VH1, 2VH2, 2VH3, 2VH4, 2VH5, 2VH6, 2VH7, 2VH9, 2VHA, 2VHB, 2VHC, 2VHD, 2VHE, 2VHF, 2VHG, 2VHH, 2VHI, 2VHJ, 2VHK, 2VHL, 2VHQ, 2VHR, 2VHS, 2VHT, 2VHU, 2VHV, 2VHW, 2VHX, 2VHY, 2VHZ, 2VI0, 2VI1, 2VI2, 2VI3, 2VI4, 2VI5, 2VI6, 2VI7, 2VI8, 2VI9, 2VIA, 2VIB, 2VIC, 2VID, 2VIE, 2VIF, 2VIG, 2VIH, 2VII, 2VIJ, 2VIM, 2VIN, 2VIO, 2VIP, 2VIQ, 2VIR, 2VIS, 2VIT, 2VIU, 2VIV, 2VIW, 2VIX, 2VIY, 2VIZ, 2VJ0, 2VJ1, 2VJ2, 2VJ3, 2VJ4, 2VJ5, 2VJ6, 2VJ7, 2VJ8, 2VJ9, 2VJA, 2VJB, 2VJC, 2VJD, 2VJE, 2VJF, 2VJH, 2VJI, 2VJJ, 2VJK, 2VJL, 2VJM, 2VJN, 2VJO, 2VJP, 2VJQ, 2VJR, 2VJT, 2VJU, 2VJV, 2VJW, 2VJX, 2VJY, 2VJZ, 2VK0, 2VK1, 2VK2, 2VK3, 2VK4, 2VK5, 2VK6, 2VK7, 2VK8, 2VK9, 2VKA, 2VKD, 2VKE, 2VKF, 2VKG, 2VKH, 2VKI, 2VKJ, 2VKL, 2VKM, 2VKN, 2VKO, 2VKP, 2VKQ, 2VKR, 2VKS, 2VKT, 2VKU, 2VKV, 2VKW, 2VKX, 2VKY, 2VKZ, 2VL0, 2VL1, 2VL2, 2VL3, 2VL4, 2VL5, 2VL6, 2VL7, 2VL8, 2VL9, 2VLA, 2VLB, 2VLC, 2VLD, 2VLE, 2VLF, 2VLG, 2VLH, 2VLI, 2VLJ, 2VLK, 2VLL, 2VLM, 2VLN, 2VLO, 2VLP, 2VLQ, 2VLR, 2VLT, 2VLU, 2VLV, 2VLW, 2VLX, 2VLY, 2VLZ, 2VM0, 2VM1, 2VM2, 2VM3, 2VM4, 2VM5, 2VM6, 2VM8, 2VM9, 2VMA, 2VMB, 2VMC, 2VMD, 2VME, 2VMF, 2VMG, 2VMH, 2VMI, 2VMJ, 2VMK, 2VML, 2VMN, 2VMO, 2VMP, 2VMQ, 2VMR, 2VMS, 2VMT, 2VMU, 2VMV, 2VMW, 2VMX, 2VMY, 2VMZ, 2VN0, 2VN1, 2VN2, 2VN3, 2VN4, 2VN5, 2VN6, 2VN7, 2VN8, 2VN9, 2VNA, 2VNC, 2VND, 2VNE, 2VNF, 2VNG, 2VNH, 2VNI, 2VNJ, 2VNK, 2VNL, 2VNM, 2VNN, 2VNO, 2VNP, 2VNQ, 2VNR, 2VNS, 2VNT, 2VNV, 2VNW, 2VNX, 2VNY, 2VNZ, 2VO0, 2VO1, 2VO2, 2VO3, 2VO4, 2VO5, 2VO6, 2VO7, 2VO8, 2VO9, 2VOA, 2VOB, 2VOC, 2VOE, 2VOF, 2VOG, 2VOH, 2VOI, 2VOJ, 2VOK, 2VOM, 2VOR, 2VOS, 2VOT, 2VOU, 2VOV, 2VOW, 2VOX, 2VOZ, 2VP0, 2VP1, 2VP2, 2VP3, 2VP4, 2VP5, 2VP6, 2VP7, 2VP8, 2VP9, 2VPA, 2VPB, 2VPD, 2VPE, 2VPF, 2VPG, 2VPH, 2VPI, 2VPJ, 2VPK, 2VPM, 2VPN, 2VPO, 2VPP, 2VPQ, 2VPR, 2VPS, 2VPT, 2VPV, 2VPW, 2VPX, 2VPY, 2VPZ, 2VQ1, 2VQ2, 2VQ3, 2VQ4, 2VQ5, 2VQ6, 2VQ7, 2VQ8, 2VQ9, 2VQA, 2VQB, 2VQC, 2VQD, 2VQG, 2VQH, 2VQI, 2VQJ, 2VQK, 2VQL, 2VQM, 2VQO, 2VQP, 2VQQ, 2VQR, 2VQS, 2VQT, 2VQU, 2VQV, 2VQW, 2VQX, 2VQY, 2VQZ, 2VR0, 2VR1, 2VR2, 2VR3, 2VR4, 2VR5, 2VR6, 2VR7, 2VR8, 2VR9, 2VRA, 2VRB, 2VRC, 2VRE, 2VRF, 2VRI, 2VRJ, 2VRK, 2VRL, 2VRM, 2VRN, 2VRO, 2VRP, 2VRQ, 2VRR, 2VRS, 2VRW, 2VRX, 2VRY, 2VRZ, 2VS0, 2VS1, 2VS3, 2VS4, 2VS5, 2VS6, 2VS7, 2VS8, 2VSA, 2VSC, 2VSD, 2VSE, 2VSF, 2VSG, 2VSH, 2VSI, 2VSK, 2VSL, 2VSM, 2VSN, 2VSO, 2VSP, 2VSQ, 2VSR, 2VSS, 2VST, 2VSU, 2VSV, 2VSW, 2VSX, 2VSY, 2VSZ, 2VT0, 2VT1, 2VT2, 2VT3, 2VT4, 2VT5, 2VT6, 2VT7, 2VT8, 2VTA, 2VTB, 2VTC, 2VTD, 2VTE, 2VTF, 2VTG, 2VTH, 2VTI, 2VTJ, 2VTK, 2VTL, 2VTM, 2VTN, 2VTO, 2VTP, 2VTQ, 2VTR, 2VTS, 2VTT, 2VTU, 2VTV, 2VTW, 2VTX, 2VTY, 2VTZ, 2VU0, 2VU1, 2VU2, 2VU3, 2VU4, 2VU5, 2VU6, 2VU7, 2VU8, 2VU9, 2VUA, 2VUB, 2VUC, 2VUD, 2VUE, 2VUF, 2VUG, 2VUH, 2VUI, 2VUJ, 2VUK, 2VUL, 2VUN, 2VUO, 2VUP, 2VUR, 2VUS, 2VUT, 2VUU, 2VUV, 2VUW, 2VUX, 2VUY, 2VUZ, 2VV0, 2VV1, 2VV2, 2VV3, 2VV4, 2VV5, 2VV6, 2VV7, 2VV8, 2VV9, 2VVA, 2VVB, 2VVC, 2VVD, 2VVE, 2VVF, 2VVG, 2VVH, 2VVI, 2VVJ, 2VVK, 2VVL, 2VVM, 2VVN, 2VVO, 2VVP, 2VVQ, 2VVR, 2VVS, 2VVT, 2VVU, 2VVV, 2VVW, 2VVX, 2VVY, 2VVZ, 2VW0, 2VW1, 2VW2, 2VW4, 2VW5, 2VW6, 2VW7, 2VW8, 2VW9, 2VWA, 2VWB, 2VWC, 2VWD, 2VWE, 2VWF, 2VWG, 2VWH, 2VWI, 2VWJ, 2VWK, 2VWL, 2VWM, 2VWN, 2VWO, 2VWP, 2VWQ, 2VWR, 2VWS, 2VWT, 2VWU, 2VWV, 2VWW, 2VWX, 2VWY, 2VWZ, 2VX0, 2VX1, 2VX2, 2VX3, 2VX4, 2VX5, 2VX6, 2VX7, 2VX8, 2VX9, 2VXA, 2VXB, 2VXC, 2VXG, 2VXH, 2VXI, 2VXJ, 2VXK, 2VXL, 2VXM, 2VXN, 2VXO, 2VXP, 2VXQ, 2VXR, 2VXS, 2VXT, 2VXU, 2VXV, 2VXW, 2VXX, 2VXY, 2VXZ, 2VY0, 2VY1, 2VY2, 2VY3, 2VY6, 2VY7, 2VY8, 2VY9, 2VYA, 2VYC, 2VYE, 2VYF, 2VYI, 2VYN, 2VYO, 2VYP, 2VYQ, 2VYR, 2VYT, 2VYU, 2VYV, 2VYW, 2VYX, 2VYY, 2VYZ, 2VZ0, 2VZ1, 2VZ2, 2VZ3, 2VZ4, 2VZ6, 2VZ7, 2VZ8, 2VZ9, 2VZA, 2VZB, 2VZC, 2VZD, 2VZE, 2VZG, 2VZI, 2VZK, 2VZL, 2VZM, 2VZN, 2VZO, 2VZP, 2VZQ, 2VZR, 2VZS, 2VZT, 2VZU, 2VZV, 2VZW, 2VZX, 2VZY, 2VZZ, 2W00, 2W01, 2W02, 2W03, 2W04, 2W05, 2W06, 2W07, 2W08, 2W09, 2W0A, 2W0B, 2W0D, 2W0F, 2W0G, 2W0H, 2W0I, 2W0J, 2W0K, 2W0L, 2W0M, 2W0O, 2W0P, 2W0Q, 2W0R, 2W0S, 2W0U, 2W0V, 2W0W, 2W0X, 2W0Z, 2W10, 2W11, 2W12, 2W13, 2W14, 2W15, 2W16, 2W17, 2W18, 2W19, 2W1A, 2W1B, 2W1C, 2W1D, 2W1E, 2W1F, 2W1G, 2W1H, 2W1I, 2W1J, 2W1K, 2W1L, 2W1M, 2W1N, 2W1P, 2W1Q, 2W1R, 2W1S, 2W1T, 2W1U, 2W1V, 2W1W, 2W1X, 2W1Y, 2W1Z, 2W20, 2W21, 2W22, 2W23, 2W24, 2W25, 2W26, 2W27, 2W29, 2W2A, 2W2B, 2W2C, 2W2D, 2W2E, 2W2F, 2W2G, 2W2I, 2W2J, 2W2K, 2W2L, 2W2M, 2W2N, 2W2O, 2W2P, 2W2Q, 2W2R, 2W2S, 2W2T, 2W2U, 2W2V, 2W2W, 2W2X, 2W31, 2W35, 2W36, 2W37, 2W38, 2W39, 2W3A, 2W3B, 2W3C, 2W3D, 2W3E, 2W3F, 2W3G, 2W3H, 2W3I, 2W3J, 2W3K, 2W3L, 2W3M, 2W3N, 2W3O, 2W3P, 2W3Q, 2W3R, 2W3S, 2W3T, 2W3U, 2W3V, 2W3W, 2W3X, 2W3Y, 2W3Z, 2W40, 2W41, 2W42, 2W43, 2W44, 2W45, 2W46, 2W47, 2W48, 2W4B, 2W4C, 2W4D, 2W4E, 2W4F, 2W4I, 2W4J, 2W4K, 2W4L, 2W4M, 2W4O, 2W4P, 2W4Q, 2W4R, 2W4S, 2W4X, 2W4Y, 2W4Z, 2W50, 2W51, 2W52, 2W53, 2W54, 2W55, 2W56, 2W57, 2W58, 2W59, 2W5A, 2W5B, 2W5E, 2W5F, 2W5G, 2W5H, 2W5I, 2W5J, 2W5K, 2W5L, 2W5M, 2W5N, 2W5O, 2W5P, 2W5Q, 2W5R, 2W5S, 2W5T, 2W5U, 2W5V, 2W5W, 2W5X, 2W5Y, 2W5Z, 2W60, 2W61, 2W62, 2W63, 2W65, 2W66, 2W67, 2W68, 2W69, 2W6A, 2W6B, 2W6C, 2W6E, 2W6F, 2W6G, 2W6H, 2W6I, 2W6J, 2W6K, 2W6L, 2W6M, 2W6N, 2W6O, 2W6P, 2W6Q, 2W6R, 2W6T, 2W6U, 2W6V, 2W6W, 2W6X, 2W6Y, 2W6Z, 2W70, 2W71, 2W72, 2W73, 2W75, 2W76, 2W77, 2W78, 2W79, 2W7A, 2W7D, 2W7E, 2W7F, 2W7G, 2W7H, 2W7I, 2W7J, 2W7K, 2W7L, 2W7M, 2W7N, 2W7O, 2W7P, 2W7Q, 2W7R, 2W7S, 2W7T, 2W7U, 2W7V, 2W7W, 2W7X, 2W7Y, 2W7Z, 2W80, 2W81, 2W82, 2W83, 2W86, 2W87, 2W88, 2W8B, 2W8C, 2W8D, 2W8F, 2W8G, 2W8H, 2W8I, 2W8J, 2W8K, 2W8L, 2W8M, 2W8N, 2W8O, 2W8P, 2W8Q, 2W8R, 2W8S, 2W8T, 2W8U, 2W8V, 2W8W, 2W8X, 2W8Y, 2W8Z, 2W90, 2W91, 2W92, 2W93, 2W94, 2W95, 2W96, 2W97, 2W98, 2W99, 2W9A, 2W9B, 2W9C, 2W9D, 2W9E, 2W9F, 2W9G, 2W9H, 2W9I, 2W9J, 2W9L, 2W9M, 2W9N, 2W9P, 2W9Q, 2W9R, 2W9S, 2W9T, 2W9X, 2W9Y, 2W9Z, 2WA0, 2WA1, 2WA2, 2WA3, 2WA4, 2WA5, 2WA6, 2WA7, 2WA8, 2WA9, 2WAA, 2WAB, 2WAC, 2WAD, 2WAE, 2WAF, 2WAG, 2WAH, 2WAJ, 2WAL, 2WAM, 2WAN, 2WAO, 2WAP, 2WAQ, 2WAR, 2WAS, 2WAT, 2WAU, 2WAW, 2WAX, 2WAY, 2WAZ, 2WB0, 2WB1, 2WB2, 2WB3, 2WB4, 2WB5, 2WB6, 2WB7, 2WB8, 2WB9, 2WBA, 2WBB, 2WBC, 2WBD, 2WBF, 2WBG, 2WBH, 2WBI, 2WBJ, 2WBK, 2WBL, 2WBM, 2WBN, 2WBO, 2WBP, 2WBQ, 2WBS, 2WBT, 2WBU, 2WBV, 2WBW, 2WBX, 2WBY, 2WBZ, 2WC0, 2WC1, 2WC3, 2WC4, 2WC5, 2WC6, 2WC7, 2WC8, 2WC9, 2WCA, 2WCB, 2WCD, 2WCE, 2WCF, 2WCG, 2WCH, 2WCI, 2WCJ, 2WCK, 2WCL, 2WCM, 2WCO, 2WCP, 2WCQ, 2WCR, 2WCS, 2WCT, 2WCU, 2WCV, 2WCW, 2WCX, 2WCZ, 2WD0, 2WD1, 2WD2, 2WD3, 2WD4, 2WD5, 2WD6, 2WD7, 2WD8, 2WD9, 2WDA, 2WDB, 2WDC, 2WDD, 2WDE, 2WDF, 2WDO, 2WDP, 2WDQ, 2WDR, 2WDS, 2WDT, 2WDU, 2WDV, 2WDW, 2WDY, 2WDZ, 2WE0, 2WE1, 2WE2, 2WE3, 2WE4, 2WE5, 2WE6, 2WE7, 2WE8, 2WE9, 2WEA, 2WEB, 2WEC, 2WED, 2WEE, 2WEF, 2WEG, 2WEH, 2WEI, 2WEJ, 2WEK, 2WEL, 2WEO, 2WEP, 2WEQ, 2WER, 2WES, 2WET, 2WEU, 2WEV, 2WEW, 2WEX, 2WEY, 2WEZ, 2WF0, 2WF1, 2WF2, 2WF3, 2WF4, 2WF5, 2WF6, 2WF7, 2WF8, 2WF9, 2WFA, 2WFB, 2WFC, 2WFD, 2WFE, 2WFG, 2WFH, 2WFI, 2WFJ, 2WFK, 2WFL, 2WFM, 2WFN, 2WFO, 2WFP, 2WFQ, 2WFR, 2WFT, 2WFU, 2WFV, 2WFW, 2WFX, 2WFY, 2WFZ, 2WG0, 2WG1, 2WG2, 2WG3, 2WG4, 2WG5, 2WG6, 2WG7, 2WG8, 2WG9, 2WGB, 2WGC, 2WGD, 2WGE, 2WGF, 2WGG, 2WGH, 2WGI, 2WGJ, 2WGK, 2WGL, 2WGM, 2WGP, 2WGQ, 2WGR, 2WGS, 2WGT, 2WGU, 2WGV, 2WGW, 2WGX, 2WGY, 2WGZ, 2WH0, 2WH5, 2WH6, 2WH7, 2WH8, 2WHB, 2WHD, 2WHE, 2WHF, 2WHG, 2WHH, 2WHI, 2WHJ, 2WHK, 2WHL, 2WHM, 2WHN, 2WHO, 2WHP, 2WHQ, 2WHR, 2WHS, 2WHT, 2WHU, 2WHV, 2WHW, 2WHX, 2WHY, 2WHZ, 2WI0, 2WI1, 2WI2, 2WI3, 2WI4, 2WI5, 2WI6, 2WI7, 2WI8, 2WI9, 2WIA, 2WIB, 2WIC, 2WID, 2WIE, 2WIF, 2WIG, 2WIH, 2WII, 2WIJ, 2WIK, 2WIL, 2WIM, 2WIN, 2WIO, 2WIP, 2WIQ, 2WIS, 2WIT, 2WIU, 2WIV, 2WIW, 2WIY, 2WIZ, 2WJ0, 2WJ1, 2WJ2, 2WJ3, 2WJ4, 2WJ5, 2WJ6, 2WJ7, 2WJ9, 2WJA, 2WJD, 2WJE, 2WJF, 2WJG, 2WJH, 2WJI, 2WJJ, 2WJK, 2WJL, 2WJM, 2WJN, 2WJO, 2WJP, 2WJQ, 2WJR, 2WJS, 2WJU, 2WJV, 2WJW, 2WJX, 2WJY, 2WJZ, 2WK0, 2WK1, 2WK2, 2WK3, 2WK4, 2WK5, 2WK6, 2WK7, 2WK8, 2WK9, 2WKA, 2WKB, 2WKC, 2WKD, 2WKE, 2WKF, 2WKG, 2WKH, 2WKI, 2WKJ, 2WKK, 2WKL, 2WKM, 2WKN, 2WKO, 2WKP, 2WKQ, 2WKR, 2WKS, 2WKT, 2WKU, 2WKV, 2WKW, 2WKX, 2WKY, 2WKZ, 2WL0, 2WL1, 2WL3, 2WL4, 2WL5, 2WL6, 2WL7, 2WL8, 2WL9, 2WLA, 2WLB, 2WLC, 2WLD, 2WLE, 2WLF, 2WLG, 2WLH, 2WLI, 2WLJ, 2WLK, 2WLL, 2WLM, 2WLN, 2WLO, 2WLP, 2WLQ, 2WLR, 2WLS, 2WLT, 2WLU, 2WLV, 2WLW, 2WLX, 2WLY, 2WLZ, 2WM0, 2WM1, 2WM2, 2WM3, 2WM4, 2WM5, 2WM8, 2WM9, 2WMA, 2WMB, 2WMC, 2WMD, 2WME, 2WMF, 2WMG, 2WMH, 2WMI, 2WMJ, 2WMK, 2WML, 2WMM, 2WMN, 2WMO, 2WMP, 2WMQ, 2WMR, 2WMS, 2WMT, 2WMU, 2WMV, 2WMW, 2WMX, 2WMY, 2WMZ, 2WN2, 2WN3, 2WN4, 2WN5, 2WN6, 2WN7, 2WN8, 2WN9, 2WNB, 2WNC, 2WND, 2WNE, 2WNF, 2WNG, 2WNH, 2WNI, 2WNJ, 2WNK, 2WNL, 2WNN, 2WNO, 2WNP, 2WNQ, 2WNR, 2WNS, 2WNT, 2WNU, 2WNV, 2WNW, 2WNX, 2WNY, 2WNZ, 2WO0, 2WO1, 2WO2, 2WO3, 2WO4, 2WO5, 2WO6, 2WO7, 2WO8, 2WO9, 2WOA, 2WOB, 2WOC, 2WOD, 2WOE, 2WOF, 2WOG, 2WOH, 2WOI, 2WOJ, 2WOK, 2WOL, 2WOM, 2WON, 2WOO, 2WOP, 2WOQ, 2WOR, 2WOS, 2WOT, 2WOU, 2WOV, 2WOW, 2WOX, 2WOY, 2WOZ, 2WP0, 2WP1, 2WP2, 2WP3, 2WP4, 2WP5, 2WP6, 2WP7, 2WP8, 2WP9, 2WPA, 2WPB, 2WPC, 2WPD, 2WPE, 2WPF, 2WPG, 2WPH, 2WPI, 2WPJ, 2WPK, 2WPL, 2WPM, 2WPN, 2WPO, 2WPQ, 2WPR, 2WPS, 2WPT, 2WPU, 2WPV, 2WPW, 2WPX, 2WPY, 2WPZ, 2WQ0, 2WQ1, 2WQ2, 2WQ3, 2WQ4, 2WQ5, 2WQ6, 2WQ7, 2WQ8, 2WQ9, 2WQA, 2WQB, 2WQD, 2WQE, 2WQF, 2WQH, 2WQI, 2WQJ, 2WQK, 2WQL, 2WQM, 2WQN, 2WQO, 2WQP, 2WQQ, 2WQR, 2WQS, 2WQT, 2WQU, 2WQV, 2WQW, 2WQX, 2WQY, 2WQZ, 2WR0, 2WR1, 2WR2, 2WR3, 2WR4, 2WR5, 2WR6, 2WR7, 2WR8, 2WR9, 2WRA, 2WRB, 2WRC, 2WRD, 2WRE, 2WRF, 2WRG, 2WRH, 2WRM, 2WRS, 2WRT, 2WRU, 2WRV, 2WRW, 2WRX, 2WRY, 2WRZ, 2WS0, 2WS1, 2WS2, 2WS3, 2WS4, 2WS6, 2WS7, 2WSA, 2WSB, 2WSD, 2WSH, 2WSI, 2WSJ, 2WSK, 2WSL, 2WSM, 2WSN, 2WSO, 2WSP, 2WSQ, 2WSR, 2WSS, 2WST, 2WSU, 2WSV, 2WSW, 2WSX, 2WSY, 2WT0, 2WT1, 2WT2, 2WT4, 2WT7, 2WT8, 2WT9, 2WTA, 2WTB, 2WTC, 2WTD, 2WTE, 2WTF, 2WTG, 2WTH, 2WTI, 2WTJ, 2WTK, 2WTL, 2WTM, 2WTN, 2WTO, 2WTP, 2WTR, 2WTS, 2WTT, 2WTU, 2WTV, 2WTW, 2WTX, 2WTY, 2WTZ, 2WU0, 2WU1, 2WU2, 2WU3, 2WU4, 2WU5, 2WU6, 2WU7, 2WU8, 2WU9, 2WUA, 2WUB, 2WUC, 2WUD, 2WUE, 2WUF, 2WUG, 2WUH, 2WUI, 2WUJ, 2WUK, 2WUL, 2WUQ, 2WUR, 2WUS, 2WUT, 2WUU, 2WUV, 2WUW, 2WUX, 2WUY, 2WUZ, 2WV0, 2WV1, 2WV2, 2WV3, 2WV4, 2WV5, 2WV6, 2WV7, 2WV8, 2WV9, 2WVA, 2WVB, 2WVC, 2WVD, 2WVE, 2WVF, 2WVG, 2WVH, 2WVI, 2WVJ, 2WVK, 2WVL, 2WVM, 2WVN, 2WVO, 2WVP, 2WVQ, 2WVR, 2WVS, 2WVT, 2WVU, 2WVV, 2WVX, 2WVY, 2WVZ, 2WW0, 2WW1, 2WW2, 2WW3, 2WW4, 2WW5, 2WW6, 2WW7, 2WW8, 2WWC, 2WWD, 2WWE, 2WWF, 2WWG, 2WWH, 2WWI, 2WWJ, 2WWK, 2WWM, 2WWN, 2WWO, 2WWP, 2WWR, 2WWT, 2WWU, 2WWW, 2WWX, 2WWZ, 2WX0, 2WX1, 2WX2, 2WX3, 2WX4, 2WX5, 2WX9, 2WXB, 2WXD, 2WXF, 2WXG, 2WXH, 2WXI, 2WXJ, 2WXK, 2WXL, 2WXM, 2WXN, 2WXO, 2WXP, 2WXQ, 2WXR, 2WXT, 2WXU, 2WXV, 2WXW, 2WXX, 2WXY, 2WXZ, 2WY0, 2WY1, 2WY3, 2WY4, 2WY6, 2WY7, 2WY8, 2WYA, 2WYB, 2WYC, 2WYD, 2WYE, 2WYF, 2WYG, 2WYH, 2WYI, 2WYJ, 2WYK, 2WYL, 2WYM, 2WYN, 2WYO, 2WYP, 2WYQ, 2WYR, 2WYS, 2WYT, 2WYU, 2WYV, 2WYW, 2WYZ, 2WZ0, 2WZ1, 2WZ5, 2WZ6, 2WZ7, 2WZ8, 2WZ9, 2WZA, 2WZB, 2WZC, 2WZD, 2WZE, 2WZF, 2WZG, 2WZH, 2WZI, 2WZJ, 2WZK, 2WZL, 2WZM, 2WZN, 2WZO, 2WZP, 2WZQ, 2WZS, 2WZT, 2WZV, 2WZW, 2WZX, 2WZY, 2WZZ, 2X00, 2X01, 2X02, 2X03, 2X04, 2X05, 2X06, 2X07, 2X08, 2X09, 2X0A, 2X0B, 2X0C, 2X0D, 2X0E, 2X0F, 2X0G, 2X0H, 2X0I, 2X0J, 2X0K, 2X0L, 2X0N, 2X0O, 2X0P, 2X0Q, 2X0R, 2X0S, 2X0U, 2X0V, 2X0W, 2X0X, 2X0Y, 2X10, 2X11, 2X12, 2X13, 2X14, 2X15, 2X16, 2X17, 2X18, 2X19, 2X1B, 2X1C, 2X1D, 2X1E, 2X1G, 2X1H, 2X1I, 2X1J, 2X1K, 2X1L, 2X1M, 2X1N, 2X1O, 2X1P, 2X1Q, 2X1R, 2X1S, 2X1T, 2X1U, 2X1V, 2X1W, 2X1X, 2X22, 2X23, 2X24, 2X25, 2X26, 2X27, 2X28, 2X29, 2X2A, 2X2B, 2X2C, 2X2D, 2X2E, 2X2F, 2X2G, 2X2H, 2X2I, 2X2J, 2X2K, 2X2L, 2X2M, 2X2N, 2X2O, 2X2P, 2X2R, 2X2S, 2X2T, 2X2U, 2X2V, 2X2W, 2X2Y, 2X2Z, 2X30, 2X32, 2X34, 2X35, 2X36, 2X38, 2X39, 2X3A, 2X3B, 2X3C, 2X3D, 2X3E, 2X3F, 2X3G, 2X3H, 2X3J, 2X3K, 2X3L, 2X3M, 2X3N, 2X3O, 2X3T, 2X3U, 2X3V, 2X3W, 2X3X, 2X3Y, 2X40, 2X41, 2X42, 2X44, 2X45, 2X46, 2X47, 2X48, 2X49, 2X4A, 2X4D, 2X4F, 2X4G, 2X4H, 2X4I, 2X4J, 2X4K, 2X4L, 2X4M, 2X4N, 2X4O, 2X4P, 2X4Q, 2X4R, 2X4S, 2X4T, 2X4U, 2X4W, 2X4X, 2X4Y, 2X4Z, 2X50, 2X51, 2X52, 2X53, 2X54, 2X55, 2X56, 2X57, 2X58, 2X5A, 2X5C, 2X5D, 2X5F, 2X5G, 2X5H, 2X5J, 2X5K, 2X5L, 2X5N, 2X5O, 2X5P, 2X5Q, 2X5R, 2X5S, 2X5T, 2X5U, 2X5V, 2X5W, 2X5X, 2X5Y, 2X5Z, 2X60, 2X61, 2X62, 2X63, 2X64, 2X65, 2X66, 2X67, 2X68, 2X69, 2X6A, 2X6B, 2X6C, 2X6D, 2X6E, 2X6F, 2X6G, 2X6H, 2X6I, 2X6J, 2X6K, 2X6L, 2X6M, 2X6N, 2X6O, 2X6P, 2X6Q, 2X6R, 2X6S, 2X6T, 2X6U, 2X6V, 2X6W, 2X6X, 2X6Y, 2X70, 2X71, 2X72, 2X74, 2X75, 2X76, 2X77, 2X78, 2X79, 2X7A, 2X7B, 2X7C, 2X7D, 2X7E, 2X7F, 2X7G, 2X7H, 2X7I, 2X7J, 2X7K, 2X7L, 2X7M, 2X7O, 2X7P, 2X7Q, 2X7R, 2X7S, 2X7T, 2X7U, 2X7V, 2X7W, 2X7X, 2X7Y, 2X7Z, 2X80, 2X81, 2X82, 2X83, 2X85, 2X86, 2X87, 2X88, 2X89, 2X8A, 2X8B, 2X8C, 2X8D, 2X8E, 2X8F, 2X8G, 2X8H, 2X8I, 2X8J, 2X8K, 2X8L, 2X8M, 2X8O, 2X8P, 2X8R, 2X8S, 2X8T, 2X8U, 2X8W, 2X8X, 2X8Y, 2X8Z, 2X90, 2X91, 2X92, 2X93, 2X94, 2X95, 2X96, 2X97, 2X98, 2X99, 2X9A, 2X9B, 2X9C, 2X9D, 2X9E, 2X9F, 2X9G, 2X9H, 2X9I, 2X9J, 2X9K, 2X9L, 2X9M, 2X9N, 2X9O, 2X9P, 2X9Q, 2X9V, 2X9W, 2X9X, 2X9Y, 2X9Z, 2XA0, 2XA1, 2XA2, 2XA3, 2XA4, 2XA5, 2XA7, 2XA8, 2XA9, 2XAA, 2XAB, 2XAC, 2XAD, 2XAE, 2XAF, 2XAG, 2XAH, 2XAJ, 2XAK, 2XAL, 2XAM, 2XAN, 2XAO, 2XAP, 2XAQ, 2XAR, 2XAS, 2XAT, 2XAU, 2XAV, 2XAW, 2XAX, 2XAY, 2XAZ, 2XB0, 2XB1, 2XB3, 2XB4, 2XB5, 2XB6, 2XB7, 2XB8, 2XB9, 2XBA, 2XBB, 2XBF, 2XBG, 2XBI, 2XBJ, 2XBK, 2XBL, 2XBN, 2XBP, 2XBQ, 2XBR, 2XBS, 2XBT, 2XBU, 2XBV, 2XBW, 2XBX, 2XBY, 2XBZ, 2XC0, 2XC1, 2XC2, 2XC3, 2XC4, 2XC5, 2XC8, 2XC9, 2XCA, 2XCB, 2XCC, 2XCD, 2XCE, 2XCF, 2XCG, 2XCH, 2XCI, 2XCJ, 2XCK, 2XCL, 2XCM, 2XCN, 2XCO, 2XCP, 2XCQ, 2XCR, 2XCS, 2XCT, 2XCU, 2XCV, 2XCW, 2XCX, 2XCY, 2XCZ, 2XD1, 2XD2, 2XD3, 2XD4, 2XD5, 2XD6, 2XD7, 2XD9, 2XDA, 2XDE, 2XDG, 2XDH, 2XDJ, 2XDK, 2XDL, 2XDM, 2XDN, 2XDO, 2XDP, 2XDQ, 2XDR, 2XDS, 2XDU, 2XDV, 2XDW, 2XDX, 2XDY, 2XE0, 2XE1, 2XE2, 2XE3, 2XE4, 2XE5, 2XE6, 2XE7, 2XE8, 2XEC, 2XED, 2XEE, 2XEF, 2XEG, 2XEH, 2XEI, 2XEJ, 2XEL, 2XEM, 2XEN, 2XEP, 2XEQ, 2XER, 2XES, 2XET, 2XEU, 2XEV, 2XEW, 2XEX, 2XEY, 2XEZ, 2XF0, 2XF1, 2XF2, 2XF3, 2XF4, 2XF5, 2XF6, 2XF7, 2XF8, 2XFA, 2XFD, 2XFE, 2XFF, 2XFG, 2XFH, 2XFI, 2XFJ, 2XFK, 2XFL, 2XFN, 2XFO, 2XFP, 2XFQ, 2XFR, 2XFS, 2XFT, 2XFU, 2XFV, 2XFW, 2XFX, 2XFY, 2XG3, 2XG4, 2XG5, 2XG6, 2XG7, 2XG8, 2XG9, 2XGA, 2XGB, 2XGC, 2XGD, 2XGE, 2XGF, 2XGG, 2XGI, 2XGL, 2XGM, 2XGN, 2XGO, 2XGP, 2XGQ, 2XGR, 2XGS, 2XGT, 2XGU, 2XGV, 2XGW, 2XGX, 2XGY, 2XGZ, 2XH0, 2XH1, 2XH2, 2XH3, 2XH4, 2XH5, 2XH6, 2XH7, 2XH8, 2XH9, 2XHA, 2XHB, 2XHC, 2XHD, 2XHE, 2XHF, 2XHG, 2XHH, 2XHI, 2XHJ, 2XHK, 2XHL, 2XHM, 2XHN, 2XHR, 2XHS, 2XHT, 2XHU, 2XHV, 2XHW, 2XHX, 2XHY, 2XHZ, 2XI1, 2XI2, 2XI3, 2XI4, 2XI5, 2XI6, 2XI7, 2XI8, 2XI9, 2XIB, 2XIC, 2XID, 2XIF, 2XIG, 2XIH, 2XII, 2XIJ, 2XIK, 2XIL, 2XIM, 2XIN, 2XIO, 2XIQ, 2XIR, 2XIS, 2XIT, 2XIU, 2XIV, 2XIW, 2XIX, 2XIY, 2XIZ, 2XJ0, 2XJ1, 2XJ2, 2XJ3, 2XJ4, 2XJ5, 2XJ6, 2XJ7, 2XJ8, 2XJ9, 2XJA, 2XJB, 2XJC, 2XJD, 2XJE, 2XJF, 2XJG, 2XJJ, 2XJK, 2XJL, 2XJM, 2XJN, 2XJO, 2XJP, 2XJQ, 2XJR, 2XJS, 2XJT, 2XJU, 2XJV, 2XJW, 2XJX, 2XJY, 2XJZ, 2XK1, 2XK2, 2XK3, 2XK4, 2XK5, 2XK6, 2XK7, 2XK8, 2XK9, 2XKA, 2XKB, 2XKC, 2XKD, 2XKE, 2XKF, 2XKG, 2XKH, 2XKI, 2XKJ, 2XKK, 2XKL, 2XKM, 2XKN, 2XKO, 2XKP, 2XKQ, 2XKR, 2XKW, 2XL2, 2XL3, 2XL4, 2XL6, 2XL7, 2XL8, 2XL9, 2XLA, 2XLB, 2XLC, 2XLD, 2XLE, 2XLF, 2XLG, 2XLH, 2XLL, 2XLM, 2XLN, 2XLO, 2XLP, 2XLQ, 2XLR, 2XLS, 2XLT, 2XLU, 2XLV, 2XLW, 2XLY, 2XM0, 2XM1, 2XM2, 2XM3, 2XM4, 2XM5, 2XM7, 2XM8, 2XM9, 2XMA, 2XMB, 2XMC, 2XMD, 2XME, 2XMF, 2XMG, 2XMH, 2XMI, 2XMJ, 2XMK, 2XML, 2XMM, 2XMN, 2XMO, 2XMP, 2XMQ, 2XMR, 2XMS, 2XMT, 2XMU, 2XMV, 2XMW, 2XMX, 2XMY, 2XMZ, 2XN0, 2XN1, 2XN2, 2XN3, 2XN4, 2XN5, 2XN6, 2XN7, 2XN8, 2XN9, 2XNA, 2XNB, 2XNC, 2XND, 2XNE, 2XNG, 2XNH, 2XNI, 2XNJ, 2XNK, 2XNM, 2XNN, 2XNO, 2XNP, 2XNQ, 2XNS, 2XNT, 2XNU, 2XNV, 2XNX, 2XNY, 2XO2, 2XO3, 2XO4, 2XO5, 2XO6, 2XO7, 2XO8, 2XOA, 2XOC, 2XOD, 2XOE, 2XOF, 2XOG, 2XOI, 2XOK, 2XOL, 2XOM, 2XON, 2XOT, 2XOV, 2XOW, 2XOX, 2XOY, 2XOZ, 2XP0, 2XP1, 2XP2, 2XP3, 2XP4, 2XP5, 2XP6, 2XP7, 2XP8, 2XP9, 2XPA, 2XPB, 2XPC, 2XPD, 2XPE, 2XPG, 2XPH, 2XPI, 2XPK, 2XPL, 2XPN, 2XPO, 2XPP, 2XPS, 2XPT, 2XPU, 2XPV, 2XPW, 2XPX, 2XPY, 2XPZ, 2XQ0, 2XQ1, 2XQ2, 2XQ3, 2XQ4, 2XQ5, 2XQ6, 2XQ7, 2XQ8, 2XQ9, 2XQA, 2XQB, 2XQC, 2XQF, 2XQG, 2XQH, 2XQI, 2XQJ, 2XQK, 2XQN, 2XQO, 2XQQ, 2XQR, 2XQS, 2XQT, 2XQU, 2XQV, 2XQW, 2XQX, 2XQY, 2XR0, 2XR1, 2XR4, 2XR5, 2XR6, 2XR7, 2XR8, 2XR9, 2XRA, 2XRB, 2XRC, 2XRD, 2XRE, 2XRF, 2XRG, 2XRH, 2XRI, 2XRL, 2XRM, 2XRN, 2XRO, 2XRQ, 2XRS, 2XRU, 2XRW, 2XRX, 2XRY, 2XRZ, 2XS0, 2XS1, 2XS3, 2XS4, 2XS6, 2XS8, 2XSA, 2XSB, 2XSC, 2XSD, 2XSE, 2XSF, 2XSG, 2XSH, 2XSI, 2XSJ, 2XSK, 2XSM, 2XSN, 2XSO, 2XSP, 2XSQ, 2XSR, 2XSS, 2XST, 2XSU, 2XSV, 2XSW, 2XSX, 2XSZ, 2XT0, 2XT1, 2XT2, 2XT3, 2XT4, 2XT6, 2XT9, 2XTA, 2XTB, 2XTC, 2XTD, 2XTE, 2XTH, 2XTI, 2XTJ, 2XTK, 2XTL, 2XTM, 2XTN, 2XTO, 2XTP, 2XTQ, 2XTR, 2XTS, 2XTT, 2XTU, 2XTV, 2XTW, 2XTX, 2XTY, 2XTZ, 2XU0, 2XU1, 2XU2, 2XU3, 2XU4, 2XU5, 2XU6, 2XU7, 2XU8, 2XU9, 2XUA, 2XUB, 2XUC, 2XUD, 2XUE, 2XUF, 2XUG, 2XUH, 2XUI, 2XUJ, 2XUK, 2XUL, 2XUM, 2XUO, 2XUP, 2XUQ, 2XUR, 2XUS, 2XUT, 2XUU, 2XUV, 2XUW, 2XUZ, 2XV0, 2XV1, 2XV2, 2XV3, 2XV4, 2XV5, 2XV6, 2XV7, 2XVA, 2XVB, 2XVC, 2XVD, 2XVE, 2XVF, 2XVG, 2XVH, 2XVI, 2XVJ, 2XVK, 2XVL, 2XVM, 2XVN, 2XVO, 2XVP, 2XVQ, 2XVS, 2XVT, 2XVU, 2XVV, 2XVW, 2XVX, 2XVY, 2XVZ, 2XW0, 2XW1, 2XW6, 2XW7, 2XW9, 2XWA, 2XWB, 2XWC, 2XWD, 2XWE, 2XWG, 2XWH, 2XWI, 2XWJ, 2XWK, 2XWL, 2XWM, 2XWN, 2XWO, 2XWP, 2XWQ, 2XWR, 2XWS, 2XWT, 2XWU, 2XWV, 2XWX, 2XWY, 2XWZ, 2XX0, 2XX1, 2XX2, 2XX3, 2XX4, 2XX5, 2XX6, 2XX7, 2XX8, 2XX9, 2XXB, 2XXC, 2XXD, 2XXF, 2XXG, 2XXH, 2XXI, 2XXJ, 2XXK, 2XXL, 2XXM, 2XXN, 2XXP, 2XXQ, 2XXR, 2XXT, 2XXU, 2XXV, 2XXW, 2XXX, 2XXY, 2XXZ, 2XY1, 2XY2, 2XY3, 2XY4, 2XY5, 2XY6, 2XY7, 2XY9, 2XYA, 2XYB, 2XYC, 2XYD, 2XYE, 2XYF, 2XYG, 2XYH, 2XYI, 2XYJ, 2XYK, 2XYL, 2XYM, 2XYN, 2XYO, 2XYP, 2XYQ, 2XYR, 2XYS, 2XYT, 2XYU, 2XYV, 2XYW, 2XYX, 2XZ0, 2XZ1, 2XZ2, 2XZ3, 2XZ4, 2XZ5, 2XZ6, 2XZ7, 2XZ8, 2XZ9, 2XZA, 2XZC, 2XZD, 2XZE, 2XZF, 2XZG, 2XZI, 2XZJ, 2XZK, 2XZP, 2XZQ, 2XZR, 2XZS, 2XZT, 2XZU, 2XZV, 2XZW, 2XZZ, 2Y00, 2Y01, 2Y02, 2Y03, 2Y04, 2Y05, 2Y06, 2Y07, 2Y08, 2Y09, 2Y0A, 2Y0B, 2Y0C, 2Y0D, 2Y0E, 2Y0F, 2Y0G, 2Y0H, 2Y0I, 2Y0J, 2Y0K, 2Y0L, 2Y0M, 2Y0N, 2Y0O, 2Y0P, 2Y0Q, 2Y0R, 2Y0S, 2Y0T, 2Y1A, 2Y1B, 2Y1C, 2Y1D, 2Y1E, 2Y1F, 2Y1G, 2Y1H, 2Y1I, 2Y1J, 2Y1K, 2Y1L, 2Y1M, 2Y1N, 2Y1O, 2Y1P, 2Y1Q, 2Y1R, 2Y1T, 2Y1V, 2Y1W, 2Y1X, 2Y1Y, 2Y1Z, 2Y20, 2Y21, 2Y22, 2Y23, 2Y24, 2Y25, 2Y27, 2Y28, 2Y29, 2Y2A, 2Y2B, 2Y2C, 2Y2D, 2Y2E, 2Y2F, 2Y2G, 2Y2H, 2Y2I, 2Y2J, 2Y2K, 2Y2L, 2Y2M, 2Y2N, 2Y2O, 2Y2P, 2Y2Q, 2Y2T, 2Y2U, 2Y2V, 2Y2W, 2Y2X, 2Y2Y, 2Y2Z, 2Y30, 2Y31, 2Y32, 2Y33, 2Y34, 2Y35, 2Y36, 2Y37, 2Y38, 2Y39, 2Y3A, 2Y3B, 2Y3C, 2Y3D, 2Y3E, 2Y3F, 2Y3G, 2Y3H, 2Y3I, 2Y3J, 2Y3K, 2Y3L, 2Y3M, 2Y3N, 2Y3P, 2Y3Q, 2Y3R, 2Y3S, 2Y3U, 2Y3V, 2Y3W, 2Y3X, 2Y3Y, 2Y3Z, 2Y40, 2Y41, 2Y42, 2Y43, 2Y44, 2Y46, 2Y48, 2Y4A, 2Y4D, 2Y4E, 2Y4F, 2Y4G, 2Y4I, 2Y4J, 2Y4K, 2Y4L, 2Y4M, 2Y4N, 2Y4O, 2Y4P, 2Y4R, 2Y4S, 2Y4T, 2Y4U, 2Y4V, 2Y4X, 2Y4Y, 2Y4Z, 2Y50, 2Y51, 2Y52, 2Y53, 2Y54, 2Y55, 2Y56, 2Y57, 2Y58, 2Y59, 2Y5A, 2Y5B, 2Y5C, 2Y5D, 2Y5E, 2Y5F, 2Y5G, 2Y5H, 2Y5I, 2Y5J, 2Y5K, 2Y5L, 2Y5N, 2Y5P, 2Y5Q, 2Y5S, 2Y5T, 2Y5W, 2Y5Y, 2Y5Z, 2Y60, 2Y61, 2Y62, 2Y63, 2Y64, 2Y65, 2Y66, 2Y67, 2Y68, 2Y69, 2Y6A, 2Y6B, 2Y6C, 2Y6D, 2Y6E, 2Y6F, 2Y6G, 2Y6H, 2Y6I, 2Y6J, 2Y6K, 2Y6L, 2Y6M, 2Y6O, 2Y6P, 2Y6Q, 2Y6R, 2Y6S, 2Y6T, 2Y6U, 2Y6V, 2Y6W, 2Y6X, 2Y6Y, 2Y6Z, 2Y70, 2Y71, 2Y72, 2Y73, 2Y74, 2Y75, 2Y76, 2Y77, 2Y78, 2Y79, 2Y7A, 2Y7B, 2Y7D, 2Y7E, 2Y7F, 2Y7G, 2Y7I, 2Y7J, 2Y7K, 2Y7L, 2Y7M, 2Y7N, 2Y7O, 2Y7P, 2Y7Q, 2Y7R, 2Y7S, 2Y7T, 2Y7U, 2Y7V, 2Y7W, 2Y7X, 2Y7Y, 2Y7Z, 2Y80, 2Y81, 2Y82, 2Y84, 2Y85, 2Y87, 2Y88, 2Y89, 2Y8A, 2Y8B, 2Y8C, 2Y8D, 2Y8E, 2Y8F, 2Y8G, 2Y8H, 2Y8I, 2Y8K, 2Y8L, 2Y8N, 2Y8O, 2Y8P, 2Y8Q, 2Y8R, 2Y8S, 2Y8T, 2Y8U, 2Y8V, 2Y90, 2Y91, 2Y92, 2Y93, 2Y96, 2Y98, 2Y99, 2Y9E, 2Y9F, 2Y9G, 2Y9M, 2Y9N, 2Y9P, 2Y9Q, 2Y9R, 2Y9U, 2Y9W, 2Y9X, 2Y9Y, 2Y9Z, 2YA0, 2YA1, 2YA2, 2YA3, 2YA4, 2YA5, 2YA6, 2YA7, 2YA8, 2YA9, 2YAA, 2YAB, 2YAC, 2YAD, 2YAE, 2YAF, 2YAH, 2YAJ, 2YAK, 2YAL, 2YAM, 2YAN, 2YAO, 2YAP, 2YAQ, 2YAR, 2YAS, 2YAT, 2YAU, 2YAV, 2YAW, 2YAX, 2YAY, 2YAZ, 2YB0, 2YB1, 2YB4, 2YB5, 2YB6, 2YB7, 2YB8, 2YB9, 2YBA, 2YBD, 2YBE, 2YBF, 2YBG, 2YBH, 2YBI, 2YBJ, 2YBK, 2YBL, 2YBM, 2YBN, 2YBO, 2YBP, 2YBQ, 2YBR, 2YBS, 2YBT, 2YBU, 2YBV, 2YBX, 2YBY, 2YC0, 2YC1, 2YC2, 2YC3, 2YC4, 2YC5, 2YCA, 2YCB, 2YCC, 2YCD, 2YCE, 2YCF, 2YCG, 2YCH, 2YCI, 2YCJ, 2YCK, 2YCL, 2YCM, 2YCN, 2YCP, 2YCQ, 2YCR, 2YCS, 2YCT, 2YCU, 2YCW, 2YCX, 2YCY, 2YCZ, 2YD0, 2YD1, 2YD2, 2YD3, 2YD4, 2YD5, 2YD6, 2YD7, 2YD8, 2YD9, 2YDA, 2YDB, 2YDC, 2YDD, 2YDE, 2YDF, 2YDG, 2YDI, 2YDJ, 2YDK, 2YDL, 2YDM, 2YDO, 2YDP, 2YDQ, 2YDR, 2YDS, 2YDT, 2YDU, 2YDV, 2YDW, 2YDX, 2YDY, 2YDZ, 2YE0, 2YE1, 2YE2, 2YE3, 2YE4, 2YE5, 2YE6, 2YE7, 2YE8, 2YE9, 2YEA, 2YEB, 2YEC, 2YED, 2YEE, 2YEF, 2YEG, 2YEH, 2YEI, 2YEJ, 2YEK, 2YEL, 2YEM, 2YEO, 2YEP, 2YEQ, 2YER, 2YES, 2YET, 2YEU, 2YEV, 2YEX, 2YEY, 2YEZ, 2YF0, 2YF1, 2YF2, 2YF3, 2YF4, 2YF5, 2YF6, 2YF9, 2YFA, 2YFB, 2YFC, 2YFD, 2YFE, 2YFH, 2YFI, 2YFJ, 2YFK, 2YFL, 2YFN, 2YFO, 2YFP, 2YFQ, 2YFR, 2YFS, 2YFT, 2YFU, 2YFV, 2YFW, 2YFX, 2YFY, 2YFZ, 2YG0, 2YG1, 2YG2, 2YG3, 2YG4, 2YG5, 2YG6, 2YG7, 2YG8, 2YG9, 2YGA, 2YGB, 2YGC, 2YGE, 2YGF, 2YGG, 2YGK, 2YGL, 2YGM, 2YGN, 2YGO, 2YGP, 2YGQ, 2YGS, 2YGT, 2YGU, 2YGV, 2YGW, 2YGX, 2YGY, 2YH2, 2YH3, 2YH5, 2YH6, 2YH9, 2YHA, 2YHB, 2YHC, 2YHD, 2YHE, 2YHF, 2YHG, 2YHI, 2YHJ, 2YHK, 2YHN, 2YHO, 2YHS, 2YHT, 2YHU, 2YHV, 2YHW, 2YHX, 2YHY, 2YI0, 2YI1, 2YI2, 2YI3, 2YI4, 2YI5, 2YI6, 2YI7, 2YI8, 2YI9, 2YIA, 2YIB, 2YIC, 2YID, 2YIG, 2YIH, 2YII, 2YIJ, 2YIK, 2YIL, 2YIM, 2YIN, 2YIO, 2YIP, 2YIQ, 2YIR, 2YIS, 2YIT, 2YIU, 2YIV, 2YIW, 2YIX, 2YIY, 2YIZ, 2YJ0, 2YJ1, 2YJ2, 2YJ3, 2YJ4, 2YJ5, 2YJ6, 2YJ7, 2YJ8, 2YJ9, 2YJA, 2YJB, 2YJC, 2YJD, 2YJE, 2YJF, 2YJG, 2YJH, 2YJJ, 2YJK, 2YJL, 2YJM, 2YJN, 2YJP, 2YJQ, 2YJR, 2YJS, 2YJT, 2YJV, 2YJW, 2YJX, 2YJZ, 2YK0, 2YK1, 2YK2, 2YK3, 2YK4, 2YK5, 2YK6, 2YK7, 2YK9, 2YKB, 2YKC, 2YKD, 2YKE, 2YKF, 2YKH, 2YKI, 2YKJ, 2YKK, 2YKL, 2YKM, 2YKN, 2YKO, 2YKP, 2YKQ, 2YKS, 2YKT, 2YKU, 2YKV, 2YKX, 2YKY, 2YKZ, 2YL0, 2YL1, 2YL2, 2YL3, 2YL5, 2YL6, 2YL7, 2YL8, 2YL9, 2YLA, 2YLB, 2YLC, 2YLD, 2YLE, 2YLF, 2YLG, 2YLH, 2YLI, 2YLJ, 2YLK, 2YLL, 2YLM, 2YLN, 2YLO, 2YLP, 2YLQ, 2YLR, 2YLS, 2YLT, 2YLW, 2YLX, 2YLY, 2YLZ, 2YM0, 2YM1, 2YM2, 2YM3, 2YM4, 2YM5, 2YM6, 2YM7, 2YM8, 2YM9, 2YMA, 2YMB, 2YMD, 2YME, 2YMK, 2YML, 2YMM, 2YMO, 2YMP, 2YMQ, 2YMS, 2YMT, 2YMU, 2YMV, 2YMW, 2YMX, 2YMY, 2YMZ, 2YN0, 2YN1, 2YN2, 2YN3, 2YN4, 2YN5, 2YN6, 2YN7, 2YN8, 2YNA, 2YNB, 2YNC, 2YND, 2YNE, 2YNF, 2YNG, 2YNH, 2YNI, 2YNK, 2YNM, 2YNN, 2YNO, 2YNP, 2YNQ, 2YNR, 2YNS, 2YNT, 2YNU, 2YNV, 2YNW, 2YNX, 2YNY, 2YNZ, 2YO0, 2YO1, 2YO2, 2YO3, 2YOA, 2YOB, 2YOC, 2YOE, 2YOF, 2YOG, 2YOH, 2YOI, 2YOJ, 2YOK, 2YOL, 2YOO, 2YOP, 2YOQ, 2YOR, 2YOU, 2YOV, 2YOW, 2YOX, 2YOY, 2YOZ, 2YP0, 2YP1, 2YP2, 2YP3, 2YP4, 2YP5, 2YP6, 2YP7, 2YP8, 2YP9, 2YPA, 2YPB, 2YPC, 2YPD, 2YPE, 2YPF, 2YPG, 2YPH, 2YPI, 2YPJ, 2YPK, 2YPL, 2YPM, 2YPN, 2YPO, 2YPP, 2YPQ, 2YPR, 2YPS, 2YPT, 2YPU, 2YPV, 2YPY, 2YPZ, 2YQ0, 2YQ1, 2YQ2, 2YQ3, 2YQ4, 2YQ5, 2YQ6, 2YQ7, 2YQ8, 2YQ9, 2YQB, 2YQC, 2YQH, 2YQJ, 2YQS, 2YQU, 2YQY, 2YQZ, 2YR0, 2YR1, 2YR2, 2YR4, 2YR5, 2YR6, 2YRF, 2YRI, 2YRR, 2YRS, 2YRW, 2YRX, 2YS6, 2YS7, 2YSK, 2YSS, 2YSU, 2YSW, 2YT4, 2YTZ, 2YU1, 2YU2, 2YUT, 2YV0, 2YV1, 2YV2, 2YV3, 2YV4, 2YV5, 2YV6, 2YV7, 2YV8, 2YV9, 2YVA, 2YVB, 2YVC, 2YVE, 2YVF, 2YVG, 2YVH, 2YVI, 2YVJ, 2YVK, 2YVL, 2YVM, 2YVN, 2YVO, 2YVP, 2YVQ, 2YVR, 2YVS, 2YVT, 2YVU, 2YVV, 2YVW, 2YVX, 2YVY, 2YVZ, 2YW0, 2YW2, 2YW3, 2YW4, 2YW6, 2YW7, 2YW8, 2YW9, 2YWA, 2YWB, 2YWC, 2YWD, 2YWE, 2YWF, 2YWG, 2YWH, 2YWI, 2YWJ, 2YWK, 2YWL, 2YWM, 2YWN, 2YWO, 2YWP, 2YWQ, 2YWR, 2YWV, 2YWW, 2YWX, 2YWY, 2YWZ, 2YX0, 2YX1, 2YX2, 2YX4, 2YX5, 2YX6, 2YX7, 2YX8, 2YX9, 2YXB, 2YXC, 2YXD, 2YXE, 2YXF, 2YXG, 2YXH, 2YXJ, 2YXL, 2YXM, 2YXN, 2YXO, 2YXP, 2YXQ, 2YXR, 2YXS, 2YXT, 2YXU, 2YXV, 2YXW, 2YXX, 2YXY, 2YXZ, 2YY0, 2YY1, 2YY2, 2YY3, 2YY4, 2YY5, 2YY6, 2YY7, 2YY8, 2YY9, 2YYA, 2YYB, 2YYE, 2YYG, 2YYH, 2YYI, 2YYJ, 2YYK, 2YYL, 2YYM, 2YYN, 2YYO, 2YYR, 2YYS, 2YYT, 2YYU, 2YYV, 2YYW, 2YYX, 2YYY, 2YYZ, 2YZ1, 2YZ2, 2YZ3, 2YZ5, 2YZ7, 2YZ8, 2YZA, 2YZB, 2YZC, 2YZD, 2YZE, 2YZG, 2YZH, 2YZI, 2YZJ, 2YZK, 2YZL, 2YZM, 2YZN, 2YZO, 2YZQ, 2YZR, 2YZS, 2YZT, 2YZU, 2YZV, 2YZW, 2YZY, 2Z00, 2Z01, 2Z02, 2Z04, 2Z06, 2Z07, 2Z08, 2Z09, 2Z0A, 2Z0B, 2Z0D, 2Z0E, 2Z0F, 2Z0G, 2Z0I, 2Z0J, 2Z0K, 2Z0L, 2Z0M, 2Z0N, 2Z0O, 2Z0P, 2Z0Q, 2Z0R, 2Z0S, 2Z0T, 2Z0U, 2Z0V, 2Z0W, 2Z0X, 2Z0Y, 2Z0Z, 2Z10, 2Z11, 2Z12, 2Z13, 2Z14, 2Z15, 2Z16, 2Z17, 2Z18, 2Z19, 2Z1A, 2Z1B, 2Z1C, 2Z1D, 2Z1E, 2Z1F, 2Z1G, 2Z1H, 2Z1I, 2Z1J, 2Z1K, 2Z1M, 2Z1N, 2Z1O, 2Z1P, 2Z1Q, 2Z1S, 2Z1T, 2Z1U, 2Z1V, 2Z1W, 2Z1X, 2Z1Y, 2Z1Z, 2Z20, 2Z21, 2Z22, 2Z23, 2Z24, 2Z25, 2Z26, 2Z27, 2Z28, 2Z29, 2Z2A, 2Z2B, 2Z2C, 2Z2E, 2Z2F, 2Z2I, 2Z2J, 2Z2K, 2Z2L, 2Z2M, 2Z2N, 2Z2O, 2Z2P, 2Z2R, 2Z2S, 2Z2T, 2Z2U, 2Z2W, 2Z2X, 2Z2Y, 2Z2Z, 2Z30, 2Z31, 2Z32, 2Z34, 2Z35, 2Z36, 2Z37, 2Z38, 2Z39, 2Z3A, 2Z3B, 2Z3C, 2Z3D, 2Z3E, 2Z3F, 2Z3G, 2Z3H, 2Z3I, 2Z3J, 2Z3K, 2Z3L, 2Z3M, 2Z3N, 2Z3O, 2Z3P, 2Z3Q, 2Z3R, 2Z3T, 2Z3U, 2Z3V, 2Z3W, 2Z3X, 2Z3Y, 2Z3Z, 2Z41, 2Z42, 2Z43, 2Z44, 2Z45, 2Z46, 2Z47, 2Z48, 2Z49, 2Z4B, 2Z4E, 2Z4G, 2Z4H, 2Z4I, 2Z4J, 2Z4O, 2Z4P, 2Z4Q, 2Z4R, 2Z4S, 2Z4T, 2Z4U, 2Z4V, 2Z4W, 2Z4X, 2Z4Y, 2Z4Z, 2Z50, 2Z51, 2Z52, 2Z53, 2Z54, 2Z55, 2Z56, 2Z57, 2Z58, 2Z5B, 2Z5C, 2Z5D, 2Z5E, 2Z5F, 2Z5G, 2Z5H, 2Z5I, 2Z5J, 2Z5K, 2Z5L, 2Z5M, 2Z5N, 2Z5O, 2Z5P, 2Z5Q, 2Z5R, 2Z5S, 2Z5T, 2Z5U, 2Z5W, 2Z5X, 2Z5Y, 2Z5Z, 2Z60, 2Z61, 2Z62, 2Z63, 2Z64, 2Z65, 2Z66, 2Z67, 2Z68, 2Z69, 2Z6A, 2Z6B, 2Z6C, 2Z6D, 2Z6E, 2Z6F, 2Z6G, 2Z6H, 2Z6I, 2Z6J, 2Z6K, 2Z6M, 2Z6N, 2Z6O, 2Z6P, 2Z6Q, 2Z6R, 2Z6S, 2Z6T, 2Z6U, 2Z6V, 2Z6W, 2Z6X, 2Z6Y, 2Z6Z, 2Z70, 2Z71, 2Z72, 2Z73, 2Z76, 2Z77, 2Z78, 2Z79, 2Z7A, 2Z7B, 2Z7C, 2Z7E, 2Z7F, 2Z7G, 2Z7H, 2Z7I, 2Z7J, 2Z7K, 2Z7L, 2Z7Q, 2Z7R, 2Z7S, 2Z7U, 2Z7W, 2Z7X, 2Z7Y, 2Z7Z, 2Z80, 2Z81, 2Z82, 2Z83, 2Z84, 2Z85, 2Z86, 2Z87, 2Z8A, 2Z8C, 2Z8D, 2Z8E, 2Z8F, 2Z8G, 2Z8H, 2Z8I, 2Z8J, 2Z8K, 2Z8L, 2Z8M, 2Z8N, 2Z8O, 2Z8P, 2Z8Q, 2Z8R, 2Z8S, 2Z8U, 2Z8V, 2Z8W, 2Z8X, 2Z8Y, 2Z8Z, 2Z90, 2Z91, 2Z92, 2Z93, 2Z94, 2Z95, 2Z97, 2Z98, 2Z99, 2Z9A, 2Z9B, 2Z9C, 2Z9D, 2Z9G, 2Z9H, 2Z9I, 2Z9J, 2Z9K, 2Z9L, 2Z9N, 2Z9O, 2Z9S, 2Z9T, 2Z9U, 2Z9V, 2Z9W, 2Z9X, 2Z9Y, 2Z9Z, 2ZA0, 2ZA1, 2ZA2, 2ZA3, 2ZA4, 2ZA5, 2ZA6, 2ZA7, 2ZA8, 2ZA9, 2ZAA, 2ZAB, 2ZAC, 2ZAD, 2ZAE, 2ZAF, 2ZAG, 2ZAI, 2ZAK, 2ZAL, 2ZAM, 2ZAN, 2ZAO, 2ZAS, 2ZAT, 2ZAU, 2ZAV, 2ZAW, 2ZAX, 2ZAY, 2ZAZ, 2ZB0, 2ZB1, 2ZB2, 2ZB3, 2ZB4, 2ZB5, 2ZB6, 2ZB7, 2ZB8, 2ZB9, 2ZBA, 2ZBB, 2ZBC, 2ZBD, 2ZBE, 2ZBF, 2ZBG, 2ZBH, 2ZBI, 2ZBJ, 2ZBK, 2ZBL, 2ZBM, 2ZBN, 2ZBO, 2ZBP, 2ZBQ, 2ZBR, 2ZBS, 2ZBT, 2ZBU, 2ZBV, 2ZBW, 2ZBX, 2ZBY, 2ZBZ, 2ZC0, 2ZC1, 2ZC2, 2ZC3, 2ZC4, 2ZC5, 2ZC6, 2ZC7, 2ZC8, 2ZC9, 2ZCA, 2ZCB, 2ZCC, 2ZCE, 2ZCF, 2ZCG, 2ZCH, 2ZCI, 2ZCJ, 2ZCK, 2ZCL, 2ZCM, 2ZCN, 2ZCO, 2ZCQ, 2ZCR, 2ZCS, 2ZCT, 2ZCU, 2ZCV, 2ZCW, 2ZCX, 2ZCY, 2ZCZ, 2ZD0, 2ZD1, 2ZD2, 2ZD7, 2ZD8, 2ZD9, 2ZDA, 2ZDC, 2ZDG, 2ZDH, 2ZDI, 2ZDJ, 2ZDK, 2ZDL, 2ZDM, 2ZDN, 2ZDO, 2ZDP, 2ZDQ, 2ZDR, 2ZDS, 2ZDT, 2ZDU, 2ZDV, 2ZDX, 2ZDY, 2ZDZ, 2ZE0, 2ZE1, 2ZE2, 2ZE3, 2ZE4, 2ZE5, 2ZE6, 2ZE7, 2ZE8, 2ZE9, 2ZEB, 2ZEC, 2ZED, 2ZEE, 2ZEF, 2ZEG, 2ZEH, 2ZEJ, 2ZEL, 2ZEM, 2ZEN, 2ZEO, 2ZEP, 2ZEQ, 2ZET, 2ZEU, 2ZEV, 2ZEW, 2ZEX, 2ZEY, 2ZEZ, 2ZF0, 2ZF3, 2ZF4, 2ZF5, 2ZF8, 2ZF9, 2ZFA, 2ZFB, 2ZFC, 2ZFD, 2ZFE, 2ZFF, 2ZFG, 2ZFH, 2ZFI, 2ZFJ, 2ZFK, 2ZFL, 2ZFM, 2ZFN, 2ZFO, 2ZFP, 2ZFQ, 2ZFR, 2ZFS, 2ZFT, 2ZFU, 2ZFW, 2ZFX, 2ZFY, 2ZFZ, 2ZG0, 2ZG1, 2ZG2, 2ZG3, 2ZG6, 2ZG7, 2ZG8, 2ZG9, 2ZGA, 2ZGB, 2ZGC, 2ZGD, 2ZGG, 2ZGH, 2ZGI, 2ZGJ, 2ZGK, 2ZGL, 2ZGM, 2ZGN, 2ZGO, 2ZGP, 2ZGQ, 2ZGR, 2ZGS, 2ZGT, 2ZGU, 2ZGV, 2ZGW, 2ZGX, 2ZGY, 2ZGZ, 2ZHD, 2ZHE, 2ZHF, 2ZHG, 2ZHH, 2ZHI, 2ZHJ, 2ZHK, 2ZHL, 2ZHM, 2ZHN, 2ZHO, 2ZHP, 2ZHQ, 2ZHR, 2ZHS, 2ZHT, 2ZHU, 2ZHV, 2ZHW, 2ZHX, 2ZHY, 2ZHZ, 2ZI2, 2ZI3, 2ZI4, 2ZI5, 2ZI6, 2ZI7, 2ZI8, 2ZI9, 2ZIA, 2ZIB, 2ZIC, 2ZID, 2ZIE, 2ZIF, 2ZIG, 2ZIH, 2ZII, 2ZIJ, 2ZIK, 2ZIL, 2ZIM, 2ZIN, 2ZIO, 2ZIQ, 2ZIR, 2ZIS, 2ZIT, 2ZIU, 2ZIV, 2ZIW, 2ZIX, 2ZIY, 2ZIZ, 2ZJ0, 2ZJ1, 2ZJ2, 2ZJ3, 2ZJ4, 2ZJ5, 2ZJ6, 2ZJ7, 2ZJ8, 2ZJ9, 2ZJA, 2ZJB, 2ZJC, 2ZJD, 2ZJF, 2ZJG, 2ZJH, 2ZJI, 2ZJJ, 2ZJK, 2ZJL, 2ZJM, 2ZJN, 2ZJO, 2ZJS, 2ZJT, 2ZJU, 2ZJV, 2ZJW, 2ZJX, 2ZJY, 2ZJZ, 2ZK0, 2ZK1, 2ZK2, 2ZK3, 2ZK4, 2ZK5, 2ZK6, 2ZK7, 2ZK9, 2ZKA, 2ZKB, 2ZKC, 2ZKD, 2ZKE, 2ZKF, 2ZKG, 2ZKH, 2ZKI, 2ZKJ, 2ZKL, 2ZKM, 2ZKN, 2ZKS, 2ZKT, 2ZKU, 2ZKW, 2ZKX, 2ZKY, 2ZKZ, 2ZL0, 2ZL1, 2ZL2, 2ZL3, 2ZL4, 2ZL5, 2ZL6, 2ZL7, 2ZL8, 2ZL9, 2ZLA, 2ZLB, 2ZLC, 2ZLD, 2ZLF, 2ZLG, 2ZLT, 2ZLU, 2ZLV, 2ZLW, 2ZLX, 2ZLY, 2ZM0, 2ZM1, 2ZM2, 2ZM3, 2ZM4, 2ZM7, 2ZM8, 2ZM9, 2ZMA, 2ZMB, 2ZMC, 2ZMD, 2ZME, 2ZMF, 2ZMH, 2ZMI, 2ZMJ, 2ZMK, 2ZML, 2ZMM, 2ZMN, 2ZMU, 2ZMV, 2ZMW, 2ZMX, 2ZMY, 2ZMZ, 2ZN7, 2ZN8, 2ZN9, 2ZNB, 2ZNC, 2ZND, 2ZNE, 2ZNH, 2ZNJ, 2ZNK, 2ZNL, 2ZNM, 2ZNN, 2ZNO, 2ZNP, 2ZNQ, 2ZNR, 2ZNS, 2ZNT, 2ZNU, 2ZNV, 2ZNW, 2ZNX, 2ZNY, 2ZNZ, 2ZO0, 2ZO1, 2ZO2, 2ZO3, 2ZO4, 2ZO5, 2ZO6, 2ZO7, 2ZO9, 2ZOA, 2ZOC, 2ZOD, 2ZOE, 2ZOF, 2ZOG, 2ZOH, 2ZOK, 2ZOL, 2ZOM, 2ZON, 2ZOO, 2ZOP, 2ZOQ, 2ZOS, 2ZOT, 2ZOU, 2ZOV, 2ZOW, 2ZOX, 2ZOY, 2ZOZ, 2ZP0, 2ZP1, 2ZP2, 2ZP3, 2ZP4, 2ZP5, 2ZP6, 2ZP7, 2ZP8, 2ZP9, 2ZPA, 2ZPB, 2ZPC, 2ZPD, 2ZPE, 2ZPF, 2ZPG, 2ZPH, 2ZPI, 2ZPK, 2ZPL, 2ZPM, 2ZPN, 2ZPO, 2ZPQ, 2ZPR, 2ZPS, 2ZPT, 2ZPU, 2ZPX, 2ZPY, 2ZQ0, 2ZQ1, 2ZQ2, 2ZQ3, 2ZQ4, 2ZQ5, 2ZQ7, 2ZQ8, 2ZQ9, 2ZQA, 2ZQB, 2ZQC, 2ZQD, 2ZQE, 2ZQJ, 2ZQK, 2ZQM, 2ZQN, 2ZQO, 2ZQP, 2ZQQ, 2ZQR, 2ZQS, 2ZQT, 2ZQU, 2ZQV, 2ZQX, 2ZQY, 2ZQZ, 2ZR0, 2ZR1, 2ZR2, 2ZR3, 2ZR4, 2ZR5, 2ZR6, 2ZR7, 2ZR8, 2ZR9, 2ZRA, 2ZRB, 2ZRC, 2ZRD, 2ZRE, 2ZRF, 2ZRG, 2ZRH, 2ZRI, 2ZRJ, 2ZRK, 2ZRL, 2ZRM, 2ZRN, 2ZRO, 2ZRP, 2ZRQ, 2ZRR, 2ZRS, 2ZRT, 2ZRU, 2ZRV, 2ZRW, 2ZRX, 2ZRY, 2ZRZ, 2ZS0, 2ZS1, 2ZS6, 2ZS7, 2ZS8, 2ZS9, 2ZSA, 2ZSB, 2ZSC, 2ZSD, 2ZSE, 2ZSF, 2ZSG, 2ZSH, 2ZSI, 2ZSJ, 2ZSK, 2ZSL, 2ZSM, 2ZSN, 2ZSO, 2ZSP, 2ZSQ, 2ZSR, 2ZSS, 2ZST, 2ZSU, 2ZSV, 2ZSW, 2ZSX, 2ZSY, 2ZSZ, 2ZT0, 2ZT1, 2ZT2, 2ZT3, 2ZT4, 2ZT5, 2ZT6, 2ZT7, 2ZT8, 2ZT9, 2ZTA, 2ZTB, 2ZTC, 2ZTD, 2ZTE, 2ZTG, 2ZTH, 2ZTI, 2ZTJ, 2ZTK, 2ZTL, 2ZTM, 2ZTS, 2ZTT, 2ZTU, 2ZTV, 2ZTW, 2ZTX, 2ZTY, 2ZTZ, 2ZU0, 2ZU1, 2ZU2, 2ZU3, 2ZU4, 2ZU5, 2ZU6, 2ZU7, 2ZU8, 2ZU9, 2ZUA, 2ZUB, 2ZUC, 2ZUD, 2ZUE, 2ZUF, 2ZUG, 2ZUH, 2ZUI, 2ZUJ, 2ZUK, 2ZUL, 2ZUM, 2ZUN, 2ZUP, 2ZUQ, 2ZUR, 2ZUS, 2ZUT, 2ZUU, 2ZUV, 2ZUW, 2ZUX, 2ZUY, 2ZV2, 2ZV3, 2ZV6, 2ZV7, 2ZV8, 2ZV9, 2ZVA, 2ZVB, 2ZVC, 2ZVD, 2ZVF, 2ZVI, 2ZVJ, 2ZVK, 2ZVL, 2ZVM, 2ZVN, 2ZVO, 2ZVP, 2ZVQ, 2ZVR, 2ZVS, 2ZVT, 2ZVU, 2ZVV, 2ZVW, 2ZVX, 2ZVY, 2ZVZ, 2ZW0, 2ZW1, 2ZW2, 2ZW3, 2ZW4, 2ZW5, 2ZW6, 2ZW7, 2ZW9, 2ZWA, 2ZWD, 2ZWE, 2ZWF, 2ZWG, 2ZWI, 2ZWJ, 2ZWK, 2ZWL, 2ZWM, 2ZWN, 2ZWO, 2ZWP, 2ZWR, 2ZWS, 2ZWT, 2ZWU, 2ZWV, 2ZWY, 2ZWZ, 2ZX0, 2ZX1, 2ZX2, 2ZX3, 2ZX4, 2ZX5, 2ZX6, 2ZX7, 2ZX8, 2ZX9, 2ZXA, 2ZXB, 2ZXC, 2ZXD, 2ZXE, 2ZXF, 2ZXG, 2ZXH, 2ZXI, 2ZXJ, 2ZXK, 2ZXL, 2ZXM, 2ZXN, 2ZXO, 2ZXP, 2ZXQ, 2ZXR, 2ZXT, 2ZXV, 2ZXW, 2ZXX, 2ZXY, 2ZXZ, 2ZY0, 2ZY1, 2ZY2, 2ZY3, 2ZY4, 2ZY5, 2ZY9, 2ZYA, 2ZYB, 2ZYC, 2ZYD, 2ZYF, 2ZYG, 2ZYH, 2ZYI, 2ZYJ, 2ZYK, 2ZYL, 2ZYM, 2ZYN, 2ZYO, 2ZYP, 2ZYQ, 2ZYR, 2ZYS, 2ZYT, 2ZYU, 2ZYV, 2ZYW, 2ZYZ, 2ZZ0, 2ZZ1, 2ZZ2, 2ZZ3, 2ZZ4, 2ZZ5, 2ZZ6, 2ZZ7, 2ZZ8, 2ZZA, 2ZZB, 2ZZC, 2ZZD, 2ZZE, 2ZZF, 2ZZG, 2ZZI, 2ZZJ, 2ZZK, 2ZZL, 2ZZO, 2ZZP, 2ZZR, 2ZZS, 2ZZT, 2ZZU, 2ZZV, 2ZZW, 2ZZX, 302D, 303D, 304D, 305D, 306D, 307D, 309D, 311D, 312D, 313D, 314D, 316D, 317D, 318D, 319D, 31BI, 320D, 321D, 322D, 323D, 324D, 325D, 327D, 328D, 329D, 32C2, 330D, 331D, 334D, 335D, 336D, 338D, 343D, 344D, 345D, 348D, 349D, 351C, 351D, 352D, 355D, 35C8, 360D, 362D, 368D, 369D, 370D, 371D, 372D, 375D, 376D, 378D, 382D, 386D, 388D, 389D, 390D, 391D, 392D, 393D, 394D, 395D, 396D, 399D, 3A01, 3A02, 3A03, 3A04, 3A05, 3A06, 3A07, 3A08, 3A09, 3A0A, 3A0B, 3A0C, 3A0D, 3A0E, 3A0F, 3A0G, 3A0H, 3A0I, 3A0J, 3A0K, 3A0M, 3A0N, 3A0O, 3A0R, 3A0S, 3A0T, 3A0U, 3A0V, 3A0W, 3A0X, 3A0Y, 3A0Z, 3A10, 3A11, 3A12, 3A13, 3A14, 3A15, 3A16, 3A17, 3A18, 3A19, 3A1A, 3A1B, 3A1C, 3A1D, 3A1E, 3A1F, 3A1G, 3A1H, 3A1I, 3A1J, 3A1K, 3A1L, 3A1M, 3A1N, 3A1P, 3A1Q, 3A1S, 3A1T, 3A1U, 3A1V, 3A1W, 3A1Y, 3A1Z, 3A20, 3A21, 3A22, 3A23, 3A24, 3A25, 3A26, 3A27, 3A28, 3A29, 3A2A, 3A2B, 3A2C, 3A2E, 3A2F, 3A2G, 3A2H, 3A2I, 3A2J, 3A2L, 3A2M, 3A2N, 3A2O, 3A2P, 3A2Q, 3A2S, 3A2V, 3A2W, 3A2X, 3A2Y, 3A2Z, 3A30, 3A31, 3A32, 3A33, 3A34, 3A35, 3A36, 3A37, 3A38, 3A39, 3A3B, 3A3C, 3A3D, 3A3E, 3A3F, 3A3G, 3A3H, 3A3I, 3A3J, 3A3K, 3A3N, 3A3O, 3A3P, 3A3Q, 3A3R, 3A3T, 3A3U, 3A3V, 3A3W, 3A3X, 3A3Y, 3A3Z, 3A40, 3A42, 3A43, 3A44, 3A45, 3A46, 3A47, 3A4A, 3A4C, 3A4D, 3A4E, 3A4F, 3A4G, 3A4H, 3A4I, 3A4J, 3A4K, 3A4L, 3A4M, 3A4N, 3A4O, 3A4P, 3A4R, 3A4S, 3A4T, 3A4U, 3A4V, 3A4W, 3A4X, 3A4Y, 3A4Z, 3A50, 3A51, 3A52, 3A54, 3A55, 3A56, 3A57, 3A58, 3A59, 3A5A, 3A5B, 3A5C, 3A5D, 3A5E, 3A5F, 3A5G, 3A5I, 3A5J, 3A5K, 3A5L, 3A5M, 3A5N, 3A5O, 3A5P, 3A5Q, 3A5R, 3A5S, 3A5T, 3A5U, 3A5V, 3A5W, 3A5Y, 3A5Z, 3A60, 3A61, 3A62, 3A64, 3A65, 3A66, 3A67, 3A68, 3A6B, 3A6C, 3A6D, 3A6E, 3A6F, 3A6G, 3A6H, 3A6J, 3A6K, 3A6L, 3A6M, 3A6N, 3A6O, 3A6Q, 3A6R, 3A6S, 3A6T, 3A6U, 3A6V, 3A6Z, 3A70, 3A71, 3A72, 3A73, 3A74, 3A75, 3A76, 3A77, 3A78, 3A79, 3A7A, 3A7B, 3A7C, 3A7D, 3A7E, 3A7F, 3A7G, 3A7H, 3A7I, 3A7J, 3A7K, 3A7L, 3A7M, 3A7N, 3A7O, 3A7P, 3A7Q, 3A7R, 3A7S, 3A7T, 3A7U, 3A7V, 3A7W, 3A7X, 3A7Y, 3A7Z, 3A80, 3A81, 3A82, 3A83, 3A84, 3A85, 3A86, 3A87, 3A88, 3A89, 3A8A, 3A8B, 3A8C, 3A8D, 3A8E, 3A8G, 3A8H, 3A8I, 3A8J, 3A8K, 3A8L, 3A8M, 3A8N, 3A8O, 3A8P, 3A8Q, 3A8R, 3A8S, 3A8T, 3A8U, 3A8W, 3A8X, 3A8Y, 3A8Z, 3A90, 3A91, 3A92, 3A93, 3A94, 3A95, 3A96, 3A98, 3A99, 3A9B, 3A9C, 3A9E, 3A9F, 3A9G, 3A9H, 3A9I, 3A9J, 3A9K, 3A9L, 3A9M, 3A9Q, 3A9R, 3A9S, 3A9T, 3A9U, 3A9V, 3A9W, 3A9X, 3A9Y, 3A9Z, 3AA0, 3AA1, 3AA2, 3AA3, 3AA4, 3AA5, 3AA6, 3AA7, 3AA8, 3AA9, 3AAA, 3AAB, 3AAC, 3AAD, 3AAE, 3AAF, 3AAG, 3AAI, 3AAJ, 3AAK, 3AAL, 3AAM, 3AAP, 3AAQ, 3AAR, 3AAS, 3AAT, 3AAU, 3AAV, 3AAW, 3AAX, 3AAY, 3AAZ, 3AB0, 3AB1, 3AB2, 3AB3, 3AB4, 3AB5, 3AB6, 3AB7, 3AB8, 3AB9, 3ABA, 3ABB, 3ABD, 3ABE, 3ABF, 3ABG, 3ABH, 3ABI, 3ABK, 3ABL, 3ABM, 3ABN, 3ABO, 3ABQ, 3ABR, 3ABS, 3ABT, 3ABU, 3ABV, 3ABW, 3ABX, 3ABZ, 3AC0, 3AC1, 3AC2, 3AC3, 3AC4, 3AC5, 3AC8, 3AC9, 3ACA, 3ACB, 3ACC, 3ACD, 3ACF, 3ACG, 3ACH, 3ACI, 3ACJ, 3ACK, 3ACL, 3ACO, 3ACP, 3ACS, 3ACT, 3ACW, 3ACX, 3ACY, 3ACZ, 3AD4, 3AD5, 3AD6, 3AD7, 3AD8, 3AD9, 3ADA, 3ADE, 3ADF, 3ADG, 3ADJ, 3ADK, 3ADM, 3ADO, 3ADP, 3ADR, 3ADS, 3ADT, 3ADU, 3ADV, 3ADW, 3ADX, 3ADY, 3ADZ, 3AE0, 3AE1, 3AE2, 3AE3, 3AE4, 3AE5, 3AE6, 3AE7, 3AE8, 3AE9, 3AEA, 3AEB, 3AEC, 3AED, 3AEE, 3AEF, 3AEG, 3AEH, 3AEI, 3AEJ, 3AEK, 3AEL, 3AEM, 3AEN, 3AEO, 3AEP, 3AEQ, 3AER, 3AES, 3AET, 3AEU, 3AEX, 3AEY, 3AEZ, 3AF0, 3AF1, 3AF2, 3AF3, 3AF4, 3AF5, 3AF6, 3AF7, 3AF8, 3AF9, 3AFA, 3AFB, 3AFC, 3AFE, 3AFF, 3AFG, 3AFH, 3AFI, 3AFJ, 3AFK, 3AFL, 3AFM, 3AFN, 3AFO, 3AFP, 3AFQ, 3AFR, 3AFV, 3AG0, 3AG1, 3AG2, 3AG3, 3AG4, 3AG5, 3AG6, 3AG7, 3AG9, 3AGA, 3AGB, 3AGC, 3AGD, 3AGE, 3AGF, 3AGG, 3AGH, 3AGI, 3AGK, 3AGL, 3AGM, 3AGN, 3AGO, 3AGP, 3AGQ, 3AGR, 3AGT, 3AGU, 3AGW, 3AGX, 3AGY, 3AGZ, 3AH1, 3AH2, 3AH3, 3AH4, 3AH5, 3AH6, 3AH7, 3AH8, 3AH9, 3AHA, 3AHC, 3AHD, 3AHE, 3AHF, 3AHG, 3AHH, 3AHI, 3AHJ, 3AHM, 3AHN, 3AHO, 3AHP, 3AHQ, 3AHR, 3AHS, 3AHT, 3AHV, 3AHW, 3AHX, 3AHY, 3AHZ, 3AI0, 3AI1, 3AI2, 3AI3, 3AI4, 3AI5, 3AI7, 3AI8, 3AI9, 3AIA, 3AIB, 3AIC, 3AID, 3AIE, 3AIG, 3AIH, 3AII, 3AIK, 3AIL, 3AIM, 3AIN, 3AIO, 3AIQ, 3AIR, 3AIS, 3AIU, 3AIV, 3AIW, 3AIX, 3AIZ, 3AJ1, 3AJ2, 3AJ3, 3AJ4, 3AJ5, 3AJ6, 3AJ7, 3AJ8, 3AJ9, 3AJA, 3AJB, 3AJC, 3AJD, 3AJE, 3AJF, 3AJG, 3AJH, 3AJI, 3AJJ, 3AJK, 3AJL, 3AJM, 3AJN, 3AJO, 3AJP, 3AJQ, 3AJR, 3AJV, 3AJW, 3AJX, 3AJY, 3AJZ, 3AK0, 3AK1, 3AK2, 3AK3, 3AK4, 3AK5, 3AK8, 3AK9, 3AKA, 3AKB, 3AKC, 3AKD, 3AKE, 3AKF, 3AKG, 3AKH, 3AKI, 3AKJ, 3AKK, 3AKL, 3AKM, 3AKN, 3AKO, 3AKP, 3AKQ, 3AKR, 3AKS, 3AKT, 3AKY, 3AL1, 3AL2, 3AL3, 3AL4, 3AL5, 3AL6, 3AL7, 3AL8, 3AL9, 3ALA, 3ALB, 3ALD, 3ALE, 3ALF, 3ALG, 3ALJ, 3ALL, 3ALM, 3ALN, 3ALO, 3ALP, 3ALQ, 3ALR, 3ALS, 3ALT, 3ALU, 3ALW, 3ALX, 3ALY, 3ALZ, 3AM2, 3AM3, 3AM4, 3AM5, 3AM6, 3AM7, 3AM8, 3AM9, 3AMA, 3AMB, 3AMC, 3AMD, 3AME, 3AMF, 3AMG, 3AMH, 3AMI, 3AMJ, 3AMK, 3AML, 3AMM, 3AMN, 3AMO, 3AMP, 3AMQ, 3AMR, 3AMS, 3AMV, 3AMY, 3AMZ, 3AN1, 3AN2, 3AN3, 3AN4, 3ANA, 3ANG, 3ANI, 3ANJ, 3ANK, 3ANL, 3ANM, 3ANN, 3ANO, 3ANP, 3ANQ, 3ANR, 3ANS, 3ANT, 3ANU, 3ANV, 3ANW, 3ANX, 3ANY, 3ANZ, 3AO0, 3AO1, 3AO2, 3AO3, 3AO4, 3AO5, 3AO9, 3AOA, 3AOB, 3AOC, 3AOD, 3AOE, 3AOF, 3AOG, 3AOK, 3AON, 3AOP, 3AOS, 3AOT, 3AOU, 3AOV, 3AOW, 3AOX, 3AP1, 3AP2, 3AP3, 3AP4, 3AP5, 3AP6, 3AP7, 3AP9, 3APA, 3APB, 3APC, 3APD, 3APF, 3APG, 3APM, 3APN, 3APO, 3APP, 3APQ, 3APR, 3APS, 3APT, 3APU, 3APV, 3APW, 3APX, 3APY, 3APZ, 3AQ0, 3AQ1, 3AQ2, 3AQ3, 3AQ4, 3AQ5, 3AQ6, 3AQ7, 3AQ8, 3AQ9, 3AQA, 3AQB, 3AQC, 3AQD, 3AQE, 3AQF, 3AQG, 3AQI, 3AQJ, 3AQK, 3AQL, 3AQM, 3AQN, 3AQO, 3AQP, 3AQQ, 3AQS, 3AQT, 3AQU, 3AQV, 3AQX, 3AQY, 3AQZ, 3AR2, 3AR3, 3AR4, 3AR5, 3AR6, 3AR7, 3AR8, 3AR9, 3ARA, 3ARB, 3ARD, 3ARE, 3ARF, 3ARG, 3ARJ, 3ARK, 3ARL, 3ARN, 3ARO, 3ARP, 3ARQ, 3ARR, 3ARS, 3ART, 3ARU, 3ARV, 3ARW, 3ARX, 3ARY, 3ARZ, 3AS0, 3AS1, 3AS2, 3AS3, 3AS4, 3AS5, 3AS8, 3ASA, 3ASB, 3ASD, 3ASE, 3ASF, 3ASG, 3ASH, 3ASI, 3ASJ, 3ASK, 3ASL, 3ASM, 3ASN, 3ASO, 3ASP, 3ASQ, 3ASR, 3ASS, 3AST, 3ASU, 3ASV, 3ASW, 3ASX, 3ASY, 3ASZ, 3AT0, 3AT1, 3AT2, 3AT3, 3AT4, 3AT5, 3AT6, 3AT7, 3AT8, 3AT9, 3ATA, 3ATB, 3ATD, 3ATE, 3ATF, 3ATG, 3ATH, 3ATI, 3ATJ, 3ATK, 3ATL, 3ATM, 3ATN, 3ATO, 3ATP, 3ATQ, 3ATR, 3ATS, 3ATT, 3ATU, 3ATV, 3ATW, 3ATY, 3ATZ, 3AU0, 3AU1, 3AU2, 3AU3, 3AU4, 3AU5, 3AU6, 3AU7, 3AU8, 3AU9, 3AUA, 3AUB, 3AUC, 3AUD, 3AUE, 3AUF, 3AUG, 3AUH, 3AUI, 3AUJ, 3AUK, 3AUL, 3AUM, 3AUN, 3AUO, 3AUP, 3AUQ, 3AUR, 3AUS, 3AUT, 3AUU, 3AUV, 3AUW, 3AUX, 3AUY, 3AUZ, 3AV0, 3AV1, 3AV2, 3AV3, 3AV4, 3AV5, 3AV6, 3AV7, 3AV8, 3AV9, 3AVA, 3AVB, 3AVC, 3AVE, 3AVF, 3AVG, 3AVH, 3AVI, 3AVJ, 3AVK, 3AVL, 3AVM, 3AVN, 3AVO, 3AVP, 3AVQ, 3AVR, 3AVS, 3AVZ, 3AW0, 3AW1, 3AW5, 3AW6, 3AW7, 3AW8, 3AW9, 3AWD, 3AWE, 3AWF, 3AWG, 3AWH, 3AWI, 3AWJ, 3AWK, 3AWM, 3AWN, 3AWO, 3AWP, 3AWQ, 3AWR, 3AWS, 3AWT, 3AWU, 3AWV, 3AWW, 3AWX, 3AWY, 3AWZ, 3AX0, 3AX1, 3AX2, 3AX3, 3AX4, 3AX5, 3AX6, 3AX7, 3AX8, 3AX9, 3AXA, 3AXB, 3AXC, 3AXD, 3AXE, 3AXF, 3AXG, 3AXH, 3AXI, 3AXJ, 3AXK, 3AXL, 3AXM, 3AXS, 3AXT, 3AXW, 3AXX, 3AXY, 3AXZ, 3AY0, 3AY2, 3AY3, 3AY4, 3AY5, 3AY6, 3AY7, 3AY8, 3AY9, 3AYA, 3AYC, 3AYD, 3AYE, 3AYF, 3AYG, 3AYH, 3AYI, 3AYJ, 3AYL, 3AYM, 3AYN, 3AYQ, 3AYR, 3AYS, 3AYT, 3AYU, 3AYV, 3AYW, 3AYX, 3AYY, 3AYZ, 3AZ1, 3AZ2, 3AZ3, 3AZ4, 3AZ5, 3AZ6, 3AZ7, 3AZ8, 3AZ9, 3AZA, 3AZB, 3AZC, 3AZD, 3AZE, 3AZF, 3AZG, 3AZH, 3AZI, 3AZJ, 3AZK, 3AZL, 3AZM, 3AZN, 3AZO, 3AZP, 3AZQ, 3AZR, 3AZS, 3AZT, 3AZU, 3AZV, 3AZW, 3AZX, 3AZY, 3AZZ, 3B00, 3B01, 3B02, 3B03, 3B04, 3B05, 3B06, 3B07, 3B08, 3B09, 3B0A, 3B0B, 3B0C, 3B0D, 3B0F, 3B0G, 3B0H, 3B0I, 3B0J, 3B0K, 3B0L, 3B0M, 3B0N, 3B0O, 3B0P, 3B0Q, 3B0R, 3B0T, 3B0W, 3B0X, 3B0Y, 3B0Z, 3B12, 3B13, 3B18, 3B1B, 3B1C, 3B1D, 3B1E, 3B1F, 3B1J, 3B1K, 3B1L, 3B1M, 3B1N, 3B1O, 3B1P, 3B1Q, 3B1R, 3B1S, 3B1T, 3B1U, 3B1V, 3B1W, 3B1X, 3B1Y, 3B1Z, 3B20, 3B21, 3B23, 3B24, 3B25, 3B26, 3B27, 3B28, 3B29, 3B2D, 3B2E, 3B2F, 3B2G, 3B2H, 3B2I, 3B2J, 3B2K, 3B2L, 3B2M, 3B2N, 3B2P, 3B2Q, 3B2R, 3B2S, 3B2T, 3B2U, 3B2V, 3B2W, 3B2X, 3B2Y, 3B2Z, 3B30, 3B32, 3B33, 3B34, 3B35, 3B36, 3B37, 3B38, 3B39, 3B3A, 3B3B, 3B3C, 3B3D, 3B3F, 3B3G, 3B3I, 3B3J, 3B3K, 3B3L, 3B3M, 3B3N, 3B3O, 3B3P, 3B3Q, 3B3R, 3B3S, 3B3T, 3B3V, 3B3W, 3B3X, 3B40, 3B42, 3B43, 3B44, 3B45, 3B46, 3B47, 3B48, 3B49, 3B4D, 3B4F, 3B4M, 3B4N, 3B4O, 3B4P, 3B4Q, 3B4R, 3B4S, 3B4T, 3B4U, 3B4V, 3B4W, 3B4X, 3B4Y, 3B50, 3B51, 3B52, 3B53, 3B54, 3B55, 3B56, 3B57, 3B59, 3B5B, 3B5D, 3B5E, 3B5G, 3B5H, 3B5I, 3B5J, 3B5K, 3B5L, 3B5M, 3B5N, 3B5O, 3B5P, 3B5Q, 3B5R, 3B5T, 3B5W, 3B5X, 3B5Y, 3B5Z, 3B60, 3B61, 3B62, 3B64, 3B65, 3B66, 3B67, 3B68, 3B69, 3B6A, 3B6B, 3B6C, 3B6D, 3B6E, 3B6F, 3B6G, 3B6H, 3B6I, 3B6J, 3B6K, 3B6L, 3B6M, 3B6N, 3B6O, 3B6P, 3B6Q, 3B6R, 3B6S, 3B6T, 3B6U, 3B6V, 3B6W, 3B6X, 3B6Y, 3B6Z, 3B70, 3B71, 3B72, 3B73, 3B74, 3B75, 3B76, 3B77, 3B78, 3B79, 3B7A, 3B7B, 3B7C, 3B7D, 3B7E, 3B7F, 3B7G, 3B7H, 3B7I, 3B7J, 3B7K, 3B7L, 3B7M, 3B7N, 3B7O, 3B7P, 3B7Q, 3B7R, 3B7S, 3B7T, 3B7U, 3B7V, 3B7W, 3B7X, 3B7Y, 3B7Z, 3B80, 3B81, 3B82, 3B83, 3B84, 3B85, 3B86, 3B87, 3B88, 3B89, 3B8A, 3B8B, 3B8C, 3B8D, 3B8E, 3B8F, 3B8G, 3B8H, 3B8I, 3B8J, 3B8L, 3B8M, 3B8N, 3B8O, 3B8P, 3B8Q, 3B8R, 3B8S, 3B8T, 3B8U, 3B8V, 3B8W, 3B8X, 3B8Y, 3B8Z, 3B90, 3B92, 3B93, 3B94, 3B95, 3B96, 3B97, 3B98, 3B99, 3B9A, 3B9B, 3B9C, 3B9D, 3B9E, 3B9F, 3B9G, 3B9H, 3B9I, 3B9J, 3B9K, 3B9L, 3B9M, 3B9N, 3B9O, 3B9P, 3B9Q, 3B9R, 3B9S, 3B9T, 3B9U, 3B9V, 3B9W, 3B9X, 3B9Y, 3B9Z, 3BA0, 3BA1, 3BA2, 3BA3, 3BA4, 3BA5, 3BA6, 3BA7, 3BA8, 3BA9, 3BAA, 3BAB, 3BAC, 3BAD, 3BAE, 3BAF, 3BAG, 3BAH, 3BAI, 3BAJ, 3BAK, 3BAL, 3BAM, 3BAN, 3BAO, 3BAP, 3BAQ, 3BAR, 3BAS, 3BAT, 3BAU, 3BAV, 3BAW, 3BAX, 3BAY, 3BAZ, 3BB0, 3BB1, 3BB2, 3BB3, 3BB4, 3BB5, 3BB6, 3BB7, 3BB8, 3BB9, 3BBA, 3BBB, 3BBC, 3BBD, 3BBE, 3BBF, 3BBH, 3BBJ, 3BBL, 3BBP, 3BBR, 3BBT, 3BBW, 3BBY, 3BBZ, 3BC1, 3BC2, 3BC3, 3BC4, 3BC5, 3BC8, 3BC9, 3BCA, 3BCB, 3BCC, 3BCD, 3BCE, 3BCF, 3BCG, 3BCH, 3BCI, 3BCJ, 3BCK, 3BCM, 3BCN, 3BCO, 3BCP, 3BCQ, 3BCR, 3BCS, 3BCT, 3BCU, 3BCV, 3BCW, 3BCX, 3BCY, 3BCZ, 3BD0, 3BD1, 3BD2, 3BD3, 3BD4, 3BD5, 3BD6, 3BD7, 3BD8, 3BD9, 3BDA, 3BDB, 3BDC, 3BDD, 3BDE, 3BDF, 3BDG, 3BDH, 3BDI, 3BDJ, 3BDK, 3BDL, 3BDM, 3BDN, 3BDP, 3BDQ, 3BDR, 3BDU, 3BDV, 3BDW, 3BDX, 3BDY, 3BDZ, 3BE0, 3BE1, 3BE2, 3BE3, 3BE4, 3BE5, 3BE6, 3BE7, 3BE8, 3BE9, 3BEA, 3BEB, 3BEC, 3BED, 3BEE, 3BEF, 3BEG, 3BEH, 3BEI, 3BEJ, 3BEL, 3BEM, 3BEN, 3BEO, 3BEP, 3BEQ, 3BER, 3BES, 3BET, 3BEU, 3BEV, 3BEW, 3BEX, 3BEY, 3BEZ, 3BF0, 3BF1, 3BF2, 3BF3, 3BF4, 3BF5, 3BF6, 3BF7, 3BF8, 3BFA, 3BFB, 3BFC, 3BFD, 3BFE, 3BFF, 3BFG, 3BFH, 3BFI, 3BFJ, 3BFK, 3BFM, 3BFN, 3BFO, 3BFP, 3BFQ, 3BFR, 3BFT, 3BFU, 3BFV, 3BFW, 3BFX, 3BG0, 3BG1, 3BG2, 3BG3, 3BG4, 3BG5, 3BG6, 3BG7, 3BG8, 3BG9, 3BGA, 3BGB, 3BGC, 3BGD, 3BGE, 3BGF, 3BGG, 3BGH, 3BGI, 3BGJ, 3BGK, 3BGL, 3BGM, 3BGO, 3BGP, 3BGQ, 3BGR, 3BGS, 3BGT, 3BGU, 3BGV, 3BGW, 3BGX, 3BGY, 3BGZ, 3BH0, 3BH1, 3BH2, 3BH3, 3BH4, 3BH6, 3BH7, 3BH8, 3BH9, 3BHB, 3BHD, 3BHE, 3BHF, 3BHG, 3BHH, 3BHI, 3BHJ, 3BHK, 3BHL, 3BHM, 3BHN, 3BHO, 3BHP, 3BHQ, 3BHR, 3BHS, 3BHT, 3BHU, 3BHV, 3BHW, 3BHX, 3BHY, 3BI0, 3BI1, 3BI2, 3BI3, 3BI4, 3BI5, 3BI6, 3BI7, 3BI9, 3BIA, 3BIB, 3BIC, 3BID, 3BIE, 3BIF, 3BIG, 3BIH, 3BII, 3BIJ, 3BIK, 3BIL, 3BIM, 3BIN, 3BIO, 3BIP, 3BIQ, 3BIR, 3BIS, 3BIT, 3BIU, 3BIV, 3BIW, 3BIX, 3BIY, 3BIZ, 3BJ1, 3BJ2, 3BJ3, 3BJ4, 3BJ5, 3BJ6, 3BJ7, 3BJ8, 3BJ9, 3BJA, 3BJB, 3BJC, 3BJD, 3BJE, 3BJF, 3BJG, 3BJH, 3BJI, 3BJK, 3BJL, 3BJM, 3BJN, 3BJO, 3BJP, 3BJQ, 3BJR, 3BJS, 3BJT, 3BJU, 3BJV, 3BJW, 3BJX, 3BJY, 3BJZ, 3BK0, 3BK1, 3BK2, 3BK3, 3BK5, 3BK6, 3BK7, 3BK8, 3BK9, 3BKB, 3BKC, 3BKD, 3BKF, 3BKH, 3BKI, 3BKJ, 3BKK, 3BKL, 3BKM, 3BKN, 3BKP, 3BKQ, 3BKR, 3BKS, 3BKT, 3BKU, 3BKV, 3BKW, 3BKX, 3BKY, 3BKZ, 3BL0, 3BL1, 3BL2, 3BL3, 3BL4, 3BL5, 3BL6, 3BL7, 3BL8, 3BL9, 3BLA, 3BLB, 3BLC, 3BLD, 3BLE, 3BLF, 3BLG, 3BLH, 3BLI, 3BLJ, 3BLK, 3BLL, 3BLM, 3BLN, 3BLO, 3BLP, 3BLQ, 3BLR, 3BLS, 3BLT, 3BLU, 3BLV, 3BLW, 3BLX, 3BLY, 3BLZ, 3BM1, 3BM2, 3BM3, 3BM4, 3BM5, 3BM6, 3BM7, 3BM8, 3BM9, 3BMA, 3BMB, 3BMC, 3BMN, 3BMO, 3BMP, 3BMQ, 3BMV, 3BMW, 3BMX, 3BMY, 3BMZ, 3BN0, 3BN1, 3BN3, 3BN4, 3BN6, 3BN7, 3BN8, 3BN9, 3BNA, 3BNB, 3BNC, 3BND, 3BNE, 3BNF, 3BNG, 3BNH, 3BNI, 3BNJ, 3BNK, 3BNM, 3BNU, 3BNV, 3BNW, 3BNX, 3BNY, 3BNZ, 3BO5, 3BO6, 3BO7, 3BO8, 3BO9, 3BOA, 3BOB, 3BOC, 3BOD, 3BOE, 3BOF, 3BOH, 3BOI, 3BOJ, 3BOK, 3BOL, 3BOM, 3BON, 3BOO, 3BOP, 3BOQ, 3BOR, 3BOS, 3BOV, 3BOW, 3BOX, 3BP1, 3BP2, 3BP3, 3BP4, 3BP5, 3BP6, 3BP7, 3BP8, 3BP9, 3BPB, 3BPC, 3BPD, 3BPF, 3BPJ, 3BPK, 3BPL, 3BPM, 3BPN, 3BPO, 3BPP, 3BPQ, 3BPR, 3BPS, 3BPT, 3BPU, 3BPV, 3BPW, 3BPX, 3BPZ, 3BQ0, 3BQ1, 3BQ2, 3BQ3, 3BQ4, 3BQ5, 3BQ6, 3BQ7, 3BQ8, 3BQ9, 3BQA, 3BQB, 3BQC, 3BQD, 3BQE, 3BQF, 3BQG, 3BQH, 3BQI, 3BQJ, 3BQK, 3BQL, 3BQM, 3BQN, 3BQO, 3BQP, 3BQQ, 3BQR, 3BQT, 3BQU, 3BQV, 3BQW, 3BQX, 3BQY, 3BQZ, 3BR0, 3BR1, 3BR2, 3BR3, 3BR5, 3BR6, 3BR8, 3BR9, 3BRA, 3BRB, 3BRC, 3BRD, 3BRE, 3BRF, 3BRG, 3BRH, 3BRI, 3BRJ, 3BRK, 3BRL, 3BRM, 3BRN, 3BRO, 3BRP, 3BRQ, 3BRR, 3BRS, 3BRT, 3BRU, 3BRV, 3BRW, 3BRX, 3BRY, 3BRZ, 3BS0, 3BS1, 3BS2, 3BS3, 3BS4, 3BS5, 3BS6, 3BS7, 3BS8, 3BS9, 3BSA, 3BSC, 3BSD, 3BSE, 3BSF, 3BSG, 3BSH, 3BSM, 3BSQ, 3BSS, 3BSW, 3BSY, 3BSZ, 3BT0, 3BT1, 3BT2, 3BT3, 3BT4, 3BT5, 3BT6, 3BT8, 3BT9, 3BTA, 3BTC, 3BTD, 3BTE, 3BTF, 3BTG, 3BTH, 3BTI, 3BTJ, 3BTK, 3BTL, 3BTM, 3BTN, 3BTO, 3BTP, 3BTQ, 3BTR, 3BTS, 3BTT, 3BTU, 3BTV, 3BTW, 3BTX, 3BTY, 3BTZ, 3BU0, 3BU1, 3BU2, 3BU3, 3BU4, 3BU5, 3BU6, 3BU7, 3BU8, 3BU9, 3BUA, 3BUB, 3BUC, 3BUD, 3BUE, 3BUF, 3BUG, 3BUH, 3BUI, 3BUJ, 3BUK, 3BUL, 3BUO, 3BUP, 3BUQ, 3BUR, 3BUS, 3BUT, 3BUU, 3BUV, 3BUW, 3BUY, 3BUZ, 3BV0, 3BV2, 3BV3, 3BV4, 3BV6, 3BV7, 3BV8, 3BV9, 3BVA, 3BVB, 3BVC, 3BVD, 3BVE, 3BVF, 3BVG, 3BVH, 3BVI, 3BVJ, 3BVK, 3BVL, 3BVM, 3BVN, 3BVO, 3BVP, 3BVQ, 3BVS, 3BVT, 3BVU, 3BVV, 3BVW, 3BVX, 3BVZ, 3BW1, 3BW2, 3BW3, 3BW4, 3BW6, 3BW7, 3BW8, 3BW9, 3BWA, 3BWB, 3BWC, 3BWD, 3BWE, 3BWF, 3BWG, 3BWH, 3BWI, 3BWJ, 3BWK, 3BWL, 3BWM, 3BWN, 3BWO, 3BWQ, 3BWR, 3BWS, 3BWT, 3BWU, 3BWV, 3BWW, 3BWX, 3BWY, 3BWZ, 3BX1, 3BX4, 3BX5, 3BX7, 3BX8, 3BX9, 3BXA, 3BXB, 3BXC, 3BXD, 3BXE, 3BXF, 3BXG, 3BXH, 3BXI, 3BXJ, 3BXK, 3BXL, 3BXM, 3BXN, 3BXO, 3BXP, 3BXQ, 3BXR, 3BXS, 3BXU, 3BXV, 3BXW, 3BXX, 3BXY, 3BXZ, 3BY0, 3BY1, 3BY2, 3BY4, 3BY5, 3BY6, 3BY7, 3BY8, 3BY9, 3BYA, 3BYB, 3BYC, 3BYD, 3BYI, 3BYJ, 3BYK, 3BYL, 3BYM, 3BYN, 3BYO, 3BYP, 3BYQ, 3BYR, 3BYS, 3BYT, 3BYU, 3BYV, 3BYW, 3BYX, 3BYY, 3BYZ, 3BZ0, 3BZ1, 3BZ2, 3BZ3, 3BZ4, 3BZ5, 3BZ6, 3BZ7, 3BZ8, 3BZ9, 3BZA, 3BZB, 3BZC, 3BZD, 3BZE, 3BZF, 3BZG, 3BZH, 3BZI, 3BZJ, 3BZK, 3BZL, 3BZM, 3BZN, 3BZO, 3BZP, 3BZQ, 3BZR, 3BZS, 3BZT, 3BZU, 3BZV, 3BZW, 3BZX, 3BZY, 3BZZ, 3C00, 3C01, 3C02, 3C03, 3C04, 3C05, 3C06, 3C07, 3C08, 3C09, 3C0A, 3C0B, 3C0C, 3C0D, 3C0E, 3C0F, 3C0G, 3C0H, 3C0I, 3C0J, 3C0K, 3C0L, 3C0M, 3C0N, 3C0O, 3C0P, 3C0Q, 3C0R, 3C0S, 3C0T, 3C0U, 3C0V, 3C0W, 3C0X, 3C0Y, 3C0Z, 3C10, 3C11, 3C12, 3C13, 3C14, 3C15, 3C16, 3C17, 3C18, 3C19, 3C1A, 3C1B, 3C1C, 3C1D, 3C1E, 3C1F, 3C1G, 3C1H, 3C1I, 3C1J, 3C1K, 3C1L, 3C1M, 3C1N, 3C1O, 3C1Q, 3C1R, 3C1S, 3C1T, 3C1U, 3C1V, 3C1X, 3C1Y, 3C1Z, 3C20, 3C21, 3C22, 3C23, 3C24, 3C25, 3C26, 3C27, 3C28, 3C29, 3C2A, 3C2B, 3C2C, 3C2E, 3C2F, 3C2G, 3C2H, 3C2I, 3C2K, 3C2L, 3C2M, 3C2O, 3C2P, 3C2Q, 3C2R, 3C2S, 3C2T, 3C2U, 3C2V, 3C2W, 3C2X, 3C2Y, 3C30, 3C31, 3C32, 3C33, 3C34, 3C35, 3C36, 3C37, 3C38, 3C39, 3C3A, 3C3B, 3C3C, 3C3D, 3C3E, 3C3I, 3C3J, 3C3K, 3C3L, 3C3M, 3C3N, 3C3O, 3C3P, 3C3Q, 3C3R, 3C3S, 3C3T, 3C3U, 3C3V, 3C3W, 3C3X, 3C3Y, 3C41, 3C43, 3C45, 3C46, 3C48, 3C49, 3C4A, 3C4B, 3C4C, 3C4E, 3C4F, 3C4H, 3C4J, 3C4M, 3C4N, 3C4O, 3C4P, 3C4Q, 3C4R, 3C4S, 3C4T, 3C4U, 3C4V, 3C4W, 3C4X, 3C4Y, 3C4Z, 3C50, 3C51, 3C52, 3C56, 3C57, 3C58, 3C59, 3C5A, 3C5C, 3C5E, 3C5F, 3C5G, 3C5H, 3C5I, 3C5J, 3C5K, 3C5L, 3C5M, 3C5N, 3C5O, 3C5P, 3C5Q, 3C5R, 3C5S, 3C5T, 3C5U, 3C5V, 3C5W, 3C5X, 3C5Y, 3C5Z, 3C60, 3C61, 3C62, 3C63, 3C64, 3C65, 3C66, 3C6A, 3C6B, 3C6C, 3C6E, 3C6F, 3C6G, 3C6H, 3C6K, 3C6L, 3C6M, 3C6N, 3C6O, 3C6P, 3C6Q, 3C6S, 3C6T, 3C6U, 3C6V, 3C6W, 3C6X, 3C6Y, 3C6Z, 3C70, 3C71, 3C72, 3C73, 3C74, 3C75, 3C76, 3C77, 3C78, 3C79, 3C7A, 3C7C, 3C7D, 3C7E, 3C7F, 3C7G, 3C7H, 3C7I, 3C7J, 3C7K, 3C7L, 3C7M, 3C7N, 3C7O, 3C7P, 3C7Q, 3C7T, 3C7U, 3C7V, 3C7W, 3C7X, 3C7Y, 3C7Z, 3C80, 3C81, 3C82, 3C83, 3C84, 3C85, 3C86, 3C87, 3C88, 3C89, 3C8A, 3C8B, 3C8C, 3C8D, 3C8E, 3C8F, 3C8G, 3C8H, 3C8I, 3C8J, 3C8K, 3C8L, 3C8M, 3C8N, 3C8O, 3C8P, 3C8Q, 3C8R, 3C8S, 3C8T, 3C8U, 3C8V, 3C8W, 3C8X, 3C8Y, 3C8Z, 3C90, 3C94, 3C95, 3C96, 3C97, 3C98, 3C99, 3C9A, 3C9B, 3C9C, 3C9D, 3C9E, 3C9F, 3C9G, 3C9H, 3C9I, 3C9J, 3C9L, 3C9M, 3C9N, 3C9P, 3C9Q, 3C9R, 3C9S, 3C9T, 3C9U, 3C9W, 3C9X, 3C9Z, 3CA0, 3CA1, 3CA2, 3CA3, 3CA4, 3CA5, 3CA6, 3CA7, 3CA8, 3CA9, 3CAA, 3CAB, 3CAD, 3CAE, 3CAF, 3CAG, 3CAH, 3CAI, 3CAJ, 3CAK, 3CAL, 3CAM, 3CAN, 3CAO, 3CAP, 3CAQ, 3CAR, 3CAS, 3CAV, 3CAW, 3CAX, 3CAZ, 3CB0, 3CB2, 3CB3, 3CB4, 3CB5, 3CB6, 3CB7, 3CB8, 3CB9, 3CBB, 3CBC, 3CBD, 3CBE, 3CBF, 3CBG, 3CBH, 3CBI, 3CBJ, 3CBK, 3CBL, 3CBM, 3CBN, 3CBO, 3CBP, 3CBQ, 3CBR, 3CBS, 3CBT, 3CBU, 3CBW, 3CBX, 3CBY, 3CBZ, 3CC0, 3CC1, 3CC5, 3CC6, 3CC8, 3CC9, 3CCB, 3CCC, 3CCD, 3CCF, 3CCG, 3CCH, 3CCK, 3CCN, 3CCP, 3CCT, 3CCW, 3CCX, 3CCY, 3CCZ, 3CD0, 3CD1, 3CD2, 3CD3, 3CD4, 3CD5, 3CD7, 3CD8, 3CD9, 3CDA, 3CDB, 3CDC, 3CDD, 3CDE, 3CDF, 3CDG, 3CDH, 3CDI, 3CDJ, 3CDK, 3CDL, 3CDM, 3CDN, 3CDO, 3CDP, 3CDQ, 3CDR, 3CDS, 3CDT, 3CDU, 3CDV, 3CDW, 3CDX, 3CDY, 3CDZ, 3CE0, 3CE1, 3CE2, 3CE3, 3CE4, 3CE5, 3CE6, 3CE7, 3CE8, 3CE9, 3CEA, 3CEB, 3CEC, 3CED, 3CEG, 3CEH, 3CEI, 3CEJ, 3CEK, 3CEL, 3CEM, 3CEN, 3CEP, 3CEQ, 3CER, 3CES, 3CET, 3CEU, 3CEV, 3CEW, 3CEX, 3CEY, 3CEZ, 3CF0, 3CF1, 3CF2, 3CF3, 3CF4, 3CF6, 3CF8, 3CF9, 3CFA, 3CFB, 3CFC, 3CFD, 3CFE, 3CFF, 3CFH, 3CFI, 3CFJ, 3CFK, 3CFL, 3CFM, 3CFN, 3CFO, 3CFP, 3CFQ, 3CFR, 3CFS, 3CFT, 3CFU, 3CFV, 3CFW, 3CFX, 3CFY, 3CFZ, 3CG0, 3CG1, 3CG3, 3CG4, 3CG5, 3CG6, 3CG7, 3CG8, 3CG9, 3CGA, 3CGB, 3CGC, 3CGD, 3CGE, 3CGF, 3CGG, 3CGH, 3CGI, 3CGL, 3CGM, 3CGN, 3CGO, 3CGT, 3CGU, 3CGW, 3CGX, 3CGY, 3CGZ, 3CH0, 3CH1, 3CH2, 3CH3, 3CH4, 3CH5, 3CH6, 3CH7, 3CH8, 3CH9, 3CHB, 3CHC, 3CHD, 3CHE, 3CHF, 3CHG, 3CHH, 3CHI, 3CHJ, 3CHK, 3CHL, 3CHM, 3CHO, 3CHP, 3CHQ, 3CHR, 3CHS, 3CHT, 3CHU, 3CHV, 3CHW, 3CHX, 3CHY, 3CI0, 3CI1, 3CI3, 3CI4, 3CI5, 3CI6, 3CI7, 3CI8, 3CI9, 3CIA, 3CIB, 3CIC, 3CID, 3CIF, 3CIG, 3CIH, 3CII, 3CIJ, 3CIK, 3CIM, 3CIN, 3CIO, 3CIP, 3CIQ, 3CIR, 3CIS, 3CIT, 3CIU, 3CIV, 3CIW, 3CIX, 3CIZ, 3CJ0, 3CJ1, 3CJ2, 3CJ3, 3CJ4, 3CJ5, 3CJ7, 3CJ8, 3CJ9, 3CJA, 3CJB, 3CJC, 3CJD, 3CJE, 3CJF, 3CJG, 3CJH, 3CJJ, 3CJK, 3CJL, 3CJM, 3CJN, 3CJO, 3CJP, 3CJQ, 3CJR, 3CJS, 3CJT, 3CJW, 3CJX, 3CJY, 3CK0, 3CK1, 3CK2, 3CK4, 3CK5, 3CK6, 3CK7, 3CK8, 3CK9, 3CKA, 3CKB, 3CKC, 3CKD, 3CKE, 3CKF, 3CKG, 3CKH, 3CKI, 3CKJ, 3CKK, 3CKL, 3CKM, 3CKN, 3CKO, 3CKP, 3CKQ, 3CKR, 3CKS, 3CKT, 3CKU, 3CKV, 3CKW, 3CKX, 3CKY, 3CKZ, 3CL0, 3CL1, 3CL2, 3CL3, 3CL4, 3CL5, 3CL6, 3CL7, 3CL8, 3CL9, 3CLA, 3CLB, 3CLC, 3CLD, 3CLE, 3CLF, 3CLH, 3CLI, 3CLJ, 3CLK, 3CLL, 3CLM, 3CLN, 3CLO, 3CLP, 3CLQ, 3CLR, 3CLS, 3CLT, 3CLU, 3CLV, 3CLW, 3CLX, 3CLY, 3CLZ, 3CM0, 3CM1, 3CM2, 3CM3, 3CM4, 3CM5, 3CM6, 3CM7, 3CM8, 3CMB, 3CMC, 3CMD, 3CMF, 3CMG, 3CMI, 3CMJ, 3CML, 3CMM, 3CMN, 3CMO, 3CMP, 3CMQ, 3CMR, 3CMS, 3CMT, 3CMU, 3CMV, 3CMW, 3CMX, 3CMY, 3CMZ, 3CN0, 3CN1, 3CN2, 3CN3, 3CN4, 3CN5, 3CN6, 3CN7, 3CN8, 3CN9, 3CNA, 3CNB, 3CNC, 3CND, 3CNE, 3CNG, 3CNH, 3CNI, 3CNJ, 3CNK, 3CNL, 3CNM, 3CNN, 3CNO, 3CNP, 3CNQ, 3CNR, 3CNS, 3CNT, 3CNU, 3CNV, 3CNW, 3CNX, 3CNY, 3CNZ, 3CO0, 3CO1, 3CO2, 3CO3, 3CO4, 3CO5, 3CO6, 3CO7, 3CO8, 3CO9, 3COA, 3COB, 3COC, 3COD, 3COG, 3COH, 3COI, 3COJ, 3COK, 3COL, 3COM, 3CON, 3COO, 3COP, 3COQ, 3COR, 3COS, 3COT, 3COU, 3COV, 3COW, 3COX, 3COY, 3COZ, 3CP0, 3CP1, 3CP2, 3CP3, 3CP4, 3CP5, 3CP6, 3CP7, 3CP8, 3CP9, 3CPA, 3CPB, 3CPC, 3CPE, 3CPF, 3CPG, 3CPH, 3CPI, 3CPJ, 3CPK, 3CPL, 3CPM, 3CPO, 3CPP, 3CPQ, 3CPR, 3CPS, 3CPT, 3CPU, 3CPX, 3CPZ, 3CQ0, 3CQ1, 3CQ2, 3CQ3, 3CQ4, 3CQ5, 3CQ6, 3CQ8, 3CQ9, 3CQA, 3CQB, 3CQC, 3CQD, 3CQE, 3CQF, 3CQG, 3CQH, 3CQI, 3CQJ, 3CQK, 3CQL, 3CQN, 3CQO, 3CQP, 3CQQ, 3CQR, 3CQT, 3CQU, 3CQV, 3CQW, 3CQX, 3CQY, 3CQZ, 3CR0, 3CR2, 3CR3, 3CR4, 3CR5, 3CR6, 3CR7, 3CR8, 3CR9, 3CRA, 3CRB, 3CRC, 3CRG, 3CRH, 3CRI, 3CRJ, 3CRK, 3CRL, 3CRM, 3CRN, 3CRO, 3CRP, 3CRQ, 3CRR, 3CRT, 3CRU, 3CRV, 3CRW, 3CRX, 3CRY, 3CRZ, 3CS0, 3CS1, 3CS2, 3CS3, 3CS4, 3CS5, 3CS6, 3CS7, 3CS8, 3CS9, 3CSB, 3CSC, 3CSD, 3CSE, 3CSF, 3CSG, 3CSH, 3CSI, 3CSJ, 3CSK, 3CSL, 3CSM, 3CSN, 3CSO, 3CSP, 3CSQ, 3CSR, 3CSS, 3CST, 3CSU, 3CSV, 3CSW, 3CSX, 3CSY, 3CSZ, 3CT0, 3CT1, 3CT2, 3CT4, 3CT5, 3CT6, 3CT7, 3CT8, 3CT9, 3CTA, 3CTB, 3CTD, 3CTF, 3CTG, 3CTH, 3CTJ, 3CTK, 3CTL, 3CTM, 3CTO, 3CTP, 3CTQ, 3CTR, 3CTS, 3CTT, 3CTV, 3CTW, 3CTY, 3CTZ, 3CU0, 3CU1, 3CU2, 3CU3, 3CU4, 3CU5, 3CU7, 3CU8, 3CU9, 3CUC, 3CUE, 3CUF, 3CUG, 3CUH, 3CUI, 3CUJ, 3CUK, 3CUO, 3CUP, 3CUQ, 3CUR, 3CUS, 3CUT, 3CUU, 3CUV, 3CUW, 3CUX, 3CUY, 3CUZ, 3CV0, 3CV1, 3CV2, 3CV3, 3CV5, 3CV6, 3CV7, 3CV8, 3CV9, 3CVA, 3CVB, 3CVC, 3CVD, 3CVE, 3CVF, 3CVG, 3CVH, 3CVI, 3CVJ, 3CVK, 3CVL, 3CVM, 3CVN, 3CVO, 3CVP, 3CVQ, 3CVR, 3CVS, 3CVT, 3CVU, 3CVV, 3CVW, 3CVX, 3CVY, 3CVZ, 3CW0, 3CW1, 3CW2, 3CW3, 3CW4, 3CW7, 3CW8, 3CW9, 3CWA, 3CWB, 3CWC, 3CWD, 3CWE, 3CWF, 3CWG, 3CWI, 3CWJ, 3CWK, 3CWL, 3CWM, 3CWN, 3CWO, 3CWQ, 3CWR, 3CWS, 3CWT, 3CWU, 3CWV, 3CWW, 3CWX, 3CWY, 3CWZ, 3CX2, 3CX3, 3CX4, 3CX5, 3CX6, 3CX7, 3CX8, 3CX9, 3CXA, 3CXB, 3CXD, 3CXE, 3CXF, 3CXG, 3CXH, 3CXI, 3CXJ, 3CXK, 3CXL, 3CXM, 3CXN, 3CXO, 3CXP, 3CXQ, 3CXR, 3CXS, 3CXU, 3CXV, 3CXW, 3CXX, 3CXY, 3CXZ, 3CY0, 3CY1, 3CY2, 3CY3, 3CY4, 3CY5, 3CY6, 3CYE, 3CYF, 3CYG, 3CYH, 3CYI, 3CYJ, 3CYL, 3CYM, 3CYN, 3CYO, 3CYP, 3CYQ, 3CYR, 3CYT, 3CYU, 3CYV, 3CYW, 3CYX, 3CYY, 3CYZ, 3CZ0, 3CZ1, 3CZ2, 3CZ4, 3CZ5, 3CZ6, 3CZ7, 3CZ8, 3CZ9, 3CZA, 3CZB, 3CZC, 3CZD, 3CZE, 3CZF, 3CZG, 3CZH, 3CZJ, 3CZK, 3CZL, 3CZM, 3CZN, 3CZO, 3CZP, 3CZQ, 3CZR, 3CZS, 3CZT, 3CZU, 3CZV, 3CZX, 3CZY, 3CZZ, 3D00, 3D01, 3D02, 3D03, 3D04, 3D05, 3D06, 3D07, 3D08, 3D09, 3D0A, 3D0B, 3D0C, 3D0E, 3D0F, 3D0G, 3D0H, 3D0I, 3D0J, 3D0K, 3D0L, 3D0N, 3D0O, 3D0P, 3D0Q, 3D0R, 3D0S, 3D0T, 3D0V, 3D0W, 3D0Y, 3D0Z, 3D10, 3D11, 3D12, 3D14, 3D15, 3D17, 3D18, 3D19, 3D1A, 3D1B, 3D1C, 3D1D, 3D1E, 3D1F, 3D1G, 3D1H, 3D1I, 3D1J, 3D1K, 3D1L, 3D1M, 3D1N, 3D1O, 3D1P, 3D1Q, 3D1R, 3D1T, 3D1V, 3D1X, 3D1Y, 3D1Z, 3D20, 3D21, 3D22, 3D23, 3D24, 3D25, 3D26, 3D27, 3D28, 3D29, 3D2A, 3D2B, 3D2C, 3D2D, 3D2E, 3D2F, 3D2H, 3D2I, 3D2J, 3D2K, 3D2L, 3D2M, 3D2N, 3D2O, 3D2P, 3D2Q, 3D2R, 3D2T, 3D2U, 3D2W, 3D2Y, 3D2Z, 3D30, 3D31, 3D32, 3D33, 3D34, 3D36, 3D37, 3D38, 3D39, 3D3A, 3D3B, 3D3C, 3D3D, 3D3E, 3D3F, 3D3H, 3D3I, 3D3J, 3D3K, 3D3L, 3D3M, 3D3N, 3D3O, 3D3P, 3D3Q, 3D3R, 3D3S, 3D3T, 3D3U, 3D3V, 3D3W, 3D3X, 3D3Y, 3D3Z, 3D40, 3D41, 3D42, 3D43, 3D44, 3D45, 3D46, 3D47, 3D48, 3D49, 3D4A, 3D4B, 3D4C, 3D4D, 3D4E, 3D4F, 3D4G, 3D4I, 3D4J, 3D4K, 3D4L, 3D4M, 3D4N, 3D4O, 3D4P, 3D4Q, 3D4R, 3D4S, 3D4T, 3D4U, 3D4V, 3D4W, 3D4X, 3D4Y, 3D4Z, 3D50, 3D51, 3D52, 3D53, 3D54, 3D55, 3D57, 3D59, 3D5E, 3D5F, 3D5G, 3D5H, 3D5I, 3D5J, 3D5K, 3D5L, 3D5M, 3D5N, 3D5O, 3D5P, 3D5Q, 3D5R, 3D5S, 3D5T, 3D5U, 3D5V, 3D5W, 3D5X, 3D5Y, 3D5Z, 3D60, 3D61, 3D62, 3D63, 3D64, 3D65, 3D66, 3D67, 3D68, 3D69, 3D6A, 3D6B, 3D6C, 3D6D, 3D6E, 3D6F, 3D6G, 3D6H, 3D6I, 3D6J, 3D6K, 3D6L, 3D6M, 3D6N, 3D6O, 3D6P, 3D6Q, 3D6R, 3D6S, 3D6T, 3D6U, 3D6V, 3D6W, 3D6X, 3D6Y, 3D6Z, 3D70, 3D71, 3D72, 3D73, 3D74, 3D75, 3D76, 3D77, 3D78, 3D79, 3D7A, 3D7B, 3D7C, 3D7D, 3D7E, 3D7F, 3D7G, 3D7H, 3D7I, 3D7J, 3D7K, 3D7L, 3D7M, 3D7N, 3D7O, 3D7P, 3D7Q, 3D7R, 3D7S, 3D7T, 3D7U, 3D7V, 3D7W, 3D7Z, 3D80, 3D81, 3D82, 3D83, 3D84, 3D85, 3D87, 3D89, 3D8A, 3D8B, 3D8C, 3D8D, 3D8E, 3D8F, 3D8G, 3D8H, 3D8K, 3D8L, 3D8M, 3D8N, 3D8P, 3D8R, 3D8S, 3D8T, 3D8U, 3D8V, 3D8W, 3D8X, 3D8Y, 3D8Z, 3D90, 3D91, 3D92, 3D93, 3D94, 3D95, 3D96, 3D97, 3D98, 3D9A, 3D9B, 3D9C, 3D9D, 3D9E, 3D9F, 3D9G, 3D9H, 3D9I, 3D9J, 3D9K, 3D9L, 3D9M, 3D9N, 3D9O, 3D9P, 3D9Q, 3D9R, 3D9S, 3D9T, 3D9U, 3D9V, 3D9W, 3D9X, 3D9Y, 3D9Z, 3DA0, 3DA1, 3DA2, 3DA3, 3DA4, 3DA5, 3DA6, 3DA7, 3DA8, 3DA9, 3DAA, 3DAB, 3DAC, 3DAD, 3DAE, 3DAF, 3DAG, 3DAH, 3DAI, 3DAJ, 3DAK, 3DAL, 3DAM, 3DAN, 3DAO, 3DAP, 3DAQ, 3DAR, 3DAS, 3DAT, 3DAU, 3DAV, 3DAW, 3DAX, 3DAY, 3DAZ, 3DB0, 3DB1, 3DB2, 3DB3, 3DB4, 3DB5, 3DB6, 3DB7, 3DB8, 3DB9, 3DBA, 3DBC, 3DBD, 3DBE, 3DBF, 3DBG, 3DBH, 3DBI, 3DBJ, 3DBK, 3DBL, 3DBM, 3DBN, 3DBO, 3DBP, 3DBQ, 3DBR, 3DBS, 3DBU, 3DBV, 3DBX, 3DBY, 3DBZ, 3DC0, 3DC1, 3DC2, 3DC3, 3DC4, 3DC5, 3DC6, 3DC7, 3DC8, 3DC9, 3DCA, 3DCB, 3DCC, 3DCD, 3DCF, 3DCG, 3DCI, 3DCJ, 3DCK, 3DCL, 3DCM, 3DCN, 3DCP, 3DCQ, 3DCR, 3DCS, 3DCT, 3DCU, 3DCV, 3DCW, 3DCX, 3DCY, 3DCZ, 3DD0, 3DD1, 3DD3, 3DD4, 3DD5, 3DD6, 3DD7, 3DD8, 3DD9, 3DDA, 3DDB, 3DDC, 3DDD, 3DDE, 3DDF, 3DDG, 3DDH, 3DDI, 3DDJ, 3DDK, 3DDL, 3DDM, 3DDN, 3DDO, 3DDP, 3DDQ, 3DDR, 3DDS, 3DDT, 3DDU, 3DDV, 3DDW, 3DDY, 3DDZ, 3DE0, 3DE1, 3DE2, 3DE3, 3DE4, 3DE5, 3DE6, 3DE7, 3DE8, 3DE9, 3DEA, 3DEB, 3DEC, 3DED, 3DEE, 3DEF, 3DEH, 3DEI, 3DEJ, 3DEK, 3DEL, 3DEM, 3DEN, 3DEO, 3DEP, 3DEQ, 3DER, 3DES, 3DET, 3DEU, 3DEV, 3DEW, 3DEX, 3DEY, 3DEZ, 3DF0, 3DF6, 3DF7, 3DF8, 3DF9, 3DFA, 3DFC, 3DFE, 3DFF, 3DFG, 3DFH, 3DFI, 3DFJ, 3DFK, 3DFL, 3DFM, 3DFN, 3DFO, 3DFP, 3DFQ, 3DFR, 3DFS, 3DFT, 3DFU, 3DFV, 3DFX, 3DFY, 3DFZ, 3DG1, 3DG3, 3DG6, 3DG7, 3DG8, 3DG9, 3DGA, 3DGB, 3DGC, 3DGD, 3DGE, 3DGF, 3DGG, 3DGH, 3DGI, 3DGJ, 3DGK, 3DGL, 3DGM, 3DGN, 3DGO, 3DGP, 3DGQ, 3DGR, 3DGS, 3DGT, 3DGV, 3DGY, 3DGZ, 3DH0, 3DH1, 3DH2, 3DH4, 3DH5, 3DH6, 3DH7, 3DH8, 3DH9, 3DHA, 3DHB, 3DHC, 3DHD, 3DHE, 3DHF, 3DHG, 3DHH, 3DHI, 3DHJ, 3DHK, 3DHM, 3DHN, 3DHO, 3DHP, 3DHQ, 3DHR, 3DHT, 3DHU, 3DHV, 3DHW, 3DHX, 3DHY, 3DHZ, 3DI0, 3DI1, 3DI2, 3DI3, 3DI4, 3DI5, 3DI6, 3DI7, 3DI8, 3DI9, 3DIB, 3DIC, 3DID, 3DIE, 3DIF, 3DIH, 3DIN, 3DIP, 3DIT, 3DIV, 3DIW, 3DJ1, 3DJ3, 3DJ4, 3DJ5, 3DJ6, 3DJ7, 3DJ8, 3DJ9, 3DJA, 3DJB, 3DJC, 3DJD, 3DJE, 3DJF, 3DJG, 3DJH, 3DJI, 3DJJ, 3DJK, 3DJL, 3DJM, 3DJN, 3DJO, 3DJP, 3DJQ, 3DJR, 3DJS, 3DJT, 3DJU, 3DJV, 3DJW, 3DJX, 3DJY, 3DJZ, 3DK0, 3DK1, 3DK2, 3DK3, 3DK4, 3DK5, 3DK6, 3DK7, 3DK8, 3DK9, 3DKA, 3DKB, 3DKC, 3DKD, 3DKE, 3DKF, 3DKG, 3DKH, 3DKI, 3DKJ, 3DKK, 3DKL, 3DKM, 3DKO, 3DKP, 3DKQ, 3DKR, 3DKS, 3DKT, 3DKU, 3DKV, 3DKW, 3DKX, 3DKY, 3DKZ, 3DL0, 3DL1, 3DL2, 3DL3, 3DL4, 3DL5, 3DL6, 3DL7, 3DL8, 3DL9, 3DLA, 3DLB, 3DLC, 3DLD, 3DLE, 3DLG, 3DLH, 3DLI, 3DLJ, 3DLK, 3DLM, 3DLN, 3DLO, 3DLP, 3DLQ, 3DLR, 3DLS, 3DLT, 3DLU, 3DLV, 3DLW, 3DLX, 3DLZ, 3DM0, 3DM1, 3DM2, 3DM3, 3DM5, 3DM6, 3DM7, 3DM8, 3DM9, 3DMB, 3DMC, 3DMD, 3DME, 3DMF, 3DMG, 3DMH, 3DMI, 3DMJ, 3DMK, 3DML, 3DMM, 3DMN, 3DMO, 3DMP, 3DMQ, 3DMR, 3DMS, 3DMT, 3DMU, 3DMV, 3DMX, 3DMY, 3DMZ, 3DN0, 3DN1, 3DN2, 3DN3, 3DN4, 3DN5, 3DN6, 3DN7, 3DN8, 3DN9, 3DNA, 3DNB, 3DNC, 3DND, 3DNE, 3DNF, 3DNG, 3DNH, 3DNI, 3DNJ, 3DNK, 3DNM, 3DNP, 3DNS, 3DNT, 3DNU, 3DNV, 3DNX, 3DNZ, 3DO0, 3DO1, 3DO2, 3DO3, 3DO4, 3DO5, 3DO6, 3DO7, 3DO8, 3DO9, 3DOA, 3DOB, 3DOC, 3DOD, 3DOE, 3DOF, 3DOG, 3DOH, 3DOI, 3DOJ, 3DOK, 3DOL, 3DOM, 3DON, 3DOO, 3DOP, 3DOR, 3DOS, 3DOU, 3DOW, 3DOX, 3DOY, 3DOZ, 3DP0, 3DP1, 3DP2, 3DP3, 3DP4, 3DP5, 3DP6, 3DP7, 3DP8, 3DP9, 3DPA, 3DPB, 3DPC, 3DPD, 3DPE, 3DPF, 3DPG, 3DPH, 3DPI, 3DPJ, 3DPK, 3DPL, 3DPM, 3DPN, 3DPO, 3DPP, 3DPQ, 3DPS, 3DPT, 3DPU, 3DPW, 3DPX, 3DPY, 3DPZ, 3DQ0, 3DQ1, 3DQ2, 3DQ3, 3DQ4, 3DQ5, 3DQ6, 3DQ7, 3DQ8, 3DQ9, 3DQA, 3DQB, 3DQC, 3DQD, 3DQE, 3DQF, 3DQG, 3DQH, 3DQI, 3DQJ, 3DQK, 3DQL, 3DQM, 3DQN, 3DQO, 3DQP, 3DQQ, 3DQR, 3DQS, 3DQT, 3DQU, 3DQV, 3DQW, 3DQX, 3DQY, 3DQZ, 3DR0, 3DR1, 3DR2, 3DR3, 3DR4, 3DR5, 3DR6, 3DR7, 3DR8, 3DR9, 3DRA, 3DRB, 3DRC, 3DRD, 3DRE, 3DRF, 3DRG, 3DRH, 3DRI, 3DRJ, 3DRK, 3DRM, 3DRN, 3DRO, 3DRP, 3DRQ, 3DRR, 3DRS, 3DRT, 3DRU, 3DRW, 3DRX, 3DRY, 3DRZ, 3DS0, 3DS1, 3DS2, 3DS3, 3DS4, 3DS5, 3DS6, 3DS8, 3DS9, 3DSB, 3DSC, 3DSD, 3DSE, 3DSF, 3DSG, 3DSH, 3DSI, 3DSJ, 3DSK, 3DSL, 3DSM, 3DSN, 3DSO, 3DSP, 3DSQ, 3DSR, 3DSS, 3DST, 3DSU, 3DSV, 3DSW, 3DSX, 3DSY, 3DSZ, 3DT0, 3DT1, 3DT2, 3DT3, 3DT4, 3DT5, 3DT6, 3DT7, 3DT8, 3DT9, 3DTA, 3DTB, 3DTC, 3DTD, 3DTE, 3DTF, 3DTG, 3DTI, 3DTJ, 3DTK, 3DTM, 3DTN, 3DTO, 3DTQ, 3DTR, 3DTS, 3DTT, 3DTU, 3DTV, 3DTW, 3DTX, 3DTY, 3DTZ, 3DU0, 3DU1, 3DU2, 3DU3, 3DU4, 3DU5, 3DU6, 3DU7, 3DU8, 3DUE, 3DUF, 3DUG, 3DUH, 3DUI, 3DUK, 3DUL, 3DUP, 3DUQ, 3DUR, 3DUS, 3DUT, 3DUU, 3DUV, 3DUW, 3DUX, 3DUY, 3DUZ, 3DV0, 3DV1, 3DV2, 3DV3, 3DV4, 3DV5, 3DV6, 3DV7, 3DV8, 3DV9, 3DVA, 3DVB, 3DVC, 3DVD, 3DVE, 3DVF, 3DVG, 3DVH, 3DVI, 3DVJ, 3DVK, 3DVL, 3DVM, 3DVN, 3DVO, 3DVP, 3DVQ, 3DVR, 3DVS, 3DVT, 3DVU, 3DVW, 3DVX, 3DW0, 3DW1, 3DW3, 3DW8, 3DW9, 3DWA, 3DWB, 3DWC, 3DWD, 3DWE, 3DWF, 3DWG, 3DWH, 3DWI, 3DWJ, 3DWK, 3DWL, 3DWM, 3DWN, 3DWO, 3DWP, 3DWQ, 3DWR, 3DWS, 3DWT, 3DWV, 3DWY, 3DWZ, 3DX0, 3DX1, 3DX2, 3DX3, 3DX4, 3DX5, 3DX6, 3DX7, 3DX8, 3DX9, 3DXA, 3DXB, 3DXC, 3DXD, 3DXE, 3DXF, 3DXG, 3DXH, 3DXI, 3DXJ, 3DXK, 3DXL, 3DXM, 3DXN, 3DXO, 3DXP, 3DXQ, 3DXR, 3DXS, 3DXT, 3DXU, 3DXV, 3DXW, 3DXX, 3DXY, 3DXZ, 3DY0, 3DY3, 3DY4, 3DY5, 3DY6, 3DY7, 3DY8, 3DY9, 3DYA, 3DYB, 3DYC, 3DYD, 3DYE, 3DYF, 3DYG, 3DYH, 3DYI, 3DYJ, 3DYL, 3DYM, 3DYN, 3DYO, 3DYP, 3DYQ, 3DYR, 3DYS, 3DYT, 3DYU, 3DYV, 3DZ1, 3DZ2, 3DZ3, 3DZ4, 3DZ5, 3DZ6, 3DZ7, 3DZ8, 3DZA, 3DZB, 3DZC, 3DZD, 3DZE, 3DZF, 3DZG, 3DZH, 3DZI, 3DZJ, 3DZK, 3DZL, 3DZM, 3DZN, 3DZO, 3DZP, 3DZQ, 3DZR, 3DZT, 3DZU, 3DZV, 3DZW, 3DZX, 3DZY, 3DZZ, 3E00, 3E01, 3E02, 3E03, 3E04, 3E05, 3E07, 3E08, 3E0A, 3E0B, 3E0C, 3E0D, 3E0E, 3E0F, 3E0G, 3E0H, 3E0I, 3E0J, 3E0K, 3E0L, 3E0M, 3E0N, 3E0O, 3E0P, 3E0Q, 3E0R, 3E0S, 3E0U, 3E0V, 3E0W, 3E0X, 3E0Y, 3E0Z, 3E10, 3E11, 3E12, 3E13, 3E15, 3E16, 3E17, 3E18, 3E19, 3E1E, 3E1F, 3E1G, 3E1H, 3E1I, 3E1J, 3E1K, 3E1L, 3E1M, 3E1N, 3E1O, 3E1P, 3E1Q, 3E1R, 3E1S, 3E1T, 3E1U, 3E1V, 3E1W, 3E1X, 3E1Y, 3E1Z, 3E20, 3E21, 3E22, 3E23, 3E24, 3E25, 3E26, 3E27, 3E28, 3E29, 3E2A, 3E2B, 3E2C, 3E2D, 3E2F, 3E2H, 3E2I, 3E2J, 3E2K, 3E2L, 3E2M, 3E2N, 3E2P, 3E2Q, 3E2R, 3E2S, 3E2T, 3E2U, 3E2V, 3E2W, 3E2X, 3E2Y, 3E2Z, 3E30, 3E31, 3E32, 3E33, 3E34, 3E35, 3E37, 3E38, 3E39, 3E3A, 3E3B, 3E3C, 3E3D, 3E3E, 3E3F, 3E3G, 3E3H, 3E3I, 3E3K, 3E3L, 3E3M, 3E3N, 3E3O, 3E3P, 3E3Q, 3E3R, 3E3S, 3E3T, 3E3U, 3E3V, 3E3X, 3E3Y, 3E3Z, 3E40, 3E41, 3E42, 3E43, 3E44, 3E45, 3E46, 3E47, 3E48, 3E49, 3E4A, 3E4B, 3E4C, 3E4D, 3E4E, 3E4F, 3E4G, 3E4H, 3E4N, 3E4O, 3E4P, 3E4Q, 3E4R, 3E4U, 3E4V, 3E4W, 3E4Y, 3E4Z, 3E50, 3E51, 3E53, 3E54, 3E55, 3E56, 3E57, 3E58, 3E59, 3E5A, 3E5B, 3E5D, 3E5H, 3E5I, 3E5J, 3E5K, 3E5L, 3E5M, 3E5N, 3E5O, 3E5P, 3E5Q, 3E5R, 3E5S, 3E5T, 3E5U, 3E5V, 3E5W, 3E5X, 3E5Y, 3E5Z, 3E60, 3E61, 3E62, 3E63, 3E64, 3E65, 3E66, 3E67, 3E68, 3E6A, 3E6B, 3E6C, 3E6D, 3E6E, 3E6F, 3E6G, 3E6H, 3E6I, 3E6J, 3E6K, 3E6L, 3E6M, 3E6N, 3E6O, 3E6P, 3E6Q, 3E6R, 3E6S, 3E6T, 3E6U, 3E6V, 3E6Y, 3E6Z, 3E70, 3E73, 3E74, 3E76, 3E77, 3E78, 3E79, 3E7A, 3E7B, 3E7C, 3E7D, 3E7E, 3E7F, 3E7G, 3E7H, 3E7I, 3E7J, 3E7K, 3E7L, 3E7M, 3E7N, 3E7O, 3E7P, 3E7Q, 3E7R, 3E7S, 3E7T, 3E7U, 3E7V, 3E7W, 3E7X, 3E7Y, 3E7Z, 3E80, 3E81, 3E82, 3E83, 3E84, 3E85, 3E86, 3E87, 3E88, 3E89, 3E8B, 3E8C, 3E8D, 3E8E, 3E8F, 3E8G, 3E8H, 3E8J, 3E8L, 3E8M, 3E8N, 3E8O, 3E8P, 3E8Q, 3E8R, 3E8S, 3E8T, 3E8U, 3E8V, 3E8W, 3E8X, 3E8Y, 3E8Z, 3E90, 3E91, 3E92, 3E93, 3E94, 3E95, 3E96, 3E97, 3E98, 3E99, 3E9A, 3E9B, 3E9C, 3E9D, 3E9E, 3E9F, 3E9G, 3E9H, 3E9I, 3E9J, 3E9K, 3E9L, 3E9M, 3E9N, 3E9O, 3E9P, 3E9Q, 3E9R, 3E9S, 3E9T, 3E9U, 3E9V, 3E9W, 3E9X, 3E9Y, 3E9Z, 3EA0, 3EA1, 3EA2, 3EA3, 3EA4, 3EA5, 3EA6, 3EA7, 3EA8, 3EA9, 3EAA, 3EAB, 3EAC, 3EAD, 3EAE, 3EAF, 3EAG, 3EAH, 3EAI, 3EAJ, 3EAK, 3EAM, 3EAN, 3EAO, 3EAP, 3EAQ, 3EAR, 3EAS, 3EAT, 3EAU, 3EAW, 3EAX, 3EAY, 3EAZ, 3EB0, 3EB1, 3EB2, 3EB3, 3EB4, 3EB5, 3EB6, 3EB7, 3EB8, 3EB9, 3EBA, 3EBB, 3EBC, 3EBD, 3EBE, 3EBF, 3EBG, 3EBH, 3EBI, 3EBJ, 3EBK, 3EBL, 3EBM, 3EBN, 3EBO, 3EBP, 3EBQ, 3EBR, 3EBS, 3EBT, 3EBU, 3EBV, 3EBW, 3EBX, 3EBY, 3EBZ, 3EC0, 3EC1, 3EC2, 3EC3, 3EC4, 3EC5, 3EC6, 3EC7, 3EC8, 3EC9, 3ECA, 3ECB, 3ECC, 3ECD, 3ECF, 3ECG, 3ECH, 3ECI, 3ECJ, 3ECK, 3ECL, 3ECM, 3ECN, 3ECO, 3ECP, 3ECQ, 3ECR, 3ECS, 3ECT, 3ECU, 3ECV, 3ECW, 3ECX, 3ECY, 3ECZ, 3ED0, 3ED1, 3ED3, 3ED4, 3ED5, 3ED7, 3ED8, 3ED9, 3EDA, 3EDB, 3EDC, 3EDD, 3EDE, 3EDF, 3EDG, 3EDH, 3EDI, 3EDJ, 3EDK, 3EDM, 3EDN, 3EDO, 3EDP, 3EDQ, 3EDR, 3EDT, 3EDU, 3EDV, 3EDW, 3EDX, 3EDY, 3EDZ, 3EE0, 3EE1, 3EE2, 3EE3, 3EE4, 3EE5, 3EE6, 3EE7, 3EE8, 3EE9, 3EEA, 3EEB, 3EEC, 3EED, 3EEE, 3EEF, 3EEG, 3EEH, 3EEI, 3EEJ, 3EEK, 3EEL, 3EEM, 3EEO, 3EEQ, 3EER, 3EES, 3EET, 3EEU, 3EEV, 3EEX, 3EEY, 3EEZ, 3EF0, 3EF1, 3EF2, 3EF3, 3EF4, 3EF5, 3EF6, 3EF7, 3EF8, 3EF9, 3EFA, 3EFB, 3EFC, 3EFD, 3EFE, 3EFF, 3EFG, 3EFH, 3EFI, 3EFJ, 3EFK, 3EFL, 3EFM, 3EFO, 3EFP, 3EFQ, 3EFR, 3EFS, 3EFT, 3EFU, 3EFV, 3EFW, 3EFX, 3EFY, 3EFZ, 3EG0, 3EG1, 3EG2, 3EG3, 3EG4, 3EG5, 3EG6, 3EG7, 3EG9, 3EGA, 3EGB, 3EGC, 3EGD, 3EGE, 3EGG, 3EGH, 3EGI, 3EGJ, 3EGK, 3EGL, 3EGM, 3EGN, 3EGO, 3EGQ, 3EGR, 3EGS, 3EGT, 3EGU, 3EGV, 3EGW, 3EGX, 3EGY, 3EH0, 3EH1, 3EH2, 3EH3, 3EH4, 3EH5, 3EH7, 3EH8, 3EH9, 3EHA, 3EHB, 3EHC, 3EHD, 3EHE, 3EHF, 3EHG, 3EHH, 3EHI, 3EHJ, 3EHK, 3EHM, 3EHN, 3EHQ, 3EHR, 3EHS, 3EHT, 3EHU, 3EHV, 3EHW, 3EHX, 3EHY, 3EHZ, 3EI0, 3EI1, 3EI2, 3EI3, 3EI4, 3EI5, 3EI6, 3EI7, 3EI8, 3EI9, 3EIA, 3EIB, 3EIC, 3EID, 3EIE, 3EIF, 3EIG, 3EIH, 3EII, 3EIJ, 3EIK, 3EIL, 3EIM, 3EIN, 3EIO, 3EIP, 3EIQ, 3EIR, 3EIS, 3EIT, 3EIU, 3EIV, 3EIW, 3EIX, 3EIY, 3EIZ, 3EJ0, 3EJ1, 3EJ2, 3EJ3, 3EJ5, 3EJ6, 3EJ7, 3EJ8, 3EJ9, 3EJA, 3EJB, 3EJC, 3EJD, 3EJE, 3EJF, 3EJG, 3EJH, 3EJI, 3EJJ, 3EJK, 3EJL, 3EJM, 3EJN, 3EJO, 3EJP, 3EJQ, 3EJR, 3EJS, 3EJT, 3EJU, 3EJV, 3EJW, 3EJX, 3EJY, 3EJZ, 3EK1, 3EK2, 3EK3, 3EK4, 3EK5, 3EK6, 3EK7, 3EK8, 3EK9, 3EKA, 3EKB, 3EKC, 3EKD, 3EKE, 3EKF, 3EKG, 3EKH, 3EKI, 3EKJ, 3EKK, 3EKL, 3EKM, 3EKN, 3EKO, 3EKP, 3EKQ, 3EKR, 3EKS, 3EKT, 3EKU, 3EKV, 3EKW, 3EKX, 3EKY, 3EKZ, 3EL0, 3EL1, 3EL2, 3EL3, 3EL4, 3EL5, 3EL6, 3EL7, 3EL8, 3EL9, 3ELA, 3ELB, 3ELC, 3ELD, 3ELE, 3ELF, 3ELG, 3ELH, 3ELI, 3ELJ, 3ELK, 3ELL, 3ELM, 3ELN, 3ELO, 3ELP, 3ELQ, 3ELS, 3ELU, 3ELV, 3ELW, 3ELX, 3ELY, 3ELZ, 3EM0, 3EM1, 3EM2, 3EM3, 3EM4, 3EM6, 3EMB, 3EMC, 3EMD, 3EMF, 3EMG, 3EMH, 3EMI, 3EMJ, 3EMK, 3EML, 3EMM, 3EMN, 3EMO, 3EMP, 3EMQ, 3EMR, 3EMS, 3EMT, 3EMU, 3EMV, 3EMW, 3EMX, 3EMY, 3EMZ, 3EN0, 3EN1, 3EN2, 3EN3, 3EN4, 3EN5, 3EN6, 3EN7, 3EN8, 3EN9, 3ENA, 3ENB, 3ENC, 3ENE, 3ENG, 3ENH, 3ENI, 3ENJ, 3ENK, 3ENL, 3ENM, 3ENN, 3ENO, 3ENP, 3ENQ, 3ENR, 3ENS, 3ENT, 3ENU, 3ENV, 3ENW, 3ENZ, 3EO0, 3EO1, 3EO2, 3EO3, 3EO4, 3EO5, 3EO6, 3EO7, 3EO8, 3EO9, 3EOA, 3EOB, 3EOC, 3EOD, 3EOE, 3EOF, 3EOI, 3EOJ, 3EOK, 3EOL, 3EOM, 3EON, 3EOO, 3EOP, 3EOQ, 3EOR, 3EOS, 3EOT, 3EOU, 3EOV, 3EOX, 3EOY, 3EOZ, 3EP0, 3EP1, 3EP3, 3EP4, 3EP5, 3EP6, 3EP7, 3EP8, 3EP9, 3EPA, 3EPB, 3EPE, 3EPG, 3EPI, 3EPM, 3EPN, 3EPO, 3EPP, 3EPR, 3EPS, 3EPT, 3EPU, 3EPV, 3EPW, 3EPX, 3EPY, 3EPZ, 3EQ0, 3EQ1, 3EQ2, 3EQ5, 3EQ6, 3EQ7, 3EQ8, 3EQ9, 3EQA, 3EQB, 3EQC, 3EQD, 3EQE, 3EQF, 3EQG, 3EQH, 3EQI, 3EQL, 3EQM, 3EQN, 3EQO, 3EQP, 3EQQ, 3EQR, 3EQS, 3EQU, 3EQV, 3EQW, 3EQX, 3EQY, 3EQZ, 3ER0, 3ER3, 3ER5, 3ER6, 3ER7, 3ER8, 3ERA, 3ERB, 3ERC, 3ERD, 3ERE, 3ERF, 3ERG, 3ERH, 3ERI, 3ERJ, 3ERK, 3ERM, 3ERN, 3ERO, 3ERP, 3ERQ, 3ERR, 3ERS, 3ERT, 3ERU, 3ERV, 3ERW, 3ERX, 3ERY, 3ERZ, 3ES0, 3ES1, 3ES2, 3ES3, 3ES4, 3ES6, 3ES7, 3ES8, 3ES9, 3ESA, 3ESB, 3ESC, 3ESD, 3ESF, 3ESG, 3ESH, 3ESI, 3ESJ, 3ESK, 3ESL, 3ESM, 3ESN, 3ESO, 3ESP, 3ESQ, 3ESR, 3ESS, 3EST, 3ESU, 3ESV, 3ESW, 3ESX, 3ESY, 3ESZ, 3ET0, 3ET1, 3ET2, 3ET3, 3ET4, 3ET5, 3ET6, 3ET7, 3ET8, 3ET9, 3ETA, 3ETB, 3ETC, 3ETD, 3ETE, 3ETF, 3ETG, 3ETH, 3ETI, 3ETJ, 3ETL, 3ETM, 3ETN, 3ETO, 3ETP, 3ETQ, 3ETR, 3ETS, 3ETT, 3ETU, 3ETV, 3ETW, 3ETX, 3ETY, 3ETZ, 3EU0, 3EU1, 3EU3, 3EU4, 3EU5, 3EU7, 3EU8, 3EU9, 3EUA, 3EUB, 3EUC, 3EUD, 3EUE, 3EUF, 3EUG, 3EUH, 3EUI, 3EUJ, 3EUK, 3EUL, 3EUM, 3EUN, 3EUO, 3EUP, 3EUQ, 3EUR, 3EUS, 3EUT, 3EUU, 3EUV, 3EUW, 3EUX, 3EUY, 3EUZ, 3EV0, 3EV1, 3EV2, 3EV3, 3EV4, 3EV5, 3EV6, 3EVA, 3EVB, 3EVC, 3EVD, 3EVE, 3EVF, 3EVG, 3EVI, 3EVJ, 3EVK, 3EVM, 3EVN, 3EVO, 3EVP, 3EVQ, 3EVR, 3EVS, 3EVT, 3EVU, 3EVV, 3EVW, 3EVX, 3EVY, 3EVZ, 3EW0, 3EW1, 3EW2, 3EW3, 3EW5, 3EW7, 3EW8, 3EW9, 3EWA, 3EWB, 3EWC, 3EWD, 3EWE, 3EWF, 3EWG, 3EWH, 3EWI, 3EWJ, 3EWK, 3EWL, 3EWM, 3EWN, 3EWO, 3EWP, 3EWQ, 3EWR, 3EWS, 3EWT, 3EWU, 3EWV, 3EWW, 3EWX, 3EWY, 3EWZ, 3EX1, 3EX2, 3EX3, 3EX4, 3EX6, 3EX8, 3EX9, 3EXA, 3EXB, 3EXC, 3EXD, 3EXE, 3EXF, 3EXG, 3EXH, 3EXI, 3EXJ, 3EXL, 3EXM, 3EXN, 3EXO, 3EXQ, 3EXR, 3EXS, 3EXT, 3EXU, 3EXV, 3EXW, 3EXX, 3EXY, 3EXZ, 3EY0, 3EY1, 3EY2, 3EY3, 3EY4, 3EY5, 3EY6, 3EY7, 3EY8, 3EY9, 3EYA, 3EYB, 3EYC, 3EYD, 3EYE, 3EYF, 3EYG, 3EYH, 3EYI, 3EYJ, 3EYK, 3EYL, 3EYM, 3EYO, 3EYP, 3EYQ, 3EYS, 3EYT, 3EYU, 3EYV, 3EYW, 3EYX, 3EYY, 3EYZ, 3EZ0, 3EZ1, 3EZ2, 3EZ3, 3EZ4, 3EZ5, 3EZ6, 3EZ7, 3EZ8, 3EZ9, 3EZF, 3EZG, 3EZH, 3EZI, 3EZJ, 3EZL, 3EZM, 3EZN, 3EZO, 3EZP, 3EZQ, 3EZR, 3EZS, 3EZT, 3EZU, 3EZV, 3EZW, 3EZX, 3EZY, 3EZZ, 3F00, 3F01, 3F02, 3F03, 3F04, 3F05, 3F06, 3F07, 3F08, 3F0A, 3F0B, 3F0C, 3F0D, 3F0E, 3F0F, 3F0G, 3F0H, 3F0I, 3F0L, 3F0M, 3F0N, 3F0O, 3F0P, 3F0Q, 3F0R, 3F0S, 3F0T, 3F0U, 3F0V, 3F0W, 3F0X, 3F0Y, 3F0Z, 3F10, 3F11, 3F12, 3F13, 3F14, 3F15, 3F16, 3F17, 3F18, 3F19, 3F1A, 3F1B, 3F1C, 3F1I, 3F1J, 3F1K, 3F1L, 3F1N, 3F1O, 3F1P, 3F1Q, 3F1R, 3F1S, 3F1T, 3F1V, 3F1W, 3F1X, 3F1Y, 3F1Z, 3F21, 3F22, 3F23, 3F27, 3F28, 3F29, 3F2A, 3F2B, 3F2C, 3F2D, 3F2E, 3F2F, 3F2G, 3F2H, 3F2I, 3F2K, 3F2L, 3F2M, 3F2N, 3F2O, 3F2P, 3F2R, 3F2U, 3F2V, 3F2Z, 3F31, 3F32, 3F33, 3F34, 3F35, 3F36, 3F37, 3F38, 3F39, 3F3A, 3F3B, 3F3C, 3F3D, 3F3E, 3F3F, 3F3G, 3F3H, 3F3K, 3F3M, 3F3P, 3F3Q, 3F3R, 3F3S, 3F3T, 3F3U, 3F3V, 3F3W, 3F3X, 3F3Y, 3F3Z, 3F40, 3F41, 3F42, 3F43, 3F44, 3F45, 3F46, 3F47, 3F48, 3F49, 3F4A, 3F4B, 3F4C, 3F4D, 3F4F, 3F4I, 3F4J, 3F4K, 3F4L, 3F4M, 3F4N, 3F4R, 3F4S, 3F4T, 3F4V, 3F4W, 3F4X, 3F4Y, 3F50, 3F51, 3F52, 3F53, 3F56, 3F57, 3F58, 3F59, 3F5A, 3F5B, 3F5C, 3F5D, 3F5E, 3F5F, 3F5G, 3F5H, 3F5J, 3F5K, 3F5L, 3F5M, 3F5N, 3F5O, 3F5P, 3F5Q, 3F5R, 3F5S, 3F5T, 3F5U, 3F5V, 3F5W, 3F5X, 3F61, 3F62, 3F63, 3F64, 3F65, 3F66, 3F67, 3F68, 3F69, 3F6A, 3F6B, 3F6C, 3F6D, 3F6E, 3F6F, 3F6G, 3F6H, 3F6I, 3F6J, 3F6K, 3F6L, 3F6M, 3F6N, 3F6O, 3F6P, 3F6Q, 3F6R, 3F6S, 3F6T, 3F6U, 3F6V, 3F6W, 3F6X, 3F6Y, 3F6Z, 3F70, 3F71, 3F72, 3F74, 3F75, 3F78, 3F79, 3F7A, 3F7B, 3F7C, 3F7D, 3F7E, 3F7F, 3F7G, 3F7H, 3F7I, 3F7J, 3F7K, 3F7L, 3F7M, 3F7N, 3F7O, 3F7P, 3F7Q, 3F7R, 3F7S, 3F7T, 3F7U, 3F7V, 3F7W, 3F7X, 3F7Y, 3F7Z, 3F80, 3F81, 3F82, 3F83, 3F84, 3F85, 3F88, 3F89, 3F8A, 3F8B, 3F8C, 3F8D, 3F8E, 3F8F, 3F8G, 3F8H, 3F8I, 3F8J, 3F8K, 3F8L, 3F8M, 3F8N, 3F8O, 3F8P, 3F8R, 3F8S, 3F8T, 3F8U, 3F8V, 3F8W, 3F8X, 3F8Y, 3F8Z, 3F90, 3F91, 3F92, 3F95, 3F96, 3F97, 3F98, 3F99, 3F9A, 3F9B, 3F9C, 3F9D, 3F9E, 3F9F, 3F9G, 3F9H, 3F9I, 3F9K, 3F9L, 3F9M, 3F9N, 3F9O, 3F9P, 3F9Q, 3F9R, 3F9S, 3F9T, 3F9U, 3F9V, 3F9W, 3F9X, 3F9Y, 3F9Z, 3FA0, 3FA2, 3FA3, 3FA4, 3FA5, 3FA6, 3FA7, 3FA8, 3FA9, 3FAA, 3FAC, 3FAD, 3FAH, 3FAI, 3FAJ, 3FAK, 3FAL, 3FAN, 3FAO, 3FAP, 3FAQ, 3FAS, 3FAT, 3FAU, 3FAV, 3FAW, 3FAX, 3FAY, 3FAZ, 3FB0, 3FB1, 3FB2, 3FB3, 3FB4, 3FB5, 3FB6, 3FB7, 3FB8, 3FB9, 3FBA, 3FBB, 3FBC, 3FBD, 3FBE, 3FBF, 3FBG, 3FBI, 3FBK, 3FBL, 3FBN, 3FBO, 3FBP, 3FBQ, 3FBR, 3FBS, 3FBT, 3FBU, 3FBV, 3FBW, 3FBX, 3FBY, 3FBZ, 3FC0, 3FC1, 3FC2, 3FC3, 3FC4, 3FC5, 3FC6, 3FC7, 3FC8, 3FC9, 3FCA, 3FCB, 3FCC, 3FCD, 3FCE, 3FCF, 3FCG, 3FCH, 3FCI, 3FCJ, 3FCK, 3FCL, 3FCM, 3FCN, 3FCO, 3FCP, 3FCQ, 3FCR, 3FCS, 3FCT, 3FCU, 3FCV, 3FCW, 3FCX, 3FCY, 3FCZ, 3FD0, 3FD2, 3FD3, 3FD4, 3FD5, 3FD6, 3FD7, 3FD8, 3FD9, 3FDB, 3FDC, 3FDD, 3FDE, 3FDF, 3FDG, 3FDH, 3FDI, 3FDJ, 3FDK, 3FDL, 3FDM, 3FDN, 3FDO, 3FDQ, 3FDR, 3FDS, 3FDT, 3FDU, 3FDW, 3FDX, 3FDY, 3FDZ, 3FE0, 3FE1, 3FE2, 3FE3, 3FE4, 3FE5, 3FE6, 3FE7, 3FE8, 3FE9, 3FEA, 3FEC, 3FED, 3FEE, 3FEF, 3FEG, 3FEH, 3FEI, 3FEJ, 3FEK, 3FEL, 3FEM, 3FEN, 3FEO, 3FEP, 3FEQ, 3FER, 3FES, 3FET, 3FEU, 3FEV, 3FEW, 3FEX, 3FEY, 3FEZ, 3FF0, 3FF1, 3FF2, 3FF3, 3FF4, 3FF5, 3FF6, 3FF7, 3FF8, 3FF9, 3FFA, 3FFB, 3FFC, 3FFD, 3FFE, 3FFG, 3FFH, 3FFI, 3FFK, 3FFL, 3FFM, 3FFN, 3FFO, 3FFP, 3FFQ, 3FFR, 3FFS, 3FFT, 3FFU, 3FFV, 3FFW, 3FFX, 3FFY, 3FFZ, 3FG1, 3FG2, 3FG3, 3FG4, 3FG5, 3FG6, 3FG7, 3FG8, 3FG9, 3FGA, 3FGB, 3FGC, 3FGD, 3FGE, 3FGG, 3FGH, 3FGM, 3FGN, 3FGO, 3FGP, 3FGQ, 3FGR, 3FGS, 3FGT, 3FGU, 3FGV, 3FGW, 3FGX, 3FGY, 3FGZ, 3FH0, 3FH1, 3FH2, 3FH3, 3FH4, 3FH5, 3FH6, 3FH7, 3FH8, 3FH9, 3FHA, 3FHB, 3FHC, 3FHD, 3FHE, 3FHF, 3FHG, 3FHH, 3FHI, 3FHJ, 3FHK, 3FHL, 3FHM, 3FHN, 3FHO, 3FHQ, 3FHR, 3FHU, 3FHV, 3FHW, 3FHX, 3FHY, 3FHZ, 3FI0, 3FI2, 3FI3, 3FI4, 3FI5, 3FI6, 3FI7, 3FI8, 3FI9, 3FIA, 3FIB, 3FID, 3FIE, 3FIF, 3FIG, 3FII, 3FIJ, 3FIL, 3FIM, 3FIP, 3FIQ, 3FIR, 3FIS, 3FIT, 3FIU, 3FIV, 3FIW, 3FIX, 3FJ1, 3FJ2, 3FJ4, 3FJ5, 3FJ6, 3FJ7, 3FJ8, 3FJ9, 3FJA, 3FJB, 3FJC, 3FJD, 3FJE, 3FJF, 3FJG, 3FJH, 3FJI, 3FJJ, 3FJK, 3FJL, 3FJM, 3FJN, 3FJO, 3FJP, 3FJQ, 3FJS, 3FJT, 3FJU, 3FJV, 3FJW, 3FJX, 3FJY, 3FJZ, 3FK0, 3FK1, 3FK2, 3FK3, 3FK4, 3FK5, 3FK6, 3FK7, 3FK8, 3FK9, 3FKA, 3FKB, 3FKC, 3FKD, 3FKE, 3FKF, 3FKG, 3FKH, 3FKI, 3FKJ, 3FKK, 3FKL, 3FKM, 3FKN, 3FKO, 3FKQ, 3FKR, 3FKS, 3FKT, 3FKU, 3FKV, 3FKW, 3FKY, 3FKZ, 3FL0, 3FL1, 3FL2, 3FL3, 3FL4, 3FL5, 3FL6, 3FL7, 3FL8, 3FL9, 3FLA, 3FLB, 3FLC, 3FLD, 3FLE, 3FLF, 3FLG, 3FLH, 3FLI, 3FLJ, 3FLK, 3FLL, 3FLM, 3FLN, 3FLO, 3FLP, 3FLQ, 3FLR, 3FLS, 3FLT, 3FLU, 3FLV, 3FLW, 3FLY, 3FLZ, 3FM0, 3FM1, 3FM2, 3FM3, 3FM4, 3FM5, 3FM6, 3FM7, 3FM8, 3FM9, 3FMA, 3FMB, 3FMC, 3FMD, 3FME, 3FMF, 3FMG, 3FMH, 3FMI, 3FMJ, 3FMK, 3FML, 3FMM, 3FMN, 3FMO, 3FMP, 3FMQ, 3FMR, 3FMS, 3FMT, 3FMU, 3FMV, 3FMW, 3FMX, 3FMY, 3FMZ, 3FN0, 3FN1, 3FN2, 3FN3, 3FN4, 3FN5, 3FN6, 3FN7, 3FN8, 3FN9, 3FNA, 3FNB, 3FNC, 3FND, 3FNE, 3FNF, 3FNG, 3FNH, 3FNI, 3FNJ, 3FNK, 3FNL, 3FNM, 3FNN, 3FNQ, 3FNR, 3FNS, 3FNT, 3FNU, 3FNV, 3FO0, 3FO1, 3FO2, 3FO3, 3FO5, 3FO7, 3FO8, 3FO9, 3FOA, 3FOB, 3FOC, 3FOD, 3FOE, 3FOF, 3FOG, 3FOJ, 3FOK, 3FOL, 3FOM, 3FON, 3FOO, 3FOP, 3FOQ, 3FOR, 3FOT, 3FOU, 3FOV, 3FOW, 3FP0, 3FP2, 3FP3, 3FP4, 3FP5, 3FP6, 3FP7, 3FP8, 3FP9, 3FPA, 3FPB, 3FPC, 3FPD, 3FPE, 3FPF, 3FPG, 3FPH, 3FPI, 3FPJ, 3FPK, 3FPL, 3FPM, 3FPN, 3FPO, 3FPP, 3FPQ, 3FPR, 3FPS, 3FPT, 3FPU, 3FPV, 3FPW, 3FPX, 3FPY, 3FPZ, 3FQ0, 3FQ1, 3FQ2, 3FQ3, 3FQ4, 3FQ5, 3FQ6, 3FQ7, 3FQ8, 3FQ9, 3FQA, 3FQB, 3FQC, 3FQD, 3FQE, 3FQF, 3FQG, 3FQH, 3FQI, 3FQJ, 3FQK, 3FQL, 3FQM, 3FQN, 3FQO, 3FQQ, 3FQR, 3FQS, 3FQT, 3FQU, 3FQV, 3FQW, 3FQX, 3FQY, 3FQZ, 3FR0, 3FR1, 3FR2, 3FR3, 3FR4, 3FR5, 3FR6, 3FR7, 3FR8, 3FR9, 3FRA, 3FRB, 3FRC, 3FRD, 3FRE, 3FRF, 3FRG, 3FRH, 3FRI, 3FRJ, 3FRK, 3FRL, 3FRM, 3FRN, 3FRO, 3FRP, 3FRQ, 3FRR, 3FRS, 3FRT, 3FRU, 3FRV, 3FRW, 3FRX, 3FRY, 3FRZ, 3FS1, 3FS2, 3FS3, 3FS4, 3FS5, 3FS6, 3FS7, 3FS8, 3FS9, 3FSA, 3FSB, 3FSC, 3FSD, 3FSE, 3FSF, 3FSG, 3FSH, 3FSI, 3FSJ, 3FSK, 3FSL, 3FSM, 3FSN, 3FSO, 3FSP, 3FSQ, 3FSR, 3FSS, 3FST, 3FSU, 3FSV, 3FSW, 3FSX, 3FSY, 3FSZ, 3FT0, 3FT1, 3FT2, 3FT3, 3FT4, 3FT5, 3FT6, 3FT7, 3FT8, 3FT9, 3FTB, 3FTC, 3FTD, 3FTG, 3FTH, 3FTJ, 3FTK, 3FTL, 3FTN, 3FTO, 3FTP, 3FTQ, 3FTR, 3FTS, 3FTT, 3FTU, 3FTV, 3FTW, 3FTX, 3FTY, 3FTZ, 3FU0, 3FU1, 3FU3, 3FU5, 3FU6, 3FU7, 3FU8, 3FU9, 3FUA, 3FUB, 3FUC, 3FUD, 3FUE, 3FUF, 3FUG, 3FUH, 3FUI, 3FUJ, 3FUK, 3FUL, 3FUM, 3FUN, 3FUO, 3FUP, 3FUQ, 3FUR, 3FUT, 3FUU, 3FUV, 3FUW, 3FUX, 3FUY, 3FUZ, 3FV1, 3FV2, 3FV3, 3FV4, 3FV5, 3FV6, 3FV7, 3FV8, 3FV9, 3FVA, 3FVB, 3FVC, 3FVD, 3FVE, 3FVF, 3FVG, 3FVH, 3FVI, 3FVJ, 3FVK, 3FVL, 3FVM, 3FVN, 3FVO, 3FVP, 3FVQ, 3FVR, 3FVS, 3FVT, 3FVU, 3FVV, 3FVW, 3FVX, 3FVY, 3FVZ, 3FW0, 3FW1, 3FW2, 3FW3, 3FW4, 3FW5, 3FW6, 3FW7, 3FW8, 3FW9, 3FWA, 3FWB, 3FWC, 3FWE, 3FWF, 3FWG, 3FWH, 3FWI, 3FWJ, 3FWK, 3FWL, 3FWN, 3FWP, 3FWQ, 3FWR, 3FWS, 3FWT, 3FWU, 3FWV, 3FWW, 3FWX, 3FWY, 3FWZ, 3FX0, 3FX2, 3FX3, 3FX4, 3FX5, 3FX6, 3FX7, 3FXA, 3FXB, 3FXD, 3FXE, 3FXG, 3FXH, 3FXI, 3FXJ, 3FXK, 3FXL, 3FXM, 3FXO, 3FXP, 3FXQ, 3FXR, 3FXS, 3FXT, 3FXU, 3FXV, 3FXW, 3FXX, 3FXY, 3FXZ, 3FY0, 3FY1, 3FY2, 3FY3, 3FY4, 3FY5, 3FY6, 3FY7, 3FY8, 3FY9, 3FYA, 3FYB, 3FYC, 3FYD, 3FYE, 3FYF, 3FYG, 3FYH, 3FYI, 3FYJ, 3FYK, 3FYL, 3FYM, 3FYN, 3FYO, 3FYP, 3FYQ, 3FYR, 3FYS, 3FYT, 3FYU, 3FYV, 3FYW, 3FYX, 3FYY, 3FYZ, 3FZ0, 3FZ1, 3FZ2, 3FZ3, 3FZ4, 3FZ5, 3FZ6, 3FZ7, 3FZ8, 3FZ9, 3FZA, 3FZB, 3FZC, 3FZD, 3FZE, 3FZF, 3FZG, 3FZH, 3FZI, 3FZJ, 3FZK, 3FZL, 3FZM, 3FZN, 3FZO, 3FZP, 3FZQ, 3FZR, 3FZS, 3FZT, 3FZU, 3FZV, 3FZW, 3FZX, 3FZY, 3FZZ, 3G00, 3G01, 3G02, 3G03, 3G04, 3G05, 3G06, 3G07, 3G08, 3G0A, 3G0B, 3G0C, 3G0D, 3G0E, 3G0F, 3G0G, 3G0I, 3G0J, 3G0K, 3G0L, 3G0M, 3G0O, 3G0Q, 3G0R, 3G0S, 3G0T, 3G0U, 3G0V, 3G0W, 3G0X, 3G0Y, 3G0Z, 3G10, 3G11, 3G12, 3G13, 3G14, 3G15, 3G16, 3G17, 3G18, 3G19, 3G1A, 3G1B, 3G1C, 3G1D, 3G1E, 3G1F, 3G1G, 3G1H, 3G1I, 3G1J, 3G1K, 3G1L, 3G1M, 3G1N, 3G1O, 3G1P, 3G1Q, 3G1R, 3G1S, 3G1T, 3G1U, 3G1V, 3G1W, 3G1X, 3G1Y, 3G1Z, 3G20, 3G21, 3G22, 3G23, 3G24, 3G25, 3G26, 3G27, 3G28, 3G29, 3G2A, 3G2B, 3G2C, 3G2D, 3G2E, 3G2F, 3G2G, 3G2H, 3G2I, 3G2J, 3G2K, 3G2L, 3G2M, 3G2N, 3G2O, 3G2P, 3G2Q, 3G2R, 3G2S, 3G2T, 3G2U, 3G2V, 3G2W, 3G2X, 3G2Y, 3G2Z, 3G30, 3G31, 3G32, 3G33, 3G34, 3G35, 3G36, 3G38, 3G39, 3G3A, 3G3B, 3G3C, 3G3D, 3G3E, 3G3F, 3G3G, 3G3H, 3G3I, 3G3J, 3G3K, 3G3L, 3G3M, 3G3N, 3G3O, 3G3P, 3G3Q, 3G3R, 3G3S, 3G3T, 3G3U, 3G3V, 3G3W, 3G3X, 3G3Z, 3G40, 3G42, 3G43, 3G45, 3G46, 3G48, 3G49, 3G4A, 3G4C, 3G4D, 3G4E, 3G4F, 3G4G, 3G4H, 3G4I, 3G4K, 3G4L, 3G4N, 3G4O, 3G4P, 3G4Q, 3G4R, 3G4T, 3G4U, 3G4V, 3G4W, 3G4X, 3G4Y, 3G4Z, 3G50, 3G51, 3G52, 3G53, 3G56, 3G58, 3G59, 3G5A, 3G5B, 3G5C, 3G5D, 3G5E, 3G5F, 3G5G, 3G5H, 3G5I, 3G5J, 3G5K, 3G5L, 3G5M, 3G5N, 3G5O, 3G5P, 3G5Q, 3G5R, 3G5S, 3G5T, 3G5U, 3G5V, 3G5W, 3G5X, 3G5Y, 3G5Z, 3G60, 3G61, 3G64, 3G65, 3G66, 3G67, 3G68, 3G69, 3G6A, 3G6B, 3G6D, 3G6G, 3G6H, 3G6I, 3G6J, 3G6K, 3G6L, 3G6M, 3G6N, 3G6O, 3G6P, 3G6Q, 3G6R, 3G6S, 3G6T, 3G6U, 3G6V, 3G6W, 3G6X, 3G6Y, 3G6Z, 3G70, 3G72, 3G73, 3G74, 3G75, 3G76, 3G77, 3G79, 3G7A, 3G7B, 3G7C, 3G7D, 3G7E, 3G7F, 3G7G, 3G7I, 3G7J, 3G7K, 3G7L, 3G7M, 3G7N, 3G7P, 3G7Q, 3G7R, 3G7S, 3G7T, 3G7U, 3G7V, 3G7W, 3G7X, 3G7Y, 3G7Z, 3G80, 3G81, 3G82, 3G83, 3G84, 3G85, 3G86, 3G87, 3G88, 3G89, 3G8A, 3G8B, 3G8C, 3G8D, 3G8E, 3G8F, 3G8G, 3G8H, 3G8I, 3G8K, 3G8L, 3G8M, 3G8O, 3G8Q, 3G8R, 3G8U, 3G8V, 3G8W, 3G8X, 3G8Y, 3G8Z, 3G90, 3G91, 3G93, 3G97, 3G98, 3G99, 3G9A, 3G9B, 3G9D, 3G9E, 3G9G, 3G9H, 3G9I, 3G9J, 3G9K, 3G9L, 3G9M, 3G9N, 3G9O, 3G9P, 3G9Q, 3G9R, 3G9T, 3G9U, 3G9V, 3G9W, 3G9X, 3G9Z, 3GA0, 3GA1, 3GA2, 3GA3, 3GA4, 3GA5, 3GA6, 3GA7, 3GA8, 3GA9, 3GAA, 3GAB, 3GAC, 3GAD, 3GAE, 3GAF, 3GAG, 3GAH, 3GAI, 3GAJ, 3GAK, 3GAL, 3GAM, 3GAN, 3GAQ, 3GAR, 3GAS, 3GAX, 3GAY, 3GAZ, 3GB0, 3GB2, 3GB3, 3GB4, 3GB5, 3GB6, 3GB7, 3GB8, 3GB9, 3GBA, 3GBB, 3GBD, 3GBE, 3GBF, 3GBG, 3GBH, 3GBI, 3GBJ, 3GBK, 3GBL, 3GBM, 3GBN, 3GBO, 3GBP, 3GBR, 3GBS, 3GBT, 3GBU, 3GBV, 3GBW, 3GBX, 3GBY, 3GBZ, 3GC0, 3GC1, 3GC2, 3GC3, 3GC4, 3GC5, 3GC6, 3GC7, 3GC8, 3GC9, 3GCB, 3GCD, 3GCE, 3GCF, 3GCG, 3GCH, 3GCI, 3GCJ, 3GCK, 3GCL, 3GCM, 3GCN, 3GCO, 3GCP, 3GCQ, 3GCS, 3GCT, 3GCU, 3GCV, 3GCW, 3GCX, 3GCY, 3GCZ, 3GD0, 3GD1, 3GD2, 3GD3, 3GD4, 3GD5, 3GD6, 3GD7, 3GD8, 3GD9, 3GDA, 3GDB, 3GDC, 3GDE, 3GDF, 3GDG, 3GDH, 3GDI, 3GDJ, 3GDK, 3GDL, 3GDM, 3GDN, 3GDO, 3GDP, 3GDQ, 3GDR, 3GDS, 3GDT, 3GDU, 3GDV, 3GDW, 3GDX, 3GDZ, 3GE1, 3GE2, 3GE3, 3GE4, 3GE5, 3GE6, 3GE7, 3GE8, 3GE9, 3GEA, 3GEB, 3GEC, 3GED, 3GEE, 3GEF, 3GEG, 3GEH, 3GEI, 3GEK, 3GEL, 3GEM, 3GEN, 3GEO, 3GEP, 3GEQ, 3GET, 3GEU, 3GEW, 3GEX, 3GEY, 3GEZ, 3GF0, 3GF2, 3GF3, 3GF4, 3GF5, 3GF6, 3GF7, 3GF8, 3GF9, 3GFA, 3GFB, 3GFC, 3GFD, 3GFE, 3GFF, 3GFG, 3GFH, 3GFI, 3GFJ, 3GFK, 3GFL, 3GFM, 3GFO, 3GFP, 3GFQ, 3GFR, 3GFS, 3GFT, 3GFU, 3GFV, 3GFW, 3GFX, 3GFY, 3GFZ, 3GG0, 3GG1, 3GG2, 3GG3, 3GG4, 3GG5, 3GG6, 3GG7, 3GG8, 3GG9, 3GGA, 3GGC, 3GGD, 3GGE, 3GGF, 3GGG, 3GGH, 3GGI, 3GGJ, 3GGK, 3GGL, 3GGM, 3GGN, 3GGO, 3GGP, 3GGQ, 3GGR, 3GGS, 3GGU, 3GGV, 3GGW, 3GGX, 3GGY, 3GGZ, 3GH0, 3GH1, 3GH2, 3GH3, 3GH4, 3GH5, 3GH6, 3GH7, 3GH8, 3GH9, 3GHA, 3GHB, 3GHC, 3GHD, 3GHE, 3GHF, 3GHG, 3GHH, 3GHJ, 3GHM, 3GHN, 3GHP, 3GHQ, 3GHR, 3GHS, 3GHT, 3GHU, 3GHV, 3GHW, 3GHY, 3GHZ, 3GI0, 3GI1, 3GI2, 3GI3, 3GI4, 3GI5, 3GI6, 3GI7, 3GI8, 3GI9, 3GIA, 3GIC, 3GID, 3GIE, 3GIF, 3GIG, 3GII, 3GIJ, 3GIK, 3GIL, 3GIM, 3GIN, 3GIO, 3GIP, 3GIQ, 3GIR, 3GIS, 3GIT, 3GIU, 3GIV, 3GIW, 3GIX, 3GIY, 3GIZ, 3GJ0, 3GJ1, 3GJ2, 3GJ3, 3GJ4, 3GJ5, 3GJ6, 3GJ7, 3GJ8, 3GJ9, 3GJA, 3GJB, 3GJC, 3GJD, 3GJE, 3GJF, 3GJH, 3GJJ, 3GJK, 3GJL, 3GJN, 3GJO, 3GJP, 3GJQ, 3GJR, 3GJS, 3GJT, 3GJU, 3GJW, 3GJX, 3GJY, 3GJZ, 3GK0, 3GK1, 3GK2, 3GK3, 3GK4, 3GK5, 3GK6, 3GK7, 3GK8, 3GK9, 3GKA, 3GKB, 3GKE, 3GKF, 3GKH, 3GKI, 3GKJ, 3GKK, 3GKL, 3GKM, 3GKN, 3GKO, 3GKQ, 3GKR, 3GKT, 3GKU, 3GKV, 3GKW, 3GKX, 3GKY, 3GKZ, 3GL0, 3GL1, 3GL2, 3GL3, 3GL4, 3GL5, 3GL6, 3GL9, 3GLA, 3GLB, 3GLC, 3GLD, 3GLE, 3GLF, 3GLG, 3GLH, 3GLI, 3GLJ, 3GLK, 3GLL, 3GLM, 3GLN, 3GLQ, 3GLR, 3GLS, 3GLT, 3GLU, 3GLV, 3GLW, 3GLX, 3GLY, 3GLZ, 3GM0, 3GM1, 3GM2, 3GM3, 3GM5, 3GM6, 3GM8, 3GMA, 3GMB, 3GMC, 3GMD, 3GME, 3GMF, 3GMG, 3GMH, 3GMI, 3GMJ, 3GML, 3GMM, 3GMN, 3GMO, 3GMP, 3GMQ, 3GMR, 3GMS, 3GMT, 3GMU, 3GMV, 3GMW, 3GMX, 3GMY, 3GMZ, 3GN0, 3GN1, 3GN2, 3GN3, 3GN4, 3GN5, 3GN6, 3GN7, 3GN8, 3GN9, 3GNA, 3GNB, 3GNC, 3GND, 3GNE, 3GNF, 3GNG, 3GNI, 3GNJ, 3GNK, 3GNL, 3GNM, 3GNN, 3GNO, 3GNP, 3GNQ, 3GNR, 3GNS, 3GNT, 3GNU, 3GNV, 3GNW, 3GNX, 3GNY, 3GNZ, 3GO1, 3GO2, 3GO4, 3GO5, 3GO6, 3GO7, 3GO8, 3GO9, 3GOA, 3GOB, 3GOC, 3GOD, 3GOE, 3GOF, 3GOH, 3GOI, 3GOJ, 3GOK, 3GOL, 3GOM, 3GON, 3GOO, 3GOP, 3GOQ, 3GOR, 3GOS, 3GOU, 3GOV, 3GOX, 3GOY, 3GOZ, 3GP0, 3GP1, 3GP2, 3GP3, 3GP4, 3GP5, 3GP6, 3GP7, 3GP8, 3GP9, 3GPA, 3GPB, 3GPC, 3GPD, 3GPE, 3GPG, 3GPH, 3GPI, 3GPJ, 3GPK, 3GPL, 3GPM, 3GPN, 3GPO, 3GPP, 3GPR, 3GPS, 3GPT, 3GPU, 3GPV, 3GPW, 3GPX, 3GPY, 3GQ0, 3GQ1, 3GQ2, 3GQ3, 3GQ4, 3GQ5, 3GQ7, 3GQ8, 3GQ9, 3GQA, 3GQB, 3GQC, 3GQE, 3GQF, 3GQG, 3GQH, 3GQI, 3GQJ, 3GQK, 3GQL, 3GQM, 3GQO, 3GQP, 3GQQ, 3GQR, 3GQS, 3GQT, 3GQU, 3GQV, 3GQX, 3GQY, 3GQZ, 3GR0, 3GR1, 3GR2, 3GR3, 3GR4, 3GR5, 3GR6, 3GR7, 3GR8, 3GR9, 3GRA, 3GRB, 3GRC, 3GRD, 3GRE, 3GRF, 3GRG, 3GRH, 3GRI, 3GRJ, 3GRK, 3GRL, 3GRN, 3GRO, 3GRP, 3GRR, 3GRS, 3GRT, 3GRU, 3GRV, 3GRW, 3GRY, 3GRZ, 3GS0, 3GS2, 3GS3, 3GS4, 3GS6, 3GS7, 3GS9, 3GSB, 3GSD, 3GSE, 3GSG, 3GSH, 3GSI, 3GSK, 3GSL, 3GSM, 3GSN, 3GSO, 3GSP, 3GSQ, 3GSR, 3GSS, 3GST, 3GSU, 3GSV, 3GSW, 3GSX, 3GSY, 3GSZ, 3GT0, 3GT2, 3GT3, 3GT4, 3GT5, 3GT6, 3GT7, 3GT8, 3GT9, 3GTA, 3GTC, 3GTD, 3GTE, 3GTF, 3GTH, 3GTI, 3GTN, 3GTS, 3GTT, 3GTU, 3GTV, 3GTX, 3GTY, 3GTZ, 3GU0, 3GU1, 3GU2, 3GU3, 3GU4, 3GU5, 3GU6, 3GU7, 3GU8, 3GU9, 3GUA, 3GUB, 3GUC, 3GUD, 3GUE, 3GUF, 3GUG, 3GUH, 3GUI, 3GUJ, 3GUK, 3GUL, 3GUM, 3GUN, 3GUO, 3GUP, 3GUQ, 3GUR, 3GUS, 3GUT, 3GUU, 3GUV, 3GUW, 3GUX, 3GUY, 3GUZ, 3GV0, 3GV1, 3GV2, 3GV3, 3GV4, 3GV5, 3GV6, 3GV7, 3GV8, 3GV9, 3GVA, 3GVB, 3GVC, 3GVD, 3GVE, 3GVF, 3GVG, 3GVH, 3GVI, 3GVJ, 3GVK, 3GVL, 3GVM, 3GVO, 3GVP, 3GVQ, 3GVR, 3GVT, 3GVU, 3GVV, 3GVW, 3GVX, 3GVY, 3GVZ, 3GW0, 3GW1, 3GW2, 3GW3, 3GW4, 3GW5, 3GW6, 3GW7, 3GW8, 3GW9, 3GWA, 3GWB, 3GWC, 3GWD, 3GWE, 3GWF, 3GWG, 3GWH, 3GWI, 3GWJ, 3GWK, 3GWL, 3GWM, 3GWN, 3GWO, 3GWP, 3GWQ, 3GWR, 3GWS, 3GWT, 3GWU, 3GWV, 3GWW, 3GWX, 3GWY, 3GWZ, 3GX0, 3GX1, 3GX4, 3GX8, 3GX9, 3GXA, 3GXB, 3GXD, 3GXE, 3GXF, 3GXG, 3GXH, 3GXI, 3GXK, 3GXL, 3GXM, 3GXN, 3GXO, 3GXP, 3GXQ, 3GXR, 3GXT, 3GXU, 3GXV, 3GXW, 3GXX, 3GXY, 3GXZ, 3GY0, 3GY1, 3GY2, 3GY3, 3GY4, 3GY5, 3GY6, 3GY7, 3GY8, 3GY9, 3GYA, 3GYB, 3GYC, 3GYD, 3GYE, 3GYF, 3GYG, 3GYH, 3GYI, 3GYJ, 3GYK, 3GYL, 3GYM, 3GYN, 3GYO, 3GYP, 3GYQ, 3GYR, 3GYS, 3GYT, 3GYU, 3GYV, 3GYW, 3GYX, 3GYY, 3GYZ, 3GZ0, 3GZ1, 3GZ2, 3GZ3, 3GZ4, 3GZ5, 3GZ6, 3GZ7, 3GZ8, 3GZ9, 3GZA, 3GZB, 3GZC, 3GZD, 3GZE, 3GZF, 3GZG, 3GZH, 3GZI, 3GZJ, 3GZK, 3GZL, 3GZM, 3GZN, 3GZO, 3GZP, 3GZQ, 3GZR, 3GZS, 3GZX, 3GZY, 3H00, 3H01, 3H02, 3H03, 3H04, 3H05, 3H06, 3H07, 3H08, 3H09, 3H0A, 3H0B, 3H0C, 3H0D, 3H0E, 3H0F, 3H0G, 3H0H, 3H0I, 3H0J, 3H0K, 3H0L, 3H0M, 3H0N, 3H0O, 3H0P, 3H0Q, 3H0R, 3H0S, 3H0T, 3H0U, 3H0V, 3H0W, 3H0X, 3H0Y, 3H0Z, 3H10, 3H11, 3H12, 3H13, 3H14, 3H15, 3H16, 3H17, 3H18, 3H19, 3H1A, 3H1B, 3H1C, 3H1D, 3H1E, 3H1F, 3H1G, 3H1H, 3H1I, 3H1J, 3H1K, 3H1L, 3H1M, 3H1N, 3H1O, 3H1P, 3H1Q, 3H1R, 3H1S, 3H1T, 3H1U, 3H1V, 3H1W, 3H1X, 3H1Y, 3H1Z, 3H20, 3H21, 3H22, 3H23, 3H24, 3H25, 3H26, 3H2A, 3H2B, 3H2C, 3H2D, 3H2E, 3H2F, 3H2G, 3H2H, 3H2I, 3H2J, 3H2K, 3H2L, 3H2M, 3H2N, 3H2O, 3H2P, 3H2Q, 3H2S, 3H2T, 3H2U, 3H2V, 3H2W, 3H2X, 3H2Y, 3H2Z, 3H30, 3H31, 3H32, 3H33, 3H34, 3H35, 3H36, 3H37, 3H38, 3H39, 3H3A, 3H3B, 3H3C, 3H3D, 3H3E, 3H3F, 3H3G, 3H3H, 3H3I, 3H3J, 3H3K, 3H3L, 3H3M, 3H3N, 3H3O, 3H3P, 3H3Q, 3H3R, 3H3S, 3H3T, 3H3U, 3H3X, 3H3Z, 3H40, 3H41, 3H42, 3H43, 3H44, 3H45, 3H46, 3H47, 3H49, 3H4B, 3H4C, 3H4D, 3H4E, 3H4F, 3H4G, 3H4H, 3H4I, 3H4J, 3H4K, 3H4L, 3H4M, 3H4O, 3H4P, 3H4Q, 3H4R, 3H4S, 3H4T, 3H4V, 3H4W, 3H4X, 3H4Y, 3H4Z, 3H50, 3H51, 3H52, 3H53, 3H54, 3H55, 3H56, 3H57, 3H58, 3H59, 3H5A, 3H5B, 3H5C, 3H5E, 3H5F, 3H5G, 3H5H, 3H5I, 3H5J, 3H5K, 3H5L, 3H5N, 3H5O, 3H5Q, 3H5R, 3H5S, 3H5T, 3H5U, 3H5V, 3H5W, 3H5Z, 3H60, 3H61, 3H62, 3H63, 3H64, 3H65, 3H66, 3H67, 3H68, 3H69, 3H6A, 3H6C, 3H6D, 3H6E, 3H6F, 3H6G, 3H6H, 3H6I, 3H6J, 3H6K, 3H6M, 3H6N, 3H6O, 3H6P, 3H6Q, 3H6R, 3H6S, 3H6T, 3H6U, 3H6V, 3H6W, 3H6X, 3H6Z, 3H70, 3H71, 3H72, 3H73, 3H74, 3H75, 3H76, 3H77, 3H78, 3H79, 3H7A, 3H7B, 3H7C, 3H7D, 3H7F, 3H7G, 3H7H, 3H7I, 3H7J, 3H7K, 3H7L, 3H7M, 3H7N, 3H7O, 3H7P, 3H7Q, 3H7R, 3H7S, 3H7T, 3H7U, 3H7V, 3H7W, 3H7X, 3H7Y, 3H7Z, 3H80, 3H81, 3H82, 3H83, 3H84, 3H85, 3H86, 3H87, 3H89, 3H8A, 3H8B, 3H8C, 3H8D, 3H8E, 3H8F, 3H8G, 3H8H, 3H8I, 3H8J, 3H8K, 3H8L, 3H8M, 3H8N, 3H8O, 3H8Q, 3H8R, 3H8S, 3H8T, 3H8U, 3H8V, 3H8W, 3H8X, 3H8Y, 3H8Z, 3H90, 3H91, 3H92, 3H93, 3H94, 3H95, 3H96, 3H97, 3H98, 3H99, 3H9A, 3H9B, 3H9C, 3H9D, 3H9E, 3H9F, 3H9G, 3H9H, 3H9J, 3H9K, 3H9M, 3H9N, 3H9O, 3H9P, 3H9Q, 3H9R, 3H9S, 3H9U, 3H9V, 3H9W, 3H9X, 3H9Y, 3H9Z, 3HA0, 3HA1, 3HA2, 3HA3, 3HA4, 3HA5, 3HA6, 3HA7, 3HA8, 3HA9, 3HAB, 3HAC, 3HAD, 3HAE, 3HAF, 3HAH, 3HAI, 3HAJ, 3HAK, 3HAL, 3HAM, 3HAN, 3HAO, 3HAP, 3HAQ, 3HAR, 3HAS, 3HAT, 3HAU, 3HAV, 3HAW, 3HAZ, 3HB0, 3HB1, 3HB2, 3HB3, 3HB4, 3HB5, 3HB6, 3HB7, 3HB8, 3HB9, 3HBA, 3HBB, 3HBC, 3HBD, 3HBE, 3HBF, 3HBG, 3HBH, 3HBJ, 3HBK, 3HBL, 3HBM, 3HBN, 3HBO, 3HBP, 3HBQ, 3HBR, 3HBT, 3HBU, 3HBV, 3HBW, 3HBX, 3HBZ, 3HC0, 3HC1, 3HC2, 3HC3, 3HC4, 3HC5, 3HC6, 3HC7, 3HC8, 3HC9, 3HCA, 3HCB, 3HCC, 3HCD, 3HCE, 3HCF, 3HCG, 3HCH, 3HCI, 3HCJ, 3HCL, 3HCM, 3HCN, 3HCO, 3HCP, 3HCQ, 3HCR, 3HCS, 3HCT, 3HCU, 3HCV, 3HCW, 3HCX, 3HCY, 3HCZ, 3HD0, 3HD1, 3HD2, 3HD3, 3HD4, 3HD5, 3HD6, 3HD7, 3HD8, 3HDA, 3HDB, 3HDC, 3HDD, 3HDE, 3HDF, 3HDG, 3HDH, 3HDI, 3HDJ, 3HDK, 3HDL, 3HDM, 3HDN, 3HDO, 3HDP, 3HDQ, 3HDS, 3HDT, 3HDU, 3HDV, 3HDX, 3HDY, 3HDZ, 3HE0, 3HE1, 3HE2, 3HE3, 3HE4, 3HE5, 3HE6, 3HE7, 3HE8, 3HEA, 3HEB, 3HEC, 3HEE, 3HEF, 3HEG, 3HEI, 3HEJ, 3HEK, 3HEM, 3HEN, 3HEO, 3HEP, 3HEQ, 3HER, 3HES, 3HF1, 3HF2, 3HF3, 3HF4, 3HF5, 3HF6, 3HF7, 3HF8, 3HF9, 3HFA, 3HFB, 3HFC, 3HFD, 3HFE, 3HFF, 3HFG, 3HFI, 3HFJ, 3HFK, 3HFM, 3HFN, 3HFO, 3HFP, 3HFQ, 3HFR, 3HFS, 3HFT, 3HFU, 3HFV, 3HFW, 3HFX, 3HFY, 3HFZ, 3HG0, 3HG1, 3HG2, 3HG3, 3HG4, 3HG5, 3HG6, 3HG7, 3HG9, 3HGB, 3HGF, 3HGG, 3HGI, 3HGJ, 3HGK, 3HGL, 3HGM, 3HGN, 3HGO, 3HGP, 3HGQ, 3HGR, 3HGS, 3HGT, 3HGU, 3HGV, 3HGW, 3HGX, 3HGY, 3HGZ, 3HH0, 3HH1, 3HH2, 3HH3, 3HH4, 3HH5, 3HH6, 3HH7, 3HH8, 3HHA, 3HHB, 3HHC, 3HHD, 3HHE, 3HHF, 3HHG, 3HHH, 3HHI, 3HHJ, 3HHK, 3HHL, 3HHM, 3HHP, 3HHQ, 3HHR, 3HHS, 3HHT, 3HHU, 3HHV, 3HHW, 3HHX, 3HHY, 3HI0, 3HI1, 3HI2, 3HI4, 3HI5, 3HI6, 3HI7, 3HI8, 3HI9, 3HIA, 3HIB, 3HIC, 3HID, 3HIE, 3HIF, 3HIG, 3HIH, 3HII, 3HIJ, 3HIK, 3HIL, 3HIM, 3HIN, 3HIO, 3HIP, 3HIQ, 3HIS, 3HIT, 3HIU, 3HIV, 3HIW, 3HIX, 3HIY, 3HIZ, 3HJ0, 3HJ1, 3HJ2, 3HJ3, 3HJ4, 3HJ5, 3HJ6, 3HJ7, 3HJ8, 3HJ9, 3HJA, 3HJB, 3HJC, 3HJD, 3HJE, 3HJG, 3HJH, 3HJI, 3HJJ, 3HJK, 3HJL, 3HJM, 3HJN, 3HJO, 3HJP, 3HJQ, 3HJR, 3HJS, 3HJT, 3HJU, 3HJV, 3HJX, 3HJZ, 3HK0, 3HK1, 3HK3, 3HK4, 3HK5, 3HK6, 3HK7, 3HK8, 3HK9, 3HKA, 3HKB, 3HKC, 3HKD, 3HKE, 3HKF, 3HKI, 3HKJ, 3HKK, 3HKL, 3HKM, 3HKN, 3HKO, 3HKP, 3HKQ, 3HKR, 3HKS, 3HKT, 3HKU, 3HKV, 3HKW, 3HKX, 3HKY, 3HKZ, 3HL0, 3HL1, 3HL3, 3HL4, 3HL5, 3HL6, 3HL7, 3HL8, 3HL9, 3HLA, 3HLB, 3HLC, 3HLD, 3HLE, 3HLF, 3HLG, 3HLH, 3HLI, 3HLJ, 3HLK, 3HLL, 3HLM, 3HLN, 3HLO, 3HLP, 3HLR, 3HLS, 3HLT, 3HLU, 3HLV, 3HLW, 3HLX, 3HLY, 3HLZ, 3HM0, 3HM1, 3HM2, 3HM3, 3HM4, 3HM5, 3HM6, 3HM7, 3HM8, 3HMB, 3HMC, 3HME, 3HMF, 3HMG, 3HMH, 3HMI, 3HMJ, 3HMK, 3HML, 3HMM, 3HMN, 3HMO, 3HMP, 3HMQ, 3HMR, 3HMS, 3HMT, 3HMU, 3HMV, 3HMW, 3HMX, 3HMY, 3HMZ, 3HN0, 3HN1, 3HN2, 3HN3, 3HN4, 3HN5, 3HN6, 3HN7, 3HN8, 3HNA, 3HNB, 3HNC, 3HND, 3HNE, 3HNF, 3HNG, 3HNH, 3HNI, 3HNJ, 3HNK, 3HNL, 3HNM, 3HNO, 3HNP, 3HNQ, 3HNR, 3HNS, 3HNT, 3HNU, 3HNV, 3HNW, 3HNX, 3HNY, 3HNZ, 3HO0, 3HO2, 3HO3, 3HO4, 3HO5, 3HO6, 3HO7, 3HO8, 3HO9, 3HOA, 3HOB, 3HOC, 3HOD, 3HOE, 3HOF, 3HOG, 3HOH, 3HOI, 3HOJ, 3HOK, 3HOL, 3HOM, 3HON, 3HOP, 3HOR, 3HOS, 3HOT, 3HP0, 3HP1, 3HP2, 3HP3, 3HP4, 3HP5, 3HP6, 3HP7, 3HP8, 3HP9, 3HPA, 3HPB, 3HPC, 3HPD, 3HPE, 3HPF, 3HPG, 3HPH, 3HPI, 3HPJ, 3HPK, 3HPL, 3HPM, 3HPN, 3HPO, 3HPQ, 3HPR, 3HPS, 3HPT, 3HPV, 3HPW, 3HPX, 3HPY, 3HPZ, 3HQ0, 3HQ1, 3HQ2, 3HQ4, 3HQ5, 3HQ6, 3HQ7, 3HQ8, 3HQ9, 3HQA, 3HQB, 3HQC, 3HQD, 3HQE, 3HQF, 3HQG, 3HQH, 3HQI, 3HQJ, 3HQL, 3HQM, 3HQN, 3HQO, 3HQP, 3HQQ, 3HQR, 3HQT, 3HQU, 3HQW, 3HQX, 3HQY, 3HQZ, 3HR0, 3HR1, 3HR4, 3HR5, 3HR6, 3HR7, 3HR8, 3HR9, 3HRA, 3HRB, 3HRC, 3HRD, 3HRE, 3HRF, 3HRG, 3HRH, 3HRI, 3HRK, 3HRL, 3HRM, 3HRN, 3HRO, 3HRP, 3HRQ, 3HRR, 3HRS, 3HRT, 3HRU, 3HRV, 3HRW, 3HRX, 3HRY, 3HRZ, 3HS0, 3HS1, 3HS2, 3HS3, 3HS4, 3HS5, 3HS6, 3HS7, 3HS8, 3HS9, 3HSA, 3HSC, 3HSD, 3HSE, 3HSG, 3HSH, 3HSI, 3HSJ, 3HSK, 3HSL, 3HSM, 3HSN, 3HSO, 3HSP, 3HSQ, 3HSR, 3HSS, 3HST, 3HSU, 3HSV, 3HSW, 3HSY, 3HSZ, 3HT0, 3HT1, 3HT2, 3HT3, 3HT4, 3HT5, 3HT6, 3HT7, 3HT8, 3HT9, 3HTA, 3HTB, 3HTC, 3HTD, 3HTE, 3HTF, 3HTG, 3HTH, 3HTI, 3HTJ, 3HTK, 3HTL, 3HTM, 3HTN, 3HTO, 3HTP, 3HTQ, 3HTR, 3HTS, 3HTT, 3HTU, 3HTV, 3HTW, 3HTY, 3HTZ, 3HU1, 3HU2, 3HU3, 3HU5, 3HU6, 3HU7, 3HU8, 3HU9, 3HUA, 3HUB, 3HUC, 3HUD, 3HUE, 3HUF, 3HUG, 3HUH, 3HUI, 3HUJ, 3HUK, 3HUL, 3HUM, 3HUN, 3HUO, 3HUP, 3HUQ, 3HUR, 3HUS, 3HUT, 3HUU, 3HUV, 3HV0, 3HV1, 3HV2, 3HV3, 3HV4, 3HV5, 3HV6, 3HV7, 3HV8, 3HV9, 3HVA, 3HVC, 3HVD, 3HVE, 3HVF, 3HVG, 3HVH, 3HVI, 3HVJ, 3HVK, 3HVL, 3HVM, 3HVN, 3HVO, 3HVP, 3HVQ, 3HVS, 3HVT, 3HVU, 3HVV, 3HVW, 3HVX, 3HVY, 3HVZ, 3HW1, 3HW2, 3HW3, 3HW4, 3HW5, 3HW6, 3HW7, 3HW8, 3HW9, 3HWB, 3HWC, 3HWD, 3HWE, 3HWF, 3HWG, 3HWI, 3HWJ, 3HWK, 3HWL, 3HWN, 3HWO, 3HWP, 3HWR, 3HWS, 3HWT, 3HWU, 3HWW, 3HWX, 3HX0, 3HX1, 3HX2, 3HX3, 3HX4, 3HX5, 3HX6, 3HX7, 3HX8, 3HX9, 3HXA, 3HXB, 3HXC, 3HXD, 3HXE, 3HXF, 3HXG, 3HXI, 3HXJ, 3HXK, 3HXL, 3HXN, 3HXO, 3HXP, 3HXQ, 3HXR, 3HXS, 3HXT, 3HXU, 3HXV, 3HXW, 3HXX, 3HXY, 3HXZ, 3HY0, 3HY1, 3HY2, 3HY3, 3HY4, 3HY5, 3HY6, 3HY7, 3HY8, 3HY9, 3HYB, 3HYC, 3HYD, 3HYE, 3HYF, 3HYG, 3HYH, 3HYI, 3HYJ, 3HYK, 3HYL, 3HYM, 3HYN, 3HYO, 3HYP, 3HYQ, 3HYR, 3HYS, 3HYT, 3HYU, 3HYV, 3HYW, 3HYX, 3HYY, 3HYZ, 3HZ1, 3HZ2, 3HZ3, 3HZ4, 3HZ5, 3HZ6, 3HZ7, 3HZ8, 3HZA, 3HZB, 3HZC, 3HZD, 3HZE, 3HZF, 3HZG, 3HZH, 3HZI, 3HZJ, 3HZK, 3HZL, 3HZM, 3HZN, 3HZO, 3HZP, 3HZQ, 3HZR, 3HZS, 3HZT, 3HZU, 3HZV, 3HZW, 3HZX, 3HZY, 3HZZ, 3I00, 3I01, 3I02, 3I03, 3I04, 3I05, 3I06, 3I07, 3I08, 3I09, 3I0A, 3I0C, 3I0D, 3I0E, 3I0F, 3I0G, 3I0H, 3I0I, 3I0J, 3I0K, 3I0L, 3I0M, 3I0N, 3I0O, 3I0P, 3I0Q, 3I0R, 3I0S, 3I0T, 3I0U, 3I0V, 3I0W, 3I0X, 3I0Y, 3I0Z, 3I10, 3I11, 3I12, 3I13, 3I14, 3I15, 3I16, 3I17, 3I18, 3I19, 3I1A, 3I1C, 3I1E, 3I1F, 3I1G, 3I1H, 3I1I, 3I1J, 3I1K, 3I1L, 3I1U, 3I1Y, 3I23, 3I24, 3I25, 3I26, 3I27, 3I28, 3I29, 3I2A, 3I2B, 3I2C, 3I2D, 3I2E, 3I2F, 3I2G, 3I2H, 3I2I, 3I2J, 3I2K, 3I2L, 3I2M, 3I2N, 3I2O, 3I2T, 3I2V, 3I2W, 3I2X, 3I2Y, 3I2Z, 3I30, 3I31, 3I32, 3I33, 3I34, 3I35, 3I36, 3I37, 3I38, 3I39, 3I3A, 3I3B, 3I3C, 3I3D, 3I3E, 3I3F, 3I3G, 3I3H, 3I3I, 3I3J, 3I3L, 3I3M, 3I3N, 3I3O, 3I3Q, 3I3R, 3I3S, 3I3T, 3I3U, 3I3V, 3I3W, 3I3X, 3I3Y, 3I3Z, 3I40, 3I41, 3I42, 3I43, 3I44, 3I45, 3I46, 3I47, 3I48, 3I49, 3I4A, 3I4B, 3I4C, 3I4D, 3I4E, 3I4F, 3I4G, 3I4H, 3I4I, 3I4J, 3I4K, 3I4L, 3I4O, 3I4P, 3I4Q, 3I4R, 3I4S, 3I4T, 3I4U, 3I4V, 3I4W, 3I4X, 3I4Y, 3I4Z, 3I50, 3I51, 3I52, 3I53, 3I54, 3I57, 3I58, 3I59, 3I5A, 3I5B, 3I5C, 3I5D, 3I5E, 3I5F, 3I5G, 3I5H, 3I5I, 3I5J, 3I5K, 3I5M, 3I5N, 3I5O, 3I5P, 3I5Q, 3I5R, 3I5S, 3I5T, 3I5U, 3I5V, 3I5W, 3I5Z, 3I60, 3I63, 3I64, 3I65, 3I67, 3I68, 3I69, 3I6A, 3I6B, 3I6C, 3I6D, 3I6E, 3I6F, 3I6G, 3I6H, 3I6I, 3I6J, 3I6K, 3I6L, 3I6M, 3I6N, 3I6O, 3I6P, 3I6Q, 3I6R, 3I6S, 3I6T, 3I6U, 3I6V, 3I6W, 3I6X, 3I6Y, 3I6Z, 3I70, 3I71, 3I72, 3I73, 3I74, 3I75, 3I76, 3I77, 3I78, 3I79, 3I7A, 3I7B, 3I7C, 3I7D, 3I7E, 3I7F, 3I7G, 3I7H, 3I7I, 3I7J, 3I7K, 3I7L, 3I7M, 3I7N, 3I7O, 3I7P, 3I7Q, 3I7R, 3I7S, 3I7T, 3I7U, 3I7V, 3I7W, 3I7X, 3I7Y, 3I7Z, 3I80, 3I81, 3I82, 3I83, 3I84, 3I85, 3I86, 3I87, 3I89, 3I8A, 3I8B, 3I8C, 3I8D, 3I8E, 3I8N, 3I8O, 3I8P, 3I8R, 3I8S, 3I8T, 3I8U, 3I8V, 3I8W, 3I8X, 3I8Z, 3I90, 3I91, 3I92, 3I93, 3I94, 3I95, 3I96, 3I97, 3I98, 3I99, 3I9A, 3I9F, 3I9G, 3I9H, 3I9I, 3I9J, 3I9K, 3I9L, 3I9M, 3I9N, 3I9O, 3I9P, 3I9Q, 3I9S, 3I9T, 3I9U, 3I9V, 3I9W, 3I9X, 3I9Y, 3I9Z, 3IA0, 3IA1, 3IA2, 3IA3, 3IA4, 3IA5, 3IA6, 3IA7, 3IA8, 3IA9, 3IAA, 3IAC, 3IAD, 3IAE, 3IAF, 3IAG, 3IAH, 3IAI, 3IAJ, 3IAK, 3IAL, 3IAM, 3IAN, 3IAO, 3IAP, 3IAQ, 3IAR, 3IAS, 3IAU, 3IAV, 3IAW, 3IAX, 3IAY, 3IAZ, 3IB0, 3IB1, 3IB2, 3IB3, 3IB4, 3IB5, 3IB6, 3IB7, 3IB8, 3IB9, 3IBA, 3IBB, 3IBC, 3IBD, 3IBE, 3IBF, 3IBG, 3IBH, 3IBI, 3IBJ, 3IBL, 3IBM, 3IBN, 3IBO, 3IBP, 3IBQ, 3IBR, 3IBS, 3IBT, 3IBU, 3IBV, 3IBW, 3IBX, 3IBY, 3IBZ, 3IC0, 3IC1, 3IC2, 3IC3, 3IC4, 3IC5, 3IC6, 3IC7, 3IC8, 3IC9, 3ICA, 3ICB, 3ICC, 3ICD, 3ICF, 3ICH, 3ICI, 3ICJ, 3ICK, 3ICL, 3ICM, 3ICN, 3ICO, 3ICP, 3ICR, 3ICS, 3ICT, 3ICU, 3ICV, 3ICW, 3ICX, 3ICY, 3ICZ, 3ID0, 3ID1, 3ID2, 3ID3, 3ID4, 3ID6, 3ID7, 3ID8, 3ID9, 3IDA, 3IDB, 3IDC, 3IDD, 3IDF, 3IDG, 3IDH, 3IDI, 3IDJ, 3IDM, 3IDN, 3IDO, 3IDP, 3IDQ, 3IDS, 3IDU, 3IDV, 3IDW, 3IDX, 3IDY, 3IDZ, 3IE0, 3IE2, 3IE3, 3IE4, 3IE5, 3IE7, 3IE9, 3IEA, 3IEB, 3IEC, 3IED, 3IEE, 3IEF, 3IEG, 3IEH, 3IEI, 3IEJ, 3IEK, 3IEL, 3IEM, 3IEO, 3IEP, 3IEQ, 3IER, 3IES, 3IET, 3IEU, 3IEW, 3IEX, 3IEY, 3IEZ, 3IF0, 3IF1, 3IF2, 3IF4, 3IF5, 3IF6, 3IF7, 3IF8, 3IF9, 3IFA, 3IFC, 3IFD, 3IFE, 3IFF, 3IFG, 3IFH, 3IFI, 3IFJ, 3IFK, 3IFL, 3IFN, 3IFO, 3IFP, 3IFQ, 3IFR, 3IFS, 3IFT, 3IFU, 3IFV, 3IFW, 3IFX, 3IFZ, 3IG0, 3IG1, 3IG2, 3IG3, 3IG4, 3IG5, 3IG6, 3IG7, 3IG8, 3IG9, 3IGA, 3IGB, 3IGC, 3IGD, 3IGE, 3IGF, 3IGG, 3IGH, 3IGJ, 3IGK, 3IGL, 3IGM, 3IGN, 3IGO, 3IGP, 3IGQ, 3IGR, 3IGS, 3IGT, 3IGU, 3IGV, 3IGX, 3IGY, 3IGZ, 3IH0, 3IH2, 3IH3, 3IH4, 3IH5, 3IH6, 3IH7, 3IH8, 3IH9, 3IHA, 3IHB, 3IHC, 3IHD, 3IHE, 3IHF, 3IHG, 3IHI, 3IHJ, 3IHK, 3IHL, 3IHM, 3IHO, 3IHP, 3IHQ, 3IHR, 3IHS, 3IHT, 3IHU, 3IHV, 3IHW, 3IHX, 3IHY, 3IHZ, 3II0, 3II1, 3II2, 3II3, 3II4, 3II5, 3II6, 3II7, 3II9, 3IIA, 3IIB, 3IIC, 3IID, 3IIE, 3IIF, 3IIG, 3IIH, 3III, 3IIJ, 3IIK, 3IIL, 3IIM, 3IIO, 3IIP, 3IIQ, 3IIR, 3IIT, 3IIV, 3IIW, 3IIX, 3IIY, 3IIZ, 3IJ0, 3IJ1, 3IJ2, 3IJ3, 3IJ4, 3IJ5, 3IJ6, 3IJ7, 3IJ8, 3IJ9, 3IJC, 3IJD, 3IJE, 3IJF, 3IJG, 3IJH, 3IJI, 3IJJ, 3IJL, 3IJM, 3IJO, 3IJP, 3IJQ, 3IJR, 3IJS, 3IJT, 3IJU, 3IJV, 3IJW, 3IJX, 3IJY, 3IJZ, 3IK0, 3IK1, 3IK2, 3IK3, 3IK4, 3IK5, 3IK6, 3IK7, 3IK8, 3IK9, 3IKA, 3IKB, 3IKC, 3IKD, 3IKE, 3IKF, 3IKG, 3IKH, 3IKJ, 3IKK, 3IKL, 3IKM, 3IKN, 3IKO, 3IKP, 3IKQ, 3IKR, 3IKT, 3IKV, 3IKW, 3IL0, 3IL1, 3IL2, 3IL3, 3IL4, 3IL5, 3IL6, 3IL7, 3IL8, 3IL9, 3ILA, 3ILB, 3ILC, 3ILD, 3ILE, 3ILF, 3ILG, 3ILH, 3ILI, 3ILJ, 3ILK, 3ILL, 3ILM, 3ILN, 3ILO, 3ILP, 3ILQ, 3ILR, 3ILS, 3ILT, 3ILU, 3ILV, 3ILW, 3ILX, 3ILY, 3ILZ, 3IM0, 3IM1, 3IM2, 3IM3, 3IM4, 3IM5, 3IM6, 3IM7, 3IM8, 3IM9, 3IMA, 3IMB, 3IMC, 3IMD, 3IME, 3IMF, 3IMG, 3IMH, 3IMI, 3IMJ, 3IMK, 3IML, 3IMM, 3IMN, 3IMO, 3IMP, 3IMQ, 3IMR, 3IMS, 3IMT, 3IMU, 3IMV, 3IMW, 3IMX, 3IMY, 3IN0, 3IN1, 3IN2, 3IN3, 3IN4, 3IN5, 3IN6, 3IN7, 3IN8, 3IN9, 3INA, 3INB, 3INC, 3IND, 3INE, 3INF, 3ING, 3INH, 3INJ, 3INK, 3INL, 3INM, 3INN, 3INO, 3INP, 3INQ, 3INR, 3INS, 3INT, 3INU, 3INV, 3INW, 3INX, 3INY, 3INZ, 3IO0, 3IO1, 3IO2, 3IO3, 3IO4, 3IO5, 3IO6, 3IO7, 3IO8, 3IO9, 3IOB, 3IOC, 3IOD, 3IOE, 3IOF, 3IOG, 3IOH, 3IOI, 3IOJ, 3IOK, 3IOL, 3IOM, 3ION, 3IOP, 3IOQ, 3IOR, 3IOS, 3IOT, 3IOU, 3IOV, 3IOW, 3IOX, 3IOY, 3IOZ, 3IP0, 3IP1, 3IP2, 3IP3, 3IP4, 3IP5, 3IP6, 3IP7, 3IP8, 3IP9, 3IPA, 3IPB, 3IPC, 3IPD, 3IPE, 3IPF, 3IPH, 3IPI, 3IPJ, 3IPK, 3IPL, 3IPM, 3IPO, 3IPP, 3IPQ, 3IPR, 3IPS, 3IPT, 3IPU, 3IPV, 3IPW, 3IPX, 3IPY, 3IPZ, 3IQ0, 3IQ1, 3IQ2, 3IQ3, 3IQ5, 3IQ6, 3IQ7, 3IQA, 3IQB, 3IQC, 3IQD, 3IQE, 3IQF, 3IQG, 3IQH, 3IQI, 3IQJ, 3IQL, 3IQM, 3IQO, 3IQQ, 3IQS, 3IQT, 3IQU, 3IQV, 3IQW, 3IQX, 3IQY, 3IQZ, 3IR0, 3IR1, 3IR2, 3IR3, 3IR4, 3IR5, 3IR6, 3IR7, 3IR8, 3IR9, 3IRA, 3IRB, 3IRC, 3IRD, 3IRH, 3IRM, 3IRN, 3IRO, 3IRP, 3IRQ, 3IRR, 3IRS, 3IRT, 3IRU, 3IRV, 3IRX, 3IRZ, 3IS0, 3IS1, 3IS2, 3IS3, 3IS4, 3IS5, 3IS6, 3IS7, 3IS8, 3IS9, 3ISA, 3ISB, 3ISC, 3ISD, 3ISE, 3ISF, 3ISG, 3ISH, 3ISI, 3ISJ, 3ISL, 3ISM, 3ISN, 3ISO, 3ISP, 3ISQ, 3ISR, 3ISS, 3IST, 3ISU, 3ISV, 3ISW, 3ISX, 3ISY, 3ISZ, 3IT0, 3IT1, 3IT2, 3IT3, 3IT4, 3IT5, 3IT6, 3IT7, 3IT8, 3IT9, 3ITA, 3ITB, 3ITC, 3ITD, 3ITE, 3ITF, 3ITG, 3ITH, 3ITI, 3ITJ, 3ITK, 3ITL, 3ITM, 3ITN, 3ITO, 3ITP, 3ITQ, 3ITT, 3ITU, 3ITV, 3ITW, 3ITX, 3ITY, 3ITZ, 3IU0, 3IU1, 3IU2, 3IU3, 3IU4, 3IU5, 3IU6, 3IU7, 3IU8, 3IU9, 3IUB, 3IUC, 3IUD, 3IUE, 3IUF, 3IUG, 3IUH, 3IUI, 3IUJ, 3IUK, 3IUL, 3IUM, 3IUN, 3IUO, 3IUP, 3IUQ, 3IUR, 3IUS, 3IUT, 3IUU, 3IUV, 3IUW, 3IUX, 3IUY, 3IUZ, 3IV0, 3IV1, 3IV2, 3IV3, 3IV4, 3IV5, 3IV6, 3IV7, 3IV8, 3IV9, 3IVA, 3IVB, 3IVC, 3IVD, 3IVE, 3IVF, 3IVG, 3IVH, 3IVI, 3IVL, 3IVM, 3IVP, 3IVQ, 3IVR, 3IVS, 3IVT, 3IVU, 3IVV, 3IVX, 3IVY, 3IVZ, 3IW0, 3IW1, 3IW2, 3IW3, 3IW4, 3IW5, 3IW6, 3IW7, 3IW8, 3IWA, 3IWB, 3IWC, 3IWD, 3IWE, 3IWF, 3IWG, 3IWH, 3IWI, 3IWJ, 3IWK, 3IWL, 3IWM, 3IWO, 3IWP, 3IWQ, 3IWR, 3IWT, 3IWU, 3IWV, 3IWW, 3IWX, 3IWY, 3IWZ, 3IX0, 3IX1, 3IX3, 3IX4, 3IX6, 3IX7, 3IX8, 3IX9, 3IXA, 3IXB, 3IXC, 3IXD, 3IXE, 3IXF, 3IXG, 3IXH, 3IXJ, 3IXK, 3IXL, 3IXM, 3IXN, 3IXO, 3IXP, 3IXQ, 3IXR, 3IXS, 3IXT, 3JDW, 3JPN, 3JPO, 3JPP, 3JPQ, 3JPR, 3JPS, 3JPT, 3JPU, 3JPV, 3JPW, 3JPX, 3JPY, 3JPZ, 3JQ0, 3JQ1, 3JQ3, 3JQ5, 3JQ6, 3JQ7, 3JQ8, 3JQ9, 3JQA, 3JQB, 3JQC, 3JQD, 3JQE, 3JQF, 3JQG, 3JQH, 3JQJ, 3JQK, 3JQL, 3JQM, 3JQO, 3JQP, 3JQQ, 3JQR, 3JQU, 3JQW, 3JQX, 3JQY, 3JQZ, 3JR1, 3JR2, 3JR3, 3JR4, 3JR5, 3JR6, 3JR7, 3JR8, 3JR9, 3JRA, 3JRB, 3JRC, 3JRD, 3JRE, 3JRF, 3JRG, 3JRH, 3JRI, 3JRK, 3JRM, 3JRN, 3JRO, 3JRP, 3JRQ, 3JRR, 3JRS, 3JRT, 3JRU, 3JRV, 3JRW, 3JRX, 3JRY, 3JRZ, 3JS1, 3JS2, 3JS3, 3JS4, 3JS5, 3JS6, 3JS8, 3JS9, 3JSA, 3JSB, 3JSC, 3JSD, 3JSE, 3JSF, 3JSG, 3JSI, 3JSJ, 3JSK, 3JSL, 3JSM, 3JSN, 3JSO, 3JSP, 3JSQ, 3JSR, 3JSS, 3JST, 3JSU, 3JSV, 3JSW, 3JSX, 3JSY, 3JSZ, 3JT0, 3JT1, 3JT2, 3JT3, 3JT4, 3JT5, 3JT6, 3JT7, 3JT8, 3JT9, 3JTA, 3JTB, 3JTC, 3JTD, 3JTE, 3JTF, 3JTG, 3JTH, 3JTI, 3JTJ, 3JTK, 3JTL, 3JTM, 3JTN, 3JTO, 3JTP, 3JTQ, 3JTR, 3JTS, 3JTT, 3JTU, 3JTW, 3JTX, 3JTY, 3JTZ, 3JU0, 3JU1, 3JU2, 3JU3, 3JU4, 3JU5, 3JU6, 3JU7, 3JU8, 3JU9, 3JUA, 3JUB, 3JUC, 3JUD, 3JUE, 3JUG, 3JUH, 3JUI, 3JUJ, 3JUK, 3JUL, 3JUM, 3JUN, 3JUO, 3JUP, 3JUQ, 3JUR, 3JUS, 3JUT, 3JUU, 3JUV, 3JUW, 3JUX, 3JUY, 3JUZ, 3JV0, 3JV1, 3JV2, 3JV3, 3JV4, 3JV5, 3JV6, 3JV7, 3JV8, 3JV9, 3JVA, 3JVB, 3JVC, 3JVD, 3JVE, 3JVF, 3JVG, 3JVH, 3JVI, 3JVJ, 3JVK, 3JVL, 3JVM, 3JVN, 3JVO, 3JVR, 3JVS, 3JVT, 3JVU, 3JVV, 3JVW, 3JVX, 3JVY, 3JVZ, 3JW0, 3JW1, 3JW2, 3JW3, 3JW4, 3JW5, 3JW6, 3JW7, 3JW8, 3JW9, 3JWA, 3JWB, 3JWC, 3JWD, 3JWE, 3JWF, 3JWG, 3JWH, 3JWI, 3JWJ, 3JWK, 3JWM, 3JWN, 3JWO, 3JWP, 3JWQ, 3JWR, 3JWS, 3JWT, 3JWU, 3JWV, 3JWW, 3JWX, 3JWY, 3JWZ, 3JX0, 3JX1, 3JX2, 3JX3, 3JX4, 3JX5, 3JX6, 3JX7, 3JX8, 3JX9, 3JXA, 3JXB, 3JXC, 3JXD, 3JXE, 3JXF, 3JXG, 3JXH, 3JXI, 3JXJ, 3JXO, 3JXP, 3JXS, 3JXT, 3JXU, 3JXV, 3JXW, 3JXY, 3JXZ, 3JY0, 3JY1, 3JY6, 3JY9, 3JYA, 3JYB, 3JYC, 3JYF, 3JYG, 3JYH, 3JYI, 3JYJ, 3JYL, 3JYM, 3JYN, 3JYO, 3JYP, 3JYQ, 3JYR, 3JYS, 3JYT, 3JYU, 3JYY, 3JYZ, 3JZ0, 3JZ1, 3JZ2, 3JZ3, 3JZ4, 3JZ6, 3JZ7, 3JZ9, 3JZA, 3JZB, 3JZC, 3JZD, 3JZE, 3JZF, 3JZG, 3JZH, 3JZI, 3JZJ, 3JZK, 3JZL, 3JZM, 3JZN, 3JZO, 3JZP, 3JZQ, 3JZR, 3JZS, 3JZT, 3JZU, 3JZV, 3JZY, 3JZZ, 3K00, 3K01, 3K02, 3K03, 3K04, 3K05, 3K06, 3K07, 3K08, 3K09, 3K0A, 3K0B, 3K0C, 3K0D, 3K0E, 3K0F, 3K0G, 3K0H, 3K0I, 3K0K, 3K0L, 3K0M, 3K0N, 3K0O, 3K0P, 3K0Q, 3K0R, 3K0S, 3K0T, 3K0V, 3K0W, 3K0X, 3K0Y, 3K0Z, 3K10, 3K11, 3K12, 3K13, 3K14, 3K15, 3K16, 3K17, 3K19, 3K1A, 3K1B, 3K1D, 3K1E, 3K1F, 3K1G, 3K1H, 3K1I, 3K1J, 3K1K, 3K1L, 3K1M, 3K1N, 3K1O, 3K1P, 3K1R, 3K1S, 3K1T, 3K1U, 3K1W, 3K1X, 3K1Y, 3K1Z, 3K20, 3K21, 3K22, 3K23, 3K24, 3K25, 3K26, 3K27, 3K28, 3K29, 3K2A, 3K2B, 3K2C, 3K2D, 3K2E, 3K2F, 3K2G, 3K2H, 3K2I, 3K2J, 3K2K, 3K2L, 3K2M, 3K2N, 3K2O, 3K2P, 3K2Q, 3K2R, 3K2T, 3K2U, 3K2V, 3K2W, 3K2X, 3K2Y, 3K2Z, 3K30, 3K31, 3K32, 3K33, 3K34, 3K35, 3K36, 3K37, 3K38, 3K39, 3K3A, 3K3B, 3K3C, 3K3D, 3K3E, 3K3F, 3K3G, 3K3H, 3K3I, 3K3J, 3K3K, 3K3L, 3K3N, 3K3O, 3K3P, 3K3Q, 3K3R, 3K3S, 3K3T, 3K3U, 3K3V, 3K3W, 3K40, 3K41, 3K42, 3K43, 3K44, 3K45, 3K46, 3K47, 3K48, 3K4A, 3K4B, 3K4C, 3K4D, 3K4F, 3K4G, 3K4H, 3K4I, 3K4J, 3K4K, 3K4L, 3K4M, 3K4N, 3K4O, 3K4P, 3K4Q, 3K4S, 3K4T, 3K4U, 3K4V, 3K4W, 3K4X, 3K4Y, 3K4Z, 3K50, 3K51, 3K52, 3K53, 3K54, 3K55, 3K56, 3K57, 3K58, 3K59, 3K5B, 3K5C, 3K5D, 3K5E, 3K5F, 3K5G, 3K5H, 3K5I, 3K5J, 3K5K, 3K5L, 3K5M, 3K5N, 3K5O, 3K5P, 3K5R, 3K5S, 3K5T, 3K5U, 3K5V, 3K5W, 3K5X, 3K60, 3K63, 3K65, 3K66, 3K67, 3K69, 3K6A, 3K6B, 3K6C, 3K6D, 3K6E, 3K6F, 3K6G, 3K6H, 3K6I, 3K6J, 3K6K, 3K6L, 3K6M, 3K6N, 3K6O, 3K6P, 3K6Q, 3K6R, 3K6S, 3K6T, 3K6U, 3K6V, 3K6W, 3K6X, 3K6Y, 3K6Z, 3K70, 3K71, 3K72, 3K73, 3K74, 3K75, 3K77, 3K79, 3K7A, 3K7B, 3K7C, 3K7D, 3K7E, 3K7F, 3K7G, 3K7H, 3K7I, 3K7J, 3K7K, 3K7L, 3K7M, 3K7N, 3K7O, 3K7P, 3K7Q, 3K7R, 3K7S, 3K7T, 3K7U, 3K7V, 3K7W, 3K7X, 3K7Y, 3K7Z, 3K80, 3K81, 3K82, 3K83, 3K84, 3K85, 3K86, 3K87, 3K88, 3K89, 3K8A, 3K8B, 3K8C, 3K8D, 3K8E, 3K8G, 3K8H, 3K8I, 3K8J, 3K8K, 3K8L, 3K8M, 3K8N, 3K8O, 3K8P, 3K8Q, 3K8R, 3K8S, 3K8T, 3K8U, 3K8V, 3K8W, 3K8X, 3K8Y, 3K8Z, 3K90, 3K91, 3K92, 3K93, 3K94, 3K96, 3K97, 3K98, 3K99, 3K9A, 3K9B, 3K9C, 3K9D, 3K9E, 3K9F, 3K9G, 3K9H, 3K9I, 3K9J, 3K9K, 3K9L, 3K9M, 3K9N, 3K9O, 3K9P, 3K9Q, 3K9R, 3K9S, 3K9T, 3K9U, 3K9V, 3K9W, 3K9X, 3K9Y, 3K9Z, 3KA0, 3KA2, 3KA3, 3KA4, 3KA5, 3KA6, 3KA7, 3KA8, 3KA9, 3KAA, 3KAB, 3KAC, 3KAD, 3KAE, 3KAF, 3KAG, 3KAH, 3KAI, 3KAJ, 3KAK, 3KAL, 3KAM, 3KAN, 3KAO, 3KAP, 3KAQ, 3KAR, 3KAS, 3KAT, 3KAV, 3KAW, 3KAY, 3KAZ, 3KB0, 3KB1, 3KB2, 3KB3, 3KB4, 3KB5, 3KB6, 3KB7, 3KB8, 3KB9, 3KBA, 3KBB, 3KBC, 3KBE, 3KBF, 3KBG, 3KBH, 3KBJ, 3KBK, 3KBL, 3KBM, 3KBN, 3KBO, 3KBP, 3KBQ, 3KBR, 3KBS, 3KBT, 3KBU, 3KBV, 3KBW, 3KBX, 3KBY, 3KBZ, 3KC0, 3KC1, 3KC2, 3KC3, 3KC6, 3KCC, 3KCE, 3KCF, 3KCG, 3KCH, 3KCI, 3KCJ, 3KCK, 3KCL, 3KCM, 3KCN, 3KCO, 3KCP, 3KCQ, 3KCS, 3KCT, 3KCU, 3KCV, 3KCW, 3KCX, 3KCY, 3KCZ, 3KD0, 3KD1, 3KD2, 3KD3, 3KD4, 3KD5, 3KD6, 3KD7, 3KD8, 3KD9, 3KDA, 3KDB, 3KDC, 3KDD, 3KDE, 3KDF, 3KDG, 3KDH, 3KDI, 3KDJ, 3KDK, 3KDM, 3KDN, 3KDO, 3KDP, 3KDQ, 3KDR, 3KDS, 3KDT, 3KDU, 3KDW, 3KDY, 3KDZ, 3KE0, 3KE1, 3KE2, 3KE3, 3KE4, 3KE5, 3KE6, 3KE7, 3KE8, 3KE9, 3KEA, 3KEB, 3KEC, 3KED, 3KEE, 3KEF, 3KEG, 3KEH, 3KEI, 3KEJ, 3KEK, 3KEL, 3KEM, 3KEN, 3KEO, 3KEP, 3KEQ, 3KER, 3KES, 3KET, 3KEU, 3KEV, 3KEW, 3KEX, 3KEY, 3KEZ, 3KF0, 3KF2, 3KF3, 3KF4, 3KF5, 3KF6, 3KF7, 3KF8, 3KF9, 3KFA, 3KFB, 3KFC, 3KFD, 3KFE, 3KFF, 3KFG, 3KFH, 3KFI, 3KFJ, 3KFK, 3KFL, 3KFM, 3KFN, 3KFO, 3KFP, 3KFQ, 3KFR, 3KFS, 3KFT, 3KFV, 3KFW, 3KFX, 3KFY, 3KG0, 3KG1, 3KG2, 3KG4, 3KG5, 3KG6, 3KG7, 3KG8, 3KG9, 3KGA, 3KGB, 3KGC, 3KGD, 3KGF, 3KGG, 3KGK, 3KGL, 3KGP, 3KGQ, 3KGR, 3KGS, 3KGT, 3KGU, 3KGV, 3KGW, 3KGX, 3KGY, 3KGZ, 3KH0, 3KH1, 3KH2, 3KH3, 3KH4, 3KH5, 3KH7, 3KH8, 3KH9, 3KHB, 3KHC, 3KHD, 3KHE, 3KHF, 3KHG, 3KHH, 3KHI, 3KHJ, 3KHK, 3KHL, 3KHM, 3KHN, 3KHO, 3KHP, 3KHQ, 3KHR, 3KHS, 3KHT, 3KHU, 3KHV, 3KHW, 3KHX, 3KHY, 3KHZ, 3KI0, 3KI1, 3KI2, 3KI3, 3KI4, 3KI5, 3KI6, 3KI7, 3KI8, 3KI9, 3KIA, 3KID, 3KIF, 3KIG, 3KIH, 3KII, 3KIJ, 3KIK, 3KIN, 3KIO, 3KIP, 3KIV, 3KIZ, 3KJ0, 3KJ1, 3KJ2, 3KJ4, 3KJ6, 3KJ7, 3KJD, 3KJE, 3KJF, 3KJG, 3KJH, 3KJI, 3KJJ, 3KJK, 3KJL, 3KJM, 3KJN, 3KJO, 3KJP, 3KJQ, 3KJR, 3KJS, 3KJT, 3KJV, 3KJX, 3KJY, 3KJZ, 3KK0, 3KK1, 3KK2, 3KK3, 3KK4, 3KK6, 3KK7, 3KK8, 3KK9, 3KKA, 3KKB, 3KKC, 3KKD, 3KKE, 3KKF, 3KKG, 3KKI, 3KKJ, 3KKK, 3KKL, 3KKM, 3KKN, 3KKO, 3KKP, 3KKQ, 3KKR, 3KKS, 3KKT, 3KKU, 3KKV, 3KKW, 3KKY, 3KKZ, 3KL0, 3KL1, 3KL2, 3KL3, 3KL4, 3KL5, 3KL6, 3KL7, 3KL8, 3KL9, 3KLA, 3KLB, 3KLC, 3KLD, 3KLE, 3KLF, 3KLG, 3KLH, 3KLI, 3KLJ, 3KLK, 3KLL, 3KLM, 3KLN, 3KLO, 3KLP, 3KLQ, 3KLR, 3KLS, 3KLT, 3KLU, 3KLW, 3KLX, 3KLY, 3KLZ, 3KM0, 3KM1, 3KM2, 3KM3, 3KM4, 3KM5, 3KM6, 3KM8, 3KM9, 3KMA, 3KMB, 3KMC, 3KMD, 3KME, 3KMG, 3KMH, 3KMI, 3KMJ, 3KML, 3KMM, 3KMN, 3KMO, 3KMP, 3KMR, 3KMT, 3KMU, 3KMV, 3KMW, 3KMX, 3KMY, 3KMZ, 3KN0, 3KN1, 3KN2, 3KN3, 3KN4, 3KN5, 3KN6, 3KN7, 3KN8, 3KNB, 3KND, 3KNE, 3KNF, 3KNG, 3KNP, 3KNQ, 3KNR, 3KNS, 3KNT, 3KNU, 3KNV, 3KNW, 3KNX, 3KNY, 3KNZ, 3KO0, 3KO1, 3KO2, 3KO3, 3KO4, 3KO5, 3KO6, 3KO7, 3KO8, 3KO9, 3KOB, 3KOC, 3KOD, 3KOF, 3KOG, 3KOH, 3KOI, 3KOJ, 3KOK, 3KOL, 3KOM, 3KON, 3KOO, 3KOP, 3KOQ, 3KOR, 3KOS, 3KOT, 3KOU, 3KOV, 3KOW, 3KOX, 3KOY, 3KOZ, 3KP0, 3KP1, 3KP2, 3KP3, 3KP4, 3KP5, 3KP6, 3KP7, 3KP8, 3KP9, 3KPA, 3KPB, 3KPC, 3KPD, 3KPE, 3KPF, 3KPH, 3KPJ, 3KPK, 3KPL, 3KPM, 3KPN, 3KPO, 3KPP, 3KPQ, 3KPR, 3KPS, 3KPT, 3KPU, 3KPV, 3KPW, 3KPX, 3KPY, 3KPZ, 3KQ0, 3KQ4, 3KQ5, 3KQ6, 3KQ7, 3KQA, 3KQB, 3KQC, 3KQD, 3KQE, 3KQF, 3KQG, 3KQH, 3KQI, 3KQJ, 3KQK, 3KQL, 3KQM, 3KQN, 3KQO, 3KQP, 3KQQ, 3KQR, 3KQS, 3KQT, 3KQU, 3KQV, 3KQW, 3KQX, 3KQY, 3KQZ, 3KR0, 3KR1, 3KR2, 3KR3, 3KR4, 3KR5, 3KR6, 3KR7, 3KR8, 3KR9, 3KRA, 3KRB, 3KRC, 3KRD, 3KRE, 3KRF, 3KRG, 3KRJ, 3KRK, 3KRL, 3KRM, 3KRN, 3KRO, 3KRP, 3KRQ, 3KRR, 3KRS, 3KRT, 3KRU, 3KRV, 3KRW, 3KRX, 3KRY, 3KRZ, 3KS0, 3KS2, 3KS3, 3KS4, 3KS5, 3KS6, 3KS7, 3KS9, 3KSA, 3KSB, 3KSC, 3KSD, 3KSE, 3KSF, 3KSG, 3KSH, 3KSI, 3KSJ, 3KSK, 3KSL, 3KSM, 3KSN, 3KSO, 3KSP, 3KSQ, 3KSR, 3KSS, 3KST, 3KSU, 3KSV, 3KSW, 3KSX, 3KSY, 3KSZ, 3KT0, 3KT1, 3KT2, 3KT3, 3KT4, 3KT5, 3KT6, 3KT7, 3KT8, 3KT9, 3KTA, 3KTB, 3KTC, 3KTD, 3KTF, 3KTG, 3KTH, 3KTI, 3KTJ, 3KTK, 3KTL, 3KTM, 3KTN, 3KTO, 3KTP, 3KTQ, 3KTR, 3KTS, 3KTU, 3KTX, 3KTY, 3KTZ, 3KU0, 3KU1, 3KU2, 3KU3, 3KU4, 3KU5, 3KU6, 3KU7, 3KU9, 3KUC, 3KUD, 3KUE, 3KUF, 3KUG, 3KUH, 3KUI, 3KUJ, 3KUK, 3KUL, 3KUM, 3KUN, 3KUO, 3KUP, 3KUQ, 3KUR, 3KUS, 3KUT, 3KUU, 3KUV, 3KUW, 3KUX, 3KUY, 3KUZ, 3KV0, 3KV1, 3KV2, 3KV3, 3KV4, 3KV5, 3KV6, 3KV7, 3KV8, 3KV9, 3KVA, 3KVB, 3KVC, 3KVD, 3KVE, 3KVF, 3KVG, 3KVH, 3KVI, 3KVJ, 3KVK, 3KVL, 3KVM, 3KVN, 3KVO, 3KVP, 3KVQ, 3KVR, 3KVS, 3KVT, 3KVU, 3KVV, 3KVW, 3KVX, 3KVY, 3KVZ, 3KW0, 3KW1, 3KW2, 3KW3, 3KW4, 3KW5, 3KW6, 3KW7, 3KW8, 3KW9, 3KWA, 3KWB, 3KWC, 3KWD, 3KWE, 3KWF, 3KWG, 3KWI, 3KWJ, 3KWK, 3KWL, 3KWM, 3KWN, 3KWO, 3KWP, 3KWQ, 3KWR, 3KWS, 3KWT, 3KWU, 3KWV, 3KWW, 3KWX, 3KWY, 3KWZ, 3KX0, 3KX1, 3KX2, 3KX3, 3KX4, 3KX5, 3KX6, 3KX7, 3KX8, 3KX9, 3KXA, 3KXB, 3KXC, 3KXD, 3KXE, 3KXF, 3KXG, 3KXH, 3KXI, 3KXK, 3KXL, 3KXM, 3KXN, 3KXO, 3KXP, 3KXQ, 3KXR, 3KXS, 3KXT, 3KXU, 3KXV, 3KXW, 3KXX, 3KXY, 3KXZ, 3KY2, 3KY7, 3KY8, 3KY9, 3KYA, 3KYB, 3KYC, 3KYD, 3KYE, 3KYF, 3KYG, 3KYH, 3KYI, 3KYJ, 3KYK, 3KYM, 3KYN, 3KYO, 3KYP, 3KYQ, 3KYR, 3KYS, 3KYT, 3KYU, 3KYV, 3KYW, 3KYX, 3KYY, 3KYZ, 3KZ0, 3KZ1, 3KZ3, 3KZ5, 3KZ7, 3KZ8, 3KZ9, 3KZA, 3KZB, 3KZC, 3KZD, 3KZE, 3KZF, 3KZG, 3KZH, 3KZI, 3KZJ, 3KZK, 3KZL, 3KZM, 3KZN, 3KZO, 3KZP, 3KZQ, 3KZS, 3KZT, 3KZU, 3KZV, 3KZW, 3KZX, 3KZY, 3KZZ, 3L00, 3L01, 3L02, 3L03, 3L04, 3L05, 3L06, 3L07, 3L08, 3L09, 3L0A, 3L0B, 3L0C, 3L0D, 3L0E, 3L0F, 3L0G, 3L0H, 3L0I, 3L0J, 3L0K, 3L0L, 3L0M, 3L0N, 3L0O, 3L0P, 3L0Q, 3L0R, 3L0S, 3L0T, 3L0V, 3L0W, 3L0X, 3L0Y, 3L0Z, 3L10, 3L11, 3L12, 3L13, 3L14, 3L15, 3L16, 3L17, 3L18, 3L19, 3L1A, 3L1B, 3L1C, 3L1E, 3L1F, 3L1G, 3L1H, 3L1I, 3L1J, 3L1K, 3L1L, 3L1M, 3L1N, 3L1O, 3L1P, 3L1Q, 3L1R, 3L1S, 3L1T, 3L1U, 3L1V, 3L1W, 3L1X, 3L1Y, 3L1Z, 3L20, 3L21, 3L22, 3L23, 3L24, 3L27, 3L28, 3L29, 3L2A, 3L2B, 3L2C, 3L2D, 3L2E, 3L2F, 3L2G, 3L2H, 3L2I, 3L2J, 3L2K, 3L2L, 3L2M, 3L2N, 3L2O, 3L2P, 3L2Q, 3L2R, 3L2U, 3L2V, 3L2W, 3L2X, 3L2Y, 3L2Z, 3L30, 3L31, 3L32, 3L33, 3L34, 3L38, 3L39, 3L3A, 3L3B, 3L3D, 3L3F, 3L3G, 3L3H, 3L3I, 3L3J, 3L3K, 3L3L, 3L3M, 3L3N, 3L3O, 3L3P, 3L3Q, 3L3R, 3L3S, 3L3T, 3L3U, 3L3V, 3L3X, 3L3Z, 3L40, 3L41, 3L42, 3L43, 3L44, 3L45, 3L46, 3L47, 3L48, 3L49, 3L4A, 3L4B, 3L4C, 3L4D, 3L4E, 3L4F, 3L4G, 3L4H, 3L4I, 3L4J, 3L4K, 3L4L, 3L4M, 3L4N, 3L4O, 3L4P, 3L4Q, 3L4R, 3L4S, 3L4T, 3L4U, 3L4V, 3L4W, 3L4X, 3L4Y, 3L4Z, 3L50, 3L51, 3L54, 3L56, 3L57, 3L58, 3L59, 3L5A, 3L5B, 3L5C, 3L5D, 3L5E, 3L5F, 3L5H, 3L5I, 3L5J, 3L5K, 3L5L, 3L5M, 3L5N, 3L5O, 3L5P, 3L5Q, 3L5R, 3L5S, 3L5T, 3L5U, 3L5V, 3L5W, 3L5X, 3L5Z, 3L60, 3L61, 3L62, 3L63, 3L64, 3L65, 3L66, 3L67, 3L68, 3L6A, 3L6B, 3L6C, 3L6D, 3L6E, 3L6F, 3L6G, 3L6H, 3L6I, 3L6J, 3L6N, 3L6O, 3L6P, 3L6Q, 3L6R, 3L6T, 3L6U, 3L6V, 3L6W, 3L6X, 3L6Y, 3L70, 3L71, 3L72, 3L73, 3L74, 3L75, 3L76, 3L77, 3L78, 3L79, 3L7A, 3L7B, 3L7C, 3L7D, 3L7E, 3L7F, 3L7G, 3L7H, 3L7I, 3L7J, 3L7K, 3L7L, 3L7M, 3L7N, 3L7O, 3L7P, 3L7Q, 3L7R, 3L7T, 3L7U, 3L7V, 3L7W, 3L7X, 3L7Y, 3L7Z, 3L81, 3L82, 3L84, 3L85, 3L86, 3L87, 3L88, 3L89, 3L8A, 3L8B, 3L8C, 3L8D, 3L8E, 3L8F, 3L8G, 3L8H, 3L8I, 3L8J, 3L8K, 3L8M, 3L8N, 3L8P, 3L8Q, 3L8R, 3L8S, 3L8U, 3L8V, 3L8W, 3L8X, 3L8Y, 3L8Z, 3L91, 3L92, 3L93, 3L94, 3L95, 3L9A, 3L9B, 3L9C, 3L9D, 3L9E, 3L9F, 3L9G, 3L9H, 3L9I, 3L9J, 3L9K, 3L9L, 3L9M, 3L9N, 3L9O, 3L9P, 3L9Q, 3L9R, 3L9S, 3L9T, 3L9U, 3L9V, 3L9W, 3L9X, 3L9Y, 3L9Z, 3LA0, 3LA1, 3LA2, 3LA3, 3LA4, 3LA6, 3LA7, 3LA8, 3LA9, 3LAA, 3LAC, 3LAD, 3LAE, 3LAF, 3LAG, 3LAH, 3LAI, 3LAJ, 3LAK, 3LAL, 3LAM, 3LAN, 3LAO, 3LAP, 3LAQ, 3LAR, 3LAS, 3LAT, 3LAU, 3LAW, 3LAX, 3LAY, 3LAZ, 3LB0, 3LB1, 3LB2, 3LB3, 3LB4, 3LB5, 3LB6, 3LB8, 3LB9, 3LBA, 3LBB, 3LBC, 3LBD, 3LBE, 3LBF, 3LBG, 3LBH, 3LBI, 3LBJ, 3LBK, 3LBL, 3LBM, 3LBN, 3LBO, 3LBS, 3LBW, 3LBX, 3LBY, 3LBZ, 3LC0, 3LC1, 3LC2, 3LC3, 3LC4, 3LC5, 3LC6, 3LC7, 3LC8, 3LC9, 3LCA, 3LCB, 3LCC, 3LCD, 3LCE, 3LCF, 3LCG, 3LCH, 3LCI, 3LCJ, 3LCK, 3LCL, 3LCM, 3LCN, 3LCO, 3LCP, 3LCR, 3LCS, 3LCT, 3LCU, 3LCV, 3LCW, 3LCX, 3LCY, 3LCZ, 3LD0, 3LD1, 3LD2, 3LD3, 3LD4, 3LD5, 3LD6, 3LD7, 3LD8, 3LD9, 3LDA, 3LDB, 3LDC, 3LDD, 3LDE, 3LDF, 3LDG, 3LDH, 3LDI, 3LDJ, 3LDK, 3LDL, 3LDM, 3LDN, 3LDO, 3LDP, 3LDQ, 3LDR, 3LDS, 3LDT, 3LDU, 3LDV, 3LDW, 3LDX, 3LDY, 3LDZ, 3LE0, 3LE1, 3LE2, 3LE3, 3LE4, 3LE5, 3LE6, 3LE7, 3LE8, 3LE9, 3LEA, 3LEC, 3LED, 3LEE, 3LEF, 3LEG, 3LEH, 3LEI, 3LEK, 3LEL, 3LEM, 3LEN, 3LEO, 3LEP, 3LEQ, 3LER, 3LES, 3LET, 3LEV, 3LEW, 3LEX, 3LEY, 3LEZ, 3LF0, 3LF1, 3LF2, 3LF3, 3LF4, 3LF5, 3LF6, 3LF7, 3LF9, 3LFA, 3LFB, 3LFC, 3LFD, 3LFE, 3LFF, 3LFG, 3LFH, 3LFI, 3LFJ, 3LFK, 3LFL, 3LFM, 3LFN, 3LFO, 3LFP, 3LFQ, 3LFR, 3LFS, 3LFT, 3LFU, 3LFV, 3LFX, 3LFY, 3LFZ, 3LG0, 3LG1, 3LG2, 3LG3, 3LG4, 3LG5, 3LG6, 3LG7, 3LG8, 3LGA, 3LGB, 3LGC, 3LGD, 3LGE, 3LGF, 3LGG, 3LGH, 3LGI, 3LGJ, 3LGK, 3LGL, 3LGM, 3LGN, 3LGO, 3LGP, 3LGQ, 3LGR, 3LGS, 3LGT, 3LGU, 3LGV, 3LGW, 3LGX, 3LGY, 3LGZ, 3LH0, 3LH1, 3LH2, 3LH3, 3LH4, 3LH5, 3LH8, 3LH9, 3LHA, 3LHB, 3LHC, 3LHD, 3LHE, 3LHF, 3LHG, 3LHH, 3LHI, 3LHJ, 3LHK, 3LHL, 3LHM, 3LHN, 3LHO, 3LHP, 3LHQ, 3LHR, 3LHS, 3LHT, 3LHU, 3LHV, 3LHW, 3LHX, 3LHY, 3LHZ, 3LI0, 3LI1, 3LI2, 3LI3, 3LI4, 3LI5, 3LI6, 3LI7, 3LI8, 3LI9, 3LIA, 3LIB, 3LIC, 3LID, 3LIE, 3LIF, 3LIG, 3LIH, 3LII, 3LIJ, 3LIK, 3LIL, 3LIM, 3LIN, 3LIO, 3LIP, 3LIQ, 3LIR, 3LIS, 3LIT, 3LIU, 3LIV, 3LIW, 3LIX, 3LIY, 3LIZ, 3LJ0, 3LJ1, 3LJ2, 3LJ3, 3LJ4, 3LJ5, 3LJ6, 3LJ7, 3LJ8, 3LJ9, 3LJA, 3LJB, 3LJC, 3LJD, 3LJE, 3LJF, 3LJG, 3LJI, 3LJJ, 3LJK, 3LJL, 3LJM, 3LJN, 3LJO, 3LJP, 3LJQ, 3LJR, 3LJS, 3LJT, 3LJU, 3LJW, 3LJX, 3LJY, 3LJZ, 3LK0, 3LK1, 3LK2, 3LK3, 3LK4, 3LK5, 3LK6, 3LK7, 3LK8, 3LK9, 3LKA, 3LKB, 3LKD, 3LKE, 3LKF, 3LKH, 3LKI, 3LKJ, 3LKK, 3LKL, 3LKM, 3LKN, 3LKO, 3LKP, 3LKQ, 3LKR, 3LKS, 3LKT, 3LKU, 3LKV, 3LKW, 3LKX, 3LKY, 3LKZ, 3LL0, 3LL1, 3LL2, 3LL3, 3LL4, 3LL5, 3LL7, 3LL8, 3LL9, 3LLA, 3LLB, 3LLC, 3LLD, 3LLE, 3LLF, 3LLH, 3LLI, 3LLK, 3LLL, 3LLM, 3LLN, 3LLO, 3LLP, 3LLQ, 3LLR, 3LLS, 3LLT, 3LLU, 3LLV, 3LLW, 3LLX, 3LLY, 3LLZ, 3LM0, 3LM1, 3LM2, 3LM3, 3LM4, 3LM5, 3LM6, 3LM7, 3LM8, 3LM9, 3LMA, 3LMB, 3LMC, 3LMD, 3LME, 3LMF, 3LMG, 3LMH, 3LMI, 3LMJ, 3LMK, 3LML, 3LMM, 3LMN, 3LMO, 3LMP, 3LMS, 3LMT, 3LMU, 3LMV, 3LMW, 3LMX, 3LMY, 3LMZ, 3LN0, 3LN1, 3LN2, 3LN3, 3LN4, 3LN5, 3LN6, 3LN7, 3LN8, 3LN9, 3LNB, 3LNC, 3LND, 3LNE, 3LNF, 3LNG, 3LNH, 3LNI, 3LNJ, 3LNK, 3LNL, 3LNM, 3LNN, 3LNO, 3LNP, 3LNQ, 3LNR, 3LNS, 3LNT, 3LNU, 3LNV, 3LNW, 3LNX, 3LNY, 3LNZ, 3LO0, 3LO1, 3LO2, 3LO3, 3LO4, 3LO5, 3LO6, 3LO7, 3LO8, 3LO9, 3LOC, 3LOD, 3LOE, 3LOF, 3LOG, 3LOH, 3LOI, 3LOJ, 3LOK, 3LOM, 3LON, 3LOO, 3LOP, 3LOQ, 3LOR, 3LOT, 3LOU, 3LOV, 3LOW, 3LOX, 3LOY, 3LOZ, 3LP0, 3LP1, 3LP2, 3LP3, 3LP4, 3LP5, 3LP6, 3LP7, 3LP8, 3LP9, 3LPA, 3LPB, 3LPC, 3LPD, 3LPE, 3LPF, 3LPG, 3LPH, 3LPI, 3LPJ, 3LPK, 3LPL, 3LPM, 3LPN, 3LPO, 3LPP, 3LPQ, 3LPR, 3LPS, 3LPT, 3LPU, 3LPV, 3LPW, 3LPX, 3LPY, 3LPZ, 3LQ0, 3LQ1, 3LQ2, 3LQ3, 3LQ4, 3LQ5, 3LQ6, 3LQ7, 3LQ8, 3LQ9, 3LQA, 3LQB, 3LQC, 3LQD, 3LQE, 3LQF, 3LQG, 3LQH, 3LQI, 3LQJ, 3LQK, 3LQL, 3LQM, 3LQN, 3LQQ, 3LQR, 3LQS, 3LQU, 3LQV, 3LQW, 3LQY, 3LQZ, 3LR0, 3LR1, 3LR2, 3LR3, 3LR4, 3LR5, 3LR6, 3LR7, 3LR8, 3LR9, 3LRA, 3LRB, 3LRC, 3LRD, 3LRE, 3LRF, 3LRG, 3LRH, 3LRJ, 3LRK, 3LRL, 3LRM, 3LRP, 3LRQ, 3LRS, 3LRT, 3LRU, 3LRV, 3LRX, 3LRY, 3LS0, 3LS1, 3LS2, 3LS3, 3LS4, 3LS5, 3LS6, 3LS7, 3LS8, 3LS9, 3LSA, 3LSB, 3LSC, 3LSD, 3LSE, 3LSF, 3LSG, 3LSH, 3LSI, 3LSJ, 3LSK, 3LSL, 3LSM, 3LSN, 3LSO, 3LSP, 3LSQ, 3LSR, 3LSS, 3LST, 3LSU, 3LSV, 3LSW, 3LSX, 3LSY, 3LSZ, 3LT0, 3LT1, 3LT2, 3LT3, 3LT4, 3LT5, 3LT6, 3LT7, 3LT8, 3LT9, 3LTA, 3LTB, 3LTC, 3LTD, 3LTE, 3LTF, 3LTG, 3LTH, 3LTI, 3LTJ, 3LTL, 3LTM, 3LTN, 3LTO, 3LTP, 3LTQ, 3LTS, 3LTV, 3LTW, 3LTY, 3LU1, 3LU2, 3LU6, 3LU7, 3LU8, 3LU9, 3LUA, 3LUB, 3LUC, 3LUD, 3LUF, 3LUG, 3LUH, 3LUI, 3LUJ, 3LUK, 3LUL, 3LUM, 3LUN, 3LUO, 3LUP, 3LUQ, 3LUR, 3LUS, 3LUT, 3LUU, 3LUY, 3LUZ, 3LV0, 3LV1, 3LV2, 3LV3, 3LV4, 3LV5, 3LV6, 3LV8, 3LV9, 3LVA, 3LVB, 3LVC, 3LVD, 3LVE, 3LVF, 3LVG, 3LVH, 3LVJ, 3LVK, 3LVL, 3LVM, 3LVP, 3LVQ, 3LVR, 3LVS, 3LVT, 3LVU, 3LVV, 3LVW, 3LVX, 3LVY, 3LVZ, 3LW0, 3LW1, 3LW2, 3LW3, 3LW6, 3LW7, 3LW8, 3LW9, 3LWA, 3LWB, 3LWC, 3LWD, 3LWE, 3LWF, 3LWG, 3LWH, 3LWI, 3LWJ, 3LWK, 3LWL, 3LWM, 3LWN, 3LWS, 3LWT, 3LWU, 3LWW, 3LWX, 3LWZ, 3LX0, 3LX1, 3LX2, 3LX3, 3LX4, 3LX5, 3LX6, 3LX7, 3LX8, 3LX9, 3LXA, 3LXB, 3LXC, 3LXD, 3LXE, 3LXF, 3LXG, 3LXH, 3LXI, 3LXJ, 3LXK, 3LXL, 3LXM, 3LXN, 3LXO, 3LXP, 3LXQ, 3LXR, 3LXS, 3LXT, 3LXU, 3LXV, 3LXX, 3LXY, 3LXZ, 3LY0, 3LY1, 3LY2, 3LY3, 3LY4, 3LY5, 3LY6, 3LY7, 3LY8, 3LY9, 3LYA, 3LYB, 3LYC, 3LYD, 3LYE, 3LYF, 3LYG, 3LYH, 3LYI, 3LYK, 3LYL, 3LYM, 3LYN, 3LYO, 3LYP, 3LYQ, 3LYR, 3LYS, 3LYT, 3LYU, 3LYV, 3LYW, 3LYX, 3LYY, 3LYZ, 3LZ0, 3LZ1, 3LZ2, 3LZ3, 3LZ5, 3LZ6, 3LZ7, 3LZ8, 3LZ9, 3LZA, 3LZB, 3LZC, 3LZD, 3LZE, 3LZF, 3LZG, 3LZI, 3LZJ, 3LZK, 3LZL, 3LZM, 3LZN, 3LZO, 3LZP, 3LZQ, 3LZR, 3LZS, 3LZT, 3LZU, 3LZV, 3LZW, 3LZX, 3LZY, 3LZZ, 3M00, 3M01, 3M02, 3M03, 3M04, 3M05, 3M06, 3M07, 3M08, 3M09, 3M0A, 3M0B, 3M0C, 3M0D, 3M0E, 3M0F, 3M0G, 3M0H, 3M0I, 3M0J, 3M0K, 3M0L, 3M0M, 3M0N, 3M0O, 3M0P, 3M0Q, 3M0R, 3M0S, 3M0T, 3M0U, 3M0V, 3M0W, 3M0X, 3M0Y, 3M0Z, 3M10, 3M11, 3M12, 3M13, 3M14, 3M15, 3M16, 3M17, 3M18, 3M19, 3M1A, 3M1B, 3M1C, 3M1D, 3M1E, 3M1F, 3M1G, 3M1H, 3M1I, 3M1J, 3M1K, 3M1L, 3M1M, 3M1N, 3M1O, 3M1P, 3M1Q, 3M1R, 3M1S, 3M1T, 3M1U, 3M1V, 3M1W, 3M1X, 3M1Y, 3M1Z, 3M20, 3M21, 3M22, 3M23, 3M24, 3M25, 3M26, 3M27, 3M28, 3M29, 3M2A, 3M2B, 3M2C, 3M2D, 3M2E, 3M2F, 3M2G, 3M2H, 3M2I, 3M2J, 3M2K, 3M2L, 3M2M, 3M2N, 3M2P, 3M2R, 3M2T, 3M2U, 3M2V, 3M2W, 3M2X, 3M2Y, 3M2Z, 3M30, 3M31, 3M32, 3M33, 3M34, 3M35, 3M36, 3M37, 3M38, 3M39, 3M3A, 3M3B, 3M3C, 3M3D, 3M3E, 3M3F, 3M3G, 3M3H, 3M3I, 3M3J, 3M3K, 3M3L, 3M3M, 3M3N, 3M3O, 3M3P, 3M3Q, 3M3R, 3M3S, 3M3T, 3M3U, 3M3V, 3M3W, 3M3X, 3M3Z, 3M40, 3M41, 3M42, 3M43, 3M44, 3M45, 3M46, 3M47, 3M48, 3M49, 3M4A, 3M4B, 3M4C, 3M4D, 3M4E, 3M4F, 3M4G, 3M4H, 3M4I, 3M4J, 3M4N, 3M4P, 3M4Q, 3M4R, 3M4S, 3M4T, 3M4U, 3M4V, 3M4W, 3M4X, 3M4Y, 3M4Z, 3M50, 3M51, 3M52, 3M53, 3M54, 3M55, 3M56, 3M57, 3M58, 3M59, 3M5A, 3M5B, 3M5C, 3M5D, 3M5E, 3M5G, 3M5H, 3M5I, 3M5J, 3M5K, 3M5L, 3M5M, 3M5N, 3M5O, 3M5P, 3M5Q, 3M5R, 3M5S, 3M5T, 3M5U, 3M5V, 3M5W, 3M5X, 3M5Y, 3M5Z, 3M61, 3M62, 3M63, 3M64, 3M65, 3M66, 3M67, 3M6A, 3M6B, 3M6C, 3M6D, 3M6E, 3M6F, 3M6G, 3M6H, 3M6I, 3M6J, 3M6K, 3M6L, 3M6M, 3M6N, 3M6O, 3M6P, 3M6Q, 3M6R, 3M6S, 3M6U, 3M6V, 3M6W, 3M6X, 3M6Y, 3M6Z, 3M70, 3M71, 3M72, 3M73, 3M74, 3M75, 3M76, 3M77, 3M78, 3M79, 3M7A, 3M7B, 3M7C, 3M7D, 3M7E, 3M7F, 3M7G, 3M7H, 3M7I, 3M7J, 3M7K, 3M7L, 3M7M, 3M7O, 3M7P, 3M7Q, 3M7R, 3M7S, 3M7T, 3M7U, 3M7V, 3M7W, 3M81, 3M82, 3M83, 3M84, 3M86, 3M88, 3M89, 3M8A, 3M8B, 3M8C, 3M8D, 3M8E, 3M8F, 3M8J, 3M8K, 3M8M, 3M8N, 3M8O, 3M8P, 3M8Q, 3M8R, 3M8S, 3M8T, 3M8U, 3M8V, 3M8W, 3M8Y, 3M8Z, 3M91, 3M92, 3M93, 3M94, 3M95, 3M96, 3M97, 3M98, 3M99, 3M9A, 3M9B, 3M9C, 3M9D, 3M9E, 3M9F, 3M9G, 3M9H, 3M9J, 3M9K, 3M9L, 3M9M, 3M9N, 3M9O, 3M9Q, 3M9S, 3M9U, 3M9V, 3M9W, 3M9X, 3M9Y, 3M9Z, 3MA0, 3MA2, 3MA3, 3MA5, 3MA6, 3MA7, 3MA8, 3MA9, 3MAA, 3MAB, 3MAC, 3MAD, 3MAE, 3MAF, 3MAG, 3MAH, 3MAJ, 3MAK, 3MAL, 3MAM, 3MAN, 3MAO, 3MAP, 3MAQ, 3MAR, 3MAS, 3MAT, 3MAU, 3MAV, 3MAW, 3MAX, 3MAY, 3MAZ, 3MB2, 3MB3, 3MB4, 3MB5, 3MB6, 3MB7, 3MB8, 3MB9, 3MBA, 3MBB, 3MBC, 3MBD, 3MBE, 3MBF, 3MBG, 3MBH, 3MBI, 3MBJ, 3MBK, 3MBL, 3MBM, 3MBO, 3MBP, 3MBQ, 3MBR, 3MBT, 3MBV, 3MBW, 3MBX, 3MBY, 3MBZ, 3MC0, 3MC1, 3MC2, 3MC3, 3MC4, 3MC5, 3MC6, 3MC8, 3MC9, 3MCA, 3MCB, 3MCD, 3MCE, 3MCF, 3MCG, 3MCH, 3MCI, 3MCJ, 3MCK, 3MCL, 3MCM, 3MCN, 3MCO, 3MCP, 3MCQ, 3MCR, 3MCS, 3MCT, 3MCU, 3MCV, 3MCW, 3MCX, 3MCY, 3MCZ, 3MD0, 3MD1, 3MD2, 3MD3, 3MD4, 3MD5, 3MD7, 3MD9, 3MDA, 3MDB, 3MDC, 3MDD, 3MDE, 3MDF, 3MDJ, 3MDK, 3MDL, 3MDM, 3MDN, 3MDO, 3MDP, 3MDQ, 3MDR, 3MDS, 3MDT, 3MDU, 3MDV, 3MDW, 3MDX, 3MDY, 3MDZ, 3ME0, 3ME1, 3ME2, 3ME3, 3ME4, 3ME5, 3ME6, 3ME7, 3ME8, 3ME9, 3MEA, 3MEB, 3MEC, 3MED, 3MEE, 3MEG, 3MEH, 3MEJ, 3MEK, 3MEL, 3MEM, 3MEN, 3MEP, 3MEQ, 3MER, 3MES, 3MET, 3MEU, 3MEV, 3MEW, 3MEX, 3MEY, 3MEZ, 3MF0, 3MF1, 3MF2, 3MF3, 3MF4, 3MF5, 3MF6, 3MF7, 3MF8, 3MF9, 3MFA, 3MFB, 3MFC, 3MFD, 3MFE, 3MFF, 3MFG, 3MFH, 3MFI, 3MFJ, 3MFK, 3MFL, 3MFM, 3MFN, 3MFQ, 3MFR, 3MFS, 3MFT, 3MFU, 3MFV, 3MFW, 3MFX, 3MFY, 3MG0, 3MG1, 3MG2, 3MG3, 3MG4, 3MG5, 3MG6, 3MG7, 3MG8, 3MGA, 3MGB, 3MGC, 3MGD, 3MGE, 3MGF, 3MGG, 3MGH, 3MGI, 3MGJ, 3MGK, 3MGL, 3MGM, 3MGO, 3MGP, 3MGQ, 3MGR, 3MGS, 3MGT, 3MGU, 3MGV, 3MGW, 3MGX, 3MGY, 3MGZ, 3MH0, 3MH1, 3MH2, 3MH3, 3MH4, 3MH5, 3MH6, 3MH7, 3MH8, 3MH9, 3MHA, 3MHB, 3MHC, 3MHD, 3MHE, 3MHF, 3MHG, 3MHH, 3MHI, 3MHJ, 3MHK, 3MHL, 3MHM, 3MHO, 3MHP, 3MHR, 3MHS, 3MHT, 3MHU, 3MHV, 3MHW, 3MHX, 3MHY, 3MHZ, 3MI0, 3MI1, 3MI2, 3MI3, 3MI4, 3MI5, 3MI6, 3MI7, 3MI8, 3MI9, 3MIA, 3MIB, 3MIC, 3MID, 3MIE, 3MIF, 3MIG, 3MIH, 3MII, 3MIL, 3MIM, 3MIN, 3MIO, 3MIP, 3MIS, 3MIT, 3MIU, 3MIV, 3MIW, 3MIX, 3MIY, 3MIZ, 3MJ1, 3MJ2, 3MJ4, 3MJ5, 3MJ6, 3MJ7, 3MJ8, 3MJ9, 3MJC, 3MJD, 3MJE, 3MJF, 3MJG, 3MJH, 3MJI, 3MJK, 3MJL, 3MJM, 3MJN, 3MJO, 3MJP, 3MJQ, 3MJR, 3MJS, 3MJT, 3MJU, 3MJV, 3MJW, 3MJX, 3MJY, 3MJZ, 3MK0, 3MK1, 3MK2, 3MK3, 3MK4, 3MK5, 3MK6, 3MK7, 3MK8, 3MK9, 3MKA, 3MKB, 3MKC, 3MKD, 3MKE, 3MKF, 3MKG, 3MKH, 3MKI, 3MKJ, 3MKK, 3MKL, 3MKM, 3MKN, 3MKO, 3MKP, 3MKQ, 3MKR, 3MKS, 3MKT, 3MKU, 3MKV, 3MKW, 3MKY, 3MKZ, 3ML0, 3ML1, 3ML2, 3ML3, 3ML4, 3ML5, 3ML6, 3ML8, 3ML9, 3MLA, 3MLB, 3MLC, 3MLE, 3MLF, 3MLG, 3MLH, 3MLI, 3MLJ, 3MLK, 3MLL, 3MLM, 3MLN, 3MLO, 3MLP, 3MLQ, 3MLR, 3MLS, 3MLT, 3MLU, 3MLV, 3MLW, 3MLX, 3MLY, 3MLZ, 3MM0, 3MM1, 3MM2, 3MM3, 3MM4, 3MM5, 3MM6, 3MM7, 3MM8, 3MM9, 3MMA, 3MMB, 3MMC, 3MMD, 3MME, 3MMF, 3MMG, 3MMH, 3MMI, 3MMJ, 3MMK, 3MML, 3MMN, 3MMO, 3MMP, 3MMR, 3MMS, 3MMT, 3MMU, 3MMV, 3MMW, 3MMX, 3MMY, 3MMZ, 3MN0, 3MN1, 3MN2, 3MN3, 3MN5, 3MN6, 3MN7, 3MN8, 3MN9, 3MNA, 3MNB, 3MNC, 3MND, 3MNE, 3MNF, 3MNG, 3MNH, 3MNI, 3MNJ, 3MNK, 3MNL, 3MNM, 3MNN, 3MNO, 3MNP, 3MNQ, 3MNR, 3MNS, 3MNU, 3MNV, 3MNW, 3MNX, 3MNZ, 3MO0, 3MO1, 3MO2, 3MO3, 3MO4, 3MO5, 3MO6, 3MO7, 3MO8, 3MO9, 3MOA, 3MOB, 3MOC, 3MOD, 3MOE, 3MOF, 3MOG, 3MOH, 3MOI, 3MOK, 3MOL, 3MOM, 3MON, 3MOO, 3MOP, 3MOQ, 3MOR, 3MOS, 3MOU, 3MOW, 3MOY, 3MOZ, 3MP1, 3MP2, 3MP3, 3MP4, 3MP5, 3MP6, 3MP7, 3MP8, 3MP9, 3MPA, 3MPB, 3MPC, 3MPD, 3MPE, 3MPF, 3MPG, 3MPH, 3MPI, 3MPJ, 3MPK, 3MPL, 3MPM, 3MPN, 3MPO, 3MPP, 3MPQ, 3MPR, 3MPS, 3MPT, 3MPU, 3MPV, 3MPW, 3MPX, 3MPY, 3MPZ, 3MQ0, 3MQ1, 3MQ2, 3MQ3, 3MQ4, 3MQ6, 3MQ7, 3MQ9, 3MQB, 3MQC, 3MQD, 3MQE, 3MQF, 3MQG, 3MQH, 3MQI, 3MQL, 3MQM, 3MQO, 3MQP, 3MQQ, 3MQR, 3MQS, 3MQT, 3MQW, 3MQY, 3MQZ, 3MR0, 3MR1, 3MR2, 3MR3, 3MR5, 3MR6, 3MR7, 3MR9, 3MRB, 3MRC, 3MRD, 3MRE, 3MRF, 3MRG, 3MRH, 3MRI, 3MRJ, 3MRK, 3MRL, 3MRM, 3MRN, 3MRO, 3MRP, 3MRQ, 3MRR, 3MRS, 3MRT, 3MRU, 3MRV, 3MRW, 3MRX, 3MRY, 3MS2, 3MS3, 3MS4, 3MS5, 3MS6, 3MS7, 3MS8, 3MS9, 3MSA, 3MSC, 3MSD, 3MSE, 3MSF, 3MSG, 3MSH, 3MSI, 3MSJ, 3MSK, 3MSL, 3MSN, 3MSO, 3MSQ, 3MSR, 3MSS, 3MST, 3MSU, 3MSV, 3MSW, 3MSX, 3MSY, 3MSZ, 3MT0, 3MT1, 3MT5, 3MT6, 3MT7, 3MT8, 3MT9, 3MTA, 3MTB, 3MTC, 3MTD, 3MTE, 3MTF, 3MTG, 3MTH, 3MTI, 3MTJ, 3MTK, 3MTL, 3MTN, 3MTQ, 3MTR, 3MTS, 3MTT, 3MTU, 3MTV, 3MTW, 3MTX, 3MTY, 3MU0, 3MU1, 3MU3, 3MU4, 3MU5, 3MU6, 3MU7, 3MU8, 3MUA, 3MUC, 3MUD, 3MUE, 3MUF, 3MUG, 3MUH, 3MUI, 3MUJ, 3MUK, 3MUL, 3MUN, 3MUO, 3MUP, 3MUQ, 3MUS, 3MUU, 3MUX, 3MUY, 3MUZ, 3MV0, 3MV1, 3MV2, 3MV3, 3MV4, 3MV5, 3MV6, 3MV7, 3MV8, 3MV9, 3MVA, 3MVB, 3MVC, 3MVD, 3MVE, 3MVF, 3MVG, 3MVH, 3MVI, 3MVJ, 3MVK, 3MVL, 3MVM, 3MVN, 3MVO, 3MVP, 3MVQ, 3MVR, 3MVS, 3MVT, 3MVU, 3MVV, 3MVW, 3MVX, 3MVY, 3MVZ, 3MW0, 3MW1, 3MW2, 3MW3, 3MW4, 3MW6, 3MW7, 3MW8, 3MW9, 3MWA, 3MWB, 3MWC, 3MWD, 3MWE, 3MWF, 3MWG, 3MWH, 3MWI, 3MWJ, 3MWK, 3MWL, 3MWM, 3MWN, 3MWO, 3MWP, 3MWQ, 3MWR, 3MWS, 3MWT, 3MWU, 3MWV, 3MWW, 3MWX, 3MWY, 3MWZ, 3MX0, 3MX1, 3MX2, 3MX3, 3MX4, 3MX5, 3MX6, 3MX7, 3MX8, 3MX9, 3MXA, 3MXB, 3MXC, 3MXD, 3MXE, 3MXF, 3MXG, 3MXI, 3MXJ, 3MXL, 3MXM, 3MXN, 3MXO, 3MXP, 3MXQ, 3MXR, 3MXS, 3MXT, 3MXU, 3MXV, 3MXW, 3MXX, 3MXY, 3MXZ, 3MY0, 3MY1, 3MY2, 3MY5, 3MY6, 3MY7, 3MY9, 3MYA, 3MYB, 3MYC, 3MYD, 3MYE, 3MYF, 3MYG, 3MYH, 3MYI, 3MYJ, 3MYK, 3MYL, 3MYM, 3MYN, 3MYO, 3MYP, 3MYQ, 3MYR, 3MYT, 3MYU, 3MYV, 3MYW, 3MYX, 3MYY, 3MYZ, 3MZ0, 3MZ1, 3MZ2, 3MZ3, 3MZ4, 3MZ5, 3MZ6, 3MZ7, 3MZ8, 3MZ9, 3MZB, 3MZC, 3MZD, 3MZE, 3MZF, 3MZG, 3MZH, 3MZI, 3MZK, 3MZL, 3MZN, 3MZO, 3MZQ, 3MZR, 3MZS, 3MZT, 3MZV, 3MZW, 3MZY, 3MZZ, 3N00, 3N01, 3N02, 3N03, 3N04, 3N05, 3N06, 3N07, 3N08, 3N0A, 3N0B, 3N0C, 3N0D, 3N0E, 3N0F, 3N0G, 3N0H, 3N0I, 3N0K, 3N0L, 3N0M, 3N0N, 3N0P, 3N0Q, 3N0R, 3N0S, 3N0T, 3N0U, 3N0V, 3N0W, 3N0X, 3N0Y, 3N0Z, 3N10, 3N11, 3N12, 3N13, 3N14, 3N15, 3N16, 3N17, 3N18, 3N19, 3N1A, 3N1B, 3N1C, 3N1D, 3N1E, 3N1F, 3N1G, 3N1H, 3N1I, 3N1J, 3N1K, 3N1L, 3N1M, 3N1N, 3N1O, 3N1P, 3N1Q, 3N1R, 3N1S, 3N1T, 3N1U, 3N1V, 3N1W, 3N1X, 3N1Y, 3N1Z, 3N20, 3N21, 3N23, 3N24, 3N25, 3N26, 3N27, 3N28, 3N29, 3N2A, 3N2B, 3N2C, 3N2D, 3N2E, 3N2F, 3N2G, 3N2I, 3N2J, 3N2K, 3N2L, 3N2M, 3N2N, 3N2O, 3N2P, 3N2Q, 3N2R, 3N2S, 3N2T, 3N2U, 3N2V, 3N2W, 3N2X, 3N2Y, 3N2Z, 3N30, 3N31, 3N32, 3N33, 3N34, 3N35, 3N36, 3N37, 3N38, 3N39, 3N3A, 3N3B, 3N3C, 3N3D, 3N3E, 3N3F, 3N3G, 3N3H, 3N3I, 3N3J, 3N3K, 3N3L, 3N3M, 3N3N, 3N3O, 3N3P, 3N3Q, 3N3R, 3N3S, 3N3T, 3N3U, 3N3W, 3N3X, 3N3Y, 3N3Z, 3N40, 3N41, 3N42, 3N43, 3N44, 3N45, 3N46, 3N49, 3N4A, 3N4B, 3N4C, 3N4D, 3N4E, 3N4F, 3N4G, 3N4H, 3N4I, 3N4J, 3N4K, 3N4L, 3N4M, 3N4N, 3N4O, 3N4P, 3N4Q, 3N4R, 3N4S, 3N4T, 3N4U, 3N4V, 3N4W, 3N4X, 3N4Y, 3N4Z, 3N50, 3N51, 3N52, 3N53, 3N54, 3N55, 3N56, 3N57, 3N58, 3N59, 3N5A, 3N5B, 3N5C, 3N5D, 3N5E, 3N5F, 3N5G, 3N5H, 3N5I, 3N5J, 3N5K, 3N5L, 3N5M, 3N5N, 3N5O, 3N5P, 3N5Q, 3N5R, 3N5S, 3N5T, 3N5U, 3N5V, 3N5W, 3N5X, 3N5Y, 3N5Z, 3N60, 3N61, 3N62, 3N63, 3N64, 3N65, 3N66, 3N67, 3N68, 3N69, 3N6A, 3N6B, 3N6C, 3N6D, 3N6E, 3N6F, 3N6G, 3N6H, 3N6I, 3N6J, 3N6K, 3N6L, 3N6M, 3N6N, 3N6O, 3N6Q, 3N6R, 3N6S, 3N6T, 3N6U, 3N6V, 3N6W, 3N6X, 3N6Y, 3N6Z, 3N70, 3N71, 3N72, 3N73, 3N74, 3N75, 3N76, 3N77, 3N78, 3N79, 3N7A, 3N7B, 3N7C, 3N7D, 3N7E, 3N7H, 3N7J, 3N7K, 3N7L, 3N7M, 3N7N, 3N7O, 3N7P, 3N7Q, 3N7R, 3N7S, 3N7T, 3N7U, 3N7W, 3N7X, 3N7Y, 3N7Z, 3N80, 3N81, 3N82, 3N83, 3N84, 3N85, 3N86, 3N87, 3N89, 3N8B, 3N8D, 3N8E, 3N8F, 3N8G, 3N8H, 3N8I, 3N8K, 3N8L, 3N8M, 3N8N, 3N8R, 3N8S, 3N8T, 3N8U, 3N8V, 3N8W, 3N8X, 3N8Y, 3N8Z, 3N90, 3N91, 3N92, 3N93, 3N94, 3N95, 3N96, 3N97, 3N98, 3N99, 3N9A, 3N9B, 3N9C, 3N9D, 3N9E, 3N9G, 3N9H, 3N9I, 3N9J, 3N9K, 3N9L, 3N9M, 3N9N, 3N9O, 3N9P, 3N9Q, 3N9R, 3N9S, 3N9T, 3N9U, 3N9V, 3N9W, 3N9X, 3N9Y, 3N9Z, 3NA0, 3NA1, 3NA2, 3NA3, 3NA4, 3NA5, 3NA6, 3NA7, 3NA8, 3NA9, 3NAA, 3NAB, 3NAC, 3NAD, 3NAE, 3NAF, 3NAG, 3NAH, 3NAI, 3NAK, 3NAL, 3NAM, 3NAN, 3NAO, 3NAQ, 3NAR, 3NAS, 3NAT, 3NAU, 3NAV, 3NAW, 3NAX, 3NAY, 3NAZ, 3NB0, 3NB2, 3NB5, 3NB6, 3NB7, 3NB8, 3NB9, 3NBA, 3NBB, 3NBC, 3NBD, 3NBE, 3NBF, 3NBH, 3NBI, 3NBJ, 3NBK, 3NBL, 3NBM, 3NBN, 3NBP, 3NBQ, 3NBR, 3NBS, 3NBT, 3NBU, 3NBV, 3NBW, 3NBX, 3NBY, 3NBZ, 3NC0, 3NC1, 3NC2, 3NC3, 3NC4, 3NC5, 3NC6, 3NC7, 3NC8, 3NC9, 3NCA, 3NCB, 3NCC, 3NCE, 3NCF, 3NCG, 3NCH, 3NCI, 3NCJ, 3NCK, 3NCL, 3NCO, 3NCP, 3NCQ, 3NCR, 3NCT, 3NCV, 3NCW, 3NCX, 3NCY, 3NCZ, 3ND0, 3ND1, 3ND2, 3ND5, 3ND6, 3ND7, 3ND8, 3ND9, 3NDA, 3NDC, 3NDD, 3NDE, 3NDF, 3NDG, 3NDH, 3NDI, 3NDJ, 3NDK, 3NDM, 3NDN, 3NDO, 3NDP, 3NDQ, 3NDR, 3NDS, 3NDT, 3NDU, 3NDV, 3NDW, 3NDX, 3NDY, 3NDZ, 3NE0, 3NE1, 3NE2, 3NE3, 3NE4, 3NE5, 3NE6, 3NE7, 3NE8, 3NE9, 3NEA, 3NEC, 3NED, 3NEE, 3NEF, 3NEG, 3NEH, 3NEI, 3NEJ, 3NEK, 3NEL, 3NEM, 3NEN, 3NEO, 3NEP, 3NEQ, 3NER, 3NES, 3NET, 3NEU, 3NEV, 3NEW, 3NEX, 3NEY, 3NEZ, 3NF0, 3NF1, 3NF2, 3NF3, 3NF4, 3NF5, 3NF6, 3NF7, 3NF8, 3NF9, 3NFA, 3NFB, 3NFC, 3NFD, 3NFE, 3NFF, 3NFG, 3NFH, 3NFI, 3NFK, 3NFL, 3NFM, 3NFN, 3NFP, 3NFQ, 3NFR, 3NFS, 3NFT, 3NFU, 3NFV, 3NFW, 3NFY, 3NFZ, 3NG0, 3NG1, 3NG2, 3NG3, 3NG4, 3NG5, 3NG6, 3NG7, 3NG8, 3NGA, 3NGB, 3NGC, 3NGD, 3NGF, 3NGG, 3NGH, 3NGI, 3NGJ, 3NGK, 3NGL, 3NGM, 3NGN, 3NGO, 3NGP, 3NGQ, 3NGR, 3NGS, 3NGT, 3NGU, 3NGV, 3NGW, 3NGX, 3NGY, 3NGZ, 3NH0, 3NH1, 3NH2, 3NH3, 3NH4, 3NH5, 3NH6, 3NH7, 3NH8, 3NH9, 3NHA, 3NHB, 3NHC, 3NHD, 3NHE, 3NHF, 3NHG, 3NHH, 3NHI, 3NHJ, 3NHK, 3NHL, 3NHM, 3NHN, 3NHO, 3NHP, 3NHQ, 3NHR, 3NHS, 3NHT, 3NHU, 3NHV, 3NHW, 3NHX, 3NHY, 3NHZ, 3NI0, 3NI2, 3NI5, 3NI6, 3NI7, 3NI8, 3NI9, 3NIA, 3NIB, 3NIC, 3NID, 3NIE, 3NIF, 3NIG, 3NIH, 3NII, 3NIJ, 3NIK, 3NIL, 3NIM, 3NIN, 3NIO, 3NIP, 3NIQ, 3NIR, 3NIS, 3NIT, 3NIU, 3NIV, 3NIW, 3NIX, 3NIY, 3NIZ, 3NJ0, 3NJ1, 3NJ2, 3NJ3, 3NJ4, 3NJ5, 3NJ8, 3NJ9, 3NJA, 3NJB, 3NJC, 3NJD, 3NJE, 3NJF, 3NJG, 3NJH, 3NJI, 3NJJ, 3NJK, 3NJL, 3NJM, 3NJN, 3NJO, 3NJP, 3NJQ, 3NJR, 3NJS, 3NJT, 3NJU, 3NJV, 3NJW, 3NJX, 3NJY, 3NJZ, 3NK0, 3NK1, 3NK2, 3NK3, 3NK4, 3NK5, 3NK6, 3NK7, 3NK8, 3NK9, 3NKA, 3NKC, 3NKD, 3NKE, 3NKF, 3NKG, 3NKH, 3NKJ, 3NKK, 3NKL, 3NKM, 3NKN, 3NKO, 3NKP, 3NKQ, 3NKR, 3NKS, 3NKT, 3NKU, 3NKV, 3NKW, 3NKX, 3NKY, 3NKZ, 3NL1, 3NL2, 3NL3, 3NL5, 3NL6, 3NL7, 3NL9, 3NLB, 3NLC, 3NLD, 3NLE, 3NLF, 3NLG, 3NLH, 3NLI, 3NLJ, 3NLK, 3NLL, 3NLM, 3NLN, 3NLO, 3NLP, 3NLQ, 3NLR, 3NLS, 3NLT, 3NLU, 3NLV, 3NLW, 3NLX, 3NLY, 3NLZ, 3NM0, 3NM1, 3NM2, 3NM3, 3NM4, 3NM5, 3NM6, 3NM7, 3NM8, 3NM9, 3NMB, 3NMD, 3NME, 3NMH, 3NMI, 3NMJ, 3NMK, 3NML, 3NMM, 3NMN, 3NMO, 3NMP, 3NMQ, 3NMS, 3NMT, 3NMV, 3NMW, 3NMX, 3NMZ, 3NN0, 3NN1, 3NN2, 3NN3, 3NN4, 3NN6, 3NN7, 3NN8, 3NN9, 3NNB, 3NND, 3NNE, 3NNF, 3NNG, 3NNJ, 3NNK, 3NNL, 3NNM, 3NNN, 3NNO, 3NNQ, 3NNR, 3NNS, 3NNT, 3NNU, 3NNV, 3NNW, 3NNX, 3NNY, 3NNZ, 3NO0, 3NO1, 3NO2, 3NO3, 3NO4, 3NO5, 3NO6, 3NO7, 3NO8, 3NO9, 3NOA, 3NOB, 3NOC, 3NOD, 3NOE, 3NOF, 3NOG, 3NOH, 3NOI, 3NOJ, 3NOK, 3NOL, 3NOM, 3NON, 3NOO, 3NOP, 3NOQ, 3NOR, 3NOS, 3NOT, 3NOU, 3NOV, 3NOW, 3NOX, 3NOY, 3NOZ, 3NP0, 3NP1, 3NP2, 3NP3, 3NP4, 3NP5, 3NP6, 3NP7, 3NP8, 3NP9, 3NPA, 3NPC, 3NPD, 3NPE, 3NPF, 3NPG, 3NPH, 3NPI, 3NPK, 3NPL, 3NPM, 3NPO, 3NPP, 3NPR, 3NPS, 3NPU, 3NPV, 3NPW, 3NPX, 3NPY, 3NPZ, 3NQ0, 3NQ1, 3NQ2, 3NQ3, 3NQ4, 3NQ5, 3NQ6, 3NQ7, 3NQ8, 3NQ9, 3NQA, 3NQB, 3NQC, 3NQD, 3NQE, 3NQF, 3NQG, 3NQH, 3NQI, 3NQJ, 3NQK, 3NQM, 3NQN, 3NQO, 3NQP, 3NQR, 3NQS, 3NQT, 3NQU, 3NQV, 3NQW, 3NQX, 3NQY, 3NQZ, 3NR0, 3NR1, 3NR2, 3NR3, 3NR4, 3NR5, 3NR6, 3NR7, 3NR8, 3NR9, 3NRA, 3NRB, 3NRC, 3NRD, 3NRE, 3NRF, 3NRG, 3NRH, 3NRI, 3NRJ, 3NRK, 3NRL, 3NRM, 3NRN, 3NRO, 3NRP, 3NRQ, 3NRR, 3NRS, 3NRT, 3NRU, 3NRV, 3NRW, 3NRX, 3NRY, 3NRZ, 3NS0, 3NS1, 3NS2, 3NS4, 3NS5, 3NS6, 3NS7, 3NS8, 3NS9, 3NSB, 3NSC, 3NSD, 3NSE, 3NSF, 3NSG, 3NSH, 3NSI, 3NSJ, 3NSK, 3NSL, 3NSM, 3NSN, 3NSO, 3NSP, 3NSQ, 3NSS, 3NST, 3NSU, 3NSW, 3NSX, 3NSY, 3NSZ, 3NT0, 3NT1, 3NT2, 3NT3, 3NT4, 3NT5, 3NT6, 3NT7, 3NT8, 3NT9, 3NTA, 3NTB, 3NTC, 3NTD, 3NTE, 3NTG, 3NTH, 3NTI, 3NTJ, 3NTK, 3NTL, 3NTM, 3NTN, 3NTO, 3NTP, 3NTQ, 3NTR, 3NTS, 3NTU, 3NTV, 3NTW, 3NTX, 3NTY, 3NTZ, 3NU0, 3NU1, 3NU3, 3NU4, 3NU5, 3NU6, 3NU7, 3NU8, 3NU9, 3NUA, 3NUB, 3NUC, 3NUD, 3NUE, 3NUF, 3NUG, 3NUH, 3NUI, 3NUJ, 3NUK, 3NUL, 3NUM, 3NUN, 3NUO, 3NUP, 3NUQ, 3NUR, 3NUS, 3NUT, 3NUU, 3NUV, 3NUX, 3NUY, 3NUZ, 3NV0, 3NV1, 3NV2, 3NV3, 3NV4, 3NV5, 3NV6, 3NV7, 3NV8, 3NV9, 3NVA, 3NVC, 3NVD, 3NVE, 3NVF, 3NVG, 3NVH, 3NVJ, 3NVL, 3NVM, 3NVN, 3NVO, 3NVQ, 3NVR, 3NVS, 3NVT, 3NVU, 3NVV, 3NVW, 3NVX, 3NVY, 3NVZ, 3NW0, 3NW2, 3NW3, 3NW4, 3NW5, 3NW6, 3NW7, 3NW8, 3NW9, 3NWA, 3NWB, 3NWC, 3NWD, 3NWE, 3NWF, 3NWG, 3NWH, 3NWI, 3NWJ, 3NWK, 3NWL, 3NWM, 3NWN, 3NWO, 3NWP, 3NWQ, 3NWR, 3NWS, 3NWT, 3NWU, 3NWV, 3NWW, 3NWX, 3NWY, 3NWZ, 3NX0, 3NX1, 3NX2, 3NX3, 3NX4, 3NX5, 3NX6, 3NX7, 3NX8, 3NX9, 3NXA, 3NXB, 3NXC, 3NXD, 3NXE, 3NXF, 3NXG, 3NXH, 3NXJ, 3NXK, 3NXL, 3NXN, 3NXO, 3NXP, 3NXQ, 3NXR, 3NXS, 3NXT, 3NXU, 3NXV, 3NXW, 3NXX, 3NXY, 3NXZ, 3NY0, 3NY1, 3NY2, 3NY3, 3NY4, 3NY5, 3NY6, 3NY7, 3NY8, 3NY9, 3NYA, 3NYB, 3NYC, 3NYD, 3NYE, 3NYF, 3NYG, 3NYH, 3NYI, 3NYJ, 3NYK, 3NYL, 3NYM, 3NYN, 3NYO, 3NYP, 3NYQ, 3NYR, 3NYS, 3NYT, 3NYU, 3NYV, 3NYW, 3NYX, 3NYY, 3NYZ, 3NZ0, 3NZ1, 3NZ2, 3NZ3, 3NZ4, 3NZ6, 3NZ7, 3NZ8, 3NZ9, 3NZA, 3NZB, 3NZC, 3NZD, 3NZE, 3NZG, 3NZH, 3NZI, 3NZJ, 3NZK, 3NZL, 3NZM, 3NZN, 3NZP, 3NZQ, 3NZR, 3NZS, 3NZT, 3NZU, 3NZW, 3NZX, 3NZZ, 3O00, 3O01, 3O02, 3O03, 3O04, 3O05, 3O06, 3O07, 3O08, 3O0A, 3O0D, 3O0E, 3O0F, 3O0G, 3O0H, 3O0I, 3O0J, 3O0K, 3O0L, 3O0M, 3O0N, 3O0O, 3O0P, 3O0Q, 3O0R, 3O0T, 3O0U, 3O0V, 3O0W, 3O0X, 3O0Y, 3O0Z, 3O10, 3O11, 3O12, 3O13, 3O14, 3O15, 3O16, 3O17, 3O18, 3O19, 3O1A, 3O1B, 3O1C, 3O1D, 3O1E, 3O1F, 3O1G, 3O1H, 3O1I, 3O1J, 3O1K, 3O1L, 3O1M, 3O1N, 3O1O, 3O1P, 3O1Q, 3O1R, 3O1S, 3O1T, 3O1U, 3O1V, 3O1W, 3O1X, 3O1Y, 3O1Z, 3O20, 3O21, 3O22, 3O23, 3O24, 3O26, 3O27, 3O28, 3O29, 3O2A, 3O2B, 3O2C, 3O2D, 3O2E, 3O2F, 3O2G, 3O2H, 3O2I, 3O2J, 3O2K, 3O2L, 3O2M, 3O2N, 3O2O, 3O2P, 3O2Q, 3O2R, 3O2S, 3O2T, 3O2U, 3O2V, 3O2W, 3O2X, 3O2Y, 3O31, 3O32, 3O33, 3O34, 3O35, 3O36, 3O37, 3O38, 3O39, 3O3A, 3O3B, 3O3C, 3O3D, 3O3E, 3O3F, 3O3G, 3O3H, 3O3J, 3O3K, 3O3L, 3O3M, 3O3N, 3O3O, 3O3P, 3O3Q, 3O3R, 3O3T, 3O3U, 3O3V, 3O3W, 3O3X, 3O3Z, 3O40, 3O41, 3O42, 3O43, 3O44, 3O45, 3O46, 3O47, 3O48, 3O49, 3O4A, 3O4B, 3O4C, 3O4D, 3O4F, 3O4G, 3O4H, 3O4I, 3O4J, 3O4K, 3O4L, 3O4M, 3O4N, 3O4O, 3O4P, 3O4Q, 3O4R, 3O4S, 3O4T, 3O4U, 3O4V, 3O4W, 3O4X, 3O4Y, 3O4Z, 3O50, 3O51, 3O52, 3O53, 3O55, 3O56, 3O57, 3O59, 3O5A, 3O5B, 3O5C, 3O5D, 3O5E, 3O5F, 3O5G, 3O5I, 3O5J, 3O5K, 3O5L, 3O5M, 3O5N, 3O5O, 3O5P, 3O5Q, 3O5R, 3O5S, 3O5T, 3O5U, 3O5V, 3O5W, 3O5X, 3O5Y, 3O5Z, 3O60, 3O61, 3O62, 3O63, 3O64, 3O65, 3O66, 3O69, 3O6A, 3O6B, 3O6C, 3O6D, 3O6F, 3O6G, 3O6H, 3O6I, 3O6J, 3O6K, 3O6L, 3O6M, 3O6N, 3O6O, 3O6P, 3O6Q, 3O6R, 3O6T, 3O6U, 3O6V, 3O6W, 3O6X, 3O6Y, 3O6Z, 3O70, 3O71, 3O72, 3O73, 3O74, 3O75, 3O76, 3O77, 3O78, 3O79, 3O7A, 3O7B, 3O7H, 3O7I, 3O7J, 3O7K, 3O7L, 3O7M, 3O7N, 3O7O, 3O7P, 3O7Q, 3O7R, 3O7S, 3O7T, 3O7U, 3O7W, 3O7X, 3O80, 3O81, 3O82, 3O83, 3O84, 3O85, 3O86, 3O87, 3O88, 3O89, 3O8A, 3O8B, 3O8D, 3O8E, 3O8G, 3O8H, 3O8I, 3O8J, 3O8L, 3O8M, 3O8N, 3O8O, 3O8P, 3O8Q, 3O8S, 3O8T, 3O8U, 3O8V, 3O8W, 3O8X, 3O8Y, 3O8Z, 3O90, 3O91, 3O92, 3O93, 3O94, 3O95, 3O96, 3O97, 3O98, 3O99, 3O9A, 3O9B, 3O9C, 3O9D, 3O9E, 3O9F, 3O9G, 3O9H, 3O9I, 3O9J, 3O9K, 3O9L, 3O9M, 3O9N, 3O9O, 3O9P, 3O9Q, 3O9R, 3O9S, 3O9T, 3O9U, 3O9V, 3O9W, 3O9X, 3O9Z, 3OA0, 3OA1, 3OA2, 3OA3, 3OA4, 3OA5, 3OA6, 3OA7, 3OA8, 3OA9, 3OAA, 3OAB, 3OAC, 3OAD, 3OAE, 3OAF, 3OAG, 3OAI, 3OAJ, 3OAK, 3OAM, 3OAN, 3OAO, 3OAP, 3OAU, 3OAW, 3OAX, 3OAY, 3OAZ, 3OB0, 3OB1, 3OB2, 3OB4, 3OB6, 3OB7, 3OB8, 3OB9, 3OBA, 3OBB, 3OBE, 3OBF, 3OBG, 3OBH, 3OBI, 3OBJ, 3OBK, 3OBL, 3OBP, 3OBQ, 3OBR, 3OBS, 3OBT, 3OBU, 3OBV, 3OBW, 3OBX, 3OBY, 3OBZ, 3OC0, 3OC1, 3OC2, 3OC3, 3OC4, 3OC5, 3OC6, 3OC7, 3OC8, 3OC9, 3OCA, 3OCB, 3OCC, 3OCD, 3OCE, 3OCF, 3OCG, 3OCH, 3OCI, 3OCJ, 3OCL, 3OCM, 3OCN, 3OCO, 3OCP, 3OCQ, 3OCR, 3OCS, 3OCT, 3OCU, 3OCV, 3OCW, 3OCX, 3OCY, 3OCZ, 3OD0, 3OD1, 3OD2, 3OD3, 3OD4, 3OD5, 3OD6, 3OD7, 3OD8, 3OD9, 3ODA, 3ODB, 3ODC, 3ODD, 3ODE, 3ODF, 3ODG, 3ODH, 3ODI, 3ODJ, 3ODK, 3ODL, 3ODM, 3ODN, 3ODO, 3ODP, 3ODQ, 3ODR, 3ODS, 3ODT, 3ODU, 3ODV, 3ODW, 3ODX, 3ODY, 3ODZ, 3OE0, 3OE1, 3OE2, 3OE3, 3OE4, 3OE5, 3OE6, 3OE7, 3OE8, 3OE9, 3OEA, 3OEB, 3OEC, 3OED, 3OEE, 3OEF, 3OEH, 3OEI, 3OEK, 3OEL, 3OEM, 3OEN, 3OEO, 3OEP, 3OEQ, 3OER, 3OES, 3OET, 3OEU, 3OEV, 3OEW, 3OEX, 3OEY, 3OEZ, 3OF0, 3OF1, 3OF2, 3OF3, 3OF4, 3OF5, 3OF6, 3OF7, 3OF8, 3OF9, 3OFE, 3OFF, 3OFG, 3OFH, 3OFI, 3OFJ, 3OFK, 3OFL, 3OFM, 3OFN, 3OFS, 3OFT, 3OFU, 3OFV, 3OFW, 3OG2, 3OG3, 3OG4, 3OG5, 3OG6, 3OG7, 3OG9, 3OGA, 3OGB, 3OGC, 3OGD, 3OGF, 3OGG, 3OGH, 3OGI, 3OGJ, 3OGK, 3OGL, 3OGM, 3OGN, 3OGO, 3OGP, 3OGQ, 3OGR, 3OGS, 3OGT, 3OGU, 3OGV, 3OGW, 3OGX, 3OGZ, 3OH0, 3OH1, 3OH2, 3OH3, 3OH4, 3OH6, 3OH8, 3OH9, 3OHA, 3OHB, 3OHE, 3OHF, 3OHG, 3OHH, 3OHI, 3OHL, 3OHM, 3OHN, 3OHO, 3OHP, 3OHR, 3OHS, 3OHT, 3OHU, 3OHV, 3OHW, 3OHX, 3OI7, 3OI8, 3OI9, 3OIA, 3OIB, 3OIC, 3OID, 3OIE, 3OIF, 3OIG, 3OIH, 3OII, 3OIK, 3OIL, 3OIM, 3OIO, 3OIP, 3OIQ, 3OIR, 3OIS, 3OIT, 3OIU, 3OIV, 3OIW, 3OIX, 3OIY, 3OIZ, 3OJ0, 3OJ1, 3OJ2, 3OJ3, 3OJ4, 3OJ5, 3OJ6, 3OJ7, 3OJ8, 3OJA, 3OJB, 3OJC, 3OJD, 3OJE, 3OJF, 3OJG, 3OJI, 3OJJ, 3OJK, 3OJL, 3OJM, 3OJN, 3OJO, 3OJP, 3OJS, 3OJT, 3OJU, 3OJV, 3OJW, 3OJX, 3OJY, 3OK0, 3OK5, 3OK8, 3OK9, 3OKA, 3OKC, 3OKD, 3OKE, 3OKF, 3OKG, 3OKH, 3OKI, 3OKJ, 3OKK, 3OKL, 3OKM, 3OKN, 3OKO, 3OKP, 3OKQ, 3OKR, 3OKS, 3OKT, 3OKU, 3OKV, 3OKW, 3OKX, 3OKY, 3OKZ, 3OL0, 3OL2, 3OL3, 3OL4, 3OL5, 3OLC, 3OLD, 3OLE, 3OLF, 3OLG, 3OLH, 3OLI, 3OLJ, 3OLK, 3OLL, 3OLM, 3OLN, 3OLO, 3OLP, 3OLQ, 3OLR, 3OLS, 3OLT, 3OLU, 3OLV, 3OLW, 3OLX, 3OLY, 3OLZ, 3OM0, 3OM1, 3OM2, 3OM3, 3OM4, 3OM5, 3OM6, 3OM7, 3OM8, 3OM9, 3OMA, 3OMB, 3OMC, 3OMD, 3OME, 3OMF, 3OMG, 3OMH, 3OMI, 3OMK, 3OML, 3OMM, 3OMN, 3OMO, 3OMP, 3OMQ, 3OMS, 3OMT, 3OMU, 3OMV, 3OMW, 3OMX, 3OMY, 3OMZ, 3ON0, 3ON1, 3ON2, 3ON3, 3ON4, 3ON5, 3ON6, 3ON7, 3ON9, 3ONA, 3ONB, 3ONC, 3OND, 3ONE, 3ONF, 3ONG, 3ONH, 3ONI, 3ONJ, 3ONK, 3ONL, 3ONM, 3ONN, 3ONO, 3ONP, 3ONQ, 3ONR, 3ONS, 3ONT, 3ONU, 3ONV, 3ONW, 3ONX, 3ONY, 3ONZ, 3OO0, 3OO1, 3OO2, 3OO3, 3OO4, 3OO5, 3OO6, 3OO7, 3OO8, 3OO9, 3OOA, 3OOB, 3OOC, 3OOD, 3OOE, 3OOF, 3OOG, 3OOH, 3OOI, 3OOJ, 3OOK, 3OOL, 3OOM, 3OON, 3OOO, 3OOP, 3OOQ, 3OOR, 3OOS, 3OOT, 3OOU, 3OOV, 3OOW, 3OOX, 3OOY, 3OOZ, 3OP0, 3OP1, 3OP2, 3OP3, 3OP4, 3OP5, 3OP6, 3OP7, 3OP8, 3OP9, 3OPB, 3OPC, 3OPD, 3OPE, 3OPF, 3OPG, 3OPH, 3OPI, 3OPK, 3OPL, 3OPM, 3OPN, 3OPO, 3OPP, 3OPQ, 3OPR, 3OPS, 3OPT, 3OPU, 3OPV, 3OPW, 3OPX, 3OPY, 3OPZ, 3OQ0, 3OQ1, 3OQ2, 3OQ3, 3OQ4, 3OQ5, 3OQ6, 3OQ7, 3OQ8, 3OQ9, 3OQA, 3OQB, 3OQC, 3OQD, 3OQE, 3OQF, 3OQG, 3OQH, 3OQI, 3OQJ, 3OQK, 3OQL, 3OQM, 3OQN, 3OQO, 3OQP, 3OQQ, 3OQR, 3OQS, 3OQT, 3OQU, 3OQV, 3OQY, 3OQZ, 3OR0, 3OR1, 3OR2, 3OR3, 3OR5, 3OR6, 3OR7, 3ORC, 3ORD, 3ORE, 3ORF, 3ORG, 3ORH, 3ORI, 3ORJ, 3ORK, 3ORL, 3ORM, 3ORN, 3ORO, 3ORP, 3ORQ, 3ORR, 3ORS, 3ORT, 3ORU, 3ORV, 3ORW, 3ORX, 3ORY, 3ORZ, 3OS0, 3OS1, 3OS2, 3OS3, 3OS4, 3OS5, 3OS6, 3OS7, 3OS8, 3OS9, 3OSA, 3OSC, 3OSD, 3OSE, 3OSF, 3OSG, 3OSH, 3OSI, 3OSJ, 3OSK, 3OSL, 3OSM, 3OSN, 3OSO, 3OSP, 3OSQ, 3OSR, 3OSS, 3OST, 3OSU, 3OSV, 3OSW, 3OSX, 3OSY, 3OSZ, 3OT1, 3OT2, 3OT3, 3OT4, 3OT5, 3OT6, 3OT7, 3OT8, 3OT9, 3OTB, 3OTC, 3OTD, 3OTE, 3OTF, 3OTG, 3OTH, 3OTI, 3OTJ, 3OTK, 3OTL, 3OTM, 3OTN, 3OTP, 3OTQ, 3OTR, 3OTS, 3OTT, 3OTU, 3OTV, 3OTW, 3OTX, 3OTY, 3OTZ, 3OU0, 3OU1, 3OU2, 3OU3, 3OU4, 3OU5, 3OU6, 3OU7, 3OU8, 3OU9, 3OUA, 3OUB, 3OUC, 3OUD, 3OUE, 3OUF, 3OUG, 3OUH, 3OUI, 3OUJ, 3OUK, 3OUL, 3OUM, 3OUN, 3OUO, 3OUP, 3OUQ, 3OUR, 3OUS, 3OUT, 3OUU, 3OUV, 3OUW, 3OUX, 3OUZ, 3OV0, 3OV1, 3OV2, 3OV3, 3OV4, 3OV5, 3OV6, 3OV8, 3OV9, 3OVE, 3OVG, 3OVK, 3OVM, 3OVN, 3OVO, 3OVP, 3OVQ, 3OVR, 3OVU, 3OVV, 3OVW, 3OVX, 3OVZ, 3OW1, 3OW3, 3OW4, 3OW5, 3OW6, 3OW7, 3OW8, 3OW9, 3OWA, 3OWB, 3OWC, 3OWD, 3OWE, 3OWF, 3OWG, 3OWH, 3OWJ, 3OWK, 3OWL, 3OWM, 3OWN, 3OWO, 3OWP, 3OWQ, 3OWR, 3OWS, 3OWT, 3OWU, 3OWV, 3OWX, 3OWY, 3OX1, 3OX2, 3OX3, 3OX4, 3OX5, 3OX6, 3OX7, 3OX8, 3OX9, 3OXA, 3OXC, 3OXF, 3OXG, 3OXH, 3OXI, 3OXK, 3OXL, 3OXN, 3OXO, 3OXP, 3OXQ, 3OXR, 3OXS, 3OXT, 3OXU, 3OXV, 3OXW, 3OXX, 3OXZ, 3OY0, 3OY1, 3OY2, 3OY3, 3OY4, 3OY5, 3OY6, 3OY7, 3OY8, 3OY9, 3OYA, 3OYB, 3OYC, 3OYD, 3OYE, 3OYF, 3OYG, 3OYH, 3OYI, 3OYJ, 3OYK, 3OYL, 3OYM, 3OYN, 3OYO, 3OYP, 3OYQ, 3OYR, 3OYS, 3OYT, 3OYV, 3OYW, 3OYX, 3OYY, 3OYZ, 3OZ0, 3OZ1, 3OZ2, 3OZ3, 3OZ4, 3OZ5, 3OZ6, 3OZ7, 3OZ9, 3OZA, 3OZB, 3OZC, 3OZD, 3OZE, 3OZF, 3OZG, 3OZH, 3OZI, 3OZJ, 3OZK, 3OZL, 3OZM, 3OZO, 3OZP, 3OZQ, 3OZR, 3OZS, 3OZT, 3OZU, 3OZV, 3OZW, 3OZX, 3OZY, 3OZZ, 3P01, 3P02, 3P03, 3P04, 3P05, 3P06, 3P08, 3P09, 3P0A, 3P0B, 3P0C, 3P0E, 3P0F, 3P0G, 3P0H, 3P0I, 3P0J, 3P0K, 3P0L, 3P0M, 3P0N, 3P0P, 3P0Q, 3P0R, 3P0T, 3P0U, 3P0V, 3P0W, 3P0X, 3P0Y, 3P0Z, 3P10, 3P11, 3P12, 3P13, 3P14, 3P16, 3P17, 3P19, 3P1A, 3P1B, 3P1C, 3P1D, 3P1E, 3P1F, 3P1G, 3P1H, 3P1I, 3P1J, 3P1L, 3P1M, 3P1N, 3P1O, 3P1P, 3P1Q, 3P1R, 3P1S, 3P1T, 3P1U, 3P1V, 3P1W, 3P1X, 3P1Y, 3P1Z, 3P20, 3P23, 3P24, 3P26, 3P27, 3P28, 3P2A, 3P2B, 3P2C, 3P2D, 3P2E, 3P2F, 3P2H, 3P2I, 3P2J, 3P2K, 3P2L, 3P2M, 3P2N, 3P2O, 3P2P, 3P2Q, 3P2R, 3P2S, 3P2T, 3P2U, 3P2V, 3P2W, 3P2X, 3P2Y, 3P2Z, 3P30, 3P31, 3P32, 3P33, 3P34, 3P35, 3P36, 3P37, 3P38, 3P39, 3P3A, 3P3B, 3P3C, 3P3D, 3P3E, 3P3F, 3P3G, 3P3H, 3P3I, 3P3J, 3P3K, 3P3L, 3P3N, 3P3O, 3P3P, 3P3Q, 3P3R, 3P3S, 3P3T, 3P3U, 3P3V, 3P3W, 3P3X, 3P3Y, 3P3Z, 3P40, 3P41, 3P42, 3P43, 3P44, 3P45, 3P47, 3P48, 3P4E, 3P4F, 3P4G, 3P4H, 3P4I, 3P4J, 3P4K, 3P4L, 3P4M, 3P4N, 3P4O, 3P4P, 3P4Q, 3P4R, 3P4S, 3P4T, 3P4U, 3P4V, 3P4W, 3P4X, 3P4Y, 3P4Z, 3P50, 3P51, 3P52, 3P53, 3P54, 3P55, 3P56, 3P57, 3P58, 3P5A, 3P5B, 3P5C, 3P5D, 3P5E, 3P5F, 3P5G, 3P5H, 3P5I, 3P5J, 3P5K, 3P5L, 3P5M, 3P5N, 3P5O, 3P5P, 3P5Q, 3P5R, 3P5S, 3P5T, 3P5U, 3P5V, 3P5W, 3P5X, 3P5Y, 3P5Z, 3P60, 3P61, 3P62, 3P63, 3P64, 3P65, 3P66, 3P67, 3P68, 3P69, 3P6A, 3P6B, 3P6C, 3P6D, 3P6E, 3P6F, 3P6G, 3P6H, 3P6I, 3P6J, 3P6K, 3P6L, 3P6M, 3P6N, 3P6O, 3P6P, 3P6Q, 3P6R, 3P6S, 3P6T, 3P6U, 3P6V, 3P6W, 3P6X, 3P6Z, 3P70, 3P71, 3P72, 3P73, 3P74, 3P75, 3P76, 3P77, 3P78, 3P79, 3P7A, 3P7B, 3P7C, 3P7F, 3P7G, 3P7H, 3P7I, 3P7J, 3P7K, 3P7L, 3P7M, 3P7N, 3P7O, 3P7P, 3P7Q, 3P7R, 3P7S, 3P7T, 3P7U, 3P7V, 3P7W, 3P7X, 3P7Y, 3P7Z, 3P80, 3P81, 3P82, 3P83, 3P84, 3P85, 3P86, 3P87, 3P88, 3P89, 3P8A, 3P8B, 3P8C, 3P8D, 3P8E, 3P8F, 3P8G, 3P8H, 3P8I, 3P8J, 3P8K, 3P8L, 3P8M, 3P8N, 3P8O, 3P8P, 3P8R, 3P8S, 3P8T, 3P8U, 3P8V, 3P8W, 3P8X, 3P8Y, 3P8Z, 3P90, 3P91, 3P92, 3P93, 3P94, 3P95, 3P96, 3P97, 3P98, 3P99, 3P9A, 3P9C, 3P9D, 3P9E, 3P9F, 3P9G, 3P9H, 3P9I, 3P9J, 3P9K, 3P9L, 3P9M, 3P9N, 3P9O, 3P9P, 3P9Q, 3P9R, 3P9S, 3P9T, 3P9U, 3P9V, 3P9W, 3P9X, 3P9Y, 3P9Z, 3PA1, 3PA2, 3PA3, 3PA4, 3PA5, 3PA6, 3PA7, 3PA8, 3PA9, 3PAA, 3PAB, 3PAC, 3PAE, 3PAF, 3PAG, 3PAH, 3PAJ, 3PAK, 3PAL, 3PAM, 3PAN, 3PAO, 3PAQ, 3PAR, 3PAS, 3PAU, 3PAV, 3PAW, 3PAX, 3PAY, 3PAZ, 3PB0, 3PB1, 3PB2, 3PB3, 3PB4, 3PB5, 3PB6, 3PB7, 3PB8, 3PB9, 3PBA, 3PBB, 3PBC, 3PBD, 3PBE, 3PBF, 3PBG, 3PBH, 3PBI, 3PBJ, 3PBK, 3PBL, 3PBM, 3PBN, 3PBO, 3PBP, 3PBQ, 3PBR, 3PBS, 3PBT, 3PBU, 3PBV, 3PBW, 3PBX, 3PBY, 3PBZ, 3PC0, 3PC2, 3PC3, 3PC4, 3PC6, 3PC7, 3PC8, 3PCA, 3PCB, 3PCC, 3PCD, 3PCE, 3PCF, 3PCG, 3PCH, 3PCI, 3PCJ, 3PCK, 3PCL, 3PCM, 3PCN, 3PCO, 3PCR, 3PCS, 3PCT, 3PCU, 3PCV, 3PCW, 3PCX, 3PCY, 3PCZ, 3PD0, 3PD1, 3PD2, 3PD3, 3PD4, 3PD5, 3PD6, 3PD7, 3PD8, 3PD9, 3PDB, 3PDC, 3PDD, 3PDE, 3PDF, 3PDG, 3PDH, 3PDI, 3PDJ, 3PDK, 3PDN, 3PDO, 3PDQ, 3PDS, 3PDT, 3PDU, 3PDV, 3PDW, 3PDX, 3PDY, 3PE0, 3PE1, 3PE2, 3PE3, 3PE4, 3PE5, 3PE6, 3PE7, 3PE8, 3PE9, 3PEA, 3PEB, 3PEC, 3PED, 3PEE, 3PEF, 3PEG, 3PEH, 3PEI, 3PEJ, 3PEL, 3PEN, 3PEO, 3PEP, 3PEQ, 3PER, 3PES, 3PET, 3PEU, 3PEV, 3PF0, 3PF1, 3PF2, 3PF3, 3PF4, 3PF5, 3PF6, 3PF7, 3PF8, 3PF9, 3PFB, 3PFC, 3PFD, 3PFE, 3PFF, 3PFG, 3PFH, 3PFI, 3PFJ, 3PFK, 3PFL, 3PFM, 3PFN, 3PFO, 3PFP, 3PFQ, 3PFR, 3PFS, 3PFT, 3PFU, 3PFV, 3PFW, 3PFX, 3PFY, 3PFZ, 3PG0, 3PG1, 3PG2, 3PG3, 3PG4, 3PG5, 3PG6, 3PG7, 3PG8, 3PG9, 3PGA, 3PGB, 3PGC, 3PGD, 3PGE, 3PGF, 3PGG, 3PGH, 3PGI, 3PGJ, 3PGK, 3PGL, 3PGM, 3PGP, 3PGQ, 3PGR, 3PGS, 3PGT, 3PGU, 3PGV, 3PGW, 3PGX, 3PGY, 3PGZ, 3PH0, 3PH1, 3PH2, 3PH3, 3PH4, 3PH5, 3PH6, 3PH7, 3PH9, 3PHA, 3PHB, 3PHC, 3PHD, 3PHE, 3PHF, 3PHG, 3PHH, 3PHI, 3PHJ, 3PHL, 3PHM, 3PHN, 3PHO, 3PHQ, 3PHS, 3PHT, 3PHU, 3PHV, 3PHW, 3PHX, 3PHZ, 3PI0, 3PI1, 3PI2, 3PI3, 3PI4, 3PI5, 3PI6, 3PI7, 3PI8, 3PI9, 3PIA, 3PIB, 3PIC, 3PID, 3PIE, 3PIF, 3PIG, 3PIH, 3PII, 3PIJ, 3PIK, 3PIL, 3PIM, 3PIN, 3PIQ, 3PIR, 3PIS, 3PIT, 3PIU, 3PIV, 3PIW, 3PIX, 3PIY, 3PIZ, 3PJ0, 3PJ1, 3PJ2, 3PJ3, 3PJ5, 3PJ6, 3PJ7, 3PJ8, 3PJ9, 3PJA, 3PJB, 3PJC, 3PJD, 3PJE, 3PJF, 3PJG, 3PJI, 3PJJ, 3PJK, 3PJL, 3PJN, 3PJP, 3PJQ, 3PJR, 3PJS, 3PJT, 3PJU, 3PJV, 3PJW, 3PJX, 3PJY, 3PJZ, 3PK0, 3PK1, 3PK2, 3PK3, 3PK4, 3PK5, 3PK6, 3PK7, 3PK8, 3PKA, 3PKB, 3PKC, 3PKD, 3PKE, 3PKF, 3PKG, 3PKH, 3PKI, 3PKJ, 3PKK, 3PKL, 3PKN, 3PKO, 3PKP, 3PKQ, 3PKS, 3PKT, 3PKU, 3PKV, 3PKW, 3PKX, 3PKY, 3PKZ, 3PL0, 3PL1, 3PL2, 3PL3, 3PL5, 3PL6, 3PL7, 3PL8, 3PLB, 3PLC, 3PLD, 3PLE, 3PLF, 3PLG, 3PLH, 3PLI, 3PLJ, 3PLK, 3PLL, 3PLM, 3PLN, 3PLP, 3PLQ, 3PLR, 3PLS, 3PLT, 3PLU, 3PLV, 3PLW, 3PLX, 3PLY, 3PLZ, 3PM0, 3PM1, 3PM2, 3PM3, 3PM4, 3PM5, 3PM6, 3PM7, 3PM8, 3PM9, 3PMA, 3PMB, 3PMC, 3PMD, 3PME, 3PMF, 3PMG, 3PMH, 3PMI, 3PMJ, 3PMK, 3PML, 3PMM, 3PMN, 3PMO, 3PMP, 3PMQ, 3PMR, 3PMS, 3PMT, 3PMU, 3PMV, 3PMW, 3PMX, 3PMY, 3PMZ, 3PN1, 3PN2, 3PN3, 3PN4, 3PN5, 3PN6, 3PN7, 3PN8, 3PN9, 3PNA, 3PNB, 3PNC, 3PND, 3PNE, 3PNF, 3PNG, 3PNH, 3PNI, 3PNK, 3PNL, 3PNM, 3PNN, 3PNO, 3PNP, 3PNQ, 3PNR, 3PNS, 3PNT, 3PNU, 3PNV, 3PNW, 3PNX, 3PNY, 3PNZ, 3PO0, 3PO1, 3PO4, 3PO5, 3PO6, 3PO7, 3PO8, 3PO9, 3POA, 3POB, 3POC, 3POE, 3POF, 3POG, 3POH, 3POI, 3POJ, 3POK, 3POL, 3POM, 3POO, 3POP, 3POQ, 3POR, 3POS, 3POT, 3POU, 3POV, 3POW, 3POX, 3POY, 3POZ, 3PP0, 3PP1, 3PP2, 3PP3, 3PP4, 3PP5, 3PP6, 3PP7, 3PP8, 3PP9, 3PPA, 3PPB, 3PPC, 3PPD, 3PPE, 3PPF, 3PPG, 3PPH, 3PPI, 3PPJ, 3PPK, 3PPL, 3PPM, 3PPN, 3PPO, 3PPP, 3PPQ, 3PPR, 3PPS, 3PPT, 3PPU, 3PPV, 3PPW, 3PPX, 3PPY, 3PPZ, 3PQ1, 3PQ2, 3PQ3, 3PQ4, 3PQ5, 3PQ6, 3PQ7, 3PQ8, 3PQA, 3PQB, 3PQC, 3PQD, 3PQE, 3PQF, 3PQH, 3PQI, 3PQJ, 3PQK, 3PQR, 3PQS, 3PQU, 3PQV, 3PQY, 3PQZ, 3PR0, 3PR1, 3PR2, 3PR3, 3PR4, 3PR5, 3PR6, 3PR7, 3PR8, 3PR9, 3PRA, 3PRB, 3PRC, 3PRD, 3PRE, 3PRF, 3PRG, 3PRH, 3PRI, 3PRJ, 3PRK, 3PRL, 3PRM, 3PRN, 3PRO, 3PRP, 3PRQ, 3PRR, 3PRS, 3PRT, 3PRU, 3PRV, 3PRW, 3PRX, 3PRY, 3PRZ, 3PS0, 3PS1, 3PS2, 3PS3, 3PS4, 3PS5, 3PS6, 3PS7, 3PS8, 3PS9, 3PSA, 3PSB, 3PSC, 3PSD, 3PSE, 3PSF, 3PSG, 3PSH, 3PSI, 3PSJ, 3PSK, 3PSL, 3PSM, 3PSN, 3PSO, 3PSP, 3PSQ, 3PSR, 3PSS, 3PST, 3PSU, 3PSV, 3PSW, 3PSX, 3PSY, 3PSZ, 3PT1, 3PT2, 3PT3, 3PT5, 3PT6, 3PT7, 3PT8, 3PT9, 3PTA, 3PTB, 3PTD, 3PTE, 3PTF, 3PTG, 3PTH, 3PTJ, 3PTK, 3PTL, 3PTM, 3PTN, 3PTO, 3PTQ, 3PTR, 3PTW, 3PTY, 3PTZ, 3PU2, 3PU3, 3PU5, 3PU6, 3PU7, 3PU8, 3PU9, 3PUA, 3PUB, 3PUC, 3PUD, 3PUE, 3PUF, 3PUG, 3PUH, 3PUI, 3PUJ, 3PUK, 3PUL, 3PUM, 3PUN, 3PUO, 3PUP, 3PUQ, 3PUR, 3PUS, 3PUT, 3PUU, 3PUV, 3PUW, 3PUX, 3PUY, 3PUZ, 3PV0, 3PV1, 3PV2, 3PV3, 3PV4, 3PV5, 3PV6, 3PV7, 3PV8, 3PV9, 3PVA, 3PVB, 3PVC, 3PVD, 3PVE, 3PVF, 3PVG, 3PVH, 3PVI, 3PVJ, 3PVK, 3PVL, 3PVM, 3PVN, 3PVO, 3PVP, 3PVQ, 3PVR, 3PVS, 3PVT, 3PVU, 3PVV, 3PVW, 3PVX, 3PVY, 3PVZ, 3PW0, 3PW1, 3PW2, 3PW3, 3PW4, 3PW5, 3PW7, 3PW8, 3PW9, 3PWA, 3PWB, 3PWC, 3PWD, 3PWE, 3PWF, 3PWG, 3PWH, 3PWI, 3PWJ, 3PWK, 3PWL, 3PWM, 3PWN, 3PWP, 3PWQ, 3PWR, 3PWS, 3PWT, 3PWU, 3PWV, 3PWW, 3PWX, 3PWY, 3PWZ, 3PX0, 3PX1, 3PX2, 3PX3, 3PX4, 3PX6, 3PX7, 3PX8, 3PX9, 3PXA, 3PXB, 3PXC, 3PXD, 3PXE, 3PXF, 3PXG, 3PXH, 3PXI, 3PXJ, 3PXK, 3PXL, 3PXM, 3PXN, 3PXO, 3PXP, 3PXQ, 3PXR, 3PXS, 3PXT, 3PXU, 3PXV, 3PXW, 3PXX, 3PXY, 3PXZ, 3PY0, 3PY1, 3PY2, 3PY3, 3PY4, 3PY5, 3PY6, 3PY7, 3PY8, 3PY9, 3PYA, 3PYB, 3PYC, 3PYD, 3PYE, 3PYF, 3PYG, 3PYH, 3PYI, 3PYJ, 3PYK, 3PYL, 3PYM, 3PYP, 3PYW, 3PYX, 3PYY, 3PYZ, 3PZ0, 3PZ1, 3PZ2, 3PZ3, 3PZ4, 3PZ5, 3PZ6, 3PZ7, 3PZ8, 3PZ9, 3PZA, 3PZB, 3PZC, 3PZD, 3PZE, 3PZF, 3PZG, 3PZH, 3PZI, 3PZJ, 3PZK, 3PZL, 3PZM, 3PZN, 3PZO, 3PZP, 3PZQ, 3PZR, 3PZS, 3PZT, 3PZU, 3PZV, 3PZW, 3PZY, 3PZZ, 3Q00, 3Q01, 3Q02, 3Q03, 3Q04, 3Q05, 3Q06, 3Q07, 3Q08, 3Q09, 3Q0A, 3Q0B, 3Q0C, 3Q0D, 3Q0E, 3Q0F, 3Q0G, 3Q0H, 3Q0I, 3Q0J, 3Q0K, 3Q0T, 3Q0U, 3Q0V, 3Q0W, 3Q0X, 3Q0Y, 3Q0Z, 3Q10, 3Q11, 3Q12, 3Q13, 3Q14, 3Q15, 3Q16, 3Q17, 3Q18, 3Q19, 3Q1C, 3Q1D, 3Q1E, 3Q1F, 3Q1G, 3Q1H, 3Q1I, 3Q1J, 3Q1K, 3Q1L, 3Q1M, 3Q1N, 3Q1O, 3Q1P, 3Q1S, 3Q1T, 3Q1X, 3Q1Y, 3Q20, 3Q22, 3Q23, 3Q24, 3Q25, 3Q26, 3Q27, 3Q28, 3Q29, 3Q2A, 3Q2B, 3Q2C, 3Q2D, 3Q2E, 3Q2F, 3Q2G, 3Q2H, 3Q2I, 3Q2J, 3Q2K, 3Q2L, 3Q2M, 3Q2N, 3Q2O, 3Q2P, 3Q2Q, 3Q2R, 3Q2S, 3Q2U, 3Q2V, 3Q2W, 3Q2X, 3Q2Y, 3Q2Z, 3Q30, 3Q31, 3Q32, 3Q33, 3Q34, 3Q35, 3Q36, 3Q37, 3Q38, 3Q39, 3Q3A, 3Q3B, 3Q3C, 3Q3D, 3Q3E, 3Q3F, 3Q3G, 3Q3H, 3Q3I, 3Q3J, 3Q3K, 3Q3M, 3Q3N, 3Q3O, 3Q3Q, 3Q3S, 3Q3T, 3Q3U, 3Q3V, 3Q3W, 3Q3X, 3Q3Y, 3Q40, 3Q41, 3Q43, 3Q44, 3Q45, 3Q46, 3Q47, 3Q48, 3Q49, 3Q4A, 3Q4B, 3Q4C, 3Q4D, 3Q4F, 3Q4G, 3Q4H, 3Q4I, 3Q4J, 3Q4K, 3Q4L, 3Q4N, 3Q4O, 3Q4P, 3Q4Q, 3Q4R, 3Q4S, 3Q4T, 3Q4U, 3Q4W, 3Q4Y, 3Q4Z, 3Q52, 3Q53, 3Q54, 3Q58, 3Q5C, 3Q5D, 3Q5E, 3Q5F, 3Q5G, 3Q5H, 3Q5I, 3Q5J, 3Q5K, 3Q5L, 3Q5M, 3Q5O, 3Q5P, 3Q5R, 3Q5S, 3Q5T, 3Q5U, 3Q5V, 3Q5W, 3Q5X, 3Q5Y, 3Q5Z, 3Q60, 3Q61, 3Q62, 3Q63, 3Q64, 3Q65, 3Q66, 3Q67, 3Q68, 3Q69, 3Q6A, 3Q6B, 3Q6C, 3Q6D, 3Q6E, 3Q6F, 3Q6G, 3Q6I, 3Q6J, 3Q6K, 3Q6L, 3Q6M, 3Q6N, 3Q6O, 3Q6P, 3Q6Q, 3Q6R, 3Q6S, 3Q6T, 3Q6U, 3Q6V, 3Q6W, 3Q6X, 3Q6Y, 3Q6Z, 3Q70, 3Q71, 3Q72, 3Q73, 3Q74, 3Q75, 3Q76, 3Q77, 3Q78, 3Q79, 3Q7A, 3Q7B, 3Q7C, 3Q7D, 3Q7E, 3Q7F, 3Q7G, 3Q7H, 3Q7I, 3Q7J, 3Q7K, 3Q7L, 3Q7M, 3Q7N, 3Q7O, 3Q7P, 3Q7Q, 3Q7R, 3Q7S, 3Q7T, 3Q7U, 3Q7V, 3Q7W, 3Q7X, 3Q7Y, 3Q7Z, 3Q80, 3Q81, 3Q82, 3Q83, 3Q84, 3Q85, 3Q86, 3Q87, 3Q88, 3Q89, 3Q8A, 3Q8B, 3Q8C, 3Q8D, 3Q8E, 3Q8F, 3Q8G, 3Q8H, 3Q8I, 3Q8J, 3Q8K, 3Q8L, 3Q8M, 3Q8N, 3Q8P, 3Q8Q, 3Q8R, 3Q8S, 3Q8T, 3Q8U, 3Q8V, 3Q8W, 3Q8X, 3Q8Y, 3Q90, 3Q91, 3Q92, 3Q93, 3Q94, 3Q95, 3Q96, 3Q97, 3Q98, 3Q99, 3Q9A, 3Q9B, 3Q9C, 3Q9D, 3Q9E, 3Q9F, 3Q9K, 3Q9L, 3Q9M, 3Q9N, 3Q9O, 3Q9P, 3Q9Q, 3Q9S, 3Q9T, 3Q9U, 3Q9V, 3Q9W, 3Q9X, 3Q9Y, 3Q9Z, 3QA0, 3QA2, 3QA3, 3QA8, 3QA9, 3QAA, 3QAC, 3QAE, 3QAG, 3QAH, 3QAI, 3QAK, 3QAL, 3QAM, 3QAN, 3QAO, 3QAP, 3QAQ, 3QAR, 3QAS, 3QAT, 3QAU, 3QAV, 3QAW, 3QAX, 3QAY, 3QAZ, 3QB0, 3QB1, 3QB2, 3QB3, 3QB4, 3QB5, 3QB7, 3QB8, 3QB9, 3QBA, 3QBC, 3QBD, 3QBE, 3QBF, 3QBG, 3QBH, 3QBI, 3QBJ, 3QBK, 3QBL, 3QBM, 3QBN, 3QBO, 3QBP, 3QBQ, 3QBR, 3QBT, 3QBU, 3QBV, 3QBW, 3QBX, 3QBY, 3QBZ, 3QC0, 3QC1, 3QC2, 3QC3, 3QC4, 3QC5, 3QC6, 3QC7, 3QC8, 3QC9, 3QCA, 3QCB, 3QCC, 3QCD, 3QCE, 3QCF, 3QCG, 3QCH, 3QCI, 3QCJ, 3QCK, 3QCL, 3QCM, 3QCN, 3QCP, 3QCQ, 3QCS, 3QCT, 3QCU, 3QCV, 3QCW, 3QCX, 3QCY, 3QCZ, 3QD0, 3QD2, 3QD3, 3QD4, 3QD5, 3QD6, 3QD7, 3QD8, 3QD9, 3QDA, 3QDC, 3QDD, 3QDE, 3QDF, 3QDG, 3QDH, 3QDJ, 3QDK, 3QDL, 3QDM, 3QDN, 3QDO, 3QDP, 3QDQ, 3QDR, 3QDS, 3QDT, 3QDU, 3QDV, 3QDW, 3QDX, 3QDY, 3QDZ, 3QE0, 3QE1, 3QE2, 3QE3, 3QE4, 3QE5, 3QE6, 3QE7, 3QE8, 3QE9, 3QEA, 3QEB, 3QEC, 3QED, 3QEE, 3QEF, 3QEG, 3QEH, 3QEI, 3QEJ, 3QEK, 3QEL, 3QEM, 3QEN, 3QEO, 3QEP, 3QEQ, 3QER, 3QES, 3QET, 3QEU, 3QEV, 3QEW, 3QEX, 3QEZ, 3QF0, 3QF1, 3QF2, 3QF3, 3QF4, 3QF7, 3QF9, 3QFA, 3QFB, 3QFC, 3QFD, 3QFE, 3QFF, 3QFG, 3QFH, 3QFI, 3QFJ, 3QFK, 3QFL, 3QFM, 3QFN, 3QFO, 3QFP, 3QFQ, 3QFR, 3QFS, 3QFT, 3QFU, 3QFV, 3QFW, 3QFX, 3QFY, 3QFZ, 3QG0, 3QG1, 3QG2, 3QG5, 3QG6, 3QG7, 3QGA, 3QGD, 3QGE, 3QGF, 3QGG, 3QGH, 3QGI, 3QGJ, 3QGK, 3QGL, 3QGM, 3QGN, 3QGO, 3QGP, 3QGT, 3QGU, 3QGV, 3QGW, 3QGY, 3QGZ, 3QH0, 3QH1, 3QH2, 3QH3, 3QH4, 3QH5, 3QH6, 3QH7, 3QH8, 3QH9, 3QHA, 3QHB, 3QHC, 3QHD, 3QHE, 3QHF, 3QHM, 3QHN, 3QHO, 3QHP, 3QHQ, 3QHR, 3QHS, 3QHT, 3QHW, 3QHX, 3QHY, 3QHZ, 3QI0, 3QI1, 3QI2, 3QI3, 3QI4, 3QI5, 3QI6, 3QI7, 3QI8, 3QI9, 3QIA, 3QIB, 3QIC, 3QID, 3QIH, 3QII, 3QIJ, 3QIK, 3QIL, 3QIM, 3QIN, 3QIO, 3QIP, 3QIR, 3QIS, 3QIT, 3QIU, 3QIV, 3QIW, 3QIX, 3QIY, 3QIZ, 3QJ0, 3QJ1, 3QJ3, 3QJ4, 3QJ5, 3QJ6, 3QJ7, 3QJ8, 3QJ9, 3QJA, 3QJB, 3QJC, 3QJD, 3QJE, 3QJF, 3QJG, 3QJH, 3QJI, 3QJK, 3QJM, 3QJN, 3QJO, 3QJQ, 3QJR, 3QJS, 3QJT, 3QJU, 3QJV, 3QJX, 3QJY, 3QJZ, 3QK0, 3QK1, 3QK2, 3QK3, 3QK4, 3QK5, 3QK6, 3QK7, 3QK8, 3QK9, 3QKA, 3QKB, 3QKC, 3QKD, 3QKE, 3QKG, 3QKI, 3QKJ, 3QKK, 3QKL, 3QKM, 3QKP, 3QKQ, 3QKR, 3QKS, 3QKT, 3QKU, 3QKV, 3QKW, 3QKX, 3QKY, 3QKZ, 3QL0, 3QL1, 3QL2, 3QL3, 3QL6, 3QL8, 3QL9, 3QLA, 3QLB, 3QLC, 3QLD, 3QLE, 3QLF, 3QLG, 3QLH, 3QLI, 3QLJ, 3QLK, 3QLL, 3QLM, 3QLN, 3QLP, 3QLQ, 3QLR, 3QLS, 3QLT, 3QLU, 3QLV, 3QLW, 3QLX, 3QLY, 3QLZ, 3QM0, 3QM1, 3QM2, 3QM3, 3QM4, 3QM5, 3QM6, 3QM7, 3QM8, 3QM9, 3QMA, 3QMB, 3QMC, 3QMD, 3QME, 3QMF, 3QMG, 3QMH, 3QMI, 3QMJ, 3QMK, 3QML, 3QMM, 3QMN, 3QMO, 3QMP, 3QMQ, 3QMR, 3QMS, 3QMT, 3QMU, 3QMV, 3QMW, 3QMX, 3QMZ, 3QN0, 3QN1, 3QN2, 3QN3, 3QN6, 3QN7, 3QN8, 3QN9, 3QNA, 3QNB, 3QNC, 3QND, 3QNE, 3QNF, 3QNG, 3QNI, 3QNJ, 3QNK, 3QNL, 3QNM, 3QNN, 3QNO, 3QNQ, 3QNR, 3QNS, 3QNT, 3QNU, 3QNV, 3QNW, 3QNX, 3QNY, 3QNZ, 3QO0, 3QO1, 3QO2, 3QO3, 3QO4, 3QO5, 3QO6, 3QO7, 3QO8, 3QO9, 3QOA, 3QOC, 3QOD, 3QOE, 3QOF, 3QOJ, 3QOK, 3QOL, 3QOM, 3QON, 3QOO, 3QOP, 3QOQ, 3QOR, 3QOS, 3QOT, 3QOU, 3QOV, 3QOW, 3QOX, 3QOY, 3QP0, 3QP1, 3QP2, 3QP3, 3QP4, 3QP5, 3QP6, 3QP8, 3QP9, 3QPA, 3QPB, 3QPC, 3QPD, 3QPE, 3QPF, 3QPG, 3QPH, 3QPI, 3QPJ, 3QPK, 3QPL, 3QPM, 3QPN, 3QPO, 3QPP, 3QPQ, 3QPS, 3QPT, 3QPU, 3QPV, 3QPW, 3QPX, 3QPY, 3QPZ, 3QQ0, 3QQ1, 3QQ2, 3QQ3, 3QQ4, 3QQ5, 3QQ6, 3QQ7, 3QQ8, 3QQ9, 3QQA, 3QQB, 3QQC, 3QQD, 3QQE, 3QQF, 3QQG, 3QQH, 3QQI, 3QQJ, 3QQK, 3QQL, 3QQM, 3QQN, 3QQO, 3QQP, 3QQQ, 3QQR, 3QQS, 3QQT, 3QQU, 3QQV, 3QQW, 3QQX, 3QQY, 3QQZ, 3QR0, 3QR1, 3QR2, 3QR3, 3QR5, 3QR6, 3QR7, 3QR8, 3QR9, 3QRA, 3QRB, 3QRC, 3QRD, 3QRE, 3QRF, 3QRG, 3QRH, 3QRI, 3QRJ, 3QRK, 3QRL, 3QRM, 3QRO, 3QRS, 3QRT, 3QRU, 3QRV, 3QRW, 3QRX, 3QRY, 3QS0, 3QS1, 3QS2, 3QS3, 3QS4, 3QS5, 3QS6, 3QS7, 3QS8, 3QS9, 3QSA, 3QSB, 3QSD, 3QSE, 3QSF, 3QSG, 3QSI, 3QSJ, 3QSK, 3QSL, 3QSM, 3QSP, 3QSQ, 3QSR, 3QSS, 3QST, 3QSV, 3QSZ, 3QT0, 3QT1, 3QT2, 3QT3, 3QT4, 3QT5, 3QT6, 3QT7, 3QT8, 3QT9, 3QTA, 3QTB, 3QTC, 3QTD, 3QTE, 3QTF, 3QTG, 3QTH, 3QTI, 3QTK, 3QTL, 3QTM, 3QTN, 3QTO, 3QTP, 3QTQ, 3QTR, 3QTS, 3QTT, 3QTU, 3QTV, 3QTW, 3QTX, 3QTY, 3QTZ, 3QU0, 3QU1, 3QU2, 3QU3, 3QU4, 3QU5, 3QU6, 3QU7, 3QU8, 3QU9, 3QUA, 3QUB, 3QUC, 3QUD, 3QUE, 3QUF, 3QUG, 3QUH, 3QUI, 3QUJ, 3QUK, 3QUL, 3QUM, 3QUN, 3QUO, 3QUP, 3QUQ, 3QUR, 3QUS, 3QUT, 3QUV, 3QUW, 3QUX, 3QUY, 3QUZ, 3QV0, 3QV1, 3QV2, 3QV4, 3QV6, 3QV7, 3QV8, 3QV9, 3QVA, 3QVB, 3QVC, 3QVD, 3QVE, 3QVF, 3QVG, 3QVH, 3QVI, 3QVJ, 3QVK, 3QVL, 3QVM, 3QVN, 3QVO, 3QVP, 3QVQ, 3QVR, 3QVS, 3QVT, 3QVU, 3QVV, 3QVW, 3QVX, 3QVY, 3QVZ, 3QW0, 3QW1, 3QW2, 3QW3, 3QW4, 3QW5, 3QW6, 3QW7, 3QW8, 3QW9, 3QWA, 3QWB, 3QWC, 3QWD, 3QWE, 3QWF, 3QWG, 3QWH, 3QWI, 3QWJ, 3QWK, 3QWL, 3QWM, 3QWN, 3QWO, 3QWP, 3QWQ, 3QWR, 3QWS, 3QWT, 3QWU, 3QWV, 3QWW, 3QWX, 3QWY, 3QWZ, 3QX1, 3QX2, 3QX3, 3QX4, 3QX5, 3QX7, 3QX8, 3QX9, 3QXA, 3QXB, 3QXC, 3QXD, 3QXE, 3QXF, 3QXG, 3QXH, 3QXI, 3QXJ, 3QXL, 3QXM, 3QXO, 3QXP, 3QXQ, 3QXR, 3QXS, 3QXT, 3QXU, 3QXV, 3QXW, 3QXX, 3QXY, 3QXZ, 3QY0, 3QY1, 3QY2, 3QY3, 3QY4, 3QY5, 3QY6, 3QY7, 3QY8, 3QY9, 3QYA, 3QYB, 3QYC, 3QYD, 3QYE, 3QYF, 3QYG, 3QYH, 3QYI, 3QYJ, 3QYK, 3QYL, 3QYM, 3QYN, 3QYO, 3QYP, 3QYQ, 3QYR, 3QYS, 3QYT, 3QYU, 3QYW, 3QYX, 3QYY, 3QYZ, 3QZ0, 3QZ1, 3QZ2, 3QZ3, 3QZ4, 3QZ5, 3QZ6, 3QZ7, 3QZ8, 3QZ9, 3QZA, 3QZB, 3QZC, 3QZE, 3QZF, 3QZG, 3QZH, 3QZI, 3QZL, 3QZM, 3QZN, 3QZO, 3QZP, 3QZQ, 3QZR, 3QZS, 3QZT, 3QZU, 3QZV, 3QZW, 3QZX, 3QZY, 3QZZ, 3R00, 3R01, 3R02, 3R03, 3R04, 3R05, 3R06, 3R07, 3R08, 3R09, 3R0A, 3R0D, 3R0E, 3R0F, 3R0G, 3R0H, 3R0I, 3R0J, 3R0K, 3R0L, 3R0M, 3R0N, 3R0O, 3R0P, 3R0Q, 3R0R, 3R0S, 3R0T, 3R0U, 3R0V, 3R0W, 3R0X, 3R0Y, 3R0Z, 3R10, 3R11, 3R12, 3R13, 3R15, 3R16, 3R17, 3R18, 3R19, 3R1A, 3R1B, 3R1F, 3R1G, 3R1I, 3R1J, 3R1K, 3R1M, 3R1N, 3R1O, 3R1P, 3R1Q, 3R1R, 3R1S, 3R1V, 3R1W, 3R1X, 3R1Y, 3R1Z, 3R20, 3R21, 3R22, 3R23, 3R24, 3R25, 3R26, 3R27, 3R28, 3R29, 3R2A, 3R2B, 3R2E, 3R2F, 3R2G, 3R2H, 3R2I, 3R2J, 3R2K, 3R2L, 3R2M, 3R2N, 3R2O, 3R2P, 3R2Q, 3R2R, 3R2S, 3R2T, 3R2U, 3R2V, 3R2W, 3R2X, 3R2Y, 3R30, 3R31, 3R32, 3R33, 3R34, 3R35, 3R36, 3R37, 3R38, 3R3A, 3R3B, 3R3C, 3R3D, 3R3E, 3R3F, 3R3G, 3R3H, 3R3I, 3R3J, 3R3K, 3R3L, 3R3M, 3R3O, 3R3P, 3R3Q, 3R3R, 3R3S, 3R3T, 3R3U, 3R3V, 3R3W, 3R3X, 3R3Y, 3R3Z, 3R40, 3R41, 3R42, 3R43, 3R44, 3R45, 3R46, 3R47, 3R48, 3R49, 3R4A, 3R4B, 3R4C, 3R4D, 3R4G, 3R4H, 3R4I, 3R4K, 3R4L, 3R4M, 3R4N, 3R4O, 3R4P, 3R4Q, 3R4R, 3R4S, 3R4T, 3R4U, 3R4V, 3R4X, 3R4Y, 3R4Z, 3R50, 3R51, 3R52, 3R54, 3R55, 3R56, 3R57, 3R58, 3R59, 3R5A, 3R5B, 3R5C, 3R5D, 3R5E, 3R5F, 3R5G, 3R5H, 3R5I, 3R5J, 3R5K, 3R5L, 3R5M, 3R5N, 3R5O, 3R5P, 3R5Q, 3R5R, 3R5S, 3R5T, 3R5U, 3R5V, 3R5W, 3R5X, 3R5Y, 3R5Z, 3R60, 3R61, 3R62, 3R63, 3R64, 3R65, 3R66, 3R67, 3R68, 3R69, 3R6A, 3R6B, 3R6C, 3R6D, 3R6E, 3R6F, 3R6G, 3R6H, 3R6I, 3R6J, 3R6K, 3R6L, 3R6M, 3R6N, 3R6O, 3R6P, 3R6Q, 3R6R, 3R6S, 3R6T, 3R6U, 3R6V, 3R6W, 3R6X, 3R6Y, 3R71, 3R72, 3R73, 3R74, 3R75, 3R76, 3R77, 3R79, 3R7A, 3R7B, 3R7C, 3R7D, 3R7E, 3R7F, 3R7G, 3R7I, 3R7K, 3R7L, 3R7M, 3R7N, 3R7O, 3R7P, 3R7Q, 3R7R, 3R7S, 3R7T, 3R7U, 3R7V, 3R7W, 3R7X, 3R7Y, 3R83, 3R84, 3R85, 3R86, 3R87, 3R88, 3R89, 3R8A, 3R8B, 3R8C, 3R8D, 3R8E, 3R8F, 3R8G, 3R8H, 3R8I, 3R8J, 3R8K, 3R8L, 3R8M, 3R8P, 3R8Q, 3R8R, 3R8U, 3R8V, 3R8W, 3R8X, 3R8Y, 3R8Z, 3R90, 3R91, 3R92, 3R93, 3R94, 3R95, 3R96, 3R97, 3R98, 3R99, 3R9A, 3R9B, 3R9C, 3R9D, 3R9E, 3R9F, 3R9G, 3R9H, 3R9I, 3R9J, 3R9K, 3R9L, 3R9M, 3R9N, 3R9O, 3R9P, 3R9Q, 3R9R, 3R9S, 3R9T, 3R9U, 3R9V, 3R9Y, 3R9Z, 3RA0, 3RA3, 3RA5, 3RA6, 3RA7, 3RAB, 3RAC, 3RAD, 3RAE, 3RAF, 3RAG, 3RAH, 3RAI, 3RAJ, 3RAK, 3RAL, 3RAM, 3RAN, 3RAO, 3RAP, 3RAQ, 3RAR, 3RAS, 3RAT, 3RAU, 3RAV, 3RAW, 3RAX, 3RAY, 3RAZ, 3RB0, 3RB3, 3RB4, 3RB5, 3RB6, 3RB7, 3RB8, 3RB9, 3RBA, 3RBB, 3RBC, 3RBD, 3RBE, 3RBF, 3RBG, 3RBH, 3RBI, 3RBJ, 3RBK, 3RBL, 3RBM, 3RBN, 3RBQ, 3RBS, 3RBT, 3RBU, 3RBV, 3RBW, 3RBX, 3RBY, 3RBZ, 3RC0, 3RC1, 3RC2, 3RC3, 3RC4, 3RC5, 3RC6, 3RC7, 3RC9, 3RCB, 3RCC, 3RCD, 3RCE, 3RCF, 3RCG, 3RCH, 3RCI, 3RCJ, 3RCK, 3RCL, 3RCM, 3RCN, 3RCO, 3RCP, 3RCQ, 3RCW, 3RCY, 3RCZ, 3RD0, 3RD1, 3RD2, 3RD3, 3RD4, 3RD5, 3RD6, 3RD7, 3RD8, 3RD9, 3RDA, 3RDB, 3RDC, 3RDD, 3RDE, 3RDH, 3RDI, 3RDJ, 3RDK, 3RDM, 3RDO, 3RDP, 3RDQ, 3RDR, 3RDS, 3RDT, 3RDU, 3RDV, 3RDW, 3RDX, 3RDY, 3RDZ, 3RE0, 3RE1, 3RE2, 3RE3, 3RE4, 3RE5, 3RE6, 3RE7, 3RE8, 3RE9, 3REA, 3REB, 3RED, 3REE, 3REF, 3REG, 3REH, 3REI, 3REJ, 3REK, 3REL, 3REM, 3REN, 3REO, 3REP, 3REQ, 3RES, 3RET, 3REU, 3REV, 3REW, 3REX, 3REY, 3REZ, 3RF0, 3RF1, 3RF2, 3RF3, 3RF4, 3RF5, 3RF6, 3RF7, 3RF9, 3RFA, 3RFB, 3RFC, 3RFE, 3RFF, 3RFG, 3RFH, 3RFI, 3RFJ, 3RFM, 3RFN, 3RFQ, 3RFR, 3RFS, 3RFT, 3RFU, 3RFV, 3RFW, 3RFX, 3RFY, 3RFZ, 3RG0, 3RG1, 3RG2, 3RG3, 3RG4, 3RG6, 3RG8, 3RG9, 3RGA, 3RGB, 3RGC, 3RGD, 3RGE, 3RGF, 3RGG, 3RGH, 3RGI, 3RGK, 3RGL, 3RGM, 3RGN, 3RGO, 3RGP, 3RGQ, 3RGR, 3RGS, 3RGT, 3RGU, 3RGV, 3RGW, 3RGX, 3RGY, 3RGZ, 3RH0, 3RH1, 3RH2, 3RH3, 3RH4, 3RH5, 3RH6, 3RH7, 3RH8, 3RH9, 3RHA, 3RHB, 3RHC, 3RHD, 3RHE, 3RHF, 3RHG, 3RHH, 3RHI, 3RHJ, 3RHK, 3RHL, 3RHM, 3RHN, 3RHO, 3RHP, 3RHQ, 3RHR, 3RHS, 3RHT, 3RHU, 3RHW, 3RHX, 3RHY, 3RHZ, 3RI0, 3RI1, 3RI3, 3RI4, 3RI5, 3RI6, 3RI7, 3RI8, 3RI9, 3RIA, 3RIB, 3RIC, 3RID, 3RIE, 3RIF, 3RIG, 3RIH, 3RII, 3RIJ, 3RIK, 3RIL, 3RIM, 3RIN, 3RIO, 3RIP, 3RIQ, 3RIR, 3RIS, 3RIT, 3RIU, 3RIV, 3RIW, 3RIX, 3RIY, 3RIZ, 3RJ0, 3RJ1, 3RJ2, 3RJ3, 3RJ4, 3RJ5, 3RJ6, 3RJ7, 3RJ8, 3RJ9, 3RJA, 3RJC, 3RJD, 3RJE, 3RJF, 3RJG, 3RJH, 3RJI, 3RJJ, 3RJK, 3RJL, 3RJM, 3RJN, 3RJO, 3RJP, 3RJQ, 3RJR, 3RJS, 3RJT, 3RJU, 3RJV, 3RJW, 3RJX, 3RJY, 3RJZ, 3RK0, 3RK1, 3RK2, 3RK3, 3RK4, 3RK5, 3RK6, 3RK7, 3RK8, 3RK9, 3RKB, 3RKC, 3RKD, 3RKE, 3RKG, 3RKH, 3RKI, 3RKJ, 3RKK, 3RKL, 3RKO, 3RKP, 3RKQ, 3RKR, 3RKS, 3RKT, 3RKU, 3RKV, 3RKW, 3RKX, 3RKY, 3RKZ, 3RL0, 3RL1, 3RL2, 3RL3, 3RL4, 3RL5, 3RL6, 3RL7, 3RL8, 3RL9, 3RLA, 3RLB, 3RLC, 3RLD, 3RLE, 3RLF, 3RLG, 3RLH, 3RLI, 3RLJ, 3RLK, 3RLL, 3RLM, 3RLN, 3RLO, 3RLP, 3RLQ, 3RLR, 3RLS, 3RLU, 3RLV, 3RLW, 3RLY, 3RLZ, 3RM0, 3RM1, 3RM2, 3RM3, 3RM4, 3RM5, 3RM6, 3RM7, 3RM8, 3RM9, 3RMA, 3RMB, 3RMC, 3RMD, 3RME, 3RMF, 3RMG, 3RMH, 3RMI, 3RMJ, 3RMK, 3RML, 3RMM, 3RMN, 3RMO, 3RMP, 3RMQ, 3RMR, 3RMS, 3RMT, 3RMU, 3RMV, 3RMW, 3RMX, 3RMY, 3RMZ, 3RN0, 3RN1, 3RN2, 3RN3, 3RN4, 3RN5, 3RN6, 3RN8, 3RN9, 3RNA, 3RNB, 3RNC, 3RND, 3RNE, 3RNF, 3RNG, 3RNI, 3RNJ, 3RNK, 3RNL, 3RNM, 3RNN, 3RNO, 3RNQ, 3RNR, 3RNS, 3RNT, 3RNU, 3RNV, 3RNX, 3RNY, 3RNZ, 3RO0, 3RO1, 3RO2, 3RO3, 3RO4, 3RO5, 3RO6, 3RO7, 3RO8, 3RO9, 3ROA, 3ROB, 3ROC, 3ROD, 3ROE, 3ROF, 3ROG, 3ROH, 3ROI, 3ROJ, 3ROK, 3ROL, 3ROM, 3RON, 3ROO, 3ROP, 3ROQ, 3ROR, 3ROS, 3ROT, 3ROU, 3ROV, 3ROW, 3ROX, 3ROY, 3ROZ, 3RP1, 3RP2, 3RP6, 3RP7, 3RP8, 3RP9, 3RPC, 3RPD, 3RPE, 3RPF, 3RPG, 3RPH, 3RPI, 3RPJ, 3RPK, 3RPL, 3RPM, 3RPN, 3RPO, 3RPP, 3RPQ, 3RPR, 3RPS, 3RPT, 3RPU, 3RPV, 3RPW, 3RPX, 3RPY, 3RPZ, 3RQ0, 3RQ1, 3RQ2, 3RQ3, 3RQ4, 3RQ5, 3RQ6, 3RQ7, 3RQ8, 3RQ9, 3RQA, 3RQB, 3RQC, 3RQD, 3RQE, 3RQF, 3RQG, 3RQH, 3RQI, 3RQJ, 3RQK, 3RQL, 3RQM, 3RQN, 3RQO, 3RQP, 3RQQ, 3RQR, 3RQS, 3RQT, 3RQU, 3RQV, 3RQW, 3RQX, 3RQZ, 3RR1, 3RR2, 3RR3, 3RR4, 3RR5, 3RR6, 3RR7, 3RR8, 3RRA, 3RRB, 3RRC, 3RRD, 3RRE, 3RRF, 3RRG, 3RRH, 3RRI, 3RRJ, 3RRK, 3RRL, 3RRM, 3RRN, 3RRO, 3RRP, 3RRQ, 3RRR, 3RRS, 3RRT, 3RRU, 3RRV, 3RRW, 3RRX, 3RRY, 3RRZ, 3RS0, 3RS1, 3RS2, 3RS3, 3RS4, 3RS5, 3RS6, 3RS7, 3RS8, 3RS9, 3RSB, 3RSC, 3RSD, 3RSE, 3RSF, 3RSG, 3RSH, 3RSI, 3RSJ, 3RSK, 3RSL, 3RSM, 3RSN, 3RSO, 3RSP, 3RSQ, 3RSR, 3RSS, 3RST, 3RSV, 3RSW, 3RSX, 3RSY, 3RSZ, 3RT0, 3RT1, 3RT2, 3RT3, 3RT4, 3RT5, 3RT6, 3RT7, 3RT8, 3RT9, 3RTA, 3RTB, 3RTC, 3RTD, 3RTE, 3RTF, 3RTG, 3RTH, 3RTI, 3RTK, 3RTL, 3RTM, 3RTN, 3RTO, 3RTP, 3RTQ, 3RTR, 3RTS, 3RTT, 3RTV, 3RTW, 3RTX, 3RTY, 3RU0, 3RU1, 3RU2, 3RU3, 3RU4, 3RU5, 3RU6, 3RU7, 3RU8, 3RU9, 3RUA, 3RUB, 3RUC, 3RUD, 3RUE, 3RUF, 3RUG, 3RUH, 3RUI, 3RUJ, 3RUK, 3RUM, 3RUN, 3RUO, 3RUP, 3RUQ, 3RUR, 3RUS, 3RUT, 3RUU, 3RUV, 3RUW, 3RUX, 3RUY, 3RUZ, 3RV0, 3RV1, 3RV2, 3RV3, 3RV4, 3RV5, 3RV6, 3RV7, 3RV8, 3RV9, 3RVA, 3RVC, 3RVD, 3RVF, 3RVG, 3RVH, 3RVI, 3RVJ, 3RVK, 3RVL, 3RVM, 3RVN, 3RVO, 3RVP, 3RVQ, 3RVR, 3RVS, 3RVT, 3RVU, 3RVV, 3RVW, 3RVX, 3RVY, 3RVZ, 3RW0, 3RW7, 3RW8, 3RW9, 3RWA, 3RWB, 3RWC, 3RWD, 3RWE, 3RWF, 3RWG, 3RWH, 3RWI, 3RWJ, 3RWK, 3RWL, 3RWM, 3RWN, 3RWO, 3RWP, 3RWQ, 3RWR, 3RWT, 3RWU, 3RWV, 3RWX, 3RX2, 3RX3, 3RX4, 3RX5, 3RX6, 3RX7, 3RX8, 3RX9, 3RXA, 3RXB, 3RXC, 3RXD, 3RXE, 3RXF, 3RXG, 3RXH, 3RXI, 3RXJ, 3RXK, 3RXL, 3RXM, 3RXO, 3RXP, 3RXQ, 3RXR, 3RXS, 3RXT, 3RXU, 3RXV, 3RXW, 3RXX, 3RXY, 3RXZ, 3RY0, 3RY1, 3RY2, 3RY3, 3RY4, 3RY5, 3RY6, 3RY7, 3RY8, 3RY9, 3RYA, 3RYB, 3RYC, 3RYD, 3RYE, 3RYF, 3RYH, 3RYI, 3RYJ, 3RYK, 3RYL, 3RYM, 3RYO, 3RYP, 3RYR, 3RYS, 3RYT, 3RYV, 3RYW, 3RYX, 3RYY, 3RYZ, 3RZ0, 3RZ1, 3RZ2, 3RZ3, 3RZ4, 3RZ5, 3RZ7, 3RZ8, 3RZ9, 3RZA, 3RZB, 3RZC, 3RZE, 3RZF, 3RZG, 3RZH, 3RZI, 3RZJ, 3RZK, 3RZL, 3RZM, 3RZN, 3RZP, 3RZS, 3RZU, 3RZV, 3RZW, 3RZX, 3RZY, 3RZZ, 3S00, 3S01, 3S02, 3S03, 3S04, 3S05, 3S06, 3S0A, 3S0B, 3S0C, 3S0D, 3S0E, 3S0F, 3S0G, 3S0H, 3S0I, 3S0J, 3S0K, 3S0M, 3S0N, 3S0O, 3S0P, 3S0Q, 3S0R, 3S0T, 3S0W, 3S0X, 3S0Y, 3S0Z, 3S11, 3S12, 3S13, 3S18, 3S19, 3S1A, 3S1B, 3S1C, 3S1D, 3S1E, 3S1F, 3S1G, 3S1H, 3S1I, 3S1J, 3S1K, 3S1L, 3S1S, 3S1T, 3S1U, 3S1V, 3S1W, 3S1X, 3S1Y, 3S1Z, 3S20, 3S21, 3S22, 3S23, 3S24, 3S25, 3S26, 3S27, 3S28, 3S29, 3S2A, 3S2C, 3S2E, 3S2F, 3S2G, 3S2I, 3S2J, 3S2K, 3S2L, 3S2M, 3S2N, 3S2O, 3S2P, 3S2Q, 3S2R, 3S2S, 3S2U, 3S2V, 3S2W, 3S2X, 3S2Y, 3S2Z, 3S30, 3S32, 3S33, 3S34, 3S35, 3S36, 3S37, 3S38, 3S39, 3S3A, 3S3B, 3S3C, 3S3D, 3S3E, 3S3F, 3S3G, 3S3H, 3S3I, 3S3J, 3S3K, 3S3L, 3S3M, 3S3N, 3S3O, 3S3P, 3S3Q, 3S3R, 3S3S, 3S3T, 3S3U, 3S3V, 3S3W, 3S3X, 3S3Y, 3S3Z, 3S40, 3S41, 3S42, 3S43, 3S44, 3S45, 3S46, 3S47, 3S48, 3S4A, 3S4B, 3S4C, 3S4D, 3S4E, 3S4F, 3S4J, 3S4K, 3S4L, 3S4M, 3S4O, 3S4Q, 3S4R, 3S4S, 3S4T, 3S4U, 3S4W, 3S4X, 3S4Y, 3S4Z, 3S51, 3S52, 3S53, 3S54, 3S55, 3S56, 3S57, 3S5A, 3S5B, 3S5C, 3S5D, 3S5E, 3S5F, 3S5H, 3S5I, 3S5J, 3S5K, 3S5L, 3S5M, 3S5N, 3S5O, 3S5P, 3S5Q, 3S5R, 3S5S, 3S5T, 3S5U, 3S5V, 3S5W, 3S5X, 3S5Y, 3S5Z, 3S60, 3S61, 3S62, 3S63, 3S64, 3S65, 3S66, 3S67, 3S68, 3S69, 3S6A, 3S6B, 3S6C, 3S6D, 3S6E, 3S6F, 3S6G, 3S6H, 3S6I, 3S6J, 3S6K, 3S6L, 3S6M, 3S6N, 3S6O, 3S6S, 3S6T, 3S6U, 3S6V, 3S6W, 3S6X, 3S6Y, 3S6Z, 3S70, 3S71, 3S72, 3S73, 3S74, 3S75, 3S76, 3S77, 3S78, 3S79, 3S7A, 3S7B, 3S7D, 3S7E, 3S7F, 3S7G, 3S7H, 3S7I, 3S7J, 3S7K, 3S7L, 3S7M, 3S7N, 3S7O, 3S7P, 3S7Q, 3S7R, 3S7S, 3S7T, 3S7V, 3S7W, 3S7X, 3S7Y, 3S7Z, 3S81, 3S82, 3S83, 3S84, 3S85, 3S86, 3S87, 3S88, 3S89, 3S8A, 3S8B, 3S8C, 3S8D, 3S8E, 3S8F, 3S8G, 3S8H, 3S8I, 3S8J, 3S8K, 3S8L, 3S8M, 3S8N, 3S8O, 3S8P, 3S8Q, 3S8R, 3S8S, 3S8V, 3S8W, 3S8X, 3S8Y, 3S8Z, 3S90, 3S91, 3S92, 3S93, 3S94, 3S95, 3S96, 3S97, 3S98, 3S99, 3S9A, 3S9B, 3S9C, 3S9D, 3S9E, 3S9F, 3S9G, 3S9H, 3S9I, 3S9J, 3S9K, 3S9L, 3S9M, 3S9N, 3S9O, 3S9Q, 3S9S, 3S9T, 3S9U, 3S9V, 3S9W, 3S9X, 3S9Y, 3S9Z, 3SA0, 3SA1, 3SA2, 3SA3, 3SA4, 3SA5, 3SA6, 3SA7, 3SA8, 3SA9, 3SAA, 3SAB, 3SAC, 3SAD, 3SAE, 3SAF, 3SAG, 3SAH, 3SAI, 3SAJ, 3SAL, 3SAM, 3SAN, 3SAO, 3SAP, 3SAQ, 3SAR, 3SAS, 3SAT, 3SAU, 3SAV, 3SAW, 3SAX, 3SAY, 3SAZ, 3SB0, 3SB1, 3SB2, 3SB3, 3SB4, 3SB5, 3SB6, 3SB7, 3SB8, 3SB9, 3SBA, 3SBB, 3SBC, 3SBD, 3SBE, 3SBF, 3SBG, 3SBH, 3SBI, 3SBJ, 3SBK, 3SBL, 3SBM, 3SBO, 3SBP, 3SBQ, 3SBR, 3SBS, 3SBT, 3SBU, 3SBW, 3SBX, 3SBY, 3SBZ, 3SC0, 3SC1, 3SC2, 3SC3, 3SC4, 3SC6, 3SC7, 3SC8, 3SCE, 3SCF, 3SCG, 3SCH, 3SCI, 3SCJ, 3SCK, 3SCL, 3SCM, 3SCN, 3SCO, 3SCP, 3SCQ, 3SCR, 3SCS, 3SCT, 3SCU, 3SCV, 3SCW, 3SCX, 3SCY, 3SCZ, 3SD0, 3SD2, 3SD4, 3SD5, 3SD6, 3SD7, 3SD8, 3SD9, 3SDA, 3SDB, 3SDC, 3SDD, 3SDE, 3SDF, 3SDG, 3SDH, 3SDI, 3SDJ, 3SDK, 3SDL, 3SDM, 3SDN, 3SDO, 3SDP, 3SDQ, 3SDR, 3SDS, 3SDT, 3SDU, 3SDV, 3SDW, 3SDX, 3SDY, 3SDZ, 3SE0, 3SE1, 3SE2, 3SE3, 3SE4, 3SE5, 3SE6, 3SE7, 3SE8, 3SE9, 3SEA, 3SEB, 3SEC, 3SED, 3SEE, 3SEF, 3SEI, 3SEJ, 3SEK, 3SEM, 3SEN, 3SEO, 3SEP, 3SEQ, 3SER, 3SES, 3SET, 3SEU, 3SEV, 3SEW, 3SEX, 3SEY, 3SEZ, 3SF0, 3SF4, 3SF5, 3SF6, 3SF8, 3SFC, 3SFD, 3SFE, 3SFF, 3SFG, 3SFH, 3SFI, 3SFJ, 3SFK, 3SFM, 3SFP, 3SFT, 3SFU, 3SFV, 3SFW, 3SFX, 3SFY, 3SFZ, 3SG0, 3SG1, 3SG2, 3SG3, 3SG4, 3SG5, 3SG6, 3SG7, 3SG8, 3SG9, 3SGA, 3SGB, 3SGC, 3SGD, 3SGE, 3SGG, 3SGH, 3SGI, 3SGJ, 3SGK, 3SGL, 3SGM, 3SGN, 3SGO, 3SGP, 3SGQ, 3SGR, 3SGS, 3SGT, 3SGU, 3SGV, 3SGW, 3SGX, 3SGY, 3SGZ, 3SH0, 3SH1, 3SH2, 3SH3, 3SH4, 3SH5, 3SH6, 3SH7, 3SH8, 3SH9, 3SHA, 3SHB, 3SHC, 3SHD, 3SHE, 3SHF, 3SHG, 3SHI, 3SHJ, 3SHL, 3SHO, 3SHP, 3SHQ, 3SHR, 3SHS, 3SHT, 3SHU, 3SHV, 3SHW, 3SHX, 3SHY, 3SHZ, 3SI0, 3SI1, 3SI2, 3SI3, 3SI4, 3SI5, 3SI6, 3SI7, 3SI8, 3SI9, 3SIA, 3SIB, 3SIC, 3SID, 3SIE, 3SIG, 3SIH, 3SII, 3SIJ, 3SIK, 3SIL, 3SIM, 3SIO, 3SIP, 3SIQ, 3SIR, 3SIS, 3SIT, 3SIW, 3SIX, 3SIY, 3SIZ, 3SJ3, 3SJ5, 3SJ6, 3SJ7, 3SJ8, 3SJ9, 3SJA, 3SJB, 3SJC, 3SJD, 3SJE, 3SJF, 3SJG, 3SJH, 3SJI, 3SJJ, 3SJK, 3SJL, 3SJM, 3SJN, 3SJO, 3SJP, 3SJQ, 3SJR, 3SJS, 3SJT, 3SJU, 3SJV, 3SJX, 3SJZ, 3SK0, 3SK1, 3SK2, 3SK3, 3SK4, 3SK5, 3SK6, 3SK7, 3SK8, 3SK9, 3SKA, 3SKB, 3SKC, 3SKD, 3SKE, 3SKF, 3SKG, 3SKH, 3SKJ, 3SKK, 3SKM, 3SKN, 3SKO, 3SKP, 3SKQ, 3SKS, 3SKU, 3SKV, 3SKX, 3SKY, 3SL0, 3SL1, 3SL2, 3SL3, 3SL4, 3SL5, 3SL6, 3SL7, 3SL8, 3SL9, 3SLA, 3SLB, 3SLC, 3SLD, 3SLE, 3SLF, 3SLG, 3SLH, 3SLI, 3SLJ, 3SLK, 3SLL, 3SLN, 3SLO, 3SLP, 3SLR, 3SLS, 3SLT, 3SLU, 3SLZ, 3SM0, 3SM1, 3SM2, 3SM3, 3SM4, 3SM5, 3SM8, 3SM9, 3SMA, 3SMB, 3SMC, 3SMD, 3SME, 3SMH, 3SMI, 3SMJ, 3SMK, 3SML, 3SMM, 3SMN, 3SMO, 3SMP, 3SMQ, 3SMR, 3SMS, 3SMT, 3SMV, 3SMZ, 3SN0, 3SN1, 3SN4, 3SN5, 3SN6, 3SN7, 3SN8, 3SN9, 3SNA, 3SNB, 3SNC, 3SND, 3SNE, 3SNF, 3SNG, 3SNH, 3SNI, 3SNK, 3SNL, 3SNM, 3SNN, 3SNO, 3SNS, 3SNV, 3SNX, 3SNY, 3SNZ, 3SO0, 3SO1, 3SO2, 3SO3, 3SO4, 3SO5, 3SO6, 3SO7, 3SO8, 3SO9, 3SOA, 3SOB, 3SOC, 3SOD, 3SOE, 3SOG, 3SOH, 3SOI, 3SOJ, 3SOK, 3SOL, 3SOM, 3SON, 3SOO, 3SOP, 3SOQ, 3SOR, 3SOS, 3SOT, 3SOU, 3SOV, 3SOW, 3SOX, 3SOY, 3SOZ, 3SP1, 3SP3, 3SP4, 3SP6, 3SP7, 3SP8, 3SP9, 3SPA, 3SPB, 3SPC, 3SPD, 3SPE, 3SPF, 3SPG, 3SPH, 3SPI, 3SPJ, 3SPK, 3SPL, 3SPR, 3SPS, 3SPT, 3SPU, 3SPV, 3SPW, 3SPX, 3SPY, 3SPZ, 3SQ0, 3SQ1, 3SQ2, 3SQ3, 3SQ4, 3SQ5, 3SQ6, 3SQ7, 3SQ8, 3SQ9, 3SQB, 3SQC, 3SQD, 3SQE, 3SQF, 3SQG, 3SQH, 3SQI, 3SQJ, 3SQL, 3SQM, 3SQN, 3SQO, 3SQP, 3SQQ, 3SQR, 3SQS, 3SQV, 3SQY, 3SQZ, 3SR0, 3SR1, 3SR2, 3SR3, 3SR4, 3SR5, 3SR6, 3SR7, 3SR9, 3SRA, 3SRB, 3SRC, 3SRD, 3SRE, 3SRF, 3SRG, 3SRH, 3SRI, 3SRJ, 3SRK, 3SRN, 3SRP, 3SRQ, 3SRR, 3SRS, 3SRT, 3SRU, 3SRV, 3SRW, 3SRX, 3SRY, 3SRZ, 3SS0, 3SS1, 3SS3, 3SS4, 3SS5, 3SS6, 3SS7, 3SS8, 3SS9, 3SSA, 3SSB, 3SSC, 3SSD, 3SSE, 3SSG, 3SSH, 3SSI, 3SSJ, 3SSK, 3SSL, 3SSM, 3SSN, 3SSO, 3SSP, 3SSQ, 3SSR, 3SSS, 3SST, 3SSU, 3SSV, 3SSW, 3SSX, 3SSY, 3SSZ, 3ST0, 3ST1, 3ST2, 3ST3, 3ST4, 3ST5, 3ST6, 3ST7, 3ST8, 3ST9, 3STA, 3STB, 3STC, 3STD, 3STE, 3STF, 3STG, 3STH, 3STI, 3STJ, 3STK, 3STL, 3STM, 3STN, 3STO, 3STP, 3STQ, 3STR, 3STT, 3STU, 3STV, 3STW, 3STX, 3STY, 3STZ, 3SU0, 3SU1, 3SU2, 3SU3, 3SU4, 3SU5, 3SU6, 3SU8, 3SU9, 3SUA, 3SUB, 3SUC, 3SUD, 3SUE, 3SUF, 3SUG, 3SUI, 3SUJ, 3SUK, 3SUL, 3SUM, 3SUN, 3SUO, 3SUP, 3SUQ, 3SUR, 3SUS, 3SUT, 3SUU, 3SUV, 3SUW, 3SUZ, 3SV0, 3SV1, 3SV2, 3SV3, 3SV4, 3SV5, 3SV6, 3SV7, 3SV8, 3SV9, 3SVA, 3SVB, 3SVC, 3SVD, 3SVE, 3SVF, 3SVG, 3SVH, 3SVI, 3SVJ, 3SVK, 3SVL, 3SVM, 3SVN, 3SVO, 3SVP, 3SVQ, 3SVR, 3SVS, 3SVT, 3SVU, 3SVV, 3SVW, 3SVZ, 3SW0, 3SW1, 3SW2, 3SW3, 3SW4, 3SW5, 3SW6, 3SW7, 3SW8, 3SW9, 3SWA, 3SWB, 3SWC, 3SWD, 3SWE, 3SWF, 3SWG, 3SWH, 3SWI, 3SWJ, 3SWK, 3SWL, 3SWM, 3SWN, 3SWO, 3SWP, 3SWQ, 3SWR, 3SWS, 3SWT, 3SWV, 3SWW, 3SWX, 3SWY, 3SWZ, 3SX0, 3SX1, 3SX2, 3SX3, 3SX4, 3SX5, 3SX6, 3SX7, 3SX8, 3SX9, 3SXA, 3SXB, 3SXC, 3SXD, 3SXE, 3SXF, 3SXG, 3SXH, 3SXI, 3SXJ, 3SXK, 3SXL, 3SXM, 3SXN, 3SXO, 3SXP, 3SXQ, 3SXR, 3SXS, 3SXT, 3SXU, 3SXV, 3SXW, 3SXX, 3SXY, 3SXZ, 3SY0, 3SY1, 3SY2, 3SY3, 3SY4, 3SY5, 3SY6, 3SY7, 3SY8, 3SY9, 3SYA, 3SYB, 3SYC, 3SYI, 3SYJ, 3SYK, 3SYL, 3SYM, 3SYN, 3SYO, 3SYP, 3SYQ, 3SYR, 3SYS, 3SYT, 3SYU, 3SYV, 3SYX, 3SYY, 3SYZ, 3SZ0, 3SZ1, 3SZ2, 3SZ3, 3SZ4, 3SZ5, 3SZ6, 3SZ7, 3SZ8, 3SZ9, 3SZA, 3SZB, 3SZC, 3SZD, 3SZE, 3SZF, 3SZG, 3SZH, 3SZI, 3SZJ, 3SZK, 3SZL, 3SZM, 3SZN, 3SZO, 3SZP, 3SZQ, 3SZR, 3SZS, 3SZT, 3SZU, 3SZV, 3SZW, 3SZY, 3SZZ, 3T00, 3T01, 3T02, 3T03, 3T04, 3T05, 3T06, 3T07, 3T08, 3T09, 3T0A, 3T0B, 3T0C, 3T0D, 3T0E, 3T0F, 3T0G, 3T0H, 3T0I, 3T0J, 3T0K, 3T0L, 3T0M, 3T0O, 3T0P, 3T0Q, 3T0R, 3T0S, 3T0T, 3T0U, 3T0V, 3T0W, 3T0X, 3T0Y, 3T0Z, 3T10, 3T11, 3T12, 3T13, 3T14, 3T15, 3T16, 3T19, 3T1A, 3T1B, 3T1C, 3T1D, 3T1E, 3T1F, 3T1G, 3T1I, 3T1K, 3T1L, 3T1M, 3T1N, 3T1O, 3T1P, 3T1Q, 3T1R, 3T1S, 3T1T, 3T1U, 3T1V, 3T1W, 3T1X, 3T20, 3T22, 3T24, 3T25, 3T26, 3T27, 3T28, 3T29, 3T2A, 3T2B, 3T2C, 3T2D, 3T2E, 3T2F, 3T2G, 3T2H, 3T2I, 3T2J, 3T2K, 3T2L, 3T2M, 3T2N, 3T2O, 3T2P, 3T2Q, 3T2S, 3T2T, 3T2U, 3T2V, 3T2W, 3T2X, 3T2Y, 3T2Z, 3T30, 3T31, 3T32, 3T33, 3T34, 3T35, 3T36, 3T37, 3T38, 3T39, 3T3A, 3T3C, 3T3D, 3T3E, 3T3F, 3T3G, 3T3H, 3T3I, 3T3J, 3T3K, 3T3L, 3T3M, 3T3N, 3T3P, 3T3Q, 3T3R, 3T3S, 3T3T, 3T3U, 3T3V, 3T3W, 3T3X, 3T3Y, 3T3Z, 3T40, 3T41, 3T42, 3T43, 3T44, 3T45, 3T46, 3T47, 3T48, 3T49, 3T4A, 3T4C, 3T4D, 3T4E, 3T4H, 3T4J, 3T4K, 3T4L, 3T4M, 3T4N, 3T4O, 3T4P, 3T4Q, 3T4R, 3T4S, 3T4T, 3T4U, 3T4V, 3T4W, 3T4X, 3T4Y, 3T4Z, 3T50, 3T51, 3T52, 3T53, 3T54, 3T55, 3T56, 3T57, 3T58, 3T59, 3T5A, 3T5B, 3T5C, 3T5D, 3T5E, 3T5F, 3T5G, 3T5H, 3T5I, 3T5J, 3T5K, 3T5L, 3T5M, 3T5O, 3T5P, 3T5S, 3T5T, 3T5U, 3T5V, 3T5W, 3T5X, 3T5Y, 3T5Z, 3T60, 3T61, 3T62, 3T63, 3T64, 3T65, 3T66, 3T67, 3T69, 3T6A, 3T6B, 3T6C, 3T6D, 3T6E, 3T6F, 3T6G, 3T6H, 3T6I, 3T6J, 3T6K, 3T6L, 3T6N, 3T6O, 3T6P, 3T6Q, 3T6R, 3T6S, 3T6U, 3T6V, 3T6W, 3T6X, 3T6Y, 3T6Z, 3T70, 3T71, 3T72, 3T73, 3T74, 3T77, 3T78, 3T79, 3T7A, 3T7B, 3T7C, 3T7D, 3T7E, 3T7F, 3T7G, 3T7H, 3T7I, 3T7J, 3T7K, 3T7L, 3T7M, 3T7N, 3T7O, 3T7P, 3T7Q, 3T7R, 3T7S, 3T7T, 3T7U, 3T7V, 3T7X, 3T7Y, 3T7Z, 3T80, 3T81, 3T82, 3T83, 3T84, 3T85, 3T86, 3T87, 3T88, 3T89, 3T8A, 3T8B, 3T8C, 3T8D, 3T8E, 3T8F, 3T8G, 3T8H, 3T8I, 3T8J, 3T8K, 3T8L, 3T8M, 3T8N, 3T8O, 3T8P, 3T8Q, 3T8R, 3T8S, 3T8T, 3T8U, 3T8V, 3T8W, 3T8X, 3T8Y, 3T90, 3T91, 3T92, 3T93, 3T94, 3T95, 3T96, 3T97, 3T98, 3T99, 3T9A, 3T9B, 3T9C, 3T9D, 3T9E, 3T9F, 3T9G, 3T9H, 3T9I, 3T9J, 3T9K, 3T9L, 3T9M, 3T9N, 3T9O, 3T9P, 3T9Q, 3T9T, 3T9U, 3T9V, 3T9W, 3T9X, 3T9Y, 3T9Z, 3TA0, 3TA1, 3TA2, 3TA3, 3TA4, 3TA5, 3TA6, 3TA7, 3TA8, 3TA9, 3TAB, 3TAC, 3TAD, 3TAE, 3TAF, 3TAG, 3TAH, 3TAI, 3TAJ, 3TAK, 3TAL, 3TAM, 3TAN, 3TAO, 3TAP, 3TAQ, 3TAR, 3TAS, 3TAT, 3TAU, 3TAV, 3TAW, 3TAX, 3TAY, 3TAZ, 3TB0, 3TB2, 3TB3, 3TB4, 3TB5, 3TB6, 3TB7, 3TB8, 3TB9, 3TBA, 3TBB, 3TBC, 3TBD, 3TBE, 3TBF, 3TBG, 3TBH, 3TBI, 3TBJ, 3TBK, 3TBL, 3TBM, 3TBN, 3TBO, 3TBS, 3TBT, 3TBV, 3TBW, 3TBY, 3TC1, 3TC2, 3TC3, 3TC5, 3TC6, 3TC7, 3TC8, 3TC9, 3TCA, 3TCE, 3TCF, 3TCG, 3TCH, 3TCI, 3TCJ, 3TCK, 3TCL, 3TCM, 3TCN, 3TCO, 3TCP, 3TCQ, 3TCR, 3TCS, 3TCT, 3TCU, 3TCV, 3TCX, 3TCY, 3TCZ, 3TD2, 3TD3, 3TD4, 3TD5, 3TD6, 3TD7, 3TD8, 3TD9, 3TDA, 3TDB, 3TDC, 3TDD, 3TDE, 3TDF, 3TDG, 3TDH, 3TDI, 3TDJ, 3TDK, 3TDL, 3TDM, 3TDN, 3TDO, 3TDP, 3TDQ, 3TDR, 3TDS, 3TDT, 3TDU, 3TDV, 3TDW, 3TDX, 3TDZ, 3TE0, 3TE1, 3TE2, 3TE3, 3TE4, 3TE5, 3TE6, 3TE7, 3TE8, 3TE9, 3TEA, 3TEB, 3TEC, 3TED, 3TEE, 3TEF, 3TEG, 3TEH, 3TEI, 3TEJ, 3TEK, 3TEL, 3TEM, 3TEN, 3TEO, 3TEP, 3TEQ, 3TER, 3TES, 3TET, 3TEU, 3TEV, 3TEW, 3TEX, 3TEY, 3TEZ, 3TF0, 3TF1, 3TF2, 3TF3, 3TF4, 3TF5, 3TF6, 3TF7, 3TF8, 3TF9, 3TFA, 3TFB, 3TFC, 3TFD, 3TFE, 3TFF, 3TFG, 3TFH, 3TFI, 3TFJ, 3TFK, 3TFL, 3TFM, 3TFN, 3TFO, 3TFP, 3TFQ, 3TFR, 3TFS, 3TFT, 3TFU, 3TFV, 3TFW, 3TFX, 3TFY, 3TFZ, 3TG0, 3TG1, 3TG2, 3TG3, 3TG4, 3TG5, 3TG6, 3TG7, 3TG8, 3TG9, 3TGA, 3TGB, 3TGC, 3TGD, 3TGE, 3TGG, 3TGH, 3TGI, 3TGJ, 3TGK, 3TGL, 3TGM, 3TGN, 3TGO, 3TGP, 3TGQ, 3TGR, 3TGS, 3TGT, 3TGU, 3TGV, 3TGW, 3TGX, 3TGY, 3TGZ, 3TH0, 3TH1, 3TH2, 3TH3, 3TH4, 3TH5, 3TH6, 3TH7, 3TH8, 3TH9, 3THA, 3THB, 3THC, 3THD, 3THE, 3THF, 3THG, 3THH, 3THI, 3THJ, 3THK, 3THM, 3THN, 3THO, 3THP, 3THQ, 3THR, 3THS, 3THT, 3THU, 3THV, 3THW, 3THX, 3THY, 3THZ, 3TI0, 3TI1, 3TI2, 3TI3, 3TI4, 3TI5, 3TI6, 3TI7, 3TI8, 3TI9, 3TIA, 3TIB, 3TIC, 3TID, 3TIE, 3TIF, 3TIG, 3TIH, 3TII, 3TIJ, 3TIK, 3TIM, 3TIN, 3TIO, 3TIP, 3TIQ, 3TIR, 3TIS, 3TIT, 3TIU, 3TIV, 3TIW, 3TIX, 3TIY, 3TIZ, 3TJ0, 3TJ1, 3TJ2, 3TJ3, 3TJ4, 3TJ5, 3TJ6, 3TJ7, 3TJ8, 3TJ9, 3TJA, 3TJB, 3TJC, 3TJD, 3TJE, 3TJF, 3TJG, 3TJH, 3TJI, 3TJJ, 3TJK, 3TJL, 3TJM, 3TJN, 3TJO, 3TJP, 3TJQ, 3TJR, 3TJS, 3TJT, 3TJU, 3TJV, 3TJX, 3TJY, 3TJZ, 3TK0, 3TK1, 3TK2, 3TK3, 3TK4, 3TK5, 3TK6, 3TK7, 3TK8, 3TK9, 3TKA, 3TKB, 3TKC, 3TKD, 3TKF, 3TKG, 3TKH, 3TKI, 3TKJ, 3TKK, 3TKL, 3TKM, 3TKN, 3TKP, 3TKQ, 3TKR, 3TKS, 3TKT, 3TKU, 3TKW, 3TKY, 3TKZ, 3TL0, 3TL1, 3TL2, 3TL3, 3TL4, 3TL5, 3TL6, 3TL8, 3TL9, 3TLA, 3TLB, 3TLC, 3TLD, 3TLE, 3TLF, 3TLG, 3TLH, 3TLI, 3TLJ, 3TLK, 3TLL, 3TLM, 3TLO, 3TLP, 3TLQ, 3TLR, 3TLS, 3TLT, 3TLU, 3TLV, 3TLW, 3TLX, 3TLY, 3TLZ, 3TM0, 3TM1, 3TM2, 3TM3, 3TM4, 3TM5, 3TM6, 3TM7, 3TM8, 3TM9, 3TMA, 3TMB, 3TMC, 3TMD, 3TME, 3TMG, 3TMH, 3TMJ, 3TMK, 3TML, 3TMM, 3TMN, 3TMO, 3TMP, 3TMQ, 3TMR, 3TMS, 3TMT, 3TMU, 3TMV, 3TMW, 3TMX, 3TMY, 3TMZ, 3TN0, 3TN1, 3TN2, 3TN3, 3TN4, 3TN5, 3TN6, 3TN7, 3TN8, 3TNB, 3TND, 3TNE, 3TNF, 3TNG, 3TNH, 3TNI, 3TNJ, 3TNK, 3TNL, 3TNM, 3TNN, 3TNO, 3TNP, 3TNQ, 3TNS, 3TNT, 3TNU, 3TNV, 3TNW, 3TNX, 3TNY, 3TNZ, 3TO0, 3TO1, 3TO2, 3TO3, 3TO4, 3TO5, 3TO7, 3TO8, 3TO9, 3TOA, 3TOB, 3TOC, 3TOD, 3TOE, 3TOF, 3TOG, 3TOH, 3TOI, 3TOJ, 3TOK, 3TOL, 3TOM, 3TON, 3TOP, 3TOQ, 3TOR, 3TOS, 3TOT, 3TOU, 3TOV, 3TOW, 3TOX, 3TOY, 3TOZ, 3TP0, 3TP1, 3TP2, 3TP3, 3TP4, 3TP5, 3TP6, 3TP7, 3TP8, 3TP9, 3TPA, 3TPB, 3TPC, 3TPD, 3TPE, 3TPF, 3TPI, 3TPJ, 3TPK, 3TPL, 3TPM, 3TPN, 3TPO, 3TPP, 3TPQ, 3TPR, 3TPS, 3TPT, 3TPU, 3TPV, 3TPW, 3TPX, 3TPY, 3TPZ, 3TQ0, 3TQ1, 3TQ2, 3TQ3, 3TQ4, 3TQ5, 3TQ6, 3TQ7, 3TQ8, 3TQ9, 3TQA, 3TQB, 3TQC, 3TQD, 3TQE, 3TQF, 3TQG, 3TQH, 3TQI, 3TQJ, 3TQK, 3TQL, 3TQM, 3TQN, 3TQO, 3TQP, 3TQQ, 3TQR, 3TQS, 3TQT, 3TQU, 3TQV, 3TQW, 3TQX, 3TQY, 3TQZ, 3TR0, 3TR1, 3TR2, 3TR3, 3TR4, 3TR5, 3TR6, 3TR7, 3TR8, 3TR9, 3TRB, 3TRC, 3TRD, 3TRE, 3TRF, 3TRG, 3TRH, 3TRI, 3TRJ, 3TRK, 3TRL, 3TRN, 3TRO, 3TRP, 3TRQ, 3TRR, 3TRS, 3TRT, 3TRU, 3TRV, 3TRW, 3TS1, 3TS3, 3TS4, 3TS5, 3TS6, 3TS7, 3TS8, 3TS9, 3TSA, 3TSB, 3TSC, 3TSD, 3TSE, 3TSF, 3TSG, 3TSH, 3TSI, 3TSJ, 3TSK, 3TSL, 3TSM, 3TSN, 3TSO, 3TSP, 3TSQ, 3TSR, 3TSS, 3TSU, 3TSV, 3TSW, 3TSX, 3TSY, 3TSZ, 3TT0, 3TT1, 3TT2, 3TT3, 3TT4, 3TT6, 3TT7, 3TT8, 3TT9, 3TTA, 3TTB, 3TTC, 3TTD, 3TTE, 3TTF, 3TTG, 3TTH, 3TTI, 3TTJ, 3TTK, 3TTL, 3TTM, 3TTN, 3TTO, 3TTP, 3TTQ, 3TTR, 3TTS, 3TTT, 3TTU, 3TTV, 3TTW, 3TTX, 3TTY, 3TTZ, 3TU0, 3TU1, 3TU3, 3TU4, 3TU5, 3TU6, 3TU7, 3TU8, 3TU9, 3TUA, 3TUB, 3TUC, 3TUD, 3TUE, 3TUF, 3TUG, 3TUH, 3TUI, 3TUJ, 3TUL, 3TUO, 3TUR, 3TUS, 3TUT, 3TUU, 3TUV, 3TUW, 3TUX, 3TUY, 3TUZ, 3TV0, 3TV1, 3TV2, 3TV3, 3TV4, 3TV5, 3TV6, 3TV7, 3TV8, 3TV9, 3TVA, 3TVC, 3TVD, 3TVI, 3TVJ, 3TVK, 3TVL, 3TVM, 3TVN, 3TVO, 3TVQ, 3TVR, 3TVT, 3TVU, 3TVV, 3TVW, 3TVX, 3TVY, 3TVZ, 3TW0, 3TW1, 3TW2, 3TW3, 3TW4, 3TW5, 3TW6, 3TW7, 3TW8, 3TW9, 3TWA, 3TWB, 3TWC, 3TWD, 3TWE, 3TWI, 3TWJ, 3TWK, 3TWL, 3TWM, 3TWO, 3TWP, 3TWQ, 3TWR, 3TWS, 3TWT, 3TWU, 3TWV, 3TWW, 3TWX, 3TWY, 3TWZ, 3TX0, 3TX1, 3TX2, 3TX3
[truncated: 262,880 more chars]
